# Artificial Intelligence Methods and Digital Intervention Strategies for Predicting and Managing Chronic Obstructive Pulmonary Disease Exacerbations: An Umbrella Review

**DOI:** 10.3390/healthcare13233037

**Published:** 2025-11-24

**Authors:** Marco Pozza, Nicolò Navarin, Vangelis Sakkalis, Silvia Gabrielli

**Affiliations:** 1 Department of General Psychology, University of Padova, 35131 Padova, Italy; 2 Digital Health Research, Fondazione Bruno Kessler, 38123 Trento, Italy; sgabrielli@fbk.eu; 3 Department of Mathematics, University of Padova, 35131 Padova, Italy; 4 Institute of Computer Science (ICS), Foundation for Research and Technology—Hellas (FORTH), 70013 Crete, Greece; sakkalis@ics.forth.gr

**Keywords:** COPD, exacerbation prediction, digital health interventions, low-cost wearable devices, artificial intelligence, interventions adherence, umbrella review

## Abstract

**Background**: Chronic Obstructive Pulmonary Disease (COPD) is a major global health burden in which acute exacerbations accelerate progression and increase hospitalizations. Emerging technologies, such as wearable biosensors, artificial intelligence (AI), and digital health tools, enable more proactive disease management. **Objectives**: This umbrella review synthesized evidence from systematic reviews and meta-analyses on (1) AI-driven prediction of COPD exacerbations using low-cost wearable biosignals, and (2) the effectiveness of digital health interventions on disease management, quality of life, and medication adherence. **Methods**: A systematic search of PubMed, Scopus, and Web of Science (2015–2025) identified eligible reviews. Methodological quality was assessed using AMSTAR-2, and study overlap was quantified with the Corrected Covered Area (CCA). A narrative synthesis was conducted across two research questions. Protocol registered in PROSPERO (CRD420251164450). **Results**: Twenty-seven reviews met the inclusion criteria. AI models demonstrated promising internal predictive accuracy but lacked external validation and clinical integration. Digital health interventions, such as mHealth applications and telerehabilitation, showed small to moderate improvements in quality of life and physical function. Reported effects varied considerably (OR = 0.20–2.37; I^2^ = 0–94%), indicating substantial heterogeneity across studies. Evidence for improvements in medication adherence and exacerbation reduction was inconsistent, and most included reviews were rated “Low” or “Critically Low” in methodological quality, limiting the generalizability of findings. **Conclusions**: AI and digital tools show strong promise for proactive COPD management, particularly through wearable-derived biosignals, outperforming traditional static assessments. However, their clinical readiness remains limited due to small-scale studies, interpretability challenges, inconsistent outcome measures, and a lack of external validation. To support real-world translation and regulatory adoption, future research must prioritize large-scale, rigorous, and equitable studies with standardized methodologies and robust generalizability testing.

## 1. Introduction

Chronic Obstructive Pulmonary Disease (COPD) is a progressive respiratory condition characterized by chronic airflow obstruction primarily resulting from long-term exposure to harmful substances, including cigarette smoke, environmental pollutants, and occupational hazards [1]. Clinically, COPD encompasses chronic bronchitis and emphysema [1], presenting symptoms such as persistent cough, sputum production, shortness of breath, and reduced physical capacity [2]. It represents a major global public health challenge, significantly impairing quality of life and resulting in considerable economic and social burdens [3]. According to recent World Health Organization (WHO) data [4], COPD accounted for approximately 3.5 million deaths globally in 2021 with projections indicating a rising trend driven by aging populations and continued exposure to environmental risk factors.

A critical aspect of managing COPD is the timely identification and prevention of Acute Exacerbations (AEs), episodes of sudden symptom worsening that often require emergency medical intervention and hospitalization [5]. Frequent exacerbations significantly contribute to rapid disease progression, deterioration of lung function, diminished quality of life, and increased mortality risk [6]. Consequently, effective COPD management involves proactive strategies aimed at predicting exacerbations before their clinical manifestation, allowing for early interventions to minimize their severity and impact [7].

Digital health encompasses a wide spectrum of technologies, including mobile health (mHealth), electronic health records (EHRs), health information technologies (HITs), wearable and ambient sensors, and telehealth platforms. These tools enable continuous monitoring, data sharing, and remote care delivery, offering new opportunities for chronic disease management such as COPD.

Recent advances in wearable biosensors and digital health platforms have enabled continuous, passive monitoring of physiological and behavioral data relevant to COPD management. Parameters such as respiratory rate, oxygen saturation (SpO_2_), activity level, and environmental exposure can now be captured in real time [8,9,10,11]. When analyzed using artificial intelligence (AI) techniques, including machine learning (ML) and deep learning (DL), these biosignals hold the potential for the development of predictive models that can anticipate exacerbations before overt clinical deterioration [12,13].

Yet predictive insights must be coupled with actionable interventions to be clinically meaningful. Digital health tools, including mobile apps, telemonitoring platforms, and remote coaching systems, support this need by facilitating self-management, personalized feedback, and timely communication between patients and providers [14,15]. These interventions seem to improve quality of life, enhance adherence to treatment, and reduce exacerbation frequency when integrated alongside standard care [16].

While numerous studies have explored digital innovations in COPD care, few have integrated the full continuum, which ranges from biosignal monitoring and AI-based prediction to digital intervention delivery. This umbrella review addresses that gap by synthesizing evidence across these domains, with a focus on scalable, low-cost technologies applicable in the real-world. We propose that this integrated framework represents a promising paradigm for anticipatory, personalized COPD management.

Despite substantial research on digital innovations in COPD care, most existing reviews remain domain-specific. For example, some focus on telemonitoring and wearable sensors for early exacerbation detection [17], others on AI-based prognostic modeling [18], or mHealth-supported self-management [19]. Broader syntheses have addressed digital health ecosystems [20] or adherence tools [21], while novel approaches like passive sound or speech monitoring are emerging [22]. However, these reviews generally examine isolated interventions without capturing the continuum from biosignal acquisition to AI prediction and digitally mediated response useful for researchers to develop more sophisticated solutions.

To our knowledge, this is an updated and comprehensive umbrella review that systematically synthesizes evidence across the full digital care pipeline for COPD, linking low-cost wearable-derived biosignals, AI-driven exacerbation prediction, and digital health interventions, thereby offering a comprehensive and practical perspective on technology-enabled COPD management.

Previous reviews have typically examined isolated aspects of COPD digital care, focusing either on AI-based prediction or on digital health interventions, but have not considered these domains within a unified framework. By integrating evidence across the entire digital-care continuum, from biosignal monitoring and predictive modeling to intervention delivery, this umbrella review provides a comprehensive analytical overview of how emerging technologies can be combined for proactive COPD management.

Guided by this rationale, the review addresses two primary research questions:

- RQ1: What is known about the use of wearable biosignals and AI for predicting COPD exacerbations, and what challenges have been reported in their application?
- RQ2: How do digital health interventions affect disease management, quality of life, and medication adherence in COPD patients compared to standard care?

The remainder of this paper is organized as follows: Section 2 describes the methods used for literature identification and synthesis. Section 3 presents findings for each research question. Section 4 offers a comprehensive discussion, and Section 5 concludes with key takeaways and directions for future research.

## 2. Materials and Methods

### 2.1. Study Design

This umbrella review was conducted in accordance with the Preferred Reporting Items for Systematic Reviews and Meta-Analyses (PRISMA) guidelines [23], and the best practices for conducting umbrella reviews developed by [24]. The process included defining research questions, systematically identifying and selecting relevant literature, extracting key information, and synthesizing findings across included reviews.

For each research question, the study selection followed the PICO (Patient, Intervention, Comparator, Outcome) model reported in Table 1.

The review protocol was retrospectively registered in PROSPERO (registration number: CRD420251164450) after the review was finalized; however, the registered protocol accurately reflects the methodology followed in this study minimizing potential registration bias.

### 2.2. Sources and Search Strategy

A comprehensive literature search was performed in three major databases (PubMed, Scopus, and Web of Science) for collecting peer-reviewed systematic reviews and meta-analyses published between 1 January 2015, and 1 March 2025. Two structured search queries (reported in Table 2) were developed using Boolean logic and keyword variants to directly correspond to the two research questions: (1) prediction of COPD exacerbations using AI and wearable biosignals, and (2) digital health interventions for COPD management. An overview of the database search strategy is presented in Table 2, while the complete search strings for each database (PubMed, Scopus, and Web of Science) are provided in Supplementary Table S10 to ensure transparency and reproducibility. Differences between the number of records retrieved in this review and those obtained when rerunning equivalent queries may occur due to ongoing database updates and variations in indexing or search algorithms across platforms.

### 2.3. Inclusion and Exclusion Criteria

Inclusion and exclusion criteria were defined in advance to maintain methodological consistency and keep the review focused on its core objectives. Only systematic reviews, and meta-analyses focusing on (1) wearable biosignals and AI-based models for COPD exacerbation prediction or (2) digital health interventions for disease management were considered eligible.

Studies had to be human-based, peer-reviewed, published in English between 2015 and 2025, and involve scalable, real-world applicable technologies, where scalability referred to the potential for broader implementation beyond research settings, and real-world applicability denoted feasibility and effectiveness in routine clinical care. Full criteria and screening codes are summarized in Table 3 and detailed in Supplementary Material Tables S1 and S2.

Two authors (M.P. and S.G.) independently performed a two-step screening process. In the initial phase, studies were screened based on their titles and abstracts. When eligibility could not be determined from this information alone, full-text articles were reviewed. After completing the screening, the two reviewers met to compare their evaluations and resolve any potential discrepancies. No major disagreements were identified. When uncertainties arose, they were discussed with N.N. and V.S. to ensure methodological consistency. A PRISMA flow diagram outlining the number of studies identified, screened, and included is provided in Section 3.

### 2.4. Data Extraction

Data extraction was conducted following a structured template developed by the research team before starting the extraction (see Tables S3 and S4 in the Supplementary Material for RQ1 and RQ2 data extraction, respectively). One author (M.P.) performed the initial data extraction from each eligible systematic review, while a second author (S.G.) independently verified the entries for completeness and accuracy. As in Section 2.3, discrepancies were resolved through discussion.

### 2.5. Quality Assessment

Following data extraction, M.P. and S.G. individually conducted separate evaluations of the methodological quality of the included reviews using AMSTAR-2, a widely accepted tool for appraising systematic reviews related to healthcare interventions which assesses key domains such as protocol registration, search strategy, bias assessment, and funding disclosure [25]. Inter-rater reliability was qualitatively assessed and found to be substantial, with no major discrepancies between reviewers. Therefore, no formal reconciliation or aggregation procedure was required. AMSTAR-2 final scores have been computed using the online tool developed by [25].

### 2.6. Study Overlap and Redundancy in Umbrella Reviews

Study overlap has been investigated using Corrected Covered Area (CCA) [26] available in the ccaR [27] R package version 0.1. CCA identifies the extent of primary study overlap within included reviews and helps mitigate risk of inflating certain results in narrative synthesis. CCA values were interpreted following [28] guidelines: 0–5% was classified as slight overlap, 6–10% moderate, 11–15% high, and >15% very high overlap.

This metric was used to interpret findings with appropriate caution where overlap was substantial, ensuring that redundant evidence did not bias conclusions. As this umbrella review synthesized only secondary data from published systematic reviews and meta-analyses, no reanalysis of primary data was conducted, consistent with umbrella-review methodology.

Summary values are reported in Section 3.2 (Table 5) while full overlap matrices are in the Supplementary Material (Tables S5 and S6 for RQ1 and RQ2, respectively).

### 2.7. Data Synthesis

Due to the substantial heterogeneity in intervention types, outcome definitions, and study designs, a narrative synthesis approach was adopted. Findings were organized and summarized separately for each research question and grouped by outcome domain (e.g., predictive performance, quality of life, medication adherence). When quantitative data were available, effect measures such as odds ratios (ORs) or standardized mean differences (SMD), together with heterogeneity statistics (I^2^), were extracted from the included meta-analyses and summarized descriptively. These indicators were used to illustrate the magnitude and variability of reported outcomes across reviews, and consistency of evidence was qualitatively assessed across studies. Given the methodological and statistical diversity of the included reviews, a quantitative synthesis (meta-meta-analysis) was not appropriate; results were therefore integrated narratively in accordance with current umbrella-review guidelines (Fernandez et al., 2025 [24]). Results were qualitatively grouped by intervention type (AI-based prediction vs. digital health interventions) and by outcome domain, with consideration of review quality to capture potential subgroup differences.

## 3. Results

**Figure 1:**
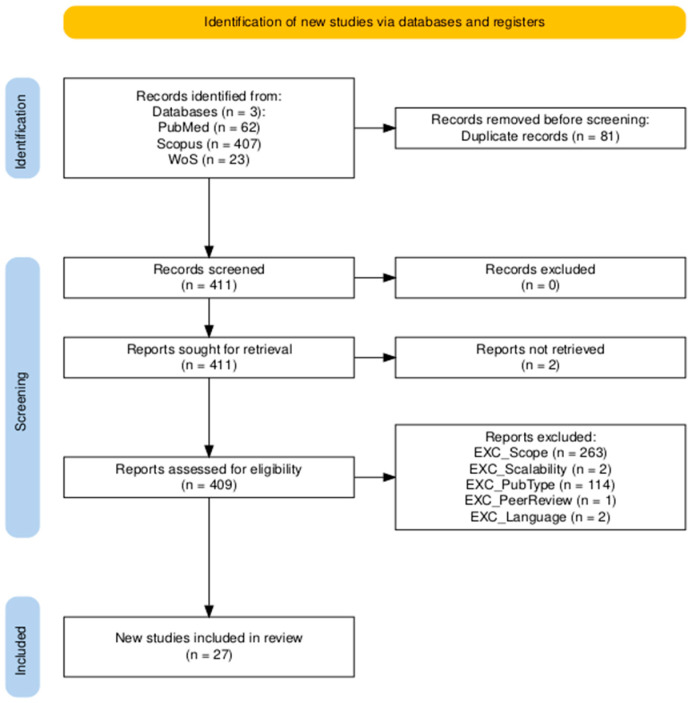
PRISMA flow diagram of the study selection process of this umbrella review, including the number of records identified, screened, assessed for eligibility, and included, along with reasons for exclusion at each stage.

A total of 27 systematic reviews and meta-analyses met the inclusion criteria. Five addressed AI-based prediction of COPD exacerbations using biosignals from wearable devices (RQ1), and 22 evaluated digital health interventions for disease management, quality of life, or medication adherence (RQ2). The selection process is summarized in Figure 1, and detailed exclusion rationale is provided in Supplementary Material Table S7. Most exclusions at the full-text screening stage were due to studies falling outside the scope of the review (EXC_scope = 263) or publication type (EXC_PubType = 114), indicating that few potentially relevant studies were missed and that the included set represents evidence saturation within the defined scope. Most included reviews focused on short-term prediction or intervention outcomes based on hospital or wearable-derived data, while only a few examined long-term monitoring or outpatient follow-up.

The details of the included systematic reviews are summarized in Table 6 for Q1 and Tables 7 and 8 for Q2. In terms of distribution, publications span from 2015 to 2025, with most (n = 21) appearing in the last five years, reflecting the growing interest and rapid development in digital health and AI applications for COPD care.

### 3.1. Quality Assessment and Risk of Bias Assessment

**Figure 2:**
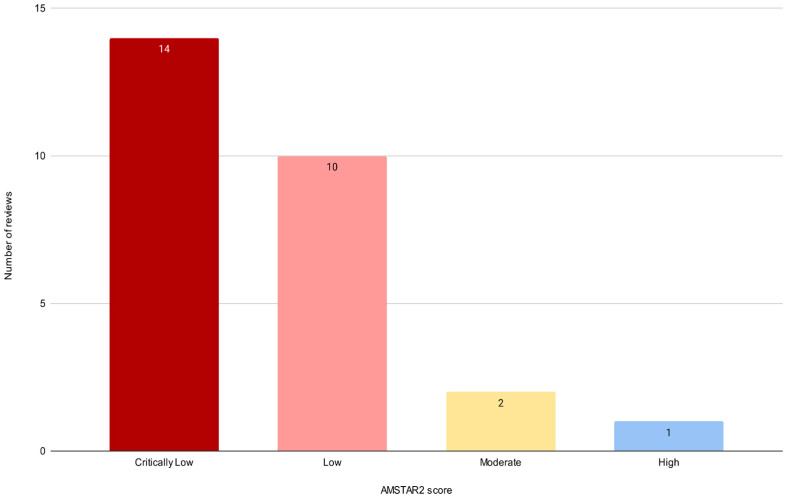
Distribution of methodological quality across included reviews according to the AMSTAR-2 assessment.

As anticipated in Section 2, the methodological quality of the 27 included reviews was evaluated using the AMSTAR-2 tool (Table 4) [25]. Based on this evaluation, only one review was rated as “High quality”, two as “Moderate”, and the vast majority as “Low” (n = 14) or “Critically Low” (n = 10). A visual summary of AMSTAR-2 ratings is provided in Figure 2, showing the distribution of reviews across quality categories (High, Moderate, Low, and Critically Low). Several critical weaknesses were common across reviews. Most notably, less than one-third (n = 7) reported a pre-registered protocol (Item 2), and only nine met the criteria for a comprehensive search strategy (Item 4). Transparency was also limited: only three reviews included a list of excluded studies with justification (Item 7), and few (n = 2) provided information on the funding sources of the studies they synthesized (Item 10). In addition, reviews often lacked structured methods for study selection (n = 23) or risk-of-bias analysis (n = 9), raising concerns about selective reporting or incomplete evidence capture. Consequently, although the included reviews offer valuable perspectives on digital innovation in COPD care, readers should interpret the results presented next with caution. Where conclusions are drawn from reviews rated as “Low” or “Critically Low” quality, they should be viewed as preliminary insights that highlight areas for further research rather than definitive findings.

### 3.2. Study Overlap

For RQ1, the overall CCA was 2.5%, reflecting slight overlap and suggesting that the included reviews largely relied on distinct sets of primary studies. However, one review pair showed a CCA of 15.1%, indicating very high overlap, likely due to a shared focus on similar technologies or predictive algorithms. RQ2 showed a similar pattern, with an overall CCA of 2.2% (slight overlap), but a maximum pairwise CCA of 23.8%, due to the repeated inclusion of widely cited trials on mobile apps and telerehabilitation. Despite these isolated cases of redundancy, the overall distribution of CCA values was skewed toward the lower end, reinforcing the uniqueness of most reviews’ evidence bases. These exceptions were accounted for in the interpretation but do not compromise the reliability of the synthesis which does not seem to be inflated by overlap of primary studies. Table 5 provides a summary estimate of redundancy in the evidence base for each question. A detailed citation matrix is also available in the Supplementary Material (Tables S5 and S6 for RQ1 and RQ2, respectively).

**Table 5:** CCA values for Research Questions 1 and 2, reflecting the aggregate degree of primary study overlap across included systematic reviews. These values provide a summary estimate of redundancy in the evidence base for each question.

RQ	# Reviews	N	r	c	CCA Proportion	CCA Percentage
1	5	152	135	5	0.0253	2.5%
2	22	532	359	23	0.0219	2.2%

RQ: research question; N: repeated study occurrences; r: unique primary studies; c: number of reviews; CCA Proportion: calculated overlap ratio; CCA Percentage: overlap as a percentage.

### 3.3. Q1: Predictive Models for COPD Exacerbations Using Biosignals and AI

#### 3.3.1. Overview of Populations and Data Sources

The systematic reviews included in this umbrella review covered a wide and clinically relevant range of COPD populations. These populations ranged from home-based individuals monitored remotely to patients hospitalized for acute exacerbations. Most studies focused on adults aged 65 to 75 with a confirmed COPD diagnosis. Participants were often selected because they had a high risk of exacerbations due to past episodes or comorbidities and the majority of samples exhibited a predominance of male participants. None of the studies included in the reviews examined gender-specific prediction dynamics; gender was occasionally reported as a predictor but without stratified analyses [29,32]. Additionally, the evidence summarized in the literature corpus came mainly from high-income countries, particularly the UK, Spain, and the US, and, to date, no models have been externally validated in low-income settings. AI models have been trained and tested on various data sources that included randomized controlled trials, hospital records, patient registries, and remote monitoring systems. Vital signs such as heart rate, respiratory rate, SpO_2_, FEV_1_, and FVC were collected by researchers using both clinical-grade devices (e.g., spirometers, oximeters) and wearable sensors. Many studies used wearables like Fitbit, Garmin, and ActiGraph GT3X to also gather behavioral data (e.g., physical activity, sleep, gait) [17,30,31]. While most of these devices are not specifically approved by regulation bodies for COPD diagnosis or treatment, they are widely used in research and remote monitoring contexts for early detection of exacerbations. Many studies in the literature corpus also used patient-reported outcomes collected through mobile apps like myCOPD [30] and Re-Admit [30]. Among them, some used standardized tools such as the COPD Assessment Test (CAT) [29] or the St. George’s Respiratory Questionnaire (SGRQ) [32]. Environmental data, including air quality and temperature, were also included in some studies using APIs like WAQI [30] and OpenWeather [30]. In terms of datasets, studies included in the reviews relied on both proprietary and public multimodal resources. Among the public ones, the most popular are the Capnobase TBME Respiratory Rate benchmark dataset (CapnoBase TBME RR) [30], the Methodist Environment for Translational Enhancement and Outcomes Research (METEOR) [30], and the Clinical Practice Research Datalink (CPRD) [30]. CapnoBase TBME RR [53] contains recordings from 42 subjects (13 adults; 29 children and neonates), each about 8 min long, collected during elective surgeries and routine anesthesia. It includes simultaneous ECG, PPG, and capnography (CO_2_ waveforms), as well as expert-annotated labels for pulse peaks and breaths. METEOR [54] is a clinical data warehouse within the Houston Methodist hospital system. It contains medical records of patients with COPD as the primary diagnosis, including demographics, comorbidities, lab data, vital signs, admission type, Rothman Index (a composite illness severity score from 26 physiological parameters) [55], medications, procedures, and admission complaints. CPRD [56] is a large longitudinal primary care EHR database in the UK, managed by MHRA and NIHR, with data from GP surgeries across the country. It covers approximately 60 million patients. The data are anonymized and include demographics, diagnoses, symptoms, prescriptions, lab tests, referrals, lifestyle factors (e.g., smoking, BMI), socioeconomic indicators, and more.

A complete overview of the data sources and their characteristics is provided in Table 6.

**Table 6:** RQ1 extracted data.

Study	Year	Type	# Studies	Population	Dataset	Data Type/Devices	AI Methods and Outcomes	Challenges
[17]	2024	SR	51	COPD frequent exacerbators, age 64–75, 40–45% female	Proprietary: TeleCare North, EDGE, Telescot	Wearables, spirometers, mHealth apps, point-of-care; biosignals (SpO_2_, RR, HR, CRP), patient-reported, environmental	Decision trees, RF, SVM, DNN, LSTM, RNN; AUROC ≤ 0.95, Sens. ≤ 99.4%, Acc. ≤ 97.4%. Strong predictive performance for early detection and hospitalization, but mixed real-world impact	No external validation; small sample sizes; inconsistent AECOPD labels
[29]	2024	SR/MA	46	AECOPD, age 60–80, 45–85% male; China (17), Spain (7), US (6), UK (5)	Multi-center hospital-based (ED, ICU, GIMD); no named cohorts	Clinical records, labs, symptom scores	Logistic regression (LASSO, stepwise); some ML (RF, XGBoost); AUC 0.80 (mortality), 0.84 (hospitalization)	98% models high bias; limited external validation; poor calibration and missing data handling
[30]	2025	SR	41	COPD all severities; age~60–70, ~65% male (when reported); samples: 16–1000 s	Public: Capnobase, METEOR, CPRD, Freesound, Respiratory DB; Proprietary: myCOPD, Re-Admit, DmD, EDGE	Biosignals, lung function, sounds, saliva, PROs, lifestyle/environmental; wearables, apps, sensors	SVM, RF, boosting, CNN, LSTM, GRU; Diagnostic Acc. 80.67–97%, Exacerbation AUROC ≤ 0.958	Poor external validation; small datasets; low interpretability; underuse of environmental/lifestyle data
[31]	2016	SR	20	Elderly COPD (frequent exacerbators), small samples (5–169); EU, US, CAN, AUS	Study-specific proprietary data	Symptoms, biosignals, lung function; sensors, e-diaries, oximeters, spirometers	Naïve Bayes, SVM, BNN, KNN, clustering; COPD AUC ≤ 0.84, Asthma AUC 0.59–0.73; exacerbation prediction 3–5 days ahead	Heterogeneous labels, small samples, no external validation, poor usability
[32]	2017	SR	25	Severe COPD (GOLD II–IV), global; mostly male (>80%)	Public: ECLIPSE, BODE; mostly proprietary	Demographics, spirometry, biomarkers (CRP, IL-6), PROs	Logistic/Cox regression; 1 RF model; AUC 0.58–0.85 (mostly 0.60–0.75)	Weak validation (only 4/27); inconsistent predictors/outcomes; no modern data (wearables, deep learning)

RQ1 column definition: Study—Reference to the review article; Year—Year the review was published; Type—Specifies the type of review (e.g., Systematic Review—SR, Meta-analysis—MA or both—SR/MA); # Studies—Total number of primary studies synthesized in the review; Population—Description of the patient population (e.g., COPD severity, age range, geographic focus); Dataset—Names or sources of datasets referenced in the included studies; Data Type/Device—General types of data and technologies used, including biosignals, clinical records, patient-reported outcomes, and devices such as wearables, mobile apps, or remote monitoring tools; AI Techniques, Inputs and Outcomes—Summary of AI/ML methods, prediction goals, model inputs, performance metrics, validation approaches, and key outcomes, including techniques used (e.g., supervised learning), prediction targets (e.g., exacerbations, hospitalizations), input features (e.g., vitals, symptoms), performance measures (e.g., AUC, accuracy), type of validation, and overall findings or effect estimates; Main Challenges/Limitations—Limitations or implementation challenges identified by the review authors. For an extended, more detailed, version of the extracted data, see Supplementary Material Table S8.

#### 3.3.2. AI Models and Performance

##### AI Architectures, Input Features, and Prediction Targets

The body of evidence synthesized in the included reviews covered a wide array of AI models designed to predict COPD exacerbations. These included traditional statistical approaches, classical ML techniques, and advanced DL architectures. Common ML models included Support Vector Machines (SVM), boosting algorithms (e.g., XGBoost, AdaBoost, LightGBM, CatBoost), Random Forests (RF), Logistic Regression (LR), and Decision Trees (DT). RF and DT models were instead favored by other researchers for their interpretability and resilience to noise, such as biosignals or cough acoustics [17,29]. On the other hand, DL models were increasingly used as datasets became larger and more complex. Convolutional Neural Networks (CNNs) were the most commonly applied, often paired with temporal architectures such as Long Short-Term Memory (LSTM) networks and Recurrent Neural Networks (RNNs). Hybrid solutions such as ConvLSTM and Spatio-Temporal Artificial Intelligence Networks (STAIN), were developed to jointly model spatial and temporal dependencies in multimodal inputs such as respiratory waveforms, physical activity, and video-based monitoring data [17,30]. Other researchers applied Bayesian networks to support probabilistic reasoning and improve interpretability, particularly in the presence of missing data. Some also used Markov Chain Monte Carlo (MCMC) methods for imputation and feature selection [17]. Despite relying on similar architectures, prediction targets varied across studies but primarily focused on acute exacerbations, hospital readmission, ICU admission, and mortality. Some works targeted composite outcomes such as time to next exacerbation or mechanical ventilation risk. As discussed in [29], some groups also differentiated between short-term predictions (e.g., within 30 days) and longer-term forecasts (e.g., over 6 or 12 months). Beyond prediction targets, considerable variation was also observed in the input features used by the models. Core clinical variables, such as age, BMI, comorbidities, medication history, and baseline pulmonary function, were frequently used, especially in models based on clinical records or hospital datasets [29,32]. In contrast, those developed on remote monitoring datasets often integrated patient-reported symptoms (e.g., breathlessness, cough, fatigue) collected via mobile application. Behavioral indicators like step count, sleep duration, and physical activity were instead common in studies using longitudinal home-monitoring data [17,30]. As anticipated in Section 3.3.1, some models went further, incorporating environmental variables (e.g., temperature, air quality), or extracting higher-order features from biosignals, such as wavelet transforms of respiratory sounds, oxygen saturation trends, or accelerometer-derived motion patterns [30]. Certain variables repeatedly emerged as strong predictors, although their relevance often depended on the dataset and prediction target. For short-term exacerbation risk, SpO_2_ consistently ranked among the most informative features, sometimes surpassing heart rate and respiratory rate [17]. In contrast, physical activity indicators, such as daily step count from accelerometers, were more often linked to hospital readmission risk during the post-discharge phase. Body weight and skin temperature, when available, were mainly incorporated into models addressing longer-term outcomes rather than imminent exacerbations [17,29].

##### Reported Model Performance and Challenges of Comparative Evaluation

Validation methods varied widely among the models. Most studies of the literature corpus relied on internal techniques, such as k-fold cross-validation, bootstrapping, or holdout splits, while only a smaller subset applied temporal validation, where training and test sets were split by time, better reflecting real-world deployment in remote monitoring contexts [32]. While internal validation strategies help reduce overfitting and serve as a preliminary check of model reliability, they do not test generalizability to independent data. Model evaluation across clinically relevant subgroups, such as gender, disease severity, or comorbidities was lacking. Additionally, only a few studies assessed model calibration, further limiting interpretability in clinical settings [32]. Finally, model development workflows often lacked pre-specified hyperparameter tuning protocols, raising the risk of a posteriori optimization and overly optimistic performance estimates [29]. Overall, these methodological limitations highlight that reported model performance cannot be interpreted in isolation. The characteristics of the datasets themselves were just as influential, shaping both the accuracy achieved and the extent to which findings could be generalized. As summarized in Table 6, the reviewed studies drew on public, proprietary, and hospital-based datasets, each with distinct advantages and drawbacks. Public datasets such as CPRD, METEOR, and CapnoBase were generally employed for long-term risk prediction, including hospitalization, readmission, or symptom progression. These EHR-based resources offered scale and demographic breadth but lacked dynamic or wearable inputs. CapnoBase was even more limited, containing only short respiratory recordings suitable for classification but not longitudinal modeling. Not surprisingly, performance was modest: AUROCs ranged from 0.58 to 0.75 [17], constrained by coarse symptom granularity, limited physiological coverage, and conservative endpoints such as mortality. Without real-time or patient-reported data, these models were less sensitive to early exacerbation detection. In contrast, proprietary datasets such as myCOPD, Re-Admit, DmD, and EDGE enabled multimodal integration of self-reports, wearable biosignals (SpO_2_, HR, RR), environmental exposures, and spirometry. These richer data streams supported short-term exacerbation prediction in remote monitoring settings and yielded some of the most optimistic AUROCs, approaching 0.95 with deep learning methods like DNNs, LSTMs, and Bayesian networks [17,30]. Several studies even reported very high specificity (97.7%), sensitivity (99.4%), and accuracy (97.4%) [30]. However, symptom-counting methods achieved more modest AUROCs (~0.74) [17], and even high-performing proprietary tools such as ACCESS and COPDPredict often drew on fewer than 100 participants, used internal validation only, and applied inconsistent exacerbation definitions [17,30]. These limitations temper confidence in their real-world applicability. Hospital-based datasets, discussed in [29], were oriented toward predicting severe adverse outcomes during AECOPD episodes, including mortality, ICU admission, and respiratory failure. Using clinical indicators, vital signs, and comorbidity-based scores such as DECAF, PEARL, and MAGENTA, models achieved pooled AUROCs of 0.80–0.84 with solid internal discrimination. Yet, when tested externally, performance often declined substantially (e.g., 0.75 to 0.66), underscoring the challenges of transferring models across patient populations and healthcare settings without adaptation. Taken together, these findings show that dataset characteristics—granularity, labeling practices, and population biases—were decisive in shaping performance. Real-time signals and patient-reported data clearly improved early detection but were underrepresented in most datasets [30]. Exacerbation definitions varied from symptom diaries to hospitalization events, complicating comparability [17,29]. Small datasets often forced deep learning models to rely on augmentation, heightening overfitting risks [30]. Furthermore, digital health platforms tended to overrepresent tech-savvy patients with milder disease, while hospital datasets overrepresented acute cases, creating systematic biases [17,29]. Beyond dataset characteristics, algorithm choice also influenced reported outcomes. When different approaches were applied to the same dataset, deep learning methods such as CNNs or LSTMs occasionally outperformed classical models like SVMs or random forests. However, these gains were inconsistent and often task-dependent [30], reflecting sensitivity to hyperparameter settings, insufficient tuning protocols, and the limited expressiveness of small training sets [17,30].

### 3.4. Q2: Impact of Digital Health Interventions on COPD Management, Quality of Life, and Medication Adherence

#### 3.4.1. Study Populations, Intervention Types, and Comparators

The study populations across the systematic reviews for Q2 consisted of adults with clinically confirmed COPD, most diagnosed by spirometry and classified according to GOLD criteria (Table 7 and Table 8). As in Q1, most participants were between 60 and 75 years old, with mean ages typically ranging from 62 to 70. Many cohorts were male-dominated, with over 60% men in several studies and some reporting male representation above 90% [47]. Most trials enrolled patients with moderate to very severe COPD (GOLD II–IV), while those with mild disease (GOLD I) were seldom included. Reporting on functional impairment, dyspnea severity, and comorbidity burden was inconsistent. Cardiovascular disease, diabetes, and depression were common comorbidities, but uneven documentation limited stratification by baseline health status. As seen for AI models for COPD, geographic distribution was heavily weighted toward high-income countries. Studies were most often conducted in Europe, North America, Australia, and South Korea, with frequent contributions from the US, the Netherlands, Spain, and the UK. In contrast, evidence from low- and middle-income countries was nearly absent, despite these regions carrying a disproportionate share of the global COPD burden. Several reviews also underscored the lack of detailed reporting on social determinants of health, digital literacy, and access to enabling technologies such as smartphones and internet connectivity. These factors are central to intervention uptake and long-term adherence, particularly among older adults and socioeconomically disadvantaged groups.

##### Digital Interventions: Types, Delivery and Comparators

The reviews identified a wide range of digital health interventions for COPD, varying in clinical focus, complexity, and integration within care systems. These included mHealth applications, telemonitoring platforms, web-based self-management portals, automated messaging tools, and video-supported pulmonary rehabilitation (PR). Many programs combined several modalities in hybrid designs. mHealth applications were the most frequent, offering symptom tracking, medication reminders, inhaler guidance, educational modules, and behavior-change features such as push notifications or gamification [19,35,37]. Telemonitoring platforms, often linked to smartphones or tablets, captured physiological data (e.g., SpO_2_, respiratory rate) for clinician review and intervention [37,41]. Web-based portals emphasized education, breathing exercises, nutrition support, and coaching [42]. Lower-intensity tools such as SMS, IVR, or chatbots reinforced adherence and symptom awareness in older or digitally underserved groups [21,42]. Video-based PR, particularly during the COVID-19 pandemic, delivered structured exercise and education with optional clinician feedback [38]. Delivery models ranged from self-directed tools, often limited by poor adherence, to hybrid approaches that included periodic video consultations with nurses or physiotherapists, which showed stronger engagement and functional benefits [37,38]. A smaller subset of fully integrated telehealth systems provided real-time monitoring and alerts embedded in clinical workflows [43,44]. Interventions also varied by synchrony: live consultations and supervised sessions offered immediate feedback but required substantial resources, while asynchronous models such as daily logs or retrospective reviews were more scalable but less responsive. Across studies, multi-modal and interactive formats combining monitoring, education, and communication achieved the most consistent clinical and behavioral gains [35,38,46]. Comparator conditions were equally heterogeneous. “Usual care” was the most common, but its scope ranged from minimal physician follow-up and prescriptions [19,39,47] to comprehensive pulmonary rehabilitation and individualized management plans in well-resourced settings [36,45]. Other comparators included face-to-face rehabilitation, behavioral counseling, or educational sessions; paper-based action plans and self-management guides; and waitlist or no-intervention controls. Hybrid comparators, such as standard care supplemented with non-digital follow-up calls or printed materials were also frequent. A few multi-arm trials contrasted digital interventions with both in-person rehabilitation and general education, or compared remote monitoring with self-monitoring and conventional care, clarifying the role of technology and clinician oversight.

#### 3.4.2. Outcomes of Digital Interventions for COPD

Digital health interventions for COPD were evaluated across a range of clinical and behavioral outcomes, most commonly health-related quality of life (QoL), functional capacity, exacerbation frequency, hospital utilization, and medication adherence. Overall, findings were mixed but generally supported digital tools as adjuncts to standard care. QoL was the most frequently assessed endpoint, measured with instruments such as CAT, SGRQ, CRQ, and EQ-5D. Several reviews reported small to moderate improvements favoring digital interventions, particularly those incorporating education, behavioral prompts, or physical activity support. Yet effect sizes often fell below minimal important differences, and heterogeneity remained high [36,37,40]. Within this context, multi-component models, especially telerehabilitation or hybrid formats, emerged as the most consistently beneficial. Similar patterns were observed for functional capacity. When evaluated with the 6MWT, ISWT, or daily step count, moderate gains were noted in several reviews, particularly when structured exercise and feedback were embedded in the intervention [37,38]. As with QoL, however, results varied depending on patient engagement, intervention duration, and follow-up length. Evidence for exacerbation rates and hospital admissions was less consistent. Some studies reported fewer exacerbations or emergency visits, especially among high-risk populations or in post-discharge settings [51,52]. Others did not confirm these benefits, and comparability was limited by inconsistent definitions of exacerbation, ranging from symptom worsening to formal clinical diagnosis. Medication adherence was examined less frequently but showed a comparable trend. Interventions that incorporated reminders, feedback, or automated alerts generally yielded modest improvements, though adherence was often inferred from indirect metrics such as app usage rather than validated measures [21,42]. Additional outcomes, including self-efficacy, dyspnea, and psychological wellbeing (anxiety, depression), were reported only sporadically. Where improvements were observed, they were more common in multi-modal, interactive interventions. However, these outcomes were typically assessed through qualitative or non-standardized measures, limiting confidence in the findings.

**Table 7:** RQ2 extracted data for population, intervention and comparator.

Study	Year	Type	Design	P—Population	I—Intervention	C—Comparator
[33]	2025	SR/MA	26—RCTs	Chronic disease pts/Mostly CVD, diabetes, COPD/Also stroke, asthma, CKD, AIDS, cancer/Mean age: 50–60s	mHealth via apps: med reminders, disease education (incl. video), symptom tracking, feedback alerts, HCP chat	Usual care: clinic visits, verbal/print education, non-digital adherence support (e.g., brochures, calls)
[34]	2021	SR/MA	14—RCTs	Adults w/COPD (mild–very severe)/Mean age 65–72/Mostly male (48–100%)	Digital (single/multi): apps, web, AV tools/Self-monitoring, breathing, education/Some via Skype or hybrid	Usual care: in-person visits, meds, emergencies/Some structured care, no digital tools
[19]	2020	SR/MA	13—RCTs	COPD pts, moderate–severe/Age 57–74/Balanced gender/Studies in NL, UK, US, China	18 trials: 11 smartphone apps, 2 tablet-based, 5 w/wearables (e.g., pedometers, oximeters)	Usual care: visits, meds, PR, verbal/leaflet education/No app or telemonitoring access
[21]	2021	SR	13—RCTs	Adults w/COPD or asthma/Asthma: age 30–50/COPD: age 65–75/Female %: 6.7–76.5%	Telemonitoring, IVR, SMS, web-based platforms/Delivered via phone, web, tablet, robots, email	Usual care: GP/specialist visits, verbal/print education, meds, some face-to-face counseling
[35]	2023	SR/MA	130—RCTs	Adults w/≥1 chronic condition/Mean age 61/41.6% female/Mostly IHD, HF, diabetes	mHealth (57), eHealth (29), devices (14), combos: mHealth + device (22), eHealth + device (6), all (2)	Usual care: standard management, in-person follow-ups, no digital tools or platforms used
[36]	2024	SR/MA	10—RCTs	COPD pts (FEV1/FVC < 70%)/GOLD stage/stable/Age 62–72/Mostly male/8 countries	mHealth apps (7–10 studies): rehab w/exercise modules/myCOPD, WeChat used in 3 studies	Usual PR: in-person, supervised exercise/Mostly hospital-based/No digital tools used
[37]	2024	SR/MA	51—RCTs	Stable COPD/Age 51–83 (mean 60–75)/Mostly male, gender mix varied across studies	6 types: telemonitoring, apps, web, calls, VR, hybrid/Duration: 12–56 weeks	Usual care: meds, in-clinic follow-up, in-person PR, non-digital education/no tech use
[38]	2024	SR	13—RCTs	Adults with COPD/Severity: mild–very severe/N = 34–409/Age: 44–75 (mean 60–70s)/Mostly male	Digital PR: web, apps, video/Exercise (endurance, strength, breathing), education, self-management, motivation	In-person PR: 2–3× weekly sessions/Exercise + education/Usual care: meds + lifestyle advice
[39]	2024	SR	35—RCTs (27), Pilot (1), Feasibility study (3), Cluster-RCTs (1), Non-RCTs (3)	Adults w/chronic diseases (e.g., T2DM, CVD, cancer, COPD)/Poor control or recent events	Digital PR: web-based platforms, apps, videoconferencing, structured online PR programs	Standard care: in-person visits, consults, print education, non-interactive tools, passive monitoring
[40]	2025	SR/MA	17—RCTs	Adults 62–74 y/Moderate–severe COPD/Multi-morbidity, ↓ mobility/N = 17–375/12 countries incl. USA, China, UK	Digital COPD care: self-mgmt tools (tracking, reminders, coaching, AI alerts), telerehab, remote HCP comms	Usual care: in-person rehab, GP/home visits, no digital tools/Some added non-digital resources
[41]	2024	SR/MA	10—RCTs	Adults w/moderate–severe COPD/Some w/frequent exacerbations/Age: 63–71/No gender data	Telehealth: mHealth, web, tablets/COPD education, PR, symptom + vital monitoring/HCP/peer comms/3–12 mo	Usual care: clinic follow-up (physicians/nurses)/Printed education: inhaler use, symptom mgmt
[42]	2025	SR	13—12 RCTs and 1 comparative cohort study	Adults w/moderate–severe COPD/Stable, post-hospital, or frequent exacerbators/Mean age 67/61% male	mHealth: apps (med reminders, symptom/activity tracking, feedback)/SMS (reminders, HCP chat)	Standard care: GP/specialist visits, meds per guidelines, occasional booklets/No digital elements
[43]	2024	SR	35—RCTs	Adults w/COPD, asthma, or both/COPD: mod–very severe/Some ≥1 exacerbation/year/Mean age: 6–73 yrs	Digital tools: real-time monitoring, teleconsults, e-diaries/Data via Bluetooth/Alerts + auto feedback	Usual care: clinic visits, med reviews, exams/Manual symptom logs/No telemonitoring or digital tools
[44]	2024	SR/MA	21—RCTs	COPD primary focus/Also bronchiectasis, ILD/Ages: mid-50–70s/Mostly male	Telerehab: video sessions, AI platforms, phone coaching, wearables for real-time exercise feedback	Usual PR: in-clinic aerobic, resistance, breathing exercises + education/Hospital or outpatient settings
[45]	2023	SR	32—RCTs (23), quasi-experimental (4), observational/cohort (4), qualitative (1)	Adults with stable COPD/No severity limits/Mostly >40 yrs/Mixed gender	Mobile apps, SMS, phone monitoring, web portals, tablet PR apps for COPD management	Usual meds only/Waitlist controls/Some single-arm studies focused on feasibility or adherence
[46]	2022	SR/MA	22—RCTs	COPD pts > 40/Mild–mod severity/59% male/Mean age 62–75/Studies: 11 countries	Telerehab: async (wearables, apps, web) + sync (live video PR)/Remote clinician monitoring	Usual care or wait-list/No structured rehab/Only standard clinical follow-up, no exercise training
[47]	2023	SR/MA	6—RCTs	Adults w/COPD/Mostly moderate–very severe/3 studies unstated severity/Mean age: 62.7–73.5/Mostly male	Web-based self-mgmt via portals, apps, WeChat, EHR tools, web call centers	Usual care: clinic visits, meds, verbal advice/2 studies: print/face-to-face education, no interactivity
[48]	2017	SR	8—RCTs (5), non-RCTs (3)	Adults with mild–moderate COPD/Mean age: 62–72/65–100% male	Smartphone-based PA tools: step count, visual feedback, texts/PA promo or exercise training	Usual care: standard COPD tx/Verbal advice to walk more/No structured PA or behavior support
[49]	2017	SR/MA	3—RCTs	Adults w/COPD/Severity not reported/Mean age: 67 (Moy), 66 (Tabak), 58 (Voncken-Brewster)	Moy: 12-mo web walking prog/Pedometer, dashboard, goals, peer forum/Step uploads weekly	Moy: Pedometer only, no goals/feedback/Tabak: PR, no app/VB: Nurse coaching, no tech
[50]	2015	SR/MA	9—RCTs	Moderate–severe COPD/Mean age: 64–73/34% women/Sites: NA, Europe, Asia	Home-based: phone calls (edu, motivation), web (symptom reporting, tailored edu, live chat)	Usual care or brief education/Home exercise w/print guides/No digital reinforcement
[51]	2016	SR/MA	6—RCTs (5), quasi-randomized controlled trial (1)	Adults w/moderate–severe COPD/Some w/full COPD spectrum/Mean age: 70/74% male	Smartphone-based COPD self-management: symptom + physio tracking, exercise programs, progress feedback	Routine care: meds, follow-up visits, education/training, pt-initiated contact during symptom worsening
[52]	2021	SR/MA	22—RCTs	Adults w/severe COPD/Some on home O2/Mean age 63–81/Male: 30–70%	Telemonitoring via web portals, mobile/tablet/PDA devices, some w/video conferencing	Usual care: clinical follow-up, guideline-based meds, emergency access, no telemonitoring

Study—Reference to review article; Year—Publication year of the review; Type—Review classification: Systematic Review (SR), Meta-analysis (MA), or both (SR/MA); Study Design—Type and total number of studies included; P—Population: characteristics of included participants (e.g., age, sex, disease severity, comorbidities); I—Digital Intervention: modality (e.g., mHealth, telemonitoring, web-based), key components, features, and intervention duration; C—Comparator: description of control conditions (e.g., usual care, routine clinical follow-up, non-digital interventions). Symbols—↓: negative or adverse effect. For extended, detailed data, see Supplementary Material Table S9.

**Table 8:** RQ2 extracted data for outcomes, heterogeneity and limitations.

Study	O—Outcomes	Heterogeneity	Limitations
[33]	Med adherence ↑ (OR 2.37; SMD 0.28) via self-report (MMAS-8, MAQ, Voils, MG) and electronic tracking; no QoL data; symptoms tracking limited; ↑ effect w/advanced reminders, interactivity, data sharing, dispensers	I^2^ = 0–85%/↑ in self-report tools/↓ w/interactive features/Random-effects model	Heterogeneity/Reporting gaps/High–unclear bias/Low quality/Publication bias
[34]	6MWD ↑ (MD = 54.33 m)/QoL ↑ (SGRQ −26.57, CAT/EQ-5D~)/Self-efficacy~/CRQ-dyspnoea ↑ (MD = 0.64)/Exacerbation, admission~/Satisfaction mixed/Adherence ✗	I^2^ = 0–87%/↑ in 6MWD (87%)/QoL varied/Low in dyspnoea, self-efficacy/Some not pooled	Heterogeneity/Reporting gaps/High–unclear bias/Unclear methods/Low quality/Small samples
[19]	Exacerbations and hospitalizations~(low certainty); QoL mostly sub-MID; SF-12 PCS ↑ (+9.2); 6MWT, anxiety, fatigue, depression, dyspnoea~; Adherence ✗; Physical activity ↑ (+9.5 min/day, 1 study); QoL meta-analysis: SMD −0.4 (ns); Certainty low–very low	I^2^ = 52–83%/↑ QoL heterogeneity (I^2^ = 83%)/No Q stats, subgroups, or publication bias tests	Heterogeneity/Reporting gaps/Unclear methods/Low quality/Small samples/Publication bias
[21]	Med adherence ↑ (13–49%) via self-report (MARS, Morisky), pill count, pharmacy, e-tracking; ~50% studies sig. (*p* < 0.04–0.001); strongest in COPD/asthma inhaler use; QoL inconsistent, no standard tools; disease mgmt indirect	No meta-analysis/Heterogeneity high due to study design, adherence definitions, reporting, delivery variation	Small samples/Heterogeneity/Low study quality
[35]	Physical activity ↑ (SMD 0.29; +971 steps/day)/QoL ↑ modestly (SMD 0.18), not sustained/Effects ↓ at follow-up/Adherence ✗/Self-care referenced, not quantified/Certainty low (bias, heterogeneity)	I^2^ = 54–84%/↑ in 6MWT, QoL, subjective PA/↓ effects w/age, BMI/Much unexplained	Heterogeneity/Reporting gaps/High–unclear bias/Low quality/Publication bias
[36]	Exacerbations, hospitalizations ↓ (mixed sig.)/QoL ↑ (CAT −1.29, *p* = 0.02)/SGRQ, EQ-5D~/Adherence underreported/↑ PA (~462 steps/day), inhaler use, self-care/Dyspnea~/Mixed but favorable overall	Low I^2^ across 6MWD, CAT, mMRC, SGRQ, hospitalizations/Funnel plot ✓/Subgroup by severity, intervention type	Small sample sizes/Some heterogeneity
[37]	6MWT ↑: Telemonitoring (+43.03 m), App (+25.74 m), VR (+18.43 m), Combined (+25.67 m); QoL ↑: App (SMD −0.47), Web (−1.49, SUCRA 94.3%), VR (−0.47); adherence ✗; eHealth ↑ self-management, engagement, ↓ readmissions	I^2^ = 12–97.8%/↑ in Web, Combined, Telemonitoring/No inconsistency (*p* > 0.65)/Sensitivity ✓	Heterogeneity/Reporting gaps/High–unclear bias/Low quality
[38]	Digital PR ↑ 6MWT, 12MWT, ESWT, steps/day (*p* < 0.001); QoL ↑ (SGRQ, CAT, CRQ, SF-36); exacerbations, ED visits, hospitalizations ↓; dyspnoea ↓ (mMRC); self-efficacy ↑ (PRAISE, GSES); anxiety/depression~(HADS); adherence ✗	Meta-analysis ✗/Clinical-methodological heterogeneity/Variability in design, tools, duration, comparators, participants	Heterogeneity/Small samples/High–unclear bias/Low study quality
[39]	mHealth → ↓ HbA1c (−0.30% to −1.95%), BP ↓ in CVD/T2DM, COPD ER visits ↓, readmissions ↓; QoL ↑ (SF-36, EQ-5D), mixed in COPD/T2DM; adherence ↑; benefits in self-monitoring, knowledge, engagement, satisfaction	Moderate heterogeneity in HbA1c/COPD variability/Influenced by design, duration, delivery, digital literacy/Sensitivity analyses ✓	Reporting issues/Heterogeneity
[40]	QoL ↑ (CAT −2.53; SGRQ ↑; EQ-5D ↑)/Self-efficacy ↑ (3–6 mo)/Dyspnea ↓ (mMRC −0.23)/6MWT~/Admissions~/Adherence not measured/Benefits: education, symptom monitoring, self-care ↑/No effect on acute outcomes or endurance	I^2^ = 0–94%/Low for CAT, mMRC, admissions/High for SGRQ, EQ-5D, QoL, 6MWT/Attributed to intervention, comparator, and design variation/No subgroup analyses	Reporting issues/Small samples/Heterogeneity/Low study quality
[41]	QoL ↑ in 9/10 studies (SGRQ, EQ-5D, CAT, CRQ, CCQ, 15D); 1 study~; self-management ↑ w/education/behavioral features; adherence not assessed but supported via reminders, education	I^2^ = 70%/χ^2^ = 30.26/Tau^2^ = 0.08/No subgroups/Variation in interventions, QoL tools, populations	Heterogeneity/Unclear methods/Low study quality
[42]	Adherence ↑ w/interactivity (e.g., reminders); QoL ↑ (CRQ +12.4, SGRQ ↓ decline); readmissions ↓ (13.7% vs. 29.1%); PA ↑ (ISWT +51 m); lung function~; adherence mostly inferred; education ↑ self-care	High clinical/methodological heterogeneity/Not quantified/Due to sample size, intervention types, outcome tools	Heterogeneity/Small samples/High–unclear bias
[43]	Cost-effectiveness ↑ (ICER €3.5–286 k/QALY); costs ↓ w/~QALYs; QoL (EQ-5D, CAT, SGRQ) mixed; Adherence ↑ w/reminders or monitoring; Disease mgmt ↑ (↓ admissions, ↑ self-care); PA and lung function~	High heterogeneity/No I^2^ or Q/Variation in DHI types, populations, methods, settings, evaluation models	Heterogeneity/Reporting gaps/High–unclear bias/Unclear methods/Low study quality
[44]	Telerehab ↑ short-term 6MWD (MD 7.52 m), ↓ dyspnea (mMRC), QoL ↑ (CAT, SGRQ short-term), HADS ↓ anxiety/depression short-term only; FEV1~; self-management ↑ (inferred); no direct behavior/adherence metrics	I^2^ = 0–56%/↓ in SGRQ, HADS/↑ in CAT/Subgroup: follow-up duration/Intervention, outcome, and population variability/Publication bias (6MWD, Egger’s *p* = 0.019) adjusted	Reporting issues/Heterogeneity
[45]	COPD outcomes ↑: ↓ exacerbations, hospital/ER visits; QoL ↑ (SGRQ, CAT, CRQ, EQ-5D); adherence ↑ via reminders, diaries, inhaler monitors; ↑ self-care, 6MWT, FEV_1_; mobile/web/tablet tools effective	Substantial heterogeneity in interventions, populations, outcomes, methods/Not quantified/Narrative synthesis used	Reporting issues/Heterogeneity/Low study quality
[46]	TR vs. no intervention: ↑ functional capacity (SMD 0.29), dyspnea (0.76), QoL (0.57); asynchronous TR ↑ vs. NI (g 0.39–0.82); smaller or~effects vs. center-based care; outcomes assessed via walk tests, CAT/SGRQ/CRQ, dyspnea scales, psychosocial tools; med adherence ✗	Substantial heterogeneity in interventions, populations, outcomes, methods/Not quantified/Narrative synthesis only	Reporting issues/Heterogeneity/Low study quality
[47]	Pulmonary function: FEV1 ↑ (ns), FEV1 L ↑ (ns), FEV1/FVC ↑ (ns), FVC ↑ (1 study), FVC%~; QoL, adherence ✗; interventions sometimes included self-monitoring, exercise, provider contact	I^2^ reported/Random-effects model used/Heterogeneity from platform, COPD severity, setting, duration, quality	Reporting issues/High–unclear bias/Unclear methods/Low study quality
[48]	PA: mixed effects (↑13% or ↓2.5%) via smartphone sensors; ISWT ↑ (21%, 18.3%) or ~; QoL~; No adherence data; Smartphone use ✓ (89–100%), some tech-related dropouts; PA correlated w/use (r = 0.62)	No I^2^/Q reported/Substantial clinical-methodological heterogeneity/No subgroup/sensitivity analyses	Reporting gaps/Small samples/High–unclear bias/Low study quality
[49]	HRQoL ↑ short-term (SGRQ, CCQ; SMD –0.22); PA ↑ (MD +864 steps); no 12-month effect; hospitalizations, AECOPD, smoking cessation~; med adherence ✗	I^2^ = 0%/*p* > 0.60/No subgroup analyses/↓ steps w/age (–33/yr, *p* = 0.03)/Random-effects	Small sample size/High–unclear risk of bias
[50]	Physical activity ↑ (MD 64.7 min/week); 6MWT~(MD −1.3 m); Dyspnea~(SMD 0.088); no sig. QoL or adherence data; assessed via accelerometers, self-reports, validated dyspnea tools; no publication bias	I^2^ = 0–85%/↓ after outlier removal/Mixed models used (I^2^ > 30%: random, ≤30%: fixed)	Heterogeneity/Reporting issues/Small samples/High–unclear risk of bias
[51]	Exacerbation ↓ (OR 0.20, 95% CI: 0.07–0.62)/I^2^ = 59%/QoL, adherence ✗/Daily symptom tracking ✓ (low certainty)/Usability, satisfaction inconsistent/No pooled data for admissions or ER visits	I^2^ = 59%/χ^2^ = 4.9 (*p* = 0.08)/Moderate heterogeneity not statistically significant	Heterogeneity/Unclear methods/Low quality/Publication bias/Small samples
[52]	Telemonitoring: Hosp. adm~(SMD –0.10); ER visits ↓ (SMD –0.14); QoL mixed (SGRQ ↑, EQ-5D/SF-36~); Time to adm~; Adherence ✗; Self-mgmt ↑ in some; Satisfaction ✓; Cost ↓ (∼50%); Mortality~; Mental health effects mixed	I^2^: hosp. adm = 24%, ER = 18%/Fixed-effects model/Clinical variation noted/No subgroup analyses	Reporting issues/Heterogeneity/Low study quality

Study—Reference to review article; O—Outcomes: Clinical outcomes (e.g., exacerbations, hospital admissions, ER visits), QoL measures (e.g., SGRQ, EQ-5D, SF-36), medication adherence, self-management, and patient satisfaction; includes direction (↑ positive effect, ↓ negative effect, ~no significant effect) and magnitude (effect size: OR, SMD) with instruments used; Heterogeneity—Statistical method for heterogeneity assessment (e.g., I^2^, Q), sources of variability, and subgroup analyses details; Limitations—Common limitations across studies. Symbols—↑: positive or beneficial effect, ↓: negative or adverse effect, ~: no significant effect, ✓: presence or confirmed result, ✗: absence or negative result. For extended, detailed data, see Supplementary Material Table S9.

## 4. Discussion

### 4.1. Limitations of This Umbrella Review

Several important limitations should be considered when interpreting the findings of this umbrella review. First, as assessed using the AMSTAR-2 tool (Table 4), the majority of systematic reviews (90%) were rated as “Low” or “Critically Low” in methodological quality. These limitations, such as a lack of protocol registration, incomplete search strategies, and insufficient bias assessment, introduce potential risks of bias and reduce the reliability of their synthesized conclusions. Second, due to heterogeneity across the included reviews, in terms of intervention types, outcome definitions, study populations, and comparator conditions, this umbrella review relied exclusively on narrative synthesis. While this provides valuable insights, it limits the ability to draw generalizable conclusions and quantify effect sizes. Third, the evidence base was heavily weighted toward studies conducted in high-income countries [45,48]. Few reviews addressed the feasibility of implementation in low-resource contexts, raising concerns about global applicability. Finally, outcome reporting was often inconsistent and lacked standardization, particularly for medication adherence and exacerbation frequency, making comparisons and synthesis difficult [21,42].

Together, these limitations highlight the need for caution when interpreting the results. While this umbrella review provides a high-level synthesis of current evidence on AI-based prediction and digital health interventions for COPD, robust and standardized research is needed to consolidate the evidence base. Given that most included reviews were rated as Low or Critically Low in methodological quality according to the AMSTAR-2 assessment, the findings of this umbrella review should be interpreted with caution and regarded as indicative trends rather than definitive conclusions.

### 4.2. Gaps and Limitations in Literature

A primary gap identified across the included reviews is the scarcity of external validation of AI predictive models. Although many models demonstrated strong internal predictive performance through methods such as k-fold cross-validation and bootstrapping [17,29,30], very few were rigorously tested on independent datasets or across diverse patient populations. This limitation raises concerns about overfitting and substantially restricts the generalizability and clinical reliability of these tools. Reviews repeatedly emphasized the need for robust external validation and calibration efforts in realistic clinical contexts [32] because no models were deemed clinically ready [31]. Another major limitation is the lack of standardization across datasets, modeling methods, and outcome definitions. Exacerbations were defined in different ways, ranging from symptom worsening to hospital admissions, while outcome reporting in digital health interventions relied on heterogeneous instruments for quality of life and poorly defined adherence measures [19,21,29,35,37,38]. Similarly, inconsistencies in biosignal collection protocols and clinical feature availability (e.g., FEV_1_, PaCO_2_) undermine reproducibility and complicate cross-study comparison. These issues underscore the urgent need for harmonized evaluation standards and the development of core outcome sets. Methodological weaknesses were also widespread. Across both AI and intervention studies, small sample sizes, short follow-up durations, and reliance on synthetic augmentation in some deep learning studies limited insights into long-term outcomes and real-world robustness [30,31,46,47]. Such weaknesses create uncertainty within the evidence base and highlight the importance of more rigorous review conduct. Limited interpretability hinders clinical adoption. While deep learning models often achieve higher predictive accuracy, they are frequently deployed as black boxes. The lack of transparent, clinician-friendly explanations undermines trust and usability. Only a few studies reported in [29,32] used inherently interpretable models like logistic regression and clinical scores, though none explicitly reported integrating interpretable frameworks or explainable AI techniques. Equity considerations were another consistent gap. Most studies were conducted in high-income countries with older, digitally literate populations, while research in low- and middle-income countries was virtually absent [17,42,43]. Socioeconomic and demographic factors, including digital access and literacy, were seldom examined [39], limiting the understanding of how interventions perform in diverse populations and raising questions about global applicability. Finally, integration into clinical workflows remains underdeveloped. AI prediction models and digital interventions were largely designed in isolation, without interoperability with electronic health records, care pathways, or clinician routines [31,44,45]. This lack of integration poses a significant barrier to sustainable implementation. Even high-performing tools risk limited real-world impact unless they are embedded within healthcare infrastructures in ways that support clinician trust, usability, and scalability. In summary, while AI and digital health interventions hold considerable promise for COPD care, their current contribution is undermined by methodological flaws, fragmented evaluation, and poor clinical integration. Without major improvements, these tools will remain prototypes rather than viable healthcare solutions.

### 4.3. Future Implications

The emerging predictive strength of AI-driven models using wearable biosignals highlights significant opportunities for more proactive and personalized COPD care. By continuously monitoring physiological markers such as oxygen saturation (SpO_2_), respiratory rate, heart rate, and physical activity, clinicians could anticipate exacerbations and initiate timely interventions, potentially reducing hospitalizations and improving clinical outcomes. To achieve this, integration into existing care workflows is critical. Many of the reviewed AI and digital tools lacked interoperability with electronic health records, significantly limiting their integration into routine clinical workflows [30,44,45]. Without interoperability across platforms, the clinical adoption of these technologies remains restricted. Investments in robust IT infrastructures, clinician involvement in system design, and attention to workflow integration will be essential to promote usability, reduce disruption, and encourage sustained adoption. Clinician trust also represents a key factor for implementation. Many AI models, particularly those based on deep learning, function as “black boxes” with limited interpretability. Enhancing transparency through explainable AI techniques, interpretable model architectures, and clear, actionable outputs could foster confidence and encourage wider usage [29,32]. Rigorous real-world validation studies are likewise necessary to confirm reliability and effectiveness across diverse clinical contexts. For patients, digital interventions have been associated with improvements in quality of life, self-management behaviors, and, in some cases, medication adherence [19,21,35,36,38,40]. These benefits appear most evident when interventions are personalized through adjustable educational content, individualized feedback, and clinician interaction [37,46]. However, disparities in digital literacy and access remain significant barriers, particularly among older adults and in low-resource environments [39]. Addressing equity will be essential to ensure all patient groups benefit from these innovations. Healthcare systems may also stand to gain from the scalability and cost-effectiveness of AI-driven tools and digital interventions, particularly in reducing healthcare utilization by preventing exacerbations and hospitalizations [43,51]. Yet, this potential will only be realized with targeted investments in technology, training, and infrastructure, alongside clear policies addressing data privacy, security, and ethical use of patient information. From a research perspective, several priorities emerge. Future studies must prioritize rigorous external validation of AI-based predictive models using independent datasets and diverse patient populations to ensure generalizability and reliability. Moving this field forward will require a decisive shift from proof-of-concept studies to large, externally validated trials that test predictive models in patient populations reflecting the diversity of COPD care across different ages, disease severities, and healthcare settings.

Standardization of outcome measures is equally important. Current variability in definitions of exacerbations, outcomes, and data collection methods complicates comparison and synthesis across studies. The field suffers from a lack of shared definitions and outcome measures, particularly in how exacerbations, quality of life, adherence, and healthcare utilization are measured and reported. Developing core outcome sets and harmonized evaluation frameworks would not only improve comparability but also strengthen the overall evidence base [35,37,38]. Moreover, long-term trials are needed to assess the sustainability and effectiveness of both predictive models and digital interventions. The predominance of short follow-up studies (e.g., [31,38,40,47]) limits insight into adherence, durability of benefits, and real-world impact. Trials extending 12 months or more would better capture long-term outcomes. Addressing equity in access must also be central to future work, with deliberate inclusion of underrepresented groups and studies conducted in low- and middle-income countries [39,43]. Equity should not be treated as an afterthought; most interventions have been developed and tested in high-income countries with digitally literate participants, leaving major gaps in understanding their effectiveness for older adults, people with limited technology access, or those in resource-constrained settings. Overcoming barriers related to affordability, connectivity, and digital literacy will be critical to designing globally relevant interventions. Finally, both AI and digital health research should integrate interpretability into model and system design. Transparent predictions and recommendations are essential not only for building clinician and patient trust, but also for complying with regulatory standards and supporting ethical implementation [29,32]. At the same time, integrating AI-based prediction and digital interventions into day-to-day COPD care will require interoperable IT systems and clear data governance standards. Without this foundation, even well-validated technologies are unlikely to progress beyond pilot projects.

In addition to the studies formally included in this umbrella review, recent publications have further examined complementary aspects of digital health and artificial intelligence in COPD care. For instance, Koh et al., 2023 [57] provided an umbrella review of telemedical interventions for COPD management, reinforcing the safety and potential of telemonitoring and remote rehabilitation as adjuncts to conventional care. Robertson et al., 2024 [58] discussed the integration of AI into COPD diagnosis and phenotyping, particularly the use of multimodal data and clinician–AI collaboration to enhance early detection. Long et al., 2023 [59] and O’Connor et al., 2025 [60] similarly highlighted the growing use of digital and mobile health tools to support self-management, rehabilitation, and monitoring in patients with moderate-to-severe COPD. Although these works fall outside the temporal and methodological scope of the registered protocol, they complement the present synthesis by illustrating the continued evolution of AI-driven and digitally enabled approaches to COPD prevention, diagnosis, and long-term management. Looking ahead, the integration of AI-driven prediction tools and digital interventions into COPD management will depend on overcoming barriers of interoperability, interpretability, and validation, as well as ensuring equitable access. Long-term, standardized, and inclusive evaluations will be critical to move from proof-of-concept to sustainable clinical adoption. Beyond these research priorities, successful clinical translation will require embedding AI-based predictive models and digital interventions within existing COPD care pathways that connect patients, clinicians, and healthcare systems. This process will rely on seamless integration with electronic health records, user-friendly interfaces, and the active involvement of clinicians in system design and validation. Data privacy, explainability, and regulatory compliance should also be prioritized to foster trust and encourage implementation. Integrating these technologies as decision-support tools, rather than replacements for clinical judgment, may provide the most practical route toward routine use in COPD management. Building on these considerations, several practical recommendations emerge for key stakeholders. For clinicians, emphasis should be placed on adopting validated and interpretable digital tools that fit smoothly within existing workflows. Developers should prioritize transparency, interoperability, and usability to ensure that AI-based systems meet both clinical and patient needs. Policymakers should support digital-health infrastructure, training, and equity initiatives to facilitate safe and inclusive implementation. Coordinated collaboration among these groups will be essential to translate technological progress into tangible benefits for COPD management.

## 5. Conclusions

This umbrella review synthesized evidence from 27 systematic reviews and meta-analyses to evaluate two interrelated domains in COPD care: (1) AI-based prediction of acute exacerbations using wearable-derived biosignals, and (2) the effectiveness of digital health interventions in improving disease management, quality of life, and medication adherence. Overall, AI-driven predictive models showed promising internal performance, particularly when leveraging wearable signals such as oxygen saturation, respiratory rate, and activity levels. These biosensor-based approaches appear to offer earlier and more dynamic detection of exacerbation risk compared to traditional methods that rely primarily on static clinical indicators, spirometry, or questionnaires. However, their clinical readiness remains limited by small datasets, inconsistent outcome definitions, limited interpretability, and the near-absence of external validation. Without robust generalizability testing, integration into clinical workflows is premature. Digital health interventions, including mobile applications, telemonitoring, and telerehabilitation, demonstrated small to moderate improvements in quality of life and functional capacity, with mixed effects on adherence and exacerbation rates. Evidence suggests that multi-component, interactive interventions yield the most consistent benefits, particularly when they complement rather than replace standard care. Yet methodological weaknesses, heterogeneous comparators, and equity gaps limit the strength and generalizability of these findings. Taken together, the reviewed evidence indicates that wearable-based predictive models and digital interventions hold significant promise for enabling proactive, personalized, and scalable COPD management. Their added value lies in offering continuous monitoring, timely risk stratification, and enhanced patient engagement, features that extend beyond the capabilities of conventional care models. Nonetheless, to translate these advances into clinical and public health impact, future research must prioritize rigorous external validation, standardization of outcome measures, attention to digital equity, and seamless integration into existing healthcare systems. Only through these steps can AI-enabled prediction and digital health interventions evolve from promising prototypes to effective, equitable, and sustainable tools for real-world COPD care.

## Figures and Tables

**Table 1:** PICO model for the research questions.

RQ	P—Patient	I—Intervention	C—Comparator	O—Outcome
RQ1	Patients with Chronic Obstructive Pulmonary Disease	Use of wearable biosignals and AI for prediction of exacerbations	Not applicable	Prediction of AECOPD, along with challenges and barriers in implementation or application
RQ2	Patients with Chronic Obstructive Pulmonary Disease	Digital health interventions	Standard care	Disease management effectiveness, quality of life and medication adherence

**Table 2:** Summary of search queries.

Code	Focus Area	Research Question	Search Query
RQ1	Modeling and Prediction	What is known about the use of wearable biosignals and artificial intelligence for predicting COPD exacerbations, and what challenges have been reported in their application?	(COPD OR chronic obstructive pulmonary disease) AND (exacerbation OR acute episode) AND (prediction OR forecasting OR early detection) AND (wearable OR wearable sensor OR wearable device) AND (biosignal OR physiological signal OR vital sign) AND (machine learning OR artificial intelligence OR deep learning) AND (systematic review OR meta-analysis OR review)
RQ2	Intervention	How do digital health interventions affect disease management, quality of life, and medication adherence in COPD patients compared to standard care?	(digital health OR mHealth OR eHealth OR telehealth OR wearable devices OR remote monitoring OR dtx OR digital intervention OR digital therapeutics) AND (chronic obstructive pulmonary disease OR COPD) AND (disease management OR self-management OR quality of life OR medication adherence) AND (effectiveness OR impact OR outcomes) AND (standard care OR usual care OR conventional care) AND (systematic review OR meta-analysis OR review)

**Table 3:** Summary of inclusion and exclusion criteria, including descriptions and unique identifiers used in the screening process.

Inclusion Criteria		
Criteria	Description	Code
Scope of Research	Review must focus on COPD exacerbations and address either AI/wearable biosignals for prediction or digital health tools for disease management.	INC_Scope
Type of Publication	Only peer-reviewed systematic reviews, meta-analyses, or umbrella reviews are included.	INC_ReviewType
Relevance of Technologies	Must assess AI/wearables for COPD prediction or digital interventions for disease management.	INC_TechRelevance
Human-Centric Base	Must be based on human studies.	INC_Humanc
Scalability	Must address scalable technologies suitable for real-world or clinical use.	INC_Scalability
Outcomes Reported	Must report outcomes like prediction accuracy or impacts on management, adherence, or quality of life.	INC_Outcomes
**Exclusion Criteria**		
**Criteria**	**Description**	**Code**
Non-relevant Topic	Excluded if not focused on COPD or if lacking AI, wearables, or digital health relevance.	EXC_Scope
Non-human Base	Reviews focusing solely on animal or in vitro studies will be excluded.	EXC_Humans
Invasive or Non-scalable	Excluded if focused only on invasive or hospital-based tools without scalable alternatives.	EXC_Scalability
Publication Type	Excluded if not a systematic review or meta-analysis.	EXC_PubType
Publication Period	Excluded if published outside the 2015–2025 range.	EXC_Year
Publication Language	Excluded if not published in English.	EXC_Language
Peer Review Status	Excluded if not published in a peer-reviewed journal	EXC_PeerReview

**Table 4:** AMSTAR2—Methodological quality of eligible studies.

#	Article	Q1	Q2	Q3	Q4	Q5	Q6	Q7	Q8	Q9	Q10	Q11	Q12	Q13	Q14	Q15	Q16	Score
Assessment of RQ1 eligible studies																		
1	[17]	Y	P	Y	P	Y	Y	N	P	N	N	X	X	N	Y	X	Y	Critically Low
2	[29]	Y	N	Y	Y	Y	Y	N	P	P	N	Y	Y	N	Y	N	Y	Low
3	[30]	N	P	Y	P	Y	N	N	P	N	N	X	X	N	N	X	Y	Critically Low
4	[31]	N	N	Y	P	Y	Y	N	Y	N	N	X	X	N	N	X	Y	Critically Low
5	[32]	Y	P	Y	P	Y	Y	N	P	Y	N	X	X	Y	Y	X	Y	Moderate
Assessment of RQ2 eligible studies																		
6	[33]	Y	N	Y	P	N	Y	P	P	Y	N	Y	N	Y	Y	Y	Y	Low
7	[34]	Y	Y	Y	Y	Y	N	Y	Y	Y	Y	Y	Y	Y	Y	N	Y	Low
8	[19]	Y	N	Y	P	Y	Y	N	Y	Y	N	Y	N	Y	Y	N	Y	Critically Low
9	[21]	Y	N	Y	P	Y	Y	N	P	Y	N	X	X	Y	Y	X	Y	Critically Low
10	[35]	Y	Y	Y	Y	Y	Y	Y	P	Y	N	Y	Y	Y	Y	Y	Y	High
11	[36]	Y	P	Y	P	Y	Y	N	P	Y	N	Y	N	Y	Y	Y	Y	Low
12	[37]	Y	P	Y	P	Y	Y	N	P	Y	N	Y	Y	Y	Y	Y	Y	Low
13	[38]	Y	P	Y	P	Y	Y	N	Y	Y	N	X	X	Y	Y	X	Y	Low
14	[39]	Y	N	Y	N	Y	Y	N	P	N	N	X	X	N	N	X	Y	Critically Low
15	[40]	Y	P	Y	P	Y	Y	N	P	Y	N	Y	Y	Y	Y	N	Y	Critically Low
16	[41]	Y	P	Y	P	Y	Y	N	Y	P	N	Y	N	Y	Y	Y	Y	Low
17	[42]	Y	P	Y	P	Y	Y	N	P	P	N	X	X	Y	N	X	Y	Low
18	[43]	Y	P	Y	P	Y	Y	N	Y	Y	N	X	X	N	Y	X	Y	Critically Low
19	[44]	Y	P	Y	P	Y	Y	N	Y	Y	N	Y	Y	N	Y	Y	Y	Critically Low
20	[45]	Y	N	N	P	Y	Y	N	P	P	N	X	X	Y	N	X	Y	Critically Low
21	[46]	Y	P	Y	P	Y	N	N	P	Y	N	Y	N	Y	Y	Y	Y	Low
22	[47]	Y	Y	Y	Y	Y	Y	N	Y	Y	N	Y	Y	Y	Y	Y	Y	Low
23	[48]	Y	Y	Y	Y	N	N	N	P	Y	N	X	X	Y	N	X	Y	Low
24	[49]	Y	Y	N	Y	Y	Y	Y	Y	Y	N	Y	Y	Y	Y	Y	Y	Moderate
25	[50]	Y	Y	N	Y	Y	Y	N	Y	Y	Y	Y	Y	Y	Y	Y	Y	Low
26	[51]	N	P	Y	Y	Y	Y	N	Y	Y	N	Y	Y	Y	Y	Y	Y	Low
27	[52]	Y	Y	Y	Y	Y	Y	N	Y	Y	N	Y	Y	Y	Y	Y	Y	Low

AMSTAR-2 items: Q1. Did the research questions and inclusion criteria for the review include the components of PICO? Q2. Did the report of the review contain an explicit statement that the review methods were established prior to the conduct of the review and did the report justify any significant deviations from the protocol? Q3. Did the review authors explain their selection of the study designs for inclusion in the review? Q4. Did the review authors use a comprehensive literature search strategy? Q5. Did the review authors perform study selection in duplicate? Q6. Did the review authors perform data extraction in duplicate? Q7. Did the review authors provide a list of excluded studies and justify the exclusions? Q8. Did the review authors describe the included studies in adequate detail? Q9. Did the review authors use a satisfactory technique for assessing the risk of bias (RoB) in individual studies that were included in the review? Q10. Did the review authors report on the sources of funding for the studies included in the review? Q11. If meta-analysis was performed, did the review authors use appropriate methods for statistical combination of results? Q12. If meta-analysis was performed, did the review authors assess the potential impact of RoB in individual studies on the results of the meta-analysis or other evidence synthesis? Q13. Did the review authors account for RoB in primary studies when interpreting/discussing the results of the review? Q14. Did the review authors provide a satisfactory explanation for, and discussion of, any heterogeneity observed in the results of the review? Q15. If they performed quantitative synthesis, did the review authors carry out an adequate investigation of publication bias (small study bias) and discuss its likely impact on the results of the review? Q16. Did the review authors report any potential sources of conflict of interest, including any funding they received for conducting the review? Note: Y (Green) = Yes; N (Red) = No; P (Yellow) = Partial; X (Grey) = Not applicable. AMSTAR Score: Blue = High; Yellow = Moderate; Pink = Low; Red = Critically Low.

## Data Availability

No new data were created or analyzed in this study. The data supporting this work are derived from previously published systematic and scoping reviews, which are cited in the manuscript.

## Supplementary Materials

The following supporting information can be downloaded at: https://www.mdpi.com/article/10.3390/healthcare13233037/s1. Table S1: Inclusion criteria, including descriptions and unique identifiers used in the screening process; Table S2: Exclusion criteria, including descriptions and unique identifiers used in the screening process; Table S3: Data extracted for RQ1 with column descriptions; Table S4: Data extracted for RQ2 with column descriptions; Table S5: CCA values for RQ1, reflecting the aggregate degree of primary study overlap across included systematic reviews; Table S6: CCA values for RQ2, reflecting the aggregate degree of primary study overlap across included systematic reviews; Table S7: List of excluded articles with bibliographic details, source database, research question code, and exclusion motivation; Table S8: Data extraction table for RQ1 studies with key details on datasets, AI/ML methods, validation, outcomes, and challenges. Study Year Review Type Number of Included Studies [17,29,31,32,33]; Table S9: Data extraction table for RQ2 studies with study design, PICOs, statistical findings, and main conclusions [36,37,38,39,40,41,42,43,44,45,46,47,48,49,50,51,52]; Table S10: Complete database search strings used in the literature search for PubMed, Scopus, and Web of Science.

## Abbreviations

The following abbreviations are used in this manuscript:

Adm	Hospital Admission
AE	Acute Exacerbation
AI	Artificial Intelligence
AECOPD	Acute Exacerbation COPD
API	Application Programming Interface
AUROC	Area Under the Receiver Operating Characteristic Curve
BMI	Body Mass Index
CAT	COPD Assessment Test
CCQ	Clinical COPD Questionnaire
CCA	Corrected Covered Area
CI	Confidence Interval
CKD	Chronic Kidney Disease
CNN	Convolutional Neural Network
COPD	Chronic Obstructive Pulmonary Disease
CRQ	Chronic Respiratory Questionnaire
CVD	Cardiovascular Disease
DL	Deep Learning
DNN	Deep Neural Network
DT	Decision Tree
EHR	Electronic Health Record
EQ-5D	EuroQol 5-Dimension
ER	Emergency Room
FEV_1_	Forced Expiratory Volume in 1 Second
FVC	Forced Vital Capacity
GOLD	Global Initiative for COPD
HCP	Healthcare Professional
HR	Heart Rate
HRQoL	Heart Rate Quality of Life
ICU	Intensive Care Unit
ISWT	Incremental Shuttle Walk Test
IVR	Interactive Voice Response
LASSO	Least Absolute Shrinkage and Selection Operator
LR	Logistic Regression
LSTM	Long Short-Term Memory
MA	Meta-Analysis
MAQ	Medication Adherence Questionnaire
MG Test	Medication-taking Graph Test
mHealth	Mobile Health
MID	Minimal Important Difference
ML	Machine Learning
MMAS-8	Morisky Medication Adherence Scale-8
mMRC	Modified Medical Research Council (Dyspnea Scale)
NCSI	Nijmegen Clinical Screening Instrument
OR	Odds Ratio
PDA	Personal Digital Assistant
PCS	Physical Component Summary (from SF-12/SF-36)
PEARL	Dyspnea, Eosinopenia, Consolidation, Acidemia, and atriaL fibrillation Score
PR	Pulmonary Rehabilitation
PROs	Patient Reported Outcomes
Pts	Patients
QoL	Quality of Life
RF	Random Forest
RNN	Recurrent Neural Network
RPM	Remote Patient Monitoring
RR	Respiratory Rate
Self-mgmt	Self-management interventions
SF-36	Short Form-36 Health Survey
SGRQ	St. George’s Respiratory Questionnaire
SMD	Standardized Mean Difference
SpO_2_	Peripheral Capillary Oxygen Saturation
SR	Systematic Review
STAIN	Spatio-Temporal Artificial Intelligence Network
SVM	Support Vector Machine
TUG	Timed Up and Go test
VR	Virtual Reality
WAQI	World Air Quality Index
6MWD	6-Minute Walk Distance
6MWT	6-Minute Walk Test
