# Supplementary material for: Artificial Intelligence Methods and Digital Intervention Strategies for Predicting and Managing Chronic Obstructive Pulmonary Disease Exacerbations: An Umbrella Review
Source: Healthcare (Basel). 2025 Nov 24;13(23):3037. doi: 10.3390/healthcare13233037 (PMC12691994; doi:10.3390/healthcare13233037)
Supplement: Supplementary file 1 [file healthcare-13-03037-s001.zip › s2_list_of_papers_excluded.pdf]

Table S7. List of excluded articles with bibliographic details, source database, research question code, and exclusion motivation.

Article	Year	Abstract	Link	RQ Code	Source (DB)	Exclusion Motivation
Machine learning and deep learning predictive models for long-te	2023	Background: Machine learning and deep learning models have been increasingly used to predict long-term disease progression in patients with chronic obstructive pulmonary disease (COPD). We aimed to summarise the performance of such prognostic models for COPD, compare their relative performances, and identify key research gaps. Methods: We conducted a systematic review and meta-analysis to compare the performance of machine learning and deep learning prognostic models and identify pathways for future research. We searched PubMed, Embase, the Cochrane Library, ProQuest, Scopus, and Web of Science from database inception to April 6, 2023, for studies in English using machine learning or deep learning to predict patient outcomes at least 6 months after initial clinical presentation in those with COPD. We included studies comprising human adults aged 18-90 years and allowed for any input modalities. We reported area under the receiver operator characteristic curve (AUC) with 95% CI for predictions of mortality, exacerbation, and decline in forced expiratory volume in 1 s (FEV1). We reported the degree of interstudy heterogeneity using Cochran's Q test (significant heterogeneity was defined as p≤0·10 or I2>50%). Reporting quality was assessed using the TRIPOD checklist and a risk-of-bias assessment was done using the PROBAST checklist. This study was registered with PROSPERO (CRD42022323052). Findings: We identified 3620 studies in the initial search. 18 studies were eligible, and, of these, 12 used conventional machine learning and six used deep learning models. Seven models analysed exacerbation risk, with only six reporting AUC and 95% CI on internal validation datasets (pooled AUC 0·77 [95% CI 0·69-0·85]) and there was significant heterogeneity (I2 97%, p<0·0001). 11 models analysed mortality risk, with only six reporting AUC and 95% CI on internal validation datasets (pooled AUC 0·77 [95% CI 0·74-0·80]) with significant degrees of heterogeneity (I2 60%, p=0·027). Two studies assessed decline in lung function and were unable to be pooled. Machine learning and deep learning models did not show significant improvement over pre-existing disease severity scores in predicting exacerbations (p=0·24). Three studies directly compared machine learning models against pre-existing severity scores for predicting mortality and pooled performance did not differ (p=0·57). Of the five studies that performed external validation, performance was worse than or equal to regression models. Incorrect handling of missing data, not reporting model uncertainty, and use of datasets that were too small relative to the number of predictive features included provided the largest risks of bias. Interpretation: There is limited evidence that conventional machine learning and deep learning prognostic models demonstrate superior performance to pre-existing disease severity scores. More rigorous adherence to reporting guidelines would reduce the risk of bias in future studies and aid study reproducibility.	https://pubmed.ncbi.nlm.nih.gov/38000872/	Q1	PubMed	EXC_Scalability
Lack of Evidence Regarding Markers Identifying Acute Heart Fail	2024	Background: Due to shared symptoms, acute heart failure (AHF) is difficult to differentiate from an acute exacerbation of COPD (AECOPD). This systematic review aimed to identify markers that can diagnose AHF underlying acute dyspnea in patients with COPD presenting at the hospital. Methods: All types of observational studies and clinical trials that investigated any marker’s ability to diagnose AHF in acutely dyspneic COPD patients were considered eligible for inclusion. An AI tool (ASReview) supported the title and abstract screening of the articles obtained from PubMed, Scopus, Web of Science, the Cochrane Library, Embase, and CINAHL until April 2023. Full text screening was independently performed by two reviewers. Twenty percent of the data extraction was checked by a second reviewer and the risk of bias was assessed in duplicate using the QUADAS-2 tool. Markers’ discriminative abilities were evaluated in terms of sensitivity, specificity, positive and negative predictive values, and the area under the curve when available. Results: The search identified 10,366 articles. After deduplication, title and abstract screening was performed on 5,386 articles, leaving 153 relevant, of which 82 could be screened full text. Ten distinct studies (reported in 16 articles) were included, of which 9 had a high risk of bias. Overall, these studies evaluated 12 distinct laboratory and 7 non-laboratory markers. BNP, NT-proBNP, MR-proANP, and inspiratory inferior vena cava diameter showed the highest diagnostic discrimination. Conclusion: There is not much evidence for the use of markers to diagnose AHF in acutely dyspneic COPD patients in the hospital setting. BNPs seem most promising, but should be interpreted alongside imaging and clinical signs, as this may lead to improved diagnostic accuracy. Future validation studies are urgently needed before any AHF marker can be incorporated into treatment decision-making algorithms for patients with COPD. Protocol Registration: CRD42022283952. © 2024 van Dijk et al.	https://www.scopus.com/inward/record.uri?eid=2-s	Q1	Scopus	EXC_Scope
Demystification of artificial intelligence for respiratory clinicians m	2023	Introduction: Asthma and chronic obstructive pulmonary disease (COPD) are leading causes of morbidity and mortality worldwide. Despite all available diagnostics and treatments, these conditions pose a significant individual, economic and social burden. Artificial intelligence (AI) promises to support clinical decision-making processes by optimizing diagnosis and treatment strategies of these heterogeneous and complex chronic respiratory diseases. Its capabilities extend to predicting exacerbation risk, disease progression and mortality, providing healthcare professionals with valuable insights for more effective care. Nevertheless, the knowledge gap between respiratory clinicians and data scientists remains a major constraint for wide application of AI and may hinder future progress. This narrative review aims to bridge this gap and encourage AI deployment by explaining its methodology and added value in asthma and COPD diagnosis and treatment. Areas covered: This review offers an overview of the fundamental concepts of AI and machine learning, outlines the key steps in building a model, provides examples of their applicability in asthma and COPD care, and discusses barriers to their implementation. Expert opinion: Machine learning can advance our understanding of asthma and COPD, enabling personalized therapy and better outcomes. Further research and validation are needed to ensure the development of clinically meaningful and generalizable models. © 2024 The Author(s). Published by Informa UK Limited, trading as Taylor & Francis Group.	https://www.scopus.com/inward/record.uri?eid=2-s	Q1	Scopus	EXC_PubType
Advancing Digital Solutions to Overcome Longstanding Barriers	2023	Maintenance therapy delivered via inhaler is central to asthma and chronic obstructive pulmonary disease (COPD) management. Poor adherence to inhaled medication and errors in inhalation technique have long represented major barriers to the optimal management of these chronic conditions. Technological innovations may provide a means of overcoming these barriers. This narrative review examines ongoing advances in digital technologies relevant to asthma and COPD with the potential to inform clinical decision-making and improve patient care. Digital inhaler devices linked to mobile apps can help bring about changes in patients’ behaviors and attitudes towards disease management, particularly when they build in elements of interactivity and gamification. They can also support ongoing technique education, empowering patients and helping providers maximize the value of consultations and develop effective action plans informed by insights into the patient’s inhaler use patterns and their respiratory health. When combined with innovative techniques such as machine learning, digital devices have the potential to predict exacerbations and prompt pre- emptive intervention. Finally, digital devices may support an advanced precision medicine approach to respiratory disease management and help support shared decision-making. Further work is needed to increase uptake of digital devices and integrate their use into care pathways before their full potential in personalized asthma and COPD management can be realized. © 2023 Bosnic-Anticevich et al.	https://www.scopus.com/inward/record.uri?eid=2-s	Q1	Scopus	EXC_PubType
Personalized medicine for patients with COPD: Where are we?	2019	Chronic airflow limitation is the common denominator of patients with chronic obstructive pulmonary disease (COPD). However, it is not possible to predict morbidity and mortality of individual patients based on the degree of lung function impairment, nor does the degree of airflow limitation allow guidance regarding therapies. Over the last decades, understanding of the factors contributing to the heterogeneity of disease trajectories, clinical presentation, and response to existing therapies has greatly advanced. Indeed, diagnostic assessment and treatment algorithms for COPD have become more personalized. In addition to the pulmonary abnormalities and inhaler therapies, extra-pulmonary features and comorbidities have been studied and are considered essential components of comprehensive disease management, including lifestyle interventions. Despite these advances, predicting and/or modifying the course of the disease remains currently impossible, and selection of patients with a beneficial response to specific interventions is unsatisfactory. Consequently, non-response to pharmacologic and non-pharmacologic treatments is common, and many patients have refractory symptoms. Thus, there is an ongoing urgency for a more targeted and holistic management of the disease, incorporating the basic principles of P4 medicine (predictive, preventive, personalized, and participatory). This review describes the current status and unmet needs regarding personalized medicine for patients with COPD. Also, it proposes a systems medicine approach, integrating genetic, environmental, (micro)biological, and clinical factors in experimental and computational models in order to decipher the multilevel complexity of COPD. Ultimately, the acquired insights will enable the development of clinical decision support systems and advance personalized medicine for patients with COPD. © 2019 Franssen et al.	https://www.scopus.com/inward/record.uri?eid=2-s	Q1	Scopus	EXC_Scope

Machine Learning Characterization of COPD Subtypes: Insights	2020	COPD is a heterogeneous syndrome. Many COPD subtypes have been proposed, but there is not yet consensus on how many COPD subtypes there are and how they should be defined. The COPD Genetic Epidemiology Study (COPDGene), which has generated 10-year longitudinal chest imaging, spirometry, and molecular data, is a rich resource for relating COPD phenotypes to underlying genetic and molecular mechanisms. In this article, we place COPDGene clustering studies in context with other highly cited COPD clustering studies, and summarize the main COPD subtype findings from COPDGene. First, most manifestations of COPD occur along a continuum, which explains why continuous aspects of COPD or disease axes may be more accurate and reproducible than subtypes identified through clustering methods. Second, continuous COPD-related measures can be used to create subgroups through the use of predictive models to define cut-points, and we review COPDGene research on blood eosinophil count thresholds as a specific example. Third, COPD phenotypes identified or prioritized through machine learning methods have led to novel biological discoveries, including novel emphysema genetic risk variants and systemic inflammatory subtypes of COPD. Fourth, trajectory-based COPD subtyping captures differences in the longitudinal evolution of COPD, addressing a major limitation of clustering analyses that are confounded by disease severity. Ongoing longitudinal characterization of subjects in COPDGene will provide useful insights about the relationship between lung imaging parameters, molecular markers, and COPD progression that will enable the identification of subtypes based on underlying disease processes and distinct patterns of disease progression, with the potential to improve the clinical relevance and reproducibility of COPD subtypes. © 2020 American College of Chest Physicians	https://www.scopus.com/inward/record.uri?eid=2-s	Q1	Scopus	EXC_Scope
CT-Based Commercial Software Applications: Improving Patient	2022	Chronic obstructive pulmonary disease (COPD) is heterogenous in its clinical manifestations and disease progression. Patients often have disease courses that are difficult to predict with readily available data, such as lung function testing. The ability to better classify COPD into well-defined groups will allow researchers and clinicians to tailor novel therapies, monitor their effects, and improve patient-centered outcomes. Different modalities of assessing these COPD phenotypes are actively being studied, and an area of great promise includes the use of quantitative computed tomography (QCT) techniques focused on key features such as airway anatomy, lung density, and vascular morphology. Over the last few decades, companies around the world have commercialized automated CT software packages that have proven immensely useful in these endeavors. This article reviews the key features of several commercial platforms, including the technologies they are based on, the metrics they can generate, and their clinical correlations and applications. While such tools are increasingly being used in research and clinical settings, they have yet to be consistently adopted for diagnostic work-up and treatment planning, and their full potential remains to be explored. © 2022 Wang et al.	https://www.scopus.com/inward/record.uri?eid=2-s	Q1	Scopus	EXC_Scalability
Developments in respiratory self-management interventions over	2023	This paper describes developments in the fields of asthma and COPD self-management interventions (SMIs) over the last two decades and discusses future directions. Evidence around SMIs has exponentially grown. Efficacy on group level is convincing and both asthma and COPD SMIs are currently recommended by respiratory guidelines. Core components of asthma SMIs are defined as education, action plans, and regular review, with some discussion about self-monitoring. Exacerbation action plans are defined as an integral part of COPD management. Patient's adherence to SMI's is however inadequate and significantly reducing the intervention's impact. Adherence could be improved by tailoring of SMIs to patients' needs, health beliefs, and capabilities; the use of shared decision making; and optimising the communication between patients and health care providers. Due to the COVID-19 pandemic, digital health innovations have rapidly been introduced and expanded. Digital technology use may increase efficiency, flexibility, and efficacy of SMIs. Furthermore, artificial intelligence can be used to e.g., predict exacerbations in action plans. Research around digital health innovations to ensure evidence-based practice is of utmost importance. Current implementation of respiratory SMIs is not satisfactory. Implementation research should be used to generate further insights, with cost-effectiveness, policy (makers), and funding being significant determinants. © The Author(s) 2023.	https://www.scopus.com/inward/record.uri?eid=2-s	Q1	Scopus	EXC_PubType
Chronic obstructive pulmonary disease risk assessment tools: is	2022	Purpose of reviewRisk assessment tools are essential in COPD care to help clinicians identify patients at higher risk of accelerated lung function decline, respiratory exacerbations, hospitalizations, and death.Recent findingsConventional methods of assessing risk have focused on spirometry, patient-reported symptoms, functional status, and a combination of these tools in composite indices. More recently, qualitatively and quantitatively assessed chest imaging findings, such as emphysema, large and small airways disease, and pulmonary vascular abnormalities have been associated with poor long-term outcomes in COPD patients. Although several blood and sputum biomarkers have been investigated for risk assessment in COPD, most still warrant further validation. Finally, novel remote digital monitoring technologies may be valuable to predict exacerbations but their large-scale performance, ease of implementation, and cost effectiveness remain to be determined.SummaryGiven the complex heterogeneity of COPD, any single metric is unlikely to fully capture the risk of poor long-term outcomes. Therefore, clinicians should review all available clinical data, including spirometry, symptom severity, functional status, chest imaging, and bloodwork, to guide personalized preventive care of COPD patients. The potential of machine learning tools and remote monitoring technologies to refine COPD risk assessment is promising but remains largely untapped pending further investigation. Copyright © 2021 Wolters Kluwer Health, Inc. All rights reserved.	https://www.scopus.com/inward/record.uri?eid=2-s	Q1	Scopus	EXC_PubType
The role of digital health in respiratory diseases management: a	2025	This review provides a detailed overview of how digital health can be utilized in the management of Interstitial Lung Disease (ILD), and Chronic Obstructive Pulmonary Disease (COPD). ILD encompasses a diverse range of lung disorders characterized by inflammation and scarring of lung tissue, leading to restrictive lung physiology and impaired gas exchange, with symptoms including progressive dyspnoea, cough, and hypoxia. COPD which ranks as the third leading cause of death globally, is characterized by chronic lung inflammation causing irreversible airflow obstruction, recurrent exacerbations. While recent advances in digital health have shown promise, predicting disease progression in patients with ILD and exacerbation in patients with COPD remains challenging. This review explores the role of digital health in managing ILD and COPD, particularly focusing on telehealth and digital health technologies. Telehealth, defined broadly as the use of electronic information and telecommunications technologies in healthcare, has become increasingly relevant, especially during the COVID-19 pandemic. This review examines the role of digital health technologies in the management of ILD and COPD, with particular focus on telemedicine, and digital health tools. Remote monitoring technologies, including home spirometry and wearable devices, have demonstrated feasibility in managing respiratory diseases. However, challenges such as evidence, data reliability, varying adherence, education, and the high costs of data collection and lack of qualified clinicians present barriers for many national health systems. Copyright © 2025 Althobiani, Russell, Jacob, Ranjan, Ahmad, Folarin, Hurst and Porter.	https://www.scopus.com/inward/record.uri?eid=2-s	Q1	Scopus	EXC_PubType
Early diagnosis and real-time monitoring of regional lung functior	2021	First-and second-hand exposure to smoke or air pollutants is the primary cause of chronic obstructive pulmonary disease (COPD) pathogenesis, where genetic and age-related factors pre-dispose the subject to the initiation and progression of obstructive lung disease. Briefly, airway inflammation, specifically bronchitis, initiates the lung disease, leading to difficulty in breathing (dys-pnea) and coughing as initial symptoms, followed by air trapping and inhibition of the flow of air into the lungs due to damage to the alveoli (emphysema). In addition, mucus obstruction and impaired lung clearance mechanisms lead to recurring acute exacerbations causing progressive decline in lung function, eventually requiring lung transplant and other lifesaving interventions to prevent mortality. It is noteworthy that COPD is much more common in the population than currently diagnosed, as only 16 million adult Americans were reported to be diagnosed with COPD as of 2018, although an additional 14 million American adults were estimated to be suffering from COPD but undiagnosed by the current standard of care (SOC) diagnostic, namely the spirometry-based pulmonary function test (PFT). Thus, the main issue driving the adverse disease outcome and significant mortality for COPD is lack of timely diagnosis in the early stages of the disease. The current treatment regime for COPD emphysema is most effective when implemented early, on COPD onset, where alleviating symptoms and exacerbations with timely intervention(s) can prevent steep lung function decline(s) and disease progression to severe emphysema. Therefore, the key to efficiently combatting COPD relies on early detection. Thus, it is important to detect early regional pulmonary function and structural changes to monitor modest disease progression for implementing timely interventions and effectively eliminating emphysema progression. Currently, COPD diagnosis involves using techniques such as COPD screening questionnaires, PFT, arterial blood gas analysis, and/or lung imaging, but these modalities are limited in their capability for early diagnosis and real-time disease monitoring of regional lung function changes. Hence, promising emerging techniques, such as X-ray phase contrast, photoacoustic tomography, ultrasound computed tomography, electrical impedance tomography, the forced oscillation technique, and the impulse oscillometry system powered by robust artificial intelligence and machine learning analysis capability are emerging as novel solu-tions for early detection and real time monitoring of COPD progression for timely intervention. We discuss here the scope, risks, and limitations of current SOC and emerging COPD diagnostics, with perspective on novel diagnostics providing real time regional lung function monitoring, and predicting exacerbation and/or disease onset for prognosis-based timely intervention(s) to limit COPD–emphysema progression. © 2021 by the authors. Licensee MDPI, Basel, Switzerland.	https://www.scopus.com/inward/record.uri?eid=2-s	Q1	Scopus	EXC_PubType

Artificial Intelligence and Machine Learning in Chronic Airway Dis	2021	Chronic airway diseases are characterized by airway inflammation, obstruction, and remodeling and show high prevalence, especially in developing countries. Among them, asthma and chronic obstructive pulmonary disease (COPD) show the highest morbidity and socioeconomic burden worldwide. Although there are extensive guidelines for the prevention, early diagnosis, and rational treatment of these lifelong diseases, their value in precision medicine is very limited. Artificial intelligence (AI) and machine learning (ML) techniques have emerged as effective methods for mining and integrating large-scale, heterogeneous medical data for clinical practice, and several AI and ML methods have recently been applied to asthma and COPD. However, very few methods have significantly contributed to clinical practice. Here, we review four aspects of AI and ML implementation in asthma and COPD to summarize existing knowledge and indicate future steps required for the safe and effective application of AI and ML tools by clinicians.	http://dx.doi.org/10.7150/ijms.58191	Q1	WoS	EXC_PubType
Assessing the Impact of New Technologies on Managing Chronic	2024	Chronic respiratory diseases (CRDs), including asthma and chronic obstructive pulmonary disease (COPD), represent significant global health challenges, contributing to substantial morbidity and mortality. As the prevalence of CRDs continues to rise, particularly in low-income countries, there is a pressing need for more efficient and personalized approaches to diagnosis and treatment. This article explores the impact of emerging technologies, particularly artificial intelligence (AI), on the management of CRDs. AI applications, including machine learning (ML), deep learning (DL), and large language models (LLMs), are transforming the landscape of CRD care, enabling earlier diagnosis, personalized treatment, and enhanced remote patient monitoring. The integration of AI with telehealth and wearable technologies further supports proactive interventions and improved patient outcomes. However, challenges remain, including issues related to data quality, algorithmic bias, and ethical concerns such as patient privacy and AI transparency. This paper evaluates the effectiveness, accessibility, and ethical implications of AI-driven tools in CRD management, offering insights into their potential to shape the future of respiratory healthcare. The integration of AI and advanced technologies in managing CRDs like COPD and asthma holds substantial potential for enhancing early diagnosis, personalized treatment, and remote monitoring, though challenges remain regarding data quality, ethical considerations, and regulatory oversight.	http://dx.doi.org/10.3390/jcm13226913	Q1	WoS	EXC_PubType
Experimental drugs in clinical trials for COPD: artificial intelligenc	2023	IntroductionTherapeutic advances in drug therapy of chronic obstructive pulmonary disease (COPD) really effective in suppressing the pathological processes underlying the disease deterioration are still needed. Artificial Intelligence (AI) via Machine Learning (ML) may represent an effective tool to predict clinical development of investigational agents. Areal coveredExperimental drugs in Phase I and II development for COPD from early 2014 to late 2022 were identified in the ClinicalTrials.gov database. Different ML models, trained from prior knowledge on clinical trial success, were used to predict the probability that experimental drugs will successfully advance toward approval in COPD, according to Bayesian inference as follows: & LE;25% low probability, >25% and & LE;50% moderate probability, >50% and & LE;75% high probability, and >75% very high probability.Expert opinionThe Artificial Neural Network and Random Forest ML models indicated that, among the current experimental drugs in clinical trials for COPD, only the bifunctional muscarinic antagonist - & beta;(2)-adrenoceptor agonists (MABA) navafenterol and batefenterol, the inhaled corticosteroid (ICS)/MABA fluticasone furoate/batefenterol, and the bifunctional phosphodiesterase (PDE) 3/4 inhibitor ensifentrine resulted to have a moderate to very high probability of being approved in the next future, however not before 2025.	http://dx.doi.org/10.1080/13543784.2023.2230138	Q1	WoS	EXC_Scope
Digital Healthcare for Airway Diseases from Personal Environme	2022	Digital technologies have emerged in various dimensions of human life, ranging from education to professional services to wellbeing. In particular, health products and services have expanded by the use and development of artificial intelligence, mobile health applications, and wearable electronic devices. Such advancements have enabled accurate and updated tracking and modeling of health conditions. For instance, digital health technologies are capable of measuring environmental pollution and predicting its adverse health effects. Several health conditions, including chronic airway diseases such as asthma and chronic obstructive pulmonary disease, can be exacerbated by pollution. These diseases impose substantial health burdens with high morbidity and mortality. Recently, efforts have been made to develop digital technologies to alleviate such conditions. Moreover, the COVID-19 pandemic has facilitated the application of telemedicine and telemonitoring for patients with chronic airway diseases. This article reviews current trends and studies in digital technology utilization for investigating and managing environmental exposure and chronic airway diseases. First, we discussed the recent progression of digital technologies in general environmental healthcare. Then, we summarized the capacity of digital technologies in predicting exacerbation and self-management of airway diseases. Concluding these reviews, we provided suggestions to improve digital health technologies' abilities to reduce the adverse effects of environmental exposure in chronic airway diseases, based on personal exposure-response modeling.	http://dx.doi.org/10.3349/ymj.2022.63.S1	Q1	WoS	EXC_PubType
Machine Learning Characterization of COPD Subtypes Insights F	2020	COPD is a heterogeneous syndrome. Many COPD subtypes have been proposed, but there is not yet consensus on how many COPD subtypes there are and how they should be defined. The COPD Genetic Epidemiology Study (COPDGene), which has generated 10-year longitudinal chest imaging, spirometry, and molecular data, is a rich resource for relating COPD phenotypes to underlying genetic and molecular mechanisms. In this article, we place COPDGene clustering studies in context with other highly cited COPD clustering studies, and summarize the main COPD subtype findings from COPDGene. First, most manifestations of COPD occur along a continuum, which explains why continuous aspects of COPD or disease axes may be more accurate and reproducible than subtypes identified through clustering methods. Second, continuous COPD-related measures can be used to create subgroups through the use of predictive models to define cut-points, and we review COPDGene research on blood eosinophil count thresholds as a specific example. Third, COPD phenotypes identified or prioritized through machine learning methods have led to novel biological discoveries, including novel emphysema genetic risk variants and systemic inflammatory subtypes of COPD. Fourth, trajectory-based COPD subtyping captures differences in the longitudinal evolution of COPD, addressing a major limitation of clustering analyses that are confounded by disease severity. Ongoing longitudinal characterization of subjects in COPDGene will provide useful insights about the relationship between lung imaging parameters, molecular markers, and COPD progression that will enable the identification of subtypes based on underlying disease processes and distinct patterns of disease progression, with the potential to improve the clinical relevance and reproducibility of COPD subtypes.	http://dx.doi.org/10.1016/j.chest.2019.11.039	Q1	WoS	EXC_Scope
Predictive performance and impact of algorithms in remote monit	2021	Background: The use of telehealth interventions, such as the remote monitoring of patient clinical data (e.g. blood pressure, blood glucose, heart rate, medication use), has been proposed as a strategy to better manage chronic conditions and to reduce the impact on patients and healthcare systems. The use of algorithms for data acquisition, analysis, transmission, communication and visualisation are now common in remote patient monitoring. However, their use and impact on chronic disease management has not been systematically investigated. Objectives: To investigate the use, impact, and performance of remote monitoring algorithms across various types of chronic conditions. Methods: A literature search of MEDLINE complete, CINHAL complete, and EMBASE was performed using search terms relating to the concepts of remote monitoring, chronic conditions, and data processing algorithms. Comparable outcomes from studies describing the impact on process measures and clinical and patient-reported outcomes were pooled for a summary effect and meta-analyses. A comparison of studies reporting the predictive performance of algorithms was also conducted using the Youden Index. Results: A total of 89 articles were included in the review. There was no evidence of a positive impact on healthcare utilisation [OR 1.09 (0.90 to 1.31); P = .35] and mortality [OR 0.83 (0.63 to 1.10); P = .208], but there was a positive effect on generic health status [SDM 0.2912 (0.06 to 0.51); P = .010] and diabetes control [SDM-0.53 (-0.74 to-0.33); P < .001; I-2 = 15.71] (with two of the three diabetes studies being identified as having a high risk of bias). While the majority of impact studies made use of heuristic threshold-based algorithms (n = 27,87%), most performance studies (n = 36, 62%) analysed non-sequential machine learning methods. There was considerable variance in the quality, sample size and performance amongst these studies. Overall, algorithms involved in diagnosis (n = 22, 47%) had superior performance to those involved in predicting a future event (n = 25, 53%). Detection of arrythmia and ischaemia utilising ECG data showed particularly promising results. Conclusion: The performance of data processing algorithms for the diagnosis of a current condition, particularly those related to the detection of arrythmia and ischaemia, is promising. However, there appears to exist minimal testing in experimental studies, with only two included impact studies citing a performance study as support for the intervention algorithm used. Because of the disconnect between performance and impact studies, there is currently limited evidence of the effect of integrating advanced inference algorithms in remote monitoring interventions. If the field of remote patient monitoring is to progress, future impact studies should address this disconnect by evaluating high performance validated algorithms in robust clinical trials.	http://dx.doi.org/10.1016/j.ijmedinf.2021.104620	Q1	WoS	EXC_Scope

Telerehabilitation for chronic respiratory disease	2021	BACKGROUND: Pulmonary rehabilitation is a proven, effective intervention for people with chronic respiratory diseases including chronic obstructive pulmonary disease (COPD), interstitial lung disease (ILD) and bronchiectasis. However, relatively few people attend or complete a program, due to factors including a lack of programs, issues associated with travel and transport, and other health issues. Traditionally, pulmonary rehabilitation is delivered in-person on an outpatient basis at a hospital or other healthcare facility (referred to as centre-based pulmonary rehabilitation). Newer, alternative modes of pulmonary rehabilitation delivery include home-based models and the use of telehealth. Telerehabilitation is the delivery of rehabilitation services at a distance, using information and communication technology. To date, there has not been a comprehensive assessment of the clinical efficacy or safety of telerehabilitation, or its ability to improve uptake and access to rehabilitation services, for people with chronic respiratory disease. OBJECTIVES: To determine the effectiveness and safety of telerehabilitation for people with chronic respiratory disease. SEARCH METHODS: We searched the Cochrane Airways Trials Register, and the Cochrane Central Register of Controlled Trials; six databases including MEDLINE and Embase; and three trials registries, up to 30 November 2020. We checked reference lists of all included studies for additional references, and handsearched relevant respiratory journals and meeting abstracts. SELECTION CRITERIA: All randomised controlled trials and controlled clinical trials of telerehabilitation for the delivery of pulmonary rehabilitation were eligible for inclusion. The telerehabilitation intervention was required to include exercise training, with at least 50% of the rehabilitation intervention being delivered by telerehabilitation. DATA COLLECTION AND ANALYSIS: We used standard methods recommended by Cochrane. We assessed the risk of bias for all studies, and used the ROBINS-I tool to assess bias in non-randomised controlled clinical trials. We assessed the certainty of evidence with GRADE. Comparisons were telerehabilitation compared to traditional in-person (centre-based) pulmonary rehabilitation, and telerehabilitation compared to no rehabilitation. We analysed studies of telerehabilitation for maintenance rehabilitation separately from trials of telerehabilitation for initial primary pulmonary rehabilitation. MAIN RESULTS: We included a total of 15 studies (32 reports) with 1904 participants, using five different models of telerehabilitation. Almost all (99%) participants had chronic obstructive pulmonary disease (COPD). Three studies were controlled clinical trials. For primary pulmonary rehabilitation, there was probably little or no difference between telerehabilitation and in-person pulmonary rehabilitation for exercise capacity measured as 6-Minute Walking Distance (6MWD) (mean difference (MD) 0.06 metres (m), 95% confidence interval (CI) -10.82 m to 10.94 m; 556 participants; four studies; moderate-certainty evidence). There may also be little or no difference for quality of life measured with the St George's Respiratory Questionnaire (SGRQ) total score (MD -1.26, 95% CI -3.97 to 1.45; 274 participants; two studies; low-certainty evidence), or for breathlessness on the Chronic Respiratory Questionnaire (CRQ) dyspnoea domain score (MD 0.13, 95% CI -0.13 to 0.40; 426 participants; three studies; low-certainty evidence). Participants were more likely to complete a program of telerehabilitation, with a 93% completion rate (95% CI 90% to 96%), compared to a 70% completion rate for in-person rehabilitation. When compared to no rehabilitation control, trials of primary telerehabilitation may increase exercise capacity on 6MWD (MD 22.17 m, 95% CI -38.89 m to 83.23 m; 94 participants; two studies; low-certainty evidence) and may also increase 6MWD when delivered as maintenance rehabilitation (MD 78.1 m, 95% CI 49.6 m to 106.6 m; 209 participants; two studies; low-certainty evidence). No adverse effects of telerehabilitation were noted over and above any reported for in-person rehabilitation or no rehabilitation. AUTHORS' CONCLUSIONS: This review suggests that primary pulmonary rehabilitation, or maintenance rehabilitation, delivered via telerehabilitation for people with chronic respiratory disease achieves outcomes similar to those of traditional centre-based pulmonary rehabilitation, with no safety issues identified. However, the certainty of the evidence provided by this review is limited by the small number of studies, of varying telerehabilitation models, with relatively few participants. Future research should consider the clinical effect of telerehabilitation for individuals with chronic respiratory diseases other than COPD, the duration of benefit of telerehabilitation beyond the period of the intervention, and the economic cost of telerehabilitation.	https://pubmed.ncbi.nlm.nih.gov/33511633/	Q2	PubMed	EXC_Scope
Telehealth interventions: remote monitoring and consultations for	2021	BACKGROUND: Chronic obstructive pulmonary disease (COPD, including bronchitis and emphysema) is a chronic condition causing shortness of breath, cough, and exacerbations leading to poor health outcomes. Face-to-face visits with health professionals can be hindered by severity of COPD or frailty, and by people living at a distance from their healthcare provider and having limited access to services. Telehealth technologies aimed at providing health care remotely through monitoring and consultations could help to improve health outcomes of people with COPD. OBJECTIVES: To assess the effectiveness of telehealth interventions that allow remote monitoring and consultation and multi-component interventions for reducing exacerbations and improving quality of life, while reducing dyspnoea symptoms, hospital service utilisation, and death among people with COPD. SEARCH METHODS: We identified studies from the Cochrane Airways Trials Register. Additional sources searched included the US National Institutes of Health Ongoing Trials Register, the World Health Organization International Clinical Trials Registry Platform, and the IEEEX Xplore Digital Library. The latest search was conducted in April 2020. We used the GRADE approach to judge the certainty of evidence for outcomes. SELECTION CRITERIA: Eligible randomised controlled trials (RCTs) included adults with diagnosed COPD. Asthma, cystic fibrosis, bronchiectasis, and other respiratory conditions were excluded. Interventions included remote monitoring or consultation plus usual care, remote monitoring or consultation alone, and mult-component interventions from all care settings. Quality of life scales included St George's Respiratory Questionnaire (SGRQ) and the COPD Assessment Test (CAT). The dyspnoea symptom scale used was the Chronic Respiratory Disease Questionnaire Self-Administered Standardized Scale (CRQ-SAS). DATA COLLECTION AND ANALYSIS: We used standard Cochrane methodological procedures. We assessed confidence in the evidence for each primary outcome using the GRADE method. Primary outcomes were exacerbations, quality of life, dyspnoea symptoms, hospital service utilisation, and mortality; a secondary outcome consisted of adverse events. MAIN RESULTS: We included 29 studies in the review (5654 participants; male proportion 36% to 96%; female proportion 4% to 61%). Most remote monitoring interventions required participants to transfer measurements using a remote device and later health professional review (asynchronous). Only five interventions transferred data and allowed review by health professionals in real time (synchronous). Studies were at high risk of bias due to lack of blinding, and certainty of evidence ranged from moderate to very low. We found no evidence on comparison of remote consultations with or without usual care. Remote monitoring plus usual care (8 studies, 1033 participants) Very uncertain evidence suggests that remote monitoring plus usual care may have little to no effect on the number of people experiencing exacerbations at 26 weeks or 52 weeks. There may be little to no difference in effect on quality of life (SGRQ) at 26 weeks (very low to low certainty) or on hospitalisation (all-cause or COPD-related; very low certainty). COPD-related hospital re-admissions are probably reduced at 26 weeks (hazard ratio 0.42, 95% confidence interval (CI) 0.19 to 0.93; 106 participants; moderate certainty). There may be little to no difference in deaths between intervention and usual care (very low certainty). We found no evidence for dyspnoea symptoms or adverse events. Remote monitoring alone (10 studies, 2456 participants) Very uncertain evidence suggests that remote monitoring may result in little to no effect on the number of people experiencing exacerbations at 41 weeks (odds ratio 1.02, 95% CI 0.67 to 1.55). There may be little to no effect on quality of life (SGRQ total at 17 weeks, or CAT at 38 and 52 weeks; very low certainty). There may be little to no effect on dyspnoea symptoms on the CRQ-SAS at 26 weeks (low certainty). There may be no difference in effects on the number of people admitted to hospital (very low certainty) or on deaths (very low certainty). We found no evidence for adverse events. Multi-component interventions with remote monitoring or consultation component (11 studies, 2165 participants) Very uncertain evidence suggests that multi-component interventions may have little to no effect on the number of people experiencing exacerbations at 52 weeks. Quality of life at 13 weeks may improve as seen in SGRQ total score (mean difference -9.70, 95% CI -18.32 to -1.08; 38 participants; low certainty) but not at 26 or 52 weeks (very low certainty). COPD assessment test (CAT) scores may improve at a mean of 38 weeks, but evidence is very uncertain and interventions are varied. There may be little to no effect on the number of people admitted to hospital at 33 weeks (low certainty). Multi-component interventions are likely to result in fewer people re-admitted to hospital at a mean of 39 weeks (OR 0.50, 95% CI 0.31 to 0.81; 344 participants, 3 studies; moderate certainty). There may be little to no difference in death at a mean of 40 weeks (very low certainty). There may be little to no effect on people experiencing adverse events (very low certainty). We found no evidence for dyspnoea symptoms. AUTHORS' CONCLUSIONS: Remote monitoring plus usual care provided asynchronously may not be beneficial overall compared to usual care alone. Some benefit is seen in reduction of COPD-related hospital re-admissions, but moderate-certainty evidence is based on one study. We have not found any evidence for dyspnoea symptoms nor harms, and there is no difference in fatalities when remote monitoring is provided in addition to usual care. Remote monitoring interventions alone are no better than usual care overall for health outcomes. Multi-component interventions with asynchronous remote monitoring are no better than usual care but may provide short-term benefit for quality of life and may result in fewer re-admissions to hospital for any cause. We are uncertain whether remote monitoring is responsible for the positive impact on re-admissions, and we are unable to discern the long-term benefits of receiving remote monitoring as part of patient care. Owing to paucity of evidence, it is unclear which COPD severity subgroups would benefit from telehealth interventions. Given there is no evidence of harm, telehealth interventions may be beneficial as an additional health resource depending on individual needs based on professional assessment. Larger studies can determine long-term effects of these interventions.	https://www.ncbi.nlm.nih.gov/pmc/articles/PMC854	Q2	PubMed	EXC_Scope

Telehealth interventions to support self-management of long-term	2017	BACKGROUND: Self-management support is one mechanism by which telehealth interventions have been proposed to facilitate management of long-term conditions. OBJECTIVE: The objectives of this metareview were to (1) assess the impact of telehealth interventions to support self-management on disease control and health care utilization, and (2) identify components of telehealth support and their impact on disease control and the process of self-management. Our goal was to synthesise evidence for telehealth-supported self-management of diabetes (types 1 and 2), heart failure, asthma, chronic obstructive pulmonary disease (COPD) and cancer to identify components of effective self-management support. METHODS: We performed a metareview (a systematic review of systematic reviews) of randomized controlled trials (RCTs) of telehealth interventions to support self-management in 6 exemplar long-term conditions. We searched 7 databases for reviews published from January 2000 to May 2016 and screened identified studies against eligibility criteria. We weighted reviews by quality (revised A Measurement Tool to Assess Systematic Reviews), size, and relevance. We then combined our results in a narrative synthesis and using harvest plots. RESULTS: We included 53 systematic reviews, comprising 232 unique RCTs. Reviews concerned diabetes (type 1: n=6; type 2, n=11; mixed, n=19), heart failure (n=9), asthma (n=8), COPD (n=8), and cancer (n=3). Findings varied between and within disease areas. The highest-weighted reviews showed that blood glucose telemonitoring with feedback and some educational and lifestyle interventions improved glycemic control in type 2, but not type 1, diabetes, and that telemonitoring and telephone interventions reduced mortality and hospital admissions in heart failure, but these findings were not consistent in all reviews. Results for the other conditions were mixed, although no reviews showed evidence of harm. Analysis of the mediating role of self-management, and of components of successful interventions, was limited and inconclusive. More intensive and multifaceted interventions were associated with greater improvements in diabetes, heart failure, and asthma. CONCLUSIONS: While telehealth-mediated self-management was not consistently superior to usual care, none of the reviews reported any negative effects, suggesting that telehealth is a safe option for delivery of self-management support, particularly in conditions such as heart failure and type 2 diabetes, where the evidence base is more developed. Larger-scale trials of telehealth-supported self-management, based on explicit self-management theory, are needed before the extent to which telehealth technologies may be harnessed to support self-management can be established.	https://www.ncbi.nlm.nih.gov/pmc/articles/PMC545	Q2	PubMed	EXC_Scope
Tailored or adapted interventions for adults with chronic obstructi	2021	BACKGROUND: Chronic obstructive pulmonary disease (COPD) is a chronic respiratory condition characterised by shortness of breath, cough and recurrent exacerbations. People with COPD often live with one or more co-existing long-term health conditions (comorbidities). People with more severe COPD often have a higher number of comorbidities, putting them at greater risk of morbidity and mortality. OBJECTIVES: To assess the effectiveness of any single intervention for COPD adapted or tailored to their comorbidity(s) compared to any other intervention for people with COPD and one or more common comorbidities (quantitative data, RCTs) in terms of the following outcomes: Quality of life, exacerbations, functional status, all-cause and respiratory-related hospital admissions, mortality, pain, and depression and anxiety. To assess the effectiveness of an adapted or tailored single COPD intervention (simple or complex) that is aimed at changing the management of people with COPD and one or more common comorbidities (quantitative data, RCTs) compared to usual care in terms of the following outcomes: Quality of life, exacerbations, functional status, all-cause and respiratory-related hospital admissions, mortality, pain, and depression and anxiety. To identify emerging themes that describe the views and experiences of patients, carers and healthcare professionals when receiving or providing care to manage multimorbidities (qualitative data). SEARCH METHODS: We searched multiple databases including the Cochrane Airways Trials Register, CENTRAL, MEDLINE, Embase, and CINAHL, to identify relevant randomised and qualitative studies. We also searched trial registries and conducted citation searches. The latest search was conducted in January 2021. SELECTION CRITERIA: Eligible randomised controlled trials (RCTs) compared a) any single intervention for COPD adapted or tailored to their comorbidity(s) compared to any other intervention, or b) any adapted or tailored single COPD intervention (simple or complex) that is aimed at changing the management of people with COPD and one or more comorbidities, compared to usual care. We included qualitative studies or mixed-methods studies to identify themes. DATA COLLECTION AND ANALYSIS: We used standard Cochrane methods for analysis of the RCTs. We used Cochrane's risk of bias tool for the RCTs and the CASP checklist for the qualitative studies. We planned to use the Mixed Methods Appraisal tool (MMAT) to assess the risk of bias in mixed-methods studies, but we found none. We used GRADE and CERQual to assess the quality of the quantitative and qualitative evidence respectively. The primary outcome measures for this review were quality of life and exacerbations. MAIN RESULTS: Quantitative studies We included seven studies (1197 participants) in the quantitative analyses, with interventions including telemonitoring, pulmonary rehabilitation, treatment optimisation, water-based exercise training and case management. Interventions were either compared with usual care or with an active comparator (such as land-based exercise training). Duration of trials ranged from 4 to 52 weeks. Mean age of participants ranged from 64 to 72 years and COPD severity ranged from mild to very severe. Trials included either people with COPD and a specific comorbidity (including cardiovascular disease, metabolic syndrome, lung cancer, head or neck cancer, and musculoskeletal conditions), or with one or more comorbidities of any type. Overall, we judged the evidence presented to be of moderate to very low certainty (GRADE), mainly due to the methodological quality of included trials and imprecision of effect estimates. Intervention versus usual care Quality of life as measured by the St George's Respiratory Questionnaire (SGRQ) total score may improve with tailored pulmonary rehabilitation compared to usual care at 52 weeks (mean difference (MD) -10.85, 95% confidence interval (CI) -12.66 to -9.04; 1 study, 70 participants; low-certainty evidence). Tailored pulmonary rehabilitation is likely to improve COPD assessment test (CAT) scores compared with usual care at 52 weeks (MD -8.02, 95% CI -9.44 to -6.60; 1 study, 70 participants, moderate-certainty evidence) and with a multicomponent telehealth intervention at 52 weeks (MD -6.90, 95% CI -9.56 to -4.24; moderate-certainty evidence). Evidence is uncertain about effects of pharmacotherapy optimisation or telemonitoring interventions on CAT improvement compared with usual care. There may be little to no difference in the number of people experiencing exacerbations, or mean exacerbations with case management compared with usual care (OR 1.09, 95% CI 0.75 to 1.57; 1 study, 470 participants; very low-certainty evidence). For secondary outcomes, six-minute walk distance (6MWD) may improve with pulmonary rehabilitation, water-based exercise or multicomponent interventions at 38 to 52 weeks (low-certainty evidence). A multicomponent intervention may result in fewer people being admitted to hospital at 17 weeks, although there may be little to no difference in a telemonitoring intervention. There may be little to no difference between intervention and usual care for mortality. Intervention versus active comparator We included one study comparing water-based and land-based exercise (30 participants). We found no evidence for quality of life or exacerbations. There may be little to no difference between water- and land-based exercise for 6MWD (MD 5 metres, 95% CI -22 to 32; 38 participants; very low-certainty evidence). Qualitative studies One nested qualitative study (21 participants) explored perceptions and experiences of people with COPD and long-term conditions, and of researchers and health professionals who were involved in an RCT of telemonitoring equipment. Several themes were identified, including health status, beliefs and concerns, reliability of equipment, self-efficacy, perceived ease of use, factors affecting usefulness and perceived usefulness, attitudes and intention, self-management and changes in healthcare use. We judged the qualitative evidence presented as of very low certainty overall. AUTHORS' CONCLUSIONS: Owing to a paucity of eligible trials, as well as diversity in the intervention type, comorbidities and the outcome measures reported, we were unable to provide a robust synthesis of data. Pulmonary rehabilitation or multicomponent interventions may improve quality of life and functional status (6MWD), but the evidence is too limited to draw a robust conclusion. The key take-home message from this review is the lack of data from RCTs on treatments for people living with COPD and comorbidities. Given the variation in number and type of comorbidity(s) an individual may have, and severity of COPD, larger studies reporting individual patient data are required to determine these effects.	https://www.ncbi.nlm.nih.gov/pmc/articles/PMC840	Q2	PubMed	EXC_Scope
Clinical-effectiveness of self-management interventions in chroni	2017	Self-management (SM) is defined as the provision of interventions to increase patients' skills and confidence, empowering the individual to take an active part in their disease management. There is uncertainty regarding the optimal format and the short- and long-term benefits of chronic obstructive pulmonary disease (COPD) SM interventions in adults. Therefore, a high-quality overview of reviews was updated to examine their clinical effectiveness. Sixteen reviews were identified, interventions were broadly classified as education or action plans, complex interventions with an SM focus, pulmonary rehabilitation (PR), telehealth and outreach nursing. Systematic review and meta-analysis quality and the risk of bias of underlying primary studies were assessed. Strong evidence was found that PR is associated with significant improvements in health-related quality of life (HRQoL). Limited to moderate evidence for complex interventions (SM focus) with limited evidence for education, action plans, telehealth interventions and outreach nursing for HRQoL was found. There was strong evidence that education is associated with a significant reduction in COPD-related hospital admissions, moderate to strong evidence that telehealth interventions and moderate evidence that complex interventions (SM focus) are associated with reduced health care utilization. These findings from a large body of evidence suggesting that SM, through education or as a component of PR, confers significant health gains in people with COPD in terms of HRQoL. SM supported by telehealth confers significant reductions in healthcare utilization, including hospitalization and emergency department visits.	https://www.ncbi.nlm.nih.gov/pmc/articles/PMC572	Q2	PubMed	EXC_Scope

Toward a digital platform for the self-management of noncommun	2020	BACKGROUND: Digital interventions are effective for health behavior change, as they enable the self-management of chronic, noncommunicable diseases (NCDs). However, they often fail to facilitate the specific or current needs and preferences of the individual. A proposed alternative is a digital platform that hosts a suite of discrete, already existing digital health interventions. A platform architecture would allow users to explore a range of evidence-based solutions over time to optimize their self-management and health behavior change. OBJECTIVE: This review aims to identify digital platform-like interventions and examine their potential for supporting self-management of NCDs and health behavior change. METHODS: A literature search was conducted in January 2020 using EBSCOhost, PubMed, Scopus, and EMBASE. No digital platforms were identified, so criteria were broadened to include digital platform-like interventions. Eligible platform-like interventions offered a suite of discrete, evidence-based health behavior change features to optimize self-management of NCDs in an adult population and provided digitally supported guidance for the user toward the features best suited to their needs and preferences. Data collected on interventions were guided by the CONSORT-EHEALTH (Consolidated Standards of Reporting Trials of Electronic and Mobile Health Applications and Online Telehealth) checklist, including evaluation data on effectiveness and process outcomes. The quality of the included literature was assessed using the Mixed Methods Appraisal Tool. RESULTS: A total of 7 studies were included for review. Targeted NCDs included cardiovascular diseases (CVD; n=3), diabetes (n=3), and chronic obstructive pulmonary disease (n=1). The mean adherence (based on the number of follow-up responders) was 69% (SD 20%). Of the 7 studies, 4 with the highest adherence rates (80%) were also guided by behavior change theories and took an iterative, user-centered approach to development, optimizing intervention relevance. All 7 interventions presented algorithm-supported user guidance tools, including electronic decision support, smart features that interact with patterns of use, and behavior change stage-matching tools. Of the 7 studies, 6 assessed changes in behavior. Significant effects in moderate-to-vigorous physical activity were reported, but for no other specific health behaviors. However, positive behavior change was observed in studies that focused on comprehensive behavior change measures, such as self-care and self-management, each of which addresses several key lifestyle risk factors (eg, medication adherence). No significant difference was found for psychosocial outcomes (eg, quality of life). Significant changes in clinical outcomes were predominately related to disease-specific, multifaceted measures such as clinical disease control and cardiovascular risk score. CONCLUSIONS: Iterative, user-centered development of digital platform structures could optimize user engagement with self-management support through existing, evidence-based digital interventions. Offering a palette of interventions with an appropriate degree of guidance has the potential to facilitate disease-specific health behavior change and effective self-management among a myriad of users, conditions, or stages of care.	https://www.ncbi.nlm.nih.gov/pmc/articles/PMC765	Q2	PubMed	EXC_Scope
Do telemedical interventions improve quality of life in patients wit	2016	OBJECTIVE: Telehealth is an approach to disease management, which may hold the potential of improving some of the features associated with COPD, including positive impact on disease progression, and thus possibly limiting further reduction in quality of life (QoL). Our objective was, therefore, to summarize studies addressing the impact of telehealth on QoL in patients with COPD. DESIGN: Systematic review. METHODS: A series of systematic searches were carried out using the following databases: PubMed, EMBASE, Cochrane Controlled Trials Register, and ClinicalTrials.gov (last updated November 2015). A predefined search algorithm was utilized with the intention to capture all results related to COPD, QoL, and telehealth published since year 2000. OUTCOME MEASURES: Primary outcome was QoL, assessed by validated measures. RESULTS: Out of the 18 studies fulfilling the criteria for inclusion in this review, three studies found statistically significant improvements in QoL for patients allocated to telemedical interventions. However, all of the other included studies found no statistically significant differences between control and telemedical intervention groups in terms of QoL. CONCLUSION: Telehealth does not make a strong case for itself when exclusively looking at QoL as an outcome, since statistically significant improvements relative to control groups have been observed only in few of the available studies. Nonetheless, this does not only rule out the possibility that telehealth is superior to standard care with regard to other outcomes but also seems to call for more research, not least in large-scale controlled trials.	https://www.ncbi.nlm.nih.gov/pmc/articles/PMC484	Q2	PubMed	EXC_Scope
Advances in remote respiratory assessments for people with chr	2018	BACKGROUND: Chronic obstructive pulmonary disease (COPD) is a leading cause of mortality. Advances in remote technologies and telemedicine provide new ways to monitor respiratory function and improve chronic disease management. However, telemedicine does not always include remote respiratory assessments, and the current state of knowledge for people with COPD has not been evaluated. OBJECTIVE: Systematically review the use of remote respiratory assessments in people with COPD, including the following questions: What devices have been used? Can acute exacerbations of chronic obstructive pulmonary disease (AECOPD) be predicted by using remote devices? Do remote respiratory assessments improve health-related outcomes? MATERIALS AND METHODS: The review protocol was registered (PROSPERO 2016:CRD42016049333). MEDLINE, EMBASE, and COMPENDEX databases were searched for studies that included remote respiratory assessments in people with COPD. A narrative synthesis was then conducted by two reviewers according to Preferred Reporting Items for Systematic Reviews and Meta-Analyses (PRISMA) guidelines. RESULTS: Fifteen studies met the inclusion criteria. Forced expiratory volume assessed daily by using a spirometer was the most common modality. Other measurements included resting respiratory rate, respiratory sounds, and end-tidal carbon dioxide level. Remote assessments had high user satisfaction. Benefits included early detection of AECOPD, improved health-related outcomes, and the ability to replace hospital care with a virtual ward. CONCLUSION: Remote respiratory assessments are feasible and when combined with sufficient organizational backup can improve health-related outcomes in some but not all cohorts. Future research should focus on the early detection, intervention, and rehabilitation for AECOPD in high-risk people who have limited access to best care and investigate continuous as well as intermittent monitoring.	https://www.ncbi.nlm.nih.gov/pubmed/29083268	Q2	PubMed	EXC_Scope
Impact of clinical pharmacist services delivered via telemedicine	2018	BACKGROUND: Utilization of telemedicine allows pharmacists to extend the reach of clinical interventions, connecting them with patients and providers, but the overall impact of these services is under-studied. OBJECTIVE: Identify the impact of clinical pharmacist telemedicine interventions on clinical outcomes, subsequently defined as clinical disease management, patient self-management, and adherence, in outpatient or ambulatory settings. METHODS: A literature search was conducted from database inception through May 2016 in Medline, SCOPUS, and EMBASE. Broad terms "telemedicine", "telehealth", and "telephone" were used in combination with "pharmacist" or "pharmacy" and "telepharmacy". The search and extraction process followed PRISMA guidelines. Results were screened for pharmacist interventions and reviewed to identify studies in outpatient our ambulatory settings. Studies of non-clinical outcomes (i.e. dispensing or product preparation) and with no comparator were excluded. The final studies were categorized by types of outcomes reported: clinical disease management, patient self-management, and adherence. RESULTS: Only 34 studies measured clinical outcomes against a comparator, consistent with the research question. The majority utilized scheduled models of care (n = 29). Telephone was the most common communication method (n = 25). The most utilized interventions were pharmacist-led telephonic clinics (n = 10). Most studies focused on chronic disease management in adults including hypertension, diabetes, anticoagulation, depression, hyperlipidemia, asthma, heart failure, HIV, PTSD, CKD, stroke, COPD and smoking cessation. Twenty-three studies had a positive impact with one reporting negative results. Higher positive impact rate was observed for scheduled (72.4%, 21/29) and continuous (100%, 2/2) models compared to responsive/reactive (25%, 1/4). CONCLUSIONS: Clinical pharmacy telemedicine interventions in the outpatient or ambulatory setting, primarily via phone, have an overall positive impact on outcomes related to clinical disease management, patient self-management, and adherence in the management of chronic diseases. Commonalities among studies with positive impact included utilization of continuous or scheduled models via telephone, with frequent monitoring and interventions. Studies identified did not evaluate benefits of video capability over telephone or cost-effectiveness, both of which are useful directions for future study.	https://www.ncbi.nlm.nih.gov/pubmed/29100941	Q2	PubMed	EXC_Scope
Patient expectations and experiences of remote monitoring for cl	2019	OBJECTIVES: To describe the range of patients' beliefs, attitudes, expectations, and experiences of remote monitoring for chronic conditions across different healthcare contexts and populations. DESIGN: We searched MEDLINE, Embase, PsychINFO, and CINAHL, Google Scholar, and reference lists of related studies through to July 2017. Thematic synthesis was used to analyse the findings of the primary studies. Study characteristics were examined to explain differences in findings. SETTING: All healthcare settings PARTICIPANTS: Adults with chronic diseases OUTCOMES: Patient beliefs, attitudes, expectations and experiences of remote monitoring RESULTS: We included 16 studies involving 307 participants with chronic obstructive pulmonary disease, heart failure, diabetes, hypertension, and end stage kidney disease. The studies were conducted in 8 countries. We identified four themes: gaining knowledge and triggering actions (tracking and responding to change, prompting timely and accessible care, supporting self-management and shared decision-making); reassurance and security (safety in being alone, peace of mind); concern about additional burden (reluctance to learn something new, lack of trust in technology, avoiding additional out-of-pocket costs), and jeopardising interpersonal connections (fear of being lost in data, losing face to face contact). CONCLUSIONS: For patients with chronic disease, remote monitoring increased their disease-specific knowledge, triggered earlier clinical assessment and treatment, improved self-management and shared decision-making. However, these potential benefits were balanced against concerns about losing interpersonal contact, and the additional personal responsibility of remote monitoring.	https://www.ncbi.nlm.nih.gov/pubmed/30784430	Q2	PubMed	EXC_Scope

Effectiveness of telemonitoring for reducing exacerbation occurre	2021	Background: Although an increasing number of studies have reported that telemonitoring (TM) in patients with chronic obstructive pulmonary disease (COPD) can be useful and efficacious for hospitalizations and quality of life, its actual utility in detecting and managing acute exacerbation of COPD (AECOPD) is less established. This meta-analysis aimed to identify the best available evidence on the effectiveness of TM targeting the early and optimized management of AECOPD in patients with a history of past AECOPD compared with a control group without TM intervention. Methods: We systematically searched PubMed, Embase, and the Cochrane Library for randomized controlled trials published from 1990 to May 2020. Primary endpoints included emergency room visits and exacerbation-related readmissions. P-values, risk ratios, odds ratios, and mean differences with 95% confidence intervals were calculated. Results: Of 505 identified citations, 17 original articles with both TM intervention and a control group were selected for the final analysis (N = 3,001 participants). TM was found to reduce emergency room visits [mean difference (MD) -0.70, 95% confidence interval (CI) -1.36 to -0.03], exacerbation-related readmissions (risk ratio 0.74, 95% CI 0.60-0.92), exacerbation-related hospital days (MD -0.60, 95% CI -1.06 to -0.13), mortality (odds ratio 0.71, 95% CI 0.54-0.93), and the St. George's Respiratory Questionnaire (SGRQ) score (MD -3.72, 95% CI -7.18 to -0.26) but did not make a difference with respect to all-cause readmissions, the rate of exacerbation-related readmissions, all-cause hospital days, time to first hospital readmission, anxiety and depression, and exercise capacity. Furthermore, the subgroup analysis by observation period showed that longer TM (≥12 months) was more effective in reducing readmissions. Conclusions: TM can reduce emergency room visits and exacerbation-related readmissions, as well as acute exacerbation (AE)-related hospital days, mortality, and the SGRQ score. The implementation of TM intervention is thus a potential protective therapeutic strategy that could facilitate the long-term management of AECOPD. Systematic Review Registration: This systematic review and meta-analysis is reported in accordance with the Preferred Reporting Items for Systematic Reviews and Meta-Analyses (PRISMA) Statement and was registered at International Prospective Register of Systematic Reviews (number: CRD42020181459).	https://www.ncbi.nlm.nih.gov/pmc/articles/PMC846	Q2	PubMed	EXC_Scope
Remote versus face-to-face check-ups for asthma	2016	BACKGROUND: Asthma remains a significant cause of avoidable morbidity and mortality. Regular check-ups with a healthcare professional are essential to monitor symptoms and adjust medication.Health services worldwide are considering telephone and internet technologies as a way to manage the rising number of people with asthma and other long-term health conditions. This may serve to improve health and reduce the burden on emergency and inpatient services. Remote check-ups may represent an unobtrusive and efficient way of maintaining contact with patients, but it is uncertain whether conducting check-ups in this way is effective or whether it may have unexpected negative consequences. OBJECTIVES: To assess the safety and efficacy of conducting asthma check-ups remotely versus usual face-to-face consultations. SEARCH METHODS: We identified trials from the Cochrane Airways Review Group Specialised Register (CAGR) up to 24 November 2015. We also searched www.clinicaltrials.gov, the World Health Organization (WHO) trials portal, reference lists of other reviews and contacted trial authors for additional information. SELECTION CRITERIA: We included parallel randomised controlled trials (RCTs) of adults or children with asthma that compared remote check-ups conducted using any form of technology versus standard face-to-face consultations. We excluded studies that used automated telehealth interventions that did not include personalised contact with a health professional. We included studies reported as full-text articles, as abstracts only and unpublished data. DATA COLLECTION AND ANALYSIS: Two review authors screened the literature search results and independently extracted risk of bias and numerical data. We resolved any disagreements by consensus, and we contacted study authors for missing information.We analysed dichotomous data as odds ratios (ORs) using study participants as the unit of analysis, and continuous data as mean differences using the random-effects models. We rated all outcomes using the Grading of Recommendations Assessment, Development and Evaluation (GRADE) approach. MAIN RESULTS: Six studies including a total of 2100 participants met the inclusion criteria: we pooled four studies including 792 people in the main efficacy analyses, and presented the results of a cluster implementation study (n = 1213) and an oral steroid tapering study (n = 95) separately. Baseline characteristics relating to asthma severity were variable, but studies generally recruited people with asthma taking regular medications and excluded those with COPD or severe asthma. One study compared the two types of check-up for oral steroid tapering in severe refractory asthma and we assessed it as a separate question. The studies could not be blinded and dropout was high in four of the six studies, which may have biased the results.We could not say whether more people who had a remote check-up needed oral corticosteroids for an asthma exacerbation than those who were seen face-to-face because the confidence intervals (CIs) were very wide (OR 1.74, 95% CI 0.41 to 7.44; 278 participants; one study; low quality evidence). In the face-to-face check-up groups, 21 participants out of 1000 had exacerbations that required oral steroids over three months, compared to 36 (95% CI nine to 139) out of 1000 for the remote check-up group. Exacerbations that needed treatment in the Emergency Department (ED), hospital admission or an unscheduled healthcare visit all happened too infrequently to detect whether remote check-ups are a safe alternative to face-to-face consultations. Serious adverse events were not reported separately from the exacerbation outcomes.There was no difference in asthma control measured by the Asthma Control Questionnaire (ACQ) or in quality of life measured on the Asthma Quality of Life Questionnaire (AQLQ) between remote and face-to-face check-ups. We could rule out significant harm of remote check-ups for these outcomes but we were less confident because these outcomes are more prone to bias from lack of blinding.The larger implementation study that compared two general practice populations demonstrated that offering telephone check-ups and proactively phoning participants increased the number of people with asthma who received a review. However, we do not know whether the additional participants who had a telephone check-up subsequently benefited in asthma outcomes. AUTHORS' CONCLUSIONS: Current randomised evidence does not demonstrate any important differences between face-to-face and remote asthma check-ups in terms of exacerbations, asthma control or quality of life. There is insufficient information to rule out differences in efficacy, or to say whether or not remote asthma check-ups are a safe alternative to being seen face-to-face.	https://www.ncbi.nlm.nih.gov/pmc/articles/PMC840	Q2	PubMed	EXC_Scope
Advanced telehealth technology improves home-based exercise	2021	QUESTIONS: How effective is home-based exercise therapy delivered using advanced telehealth technology (ATT-ET) for people with chronic obstructive pulmonary disease (COPD) compared with: no exercise therapy (ET), in/outpatient ET, and home-based ET without ATT? DESIGN: Systematic review and meta-analysis of randomised trials. PARTICIPANTS: People with stable COPD referred for ET. INTERVENTION: ATT-ET. OUTCOME MEASURES: Exercise capacity, quality of life, functional dyspnoea, cost-effectiveness and various secondary outcomes. RESULTS: Fifteen eligible trials involved 1,522 participants. Compared with no ET, ATT-ET improved exercise capacity (four studies, 6-minute walk test MD 15 m, 95% CI 5 to 24) and probably improved quality of life (four studies, SMD 0.22, 95% CI 0.00 to 0.43) and functional dyspnoea (two studies, Chronic Respiratory Questionnaire-Dyspnoea MD 2, 95% CI 0 to 4). ATT-ET had a similar effect as in/outpatient ET on functional dyspnoea (two studies, SMD -0.05, 95% CI -0.39 to 0.29) and a similar or better effect on quality of life (two studies, SMD 0.23, 95% CI -0.04 to 0.50) but its relative effect on exercise capacity was very uncertain (three studies, 6-minute walk test MD 6 m, 95% CI -26 to 37). ATT-ET had a similar effect as home-based ET without ATT on exercise capacity (three studies, 6-minute walk test MD 2 m, 95% CI -16 to 19) and similar or better effects on quality of life (three studies, SMD 0.79, 95% CI -0.04 to 1.62) and functional dyspnoea (two studies, Chronic Respiratory Questionnaire-Dyspnoea MD 2, 95% CI 0 to 4). ATT-ET had effects on most secondary outcomes that were similar to or better than each comparator. CONCLUSION: ATT-ET improves exercise capacity, functional dyspnoea and quality of life compared with no ET, although some benefits may be small. Its benefits are generally similar to in/outpatient ET and similar to or better than home-based ET without ATT. REGISTRATION: PROSPERO CRD42020165773.	https://www.ncbi.nlm.nih.gov/pubmed/33358547	Q2	PubMed	EXC_Scope
The use of wearable devices in chronic disease management to	2022	INTRODUCTION: Wearable device (WD) interventions are rapidly growing in chronic disease management; nevertheless, the effectiveness of these technologies to monitor telehealth outcomes has not been adequately discussed. This study aims to evaluate the effects of WDs in adherence and other health outcomes for people with chronic obstructive pulmonary disease (COPD), diabetes mellitus (DM), and cardiac disease (CD). METHODS: CINAHL, PsycINFO, CENTRAL, and EMBASE were searched for randomized controlled trials (RCTs) and non-RCTs from 1937 to February 2020. Studies comparing interventions with the use of WD were assessed for quality in RCTs and a meta-analysis was performed. RESULTS: Eleven studies were included in this review. All of the interventions involved WD use with educational support such as goal setting, virtual social support, e-health program, real-time feedback, written information, maintain diary, and text messaging. The meta-analysis showed no difference in adherence (p = .38). The DM group showed effects of more than a 2% reduction in weight when WDs were implemented for three months (risk ratio = 2.20; 95% confidence interval (CI) 1.38 to 3.50; p = .0009), as well as blood glucose (mean difference (MD) = -32.39; 95% CI = -48.07 to -16.72; p < .0001), haemoglobin A1c (MD = -0.69; 95% CI = -1.28 to -0.10; p = .02), and physical exercise time in the CD group (MD = 9.53; 95% CI = 0.59 to 18.47; p = .04). DISCUSSION: WD with educational support may be particularly useful for people with DM and CD to enhance support beyond usual care. The results of this review showed insufficient evidence to support the use of WD for COPD to enhance telehealth outcomes for disease management.	https://www.ncbi.nlm.nih.gov/pubmed/32819184	Q2	PubMed	EXC_Scope

Blended self-management interventions to reduce disease burde	2021	BACKGROUND: Chronic obstructive pulmonary disease (COPD) and asthma have a high prevalence and disease burden. Blended self-management interventions, which combine eHealth with face-to-face interventions, can help reduce the disease burden. OBJECTIVE: This systematic review and meta-analysis aims to examine the effectiveness of blended self-management interventions on health-related effectiveness and process outcomes for people with COPD or asthma. METHODS: PubMed, Web of Science, COCHRANE Library, Emcare, and Embase were searched in December 2018 and updated in November 2020. Study quality was assessed using the Cochrane risk of bias (ROB) 2 tool and the Grading of Recommendations, Assessment, Development, and Evaluation. RESULTS: A total of 15 COPD and 7 asthma randomized controlled trials were included in this study. The meta-analysis of COPD studies found that the blended intervention showed a small improvement in exercise capacity (standardized mean difference [SMD] 0.48; 95% CI 0.10-0.85) and a significant improvement in the quality of life (QoL; SMD 0.81; 95% CI 0.11-1.51). Blended intervention also reduced the admission rate (relative ratio [RR] 0.61; 95% CI 0.38-0.97). In the COPD systematic review, regarding the exacerbation frequency, both studies found that the intervention reduced exacerbation frequency (RR 0.38; 95% CI 0.26-0.56). A large effect was found on BMI (d=0.81; 95% CI 0.25-1.34); however, the effect was inconclusive because only 1 study was included. Regarding medication adherence, 2 of 3 studies found a moderate effect (d=0.73; 95% CI 0.50-0.96), and 1 study reported a mixed effect. Regarding self-management ability, 1 study reported a large effect (d=1.15; 95% CI 0.66-1.62), and no effect was reported in that study. No effect was found on other process outcomes. The meta-analysis of asthma studies found that blended intervention had a small improvement in lung function (SMD 0.40; 95% CI 0.18-0.62) and QoL (SMD 0.36; 95% CI 0.21-0.50) and a moderate improvement in asthma control (SMD 0.67; 95% CI 0.40-0.93). A large effect was found on BMI (d=1.42; 95% CI 0.28-2.42) and exercise capacity (d=1.50; 95% CI 0.35-2.50); however, 1 study was included per outcome. There was no effect on other outcomes. Furthermore, the majority of the 22 studies showed some concerns about the ROB, and the quality of evidence varied. CONCLUSIONS: In patients with COPD, the blended self-management interventions had mixed effects on health-related outcomes, with the strongest evidence found for exercise capacity, QoL, and admission rate. Furthermore, the review suggested that the interventions resulted in small effects on lung function and QoL and a moderate effect on asthma control in patients with asthma. There is some evidence for the effectiveness of blended self-management interventions for patients with COPD and asthma; however, more research is needed. TRIAL REGISTRATION: PROSPERO International Prospective Register of Systematic Reviews CRD42019119894; https://www.crd.york.ac.uk/prospero/display_record.php?RecordID=119894.	https://www.ncbi.nlm.nih.gov/pmc/articles/PMC804	Q2	PubMed	EXC_Scope
Telemedical Interventions for Chronic Obstructive Pulmonary Dis	2023	Background: Chronic obstructive pulmonary disease (COPD) is a growing epidemic, with a heavy associated economic burden. Education, physical activity, and pulmonary rehabilitation programs are important aspects of the management of COPD. These interventions are commonly delivered remotely as part of telemedicine interventions. Several systematic reviews and meta-analyses have been conducted to assess the effectiveness of these interventions. However, these reviews often have conflicting conclusions. Objective: We aim to conduct an umbrella review to critically appraise and summarize the available evidence on telemedicine interventions for the management of COPD. Methods: In this umbrella review, the MEDLINE, Embase, PsycINFO, and Cochrane databases were searched from inception to May 2022 for systematic reviews and meta-analyses relating to telemedicine interventions for the management of COPD. We compared odds ratios, measures of quality, and heterogeneity across different outcomes. Results: We identified 7 systematic reviews that met the inclusion criteria. Telemedicine interventions used in these reviews were teletreatment, telemonitoring, and telesupport. Telesupport interventions significantly reduced the number of inpatient days and quality of life. Telemonitoring interventions were associated with significant reductions in respiratory exacerbations and hospitalization rates. Teletreatment showed significant effectiveness in reducing respiratory exacerbations, hospitalization rate, compliance (acceptance and dropout rate), and physical activity. Among studies that used integrated telemedicine interventions, there was a significant improvement in physical activity. Conclusions: Telemedicine interventions showed noninferiority or superiority over the standard of care for the management of COPD. Telemedicine interventions should be considered as a supplement to usual methods of care for the outpatient management of COPD, with the aim of reducing the burden on health care systems.	https://pubmed.ncbi.nlm.nih.gov/36795479/	Q2	PubMed	EXC_PubType
Mobile heath applications for self-management in chronic lung di	2023	Integration of mobile health (mHealth) applications (apps) into chronic lung disease management is becoming increasingly popular. MHealth apps may support adoption of self-management behaviors to assist people in symptoms control and quality of life enhancement. However, mHealth apps' designs, features, and content are inconsistently reported, making it difficult to determine which were the effective components. Therefore, this review aims to summarize the characteristics and features of published mHealth apps for chronic lung diseases. A structured search strategy across five databases (CINAHL, Medline, Embase, Scopus and Cochrane) was performed. Randomized controlled trials investigating interactive mHealth apps in adults with chronic lung disease were included. Screening and full-text reviews were completed by three reviewers using Research Screener and Covidence. Data extraction followed the mHealth Index and Navigation Database (MIND) Evaluation Framework (https://mindapps. org/), a tool designed to help clinicians determine the best mHealth apps to address patients' needs. Over 90,000 articles were screened, with 16 papers included. Fifteen distinct apps were identified, 8 for chronic obstructive pulmonary disease (53%) and 7 for asthma (46%) self-management. Different resources informed app design approaches, accompanied with varying qualities and features across studies. Common reported features included symptom tracking, medication reminders, education, and clinical support. There was insufficient information to answer MIND questions regarding security and privacy, and only five apps had additional publications to support their clinical foundation. Current studies reported designs and features of self-management apps differently. These app design variations create challenges in determining their effectiveness and suitability for chronic lung disease self-management. Registration: PROSPERO (CRD42021260205).	https://pubmed.ncbi.nlm.nih.gov/37305790/	Q2	PubMed	EXC_Scope
Effectiveness of remote home monitoring for patients with Chron	2022	Background: Although remote home monitoring (RHM) has the capacity to prevent exacerbations in patients with chronic obstructive pulmonary disease (COPD), evidence regarding its effectiveness remains unclear. The objective of this study was to determine the effectiveness of RHM in patients with COPD. Methods: A systematic review of the scholarly literature published within the last 10 years was conducted using internationally recognized guidelines. Search strategies were applied to several electronic databases and clinical trial registries through March 2020 to identify studies comparing RHM to 'no remote home monitoring' (no RHM) or comparing RHM with provider's feedback to RHM without feedback. To critically appraise the included randomized studies, the Cochrane Collaboration risk of bias tool (ROB) was used. The quality of included non-randomized interventional and comparative observational studies was evaluated using the ACROBAT-NRSI tool from the Cochrane Collaboration. The quality of evidence relating to key outcomes was assessed using Grading of Recommendations, Assessment, Development and Evaluations (GRADE) on the following: health-related quality of life (HRQoL), patient experience and number of exacerbations, number of emergency room (ER) visits, COPD-related hospital admissions, and adherence as the proportion of patients who completed the study. Three independent reviewers assessed methodologic quality and reviewed the studies. Results: Seventeen randomized controlled trials (RCTs) and two comparative observational studies were included in the review. The primary finding of this systematic review is that a considerable amount of evidence relating to the efficacy/effectiveness of RHM exists, but its quality is low. Although RHM is safe, it does not appear to improve HRQoL (regardless of the type of RHM), lung function or self-efficacy, or to reduce depression, anxiety, or healthcare resource utilization. The inclusion of regular feedback from providers may reduce COPD-related hospital admissions. Though adherence RHM remains unclear, both patient and provider satisfaction were high with the intervention. Conclusions: Although a considerable amount of evidence to the effectiveness of RHM exists, due to heterogeneity of care settings and the low-quality evidence, they should be interpreted with caution.	https://pubmed.ncbi.nlm.nih.gov/35568904/	Q2	PubMed	EXC_Scope
Effects of Home-Based Training with Internet Telehealth Guidanc	2023	Objective: Chronic obstructive pulmonary disease (COPD) is one of the leading causes of death worldwide. Telehealth rehabilitation may offer new opportunities in patient therapy. This systematic review aimed to evaluate the effects of internet-mediated telerehabilitation and compare them with the outcomes of conventional pulmonary rehabilitation in COPD patients. Methods: Electronic databases PubMed, Prospero, Scopus, and Cochrane were searched for randomized controlled trials from January 2005 to December 2021. Two investigators reviewed studies for relevance and extracted study population, methods, and results data. Results: Ten studies were eligible for systematic review from the initial selection (n = 1492). There was considerable heterogeneity in telerehabilitation approaches. Functional exercise capacity and quality of life were assessed in all studies. None of the results were inferior to conventional care. High adherence and high levels of safety were observed. Conclusion: Telerehabilitation in COPD patients is a safe therapy approach that increases and maintains functional exercise capacity and quality of life, making it an equivalent option to conventional outpatient rehabilitation. However, there is currently a lack of a unified approach to the composition of therapy and the use of technology, which needs to be addressed in the future.	https://pubmed.ncbi.nlm.nih.gov/37876660/	Q2	PubMed	EXC_Scope

Types and Effects of Telenursing in Home Health Care: A Systen	2024	Purpose: This systematic review aimed to identify the types and effectiveness of telenursing in home health care (HHC) compared to conventional HHC. Methods: This study followed the Preferred Reporting Items for Systematic Review and Meta-Analysis 2020 guidelines. A random-effects meta-analysis was performed. The risk of bias was assessed using the Risk of Bias 2.0 tool. The quality of bias was evaluated using the Grading of Recommendations Assessment, Development, and Evaluation system. Results: A total of 17 studies published between 2003 and 2022 were included in the review. The majority of the targeted health conditions of the patients were chronic obstructive pulmonary disease or congestive heart failure (70%). We categorized telenursing interventions according to different technology (N = 4) and nursing (N = 7) types. Among the identified types of nursing, fundamental nursing to monitor patients' symptoms was the most representative. Telemonitoring was found to be the most common technology type, followed by synchronous technology using video or telephone. The telenursing outcomes, including health care utilization, physiological/psychological outcomes, and quality of life, varied. In the meta-analysis, participants who received telenursing reported fewer hospital admissions (standardized mean difference [SMD]: -0.18; confidence interval [95% CI]: -0.43 to -0.02) and emergency department visits (SMD: -0.28; 95% CI: -0.45 to -0.10). Conclusion: Telenursing in HHC tends to improve the quality of life and could result in many benefits, including a reduction in health care utilization and facilitating the management of chronic diseases. These results ultimately represent the potential effectiveness of telenursing in community health care settings.	https://pubmed.ncbi.nlm.nih.gov/37707998/	Q2	PubMed	EXC_Scope
Efficacy of self-management mobile applications for patients with	2022	Introduction: Apps can play a role in self-management of symptoms such as breathlessness. This systematic review aims to evaluate the clinical efficacy of breathlessness apps and assess the quality of those publicly available. Methods: The full protocol for this systematic review is available on PROSPERO (CRD42021246277). Studies published between January 2010 to 2022 were obtained from six academic databases and included if they evaluated apps that offered breathlessness education and personalised feedback. Outcomes of interest included change in breathlessness and quality of life. Additionally, quality assessment was conducted on apps addressing breathlessness publicly available from Google Play, iOS app store, and regulatory agency libraries. Results: A total of 2774 records were screened and 16 studies included in the systematic review. Disease groups addressed were chronic obstructive pulmonary disease, asthma, fibrotic lung disease, lung cancer, and heart failure. Use of mobile apps was found to result in clinical benefit especially when utilised for longer periods of time. For publicly available apps, a total of 776 apps were screened and 6 apps included. The mean quality rating using the Mobile App Rating Scale was 3.43 out of 5. Most apps were found to have good functionality and aesthetics; however, they were lacking in their quality of information and engagement. Conclusion: This review suggests that apps may provide clinical benefit to patients with breathlessness and can support clinical practice. However, few of those researched are available for public use and those accessible publicly were lacking in quality and compliance with m-Health best practices.	https://pubmed.ncbi.nlm.nih.gov/36037561/	Q2	PubMed	EXC_Scope
The Effectiveness of Exercise Interventions Supported by Telerel	2021	Objective: To evaluate the effectiveness of exercise interventions delivered via telerehabilitation (via videoconference) for recently hospitalized adult medical patients. Data sources: A search was undertaken across six databases for English language publications from inception to May 2020. Methods: Studies were selected if they included an exercise intervention for recently hospitalized adults, delivered by a physiotherapist via videoconference. Two reviewers independently screened 1,122 articles (21 full text screening) and assessed methodological quality using the Downs and Black Checklist. A narrative synthesis of the included studies was undertaken. Results: Three studies met eligibility criteria involving 201 participants with chronic heart failure or chronic obstructive pulmonary disease. Findings demonstrated limited evidence supporting the effectiveness of exercise delivered via telerehabilitation in improving physical function and patient reported quality of life outcomes in recently hospitalized medical patients. Telerehabilitation in this setting was also associated with high attendance rates and patient satisfaction. Conclusions: Findings provide preliminary support for the benefits of exercise interventions delivered via telerehabilitation for recently hospitalized medical patients. Results do need to be interpreted with caution as further high-quality studies specific to this method of exercise intervention delivery are needed.	https://pubmed.ncbi.nlm.nih.gov/35646230/	Q2	PubMed	EXC_Scope
A realist review of factors critical for the implementation of eHeal	2025	Background: In Canada, chronic disease is responsible for 88% of deaths and $120 billion in cost each year. With 44% of Canadian adults living with at least one chronic condition, only 66% receive necessary care. Ehealth interventions are instrumental in chronic disease management (CDM), especially since the pandemic, as they provide accessible, cost-effective solutions for self-management. Despite its promise and accelerated use, its implementation remains challenging. This paper reports on a realist review of critical factors for the implementation of eHealth interventions relevant to conditions such as heart failure, chronic obstructive pulmonary disease, chronic kidney disease, and diabetes. The findings are presented in terms of context, mechanisms, and outcomes. Methods: A realist review of the primary literature was conducted by searching five databases: Medline, Embase, Cochrane, CINALH and PsycInfo. The initial search was run for a date spanning from the databases' inception to September 2018 and subsequently updated to dates spanning from October 2018 to May 2022. A systematic and iterative approach to data extraction was used. Thematic analysis was used to identify context-mechanism-outcome (CMO) configurations. Results: Among the 13,209 citations retrieved, 64 articles were included. This paper reports the top ten configurations found to facilitate or hinder eHealth implementation. Key themes related to context, such as team-based care, and action, including program use, perceived usefulness and motivation, are reported. Conclusions: This study explores the role of context, mechanisms, and outcomes in ehealth implementation, highlighting the nonlinear relationships between these factors. Future implications include empirical testing CMOs as middle-range theories in real-world settings to determine causality	https://pubmed.ncbi.nlm.nih.gov/40176012/	Q2	PubMed	EXC_Scope
Comment on 'Comparative Effectiveness of eHealth Intervention:	2025	-	https://pubmed.ncbi.nlm.nih.gov/39831574/	Q2	PubMed	EXC_Scope
How to Enhance the Diagnosis of Early Stages of Chronic Obstru	2024	Highlights: What are the main findings? Portable spirometers are only slightly less efficient in diagnosing COPD than traditional spirometers The highest COPD prevalence was demonstrated when well-selected high-risk patients were tested What is the implication of the main finding? Portable spirometers are useful in the early diagnosis of COPD Portable spirometers enable bedside COPD diagnosis COPD is the third leading cause of death worldwide. Its diagnosis can be made with spirometry, which is underused due to its limited accessibility. Portable spirometry holds promise for enhancing the efficacy of COPD diagnoses. The study aimed to estimate COPD prevalence diagnosed with a portable spirometer in high-risk patients and compare it with COPD prevalence based on data from conventional, on-site spirometry. We also evaluated the strategy of a proactive approach to identify COPD in high-risk individuals. We conducted a systematic review of original studies on COPD targeted screening and diagnosis with portable and conventional spirometers selected from 8496 publications initially found in three databases: Cochrane, PubMed, and Embase. The inclusion criteria were met by 28 studies. COPD prevalence evaluated with the use of portable spirometers reached 20.27% and was lower compared to that estimated with the use of conventional spirometers (24.67%). In 11 included studies, postbronchodilator tests were performed with portable spirometers, which enabled a bedside COPD diagnosis. Portable spirometers can be successfully used in COPD targeted screening and diagnosis and thus enhance the detection of COPD at early stages. © 2024 by the authors.	https://www.scopus.com/inward/record.uri?eid=2-s	Q2	Scopus	EXC_Scope
Tobacco and COPD: presenting the World Health Organization (\	2024	The WHO recently published a Tobacco Knowledge Summary (TKS) synthesizing current evidence on tobacco and COPD, aiming to raise awareness among a broad audience of health care professionals. Furthermore, it can be used as an advocacy tool in the fight for tobacco control and prevention of tobacco-related disease. This article builds on the evidence presented in the TKS, with a greater level of detail intended for a lung-specialist audience. Pulmonologists have a vital role to play in advocating for the health of their patients and the wider population by sharing five key messages: (1) Smoking is the leading cause of COPD in high-income countries, contributing to approximately 70% of cases. Quitting tobacco is an essential step toward better lung health. (2) People with COPD face a significantly higher risk of developing lung cancer. Smoking cessation is a powerful measure to reduce cancer risk. (3) Cardiovascular disease, lung cancer and type-2 diabetes are common comorbidities in people with COPD. Quitting smoking not only improves COPD management, but also reduces the risk of developing these coexisting conditions. (4) Tobacco smoke also significantly impacts children's lung growth and development, increasing the risk of respiratory infections, asthma and up to ten other conditions, and COPD later in life. Governments should implement effective tobacco control measures to protect vulnerable populations. (5) The tobacco industry's aggressive strategies in the marketing of nicotine delivery systems and all tobacco products specifically target children, adolescents, and young adults. Protecting our youth from these harmful tactics is a top priority. © The Author(s) 2024.	https://www.scopus.com/inward/record.uri?eid=2-s	Q2	Scopus	EXC_Scope

Alternative Modes of Delivery in Pulmonary Rehabilitation A CRI	2024	Purpose: This review presents an overview of the safety and efficacy of alternative modes of pulmonary rehabilitation (PR) in people with chronic obstructive pulmonary disease (COPD). Review Methods: We identified recently published systematic reviews, meta-analyses, and guidelines, as well as relevant studies, exploring the safety and effectiveness of community-based PR, home-based PR, telerehabilitation, and web-based rehabilitation in people with COPD. A narrative summary of the main findings is presented. Summary: Although evidence suggests that community-based PR, home-based PR, telerehabilitation, and web-based rehabilitation are effective alternatives to center-based PR, it requires a careful interpretation as several of these programs do not comply with PR definition and have been compared with center-based PR programs that do not reach the minimal clinically important differences. Moreover, there is a huge heterogeneity among programs, and the confidence and quality of the evidence is mostly low. Hence, these novel modes of PR and center-based PR are not interchangeable. Instead, these are alternative modes aiming to increase access to PR. Questions remain regarding the most efficient way of implementing each PR mode, level of access, reimbursement policies, and data privacy in the use of technology. Standard protocols on how to set up each alternative PR mode need to be developed. Future research needs to explore how to use the treatable traits approach in combination with individual preferences and needs, program availability, safety, social support network, digital literacy, and health system context to identify the optimal PR program for each patient. Copyright © 2024 Wolters Kluwer Health, Inc. All rights reserved.	https://www.scopus.com/inward/record.uri?eid=2-s	Q2	Scopus	EXC_PubType
Filling the gaps in the evaluation and selection of mobile health te	2024	Introduction: Mobile health (mHealth) technology in respiratory medicine is a fast-growing and promising digital technology that is popular among patients and healthcare providers (HCPs). They provide reminders and step-by-step instructions for the correct inhalation technique, monitor patients' adherence to treatment, and facilitate communication between patients and HCPs. Areas Covered: While numerous mHealth apps have been developed over the years, most applications do not have supporting evidence. Selecting the best mHealth app in respiratory medicine is challenging due to limited studies carrying out mHealth app selection. Although mHealth technologies play an important part in the future of respiratory medicine, there is no single guide on the evaluation and selection of mHealth technologies for patients with pulmonary diseases. This paper aims to provide an overview of mHealth technologies, particularly emphasizing digital inhalers and standalone applications used in asthma. Additionally, it offers insights into the evaluation, selection, and pertinent considerations surrounding mHealth applications in respiratory medicine. Expert Opinion: Evaluating mHealth apps will take time, resources, and collaboration between stakeholders such as governmental regulatory bodies, subject-matter experts, and industry representatives. Filling the gaps in the evaluation and selection of the mHealth app will improve clinical decision-making, personalized treatments, self-management and disease monitoring in respiratory medicine. © 2024 Informa UK Limited, trading as Taylor & Francis Group.	https://www.scopus.com/inward/record.uri?eid=2-s	Q2	Scopus	EXC_Scope
Evidence Construction of Chuankezhi Injection Against Chronic C	2024	Objective: Chronic obstructive pulmonary disease (COPD) is a chronic respiratory disease with high prevalence, morbidity, and mortality. Chuankezhi (CKZ) injection, a Chinese patent medicine, has been commonly used for treating COPD. This study evaluated the clinical efficacy of CKZ injections in COPD patients and explored potential underlying mechanisms by integrating meta-analysis and network pharmacology. Research Methods: Randomized controlled trials (RCTs) were search in database by Web of Science, Cochrane Library and PubMed as of November 2022 for literature collection, and the Review Manager 5.4 was used to analyze the data. Through the network pharmacology method, the chemical components and their targets, as well as the disease targets were further analyzed. Results: A total of 15 RCTs including 1212 patients were included. The results of meta-analysis showed that CKZ injection can significantly improve the clinical effective rate (RR = 1.25, 95% CI: 1.14 to 1.36), and the clinical advantage was that it can significantly reduced acute exacerbation rate (RR = 0.29, 95% CI: 0.12 to 0.70) and COPD assessment test (CAT) scores (MD =−4.62, 95% CI:-8.966 to-0.28). A total of 31 chemical compounds and 178 potential targets for CKZ injection were obtained from the online databases. Molecular docking revealed that most key components and targets could form stable structure. Conclusion: This systematic review with meta-analysis and network pharmacology demonstrates that CKZ could effectively improve the clinical efficacy and safety in the treatment of COPD. Such efficacy may be related to an anti-inflammatory effect and immunoregulation of CKZ via multiple components, multiple targets and multiple pathways. © 2024 Wei et al.	https://www.scopus.com/inward/record.uri?eid=2-s	Q2	Scopus	EXC_Scope
Web-based pulmonary telehabilitation: a systematic review	2024	Web-based pulmonary telerehabilitation (WBPTR) can serve as a valuable tool when access to conventional care is limited. This review assesses a series of studies that explore pulmonary telerehabilitation programmes delivered via web-based platforms. The studies involved participants with moderate to severe chronic obstructive pulmonary disease (COPD). Of the 3190 participants, 1697 engaged in WBPTR platforms, while the remaining 1493 comprised the control groups. Sixteen studies were included in the meta-analysis. Web-based pulmonary telerehabilitation led to an increase in daily step count (MD 446.66, 95% CI 96.47 to 796.86), though this did not meet the minimum clinically important difference. Additionally, WBPTR did not yield significant improvements in the six-minute walking test (MD 5.01, 95% CI − 5.19 to 15.21), health-related quality of life as measured by the St. George's Respiratory Questionnaire (MD − 0.15, 95% CI − 2.24 to 1.95), or the Chronic Respiratory Disease Questionnaire (MD 0.17, 95% CI − 0.13 to 0.46). Moreover, there was no significant improvement in dyspnoea-related health status, as assessed by the Chronic Respiratory Disease Questionnaire (MD − 0.01, 95% CI − 0.29 to 0.27) or the modified Medical Research Council Dyspnoea Scale (MD − 0.14, 95% CI − 0.43 to 0.14). Based on these findings, this review concludes that WBPTR does not offer substantial advantages over traditional care. While slight improvements in exercise performance were observed, no meaningful enhancements were noted in dyspnoea or quality of life metrics. Overall, WBPTR remains a complementary and accessible option for managing and monitoring COPD patients. However, further research and innovation are required to improve its efficacy and adapt it to various clinical environments. © The Author(s) 2024.	https://www.scopus.com/inward/record.uri?eid=2-s	Q2	Scopus	EXC_Scope
Recent advances in understanding the role of antidepressants to	2025	Purpose of review Breathlessness is a prevalent and distressing symptom in palliative and supportive care, with limited licensed pharmacological options once disease-directed therapies are no longer effective. Antidepressants have been proposed as a potential treatment, even in the absence of comorbid mood disorders, due to their modulation of neural circuits and serotonin pathways involved in breathlessness perception. Despite their off-label use in clinical practice for managing refractory or chronic breathlessness, robust evidence supporting their efficacy is needed. This review critically evaluates the latest evidence on their potential benefits and safety in breathlessness management. Recent findingsBreathlessness is influenced by at least three interrelated axes: lung-brain, behavioural-functional, and psycho-social-spiritual. These mechanisms operate across diseases, making them relevant in palliative and supportive care. Despite promise from early case reports and small trials, two recent large, randomised studies of mirtazapine and sertraline found no benefit in alleviating breathlessness or improving other outcomes. The mirtazapine trial also reported more adverse events than placebo. Earlier trials were small with design limitations, reducing reliability. A 2016 trial of sertraline found benefits for depression in stable COPD. Recent concerns over increased morbidity associated with antidepressant use in respiratory disease highlight the need for early detection of people at risk of worsening breathlessness or depression and a holistic, individualised approach. Summary Current evidence does not support antidepressants for breathlessness in respiratory disease. Non-pharmacological approaches should be first line, given their proven benefits and low risk. Off-label medicine use requires caution and should ideally be offered within a trial or evaluation. Given the complex nature of breathlessness, future research should focus on innovating and then testing treatments and therapies in well-designed trials with appropriate outcome measures and reporting of adverse events, health care use and informal carer effects. Copyright © 2025 The Author (s). Published by Wolters Kluwer Health, Inc.	https://www.scopus.com/inward/record.uri?eid=2-s	Q2	Scopus	EXC_Scope

The Experiences and Perceptions of Telehealth in Patients Living	2025	Aim: To systematically identify, appraise and synthesise qualitative research evidence which examined the impact of telehealth on the experiences and perceptions of patients living with advanced chronic obstructive pulmonary disease, to inform the development of patient-centred telehealth. Design: Qualitative evidence synthesis. Database Searches: CINAHL, Cochrane, Embase, PUBMED, MEDLINE, EThOS, Web of Science, PsycINFO, Lenus, DART, RIAN and ProQuest were searched for primary qualitative studies undertaken between 2008 and 2023. Methods: A thematic synthesis of studies was undertaken to identify descriptive themes relating to patient views. Methodological quality was assessed using the Critical Appraisal Skills Programme framework, and confidence in review findings was assessed using the GRADE-CERQual approach. Findings: Nine studies met the inclusion criteria and were included in the final synthesis. Four analytical themes were generated (1) telehealth as a facilitator of independence, (2) the influence of patient and healthcare provider relationship on successful engagement with telehealth, (3) usability of telehealth to patients living with advanced chronic obstructive pulmonary disease and (4) trusting virtual health services and facilitating confidence in the patient/service user. Five descriptive themes emerged: (i) individualised telehealth chronic obstructive pulmonary disease care (ownership and control), (ii) managing chronic obstructive pulmonary disease exacerbations, (iii) being heard and feeling understood, (iv) telehealth as an education aid and (v) aging and virtual technology. Conclusion: Understanding the experiences of patients with chronic obstructive pulmonary disease and their engagement with telehealth is a necessary determinant of how best to utilise telehealth in this population and may serve to inform policymakers to further develop and implement telehealth into practice. Future research on patients and healthcare professionals' views on telehealth use in the palliative stage of this illness may also be valuable. Impact: Findings add value by providing healthcare providers with additional evidence to improve understanding of both telehealth complexity and human experiences and perceptions. It is anticipated that a deeper understanding of chronic obstructive pulmonary disease patients' experiences and perceptions will inform the development of strategies to maximise and enhance the application of patient-centred telehealth within the context of coping and living with a debilitating condition. Patient or Public Contribution: No patient or public contribution was utilised in this study. © 2024 The Author(s). Journal of Advanced Nursing published by John Wiley & Sons Ltd.	https://www.scopus.com/inward/record.uri?eid=2-s	Q2	Scopus	EXC_Scope
Chronic Obstructive Pulmonary Disease and Type 2 Diabetes Me	2025	Chronic obstructive pulmonary disease (COPD) and type 2 diabetes mellitus (T2DM) are highly prevalent chronic conditions, frequently coexisting due to their shared pathophysiological mechanisms and risk factors. Epidemiological studies estimate that up to 30% of COPD patients have comorbid T2DM, contributing to worsened disease progression, more hospitalizations, and higher mortality rates. Systemic inflammation in COPD contributes to insulin resistance by increasing pro-inflammatory cytokines (TNF-α, IL-6, and CRP), which impair glucose metabolism and beta-cell function. Conversely, hyperglycemia in T2DM exacerbates oxidative stress, leading to endothelial dysfunction, reduced lung function, and impaired pulmonary repair mechanisms. A comprehensive narrative review was conducted to evaluate the interplay between COPD and T2DM, examining shared pathophysiological mechanisms, clinical consequences, and management strategies. The co-occurrence of COPD and T2DM accelerates disease development, elevates hospitalization rates, and deteriorates overall prognosis. Pharmacological interactions complicate illness treatment, requiring a multidisciplinary therapy strategy. Recent data underscore the need to integrate palliative care, facilitate shared decision-making, and provide psychological support to enhance patient outcomes. Efficient therapy of COPD-T2DM comorbidity necessitates a customized, interdisciplinary strategy that targets both respiratory and metabolic health. Preliminary prognostic dialogues, palliative care, and holistic lifestyle modifications can improve patient quality of life and clinical results. © 2025 by the authors.	https://www.scopus.com/inward/record.uri?eid=2-s	Q2	Scopus	EXC_Scope
Telemonitoring in Non-invasive Ventilation	2024	[No abstract available]	https://www.scopus.com/inward/record.uri?eid=2-s	Q2	Scopus	EXC_PeerReview
Exercise-based interventions targeting balance and falls in peopl	2024	Introduction This review quantifies the mean treatment effect of exercise-based interventions on balance and falls risk in people with COPD. Methods A structured search strategy (2000–2023) was applied to eight databases to identify studies evaluating the impact of exercise-based interventions (⩾14 days in duration) on balance or falls in people with COPD. Pooled mean treatment effects (95% confidence intervals (CIs), 95% prediction intervals (PIs)) were calculated for outcomes reported in five or more studies. Inter-individual response variance and the promise of behaviour change techniques (BCTs) were explored. Results 34 studies (n=1712) were included. There were greater improvements in balance post intervention compared to controls for the Berg Balance Scale (BBS) (mean 2.51, 95% CI 0.22–4.80, 95% PI −4.60– 9.63), Timed Up and Go (TUG) test (mean −1.12 s, 95% CI −1.69– −0.55 s, 95% PI −2. 78–0.54 s), Single-Leg Stance (SLS) test (mean 3.25 s, 95% CI 2.72–3.77 s, 95% PI 2.64–3.86 s) and Activities-specific Balance Confidence (ABC) scale (mean 8.50%, 95% CI 2.41–14.58%, 95% PI −8.92–25.92%). Effect on falls remains unknown. Treatment effects were larger in male versus mixed-sex groups for the ABC scale and SLS test, and in balance training versus other exercise-based interventions for the BBS and TUG test. Falls history was not associated with changes in balance. Meta-analysis of individual response variance was not possible and study-level results were inconclusive. Eleven promising BCTs were identified (promise ratio ⩾2). Conclusion Evidence for the effect of exercise-based interventions eliciting clinically important improvements in balance for people with COPD is weak, but targeted balance training produces the greatest benefits. Future exercise interventions may benefit from inclusion of the identified promising BCTs. © The authors 2024.	https://www.scopus.com/inward/record.uri?eid=2-s	Q2	Scopus	EXC_Scope
Changes in physical activity, sedentary behaviour and sleep follo	2024	Background: The variety of innovations to traditional centre-based pulmonary rehabilitation (CBPR), including different modes of delivery and adjuncts, are likely to lead to differential responses in physical activity, sedentary behaviour and sleep. Objectives: To examine the relative effectiveness of different pulmonary rehabilitation-based interventions on physical activity, sedentary behaviour and sleep. Methods: Randomised trials in chronic respiratory disease involving pulmonary rehabilitation-based interventions were systematically searched for. Network meta-analyses compared interventions for changes in physical activity, sedentary behaviour and sleep in COPD. Results: 46 studies were included, and analyses were performed on most common outcomes: steps per day (k=24), time spent in moderate-to-vigorous physical activity (MVPA; k=12) and sedentary time (k=8). There were insufficient data on sleep outcomes (k=3). CBPR resulted in greater steps per day and MVPA and reduced sedentary time compared to usual care. CBPR+physical activity promotion resulted in greater increases in steps per day compared to both usual care and CBPR, with greater increases in MVPA and reductions in sedentary time compared to usual care, but not CBPR. Home-based pulmonary rehabilitation resulted in greater increases in steps per day and decreases in sedentary time compared to usual care. Compared to usual care, CBPR+physical activity promotion was the only intervention where the lower 95% confidence interval for steps per day surpassed the minimal important difference. No pulmonary rehabilitation-related intervention resulted in greater increases in MVPA or reductions in sedentary time compared to CBPR. Conclusion: The addition of physical activity promotion to pulmonary rehabilitation improves volume of physical activity, but not intensity, compared to CBPR. High risk of bias and low certainty of evidence suggests that these results should be viewed with caution. © The authors 2024.	https://www.scopus.com/inward/record.uri?eid=2-s	Q2	Scopus	EXC_Scope
Exploring cardiopulmonary rehabilitation in the middle east and N	2024	Background and Objectives: Cardiopulmonary Rehabilitation (CR) is crucial for managing conditions like congestive heart failure (CHF), chronic obstructive pulmonary disease (COPD), and post-COVID-19 complications. This review examines CR practices in the Middle East and North Africa (MENA) region, exploring challenges, disparities, and emerging trends. Methods: A comprehensive literature search was conducted in PubMed, Scopus, and Web of Science to identify studies published between date of inception and April 24th, 2024, focusing on CR programs, outcomes, challenges, and strategies specific to the MENA region. Data extraction included study design, population characteristics, CR interventions, and key findings. Results: CR programs in the MENA region vary widely in scope and execution. While efforts are underway to integrate CR services into national healthcare policies, significant challenges persist, including limited infrastructure, shortages of trained professionals, and cultural barriers. Emerging trends include the use of telehealth and digital monitoring tools to expand access to CR services and policy reforms aimed at improving service delivery and patient access. Conclusion: CR plays a crucial role in improving the quality of life and health outcomes for cardiopulmonary patients, including those in the MENA region. However, significant challenges hinder the widespread adoption and effectiveness of CR programs. Addressing these challenges requires efforts to increase public education, reduce costs, expand funding, and enhance interprofessional collaboration. Future research should assess virtual rehabilitation, cultural adjustments, and long-term outcomes to tailor interventions to MENA's needs, ultimately enhancing CR accessibility and patient outcomes. © 2024	https://www.scopus.com/inward/record.uri?eid=2-s	Q2	Scopus	EXC_PubType
Industry 4.0-Compliant Occupational Chronic Obstructive Pulmoı	2024	Chronic obstructive pulmonary disease (COPD) is among prevalent occupational diseases, causing early retirement and disabilities. This paper looks into occupational-related COPD prevention and intervention in the workplace for Industry 4.0-compliant occupation health and safety management. The economic burden and other severe problems caused by COPD are introduced. Subsequently, seminal research in relevant areas is reviewed. The prospects and challenges are introduced and discussed based on critical management approaches. An initial design of an Industry 4.0-compliant occupational COPD prevention system is presented at the end. © 2024 by the authors.	https://www.scopus.com/inward/record.uri?eid=2-s	Q2	Scopus	EXC_Scope

The effect of telemedicine employing telemonitoring instruments	2024	Background: Hospital readmissions pose a challenge for modern healthcare systems. Our aim was to assess the efficacy of telemedicine incorporating telemonitoring of patients' vital signs in decreasing readmissions with a focus on a specific patient population particularly prone to rehospitalization: patients with heart failure (HF) and/or chronic obstructive pulmonary disease (COPD) through a comparative effectiveness systematic review. Methods: Three major electronic databases, including PubMed, Scopus, and ProQuest's ABI/INFORM, were searched for English-language articles published between 2012 and 2023. The studies included in the review employed telemedicine incorporating telemonitoring technologies and quantified the effect on hospital readmissions in the HF and/or COPD populations. Results: Thirty scientific articles referencing twenty-nine clinical studies were identified (total of 4,326 patients) and were assessed for risk of bias using the RoB2 (nine moderate risk, six serious risk) and ROBINS-I tools (two moderate risk, two serious risk), and the Newcastle-Ottawa Scale (three good-quality, four fair-quality, two poor-quality). Regarding the primary outcome of our study which was readmissions: the readmission-related outcome most studied was all-cause readmissions followed by HF and acute exacerbation of COPD readmissions. Fourteen studies suggested that telemedicine using telemonitoring decreases the readmission-related burden, while most of the remaining studies suggested that it had a neutral effect on hospital readmissions. Examination of prospective studies focusing on all-cause readmission resulted in the observation of a clearer association in the reduction of all-cause readmissions in patients with COPD compared to patients with HF (100% vs. 8%). Conclusions: This systematic review suggests that current telemedicine interventions employing telemonitoring instruments can decrease the readmission rates of patients with COPD, but most likely do not impact the readmission-related burden of the HF population. Implementation of novel telemonitoring technologies and conduct of more high-quality studies as well as studies of populations with ≥2 chronic disease are necessary to draw definitive conclusions. Systematic Review Registration: This study is registered at the International Platform of Registered Systematic Review and Meta-analysis Protocols (INPLASY), identifier (INPLASY202460097). 2024 Stergiopoulos, Elayadi, Chen and Galiatsatos.	https://www.scopus.com/inward/record.uri?eid=2-s	Q2	Scopus	EXC_Scope
Exploring the association between asthma and chronic comorbid	2024	Asthma remains a significant global health challenge. While both the incidence and mortality rates have shown a decline, older individuals with asthma exhibit not just more severe symptoms but also demonstrate an elevated mortality rate. This phenomenon could be attributed to the presence of chronic comorbidities that exert an influence on clinical outcomes among adult patients with asthma. This review aims to present various aspects of asthma comprehensively, including the prevalence, incidence, mortality rates, and causes of death in adult patients with asthma. Additionally, this review delves into the impact of chronic comorbidities that contribute to the morbidity and mortality of patients with asthma on a global scale, encompassing conditions such as chronic kidney disease, diabetes mellitus, lung cancer, obesity, and cardiovascular disease, concerning asthma. Furthermore, the manuscript reviews the distinctions between asthma and asthma chronic obstructive pulmonary disease overlap and adds perspective on asthma as an occupational lung disease. Thus, this review aims to enhance clinicians' awareness of the significance of chronic comorbidities in the management of patients with asthma. It seeks to provide insights that contribute to a more comprehensive approach to managing patients with asthma who also have comorbid conditions. Copyright © 2024 Listyoko, Okazaki, Harada, Inui and Yamasaki.	https://www.scopus.com/inward/record.uri?eid=2-s	Q2	Scopus	EXC_Scope
Home-based management on hospital re-admission rates in COI	2024	Aim: To determine the impact of home-based management on hospital re-admission rates in patients with chronic obstructive pulmonary disease (COPD). Design: Systematic review methodology was utilized, combining meta-analysis, where appropriate, or a narrative analysis of the data from included studies. Data Sources: Electronic databases CINAHL, MEDLINE, PubMed, Embase and SAGE journals for primary papers, 2015 to 2021, were searched between December 2020 and March 2021, followed by hand-searching key journals, and reference lists of retrieved papers. Methods: The review followed the guidance of PRISMA. Data were extracted using a predesigned data extraction tool. Quality appraisal was undertaken using RevMan 'risk of bias' tool. Meta-analysis was undertaken using RevMan software. Results: This review integrates evidence from eight studies, five Random Control Trials, two observational studies and one retrospective study. The studies span three continents, Asia, Europe and North America, and include 3604 participants with COPD. Home-based management in patients with COPD resulted in a statistically significant reduction in rates of hospital readmission. For the outcomes, length of stay and mortality, while slightly in favour of home-based management, the results were not statistically significant. Conclusion: Given the burden of COPD on healthcare systems, and crucially on individuals, this review identified a reduction in hospital re-admission rate, a clinically important outcome. Impact: This study focused on the impact on hospital re-admission rates among the COPD patient cohort when home-based management was involved. A statistically significant reduction in rates of re-admission to the hospital was identified. This is positive for the patient, in terms of hospital avoidance, and reduces the burden on hospital systems. Further research is needed to determine the impact on cost-effectiveness and to quantify the most ideal type of care package that would be recommended for home-based management. © 2024 The Authors. Journal of Advanced Nursing published by John Wiley & Sons Ltd.	https://www.scopus.com/inward/record.uri?eid=2-s	Q2	Scopus	EXC_Scope
A narrative review of proactive palliative care models for people v	2025	Chronic obstructive pulmonary disease (COPD) refers to a group of lung diseases that are distinct in underlying aetiology but share a common disease course of persistent and progressive airflow restriction. People living with COPD, as well as the people who care for them, frequently have severe and unmet physical and psychosocial needs, including breathlessness, fatigue, cough, anxiety and depression. Early proactive palliative care is well placed to address these needs, yet it is frequently under-utilised in this group. This narrative review aimed to identify core components of palliative care and examine how existing models of care are implemented to better understand which models can best serve the needs of people with COPD. Symptom palliation, advance care planning, and support for caregivers emerged as the common components underpinning both generalist and specialist models of palliative care. Models of proactive palliative care were diverse in terms of where and how care was delivered as well as which health professionals were involved. Five key models of palliative care were identified: (1) multi-disciplinary integrated services, (2) nurse-led care, (3) hospice and residential aged care, (4) home-based care, and (5) telemonitoring and telehealth. Each model describes a diverse set of interventions and many of these share common elements, including the normalisation of palliative principles within routine care and the provision of diverse delivery settings to accommodate individual preferences and needs. Successful palliative care models must be practical, accessible and innovative to respond to individuals' complex and evolving needs, foster multi-disciplinary collaboration and input and optimally utilise local healthcare resources. © The Author(s), 2025.	https://www.scopus.com/inward/record.uri?eid=2-s	Q2	Scopus	EXC_PubType
Is Disease Stability an Attainable Chronic Obstructive Pulmonary	2025	Chronic obstructive pulmonary disease (COPD) is a heterogeneous lung condition characterized by progressive airflow obstruction. Despite advancements in diagnosis and treatment, the disease burden remains high; although clinical trials have shown improvements in outcomes such as exacerbations, quality of life, and lung function, improvement may not be attainable for many patients. For patients who do experience improvement, it is challenging to set management goals given the progressive nature of COPD. We therefore propose disease stability as an appropriate and attainable treatment goal. Other disease areas have developed definitions of no disease activity or remission, which provide relevant information for defining and achieving stability for patients with COPD. Disease stability builds on related concepts already defined in COPD, such as clinical control and clinically important deterioration. Current components that could form part of a disease stability definition include exacerbations, health status (including quality of life and symptoms), and lung function. Considerations should be given to intervals over which stability is defined and assessed, appropriate thresholds, and defining a composite. Ensuring a holistic approach, objective measurements, and harmonious, clear communication between patients and physicians can further support establishing disease stability. Here we propose a preliminary definition of disease stability, informed by existing research in COPD. Further research will be needed to validate the framework for use in clinical and research settings. Exploring disease stability as a goal, however, is an opportunity to develop and validate an attainable treatment target to advance the standard of care for patients with COPD. Copyright © 2025 by the American Thoracic Society.	https://www.scopus.com/inward/record.uri?eid=2-s	Q2	Scopus	EXC_Scope
Interventions to Reduce Lung Cancer and COPD-Related Stigma	2024	Background: Many individuals with lung cancer and chronic obstructive pulmonary disease (COPD) experience high levels of stigma, which is associated with psychological distress and delayed help-seeking. Purpose: To identify interventions aimed at reducing the stigma of lung cancer or COPD and to synthesize evidence on their efficacy. Methods: A systematic review was conducted by searching PubMed, Scopus, PsycINFO, and CINAHL for relevant records until March 1, 2024. Studies were eligible if they described an intervention designed to reduce internalized or external stigma associated with COPD or lung cancer and excluded if they did not report empirical findings. Results: We identified 476 papers, 11 of which were eligible for inclusion. Interventions included educational materials, guided behavior change programs, and psychotherapeutic approaches. Interventions targeted people diagnosed with, or at high risk of developing COPD or lung cancer or clinical staff. No interventions that aimed to reduce stigma associated with lung cancer or COPD in the general community were identified. Most interventions yielded a statistically significant reduction in at least one measure of stigma or a decrease in qualitatively reported stigma. Conclusions: The emerging literature on interventions to reduce stigma associated with lung cancer and COPD suggests that such interventions can reduce internalized stigma, but larger evaluations using randomized controlled trials are needed. Most studies were in the pilot stage and required further evaluation. Research is needed on campaigns and interventions to reduce stigma at the societal level to reduce exposure to external stigma amongst those with COPD and lung cancer. © 2024 The Author(s).	https://www.scopus.com/inward/record.uri?eid=2-s	Q2	Scopus	EXC_Scope

Comorbid health outcomes in patients with schizophrenia: an um	2025	There is no comprehensive umbrella review exploring the connection between schizophrenia and various health outcomes. Therefore, we aimed to systematically review existing meta-analyses about schizophrenia-associated comorbid health outcomes and validate the evidence levels. We performed an umbrella review of meta-analyses of observational studies to explore comorbid health outcomes in individuals with schizophrenia. Searches were conducted across PubMed/MEDLINE, EMBASE, ClinicalKey, and Google Scholar up to September 5, 2023, targeting meta-analyses of observational studies related to comorbid health outcomes in individuals with schizophrenia. We applied AMSTAR2 for data extraction and quality assessment, adhering to PRISMA guidelines. Evidence credibility was evaluated and categorized by evidence quality. Our protocol was registered with PROSPERO (CRD42024498833). Risk and protective factors were analyzed and presented through equivalent odds ratios (eRR). In this umbrella review, we analyzed 9 meta-analyses, including 88 original articles, covering 21 comorbid health outcomes with over 66 million participants across 19 countries. Patients with schizophrenia showed significant associations with multiple health outcomes, including asthma (eRR, 1.71 [95% CI, 1.05–2.78], class and quality of evidence [CE] = non-significant), chronic obstructive pulmonary disease (1.73 [1.25–2.37], CE = weak), pneumonia (2.63 [1.11–6.23], CE = weak), breast cancer of female patients (1.31 [1.04–1.65], CE = weak), cardiovascular disease (1.53 [1.12–2.11], CE = weak), stroke (1.71 [1.30–2.25], CE = weak), congestive heart failure (1.81 [1.21–2.69], CE = weak), sexual dysfunction (2.30 [1.75–3.04], CE = weak), fracture (1.63 [1.10–2.40], CE = weak), dementia (2.29 [1.19–4.39], CE = weak), and psoriasis (1.83 [1.18–2.83] CE = weak). Our study underscores the imperative for an integrated treatment approach to schizophrenia, highlighting its broad impact across respiratory, cardiovascular, sexual, neurological, and dermatological health domains. Given the predominantly non-significant to weak evidence levels, further studies are needed to reinforce our understanding. (Figure presented.) © The Author(s), under exclusive licence to Springer Nature Limited 2024.	https://www.scopus.com/inward/record.uri?eid=2-s	Q2	Scopus	EXC_PubType
Race Adjustment of Pulmonary Function Tests in the Diagnosis a	2024	Aim: Increasing evidence suggests that the inclusion of self-identified race in clinical decision algorithms may perpetuate long-standing inequities. Until recently, most pulmonary function tests utilized separate reference equations that are race/ethnicity based. Purpose: We assess the magnitude and scope of the available literature on the negative impact of race-based pulmonary function prediction equations on relevant outcomes in African Americans with COPD. Methods: We performed a scoping review utilizing an English language search on PubMed/Medline, Embase, Scopus, and Web of Science in September 2022 and updated it in December 2023. We searched for publications regarding the effect of race-specific vs race-neutral, race-free, or race-reversed lung function testing algorithms on the diagnosis of COPD and COPD-related physiologic and functional measures. Joanna Briggs Institute (JBI) guidelines were utilized for this scoping review. Eligibility criteria: The search was restricted to adults with COPD. We excluded publications on other lung disorders, non-English language publications, or studies that did not include African Americans. The search identified publications. Ultimately, six peer-reviewed publications and four conference abstracts were selected for this review. Results: Removal of race from lung function prediction equations often had opposite effects in African Americans and Whites, specifically regarding the severity of lung function impairment. Symptoms and objective findings were better aligned when race-specific reference values were not used. Race-neutral prediction algorithms uniformly resulted in reclassifying severity in the African Americans studied. Conclusion: The limited literature does not support the use of race-based lung function prediction equations. However, this assertion does not provide guidance for every specific clinical situation. For African Americans with COPD, the use of race-based prediction equations appears to fall short in enhancing diagnostic accuracy, classifying severity of impairment, or predicting subsequent clinical events. We do not have information comparing race-neutral vs race-based algorithms on prediction of progression of COPD. We conclude that the elimination of race-based reference values potentially reduces underestimation of disease severity in African Americans with COPD. © 2024 Davidson et al.	https://www.scopus.com/inward/record.uri?eid=2-s	Q2	Scopus	EXC_Scope
Indoor Environmental Monitoring and Chronic Respiratory Diseas	2025	Chronic respiratory diseases (CRD), which include Chronic Obstructive Pulmonary Disease (COPD) and asthma, are significant global health issues, with air quality playing a vital role in exacerbating these conditions. This systematic review explores how monitoring indoor air quality (IAQ) can help manage and reduce respiratory exacerbations in CRD patients. A search of the Web of Science database, yielding 301 articles, was conducted following PRISMA guidelines. Of these, 60 met the inclusion criteria, and after screening, 21 articles were analyzed. The review identified substantial gaps in current research: the lack of standardization in IAQ monitoring; the need for considering geographic variability and for long-term longitudinal studies; and the importance of linking monitored air quality data with respiratory health indicators. It also stressed the importance of considering the heterogeneity of patients in the methodological study design, as well as the convenience of introducing recommendation systems to assess the true impact of corrective measures on indoor air quality in the homes of chronic respiratory patients. The integration of home-based IAQ monitoring with machine learning techniques to enhance our understanding of the relationship between IAQ and respiratory health is emerging as a key area for future research. Addressing all these challenges has the potential to mitigate the impact of CRD and improve the quality of life for patients. © 2025 by the authors.	https://www.scopus.com/inward/record.uri?eid=2-s	Q2	Scopus	EXC_Scope
Content, uptake and adherence of exercise interventions after ar	2025	Introduction Pulmonary rehabilitation is underutilised in patients after an acute exacerbation of COPD (AECOPD). Retrieving information regarding the setting, training modalities and the uptake and adherence to exercise interventions for these individuals in a vulnerable state could potentially guide future research. Aim To provide a comprehensive review of the existing literature on the content, uptake and adherence of different exercise interventions for patients after an AECOPD. Methods Eight different databases were searched for 1) patients experiencing an AECOPD and 2) performing any form of exercise intervention. Information on content, uptake and adherence was collected and the Consensus on Exercise Reporting Template (CERT) checklist was performed for each included record. Results 59 distinct interventions were identified between 1998 and 2023 including a total of 9238 patients. All studies included patients requiring hospitalisation for the AECOPD, four studies additionally included patients not requiring hospitalisation for the AECOPD. Nine different settings were identified, with the majority of studies conducted in an inpatient setting (n=26) and including whole-body and strength exercises. The overall uptake was mentioned in 38 (62%) studies and was 70% with a 13% dropout rate. No paper reported the full CERT checklist. Adherence was defined a priori in 16 (27%) studies, with the most common definition being attendance of >80% of sessions. Conclusion Studies properly reporting on the uptake and adherence of well-described interventions, including information regarding fidelity, are needed to further investigate suitable programmes for patients experiencing an AECOPD. © The authors 2025.	https://www.scopus.com/inward/record.uri?eid=2-s	Q2	Scopus	EXC_Scope
Emerging Trends and Innovations in Radiologic Diagnosis of Thc	2025	Over the past decade, Investigative Radiology has published numerous studies that have fundamentally advanced the field of thoracic imaging. This review summarizes key developments in imaging modalities, computational tools, and clinical applications, highlighting major breakthroughs in thoracic diseases - lung cancer, pulmonary nodules, interstitial lung disease (ILD), chronic obstructive pulmonary disease (COPD), COVID-19 pneumonia, and pulmonary embolism - and outlining future directions. Artificial intelligence (AI)-driven computer-aided detection systems and radiomic analyses have notably improved the detection and classification of pulmonary nodules, while photon-counting detector CT (PCD-CT) and low-field MRI offer enhanced resolution or radiation-free strategies. For lung cancer, CT texture analysis and perfusion imaging refine prognostication and therapy planning. ILD assessment benefits from automated diagnostic tools and innovative imaging techniques, such as PCD-CT and functional MRI, which reduce the need for invasive diagnostic procedures while improving accuracy. In COPD, dual-energy CT-based ventilation/perfusion assessment and dark-field radiography enable earlier detection and staging of emphysema, complemented by deep learning approaches for improved quantification. COVID-19 research has underscored the clinical utility of chest CT, radiographs, and AI-based algorithms for rapid triage, disease severity evaluation, and follow-up. Furthermore, tuberculosis remains a significant global health concern, highlighting the importance of AI-assisted chest radiography for early detection and management. Meanwhile, advances in CT pulmonary angiography, including dual-energy reconstructions, allow more sensitive detection of pulmonary emboli. Collectively, these innovations demonstrate the power of merging novel imaging technologies, quantitative functional analysis, and AI-driven tools to transform thoracic disease management. Ongoing progress promises more precise and personalized diagnostic and therapeutic strategies for diverse thoracic diseases. © 2025 Wolters Kluwer Health, Inc. All rights reserved.	https://www.scopus.com/inward/record.uri?eid=2-s	Q2	Scopus	EXC_Scope

Enrollment and dropout rates of individuals with chronic obstructi	2024	Introduction: Telehealth interventions have the potential of improving health outcomes for individuals with chronic obstructive pulmonary disease (COPD). However, the precise impact of telehealth on exacerbation and hospital readmissions remains inconclusive. This lack of knowledge on the effectiveness of telehealth for COPD care might be due to lack of clarity regarding which variables are most strongly associated with enrolment and dropout rates. Objectives: Among individuals with COPD in telehealth studies, we aimed to: (1) estimate the extent to which trial-related variables are associated with enrolment and dropout rates, and identify reasons for dropouts; (2) estimate the extent to which patients-related and intervention-related variables are associated with dropout rates; (3) estimate the effect of enrolment rate and dropout rate on effect size; (4) estimate the effect of trial-related, patient-related, and intervention-related variables on effect size. Methods: A systematic literature search was conducted using four electronic databases. Two independent reviewers screened all retrieved titles, abstracts and full texts according to the inclusion criteria and extracted the data. A random-effect meta-regression analysis was conducted to estimate the overall enrolment and dropout rates, and estimated the different variables' effects on the enrolment rate, dropout rate, and effect sizes in the studies included in the review. Results: A total of 56 studies comprising 7530 participants were identified. The estimated enrolment and dropout rates were 50.3 % and 14.9 %, respectively. Trial-related variables influence enrollment and dropout rates, including RCT designs and the recruitments. The patient-related variables, including age and severity of the disease, and intervention-related variables, including the components of the intervention and mode of delivery, influence dropout rates. Studies with low dropout rates had a bigger effect size by 0.23. The main reported reasons for dropping out of the intervention were related to death (21 %) followed by lost to follow-up (14 %). Conclusion: Trial, patient, and intervention-related variables were found to influence the enrolment and dropout rates. This would help plan and develop a more appealing telehealth intervention that patients can easily accept and incorporate into their everyday lives. Registration information: International Prospective Register of Systematic Reviews (PROSPERO); ID: CRD42017078541. © 2023	https://www.scopus.com/inward/record.uri?eid=2-s	Q2	Scopus	EXC_Scope
Cost-effectiveness, use and implementation of telehealth solutior	2025	Background: According to leading health organizations such as the World Health Organization (WHO) and the Centers for Disease Control and Prevention (CDC), telehealth applications have the potential to improve patients' health, particularly for the billions of patients suffering from chronic diseases such as Congestive Heart Failure (CHF) and Chronic Obstructive Pulmonary Disease (COPD). While telehealth solutions hold promise, there is currently inadequate clinical evidence supporting their use in public health surveillance and home-based care, making it difficult to draw decisive conclusions. Objective: The objective of this work was to evaluate the cost-effectiveness, use, and implementation of telehealth solutions for patients with chronic diseases, specifically CHF and COPD, through a review of the current literature. This narrative review examined studies presenting cost-effectiveness analyses, use, and implementation of telehealth for these patients. Methods: This work implemented the Preferred Reporting Items for Systematic Reviews and Meta-Analyses guidelines. In order to receive recency and to examine recent and innovative telehealth solutions, articles published in English from 2010 to 2023 were included in the search. The inclusion criteria were papers on telehealth tools employed for CHF and COPD patients that assessed their cost-effectiveness. Results: The majority of the studies were conducted in Europe. Approximately half had an adequate sample size and tracked patients prospectively for a sufficient duration. The most frequently used telehealth method was distance monitoring, with only a few studies incorporating home visits or phone calls. The parameters monitored included blood pressure, oxygen saturation, heart rate, and spirometry, among others. General statistical analyses and regression models were the most frequently used methods, although several studies incorporated Markov models and simulations. Discussion: The majority of the papers (20 out of 26) concluded that the tools implemented led to either cost-effectiveness, cost-savings or strongly dominance. This promising result shows that telehealth is an important topic that deserves further research on its effectiveness as well as cost-effectiveness for chronic disease management. Limitations: One key limitation of this PRISMA review is that the literature search was restricted to two major diseases, and the language of the publications was exclusively English. Thus, the generalizability of the findings to other chronic diseases is subject to caution. © 2025 Fellowship of Postgraduate Medicine	https://www.scopus.com/inward/record.uri?eid=2-s	Q2	Scopus	EXC_Scope
Determining If COPD Self-Management Televisit-Based Interven	2024	Topic Importance: With telemedicine's expansion during the COVID-19 pandemic, it has become critical to evaluate whether patients have equitable access to and capabilities to use televisits optimally for improved COPD outcomes such as reduced hospitalizations. This scoping review evaluated whether televisit-based interventions are evaluated among and equitably effective in improving health care use outcomes among diverse patient populations with COPD. Review Findings: Using a systematic search for televisit-based COPD self-management interventions, we found 20 studies for inclusion, all but one of which were published before the COVID-19 pandemic. Most (11 of 20) were considered good-quality studies. Most studies (19 of 20) reported age and sex; few provided race (3 of 20) or income (1 of 20) data. The most frequently used televisit-based methods were in-person plus phone (6 of 20), video only (6 of 20), and phone only (4 of 20). Most studies (12 of 20) showed a significant reduction in at least one health care use metric; nine studies found hospitalization-related reductions. Effective interventions typically used two methods (eg, in-person plus televisits), video methods, or both. Most studies failed to report on participants' race or income, leading to a lack of data on the equity of interventions' effectiveness across diverse patient populations. Multimethod televisit-based interventions, particularly with an in-person component, most commonly were effective; no associations were seen with study quality or size. With the increasing reliance on telemedicine to provide chronic disease care, the lack of data among diverse populations since the COVID-19 pandemic began limits generalizability of these findings for real-world clinical settings. More comprehensive evaluations of televisit-based interventions are needed in the era after the pandemic within and across diverse patient populations. © 2024 American College of Chest Physicians	https://www.scopus.com/inward/record.uri?eid=2-s	Q2	Scopus	EXC_PubType
Digital health technologies to strengthen patient-centred outcome	2025	Common to all inflammatory arthritides, namely rheumatoid arthritis, psoriatic arthritis, axial spondyloarthritis, and juvenile idiopathic arthritis, is a potential for reduced mobility that manifests through joint pain, swelling, stiffness, and ultimately joint damage. Across these conditions, consensus has been reached on the need to capture outcomes related to mobility, such as functional capacity and physical activity, as core domains in randomised controlled trials. Existing endpoints within these core domains rely wholly on self-reported questionnaires that capture patients' perceptions of their symptoms and activities. These questionnaires are subjective, inherently vulnerable to recall bias, and do not capture the granularity of fluctuations over time. Several early adopters have integrated sensor-based digital health technology (DHT)-derived endpoints to measure physical function and activity in randomised controlled trials for conditions including Parkinson's disease, Duchenne's muscular dystrophy, chronic obstructive pulmonary disease, and heart failure. Despite these applications, there have been no sensor-based DHT-derived endpoints in clinical trials recruiting patients with inflammatory arthritis. Borrowing from case studies across medicine, we outline the opportunities and challenges in developing novel sensor-based DHT-derived endpoints that capture the symptoms and disease manifestations most relevant to patients with inflammatory arthritis. © 2025 Elsevier Ltd	https://www.scopus.com/inward/record.uri?eid=2-s	Q2	Scopus	EXC_Scope
A tale as old as time–the importance of accelerated lung aging in	2025	Introduction: Chronic obstructive pulmonary disease (COPD) is progressive in nature and predominantly affects older individuals. Lung function decline is a typical part of the aging process, characterized by gradual loss of lung mechanics, airway remodeling, persistent low-grade inflammation of the airways, compromised epithelial barrier function, and impaired immune responses over time. Areas covered: The pathology of the senile lung is advanced in patients with COPD, whereby genomic damages contribute to structural defects and cellular dysfunction. Primary, antagonistic, and integrative hallmarks of aging are accelerated in COPD, potentiated by cumulative injury sustained from repeated environmental exposures and the interaction with comorbidities. Identification of epigenetic profiles in COPD indicates how cellular processes contribute to the advancement of biological age. Epigenetic abnormalities unique to COPD subpopulations occur in individuals who are immunodeficient, and often experience early onset and increased severity of COPD. Expert opinion: Accelerated aging processes indicated by epigenetic and other biomarkers may be a promising avenue for early detection, prevention, and subsequent management of COPD. Understanding risk factors contributing to progressive lung function decline and implementation of mitigation strategies such as cessation of smoking, repurposing existing pharmacotherapeutics and development of novel therapies may slow age-related pathologies in COPD. (Figure presented.). © 2025 Informa UK Limited, trading as Taylor & Francis Group.	https://www.scopus.com/inward/record.uri?eid=2-s	Q2	Scopus	EXC_Scope

Cognitive Computing in Respiratory Health: Revolutionizing Med	2024	Human errors in medical practice can lead to misdiagnosis, resulting in inappropriate treatment and serious risks to patient health. Artificial Intelligence (AI) and Machine Learning (ML) have emerged as valuable tools for reducing such errors, particularly in medical diagnostics. These technologies can rapidly and accurately analyze large volumes of data, providing additional insights that help healthcare professionals to make more accurate decisions. AI excels in providing solid evidence to guide clinical decisions, reducing reliance on subjective judgments. It can analyse complex datasets and identify patterns that have chances to be overlooked by human eyes, thus leading to improved diagnostic accuracy and treatment plans. ML, a component of AI, uses adaptive models that learn from extensive datasets, though these models must be trained on high-quality data to avoid perpetuating errors or biases. In pulmonary medicine, AI and ML have shown considerable potential in diagnosing and treating conditions such as asthma, chronic obstructive pulmonary disease (COPD), and pulmonary fibrosis. These technologies help determine disease staging, forecast exacerbations, and estimate survival rates. By harnessing AI and ML, clinicians can make more precise diagnoses, customize treatments to individual needs, and detect early signs and ultimately enhance patient outcomes. Moreover, AI and ML can minimize patient risks by providing a broader and more in-depth analysis of medical data. This review explores how these technologies can process large datasets to deliver insights that surpass human capability, fostering error-free diagnosis and treatment. © 2024, Dr Tarak Nath Podder Memorial Foundation. All rights reserved.	https://www.scopus.com/inward/record.uri?eid=2-s	Q2	Scopus	EXC_Scope
Self-management interventions for chronically ill patients with lim	2024	Objectives: To support patients with limited health literacy with the challenges they face in the day-to-day management of their disease(s), numerous self-management interventions (SMIs) have been developed. To date, it is unclear to what extent SMIs have been developed for chronically ill patients with limited health literacy. This study aims to provide a description of these SMIs and to provide insight in their methodological components. Methods: A secondary analysis of the COMPAR-EU database, consisting of SMIs addressing patients with diabetes, chronic obstructive pulmonary disease, obesity and heart failure, was conducted. The database was searched for SMIs addressing health literacy, including cognitive aspects and the capacity to act. Results: Of the 1681 SMIs in the COMPAR-EU database, 35 studies addressed health literacy, describing 39 SMIs. The overview yields a high variety in interventions given, with overlapping information, but also lacking of specific details. Discussion: This descriptive analysis shows that there was a large variety in the extensiveness of the description of intervention characteristics and their justification or explanation. A focus on the broad concept of health literacy, including functional skills, cognitive skills and the capacity to act could improve the effectiveness. This should be taken into account in the future development of SMIs. © The Author(s) 2023.	https://www.scopus.com/inward/record.uri?eid=2-s	Q2	Scopus	EXC_Scope
The effects of tele-based interventions for depression and anxiet	2024	Background: Depression and anxiety are common psychiatric symptoms in patients with Chronic Obstructive Pulmonary Disease (COPD). While face-to-face psychotherapy is a common option, tele-based interventions provide a more accessible alternative. However, a comprehensive synthesis of evidence from clinical trials for COPD patients has yet to be conducted. Objective: This study aims to evaluate the effects of tele-based interventions in reducing depressive and anxiety symptoms in patients with COPD. Methods: A systematic search of PubMed, EMBASE, the Cochrane Library, Web of Science, PsycINFO, and MEDLINE databases was conducted from inception to May 5, 2024. Eligible studies included Randomized Controlled Trials (RCTs) of people with COPD patients receiving tele-based interventions reporting on the outcomes of depression or anxiety. Data extraction and quality assessment were performed independently by two researchers. The quality of the included studies was assessed using the Cochrane risk-of-bias tool. Meta-analysis was performed using RevMan (version 5.4) and Stata (version 18.0) software. Results: Following the search, 9 RCTs with a total of 2064 patients with COPD were included. The meta-analysis revealed that tele-based interventions reduced depressive symptoms in patients with COPD (Standardized Mean Difference [SMD] = −0.15, 95 % CI −0.24 to −0.06; P = 0.001). The subgroup analysis indicated that the PHQ-9 (SMD = −0.24, 95 % CI −0.37 to −0.10; P = 0.001) was better at detecting changes in depressive symptoms compared to other scales; the first 3 months of intervention (SMD = −0.36, 95 % CI −0.52 to −0.19; P < 0.001) was the most pronounced improvement; and telehealth interventions were more effective (SMD = −0.30, 95 % CI −0.46 to −0.15; P < 0.001) than telemonitoring interventions. Tele-based interventions also reduced anxiety symptoms in patients with COPD (SMD = −0.12, 95 % CI −0.22 to −0.02; P = 0.02). Conclusions: The evidence supports the efficacy of tele-based interventions in alleviating depression and anxiety symptoms in COPD patients. However, further large-scale and rigorously designed studies are warranted to strengthen the evidence. © 2024 Elsevier Inc.	https://www.scopus.com/inward/record.uri?eid=2-s	Q2	Scopus	EXC_Scope
Mapping trends and hotspots regarding the use of telenursing fo	2024	Background: Telenursing is receiving extensive attention from scholars and medical staff. However, there are few studies on the knowledge structure of telenursing for elderly individuals with chronic diseases. This study aims to demonstrate current research status and development trend of telenursing for elderly individuals with chronic diseases through a visual analysis of CiteSpace, so as to provide a more comprehensive perspective for future researches. Methods: Literature about telenursing for elderly patients with chronic diseases from 2002 to 2022 was retrieved from the Web of Science Core Collection using CiteSpace 6.1.R3. Results: A total of 375 records were obtained. Annual publication and citation frequency gradually increased over the investigated period, reaching a peak in 2022. Journal of Telemedicine and Telecare was the most prolific and the most cited journal. The United States was the most productive country, the University of Melbourne was the most productive institution, and the author CHEN C ranked the highest in the number of publications. The most popular keywords were "care," "telemedicine," "management," "older adult," "chronic disease," "health," and "heart failure," which had a high frequency and centrality. The keywords "telehealth," "randomized controlled trail," "chronic obstructive pulmonary disease," "implementation" and "time" showed the strongest citation burst. The keywords were clustered to form 10 labels. The article published in 2010 by Chaudhry SI was cited the most. The top 3 cited journals were all special journal of telemedicine. Conclusion: This study revealed current research status and development trend of telenursing for elderly individuals with chronic diseases. The bibliometric analysis of telenursing expands the knowledge field of telemedicine and provides new insights into the management of elderly patients with chronic diseases. © 2024 Lippincott Williams and Wilkins. All rights reserved.	https://www.scopus.com/inward/record.uri?eid=2-s	Q2	Scopus	EXC_PubType
Impact of lifestyle risk factors on atrial fibrillation: Mechanisms ar	2024	Both the development and progression of atrial fibrillation (AF) are affected by a range of modifiable lifestyle risk factors. These key modifiable risk factors encompass obesity, hypertension, hypercholesterolemia, diabetes mellitus, smoking, chronic obstructive pulmonary disease, alcohol consumption, exercise, sedentary lifestyle and obstructive sleep apnoea. These lifestyle-dependent factors rarely exist in isolation, but rather exist together, exerting a complex influence on the development of AF. This comprehensive review elucidates the interplay and interdependency of these lifestyle factors in the arrhythmogenesis of AF, by exploring their role in AF substrate formation, modulating properties and triggering mechanisms. We emphasize the importance of targeted prevention strategies by discussing available literature on the effectiveness of treatment strategies targeting multiple risk factors. Additionally, the clinical impacts of integrated care, nurse-led care and mobile health are discussed in the context of lifestyle improvement. These management strategies have favourable applicability in both paroxysmal and persistent AF, and are also beneficial for patients receiving AF ablation. Despite the challenges accompanying lifestyle and prevention strategies, substantial benefits are apparent, such as improved quality of life and better ablation outcomes. This review further emphasizes the essential nature of awareness of appropriate lifestyle modifications as fundamental pillars in the management of individuals with AF. © 2024 The Authors	https://www.scopus.com/inward/record.uri?eid=2-s	Q2	Scopus	EXC_Scope
Smartphone applications supporting self-management programm	2024	Introduction: Chronic Obstructive Pulmonary Disease (COPD) significantly impacts on both the quality and quantity of life for patients due to frequent exacerbations requiring hospital admissions resulting in increased morbidity and mortality. A self-management programme purpose is to increase one’s knowledge, confidence, and skills to self-manage their chronic illness such as COPD. Objective: The objective of this review will therefore answer the following research question: What is the current literature pertaining to the use of a smartphone app in supporting a comprehensive self-management programme among COPD patients? A preliminary search was conducted in, Medline, Embase and CINAHL databases to ascertain index terms and keywords. Following this a rigorous search was carried out on Medline, Embase, CINAHL, Web of Science and ASSIA. The findings from this search are presented in tabular form using the PRSIMA flow diagram. Results: In this review, fifteen studies met the inclusion criteria. Across all studies participants engaged with the app and developed self-management skills and knowledge to manage their chronic illness. However, engagement with the app without third party involvement declined over time. Technical issues did not cause harm to participants but in some cases contributed to reduced engagement. Smartphone self-management apps empowered a cohort of COPD participants to engage in managing their chronic illness which proved useful in detecting exacerbations earlier resulting in reducing the need for hospitalisations over a three-to-six-month period. By reducing hospitalisations incurred a cost savings for health care and an improved quality and quantity of life for these participants. Conclusion: It is evident from the literature that smartphone self-management apps may positively influence participants self-management decisions in terms of knowledge, increase physical activity, self-efficacy that may result in reduced hospitalisation and improved quality of life. It is clear that technical issues and sustained engagement over longer periods of time remains a challenge. © 2024 Glynn et al. This is an open access article distributed under the terms of the Creative Commons Attribution License, which permits unrestricted use, distribution, and reproduction in any medium, provided the original author and source are credited.	https://www.scopus.com/inward/record.uri?eid=2-s	Q2	Scopus	EXC_Scope

Facilitators and barriers affecting the implementation of e-health	2025	Background: Chronic respiratory diseases are important causes of disability and mortality globally. Their incidence may be higher in remote locations where healthcare is limited and risk factors, such as smoking and indoor air pollution, are more prevalent. E-health could overcome some healthcare access obstacles in remote locations, but its utilisation has been limited. An improved understanding of barriers and facilitators to the implementation of e-health in remote locations could aid enhanced application of these approaches. Methods: We performed a qualitative evidence synthesis to explore factors affecting the successful implementation of e-health interventions in remote locations for patients with chronic respiratory diseases. We searched PubMed, CINAHL, Embase, Web of Science and PsycINFO databases for qualitative and mixed-methods studies. Studies were assessed by two researchers, and 41 studies were included in the synthesis. Quality was assessed via the CASP-tool. Findings were coded with Atlas.ti software and categorised based on an adapted Digital Health Equity Framework. Results: Nineteen themes were identified across five levels (individual, interpersonal, community, society and technology), with associated facilitators and barriers for implementation. An important facilitator of e-health was its role as a tool to overcome obstacles of distance and to increase access to care and patients' self-efficacy. Potential barriers included the reduction of in-person interactions and an increased burden of work for healthcare providers. Good quality, usability, adaptability and efficacy of e-health interventions were important for implementation to be successful, as were adaptation to the local setting —including culture and language —and involvement of relevant stakeholders throughout the process. Conclusions: Several factors affecting the implementation of e-health in remote and rural locations for patients with chronic respiratory disease were identified. Intervention objectives, target population, geographical location, local culture, and available resources should be carefully considered when designing an e-health intervention. These findings can be used to inform the successful design and implementation of future e-health interventions. © The Author(s) 2025.	https://www.scopus.com/inward/record.uri?eid=2-s	Q2	Scopus	EXC_PubType
Digital Health Interventions in Older Adult Populations Living Witl	2024	Background: Globally, around 80% percent of adults aged 65 years or older are living with at least 1 chronic disease, and 68% percent have 2 or more chronic diseases. Older adults living with chronic diseases require greater health care services, but these health care services are not always easily accessible. Furthermore, the COVID-19 pandemic has resulted in unprecedented changes in the provision of health care services for older adults. During the COVID-19 pandemic, digital health interventions for chronic disease management were developed out of necessity, but the evidence regarding these and developed interventions is lacking. Objective: In this scoping review, we aim to identify available digital health interventions such as emails, text messages, voice messages, telephone calls, video calls, mobile apps, and web-based platforms for chronic disease management for older adults in high-income countries. Methods: We will follow the Arksey and O'Malley framework to conduct the scoping review. Our full search strategy was developed following a preliminary search on MEDLINE. We will include studies where older adults are at least 65 years of age, living with at least 1 chronic disease (eg, cancer, cardiovascular disease, chronic obstructive pulmonary disease, and diabetes), and residing in high-income countries. Digital health interventions will be broadly defined to include emails, text messages, voice messages, telephone calls, video calls, mobile apps, and web-based platforms. Results: This scoping review is currently ongoing. As of March 2023, our full search strategy has resulted in a total of 9901 records. We completed the screening of titles and abstracts and obtained 442 abstracts for full-text review. We are aiming to complete our full-text review in October 2024, data extraction in November 2024, and data synthesis in December 2024. Conclusions: This scoping review will generate evidence that will contribute to the further development of digital health interventions for future chronic disease management among older adults in high-income countries. More evidence-based research is needed to better understand the feasibility and limitations associated with the use of digital health interventions for this population. These evidence-based findings can then be disseminated to decision-makers and policy makers in other high-income countries. © 2024 JMIR Publications Inc.. All rights reserved.	https://www.scopus.com/inward/record.uri?eid=2-s	Q2	Scopus	EXC_Scope
Early Integration of Palliative Care in Nononcological Patients: A	2025	Introduction: Palliative care (PALC) is traditionally linked to end-of-life cancer care but also benefits advanced nononcological diseases. Objectives: This systematic review evaluated the impact of early PALC on quality of life (QOL), symptom management, advance care planning (ACP), and healthcare resource utilization (HRU) among nononcological patients. Methods: PubMed, Web of Science, and Scopus databases were searched for randomized controlled trials and clinical studies published between January 2018 and April 2023. Participants were adult patients with nononcological diseases exposed to PALC interventions compared to usual care. Outcomes included QOL, symptom management, ACP, and HRU. The risk of bias was assessed using Cochrane tools. Results: Seven studies were included involving 1118 patients. Early PALC positively affects pain interference and fatigue in heart failure (HF) patients and time until first readmission and days alive outside the hospital in end-stage liver disease (ESLD) patients. Benefits were noted in symptom burden for patients with Human Immunodeficiency Virus (HIV), anxiety and depression in stroke patients, and ACP in chronic obstructive pulmonary disease (COPD) and idiopathic pulmonary fibrosis (IPF) patients. However, results for anxiety and depression in HF patients are inconsistent, and no significant differences in QOL were observed in HF, ESLD, IPF, and COPD. The intervention did not improve overall QOL in HIV. Conclusions: The impact of early PALC on health outcomes in nononcological diseases is inconsistent. Addressing barriers to early PALC integration and conducting further high-quality research are essential for optimizing care pathways and enhancing patient outcomes. © 2025 The Authors	https://www.scopus.com/inward/record.uri?eid=2-s	Q2	Scopus	EXC_Scope
Telerehabilitation in Chronic Obstructive Pulmonary Disease (CC	2024	Telerehabilitation has emerged as a promising approach to address the challenges posed by chronic obstructive pulmonary disease (COPD) management. This article provides a comprehensive review of telerehabilitation's characteristics, components, efficacy, patient experiences, and the implications for COPD patients. Telerehabilitation involves the use of telecommunication technologies to remotely deliver rehabilitation services, including exercise training, education, and monitoring, to individuals with COPD. It addresses barriers such as geographical distance and limited access to specialized facilities, making rehabilitation accessible from home. Telerehabilitation encompasses various approaches, including telemonitoring, teleconsultation, and tele-education. Several studies have indicated positive outcomes in exercise capacity, dyspnea, and quality of life when comparing telerehabilitation interventions to standard care or traditional rehabilitation programs. However, challenges related to technology access, digital literacy, and privacy need to be addressed for equitable implementation. Interdisciplinary collaboration among healthcare professionals is essential for the successful delivery of telerehabilitation. Future research should focus on refining the interventions, standardizing the protocols, and evaluating the long-term maintenance effects. By integrating patient-centered approaches and leveraging advancements in digital technologies, telerehabilitation can revolutionize COPD management strategies, enhancing patient outcomes in the modern healthcare landscape. © 2024 JHSMR.	https://www.scopus.com/inward/record.uri?eid=2-s	Q2	Scopus	EXC_PubType
Clinically important changes and adverse events with centre-bas	2024	Objectives: To determine the proportion of people who achieve minimal clinically important differences (MCID) with centre-based or home-based pulmonary rehabilitation and to synthesise data on adverse events. Methods: Cochrane reviews and electronic databases were searched to identify randomised trials comparing centre-based to home-based pulmonary rehabilitation, or either model to usual care, in people with chronic respiratory disease. Primary outcomes were the proportion of participants achieving MCIDs in exercise capacity and disease-specific quality of life. Secondary outcomes were symptoms and adverse events. Cochrane Risk of Bias 1.0 and GRADE were used to assess the risk of bias and certainty of evidence respectively. Results: Forty-nine trials were eligible. Compared to usual care, a higher proportion of pulmonary rehabilitation participants achieved the MCID for exercise capacity (6MWT: 47% vs 20%, p = 0.11), dyspnoea (43% vs 29%, p = 0.0001), fatigue (48% vs 27%, p = 0.0002) and emotional function (37% vs 25%, p = 0.02), with all of these between group differences statistically significant except for exercise capacity. There were no differences between centre-based and home-based pulmonary rehabilitation in the proportion of participants who achieved MCIDs (34%- 58% across studies). Ninety percent of trials reported no adverse events. Certainty of evidence was low-to- moderate with all outcomes except for CRQ-mastery (centre-based vs home-based pulmonary rehabilitation, or pulmonary rehabilitation vs usual care in COPD), ESWT (pulmonary rehabilitation vs usual care in COPD) and 6MWT (pulmonary rehabilitation vs usual care in bronchiectasis) where evidence was very uncertain. Discussion: Clinically meaningful outcomes are achieved by similar proportions of participants in centre-based and home-based pulmonary rehabilitation, with few adverse events. Reporting of trial outcomes according to MCIDs is necessary for informed decision making regarding pulmonary rehabilitation models. © The Author(s) 2024.	https://www.scopus.com/inward/record.uri?eid=2-s	Q2	Scopus	EXC_Scope

Evaluation of Telemedicine Consultations Using Health Outcome	2024	Background: Despite a recent rise in adoption, telemedicine consultations retention remains challenging, and aspects around the associated experiences and outcomes remain unclear. The need to further investigate these aspects was a motivating factor for conducting this scoping review. Objective: With a focus on synchronous telemedicine consultations between patients with nonmalignant chronic illnesses and health care professionals (HCPs), this scoping review aimed to gain insights into (1) the available evidence on telemedicine consultations to improve health outcomes for patients, (2) the associated behaviors and attitudes of patients and HCPs, and (3) how supplemental technology can assist in remote consultations. Methods: PRISMA-ScR (Preferred Reporting Items for Systematic Reviews and Meta-Analyses extension for Scoping Reviews) guided the scoping review process. Inclusion criteria were (1) involving adults with nonmalignant, noncommunicable chronic conditions as the study population; (2) focusing on health outcomes and experiences of and attitudes toward synchronous telemedicine consultations between patients and HCPs; and (3) conducting empirical research. A search strategy was applied to PubMed (including MEDLINE), CINAHL Complete, APA PsycNet, Web of Science, IEEE, and ACM Digital. Screening of articles and data extraction from included articles were performed in parallel and independently by 2 researchers, who corroborated their findings and resolved any conflicts. Results: Overall, 4167 unique articles were identified from the databases searched. Following multilayer filtration, 19 (0.46%) studies fulfilled the inclusion criteria for data extraction. They investigated 6 nonmalignant chronic conditions, namely chronic obstructive pulmonary disease, diabetes, chronic kidney disease, ulcerative colitis, hypertension, and congestive heart failure, and the telemedicine consultation modality varied in each case. Most observed positive health outcomes for patients with chronic conditions using telemedicine consultations. Patients generally favored the modality's convenience, but concerns were highlighted around cost, practical logistics, and thoroughness of clinical examinations. The majority of HCPs were also in favor of the technology, but a minority experienced reduced job satisfaction. Supplemental technological assistance was identified in relation to technical considerations, improved remote workflow, and training in remote care use. Conclusions: For patients with noncommunicable chronic conditions, telemedicine consultations are generally associated with positive health outcomes that are either directly or indirectly related to their ailment, but sustained improvements remain unclear. These modalities also indicate the potential to empower such patients to better manage their condition. HCPs and patients tend to be satisfied with remote care experience, and most are receptive to the modality as an option. Assistance from supplemental technologies mostly resides in addressing technical issues, and additional modules could be integrated to address challenges relevant to patients and HCPs. However, positive outcomes and attitudes toward the modality might not apply to all cases, indicating that telemedicine consultations are more appropriate as options rather than replacements of in-person visits. © 2024 JMIR Publications Inc.. All rights reserved.	https://www.scopus.com/inward/record.uri?eid=2-s	Q2	Scopus	EXC_PubType
Contextual factors for the successful implementation of self-mana	2024	Objectives: To identify and describe the most relevant contextual factors (CFs) from the literature that influence the successful implementation of self-management interventions (SMIs) for patients living with type 2 diabetes mellitus, obesity, COPD and/or heart failure. Methods: We conducted a qualitative review of reviews. Four databases were searched, 929 reviews were identified, 460 screened and 61 reviews met the inclusion criteria. CFs in this paper are categorized according to the Tailored Implementation for Chronic Diseases framework. Results: A great variety of CFs was identified on several levels, across all four chronic diseases. Most CFs were on the level of the patient, the professional and the interaction level, while less CFs were obtained on the level of the intervention, organization, setting and national level. No differences in main themes of CFs across all four diseases were found. Discussion: For the successful implementation of SMIs, it is crucial to take CFs on several levels into account simultaneously. Person-centered care, by tailoring SMIs to patients' needs and circumstances, may increase the successful uptake, application and implementation of SMIs in real-life practice. The next step will be to identify the most important CFs according to various stakeholders through a group consensus process. © The Author(s) 2023.	https://www.scopus.com/inward/record.uri?eid=2-s	Q2	Scopus	EXC_PubType
A history of home mechanical ventilation: The past, present and f	2024	This state-of-the-art review provides an overview of the history of home mechanical ventilation (HMV), including early descriptions of mechanical ventilation from ancient and Renaissance perspectives and the mass development of ventilators designed for long-term use during the poliomyelitis epidemic. Seminal data from key clinical trials supports the application of HMV in certain patients with chronic obstructive pulmonary disease, neuromuscular disease and obesity-related respiratory failure. Innovative engineering coupled with refined physiological understanding now permits widespread delivery of home mechanical ventilation to a global population, using portable devices with advanced ventilatory modes and telemonitoring capabilities. Exponential growth in digital technology continues, and ongoing research is needed to understand how to harness clinical and physiological data to benefit patients and healthcare services in a clinically- and cost-effective manner. © The Author(s) 2024.	https://www.scopus.com/inward/record.uri?eid=2-s	Q2	Scopus	EXC_Scope
Core characteristics, and effectiveness of mobile health intervent	2025	Aim: To systematically review the core characteristics, and effectiveness of mobile health interventions (MHIs) on dyspnoea and quality of life (QoL) in older persons with chronic obstructive pulmonary disease (COPD). Methods: A comprehensive search was conducted from inception to 21 February 2023 in Chinese and English databases, with an updated search performed on 30 April 2024. Randomised controlled trials (RCTs) involving MHIs with four core functions — customisation, self-monitoring, alerts, and goal-setting — in older persons with COPD were included. Two reviewers independently identified the core characteristics of MHIs using the Template for Intervention Description and Replication (TIDieR) checklist. The Cochrane risk-of-bias tool and the Grading of Recommendations, Assessment, Development, and Evaluation (GRADE) approach were used to assess the methodological quality of RCTs and the overall quality of evidence. Results: 28 RCTs were included, with 43% of studies adequately reported core characteristics of MHIs according to the TIDieR checklist. Meta-analysis suggested that MHIs may alleviate dyspnoea and improve disease-specific QoL questionnaires (CRQ and CCQ) and generic-related QoL questionnaires (SF-36 and EQ-5D), but not other disease-specific QoL questionnaires (SGRQ and CAT). Subgroup analyses showed that multi-component MHIs were effective in improving dyspnoea. Dyspnoea showed statistically significant improvement at 3 months, 6 months and 12 months, whereas QoL improved at 4 months (SGRQ), 2 months (CAT) and 6 months (CAT). Conclusion: Reporting on the core characteristics of MHIs is currently incomplete, and it is recommended that healthcare professionals develop and report multi-component MHIs based on the TIDieR checklist to help alleviate dyspnoea and enhance QoL in older persons with COPD. © 2025 Elsevier Inc.	https://www.scopus.com/inward/record.uri?eid=2-s	Q2	Scopus	EXC_Scope
The benefits and drawbacks of home oxygen therapy for COPD:	2024	Introduction: Home oxygen therapy is one of the few interventions that can improve survival in patients with chronic obstructive pulmonary disease (COPD) when administered appropriately, although it may cause side effects and be an unnecessary burden for some patients. Areas covered: This narrative review summarizes the current literature on the assessment of hypoxemia, different types of home oxygen therapy, potential beneficial and adverse effects, and emerging research on home oxygen therapy in COPD. A literature search was performed using MEDLINE and EMBASE up to January 2024, with additional articles being identified through clinical guidelines. Expert Opinion: Hypoxemia is common in patients with more severe COPD. Long-term oxygen therapy is established to prolong survival in patients with chronic severe resting hypoxemia. Conversely, in the absence of chronic severe resting hypoxemia, home oxygen therapy has an unclear or conflicting evidence base, including for palliation of breathlessness, and is generally not recommended. However, beneficial effects in some patients cannot be precluded. Evidence is emerging on the optimal daily duration of oxygen use, the role of high-flow and auto-titrated oxygen therapy, improved informed decision-making, and telemonitoring. Further research is needed to validate novel oxygen delivery systems and monitoring tools and establish long-term effects of ambulatory oxygen therapy in COPD. © 2024 Informa UK Limited, trading as Taylor & Francis Group.	https://www.scopus.com/inward/record.uri?eid=2-s	Q2	Scopus	EXC_Scope
Assessment of Medication Adherence Using Mobile Applications	2024	Chronic obstructive pulmonary disease (COPD) is a condition that significantly impacts both patients and healthcare systems. The management of COPD involves various pharmacological intervention strategies, and addressing the issue of low adherence to these strategies has become a subject of significant interest. In response to this concern, there has been a shift toward utilizing telemedicine and mobile applications. The primary objective of this scoping review is to delineate the usage of mobile applications to enhance medication adherence in adult patients with COPD. This study involved a search of databases such as Medline, Google Scholar, Cochrane, and ClinicalTrial.gov, focusing on the literature published in English and Spanish over the last decade. The selected studies assessed interventions involving mobile applications (mobile apps) designed to improve medication adherence. Four digital aids were identified and available on online platforms, mobile apps, or both: m-PAC, myCOPD, Wellinks mHealth, and Propeller Health. Propeller Health, in particular, is an app that directly measures medication adherence through electronic medication monitors attached to participants' inhalers. Opening the app was associated with higher odds of using control medications compared to participants who did not open the app. The findings suggest that these digital interventions serve as valuable tools to enhance patient adherence to treatment. Future research should focus on evaluating the effectiveness of different digital devices, such as digital inhalers and mobile applications, that directly measure medication adherence. © 2024 by the authors.	https://www.scopus.com/inward/record.uri?eid=2-s	Q2	Scopus	EXC_PubType

Addressing Barriers to Chronic Obstructive Pulmonary Disease (	2024	Chronic obstructive pulmonary disease (COPD) is a preventable yet widespread and profoundly debilitating respiratory condition, exerting substantial personal and global health ramifications alongside significant economic implications. The first objective of this literature review was to identify reviews the barriers to optimal COPD care, categorizing them into personal patient factors, professional awareness and knowledge, patient-professional relationships, and healthcare service models, including access to care that significantly impacts the quality of COPD management. The second objective was to introduce three approaches for enhancing COPD care outcomes: Self-Management Educational Programs, Health Qigong, and Telehealth service provision, each demonstrating positive effects on COPD patients' health status. These evidence-based interventions offer promising avenues for enhancing COPD care and patient outcomes. Integrating these approaches into comprehensive COPD management strategies holds potential for improving the well-being and quality of life of individuals living with this chronic condition. © 2024 Siu and Gafni-Lachter.	https://www.scopus.com/inward/record.uri?eid=2-s	Q2	Scopus	EXC_Scope
Transforming pulmonary health care: the role of artificial intelliger	2025	Introduction: Respiratory diseases like pneumonia, asthma, and COPD are major global health concerns, significantly impacting morbidity and mortality rates worldwide. Areas covered: A selective search on PubMed, Google Scholar, and ScienceDirect (up to 2024) focused on AI in diagnosing and treating respiratory conditions like asthma, pneumonia, and COPD. Studies were chosen for their relevance to prediction models, AI-driven diagnostics, and personalized treatments. This narrative review highlights technological advancements, clinical applications, and challenges in integrating AI into standard practice, with emphasis on predictive tools, deep learning for imaging, and patient outcomes. Expert opinion: Despite these advancements, significant challenges remain in fully integrating AI into pulmonary health care. The need for large, diverse datasets to train AI models is critical, and concerns around data privacy, algorithmic transparency, and potential biases must be carefully managed. Regulatory frameworks also need to evolve to address the unique challenges posed by AI in health care. However, with continued research and collaboration between technology developers, clinicians, and policymakers, AI has the potential to revolutionize pulmonary health care, ultimately leading to more effective, efficient, and personalized care for patients. © 2025 Informa UK Limited, trading as Taylor & Francis Group.	https://www.scopus.com/inward/record.uri?eid=2-s	Q2	Scopus	EXC_PubType
Palliative care for chronic respiratory diseases in low- and middle	2025	Palliative care is essential for patients with chronic pulmonary diseases, especially in low- and middle-income countries (LMICs). Chronic respiratory diseases (CRDs), such as chronic obstructive pulmonary disease and interstitial lung diseases, cause significant morbidity and mortality globally, with a heavy burden in LMICs. Despite the need, access to palliative care in LMICs is limited, leading to inadequate symptom management and support. Palliative care benefits include improved quality of life, reduced healthcare costs, and increased patient and family satisfaction. However, barriers in LMICs, including limited resources, infrastructure, and trained providers, as well as cultural and regulatory challenges, hinder care delivery. Early integration of palliative care for patients with CRDs can enhance outcomes and reduce healthcare utilization, yet it remains underutilized in these regions. This review highlights the challenges and impact of palliative care for CRDs in these regions. Addressing these issues requires regulatory reforms, provider education, and investments in healthcare infrastructure. Solutions include national policies, training healthcare professionals, telemedicine, and research collaborations. Understanding and addressing barriers to palliative care in LMICs is crucial for improving care quality and outcomes for patients with CRDs. © The Author(s), 2025.	https://www.scopus.com/inward/record.uri?eid=2-s	Q2	Scopus	EXC_Scope
The State of the Art of eHealth Self-Management Interventions fc	2025	Background: Chronic obstructive pulmonary disease (COPD) is a common chronic incurable disease. Treatment of COPD often focuses on symptom management and progression prevention using pharmacological and nonpharmacological therapies (eg, medication, inhaler use, and smoking cessation). Self-management is an important aspect of managing COPD. Self-management interventions are increasingly delivered through eHealth, which may help people with COPD engage in self-management. However, little is known about the actual content of these eHealth interventions. Objective: This literature review aimed to investigate the state-of-the-art eHealth self-management technologies for COPD. More specifically, we aimed to investigate the functionality, modality, technology readiness level, underlying theories of the technology, the positive health dimensions addressed, the target population characteristics (ie, the intended population, the included population, and the actual population), the self-management processes, and behavior change techniques. Methods: A scoping review was performed to answer the proposed research questions. The databases PubMed, Scopus, PsycINFO (via EBSCO), and Wiley were searched for relevant articles. We identified articles published between January 1, 2012, and June 1, 2022, that described eHealth self-management interventions for COPD. Identified articles were screened for eligibility using the web-based software Rayyan.ai. Eligible articles were identified, assessed, and categorized by the reviewers, either directly or through a combination of methods, using Atlas.ti version 9.1.7.0. Thereafter, data were charted accordingly and presented with the purpose of giving an overview of currently available literature while highlighting existing gaps. Results: A total of 101 eligible articles were included. This review found that most eHealth technologies (91/101, 90.1%) enable patients to self-monitor their symptoms using (smart) measuring devices (39/91, 43%), smartphones (27/91, 30%), or tablets (25/91, 27%). The self-management process of "taking ownership of health needs" (94/101, 93.1%), the behavior change technique of "feedback and monitoring" (88/101, 87%), and the positive health dimension of "bodily functioning" (101/101, 100%) were most often addressed. The inclusion criteria of studies and the actual populations reached show that a subset of people with COPD participate in eHealth studies. Conclusions: The current body of literature related to eHealth interventions has a strong tendency toward managing the physical aspect of COPD self-management. The necessity to specify inclusion criteria to control variables, combined with the practical challenges of recruiting diverse participants, leads to people with COPD being included in eHealth studies that only represent a subgroup of the whole population. Therefore, future research should be aware of this unintentional blind spot, make efforts to reach the underrepresented population, and address multiple dimensions of the positive health paradigm. © 2025 JMIR Publications Inc.. All rights reserved.	https://www.scopus.com/inward/record.uri?eid=2-s	Q2	Scopus	EXC_PubType
Effects of different traditional Chinese exercises on pulmonary fu	2024	Objective: Traditional Chinese exercises (Taichi, Wuqinxi, Liuzijue, and Baduanjin) are considered effective alternative treatments for improving symptoms in the stable phase of COPD. However, the most effective exercise remains unknown. This study compared the effectiveness of different traditional Chinese exercises on pulmonary function in patients with stable chronic obstructive pulmonary disease (COPD) using a network meta-analysis. Methods: From database establishment until September 2023, eligible randomized controlled trials (RCTs) were searched. Two reviewers performed the risk of bias assessment of the included studies using the Cochrane Collaboration tool, and the evidence level was suggested using the GRADE system. Results: Fifty-seven studies comprising 4294 patients were included. The results of the network meta-analysis show that Baduanjin was most effective in improving the forced expiratory volume in the first second (FEV1). However, Liuzijue significantly improved the first–second forced vital capacity percentage of expected value (FEV1%) and the ratio of the forced expiratory volume in the first second to the forced vital capacity (FEV1/FVC). The probability ranking results indicated that Liuzijue was the most effective, followed by Baduanjin, Wuqinxi, and Taichi. Subgroup analysis in conjunction with intervention duration revealed that Liuzijue had a significant advantage over other interventions for improving FEV1, FEV1%, and FEV1/FVC within 6 months and improved FEV1% and FEV1/FVC for ≥ 6 months. Moreover, Subgroup analysis based on baseline pulmonary function revealed that Liuzijue had a significant advantage over other interventions for improving FEV1% within severe and moderate groups. Finally, Subgroup analysis based on the frequency of interventions showed that Liuzijue was still more effective in improving FEV1, FEV1%, and FEV1/FVC in the ≥ three times one week. Conclusion: Liuzijue was more effective than Taichi, Wuqinxi, Liuzijue, and Baduanjin in improving pulmonary function in patients with stable COPD. © The Author(s) 2024.	https://www.scopus.com/inward/record.uri?eid=2-s	Q2	Scopus	EXC_Scope
Applying behavioral change theories to optimize pulmonary reha	2024	This review meticulously evaluates the integration of behavioral change theories into pulmonary rehabilitation programs for chronic obstructive pulmonary disease (COPD) management, addressing the critical need for enhanced patient compliance and improved therapeutic outcomes. With COPD posing significant global health challenges, characterized by high morbidity and mortality rates, the manuscript underscores the potential of Self-Determination Theory, Social Cognitive Theory, the Transtheoretical Model, the Health Belief Model, and the Theory of Planned Behavior to foster meaningful health behavior changes among patients. Through a comprehensive literature analysis, it reveals how each model contributes to understanding patient behaviors in pulmonary rehabilitation contexts, advocating for their systematic application to craft more effective, patient-centered interventions. Despite the proven efficacy of these theories in various health domains, their current underutilization in pulmonary rehabilitation underscores a gap between theoretical knowledge and clinical practice. The review calls for an interdisciplinary approach that bridges this gap, highlighting the urgency of developing actionable, theory-based behavioral intervention plans. By doing so, it aims to advance COPD management strategies, ultimately improving the quality of life for individuals living with this debilitating disease. © 2024 Lippincott Williams and Wilkins. All rights reserved.	https://www.scopus.com/inward/record.uri?eid=2-s	Q2	Scopus	EXC_Scope

Digital remote maintenance inhaler adherence interventions in C	2024	Introduction Sub-optimal inhaler adherence undermines the efficacy of pharmacotherapy in COPD. Digitalised care pathways are increasingly used to improve inhaler-use behaviour remotely. This review investigated the feasibility and impact of remote electronic inhaler adherence monitoring (EIM) and intervention platforms on clinical outcomes in COPD. Methods A literature search was conducted and studies investigating maintenance inhaler use among people with COPD using digital technology were selected. Pairwise and proportional meta-analyses were employed with heterogeneity assessed using I2 statistics. When meta-analysis was not feasible, a narrative synthesis of outcomes was conducted. Results We included 10 studies including 1432 people with COPD whose maintenance inhaler usage was supported by digital inhalers and apps featuring audiovisual reminders and educational content with or without engagement with healthcare providers (HCPs). Inhaler adherence rate (AR) varied with calculation methods, but an overall suboptimal adherence was observed among people with COPD. HCP-led adherence interventions alongside EIM improved mean AR by 18% (95% CI 9–27) versus passive EIM only. Enhanced AR may reduce COPD-related healthcare utilisation with little impact on health-related quality of life and exacerbation rate. Despite encountering technical issues among 14% (95% CI 5–23%) of participants, 85% (95% CI 76–94%) found digital platforms convenient to use, while 91% (95% CI 79– 100%) perceived inhaler reminders as helpful. Conclusion Digitalised interventions can enhance maintenance inhaler adherence in COPD but their overall effect on clinical outcomes remains uncertain. Further work is required to tailor interventions to individuals' adherence behaviour and investigate their longer-term impact. © The authors 2024.	https://www.scopus.com/inward/record.uri?eid=2-s	Q2	Scopus	EXC_Scope
Early, integrated palliative care for people with chronic respiratory	2025	Lung cancer and chronic non-malignant respiratory disease cause pervasive, multifactorial suffering for patients and informal carers alike. Palliative care aims to reduce suffering and improve quality of life for patients and their families. An established evidence base exists that has demonstrated the essential role of specialist palliative care for people with lung cancer. Emerging evidence supports similar benefits among people with chronic respiratory disease. Many lessons can be learnt from lung cancer care, particularly as the model of care delivery has transformed over recent decades due to major advances in the diagnostic pathway and the development of new treatments. This narrative review aims to summarize the evidence for specialist palliative care in lung cancer and chronic respiratory disease, by highlighting seven key lessons from lung cancer care that can inform the development of proactive, integrated models of palliative care among those with chronic respiratory disease. These seven lessons emphasize (1) managing challenging symptoms; (2) the efficacy of specialist palliative care; (3) the importance of providing specialist palliative care integrated with disease-directed care according to patients' needs not prognosis; (4) the need for new models of collaborative palliative care, (5) which are culturally appropriate and (6) able to evolve with changes in disease-directed care. Finally, we discuss (7) some of the critical research gaps that persist and reduce implementation in practice. © The Author(s), 2025.	https://www.scopus.com/inward/record.uri?eid=2-s	Q2	Scopus	EXC_Scope
Systematic review of tailored dietary advice and dietitian involver	2024	Background: Chronic obstructive pulmonary disease (COPD) is a leading public health concern globally. Interdisciplinary pulmonary rehabilitation programs exist and should ideally consider nutritional health impacts since the nutritional status of COPD patients is often compromised. However, little is known about the role of dietary counseling in COPD management. Research question: Does providing tailored dietary advice to adult patients with COPD improve outcomes? Study design and methods: We conducted a systematic review. The following electronic databases and registrars were used: MEDLINE, EMBASE, Web of Science, CINAHL, Cochrane Library, and ClinicalTrials.gov. The original search was conducted in June 2021 with an updated search conducted on February 21, 2024. Validity and bias assessments were completed. Results: We selected 14 articles for inclusion. Multiple outcomes were considered including functional, body composition, nutritional intake, cost analyses, quality of life, and others. The most common measured outcomes were quality of life and the 6 min walk test. A number of interventions were used with most interventions being interdisciplinary pulmonary rehabilitation packages where nutrition counseling was one component. A number of interventions showed positive results but there tended to be inconsistency. Interpretation: Evidence shows that various interventions appear to improve outcomes, but it is difficult to determine if improvements are due to nutritional intervention specifically or a rehabilitation program as a whole. More specific randomized controlled trials should be completed regarding tailored nutritional counseling and therapy in adults with COPD to determine the benefits attributable to nutritional interventions. © 2024	https://www.scopus.com/inward/record.uri?eid=2-s	Q2	Scopus	EXC_Scope
Early Identification of Exacerbations in Patients with Chronic Obs	2025	Exacerbations of Chronic Obstructive Pulmonary Disease (COPD) have a substantial effect on overall disease management, health system costs, and patient outcomes. However, exacerbations are often underdiagnosed or recognized with great delay due to several factors such as patients' inability to differentiate between acute episodes and symptom fluctuations, delays in seeking medical assistance, and disparities in dyspnea perception. Self-management intervention plans, telehealth and smartphone-based programs provide educational material, counseling, virtual hospitals and telerehabilitation, and help COPD patients to identify exacerbations early. Moreover, biomarkers such as blood eosinophil count, fibrinogen, CRP, Serum amyloid A(SAA),together with imaging parameters such as the pulmonary artery-to-aorta diameter ratio, have emerged as potential predictors of exacerbations, yet their clinical utility is limited by variability and lack of specificity. In this review, we provide information regarding the importance of the early identification of exacerbation events in COPD patients and the available methods which can be used for this purpose. © 2025 by the authors.	https://www.scopus.com/inward/record.uri?eid=2-s	Q2	Scopus	EXC_PubType
Breaking down barriers to COPD management in primary care: a	2024	Chronic obstructive pulmonary disease (COPD) is a highly prevalent yet under-recognized and sub-optimally managed disease that is associated with substantial morbidity and mortality. Primary care providers (PCPs) are at the frontlines of COPD management, and they play a critical role across the full spectrum of the COPD patient journey from initial recognition and diagnosis to treatment optimization and referral to specialty care. The Canadian Thoracic Society (CTS) recently updated their guideline on pharmacotherapy in patients with stable COPD, and there are several key changes that have a direct impact on COPD management in the primary care setting. Notably, it is the first guideline to formally make recommendations on mortality reduction in COPD, which elevates this disease to the same league as other chronic diseases that are commonly managed in primary care and where optimized pharmacotherapy can reduce all-cause mortality. It also recommends earlier and more aggressive initial maintenance inhaler therapy across all severities of COPD, and preferentially favors the use of single inhaler therapies over multiple inhaler regimens. This review summarizes some of the key guideline changes and offers practical tips on how to implement the new recommendations in primary care. It also addresses other barriers to optimal COPD management in the primary care setting that are not addressed by the guideline update and suggests strategies on how they could be overcome. Copyright © 2024 Kaplan, Babineau, Hauptman, Levitz, Lin and Yang.	https://www.scopus.com/inward/record.uri?eid=2-s	Q2	Scopus	EXC_Scope
Using machine learning for early detection of chronic obstructive	2024	Chronic obstructive pulmonary disease (COPD) is a prevalent respiratory disease and ranks third in global mortality rates, imposing a significant burden on patients and society. This review looks at recent research, both domestically and abroad, on the application of machine learning (ML) for early COPD screening. The review discusses the practical application, key optimization points, and prospects of ML techniques in early COPD screening. The aim is to establish a scientific foundation and reference framework for future research and the development of screening strategies. © The Author(s) 2024.	https://www.scopus.com/inward/record.uri?eid=2-s	Q2	Scopus	EXC_PubType
Lung Transplantation	2024	[No abstract available]	https://www.scopus.com/inward/record.uri?eid=2-s	Q2	Scopus	EXC_PubType
Pulmonary Rehabilitation in Patients with Operable Non-Small C	2025	Lung cancer is the leading cause of cancer-related death worldwide, and patients with operable early-stage NSCLC are typically managed surgically. While effective, surgical resection can significantly impact pulmonary function and quality of life. Pulmonary rehabilitation (PR) is a comprehensive, multimodal approach that is an established cornerstone in the treatment of COPD. It has similarly demonstrated multiple benefits in patients with lung cancer who have undergone lobectomy or resection by improving pulmonary function, increasing exercise tolerance, improving nutritional status, providing psychological support, and enhancing quality of life. Despite this, PR for early-stage operable NSCLC is oftentimes not standardized, and challenges to adherence remain. In this review, we examine the components of PR, the role of PR in pre- and postoperative settings in patients with early-stage NSCLC, implementation strategies for PR, and future directions and challenges of PR in operable NSCLC. © 2025 by the authors.	https://www.scopus.com/inward/record.uri?eid=2-s	Q2	Scopus	EXC_Scope

Implementation of the Care Bundle for the Management of Chron	2024	Chronic obstructive pulmonary disease (COPD) is often part of a more complex cardiopulmonary disease, especially in older patients. The differential diagnosis of the acute exacerbation of COPD and/or heart failure (HF) in emergency settings is challenging due to their frequent coexistence and symptom overlap. Both conditions have a detrimental impact on each other's prognosis, leading to increased mortality rates. The timely diagnosis and treatment of COPD and coexisting factors like left ventricular overload or HF in inpatient and outpatient care can improve prognosis, quality of life, and long-term outcomes, helping to avoid exacerbations and hospitalization, which increase future exacerbation risk. This work aims to address existing gaps, providing management recommendations for COPD with/without HF, particularly when both conditions coexist. During virtual meetings, a panel of experts (the authors) discussed and reached a consensus on the differential and paired diagnosis of COPD and HF, providing suggestions for risk stratification, accurate diagnosis, and appropriate therapy for inpatients and outpatients. They emphasize that when COPD and HF are concomitant, both conditions should receive adequate treatment and that recommended HF treatments are not contraindicated in COPD and have favorable effects. Accurate diagnosis and therapy is crucial for effective treatment, reducing hospital readmissions and associated costs. The management considerations discussed in this study can potentially be extended to address other cardiopulmonary challenges frequently encountered by COPD patients. © 2024 by the authors.	https://www.scopus.com/inward/record.uri?eid=2-s	Q2	Scopus	EXC_Scope
Community Health Workers and Technology Interventions' Impac	2025	Background: Palliative care has the potential to relieve burdened global health systems but is in short supply in many low-resource settings. Community health workers (CHWs) and digital health tools/telephonic support have the potential to scale scarce palliative care resources and improve outcomes for seriously ill adults in home/community settings. Aim: To describe the utilization of CHWs and digital health/telephony in the palliative care of seriously ill adults in these settings. Design: We conducted a scoping review following the Preferred Reporting Items for Systematic Reviews and Meta-Analyses extension for scoping reviews guidelines. Search terms were developed with a health sciences librarian. Data Sources: The databases PubMed, EMBASE, LILACS, and CINAHL were searched for articles published from January 1, 2012, to December 30, 2023. Results: A total of 31 articles out of 7518 screened were included in the final analysis. Studies were mostly conducted in the United States. Most interventions were remote, with only four addressing rural or minority populations. Nineteen targeted advanced cancer, with others focusing on chronic obstructive pulmonary disease, heart failure, renal disease, and hospice care. CHWs and digital health/telephony were commonly used for physical and psychological care. Culturally tailored interventions with CHWs were few but effective. Patient quality of life, health care utilization, and caregiver outcomes were significantly impacted. Conclusions: CHWs and digital health/telephony can improve quality of life, health care use, and caregiver support. Most research focuses on physical and psychological aspects of care instead of cultural aspects of care. Future research is needed to explore culturally tailored interventions in minority populations and low- and middle-income countries, as well as investigate emerging remote technologies to allow for scaling palliative care into home/community settings. Copyright 2025, Mary Ann Liebert, Inc., publishers.	https://www.scopus.com/inward/record.uri?eid=2-s	Q2	Scopus	EXC_Scope
Diversity in pulmonary rehabilitation clinical trials: a systematic re	2024	Background: Underrepresentation of minority groups in clinical trials may hinder the potential benefits of pulmonary rehabilitation (PR) programs for individuals with chronic obstructive pulmonary disease (COPD). The aim of this work was to determine whether participants in PR randomized control trials (RCTs) conducted in the U.S.A., Canada, the UK, and Australia are representative of ethnicity, sex, gender, and sociodemographic characteristics. Research design: A systematic search was performed for relevant literature from inception to December 2022. Titles and abstracts were screened before undergoing a full article review. Relevant data on reporting of age, sex, gender, ethnicity, and sociodemographic characteristics of participants was extracted. Results: Thirty-six RCTs met the inclusion criteria. Only 6% of publications reported on ethnicity, with ≥90% of participants reported as 'White.' All 36 papers reported on age, with the mean between 60 and 69 years old. Thirty-five studies reported on sex (97%), with the majority (67%) reporting more male than female participants. There was no mention of different genders in any paper. Other sociodemographic factors were reported in 7 (19%) papers. Conclusions: Inclusivity and representation in clinical trials are essential to ensure that research findings are generalizable. Clinical trialists need to consider the demographics of today's society during recruitment. © 2024 Informa UK Limited, trading as Taylor & Francis Group.	https://www.scopus.com/inward/record.uri?eid=2-s	Q2	Scopus	EXC_Scope
Population risk stratification tools and interventions for chronic di	2025	Background: Population risk stratification (RS) tools have been proposed to tailor interventions, prioritize resources, and proactively manage high-risk individuals with chronic diseases in primary care settings. This study aims to explore the available evidence on the use of population RS tools in primary care settings, specifically evaluating the impact of targeted interventions based on RS tools on selected chronic patients and healthcare utilization outcomes. Methods: A systematic literature review was conducted across multiple electronic databases to identify relevant articles assessing the impact of targeted interventions based on RS tools in the management of chronic disease patients within primary care settings. We included studies meeting the following inclusion criteria: randomized controlled trials (RCTs), controlled clinical trials (CCTs) or before-after studies (BAs); adults with heart failure, chronic kidney disease, type 2 diabetes mellitus, chronic obstructive pulmonary disease, or dementia; interventions relying on RS tools; comparators with or without RS tools; and outcomes including Emergency Department (ED) visits, outpatient visits, hospitalizations, mortality, and costs. Results: A total of seven studies met the inclusion criteria, comprising one RCT, two CCTs, and four controlled BAs. The findings revealed mixed effects of interventions on patients identified using RS tools. Among the included studies, four reported significant reductions in ED visits. Two studies reported an increase in outpatient visits. Hospitalization rates were reduced in three studies, and two studies reported significant reductions in overall mortality. However, the impact on healthcare costs was inconclusive. Conclusions: The evidence on the effectiveness of RS tools for chronic disease management in primary care settings remains limited. While some studies demonstrated positive outcomes in reducing hospitalizations, ED visits, and mortality, the overall impact on outpatient service use and healthcare costs varied. Further high-quality studies are needed to evaluate the long-term benefits and cost-effectiveness of RS tools in chronic disease management within primary care. © The Author(s) 2025.	https://www.scopus.com/inward/record.uri?eid=2-s	Q2	Scopus	EXC_Scope
The effect of virtual reality technology in exercise and lung functi	2024	Background: Chronic obstructive pulmonary disease (COPD) is a serious chronic disease worldwide, with significant negative impacts on the quality of life, family economic burden, and social healthcare burden of patients. Aims: The aim of this study was to explore the effects of virtual reality technology on exercise function and lung function in COPD patients. Methods: A meta-analysis of randomized controlled trials was utilized. PubMed, Embase, Cochrane Library, Web of Science, PsycINFO, CINAHL, Medline, Scopus, China National Knowledge Infrastructure (CNKI), Wanfang Database, Weipu Database (VIP), and Chinese Biomedical Database (CBM) were systematically searched. We included randomized controlled trials published from the establishment of the database to August 10, 2022, on virtual reality technology in COPD patients. Literature retrieval and screening was carried out independently by two reviewers to obtain literature that met our inclusion and exclusion criteria and to extract relevant data. Two reviewers assessed the risk of bias in the included literature. A meta-analysis was performed using Revman 5.4 Software. Results: A total of 10 randomized controlled trials with 539 participants were included. The results showed that virtual reality technology significantly improved the lung function of COPD patients, such as forced expiratory volume (FEV1; MD = 7.29, 95% CI [4.34, 10.24], p <.01) and forced expiratory volume/forced vital capacity (FEV1/FVC; MD = 6.71, 95% CI [4.72, 8.71], p <.01). The combined intervention with different virtual reality technology had different effects on motor function. Compared with endurance training (ET) alone, virtual reality technology combined with ET had no significant effect on the 6-minute walk test (6WMT) in COPD patients (p >.05). Compared with pulmonary rehabilitation (PR) alone, virtual reality technology combined with PR was more effective in increasing 6WMT in COPD patients (MD = 30.80, 95% CI [10.85, 50.74], p <.01). Linking Evidence to Action: Virtual reality technology can help to improve lung function in COPD patients, and virtual reality combined with PR can improve exercise tolerance in COPD patients. However, due to the limited number of included studies, large-sample, multicenter, high-quality randomized controlled trial studies are needed to provide clear evidence. © 2024 Sigma Theta Tau International.	https://www.scopus.com/inward/record.uri?eid=2-s	Q2	Scopus	EXC_Scope
Adherence-enhancing interventions for pharmacological and oxy	2024	Introduction Adherence to COPD management strategies is complex, and it is unclear which intervention may enhance it. Objectives We aim to evaluate the effectiveness of adherence-enhancing interventions, alone or compared to interventions, for patients with COPD. Methods This review comprises a component network meta-analysis with a structured narrative synthesis. We searched MEDLINE, Embase, CENTRAL, CINAHL and trial registries on 9 September 2023. We included controlled studies that explored adherence in patients with COPD. Two review authors independently performed the study selection, data extraction and the risk of bias assessment. We involved patients with COPD in developing this systematic review through focus group interviews and displayed the findings in pre-designed logic models. Results We included 33 studies with 5775 participants. We included 13 studies in the component network meta-analysis that explored adherence. It was mainly assessed through questionnaires. As a continuous outcome, there was a tendency mainly for education (standardised mean difference 1.26, 95% CI 1.13–1.38, very low certainty of evidence) and motivation (mean difference 1.85, 95% CI 1.19–2.50, very low certainty of evidence) to improve adherence. As a dichotomous outcome (e.g. adherent/non-adherent), we found a possible benefit with education (odds ratio 4.77, 95% CI 2.25–10.14, low certainty of evidence) but not with the other components. We included six studies that reported quality of life in the component network meta-analysis. Again, we found a benefit of education (mean difference −9.70, 95% CI −10.82– −8.57, low certainty of evidence) but not with the other components. Conclusions Education may improve adherence and quality of life in COPD patients. Patient focus group interviews indicated that interventions that strengthen patients' self-efficacy and help them to achieve individual goals are the most helpful. © The authors 2024.	https://www.scopus.com/inward/record.uri?eid=2-s	Q2	Scopus	EXC_Scope

Application of e-Health tools in the assessment of inhalation ther	2025	Background: Chronic obstructive pulmonary disease (COPD) is a common respiratory disorder, and the assessment of inhalation therapy adherence is an important component of management in COPD patients. The emergence of e-Health tools provides new prospects for the assessment of inhalation therapy adherence. However, there is no comprehensive summary of the application of e-Health tools in assessing inhalation therapy adherence in COPD patients. Objectives: This review aims to clarify the current state, effects, benefits, and limitations of using e-Health tools in assessing inhalation therapy adherence in COPD patients and provide future directions and recommendations for development in this field. Methods: This scoping review follows the 5-step framework developed by Arksey and O'Malley. Literature on the practical application of e-Health tools was systematically searched from PubMed, Embase, Web of Science, CINAHL, and Cochrane Library, spanning from inception to April 2024. Additionally, VOSviewer (version 1.6.20) was used to construct visualization maps of countries, institutions, authors, and keywords to investigate the internal relations of included literature and to explore research hotspots. Results: A total of 26 studies were included. The e-Health tools mainly include electronic monitoring devices (EMDs), smartphone app, electronic prescription, and web-based tool. e-Health tools can assess inhalation therapy adherence in COPD patients in real-time and objectively, and improve inhalation therapy adherence and clinical outcomes. Bibliometric analysis indicates that there is no network of co-authorship between countries or academic organizations. Two collaborative networks have been formed centered on Greene G. EMDs and disease exacerbations are the popular research directions. Conclusions: e-Health tools have a wide range of applications and promising prospects in the assessment of inhalation therapy adherence in COPD patients. In the future, it is necessary to strengthen the cooperation between countries or research institutions, explore the cost-effectiveness of e-Health tools, and improve their accessibility and usability while adopting integrated design and combining artificial intelligence to improve the effectiveness of e-Health tools in the management of COPD. © 2024 Elsevier Ltd	https://www.scopus.com/inward/record.uri?eid=2-s	Q2	Scopus	EXC_PubType
What kind of non-pharmacological strategy for reducing sedentar	2024	Introduction: Chronic Obstructive Pulmonary Disease (COPD) is usually associated with sedentary behavior (SB). Literature reports a harmful impact of SB on the physical, mental, and social health of individuals with COPD. However, Pulmonary Rehabilitation (PR) programs seem to have no clear effect on changing SB. Therefore, our study aimed to identify the strategies used in the literature to reduce SB among individuals with COPD. Method: A scoping review was conducted to summarize the current literature regarding the proposed strategies to reduce SB in individuals with COPD. Searches were conducted in PUBMED; SCOPUS and COCHRANE LIBRARY for studies published from 2010 to march 2024. Results: Twenty four articles were retained for our review. Most of the identified strategies in the literature (21/24 articles) are based on behavioral approaches, with various forms: promoting self-efficacy, self-management and self-regulation of one's own behavior (12 articles), goal setting (10 articles), constant feedback (11 articles), therapeutic education (8 articles), motivational strategies (6 articles), re-engagement in meaningful activities (4 articles), promoting light intensity physical activity (LPA) (6 articles) and social support (6 articles). In association with the behavioral strategies, wearable connected technologies have been used in 4 articles, alone or combined with physical exercise programs included or not in a PR program. The home is associated with 83 % of interventions as a place for initial and continuous implementation of behavioral strategies. Conclusion: It would seem appropriate to focus on combined strategies to reduce SB in individuals with COPD (improvement of physical abilities and behavioral strategies). Further research is needed to only target the reduction of SB and to evaluate the effects of various interventions. © 2024 The Authors	https://www.scopus.com/inward/record.uri?eid=2-s	Q2	Scopus	EXC_Scope
Chronic Obstructive Lung Disease: Treatment Guidelines and Re	2024	Chronic Obstructive Pulmonary Disease (COPD) constitutes a major public health problem, and it is anticipated that its prevalence will continue to increase in the future. Its progressive nature requires a continuous and well-coordinated care approach. The follow-up for COPD should primarily focus on disease screening and control, which includes monitoring of pulmonary function, prevention of exacerbations, identification of aggravating factors and comorbidities, as well as ensuring treatment adequacy and adherence. However, existing clinical practice guidelines and consensus documents offer limited recommendations for the follow-up. In this context, we undertake a review of COPD treatment and the continuity of care recommendations endorsed by several scientific societies. Moreover, we underscore the importance of the involvement of nursing and community pharmacy in this process, as well as the utilization of quality indicators in the provision of care for the disease. © 2024 by the authors.	https://www.scopus.com/inward/record.uri?eid=2-s	Q2	Scopus	EXC_Scope
Effectiveness of gamified exercise programs on the level of phys	2024	Purpose: to assess the effects of supervised and unsupervised gamified exercise programs on physical activity level, sedentary behavior and quality of life in patients with non-communicable chronic diseases. Materials and methods: Six database were searched. Methodological quality of included studies, the quality of reporting interventions and the quality of the applications were assessed using the PEDro, TIDieR and MARS, respectively. Results: Nine studies were included (n = 974; 60.2 ± 5.7 years). Three of them enrolled individuals with cancer, one with stroke, one with multiple sclerosis, one with COPD, two with Diabetes Mellitus, and one with knee and hip osteoarthritis. Gamification was performed via a smartphone application in three studies (MARS = 13.4 ± 9.75pts, ranging from 10.9 to 16.9pts). The intervention was supervised in six studies. The PEDro and TIDIeR scores were 5.5 ± 1.3 (ranging 0–8pts) and 16.11 ± 3.14 (ranging 10–20pts), respectively. Supervised gamified interventions increased the level of physical activity compared to usual supervised exercises. Quality of life was similar between groups. Unsupervised interventions were similar for all outcomes evaluated. Conclusions: Supervised gamified exercise programs seem to increase the level of physical activity compared to usual exercises in patients with chronic diseases. However, studies with better methodological qualities and subgroup analyzes are needed. © 2024 Informa UK Limited, trading as Taylor & Francis Group.	https://www.scopus.com/inward/record.uri?eid=2-s	Q2	Scopus	EXC_Scope
Overcoming challenges of managing chronic obstructive pulmona	2024	Introduction: Chronic obstructive pulmonary disease (COPD) ranks among the top three global causes of death, with 90% of fatalities concentrated in low- and middle-income countries (LMICs). The projected rise in COPD burden, especially in LMICs, emphasizes the need to address the challenges for effective control and reversal of this trend. We aimed to provide an overview, and propose potential solutions to these challenges. Areas covered: We highlight the challenges faced in managing COPD in LMICs and put forward the potential approaches to mitigate the same. Expert opinion: In LMICs, the effective management of COPD encounters numerous barriers. These include limited access to critical diagnostic services, inadequately trained healthcare personnel, shortages of inhaler medications, oxygen therapy, insufficient access to vaccines, and pulmonary rehabilitation programs. Compounding the above challenges is the late presentation due to misdiagnosis by health workers, and limited access to vital diagnostics. Moreover, the pharmacological armamentarium for optimal COPD therapy, notably inhaled therapies, face constraints in both access and affordability. We propose multi-level and multifaceted interventions to address the urgent need for enhanced respiratory care, human resource capacity building, relevant diagnostic approaches, increased access to medications, government, regional and global efforts to achieve optimal COPD management in LMICs. © 2024 The Author(s). Published by Informa UK Limited, trading as Taylor & Francis Group.	https://www.scopus.com/inward/record.uri?eid=2-s	Q2	Scopus	EXC_Scope
Machine learning-based methods for detecting respiratory abnor	2025	Respiratory abnormalities pose a significant health burden, often demanding timely and accurate diagnosis. Traditional methods have limitations, prompting exploration of novel approaches. This review explores the potential of machine learning, leveraging both audio and visual analysis, for detecting respiratory abnormalities. We explore various methods employed, analyzing audio features like MFCCs and spectral energy, and exploring diverse visual features like chest wall motion and depth maps. Different machine learning techniques, including CNNs and RNNs, are discussed, highlighting their applications in detecting specific conditions like asthma and COPD. We systematically evaluate their performance, analyzing strengths and limitations of both audio-based and visual-based approaches. Further, we explore the potential of multimodal analysis, combining audio and visual information for enhanced performance. Reviewing existing studies, we assess the advantages gained over unimodal methods. Finally, we explore the clinical implications and future directions, discussing potential applications like remote monitoring and early diagnosis, while acknowledging remaining challenges and ethical considerations. By comprehensively examining the landscape of machine learning for audio and visual analysis in respiratory abnormality detection, this review offers valuable insights for researchers and clinicians, ultimately accelerating the translation of these promising methods into real-world practice. © 2025	https://www.scopus.com/inward/record.uri?eid=2-s	Q2	Scopus	EXC_Scope

Identifying Concepts of Physical Activity Which Are Clinically Mea	2025	Physical activity (PA) is indispensable for overall health. Sub-optimal PA is linked to reduced quality of life (QOL) and premature death. In clinical research and therapeutics development, defining aspects of PA that are meaningful to patients and care providers is essential for designing tailored interventions, identifying individual contextual factors, and enhancing patient satisfaction and engagement in their own well-being. As digital health technologies (DHTs) measuring PA rapidly evolve, there is an opportunity to further define concepts. A systematic review of qualitative studies to identify concepts of PA that are meaningful to patients and care providers was conducted. Conditions covered included Parkinson's disease, multiple sclerosis, chronic obstructive pulmonary disease, cancer, Duchenne muscular dystrophy, chronic heart failure, sickle cell disease, osteoarthritis, and sarcopenia. We analyzed studies published in the last 20 years utilizing qualitative or mixed methods techniques to describe aspects of PA that patients want to prevent from worsening or improve. Among the 5228 articles returned, 105 studies were included. Thematic synthesis revealed five meaningful aspects of health (MAH) related to PA: ambulation-dependent activities, balance-dependent activities, activities needing upper limb function, changing body positions, and participating in activities of different intensities. Patients also reported PA as important to QOL and influenced by internal and external facilitators and barriers. This research presents new findings related to PA MAHs across various therapeutic areas, which go beyond walking. The findings provide a foundation for defining concepts of interest, measures, and endpoints, with applications in clinical research and care, including patient-focused development of digitally derived measures. © 2025 The Author(s). Clinical and Translational Science published by Wiley Periodicals LLC on behalf of American Society for Clinical Pharmacology and Therapeutics.	https://www.scopus.com/inward/record.uri?eid=2-s	Q2	Scopus	EXC_Scope
Efficacy and Safety of Acupuncture in Managing COPD: An Over	2024	Background: Acupuncture has been used as an adjuvant therapy for Chronic obstructive pulmonary disease (COPD). However, systematic reviews (SRs) and meta-analyses (MAs) have reported inconsistent results and unknown quality. This overview aimed to summarize the current SRs/MAs to provide evidence for the effectiveness and safety of acupuncture in the treatment of COPD. Methods: SRs/MAs were searched via eight databases from their establishment to December 31, 2023. The methodological quality was assessed by A Measurement Tool to Assess Systematic Reviews 2 (AMSTAR 2). The risk of bias was assessed using the Risk of Bias in Systematic Review (ROBIS) tool. The Preferred Reporting Items for Systematic Reviews and Meta-analyses for Acupuncture (PRISMA-A) to evaluate the reporting quality. The Grading of Recommendations Assessment, Development and Evaluation (GRADE) was used to determine the strength of evidence. In addition, we also conducted an analysis of the acupuncture points used in the primary RCTs. Results: Twenty-two SRs/MAs were included in this overview. Based on the assessment using AMSTAR 2, nineteen SRs/MAs were “critically low”. Eight SRs/MAs had a low risk of bias. Based on PRISMA-A, the reporting completeness of eighteen SRs/MAs were more than 70%. As for GRADE assessment, only three outcome measures were of high quality. COPD patients can benefit from moxibustion, acupoint application, acupoint catgut embedding, manual acupuncture, and electroacupuncture, as indicated by effectiveness in measures including lung function, 6MWD, mMRC, CAT, and acute exacerbation. In addition, the efficacy of TENS needed to be further demonstrated. The commonly used acupuncture points in the RCTs include BL13, BL23, and EX-B1. Conclusion: Evidence from SRs showed that acupuncture is beneficial to lung function, acute exacerbation, 6MWD, mMRC and CAT. For SGRQ and brog scale, acupuncture should be used selectively, but this finding should still be taken with caution. © 2024 Zeng et al.	https://www.scopus.com/inward/record.uri?eid=2-s	Q2	Scopus	EXC_Scope
Increasing exercise capacity and physical activity in the COPD p	2024	Higher levels of exercise capacity and physical activity are desired outcomes in the comprehensive management of the COPD patient. In addition, improvements in exercise capacity and physical activity are instrumental to optimising other important therapeutic goals, such as improved health status, reduced healthcare utilisation and increased survival. Four general approaches towards increasing exercise capacity and physical activity in individuals with COPD will be discussed in this review: 1) pharmacological intervention, especially the administration of long-acting bronchodilators; 2) pulmonary rehabilitation, including exercise training and collaborative self-management; 3) behavioural interventions; and 4) web-based interventions. These are by no means the only approaches, nor are they mutually exclusive: indeed, combining them, as necessary, to meet the needs of the individual respiratory patient may promote optimal outcomes, although further research is necessary in this area. © ERS 2024.	https://www.scopus.com/inward/record.uri?eid=2-s	Q2	Scopus	EXC_Scope
POTENTIAL DRUG DELIVERY SYSTEMS AND DEVICE COMB	2025	Chronic Obstructive Pulmonary Disease (COPD) presents with chronic lung inflammation and poorly reversible airflow limitation, necessitating bronchodilators for management. The Global Initiative for COPD recommends combining Long-Acting Beta-Agonists (LABAs) and Long-Acting Antimuscarinic Agents (LAMAs) for most COPD patients. Developing fixed LAMA/lABA combinations is crucial. Adding an Inhaled Corticosteroid (ICS) to this combination may offer additional benefits, including preventing exacerbations. The GOLD report emphasizes diagnosis, prevention, exacerbation management, and addressing comorbidities. It advocates for holistic COPD management, integrating pharmacologic and non-pharmacologic approaches. Novel strategies like mono, dual, and triple therapies are recommended. The review highlights COPD's impact on COVID-19, comorbidities, and relevant patents concerning COPD and bronchodilators. The bronchodilator treatments may improve their efficacy in this critical aspect of COPD. Research shows that dual bronchodilation improves lung function and symptoms more consistently than mono-bronchodilation while potentially lowering the risk of exacerbations and disease deterioration and having a similar safety profile. © 2025 The Authors. Published by Innovare Academic Sciences Pvt Ltd.	https://www.scopus.com/inward/record.uri?eid=2-s	Q2	Scopus	EXC_Scope
Effectiveness of self-management digital interventions in improvi	2025	Objective: This review will aim to assess the effectiveness of self-management digital interventions in improving health-related outcomes in patients with chronic obstructive pulmonary disease (COPD). Introduction: Respiratory rehabilitation programs that promote self-management are crucial for COPD patients. However, current face-to-face strategies involve challenges, such as low participation, high dropout rates, and short-lived post-intervention benefits. Digital self-management interventions may address these issues by improving access to support and enhancing health-related outcomes. Several systematic reviews have examined the effectiveness of these interventions in improving health-related outcomes, prompting the current umbrella review to summarize the existing evidence. Inclusion criteria: This review will consider systematic reviews that examine the effectiveness of self-management digital interventions in improving health-related outcomes in COPD patients. It will not be restricted by COPD stage, phase, comorbidity, sex or gender, setting, or context. Methods: The JBI methodology for umbrella reviews will be followed. A comprehensive search will be conducted in PubMed, Embase, Cochrane Database of Systematic Reviews, CINAHL, PEDro, the International Network of Agencies for Health Technology Assessment, Epistemonikos, Web of Science, ProQuest, and COPD-related websites to identify reviews published in any language from inception to date. Titles and abstracts, and then full texts, will be screened independently by 2 reviewers against the inclusion criteria. Methodological quality will be assessed using the JBI critical appraisal checklist for systematic reviews and research syntheses. Data will be extracted using an adapted and piloted JBI data extraction tool. The findings will be presented in tabular format, along with narrative descriptions and visual support. A citation matrix will be produced to assess study overlap. Umbrella review registration: PROSPERO CRD42024517476. © 2025 JBI.	https://www.scopus.com/inward/record.uri?eid=2-s	Q2	Scopus	EXC_PubType
Assessing the comparative effects of interventions in COPD: a tu	2024	To optimize patient outcomes, healthcare decisions should be based on the most up-to-date high-quality evidence. Randomized controlled trials (RCTs) are vital for demonstrating the efficacy of interventions; however, information on how an intervention compares to already available treatments and/or fits into treatment algorithms is sometimes limited. Although different therapeutic classes are available for the treatment of chronic obstructive pulmonary disease (COPD), assessing the relative efficacy of these treatments is challenging. Synthesizing evidence from multiple RCTs via meta-analysis can help provide a comprehensive assessment of all available evidence and a “global summary” of findings. Pairwise meta-analysis is a well-established method that can be used if two treatments have previously been examined in head-to-head clinical trials. However, for some comparisons, no head-to-head studies are available, for example the efficacy of single-inhaler triple therapies for the treatment of COPD. In such cases, network meta-analysis (NMA) can be used, to indirectly compare treatments by assessing their effects relative to a common comparator using data from multiple studies. However, incorrect choice or application of methods can hinder interpretation of findings or lead to invalid summary estimates. As such, the use of the GRADE reporting framework is an essential step to assess the certainty of the evidence. With an increasing reliance on NMAs to inform clinical decisions, it is now particularly important that healthcare professionals understand the appropriate usage of different methods of NMA and critically appraise published evidence when informing their clinical decisions. This review provides an overview of NMA as a method for evidence synthesis within the field of COPD pharmacotherapy. We discuss key considerations when conducting an NMA and interpreting NMA outputs, and provide guidance on the most appropriate methodology for the data available and potential implications of the incorrect application of methods. We conclude with a simple illustrative example of NMA methodologies using simulated data, demonstrating that when applied correctly, the outcome of the analysis should be similar regardless of the methodology chosen. © The Author (s) 2024.	https://www.scopus.com/inward/record.uri?eid=2-s	Q2	Scopus	EXC_Scope

THE URGENT NEED TO INCREASE THE PORTFOLIO OF REH	2024	Pulmonary rehabilitation (PR) plays a crucial role in improving outcomes for individuals with chronic respiratory diseases. The most outstanding challenge in PR is the low referral, uptake, and adherence that is dramatically low in the post-hospitalization period in rural, minority, and low-income populations. Research efforts to increase the portfolio of PR through the testing and implementation of new and effective options for home-based and community-based programs are potential research targets. Other potential foci of research efforts are the pursuit of behavior-change techniques to achieve a lifestyle change during PR and the referral process to increase uptake and adherence rates. Creating new rehabilitation options for chronic lung disease that can reach more people may require expanding the definition of PR for chronic lung disease to one more flexible and perhaps concordant with the World Health Organization's definition of rehabilitation as "a set of interventions designed to optimize functioning and reduce disability in individuals with health conditions in interaction with their environment." While efforts to augment access to conventional PR are warranted, it is time to devote research efforts to reach many more individuals with chronic lung disease, particularly minorities, rural, and low-income individuals that currently have no access to any rehabilitation, a social injustice that requires action. This manuscript outlines suggested future directions in rehabilitation research with the pursuit of evidence to support feasible and effective programs that may increase the rehabilitation portfolio to accommodate most individuals with chronic lung disease. Copyright © 2024 Wolters Kluwer Health, Inc. All rights reserved.	https://www.scopus.com/inward/record.uri?eid=2-s	Q2	Scopus	EXC_Scope
Optimizing Pulmonary Rehabilitation in Saudi Arabia: Current Pra	2025	Chronic respiratory diseases (CRDs) are a significantly major cause of mortality in Saudi Arabia, with their progression frequently involving comorbidities and exacerbations that extend beyond the lungs. This review considers the current state of pulmonary rehabilitation (PR) in Saudi Arabia, this being a well-known non-pharmacological intervention to help control and reduce the burden of CRDs, highlighting the intervention's availability, multidisciplinary approach, and integration within the healthcare system, as well as examining the diseases' contribution to overall symptom severity, impairing daily activities and significantly worsening the patient's quality of life. Although PR is strongly recommended for managing CRDs, its utilization in Saudi Arabia remains limited or unavailable in many regions. Key barriers to PR access include inadequate awareness among healthcare providers and patients, logistical challenges, and an insufficient number of specialized facilities and trained professionals. Expanding PR programs in Saudi Arabia requires addressing geographical barriers, ensuring adequate space, resources, and trained personnel, and raising awareness among healthcare providers through education and training. Integrating PR principles into medical education and offering incentives for specialization can help overcome personnel shortages. Additionally, promoting telerehabilitation can enhance patient compliance and ensure the long-term success of PR programs. These initiatives aim to optimize PR services and improve patient outcomes across the nation. © 2025 by the author.	https://www.scopus.com/inward/record.uri?eid=2-s	Q2	Scopus	EXC_Scope
Effectiveness of virtual reality-based therapy in pulmonary rehab	2024	Background: In addition to conventional pulmonary rehabilitation (PR) programs for the treatment of chronic obstructive pulmonary disease (COPD), the use of virtual reality-based therapy (VRBT) has been proposed as an effective complementary tool to be included in PR programs for COPD. Objectives: To analyze the effectiveness of VRBT on functional capacity, pulmonary function, and functional mobility in patients with COPD. Methods: A meta-analysis was carried out through a bibliographic search in PubMed (Medline), WOS, PEDro, CINAHL, CENTRAL, and Scopus since inception up to June 2023. The risk of bias was assessed using the PEDro scale, and the effect was determined using the standardized mean difference (SMD) and its 95 % confidence interval (95 % CI) in a random effects model. Results: Five RCTs, providing data from 344 participants with a mean age 65.7 ± 5.3 years old, were included. The mean methodological quality of the studies included was good (6.8 ± 1.6 points). The meta-analysis showed that VRBT was effective in increasing functional capacity, assessed with the 6 Min Walking Test, (SMD=0.4, 95 % CI 0.07 to 0.71, p = 0.017); pulmonary function, assessed with FEV1 (SMD=0.33, 95 %CI 0.01 to 0.65, p = 0.048); and functional mobility, assessed with the Get Up and Go Test (SMD=0.77, 95 % CI 0.5 to 1.1, p<0.001) in patients with COPD. Conclusion: VRBT is suggested to be effective in increasing functional capacity, pulmonary function, and functional mobility in patients with COPD. Non-immersive VRBT is the most used modality of VRBT in PR. © 2024 The Author(s)	https://www.scopus.com/inward/record.uri?eid=2-s	Q2	Scopus	EXC_Scope
Everyday technology and assistive technology supporting everyd	2024	Introduction: People living with chronic obstructive pulmonary disease (COPD) encounter challenges in everyday life activities due to symptoms like breathlessness and fatigue. Compensatory strategies, such as using everyday technology (mechanical, electronic and digital equipment and functions encountered daily) and assistive technology (products, instruments, or equipment adapted or designed to improve functioning of people with disabilities), are crucial for supporting everyday life activities; thus, it is essential to explore therapeutic potentials of these technologies. The present review aims to synthesise research literature concerning the use of everyday technology and assistive technology to support everyday activities among persons living with COPD. Methods: A narrative review was conducted with a systematic search in five bibliographic databases. Three sets of search terms were used: (i) everyday technology, assistive technology, and related terms, (ii) everyday life activities and related terms, and (iii) chronic obstructive pulmonary disease and related terms. Results: Screening resulted in 26 included articles.Following the American Occupational Therapy Association framework, the identified articles show six categories of everyday life activities supported by everyday technologies and assistive technologies: health management, social participation, activities of daily living, instrumental activities of daily living, leisure, and rest and sleep. Conclusion: Most articles focus on everyday technology for health management; however, everyday technology may hold unexpected potential to support a broader array of everyday life activities. Little is known about assistive technology to support everyday life activities for people with COPD, though it is described as crucial for independence and energy conservation. © 2024 The Author(s). Published by Informa UK Limited, trading as Taylor & Francis Group.	https://www.scopus.com/inward/record.uri?eid=2-s	Q2	Scopus	EXC_PubType
Using Virtual Reality to Improve Outcomes Related to Quality of	2025	Background: Virtual reality (VR) has promise as an innovative nonpharmacologic treatment for improving a patient's quality of life. VR can be used as an adjunct or treatment for many acute and chronic conditions, including serious illnesses. Objective: This systematic review aims to assess the current state of the literature of randomized controlled trials that use VR in patients with serious illnesses. Two secondary aims include assessing intervention components associated with improved quality of life and functional outcomes among older adults, as well as evaluating how well the randomized controlled trials adhere to consensus standards for VR research. Methods: We searched PubMed, Embase, and CINAHL for randomized controlled studies published at any time. We screened and accepted studies that reported outcomes related to patients' quality of life, provided an immersive VR intervention, and included patients with serious illness. We narratively summarized key attributes of publications that shed light on study efficacy, generalizability, replicability, and clinical utility. All studies were assessed for study quality with the Cochrane Risk of Bias tool and for concordance with 8 recent consensus standards for VR research. Results: From the 12,621 articles searched in May 2024, a total of 24 (0.19%) studies met the inclusion criteria, and of these, 88% (21/24) reported an improvement in at least 1 patient quality of life outcome and 67% (16/24) had a high risk of bias. In 7 (n=24, 29%) studies, VR was used to provide distraction therapy to reduce pain. In total, 5 (n=24, 21%) studies included training, supervision, and assistance in VR use, which demonstrated improvements in patient quality of life–related outcomes. Of 24 studies, 9 (38%) included patients with stroke, 9 (38%) included patients with cancer, 4 (17%) included patients with cardiovascular disease, 1 (4%) included patients with chronic obstructive pulmonary disease, and 1 (4%) included patients who reported pain in hospital. In all 9 studies that included patients with stroke, the main purpose of VR was to improve mobility and strength; these studies had higher frequency and longer durations of VR use, ranging from 2 to 9 weeks, as compared to a VR use duration of <2 weeks for studies aiming to reduce pain or anxiety. Regarding consensus standards for VR research, 29% (7/24) of the studies adhered to all 8 criteria, and all studies (24/24, 100%) adhered to ≥5 criteria. Conclusions: Nascent evidence suggests VR's potential in mitigating pain, anxiety, and depression and improving mobility among persons with serious illnesses. Most studies did not provide detailed information about unassisted or assisted use, suggesting that VR for older adults is currently most appropriate for observed settings with assistance available. Trial Registration: PROSPERO CRD42022346178; https://www.crd.york.ac.uk/prospero/display_record.php?RecordID=346178 ©Bhagvat Maheta, Alexandra Kraft, Nickolas Interrante, Soraya Fereydooni, Jeremy Bailenson, Brian Beams, Christina Keny, Thomas Osborne, Karleen Giannitrapani, Karl Lorenz.	https://www.scopus.com/inward/record.uri?eid=2-s	Q2	Scopus	EXC_Scope
Understanding the Gaps in the Reporting of COPD Exacerbation	2024	Exacerbations of chronic obstructive pulmonary disease (COPD) are associated with loss of lung function, poor quality of life, loss of exercise capacity, risk of serious cardiovascular events, hospitalization, and death. However, patients underreport exacerbations, and evidence suggests that unreported exacerbations have similar negative health implications for patients as those that are reported. Whilst there is guidance for physicians to identify patients who are at risk of exacerbations, they do not help patients recognise and report them. Newly developed tools, such as the COPD Exacerbation Recognition Tool (CERT) have been designed to achieve this objective. This review focuses on the underreporting of COPD exacerbations by patients, the factors associated with this, the consequences of underreporting, and potential solutions. © 2024 The Author(s). Published with license by Taylor & Francis Group, LLC.	https://www.scopus.com/inward/record.uri?eid=2-s	Q2	Scopus	EXC_Scope

Nurse-Led/Involved Home-Based Interventions for Older Adults	2025	Aims: To determine the effectiveness of nurse-led/involved home-based interventions for older people with COPD and to explore the experiences of older people and nurses with the interventions. Design: A mixed-methods systematic review following the JBI methodology for mixed-methods systematic reviews. Data Sources: The search included relevant and peer-reviewed studies published from January 2010 to December 2023 in CINAHL, MEDLINE, Cochrane Central Register of Controlled Trials, PsycINFO, EMBASE, JBI, EMCARE and ProQuest. Review Methods: English-language reports of nurse-led/involved home-based interventions for people with COPD were included based on authors' consensus. Three reviewers performed independent quality appraisal using JBI tools. A convergent segregated approach was used for data synthesis and integration. Results: Seven interventions were identified in two mixed-methods, two qualitative, two quasi-experimental studies, and one secondary analysis from a randomised control trial. The effectiveness of the interventions was measured with various outcomes and was effective to some extent, with reduced hospitalisation, hospitalisation days, hospitalisation cost and all-paid claims. However, the outcomes were not statistically significant, and the effectiveness was inconclusive. While patients appreciated support and resources, some perceived them as a double-edged sword. Conclusions: Patients preferred more holistic interventions over extended periods. The inconclusive findings and limitations warrant further research with larger sample sizes and comparable measurement tools and outcomes. Impact: This is the first mixed-methods systematic review on the effectiveness of home interventions for people with COPD with a clear definition of 'nurse-led'. Nurses felt highly valued by patients and other health professionals; however, they reported a lack of support from management. The lack of interventions led by nurses challenges them to lead, deliver and evaluate what matters to people with COPD. Reporting Method: This systematic review was reported in accordance with the Referred Reporting Items for Systematic Reviews and Meta-Analyses (PRISMA) guidelines. Patient or Public Contribution: Not applicable. © 2025 John Wiley & Sons Ltd.	https://www.scopus.com/inward/record.uri?eid=2-s	Q2	Scopus	EXC_Scope
The effects of green exercise on the mental and physical health	2025	Green exercise, defined as physical activity in natural settings, shows promise for enhancing exercise participation and improving health. This systematic review aimed to assess the effectiveness of green exercise in people with chronic conditions. Seven electronic databases were searched and of the 7801 screened articles, 14 trials met the inclusion criteria. Green exercise was a safe and well-tolerated intervention, with low drop-out levels. It was found to positively affect participants' quality of life in three studies and mental health in four studies. Compared to non-exercise groups, green exercise significantly improved physical and mental health in patients with breast cancer, COPD, cardiovascular disease risk, chronic low back pain, obesity, and diabetes. However, it had no impact on the physical health of stroke patients or the cognitive performance of those with ADHD. Green exercise appears to be a safe intervention that can improve various chronic health issues. © 2024 Informa UK Limited, trading as Taylor & Francis Group.	https://www.scopus.com/inward/record.uri?eid=2-s	Q2	Scopus	EXC_Scope
Remote digital monitoring for selected chronic diseases in primar	2023	Objectives: This is a protocol for a Cochrane Review (intervention). The objectives are as follows:. To assess the benefits and harms of remote digital monitoring for adults with selected chronic diseases (hypertension, type 2 diabetes, chronic obstructive pulmonary disease, congestive heart failure, and asthma) in primary healthcare settings. Copyright © 2023 The Cochrane Collaboration. Published by John Wiley & Sons, Ltd.	https://www.scopus.com/inward/record.uri?eid=2-s	Q2	Scopus	EXC_Scope
Comparison of pulmonary rehabilitation and chest physiotherapy	2023	INTRODUCTION: Chronic obstructive pulmonary disease (COPD) has become the third foremost cause of mortality by 2020. Due to the high incidence rate of COPD among the population, it has become a major social and health burden. This meta-analysis was designed to appraise the long-term effect of the pulmonary rehabilitation (PR) program among elder COPD patients in terms of improvements in exercise capacity and quality of life. EVIDENCE ACQUISITION: Research papers were extracted from different databases including MEDLINE, Scopus, EMBASE, PubMed, ScienceDirect, and Resources Information Center. For data extraction, MeSH keywords of "Pulmonary Rehabilitation (PR)" "COPD elder patients" "Short term outcomes" "long-term outcomes" "improvements in quality of life" and "reduced physical activity" were used. The time duration of the study was limited from January 2010 to April 2022. PRISMA standards were followed in the meta-analysis. EVIDENCE SYNTHESIS: Approximately, 1852 elder COPD patients (66.8±7.8 years) were examined from 18 randomized control trials (RCT)-based studies. The standard mean difference of 7 RCT studies of Six-Minute Walk Test (6MWT) Score was 0.54 (0.39-0.70) at I2=71%, CI=95%, Z=6.83, (P<0.00001). Difference between the PR and without PR groups showed slight improvements in the 6MWT Score. The CRQ Score assessed through the questionnaire showed improvements from baseline values after receiving the PR program. The standard deviation measured was 0.30 (0.54-0.5) CI=95%, Z=2.36. CONCLUSIONS: There was a significant difference in 6MWT value when measured among COPD patients receiving PR as compared to patients without PR. PR has a significant role in the improvement of exercise capacity and quality of life. This intervention should be sustained long duration to avoid asthmatic attacks and airflow blockage. © The Authors 2023.	https://www.scopus.com/inward/record.uri?eid=2-s	Q2	Scopus	EXC_Scope
The Use of eHealth for Pharmacotherapy Management With Pati	2023	Background: eHealth is increasingly considered an important tool for supporting pharmacotherapy management. Objective: We aimed to assess the (1) use of eHealth in pharmacotherapy management with patients with asthma or chronic obstructive pulmonary disease (COPD), diabetes, or cardiovascular disease (CVD); (2) effectiveness of these interventions on pharmacotherapy management and clinical outcomes; and (3) key factors contributing to the success of eHealth interventions for pharmacotherapy management. Methods: We conducted a scoping review following the PRISMA-ScR (Preferred Reporting Items for Systematic Reviews and Meta-Analyses extension for scoping review) statement. Databases searched included Embase, MEDLINE (PubMed), and Cochrane Library. Screening was conducted by 2 independent researchers. Eligible articles were randomized controlled trials and cohort studies assessing the effect of an eHealth intervention for pharmacotherapy management compared with usual care on pharmacotherapy management or clinical outcomes in patients with asthma or COPD, CVD, or diabetes. The interventions were categorized by the type of device, pharmacotherapy management, mode of delivery, features, and domains described in the conceptual model for eHealth by Shaw at al (Health in our Hands, Interacting for Health, Data Enabling Health). The effectiveness on pharmacotherapy management outcomes and patient- and clinician-reported clinical outcomes was analyzed per type of intervention categorized by number of domains and features to identify trends. Results: Of 63 studies, 16 (25%), 31 (49%), 13 (21%), and 3 (5%) included patients with asthma or COPD, CVD, diabetes, or CVD and diabetes, respectively. Most (38/63, 60%) interventions targeted improving medication adherence, often combined for treatment plan optimization. Of the 16 asthma or COPD interventions, 6 aimed to improve inhaled medication use. The majority (48/63, 76%) of the studies provided an option for patient feedback. Most (20/63, 32%) eHealth interventions combined all 3 domains by Shaw et al, while 25% (16/63) combined Interacting for Health with Data Enabling Health. Two-thirds (42/63, 67%) of the studies showed a positive overall effect. Respectively, 48% (23/48), 57% (28/49), and 39% (12/31) reported a positive effect on pharmacotherapy management and clinician- and patient-reported clinical outcomes. Pharmacotherapy management and patient-reported clinical outcomes, but not clinician-reported clinical outcomes, were more often positive in interventions with ≥3 features. There was a trend toward more studies reporting a positive effect on all 3 outcomes with more domains by Shaw et al. Of the studies with interventions providing patient feedback, more showed a positive clinical outcome, compared with studies with interventions without feedback. This effect was not seen for pharmacotherapy management outcomes. Conclusions: There is a wide variety of eHealth interventions combining various domains and features to target pharmacotherapy management in asthma or COPD, CVD, and diabetes. Results suggest feedback is key for a positive effect on clinician-reported clinical outcomes. eHealth interventions become more impactful when combining domains. © 2023 Journal of Medical Internet Research. All rights reserved.	https://www.scopus.com/inward/record.uri?eid=2-s	Q2	Scopus	EXC_Scope
Healthcare experiences of adults with COPD across community	2023	Background Studies investigating lived experiences of patients with COPD raise important concerns about interactions with healthcare professionals. Patients often describe feelings of guilt and shame associated with their COPD and may experience stigma and poor patient experience of care. The aims and objectives of the present study were to systematically scope and synthesise findings from peer-reviewed qualitative studies describing healthcare experiences of patients living with COPD across community care settings. Methods A meta-ethnography was undertaken. Database searches were performed in Ovid MEDLINE, PsychINFO, Ovid Emcare, CINAHL Plus and Sociological Abstracts. Eligible qualitative studies were included. Study screening and data extraction was performed by two independent reviewers. A "line-of-argument" synthesis and deductive and inductive analysis was used to identify key themes, where the deductive element aligned to Wong and Haggerty's six key dimensions of patient experiences. Results Data from 23 studies were included. Experiences and their meaning to patients were explored within the context of six domains of patient experience including access, interpersonal communication, continuity and coordination, comprehensiveness and trust. Inductive coding revealed emotion, stigma, identity and vulnerability shaped healthcare experiences of adults with COPD. Implications Experiences often fell short of what was expected and needed in community settings. Adopting strategies to improve experiences of care in the community can be expected to improve self-management and contribute to improved health outcomes and quality of life. These strategies should take account of vulnerability, stigma and emotions such as guilt and blame that are potent affective drivers of the experience of care for patients with COPD. © The authors 2023.	https://www.scopus.com/inward/record.uri?eid=2-s	Q2	Scopus	EXC_PubType

Digital healthcare in COPD management: a narrative review on t	2022	Chronic obstructive pulmonary disease (COPD) remains a leading cause of morbidity and mortality despite current treatment strategies which focus on smoking cessation, pulmonary rehabilitation, and symptomatic relief. A focus of COPD care is to encourage self-management, particularly during COVID-19, where much face-to-face care has been reduced or ceased. Digital health solutions may offer affordable and scalable solutions to support COPD patient education and self-management, such solutions could improve clinical outcomes and expand service reach for limited additional cost. However, optimal ways to deliver digital medicine are still in development, and there are a number of important considerations for clinicians, commissioners, and patients to ensure successful implementation of digitally augmented care. In this narrative review, we discuss advantages, pitfalls, and future prospects of digital healthcare, which offer a variety of tools including self-management plans, education videos, inhaler training videos, feedback to patients and healthcare professionals (HCPs), exacerbation monitoring, and pulmonary rehabilitation. We discuss the key issues with sustaining patient and HCP engagement and limiting attrition of use, interoperability with devices, integration into healthcare systems, and ensuring inclusivity and accessibility. We explore the essential areas of research beyond determining safety and efficacy to understand the acceptability of digital healthcare solutions to patients, clinicians, and healthcare systems, and hence ways to improve this and sustain engagement. Finally, we explore the regulatory challenges to ensure quality and engagement and effective integration into current healthcare systems and care pathways, while maintaining patients' autonomy and privacy. Understanding and addressing these issues and successful incorporation of an acceptable, simple, scalable, affordable, and future-proof digital solution into healthcare systems could help remodel global chronic disease management and fractured healthcare systems to provide best patient care and optimisation of healthcare resources to meet the global burden and unmet clinical need of COPD. © The Author(s), 2022.	https://www.scopus.com/inward/record.uri?eid=2-s	Q2	Scopus	EXC_PubType
The Challenges of Spirometric Diagnosis of COPD	2023	Chronic obstructive pulmonary disease (COPD) is one of the top causes of morbidity and mortality worldwide. Although for many years its accurate diagnosis has been a focus of intense research, it is still challenging. Due to its simplicity, portability, and low cost, spirometry has been established as the main tool to detect this condition, but its flawed performance makes it an imperfect COPD diagnosis gold standard. This review aims to provide an up-to-date literature overview of recent studies regarding COPD diagnosis; we seek to identify their limitations and establish perspectives for spirometric diagnosis of COPD in the XXI century by combining deep clinical knowledge of the disease with advanced computer analysis techniques. © 2023 Adriana Maldonado-Franco et al.	https://www.scopus.com/inward/record.uri?eid=2-s	Q2	Scopus	EXC_Scope
Effectiveness of patient activation interventions on chronic obstru	2022	Background: Chronic obstructive pulmonary disease is the third leading cause of death worldwide. Although there is currently no cure for chronic obstructive pulmonary disease, the available self-management strategies can result in improving the symptoms, slowing the disease progression, reducing the frequency of acute exacerbations, improving the patients' quality of life and minimising health care utilisation-associated costs. Patient activation is often considered an essential driver of self-management; however, there are contradictory evidence about its impact on chronic obstructive pulmonary disease self-management. Objective: This review aims to fill this gap by collating the available evidence on the effectiveness of patient activation-driven chronic obstructive pulmonary disease self-management interventions. Methods: Databases including MEDLINE, Academic Search Complete, CINAHL Plus, Science Citation Index, Social Sciences Citation Index, Scopus, APA PsychInfo, EMBASE and ScienceDirect were searched for randomised controlled trials of patient activation-driven chronic obstructive pulmonary disease self-management interventions between 2004 and July 2020. The search terms included chronic obstructive pulmonary disease, self-management/self-care and patient activation/patient engagement. Findings: The initial search resulted in 645 articles, and after reviewing, 10 randomised controlled trials met the inclusion and exclusion criteria. Our review found that patient activation level had a positive association with chronic obstructive pulmonary disease self-management and clinical outcomes, and higher patient activation levels led to better outcomes. The interventions also led to moderate improvements in patient activation level. However, improved patient activation levels did not improve hospitalisation rates, quality of life and mental health. Conclusion: Our findings suggest that patient activation can be used as a reliable tool for improving chronic obstructive pulmonary disease self-management and clinical outcomes; however, it should encompass all aspects of patient activation, especially the emotional aspect. © 2022 National Rural Health Alliance Ltd.	https://www.scopus.com/inward/record.uri?eid=2-s	Q2	Scopus	EXC_Scope
Telehealth for the Longitudinal Management of Chronic Conditior	2022	Background: Extensive literature support telehealth as a supplement or adjunct to in-person care for the management of chronic conditions such as congestive heart failure (CHF) and type 2 diabetes mellitus (T2DM). Evidence is needed to support the use of telehealth as an equivalent and equitable replacement for in-person care and to assess potential adverse effects. Objective: We conducted a systematic review to address the following question: among adults, what is the effect of synchronous telehealth (real-time response among individuals via phone or phone and video) compared with in-person care (or compared with phone, if synchronous video care) for chronic management of CHF, chronic obstructive pulmonary disease, and T2DM on key disease-specific clinical outcomes and health care use? Methods: We followed systematic review methodologies and searched two databases (MEDLINE and Embase). We included randomized or quasi-experimental studies that evaluated the effect of synchronously delivered telehealth for relevant chronic conditions that occurred over ≥2 encounters and in which some or all in-person care was supplanted by care delivered via phone or video. We assessed the bias using the Cochrane Effective Practice and Organization of Care risk of bias (ROB) tool and the certainty of evidence using the Grading of Recommendations Assessment, Development, and Evaluation. We described the findings narratively and did not conduct meta-analysis owing to the small number of studies and the conceptual heterogeneity of the identified interventions. Results: We identified 8662 studies, and 129 (1.49%) were reviewed at the full-text stage. In total, 3.9% (5/129) of the articles were retained for data extraction, all of which (5/5, 100%) were randomized controlled trials. The CHF study (1/5, 20%) was found to have high ROB and randomized patients (n=210) to receive quarterly automated asynchronous web-based review and follow-up of telemetry data versus synchronous personal follow-up (in-person vs phone-based) for 1 year. A 3-way comparison across study arms found no significant differences in clinical outcomes. Overall, 80% (4/5) of the studies (n=466) evaluated synchronous care for patients with T2DM (ROB was judged to be low for 2, 50% of studies and high for 2, 50% of studies). In total, 20% (1/5) of the studies were adequately powered to assess the difference in glycosylated hemoglobin level between groups; however, no significant difference was found. Intervention design varied greatly from remote monitoring of blood glucose combined with video versus in-person visits to an endocrinology clinic to a brief, 3-week remote intervention to stabilize uncontrolled diabetes. No articles were identified for chronic obstructive pulmonary disease. Conclusions: This review found few studies with a variety of designs and interventions that used telehealth as a replacement for in-person care. Future research should consider including observational studies and studies on additional highly prevalent chronic diseases. © 2022 Journal of Medical Internet Research. All rights reserved.	https://www.scopus.com/inward/record.uri?eid=2-s	Q2	Scopus	EXC_Scope

Self-management for people with chronic obstructive pulmonary	2022	Background: Self-management interventions help people with chronic obstructive pulmonary disease (COPD) to acquire and practise the skills they need to carry out disease-specific medical regimens, guide changes in health behaviour and provide emotional support to enable them to control their disease. Since the 2014 update of this review, several studies have been published. Objectives: Primary objectives. To evaluate the effectiveness of COPD self-management interventions compared to usual care in terms of health-related quality of life (HRQoL) and respiratory-related hospital admissions.To evaluate the safety of COPD self-management interventions compared to usual care in terms of respiratory-related mortality and all-cause mortality. Secondary objectives. To evaluate the effectiveness of COPD self-management interventions compared to usual care in terms of other health outcomes and healthcare utilisation.To evaluate effective characteristics of COPD self-management interventions. Search methods: We searched the Cochrane Airways Trials Register, CENTRAL, MEDLINE, EMBASE, trials registries and the reference lists of included studies up until January 2020. Selection criteria: Randomised controlled trials (RCTs) and cluster-randomised trials (CRTs) published since 1995. To be eligible for inclusion, self-management interventions had to include at least two intervention components and include an iterative process between participant and healthcare provider(s) in which goals were formulated and feedback was given on self-management actions by the participant. Data collection and analysis: Two review authors independently selected studies for inclusion, assessed trial quality and extracted data. We resolved disagreements by reaching consensus or by involving a third review author. We contacted study authors to obtain additional information and missing outcome data where possible. Primary outcomes were health-related quality of life (HRQoL), number of respiratory-related hospital admissions, respiratory-related mortality, and all-cause mortality. When appropriate, we pooled study results using random-effects modelling meta-analyses. Main results: We included 27 studies involving 6008 participants with COPD. The follow-up time ranged from two-and-a-half to 24 months and the content of the interventions was diverse. Participants' mean age ranged from 57 to 74 years, and the proportion of male participants ranged from 33% to 98%. The post-bronchodilator forced expiratory volume in one second (FEV1) to forced vital capacity (FVC) ratio of participants ranged from 33.6% to 57.0%. The FEV1/FVC ratio is a measure used to diagnose COPD and to determine the severity of the disease. Studies were conducted on four different continents (Europe (n = 15), North America (n = 8), Asia (n = 1), and Oceania (n = 4); with one study conducted in both Europe and Oceania). Self-management interventions likely improve HRQoL, as measured by the St. George's Respiratory Questionnaire (SGRQ) total score (lower score represents better HRQoL) with a mean difference (MD) from usual care of -2.86 points (95% confidence interval (CI) -4.87 to -0.85; 14 studies, 2778 participants; low-quality evidence). The pooled MD of -2.86 did not reach the SGRQ minimal clinically important difference (MCID) of four points. Self-management intervention participants were also at a slightly lower risk for at least one respiratory-related hospital admission (odds ratio (OR) 0.75, 95% CI 0.57 to 0.98; 15 studies, 3263 participants; very low-quality evidence). The number needed to treat to prevent one respiratory-related hospital admission over a mean of 9.75 months' follow-up was 15 (95% CI 8 to 399) for participants with high baseline risk and 26 (95% CI 15 to 677) for participants with low baseline risk. No differences were observed in respiratory-related mortality (risk difference (RD) 0.01, 95% CI -0.02 to 0.04; 8 studies, 1572 participants; low-quality evidence) and all-cause mortality (RD -0.01, 95% CI -0.03 to 0.01; 24 studies, 5719 participants; low-quality evidence). We graded the evidence to be of 'moderate' to 'very low' quality according to GRADE. All studies had a substantial risk of bias, because of lack of blinding of participants and personnel to the interventions, which is inherently impossible in a self-management intervention. In addition, risk of bias was noticeably increased because of insufficient information regarding a) non-protocol interventions, and b) analyses to estimate the effect of adhering to interventions. Consequently, the highest GRADE evidence score that could be obtained by studies was 'moderate'. Authors' conclusions: Self-management interventions for people with COPD are associated with improvements in HRQoL, as measured with the SGRQ, and a lower probability of respiratory-related hospital admissions. No excess respiratory-related and all-cause mortality risks were observed, which strengthens the view that COPD self-management interventions are unlikely to cause harm. By using stricter inclusion criteria, we decreased heterogeneity in studies, but also reduced the number of included studies and therefore our capacity to conduct subgroup analyses. Data were therefore still insufficient to reach clear conclusions about effective (intervention) characteristics of COPD self-management interventions. As tailoring of COPD self-management interventions to individuals is desirable, heterogeneity is and will likely remain present in self-management interventions. For future studies, we would urge using only COPD self-management interventions that include iterative interactions between participants and healthcare professionals who are competent using behavioural change techniques (BCTs) to elicit participants' motivation, confidence and competence to positively adapt their health behaviour(s) and develop skills to better manage their disease. In addition, to inform further subgroup and meta-regression analyses and to provide stronger conclusions regarding effective COPD self-management interventions, there is a need for more homogeneity in outcome measures. More attention should be paid to behavioural outcome measures and to providing more detailed, uniform and transparently reported data on self-management intervention components and BCTs. Assessment of outcomes over the long term is also recommended to capture changes in people's behaviour. Finally, information regarding non-protocol interventions as well as analyses to estimate the effect of adhering to interventions should be included to increase the quality of evidence. Copyright © 2022 The Cochrane Collaboration. Published by John Wiley & Sons, Ltd.	https://www.scopus.com/inward/record.uri?eid=2-s	Q2	Scopus	EXC_Scope
Promoting Exercise Training Remotely	2022	There has been increased incentivization to develop remote exercise training programs for those living with chronic respiratory diseases, such as chronic obstructive pulmonary disease (COPD). Remote programs offer patients an opportunity to overcome barriers to accessing traditional in-person programs, such as pulmonary rehabilitation (PR). Methods to deliver exercise training remotely range in complexity and types of technological modalities, including phone calls, real-time video conferencing, web-and app-based platforms, video games, and virtual reality (VR). There are a number of studies demonstrating the effectiveness of these programs on exercise capacity, dyspnea, and health-related quality of life (HRQL). However, there is great variation in these programs, making it difficult to assess findings across studies. Other aspects that contribute to the effectiveness of these programs include stakeholder perceptions, such as motivation and willingness to engage, and adherence. Finally, while the intent of these remote programs is to overcome barriers to access, they may inadvertently exacerbate access disparities. Future program development efforts should focus on standardizing how remote exercise training is delivered, engaging stakeholders early on to develop patient-centered programs that patients will want to use, and understanding the heterogeneous preferences and needs of those living with chronic respiratory disease in order to facilitate engagement with these programs. © 2022 by the authors. Licensee MDPI, Basel, Switzerland.	https://www.scopus.com/inward/record.uri?eid=2-s	Q2	Scopus	EXC_Scope
What are the digitally enabled psychosocial interventions deliver	2023	Objectives. Computer-mediated and telephone communication connecting professionals and patients (eHealth) is well established. Yet there is little information about psychosocial interventions delivered by trained practitioners for a palliative care population. The aim is to describe digitally enabled psychosocial interventions offered to adults with life-shortening or terminal illnesses and carers/families receiving palliative care, and how these are delivered and evaluated. Methods. Using Joanna Briggs Institute scoping review methodology, 4 databases (MEDLINE, CINAHL, PsycINFO, and Academic Search Ultimate) were searched (January 2011–April 2021). Inclusion criteria: (a) any design reporting and (b) psychosocial interventions delivered digitally by palliative care health and social care practitioners to (c) adults with life-shortening illnesses. Results. Included papers (n=16) were from Europe ((n=8), Asia (n=2), and the USA (n=6). Research designs encompassed pre- and post-studies, randomized control trials, feasibility, and pilot studies. Tools evaluated psychological, somatic, functional, and psychosocial outcomes. Underpinning approaches included cognitive behavioral therapy, Erikson's life review, coping skills training, psychoeducation, problem-solving therapy, counseling, emotional support and advice, and art therapy. Delivery tools used were telephones, text messages and emails, websites, videos, workbooks, and compact discs. Practitioners included counselors, psychotherapists, psychologists, art therapists, social workers, registered nurses, and trainees. Patients had Alzheimer's disease and related dementias, advanced cancers, chronic obstructive pulmonary disease, and heart failure. Significance of results. COVID-19 has accelerated the usages of digitally enabled psychosocial interventions. Evidence indicates a growing interest in hybrid, novel, synchronous, and asynchronous digital psychosocial interventions for adults with life-shortening illnesses and their caregivers receiving palliative care. © The Author(s), 2023. Published by Cambridge University Press.	https://www.scopus.com/inward/record.uri?eid=2-s	Q2	Scopus	EXC_PubType
Nurse-Led Interventions in Chronic Obstructive Pulmonary Disea	2022	Chronic obstructive pulmonary disease (COPD) is the third leading cause of death worldwide, causing 3.32 million deaths in 2019. COPD management has increasingly become a major component of general and hospital practice and has led to a different model of care. Nurse-led interventions have shown beneficial effects on COPD patient satisfaction and clinical outcomes. This systematic review was conducted to identify and assess nurse-led interventions in COPD patients in terms of mental, physical, and clinical status. The review was carried out following the Preferred Reporting Items for Systematic Reviews and Meta-analyses (PRISMA) statement. The relevance of each manuscript was assessed according to the inclusion criteria, and we retrieved full texts, as required, to reach our conclusions. Data extraction was performed independently by two reviewers, and the risk of bias was assessed using the Cochrane Risk of Bias tool. Forty-eight articles were included in the analysis, which focused on the management of COPD patients by hospital, respiratory and primary nursing care. Nursing management was shown to be highly effective in improving quality of life, emotional state, and pulmonary and physical capacity in COPD patients. In comparison, hospital and respiratory nurses carried out interventions with higher levels of effectiveness than community nurses. © 2022 by the authors.	https://www.scopus.com/inward/record.uri?eid=2-s	Q2	Scopus	EXC_Scope

Non-Pharmacological Treatments of Asthma Chronic Obstructive	2022	[No abstract available]	https://www.scopus.com/inward/record.uri?eid=2-s	Q2	Scopus	EXC_Scope
Telerehabilitation in pulmonary diseases	2023	Purpose of reviewTelerehabilitation is an alternative delivery model for pulmonary rehabilitation, an evidence-based nonpharmacological intervention, in people with chronic pulmonary disease. This review synthesizes current evidence regarding the telerehabilitation model for pulmonary rehabilitation with an emphasis on its potential and implementation challenges, as well as the clinical experiences from the COVID-19 pandemic.Recent findingsDifferent models of telerehabilitation for delivering pulmonary rehabilitation exist. Current studies comparing telerehabilitation to centre-based pulmonary rehabilitation primarily focus on the evaluation in people with stable chronic obstructive pulmonary disease, which demonstrated equivalent improvements in exercise capacity, health-related quality of life and symptoms with improved programme completion rates. Although telerehabilitation may improve access to pulmonary rehabilitation by addressing travel burden, improving schedule flexibility and geographic disparity, there are challenges of ensuring satisfaction of healthcare interactions and delivering core components of initial patient assessment and exercise prescription remotely.SummaryFurther evidence is needed on the role of telerehabilitation in various chronic pulmonary diseases, as well as the effectiveness of different modalities in delivering telerehabilitation programmes. Economic and implementation evaluation of currently available and emerging models of telerehabilitation in delivering pulmonary rehabilitation are needed to ensure sustainable adoption into clinical management for people with chronic pulmonary disease. © 2023 Lippincott Williams and Wilkins. All rights reserved.	https://www.scopus.com/inward/record.uri?eid=2-s	Q2	Scopus	EXC_Scope
Osteosarcopenia in Patients with Chronic Obstructive Pulmonary	2022	Chronic obstructive pulmonary disease (COPD) is a burdensome condition affecting a growing number of people worldwide, frequently related to major comorbidities and functional impairment. In these patients, several factors might have a role in promoting both bone and muscle loss, including systemic inflammation, corticosteroid therapies, sedentary behaviours, deconditioning, malnutrition, smoking habits, and alcohol consumption. On the other hand, bone and muscle tissues share several linkages from functional, embryological, and biochemical points of view. Osteosarcopenia has been recently defined by the coexistence of osteoporosis and sarcopenia, but the precise mechanisms underpinning osteosarcopenia in patients with COPD are still unknown. In this scenario, a deeper understanding of the molecular basis of osteosarcopenia might guide clinicians in a personalized approach integrating skeletal muscle health with the pulmonary rehabilitation framework in COPD. Taken together, our results summarized the currently available evidence about the multilevel interactions between osteosarcopenia and COPD to pave the way for a comprehensive approach targeting the most common risk factors of these pathological conditions. Further studies are needed to clarify the role of modern clinical strategies and telemedicine solutions to optimize healthcare delivery in patients with COPD, including osteopenia, osteoporosis, and sarcopenia screening in these subjects. © 2022 by the authors.	https://www.scopus.com/inward/record.uri?eid=2-s	Q2	Scopus	EXC_Scope
Effectiveness of motivational interviewing among patients with C	2022	Objective: To evaluate the effectiveness of motivational interviewing (MI) for COPD in behavioral changes and health outcomes, and also verify the reliability of results in conjunction with trial sequential analysis and the Grading of Recommendations Assessment, Development, and Evaluation tool. Methods: Studies that implemented MI interventions for COPD patients were systematically searched by eight databases from inception to December 2021. Study screening, quality assessment, data extraction, and meta-analysis were conducted according to Cochrane standards. Results: Twenty-one studies involving 2344 patients were included. The results of meta-analyses indicated that MI made significant improvement in self-efficacy, lung function, quality of life, emotion, and COPD-related admission, but not in self-management and exercise capacity. Subgroup analyses found that the intervention duration was inversely associated with effect size for both self-efficacy and negative emotion severity. The trial sequential analysis showed MI improved patients' lung function and reduced COPD-related hospitalization with certainty, but the findings for exercise capacity need to be confirmed by further research. Conclusions: This systematic review suggested the positive effects of MI on self-efficacy, lung function, quality of life, emotion and COPD-related hospitalization. To make a firm conclusion, more well-designed clinical trials with bigger sample sizes required. Practice implications: Clinical and community nurses can use MI for COPD to increase healthy behaviors. Trial registration: CRD42021278674. © 2022 Elsevier B.V.	https://www.scopus.com/inward/record.uri?eid=2-s	Q2	Scopus	EXC_Scope
Evidence-based management approaches for patients with seve	2022	Background: Patients with chronic obstructive pulmonary disease (COPD) face limited treatment options and inadequate access to palliative care. Aim: To provide a pragmatic overview of clinical guidelines and produce evidence-based recommendations for severe COPD. Interventions for which there is inconsistent evidence to support their use and areas requiring further research were identified. Design: Practice review of guidelines supported by scoping review methodology to examine the evidence reporting the use of guideline-recommended interventions. Data sources: An electronic search was undertaken in MEDLINE, EMBASE, PsycINFO, CINAHL and The Cochrane Database of Systematic Reviews, complemented by web searching for guidelines and publications providing primary evidence (July 2021). Guidelines published within the last 5 years and evidence in the last 10 years were included. Results: Severe COPD should be managed using a multidisciplinary approach with a holistic assessment. For stable patients, long-acting beta-agonist/long-acting muscarinic antagonist and pulmonary rehabilitation are recommended. Low dose opioids, self-management, handheld fan and nutritional support may provide small benefits, whereas routine corticosteroids should be avoided. For COPD exacerbations, systematic corticosteroids, non-invasive ventilation and exacerbation action plans are recommended. Short-acting inhaled beta-agonists and antibiotics may be considered but pulmonary rehabilitation should be avoided during hospitalisation. Long term oxygen therapy is only recommended for patients with chronic severe hypoxaemia. Short-acting anticholinergic inhalers, nebulised opioids, oral theophylline or telehealth are not recommended. Conclusions: Recommended interventions by guidelines are not always supported by high-quality evidence. Further research is required on efficacy and safety of inhaled corticosteroids, antidepressants, benzodiazepines, mucolytics, relaxation and breathing exercises. © The Author(s) 2022.	https://www.scopus.com/inward/record.uri?eid=2-s	Q2	Scopus	EXC_PubType
The Influence of Sex, Gender, or Age on Outcomes of Digital Tec	2022	Background: Chronic obstructive pulmonary disease (COPD) is a common chronic disease that can be treated and monitored with various digital technologies. Digital technologies offer unique opportunities for treating and monitoring people with chronic diseases, but little is known about whether the outcomes of such technologies depend on sex, gender, or age in people with COPD. Objective: The general objective of this study is to assess the possible influence of sex, gender, or age on outcomes of digital technologies for treatment and monitoring of COPD through an overview of systematic reviews. Methods: The study is planned as an overview of systematic reviews. Study reporting is based on the PRISMA (Preferred Reporting Items for Systematic reviews and Meta-Analyses) 2020 guidelines because guidelines for overviews are not available as of this writing. The information sources for the overview will include 4 bibliographic databases (MEDLINE, Cochrane Library, Epistemonikos, and Web of Science) as well as the bibliographies of the included systematic reviews. The electronic search strategy will be developed and conducted in collaboration with an experienced database specialist. The search results will be presented in accordance with the PRISMA 2020 guidelines. The eligibility of studies is based on the population, intervention, comparison, outcomes, and study design (PICOS) criteria: (1) people with COPD (population), (2) digital technology intervention for treatment or monitoring (intervention), (3) any control group or no control group (comparison), (4) any outcome, and (5) systematic review of randomized controlled trials or non–randomized controlled trials with or without a meta-analysis (study design). Critical appraisal of the included systematic reviews will be performed using A Measurement Tool to Assess Systematic Reviews, version 2 (AMSTAR 2). Data will be extracted using a standardized data extraction sheet. Results: The literature search is scheduled for June 2022. We expect to select the relevant systematic reviews, code the data, and appraise the systematic reviews by December 2022. Conclusions: There is a growing recognition that the influence of sex, gender, or age should be considered in research design and outcome reporting in the context of health care interventions. Our overview will identify systematic reviews of various digital technologies for treatment or monitoring of COPD. The most interesting aspect of the overview will be to investigate if any systematic reviews considered the influence of sex, gender, or age on the outcomes of such digital technologies in COPD. Evidence from the overview could be used to guide more individualized (sex, gender, or age-based) recommendations for the use of digital technologies among people with COPD. © Katja Matthias, Ivonne Honekamp, Karina Karolina De Santis.	https://www.scopus.com/inward/record.uri?eid=2-s	Q2	Scopus	EXC_PubType
The role of the clinical pharmacist in the respiratory or sleep mult	2023	The role of the pharmacist has evolved significantly, not least over the last 20 years. It delivers a skilled profession with a vital role in medicines optimisation and the management of patients with a respiratory or sleep disorder. While pharmacists are capable of acting as independent practitioners delivering direct patient care, this article explores their contribution to multidisciplinary teams within asthma, COPD, cystic fibrosis, tuberculosis, interstitial lung disease and sleep medicine. Having identified patient cohorts needing specialist medicines support, notably those with poor medicines adherence or specific medicines-related needs (for example during adolescence, or women who are pregnant or breastfeeding), these pharmacists work within primary, secondary and specialist tertiary care. The aim of this review is to share and inspire innovative models of working to include more pharmacists in respiratory and sleep medicine. © ERS 2023.	https://www.scopus.com/inward/record.uri?eid=2-s	Q2	Scopus	EXC_Scope

Implementation of e–Mental Health Interventions for Informal Ca	2022	Background: Informal caregivers commonly experience mental health difficulties related to their caregiving role. e–Mental health interventions provide mental health support in a format that may be more accessible to informal caregivers. However, e–mental health interventions are seldom implemented in real-world practice. Objective: This mixed methods systematic review aimed to examine factors associated with the effectiveness and implementation of e–mental health interventions for informal caregivers of adults with chronic diseases. To achieve this aim, two approaches were adopted: combinations of implementation and intervention characteristics sufficient for intervention effectiveness were explored using qualitative comparative analysis, and barriers to and facilitators of implementation of e–mental health interventions for informal caregivers were explored using thematic synthesis. Methods: We identified relevant studies published from January 1, 2007, to July 6, 2022, by systematically searching 6 electronic databases and various secondary search strategies. Included studies reported on the effectiveness or implementation of e–mental health interventions for informal caregivers of adults with cancer, chronic obstructive pulmonary disease, dementia, diabetes, heart disease, or stroke. Randomized controlled trials reporting on caregivers' mental health outcomes were included in a crisp-set qualitative comparative analysis. We assessed randomized controlled trials for bias using the Risk of Bias 2.0 tool, and we assessed how pragmatic or explanatory their trial design was using the Pragmatic Explanatory Continuum Indicator Summary 2 tool. Studies of any design reporting on implementation were included in a thematic synthesis using the Consolidated Framework for Implementation Research to identify barriers to and facilitators of implementation. Results: Overall, 53 reports, representing 29 interventions, were included in the review. Most interventions (27/29, 93%) focused on informal cancer or dementia caregivers. In total, 14 reports were included in the qualitative comparative analysis, exploring conditions including the presence of peer or professional support and key persuasive design features. Low consistency and coverage prevented the determination of condition sets sufficient for intervention effectiveness. Overall, 44 reports were included in the thematic synthesis, and 152 barriers and facilitators were identified, with the majority related to the intervention and individual characteristic domains of the Consolidated Framework for Implementation Research. Implementation barriers and facilitators in the inner setting (eg, organizational culture) and outer setting (eg, external policies and resources) domains were largely unexplored. Conclusions: e–Mental health interventions for informal caregivers tend to be well-designed, with several barriers to and facilitators of implementation identified related to the intervention and individual user characteristics. Future work should focus on exploring the views of stakeholders involved in implementation to determine barriers to and facilitators of implementing e–mental health interventions for informal caregivers, focusing on inner and outer setting barriers and facilitators. Trial Registration: PROSPERO (International Prospective Register of Systematic Reviews) CRD42020155727; https://www.crd.york.ac.uk/prospero/display_record.php?ID=CRD42020155727 International Registered Report Identifier (IRRID): RR2-10.1136/bmjopen-2019-035406. ©Chelsea Coumoundouros, Erika Mårtensson, Giulia Ferraris, Justine Margaux Zuidberg, Louise von Essen, Robbert Sanderman, Joanne Woodford.	https://www.scopus.com/inward/record.uri?eid=2-s	Q2	Scopus	EXC_Scope
Chronic Bronchial Infection in Stable COPD; [Infección bronquial	2023	Classically, the role of pulmonary infection in patients with chronic obstructive pulmonary disease (COPD) has focused on periods of exacerbation. However, in a clinically stable situation, little attention has been paid to the role play by the presence of potentially pathogenic micro-organisms (especially when they are repeatedly isolated, which is known as chronic bronchial infection [CBI]) in the pathogenesis, prognosis and disease treatment. In recent years, however, some evidence has been published on the deleterious effect of CBI in COPD since it has been associated to greater local inflammation, greater disease severity, poorer health-related quality of life, greater number and severity of exacerbations and cardiovascular events, presence of bronchiectasis, rapid decline of lung function, and even higher mortality (especially related to Pseudomonas aeruginosa infection). It is not known, however, what is the role of anti-inflammatory or antibiotic treatments on CBI in stable COPD patients. This review focused on the scientific evidence on this topic that exists up to now, from the very definition of IBC in COPD to the possible treatments that this situation entails, as well as the numerous future challenges it generates, especially the development of knowledge of the pulmonary microbiome, its alterations and its consequences. © 2023 Sociedad Española de Neumología y Cirugía Torácica (SEPAR)	https://www.scopus.com/inward/record.uri?eid=2-s	Q2	Scopus	EXC_Scope
COPD: Providing the right treatment for the right patient at the ri	2023	Chronic Obstructive Pulmonary Disease (COPD) is a common disease associated with significant morbidity and mortality that is both preventable and treatable. However, a major challenge in recognizing, preventing, and treating COPD is understanding its complexity. While COPD has historically been characterized as a disease defined by airflow limitation, we now understand it as a multi-component disease with many clinical phenotypes, systemic manifestations, and associated co-morbidities. Evidence is rapidly emerging in our understanding of the many factors that contribute to the pathogenesis of COPD and the identification of "early" or "pre-COPD" which should provide exciting opportunities for early treatment and disease modification. In addition to breakthroughs in our understanding of the origins of COPD, we are optimizing treatment strategies and delivery of care that are showing impressive benefits in patient-centered outcomes and healthcare utilization. This special issue of Respiratory Medicine, "COPD: Providing the Right Treatment for the Right Patient at the Right Time" is a summary of the proceedings of a conference held in Stresa, Italy in April 2022 that brought together international experts to discuss emerging evidence in COPD and Pulmonary Rehabilitation in honor of a distinguished friend and colleague, Claudio Ferdinando Donor (1948–2021). Claudio was a true pioneer in the field of pulmonary rehabilitation and the comprehensive care of individuals with COPD. He held numerous leadership roles in in the field, provide editorial stewardship of several respiratory journals, authored numerous papers, statement and guidelines in COPD and Pulmonary Rehabilitation, and provided mentorship to many in our field. Claudio's most impressive talent was his ability to organize spectacular conferences and symposia that highlighted cutting edge science and clinical medicine. It is in this spirit that this conference was conceived and planned. These proceedings are divided into 4 sections which highlight crucial areas in the field of COPD: (1) New concepts in COPD pathogenesis; (2) Enhancing outcomes in COPD; (3) Non-pharmacologic management of COPD; and (4) Optimizing delivery of care for COPD. These presentations summarize the newest evidence in the field and capture lively discussion on the exciting future of treating this prevalent and impactful disease. We thank each of the authors for their participation and applaud their efforts toward pushing the envelope in our understanding of COPD and optimizing care for these patients. We believe that this edition is a most fitting tribute to a dear colleague and friend and will prove useful to students, clinicians, and researchers as they continually strive to provide the right treatment for the right patient at the right time. It has been our pleasure and a distinct honor to serve as editors and oversee such wonderful scholarly work. © 2022 Elsevier Ltd	https://www.scopus.com/inward/record.uri?eid=2-s	Q2	Scopus	EXC_Scope
Integrating digital inhalers into clinical care of patients with asthm	2022	Modernizing inhaled medications through digital technology can help address persistent problems of non-adherence and poor inhaler technique in patients with obstructive lung diseases. With a growing body of supportive clinical studies, advances in digital inhaler sensors and platforms, greater support from payers and healthcare organizations, significant growth with these technologies is expected. While all digital (smart) inhalers record adherence, these are distinguished by their compatibility with commercial inhalers, capabilities to guide inhaler technique, use of patient-reported outcomes, and user-friendliness for both the healthcare professional (HCP) and patient. Due to the complexity and novelty of employing digital inhalers, collaboration with multiple entities within health systems is necessary and a well-planned integration is needed. For HCPs and patients, cybersecurity and privacy are critical, it will require review by each healthcare organization. In the US, some payers reimburse for remote monitoring using digital inhalers, but reimbursement is currently unavailable in other countries. There are several models for remote patient care, as employing an active, ongoing digital interface between the HCP and patient or they may choose to only review data at clinical encounters. Personalization of therapies and feedback are key to success. While digital inhaler malfunction uncommonly occurs, patient attrition over a year is significant. Some patients will be challenged to use digital platforms or have the necessary technology. Additional research is needed to address cost-effectiveness, in vivo accuracy of inspiratory measurement capable devices, ability to teach inhaler technique, their application for monitoring lung function, and lastly real-world adoption and implementation in routine clinical practice. © 2022 Elsevier Ltd	https://www.scopus.com/inward/record.uri?eid=2-s	Q2	Scopus	EXC_Scope
Impact of integrated health care on elderly population: A systema	2022	Background: Care fragmentation in the elderly population prompted the need for integrated health care systems. However, evidence regarding the impact of the integrated care system in Taiwan is unclear. We aimed to conduct a systematic review to evaluate the impact of Taiwan's integrated health care programs on geriatric population. Methods: We searched bibliographic databases MEDLINE, Embase, Web of Science, and Airiti Library for relevant publications throughout May 2022. Studies investigating the effectiveness of Taiwan's integrated care programs were included. We used the critical appraisal skills programme (CASP) checklist, to assess the risk of bias of included studies. Results: Thirty-four studies, with a total of 838,026 study subjects, were assessed. The systematic review on 11 subthemes (diabetes mellitus, chronic kidney disease, hepatitis C virus, fractures, cancer, dementia, atrial fibrillation, chronic obstructive pulmonary disease, mechanical ventilation, terminal illness, outpatients and community-dwelling patients), demonstrated that the implementation of integrated health care could not only provide benefits on survival, self-care ability, health quality, physical, and functional rehabilitation outcomes, but also significantly reduce medical utilization and expenditures. Conclusion: The integrated health care system for multiple morbidities benefits the Taiwanese geriatric population in physical and functional outcomes. The thematic synthesis provides references for future rigorous clinical trials. © 2022	https://www.scopus.com/inward/record.uri?eid=2-s	Q2	Scopus	EXC_Scope

Telemedicine in the management of chronic obstructive pulmona	2023	Telemedicine is defined as the use of electronic technology for information and communication by healthcare professionals with patients (or care givers) aiming at providing and supporting healthcare to patients away from healthcare institutions. This systematic review over the last decade (2013–2022) investigates the use of telemedicine in patients with chronic obstructive pulmonary disease (COPD). We identified 53 publications related to: (1) home tele-monitorization; (2) tele-education and self-management; (3) telerehabilitation; and (4) mobile health (mHealth). Results showed that, although evidence is still weak in many of these domains, results are positive in terms of improvement of health-status, use of health-care resources, feasibility, and patient satisfaction. Importantly, no safety issues were identified. Thus, telemedicine can be considered today as a potential complement to usual healthcare. © 2023 Elsevier España, S.L.U.	https://www.scopus.com/inward/record.uri?eid=2-s	Q2	Scopus	EXC_Language
Pulmonary Rehabilitation Using Minimal Equipment for People W	2023	Objective. Pulmonary rehabilitation programs that use minimal equipment for exercise training, rather than gymnasium equipment, would enable delivery of pulmonary rehabilitation to a greater number of people with chronic obstructive pulmonary disease (COPD). The effectiveness of minimal equipment programs in people with COPD is unclear. This systematic review and meta-analysis aimed to determine the effects of pulmonary rehabilitation using minimal equipment for aerobic and/or resistance training in people with COPD. Methods. Literature databases were searched up to September 2022 for randomized controlled trials (RCTs) comparing the effect of minimal equipment programs with usual care or with exercise equipment-based programs for exercise capacity, health-related quality of life (HRQoL), and strength. Results. Nineteen RCTs were included in the review and 14 RCTs were included in the meta-analyses, which reported low to moderate certainty of evidence. Compared with usual care, minimal equipment programs increased 6-minute walk distance (6MWD) by 85 m (95% CI = 37 to 132 m). No difference in 6MWD was observed between minimal equipment and exercise equipment-based programs (14 m, 95% CI = −27 to 56 m). Minimal equipment programs were more effective than usual care for improving HRQoL (standardized mean difference = 0.99, 95% CI = 0.31 to 1.67) and were not different from exercise equipment-based programs for improving upper limb strength (6 N, 95% CI = −2 to 13 N) or lower limb strength (20 N, 95% CI = −30 to 71 N). Conclusion. In people with COPD, pulmonary rehabilitation programs using minimal equipment elicit clinically significant improvements in 6MWD and HRQoL and are comparable with exercise equipment–based programs for improving 6MWD and strength. Impact. Pulmonary rehabilitation programs using minimal equipment may be a suitable alternative in settings where access to gymnasium equipment is limited. Delivery of pulmonary rehabilitation programs using minimal equipment may improve access to pulmonary rehabilitation worldwide, particularly in rural and remote areas and in developing countries. © The Author(s) 2023. Published by Oxford University Press on behalf of the American Physical Therapy Association.	https://www.scopus.com/inward/record.uri?eid=2-s	Q2	Scopus	EXC_Scope
Efficacy of tele-rehabilitation in patients with chronic obstructive p	2023	Since 2020 we have lived an exceptional situation that made us experience a complete lockdown due to SARS-CoV-2, what affected the treatments of different pathologies, such as the chronic obstructive pulmonary disease (COPD). Because of those reasons, it has arisen the idea of implementing the tele-rehabilitation program as a treatment of these pathologies. The search was done between the months of October and November 2020, with the aim of analyzing and updating the efficacy of the tele-rehabilitation in patients who have COPD, finding eight articles which met the inclusion criteria. The pulmonary tele-rehabilitation is able to improve the quality of life and physical state, and decreasing the number of hospitalizations and exacerbations. Furthermore, patients showed a great level of satisfaction and adherence to this treatment program. The pulmonary tele-rehabilitation can achieve similar results as of pulmonary rehabilitation. For this reason, people who have difficulties to go to their outpatients clinic or even in a lockdown can use it. However, it is necessary to investigate which tele-rehabilitation program is better. © 2022	https://www.scopus.com/inward/record.uri?eid=2-s	Q2	Scopus	EXC_Scope
Pulmonary rehabilitation outcomes in individuals with chronic obs	2022	Background: The magnitude of response to pulmonary rehabilitation (PR) is influenced by the selection of outcomes and measures. Objectives: This systematic review aimed to review all outcomes and measures used in clinical trials of PR for individuals with chronic obstructive pulmonary disease (COPD). Methods: The review involved a search of Scopus, Web of Knowledge, Cochrane Library, EBSCO, Science Direct and PubMed databases for studies of stable individuals with COPD undergoing PR. Frequency of reporting for each domain, outcome and measure was synthesized by using Microsoft Excel. Results: We included 267 studies (43153 individuals with COPD). A broad range of domains (n = 22), outcomes (n = 163) and measures (n = 217) were reported. Several measures were used for the same outcome. The most reported outcomes were exercise capacity (n = 218) assessed with the 6-min walk test (n = 140), health-related quality of life (n = 204) assessed with the Saint George's respiratory questionnaire (n = 99), and symptoms (n = 158) assessed with the modified Medical Research Council dyspnoea scale (n = 56). The least reported outcomes were comorbidities, adverse events and knowledge. Conclusions: This systematic review reinforces the need for a core outcome set for PR in individuals with COPD because of high heterogeneity in reported outcomes and measures. Future studies should assess the importance of each outcome for PR involving different stakeholders. PROSPERO ID: CRD42017079935 © 2021 Elsevier Masson SAS	https://www.scopus.com/inward/record.uri?eid=2-s	Q2	Scopus	EXC_Scope
The Effectiveness and Safety of Long-Term Macrolide Therapy fo	2023	Background: Chronic obstructive pulmonary disease (COPD) is a prevalent condition with fewer treatments available as the severity increases. Previous systematic reviews have demonstrated the benefits of long-term macrolide use. However, the therapeutic differences between different macrolides and the optimal duration of use remain unclear. Methods: A systematic review and meta-analysis were conducted to assess the effectiveness of long-term macrolide use in reducing COPD exacerbations, compare the therapeutic differences among macrolides, and determine the appropriate treatment duration. Four databases (PubMed, Cochrane Library, Web of Science, and ICHU-SHI) were searched until 20 March 2023, and a random-effects model was used to calculate the pooled effect. Results: The meta-analysis included nine randomized controlled trials involving 1965 patients. The analysis revealed an odds ratio (OR) of 0.34 (95% confidence interval [CI] 0.19, 0.59, p < 0.001) for the reduction in exacerbation frequency. Notably, only azithromycin or erythromycin showed suppression of COPD exacerbations. The ORs for reducing exacerbation frequency per year and preventing hospitalizations were −0.50 (95% CI: −0.81, −0.19; p = 0.001) and 0.60 (95% CI: 0.3, 0.97; p = 0.04), respectively. Statistical analyses showed no significant differences between three- and six-month macrolide prescriptions. However, studies involving a twelve-month prescription showed an OR of 0.27 (95% CI: 0.11, 0.68; p = 0.005; I2 = 81%). Although a significant improvement in St George's Respiratory Questionnaire (SGRQ) total scores was observed with a mean difference of −4.42 (95% CI: −9.0, 0.16; p = 0.06; I2 = 94%), the minimal clinically important difference was not reached. While no adverse effects were observed between the two groups, several studies have reported an increase in bacterial resistance. Conclusions: Long-term use of azithromycin or erythromycin suppresses COPD exacerbations, and previous studies have supported the advantages of a 12-month macrolide prescription over a placebo. © 2023 by the authors.	https://www.scopus.com/inward/record.uri?eid=2-s	Q2	Scopus	EXC_Scope
Monitoring Long Term Noninvasive Ventilation: Benefits, Caveats	2022	Long term noninvasive ventilation (LTNIV) is a recognized treatment for chronic hypercapnic respiratory failure (CHRF). COPD, obesity-hypoventilation syndrome, neuromuscular disorders, various restrictive disorders, and patients with sleep-disordered breathing are the major groups concerned. The purpose of this narrative review is to summarize current knowledge in the field of monitoring during home ventilation. LTNIV improves symptoms related to CHRF, diurnal and nocturnal blood gases, survival, and health-related quality of life. Initially, patients with LTNIV were most often followed through elective short in-hospital stays to ensure patient comfort, correction of daytime blood gases and nocturnal oxygenation, and control of nocturnal respiratory events. Because of the widespread use of LTNIV, elective in-hospital monitoring has become logistically problematic, time consuming, and costly. LTNIV devices presently have a built-in software which records compliance, leaks, tidal volume, minute ventilation, cycles triggered and cycled by the patient and provides detailed pressure and flow curves. Although the engineering behind this information is remarkable, the quality and reliability of certain signals may vary. Interpretation of the curves provided requires a certain level of training. Coupling ventilator software with nocturnal pulse oximetry or transcutaneous capnography performed at the patient's home can however provide important information and allow adjustments of ventilator settings thus potentially avoiding hospital admissions. Strategies have been described to combine different tools for optimal detection of an inefficient ventilation. Recent devices also allow adapting certain parameters at a distance (pressure support, expiratory positive airway pressure, back-up respiratory rate), thus allowing progressive changes in these settings for increased patient comfort and tolerance, and reducing the requirement for in-hospital titration. Because we live in a connected world, analyzing large groups of patients through treatment of "big data" will probably improve our knowledge of clinical pathways of our patients, and factors associated with treatment success or failure, adherence and efficacy. This approach provides a useful add-on to randomized controlled studies and allows generating hypotheses for better management of HMV. Copyright © 2022 Janssens, Cantero, Pasquina, Georges and Rabec.	https://www.scopus.com/inward/record.uri?eid=2-s	Q2	Scopus	EXC_Scope

Health literacy interventions among patients with chronic disease	2023	Objectives: This study was conducted to determine the effectiveness and impact of health literacy interventions for patients with chronic diseases. Methods: We searched PubMed, Web of Science, Embase, Scopus, and EBSCO CINAHL from inception through March 2022. Eligible chronic diseases include diabetes, heart disease, cancer, and chronic obstructive pulmonary disease. RCTs were included in eligible studies to assess health literacy and other relevant health outcomes. Two investigators selected studies, extracted data, and assessed the methodological quality of included studies independently. Results: A total of 18 studies involving 5384 participants were included in the final analysis. The implementation of health literacy interventions exhibited a significant improvement in the health literacy level of individuals diagnosed with chronic diseases (SMD = 0.75, 95% CI = 0.40–1.10). Analysis of heterogeneity sources indicated statistically significant variations in the effects of interventions across different diseases and age groups ($P < 0.05$). However, no significant impact was observed on patients with chronic obstructive pulmonary disease (COPD), interventions with a follow-up duration exceeding three months, or application-based interventions on the health literacy level of individuals with chronic diseases. Remarkably, our findings revealed that health literacy interventions exerted a positive influence on health status (SMD = 0.74, 95% CI = 0.13–1.34), depression and anxiety (SMD = 0.90, 95% CI = 0.17–1.63), as well as self-efficacy (SMD = 0.28, 95% CI = 0.15–0.41) among patients diagnosed with chronic diseases. Furthermore, a specific analysis was conducted to evaluate the effects of these interventions on hypertension and diabetes control. The results demonstrated that health literacy interventions were more effective in enhancing hypertension control compared to diabetes control. Conclusion: Health literacy interventions have demonstrated effectiveness in improving the health of patients with chronic diseases. The importance of emphasizing the quality of these interventions cannot be overstated, as factors such as appropriate intervention tools, extended intervention duration, and reliable primary care services play crucial roles in their efficacy. © 2023 Elsevier B.V.	https://www.scopus.com/inward/record.uri?eid=2-s	Q2	Scopus	EXC_Scope
Clinical Impact of Electronic Monitoring Devices of Inhalers in Ad	2023	We conducted a systematic review and meta-analysis to gain insight into the characteristics and clinical impact of electronic monitoring devices of inhalers (EMDs) and their clinical interventions in adult patients with asthma or COPD. The search included PubMed, Web of Science, Cochrane, Scopus and Embase databases, as well as official EMDs websites. We found eight observational studies and ten clinical trials, assessing a wide range of clinical outcomes. Results from the meta-analysis on adherence to inhalers in a period over three months were favourable in the EMD group (fixed effects model: SMD: 0.36 [0.25–0.48]; random effects model SMD: 0.41 [0.22–0.60]). An exploratory meta-analysis found an improvement in ACT score (fixed effect model SMD: 0.25 [0.11–0.39]; random effects model: SMD: 0.47 [−0.14–1.08]). Other clinical outcomes showed mixed results in the descriptive analyses. The findings of this review highlight the benefits of EMDs in the optimization of adherence to inhaled therapy as well as the potential interest in other clinical outcomes. © 2023 by the authors.	https://www.scopus.com/inward/record.uri?eid=2-s	Q2	Scopus	EXC_Scope
Chronic obstructive pulmonary disease	2022	Chronic obstructive pulmonary disease (COPD) is a major cause of morbidity, mortality, and health-care use worldwide. COPD is caused by exposure to inhaled noxious particles, notably tobacco smoke and pollutants. However, the broad range of factors that increase the risk of development and progression of COPD throughout the life course are increasingly being recognised. Innovations in omics and imaging techniques have provided greater insight into disease pathobiology, which might result in advances in COPD prevention, diagnosis, and treatment. Although few novel treatments have been approved for COPD in the past 5 years, advances have been made in targeting existing therapies to specific subpopulations using new biomarker-based strategies. Additionally, COVID-19 has undeniably affected individuals with COPD, who are not only at higher risk for severe disease manifestations than healthy individuals but also negatively affected by interruptions in health-care delivery and social isolation. This Seminar reviews COPD with an emphasis on recent advances in epidemiology, pathophysiology, imaging, diagnosis, and treatment. © 2022 Elsevier Ltd	https://www.scopus.com/inward/record.uri?eid=2-s	Q2	Scopus	EXC_Scope
Are older adults considered in asthma and chronic obstructive pu	2023	Background: The use of mobile health (mHealth) for asthma and chronic obstructive pulmonary disease (COPD) is rapidly growing and may help address the complex respiratory care needs of our ageing population. However, little is currently known about how airways mHealth is developed and used among older adults (≥65 years). Objective: To identify if and how older adults with asthma and COPD have been incorporated across the mHealth research cycle. Methods: We searched Ovid MEDLINE, EMBASE, CINAHL and the Cochrane Central Registry of Controlled Trials for studies pertaining to the development or evaluation of asthma and COPD mHealth for adults published after 2010. Study, participant and mHealth details, including any considerations of older age, were extracted, synthesised and charted. Results: A total of 334 studies of 191 mHealth tools were identified. Adults ≥65 years old were included in 33.3% of asthma mHealth studies and 85.3% of COPD studies. Discussions of older age focused on barriers to technology use. Methodologic and/or analytic considerations of older age were mostly absent throughout the research cycle. Among the 28 instances quantitative age-related analyses were detailed, 12 described positive mHealth use and satisfaction outcomes in older adults versus negative or equivocal outcomes. Conclusion: We identified an overall lack of consideration for older age throughout the airways mHealth research cycle, even among COPD mHealth studies that predominantly included older adults. We also found a contrast between the perceptions of how older age might negatively influence mHealth use and available quantitative evaluations. Future airways mHealth research must better integrate the needs and concerns of older adults. © 2023 The Author(s). Published by Oxford University Press on behalf of the British Geriatrics Society. All rights reserved.	https://www.scopus.com/inward/record.uri?eid=2-s	Q2	Scopus	EXC_PubType
Effects of different interventions on smoking cessation in chronic	2022	Background and objectives: Smoking is responsible for 9 out of 10 deaths related to chronic obstructive pulmonary disease, and this number can be reduced by quitting smoking. In this study, the effect of different interventions on smoking cessation of patients with chronic obstructive pulmonary disease was assessed through a network meta-analysis. Methods: Eight databases were searched to obtain randomized controlled trials involving different interventions for smoking cessation in chronic obstructive pulmonary disease patients. The Cochrane Handbook tool was employed to assess the risk bias of included studies. Network meta-analysis was performed using STATA software. Results: A total of 23 studies involving 13,480 patients were included. Eight studies were rated as having a high risk of bias, seven studies had a low risk, and in eight studies, the risk was unclear. All studies employed 13 different interventions, including eight monotherapies and five combination therapies. Network meta-analysis showed that a combination of behavioral therapy and pharmacotherapy was superior in achieving patients' smoking cessation compared to monotherapy. Moreover, varenicline was more helpful for smoking cessation than other single interventions. The final surface under the cumulative ranking curve value indicated that cognitive behavior therapy combined with bupropion achieved the best smoking cessation effect. Conclusions: The obtained results indicate that a combination of behavioral therapy and pharmacotherapy is most powerful in helping chronic obstructive pulmonary disease patients to quit smoking. Researchers should focus more on the safety of pharmacotherapeutic interventions. Moreover, more high-quality trials investigating the stability of evidence levels of different interventions on abstinence must be conducted. © 2022 Elsevier Ltd	https://www.scopus.com/inward/record.uri?eid=2-s	Q2	Scopus	EXC_Scope
Remote patient monitoring in the management of chronic obstruc	2022	Remote patient monitoring allows monitoring high-risk patients through implementation of an expanding number of technologies in coordination with a healthcare team to augment care, with the potential to provide early detection of exacerbation, prompt access to therapy and clinical services, and ultimately improved patient outcomes and decreased healthcare utilization. In this review, we describe the application of remote patient monitoring in chronic obstructive pulmonary disease including the potential benefits and possible barriers to implementation both for the individual and the healthcare system. © 2022 Journal of Investigative Medicine. All rights reserved.	https://www.scopus.com/inward/record.uri?eid=2-s	Q2	Scopus	EXC_PubType
Rehabilitation Technologies for Chronic Conditions: Will We Sink	2023	Introduction: Chronic conditions such as stroke, Parkinson's disease, spinal cord injury, multiple sclerosis, vestibular disorders, chronic pain, arthritis, diabetes, chronic obstructive pulmonary disease (COPD), and heart disease are leading causes of disability among middle-aged and older adults. While evidence-based treatment can optimize clinical outcomes, few people with chronic conditions engage in the recommended levels of exercise for clinical improvement and successful management of their condition. Rehabilitation technologies that can augment therapeutic care—i.e., exoskeletons, virtual/augmented reality, and remote monitoring—offer the opportunity to bring evidence-based rehabilitation into homes. Successful integration of rehabilitation techniques at home could help recovery and access and foster long term self-management. However, widespread uptake of technology in rehabilitation is still limited, leaving many technologies developed but not adopted. Methods: In this narrative review, clinical need, efficacy, and obstacles and suggestions for implementation are discussed. The use of three technologies is reviewed in the management of the most prevalent chronic diseases that utilize rehabilitation services, including common neurological, musculoskeletal, metabolic, pulmonary, and cardiac conditions. The technologies are (i) exoskeletons, (ii) virtual and augmented reality, and (iii) remote monitoring. Results: Effectiveness evidence backing the use of technology in rehabilitation is growing but remains limited by high heterogeneity, lack of long-term outcomes, and lack of adoption outcomes. Conclusion: While rehabilitation technologies bring opportunities to bridge the gap between clinics and homes, there are many challenges with adoption. Hybrid effectiveness and implementation trials are a possible path to successful technology development and adoption. © 2023 by the authors.	https://www.scopus.com/inward/record.uri?eid=2-s	Q2	Scopus	EXC_Scope

Respiratory telerehabilitation in cases of COPD; [Mise au point s	2022	Telerehabilitation brings together a set of rehabilitation practices applied remotely by means of information and communication technologies. Even though it has been taking on increasing importance in many health fields over 10 years, telerehabilitation had yet to find its place in pulmonary rehabilitation before 2020, when the pandemic situation impelled numerous teams to put it to work. Pilot studies on respiratory diseases, primarily COPD, along with recent data from randomized or non-randomized studies, have enhanced our understanding of "remote" practice. In this review of the literature, we will show that pulmonary telerehabilitation is feasible, safe and likely to yield short-term (and possibly longer term) effects generally similar to those achieved in the pulmonary rehabilitation programs of specialized centers, especially as regards some indicators of exercise tolerance, dyspnea or patient quality of life. However, the number of studies and patients included in these programs remains too limited in terms of modalities, duration, long-term effects, or adaptations in case of exacerbation to be the subject of recommendations. The potential of respiratory telerehabilitation justifies continuing clinical trials and experiments, which need to be coordinated with the interventions characterizing a conventional program. © 2021 SPLF	https://www.scopus.com/inward/record.uri?eid=2-s	Q2	Scopus	EXC_PubType
Phenotypes, Etiotypes, and Endotypes of Exacerbations of Chro	2023	Chronic obstructive pulmonary disease is a major health problem with a high prevalence, a rising incidence, and substantial morbidity and mortality. Its course is punctuated by acute episodes of increased respiratory symptoms, termed exacerbations of chronic obstructive pulmonary disease (ECOPD). ECOPD are important events in the natural history of the disease, as they are associated with lung function decline and prolonged negative effects on quality of life. The present-day therapy for ECOPD with short courses of antibiotics and steroids and escalation of bronchodilators has resulted in only modest improvements in outcomes. Recent data indicate that ECOPD are heterogeneous, raising the need to identify distinct etioendophenotypes, incorporating traits of the acute event and of patients who experience recurrent events, to develop novel and targeted therapies. These characterizations can provide a complete clinical picture, the severity of which will dictate acute pharmacological treatment, and may also indicate whether a change in maintenance therapy is needed to reduce the risk of future exacerbations. In this review we discuss the latest knowledge of ECOPD types on the basis of clinical presentation, etiology, natural history, frequency, severity, and biomarkers in an attempt to characterize these events. Copyright © 2023 by the American Thoracic Society.	https://www.scopus.com/inward/record.uri?eid=2-s	Q2	Scopus	EXC_Scope
Analysis of the effectiveness of remote intervention of patients af	2023	Objectives: The aim of the study was to verify the effectiveness of telemedicine in randomized controlled trials (RCTs) in terms of its influence on physical conditioning, quality of life, and health conditions in adults/elderly people affected by chronic diseases. Design: This was a systematic review and meta-analysis. Methods: The search covered RCTs published from 2011 to 2021 and was conducted using the PubMed, Embase, PEDro, Lilacs, and Cochrane Library databases, on volunteers of both sexes, that were rehabilitated, and/or monitored, and/or evaluated, specifically, via remote care. The extraction, quality of studies, and risk of bias were assessed using the RoB2 (risk of bias) tool, for analysis of the strength of evidence, the GRADE (Grading of Recommendations Assessment, Developing, and Evaluation) method was used, and for the preparation of meta-analysis was used at RevMan 5.4 (Review Manager) was used. Results: The database search identified 3949 potential articles for screening, 13 of which were eligible for the present systematic review, involving 1469 participants with chronic diseases (chronic obstructive pulmonary disease (COPD), asthma, heart failure, diabetes mellitus, and fibromyalgia). Through the meta-analysis, an advantage was identified for the remote intervention on physical conditioning ($p = 0.001$), with an estimated effect of 0.29 (0.11, 0.46) and on health conditions ($p = 0.0004$), –0.30 (–0.47, –0.14), while for quality of life, no significant difference was identified ($p = 0.90$), 0.01 (–0.13, 0.14). Conclusion: Telemedicine has clinical effectiveness for the outcome of physical conditioning and general health conditions in adults with chronic diseases when compared to usual care without face-to-face intervention. © The Author(s) 2023.	https://www.scopus.com/inward/record.uri?eid=2-s	Q2	Scopus	EXC_Scope
Digital Health Interventions for Depression and Anxiety Among P	2022	Background: Chronic conditions are characterized by their long duration (≥1 year), need for ongoing medical attention, and limitations in activities of daily living. These can often co-occur with depression and anxiety as common and detrimental comorbidities among the growing population living with chronic conditions. Digital health interventions (DHIs) hold promise in overcoming barriers to accessing mental health support for these individuals; however, the design and implementation of DHIs for depression and anxiety in people with chronic conditions are yet to be explored. Objective: This study aimed to explore what is known in the literature regarding DHIs for the prevention, detection, or treatment of depression and anxiety among people with chronic conditions. Methods: A scoping review of the literature was conducted using the Arksey and O'Malley framework. Searches of the literature published in 5 databases between 1990 and 2019 were conducted in April 2019 and updated in March 2021. To be included, studies must have described a DHI tested with, or designed for, the prevention, detection, or treatment of depression or anxiety in people with common chronic conditions (arthritis, asthma, diabetes mellitus, heart disease, chronic obstructive pulmonary disease, cancer, stroke, and Alzheimer disease or dementia). Studies were independently screened by 2 reviewers against the inclusion and exclusion criteria. Both quantitative and qualitative data were extracted, charted, and synthesized to provide a descriptive summary of the trends and considerations for future research. Results: Database searches yielded 11,422 articles across the initial and updated searches, 53 (0.46%) of which were included in this review. DHIs predominantly sought to provide treatment (44/53, 83%), followed by detection (5/53, 9%) and prevention (4/53, 8%). Most DHIs were focused on depression (36/53, 68%), guided (32/53, 60%), tailored to chronic physical conditions (19/53, 36%), and delivered through web-based platforms (20/53, 38%). Only 2 studies described the implementation of a DHI. Conclusions: As a growing research area, DHIs offer the potential to address the gap in care for depression and anxiety among people with chronic conditions; however, their implementation in standard care is scarce. Although stepped care has been identified as a promising model to implement efficacious DHIs, few studies have investigated the use of DHIs for depression and anxiety among chronic conditions using such models. In developing stepped care, we outlined DHI tailoring, guidance, and intensity as key considerations that require further research. © 2022 Journal of Medical Internet Research. All rights reserved.	https://www.scopus.com/inward/record.uri?eid=2-s	Q2	Scopus	EXC_PubType
Conceptualisations of COPD self-management: A narrative revie	2023	Aim: To examine how self-management is conceptualised in the research literature on chronic obstructive pulmonary disease (COPD). Methods: A narrative review was undertaken to search the research literature on COPD self-management. Ten databases (2000–2021) were searched for published texts. Sixty-two articles met the inclusion criteria. A thematic analysis was conducted of the literature. Results: Three conceptualisations of COPD self-management were identified: 1) a dominant medicocentric conceptualisation which represented self-management as medical in focus; 2) a less dominant experiential conceptualisation that viewed it as arising from the experiences of people living with COPD; and 3) a smaller body of literature that attempted to integrate medicocentric and experiential conceptualisations of self-management. Discussion: The dominance of the medicocentric conceptualisation of self-management and the polarisation of medicocentric and experiential perspectives were striking. An integrated conceptualisation of self-management has the potential to unite these competing perspectives and promote collaborative relationships between individuals and professionals, so long as the underlying values informing it are made explicit. However, there is a dearth of literature on this approach and it would benefit from more attention. Methods such as Co-production and the Personal Outcomes Approach offer the potential to support an integrated perspective in clinical practice. © The Author(s) 2022.	https://www.scopus.com/inward/record.uri?eid=2-s	Q2	Scopus	EXC_PubType
COPD-Related Anxiety: A Systematic Review of Patient Perspec	2023	Background: Anxiety in patients with chronic obstructive pulmonary disease (COPD) is prevalent but often unidentified and therefore not adequately managed. Clinicians find it difficult to detect anxiety symptoms and to differentiate subclinical anxiety from anxiety disorders, because of the considerable overlap between symptoms of COPD and anxiety. Purpose: We synthesized existing qualitative research on patients' experiences of COPD-related anxiety with the purpose of gaining a richer understanding and proposing a model of the construct. Methods: Searches for qualitative studies of patients' experiences of COPD-related anxiety were conducted independently by two authors in the databases of PubMed (MEDLINE), CINAHL (EBSCO), and PsycInfo (APA). English-language studies including patients diagnosed with COPD were reviewed, and data were analyzed using thematic analysis. Results: A total of 41 studies were included in the review. Four themes related to COPD-related anxiety were identified: initial events; internal maintaining factors; external maintaining factors; and behavioral maintaining factors. Based on the identified four themes, a conceptual model of COPD-related anxiety from the patient perspective was developed. Conclusion: A conceptual model of COPD-related anxiety from the patient perspective is now available, with the potential to inform future attempts at improving identification and management of COPD-related anxiety. Future research should focus on the development of a COPD-specific anxiety questionnaire containing domains that are relevant from the patient perspective. © 2023 Christiansen et al.	https://www.scopus.com/inward/record.uri?eid=2-s	Q2	Scopus	EXC_Scope

Digital health in chronic obstructive pulmonary disease	2023	Chronic obstructive pulmonary disease (COPD) can be prevented and treated through effective care, reducing exacerbations and hospitalizations. Early identification of individuals at high risk of COPD exacerbation is an opportunity for preventive measures. However, many patients struggle to follow their treatment plans because of a lack of knowledge about the disease, limited access to resources, and insufficient clinical support. The growth of digital health—which encompasses advancements in health information technology, artificial intelligence, telehealth, the Internet of Things, mobile health, wearable technology, and digital therapeutics—offers opportunities for improving the early diagnosis and management of COPD. This study reviewed the field of digital health in terms of COPD. The findings showed that despite significant advances in digital health, there are still obstacles impeding its effectiveness. Finally, we highlighted some of the major challenges and possibilities for developing and integrating digital health in COPD management. © 2023 The Authors. Chronic Diseases and Translational Medicine published by John Wiley & Sons, Ltd on behalf of Chinese Medical Association.	https://www.scopus.com/inward/record.uri?eid=2-s	Q2	Scopus	EXC_PubType
The Use of Gamification in the Self-Management of Patients Witl	2023	Background: Chronic disease self-management is a public health issue of worldwide concern, and gamification is an emerging strategy to improve patients' participation in chronic disease self-management. Some studies have summarized designs for the gamification of chronic disease self-management from the perspective of eHealth technology, but they have not mentioned differences in design methods, functions, and evaluation methods of gamified designs for self-management in different chronic diseases. Objective: This scoping review aims to synthesize the characteristics of realization forms, functions, and evaluation methods in chronic disease self-management gamification to improve self-management among the chronic disease population. Methods: We applied a methodological framework for scoping reviews and the PRISMA-ScR (Preferred Reporting Items for Systematic Reviews and Meta-Analyses extension for Scoping Reviews) checklist. As of January 7, 2023, we systematically searched 9 databases for relevant studies from January 2012 to December 2022. Related data were extracted based on the research questions. We calculated the frequencies, charted the quantitative data, and coded the extracted material for qualitative content analysis. Results: We retrieved 16,221 records, of which 70 (0.43%) met the eligibility criteria. In the included research, the target populations for gamified designs for self-management of chronic diseases included patients with stroke, cancer, diabetes, chronic obstructive pulmonary disease, coronary heart disease, obesity, and hypertension. Almost all studies mentioned technical support for gamification (68/70, 97%), mainly in the form of active video games (58/70, 83%); however, less than half of the studies mentioned the theoretical basis for gamification (31/70, 44%). There were 37 concepts or theories relevant to gamification design, most of which were in the field of psychology or were cross-disciplinary (n=33, 89%). Gamification for the self-management of chronic diseases has been widely recognized, including for promoting physical exercise and rehabilitation training (48/99, 48%), increasing initiative for symptom management (18/99, 18%), providing psychological support (14/99, 14%), improving cognitive function (12/99, 12%), and improving medication adherence (7/99, 7%). A total of 39 studies mentioned the gamification effect; however, we did not find a unified evaluation standard. Conclusions: This scoping review focuses on gamification designs for chronic disease self-management and summarizes the realization forms and functions of gamification in self-management for different patient populations. With practice in a gamified internet-based environment, patients can not only master the knowledge and skills of self-management in fascinating scenarios but also benefit from gaming experience and make better health-related decisions in real life. It is worth noting that a comprehensive evaluation of the users as well as a personalized and targeted intervention should be developed before gamification. © 2023 JMIR Publications Inc.. All Rights Reserved.	https://www.scopus.com/inward/record.uri?eid=2-s	Q2	Scopus	EXC_PubType
Towards the elimination of chronic obstructive pulmonary disease	2022	[No abstract available]	https://www.scopus.com/inward/record.uri?eid=2-s	Q2	Scopus	EXC_PubType
Investigating the prognostic value of digital mobility outcomes in	2023	Background: Reduced mobility is a central feature of COPD. Assessment of mobility outcomes that can be measured digitally (digital mobility outcomes (DMOs)) in daily life such as gait speed and steps per day is increasingly possible using devices such as pedometers and accelerometers, but the predictive value of these measures remains unclear in relation to key outcomes such as hospital admission and survival. Methods: We conducted a systematic review, nested within a larger scoping review by the MOBILISE-D consortium, addressing DMOs in a range of chronic conditions. Qualitative and quantitative analysis considering steps per day and gait speed and their association with clinical outcomes in COPD patients was performed. Results: 21 studies (6076 participants) were included. Nine studies evaluated steps per day and 11 evaluated a measure reflecting gait speed in daily life. Negative associations were demonstrated between mortality risk and steps per day (per 1000 steps) (hazard ratio (HR) 0.81, 95% CI 0.75–0.88, p<0.001), gait speed (<0.80 m·s^{-1}) (HR 3.55, 95% CI 1.72–7.36, p<0.001) and gait speed (per 1.0 m·s^{-1}) (HR 7.55, 95% CI 1.11–51.3, p=0.04). Fewer steps per day (per 1000) and slow gait speed (<0.80 m·s^{-1}) were also associated with increased healthcare utilisation (HR 0.80, 95% CI 0.72–0.88, p<0.001; OR 3.36, 95% CI 1.42–7.94, p=0.01, respectively). Available evidence was of low-moderate quality with few studies eligible for meta-analysis. Conclusion: Daily step count and gait speed are negatively associated with mortality risk and other important outcomes in people with COPD and therefore may have value as prognostic indicators in clinical trials, but the quantity and quality of evidence is limited. Larger studies with consistent methodologies are called for. © The authors 2023.	https://www.scopus.com/inward/record.uri?eid=2-s	Q2	Scopus	EXC_Scope
Effects of home-based telehealth on the physical condition and p	2023	Aims: This systematic review and meta-analysis aimed to evaluate the effects of home-based telehealth compared with usual care on six-minute walking distance (6MWD), health-related quality of life, anxiety and depression in patients with chronic obstructive pulmonary disease. Methods: We identified randomized controlled trials through a systematic multidatabase search. Titles and abstracts were assessed for relevance. Two authors independently extracted data and assessed the risk of bias and quality of evidence. Meta-analyses were conducted using Review Manager and Stata. Results: We included 32 randomized controlled trials (n = 5232). Devices used for home-based telehealth interventions included telephones, videos, and combined devices. The quality of the evidence was downgraded due to high risk of bias, imprecision, and inconsistency. Home-based telehealth significantly increased 6MWD by 35 m (SD = 30.42) and reduced symptom burden by 3 points (SD = −2.30) on the COPD assessment test compared with usual care. However, no significant differences in anxiety and depression were noted between the home-based telehealth group and the standard care group. In subgroup analysis, home-based telehealth significantly improved 6MWD and health status after 6–12 months and >12 months. Conclusion: Low quality evidence showed that home-based telehealth interventions reduce symptom burden and increase walking distance to a clinically meaningful extent in patients with COPD. However, no effects on depression and anxiety were observed. © 2022 John Wiley & Sons Australia, Ltd.	https://www.scopus.com/inward/record.uri?eid=2-s	Q2	Scopus	EXC_Scope
Pulmonary rehabilitation in a postcoronavirus disease 2019 worl	2022	Purpose of reviewPulmonary rehabilitation improves clinical outcomes in patients with chronic obstructive pulmonary disease (COPD). Traditional centre-based (in-person) pulmonary rehabilitation was largely shut down in response to the COVID-19 pandemic, forcing many centres to rapidly shift to remote home-based programs in the form of telerehabilitation (tele-pulmonary rehabilitation). This review summarizes the recent evidence for the feasibility and effectiveness of remote pulmonary rehabilitation programs, and their implications for the delivery of pulmonary rehabilitation in a postpandemic world.Recent findingsA number of innovative adaptations to pulmonary rehabilitation in response to COVID-19 have been reported, and the evidence supports tele-pulmonary rehabilitation as a viable alternative to traditional centre-based pulmonary rehabilitation. However, these studies also highlight the challenges that must be surmounted in order to see its widespread adoption.SummaryThere are outstanding questions regarding the optimal model for tele-pulmonary rehabilitation. In the post-COVID-19 world, a 'hybrid' model may be more desirable, with some components held in person and others via telehealth technology. This would be determined by the infrastructure and expertise of individual centres, and the needs of their patients. In order to achieve a truly patient-centred pulmonary rehabilitation program, high-quality studies addressing these outstanding questions, as well as multidisciplinary collaboration, are required. Copyright © 2021 Wolters Kluwer Health, Inc. All rights reserved.	https://www.scopus.com/inward/record.uri?eid=2-s	Q2	Scopus	EXC_Scope
The Role of Digital Tools in the Timely Diagnosis and Prevention	2022	Chronic obstructive pulmonary disease (COPD) is a chronic inflammatory disease of the airways and lung parenchyma with multiple systemic manifestations. Exacerbations of COPD are important events during the course of the disease, as they are associated with increased mortality, severe impairment of health-related quality of life, accelerated decline in lung function, significant reduction in physical activity, and substantial economic burden. Telemedicine is the use of communication technologies to transmit medical data over short or long distances and to deliver healthcare services. The need to limit in-person appointments during the COVID-19 pandemic has caused a rapid increase in telemedicine services. In the present review of the literature covering published randomized controlled trials reporting results regarding the use of digital tools in acute exacerbations of COPD, we attempt to clarify the effectiveness of telemedicine for identifying, preventing, and reducing COPD exacerbations and improving other clinically relevant outcomes, while describing in detail the specific telemedicine interventions used. © 2022 by the authors. Licensee MDPI, Basel, Switzerland.	https://www.scopus.com/inward/record.uri?eid=2-s	Q2	Scopus	EXC_PubType

Existential suffering in the day to day lives of those living with pal	2022	Background: The impact of living with palliative care needs arising from COPD disrupts an individual's existential situation. However, no comprehensive synthesis of existing research has been published to determine the presentation and impact of existential suffering. Aim: To provide a synthesis of existing evidence on existential suffering for those living with palliative care needs arising from COPD. Design: This is an integrative review paper, undertaken using the methodological approach developed by Soares and reported in accordance with PRISMA guidelines. Data analysis was undertaking using an integrated convergent synthesis approach. Data sources: Nine electronic databases were searched from April 2019 to December 2019. A second search was undertaken in January 2021 to identify recently published papers meeting the inclusion and exclusion criteria. No date restrictions were imposed. Only papers published in the English Language were considered for inclusion. Empirical research papers employing qualitative and/or quantitative methodologies and systematic literature reviews were included. Articles were accepted for inclusion if they discussed any component of existential suffering when living with COPD and palliative care needs. Results: Thirty-five papers were included within this review comprising of seven systematic reviews, 10 quantitative studies and 18 qualitative studies. The following themes relating to existential suffering were found: Liminality, Lamented Life, Loss of Personal Liberty, Life meaning and Existential isolation. The absence of life meaning, and purpose was of most importance to participants. Conclusions: This review suggests existential suffering is present and of significant impact within the daily lives of those living with palliative care needs arising from COPD. The absence of life meaning has the most significant impact. Further research is required to understand the essential components of an intervention to address existential suffering for this patient group, to ensure holistic palliative care delivery. © The Author(s) 2022.	https://www.scopus.com/inward/record.uri?eid=2-s	Q2	Scopus	EXC_Scope
Behaviour change and physical activity interventions for physical	2022	Background: Chronic obstructive pulmonary disease (COPD) is a complex respiratory disease and the third leading cause of death worldwide. Pulmonary rehabilitation is recognised as the gold standard of care in the management of COPD, however engagement with pulmonary rehabilitation is low and maintenance of a physically active lifestyle in community dwelling adults with COPD is poor. Supporting positive behaviour change in people with COPD could help to increase their engagement with physical activity. This systematic review will examine behaviour change and physical activity interventions delivered to community dwelling adults with COPD with the aim of increasing physical activity engagement. Interventions will be mapped against Michie's theoretical domains framework (TDF) to inform clinical practice and health policy. Methods: The following databases will be searched from inception until December 2021: Web of Science, CENTRAL, MEDLINE (via EBSCO), EMBASE, APA PsychINFO, CINAHL (via EBSCO), AMED, PROSPERO, Cochrane Airways Trials Register. Reference lists of the relevant studies and grey literature will be searched using Grey Literature Report, Open Grey and Google Scholar search engines. Relevant studies will be systematically reviewed and subject to quality appraisal to determine the impact of behaviour change and physical activity interventions on outcomes of community-dwelling adults with COPD. Interventions will be mapped to Michie's TDF and a narrative synthesis with respect to nature, effectiveness on target population and setting/environment will be provided. Findings will be reported in relation to the generalisability of the primary results and research question, and will include secondary findings on quality of life, self-reported participation in physical activity, exercise capacity, adverse events and intervention adherence. The review will be presented according to the PRISMA guidelines 2020. Conclusions: This systematic review is necessary to explain the impact of behaviour change and physical activity interventions on outcomes of community dwelling people with COPD. PROSPERO registration: CRD42021264965 (29.06.2021). Copyright: © 2022 Hanrahan C et al.	https://www.scopus.com/inward/record.uri?eid=2-s	Q2	Scopus	EXC_PubType
Non-pharmacological and non-invasive interventions for chronic	2023	Objectives: Chronic Obstructive Pulmonary Disease (COPD) is complicated by chronic pain. People with COPD report higher pain prevalence than the general population. Despite this, chronic pain management is not reflected in current COPD clinical guidelines and pharmacological treatments are often ineffective. We conducted a systematic review that aimed to establish the efficacy of existing non-pharmacological and non-invasive interventions on pain and identify behaviour change techniques (BCTs) associated with effective pain management. Methods: A systematic review was conducted with reference to Preferred Reporting Items for Systematic Review (PRISMA) [1], Systematic review without Meta analysis (SWIM) standards [2] and Grading of Recommendations Assessment, Development and Evaluation (GRADE) guidelines [3]. We searched 14 electronic databases for controlled trials of non-pharmacological and non-invasive interventions where the outcome measure assessed pain or contained a pain subscale. Results: Twenty-nine studies were identified involving 3,228 participants. Seven interventions reported a minimally important clinical difference in pain outcomes, although only two of these reached statistical significance ($p < 0.05$). A third study reported statistically significant outcomes, but this was not clinically significant ($p = 0.0273$). Issues with intervention reporting prevented identification of active intervention ingredients (i.e., BCTs). Conclusions: Pain appears to be a meaningful issue for many individuals with COPD. However, intervention heterogeneity and issues with methodological quality limit certainty about the effectiveness of currently available non-pharmacological interventions. An improvement in reporting is required to enable identification of active intervention ingredients associated with effective pain management. © 2023 The Authors	https://www.scopus.com/inward/record.uri?eid=2-s	Q2	Scopus	EXC_Scope
Pharmacological, Nutritional, and Rehabilitative Interventions to I	2022	Osteoporosis is a highly prevalent condition affecting a growing number of patients affected by chronic obstructive pulmonary disease (COPD), with crucial implications for risk of fragility fractures, hospitalization, and mortality. Several risk factors have been identified to have a role in osteoporosis development in COPD patients, including corticosteroid therapy, systemic inflammation, smoke, physical activity levels, malnutrition, and sarcopenia. In this scenario, a personalized multitarget intervention focusing on the pathological mechanisms underpinning osteoporosis is mandatory to improve bone health in these frail patients. Specifically, physical exercise, nutritional approach, dietary supplements, and smoke cessation are the cornerstone of the lifestyle approach to osteoporosis in COPD patients, improving not only bone health but also physical performance and balance. On the other hand, pharmacological treatment should be considered for both the prevention and treatment of osteoporosis in patients at higher risk of fragility fractures. Despite these considerations, several barriers still affect the integration of a personalized approach to managing osteoporosis in COPD patients. However, digital innovation solutions and telemedicine might have a role in optimizing sustainable networking between hospital assistance and community settings to improve bone health and reduce sanitary costs of the long-term management of COPD patients with osteoporosis. © 2022 by the authors.	https://www.scopus.com/inward/record.uri?eid=2-s	Q2	Scopus	EXC_Scope
COVID-19 infection in patients with chronic obstructive pulmonar	2022	Introduction: Patients with chronic obstructive pulmonary disease (COPD) are a vulnerable group in terms of the outcome of coronavirus infection in relation to their disease or its treatment, with a higher risk of developing serious complications compared to the healthy population. Aim: The aim of our summary study is to review the background and health outcomes of chronic obstructive pulmonary disease and COVID-19 infection in the presence of both diseases. Methods: Review of national and international medical databases (PubMed, MEDLINE, and MOB) with keywords COPD, COVID-19, disease risk, cause, prevention, complications, and prognosis. Results: Meta-analyses show that COPD is one of the most common underlying conditions in patients hospitalized for COVID-19. Such patients are five times more likely to develop a serious complication due to oxygen supply problems therefore they are more likely to be admitted to intensive care units, where they may require mechanical ventilation. In the case of underlying COPD, the usual care plan for COVID-19 infection should be followed, as well as all public health recommendations to minimize the risk of developing and transmitting COVID-19. Conclusion: Coronavirus infection is especially dangerous for COPD patients, who are much more likely to become seriously ill, so increased surveillance, prevention, early detection, adequate treatment and rehabilitation of the disease group are of paramount importance. © 2022 The Author(s).	https://www.scopus.com/inward/record.uri?eid=2-s	Q2	Scopus	EXC_PubType
COPD and multimorbidity: recognising and addressing a syndem	2023	Most patients with chronic obstructive pulmonary disease (COPD) have at least one additional, clinically relevant chronic disease. Those with the most severe airflow obstruction will die from respiratory failure, but most patients with COPD die from non-respiratory disorders, particularly cardiovascular diseases and cancer. As many chronic diseases have shared risk factors (eg, ageing, smoking, pollution, inactivity, and poverty), we argue that a shift from the current paradigm in which COPD is considered as a single disease with comorbidities, to one in which COPD is considered as part of a multimorbid state—with co-occurring diseases potentially sharing pathobiological mechanisms—is needed to advance disease prevention, diagnosis, and management. The term syndemics is used to describe the co-occurrence of diseases with shared mechanisms and risk factors, a novel concept that we propose helps to explain the clustering of certain morbidities in patients diagnosed with COPD. A syndemics approach to understanding COPD could have important clinical implications, in which the complex disease presentations in these patients are addressed through proactive diagnosis, assessment of severity, and integrated management of the COPD multimorbid state, with a patient-centred rather than a single-disease approach. © 2023 Elsevier Ltd	https://www.scopus.com/inward/record.uri?eid=2-s	Q2	Scopus	EXC_Scope

Design and delivery of home-based telehealth pulmonary rehabil	2022	Rationale. Home-based telehealth pulmonary rehabilitation (HTPR) for chronic obstructive pulmonary disease (COPD) is increasingly common partly due to the COVID-19 pandemic. However, optimal HTPR programming has not been described. This review provides a comprehensive overview of the design, delivery, and effects of HTPR for people with COPD. Methods: Relevant databases were searched to July 2021 for studies on adults with COPD utilizing information or communication technology to monitor or deliver HTPR. A meta-analysis was performed on a subset of randomized controlled trials. Results: Of 3124 records retrieved, 38 studies evaluating 1993 individuals with stable COPD (age 54–75 and FEV1 31–92% predicted) were included. Program components included exercise and education (n = 17) or exercise alone (n = 15) with in-clinic baseline assessments commonly conducted (n = 26). Few trials (n = 7) featured synchronous virtual exercise supervision. Aerobic exercise commonly involved walking (n = 14) and cycling (n = 11) and most programs included resistance training (n = 25). Exercise progressions and emergency action plans were inconsistently reported. Meta-analysis demonstrated HTPR was comparable to outpatient PR and had a greater effect than usual care for the modified Medical Research Council dyspnea scale (mean difference [95 % CI]: −0.49 [−0.77, −0.22], p < 0.01) and COPD Assessment Test score (−4.90 [−7.13, −2.67], p < 0.01). Neither HTPR nor outpatient PR impacted sedentary time or step count. Only 6% of studies reported race and no studies reported participant ethnicity. Conclusion: This review revealed the heterogeneity of HTPR program designs in COPD. HTPR programs had similar effects to outpatient PR programs and greater effects than usual care for people with COPD. © 2022 Elsevier B.V.	https://www.scopus.com/inward/record.uri?eid=2-s	Q2	Scopus	EXC_Scope
Evidence and User Considerations of Home Health Monitoring fc	2022	Background: Home health monitoring shows promise in improving health outcomes; however, navigating the literature remains challenging given the breadth of evidence. There is a need to summarize the effectiveness of monitoring across health domains and identify gaps in the literature. In addition, ethical and user-centered frameworks are important to maximize the acceptability of health monitoring technologies. Objective: This review aimed to summarize the clinical evidence on home-based health monitoring through a scoping review and outline ethical and user concerns and discuss the challenges of the current user-oriented conceptual frameworks. Methods: A total of 2 literature reviews were conducted. We conducted a scoping review of systematic reviews in Scopus, MEDLINE, Embase, and CINAHL in July 2021. We included reviews examining the effectiveness of home-based health monitoring in older adults. The exclusion criteria included reviews with no clinical outcomes and lack of monitoring interventions (mobile health, telephone, video interventions, virtual reality, and robots). We conducted a quality assessment using the Assessment of Multiple Systematic Reviews (AMSTAR-2). We organized the outcomes by disease and summarized the type of outcomes as positive, inconclusive, or negative. Second, we conducted a literature review including both systematic reviews and original articles to identify ethical concerns and user-centered frameworks for smart home technology. The search was halted after saturation of the basic themes presented. Results: The scoping review found 822 systematic reviews, of which 94 (11%) were included and of those, 23 (24%) were of medium or high quality. Of these 23 studies, monitoring for heart failure or chronic obstructive pulmonary disease reduced exacerbations (4/7, 57%) and hospitalizations (5/6, 83%); improved hemoglobin A1c (1/2, 50%); improved safety for older adults at home and detected changing cognitive status (2/3, 66%) reviews; and improved physical activity, motor control in stroke, and pain in arthritis in (3/3, 100%) rehabilitation studies. The second literature review on ethics and user-centered frameworks found 19 papers focused on ethical concerns, with privacy (12/19, 63%), autonomy (12/19, 63%), and control (10/19, 53%) being the most common. An additional 7 user-centered frameworks were studied. Conclusions: Home health monitoring can improve health outcomes in heart failure, chronic obstructive pulmonary disease, and diabetes and increase physical activity, although review quality and consistency were limited. Long-term generalized monitoring has the least amount of evidence and requires further study. The concept of trade-offs between technology usefulness and acceptability is critical to consider, as older adults have a hierarchy of concerns. Implementing user-oriented frameworks can allow long-term and larger studies to be conducted to improve the evidence base for monitoring and increase the receptiveness of clinicians, policy makers, and end users. © 2022 JMIR Human Factors. All rights reserved.	https://www.scopus.com/inward/record.uri?eid=2-s	Q2	Scopus	EXC_PubType
Closing the Gap between Inpatient and Outpatient Settings: Integ	2022	Pulmonary rehabilitation (PR) is a well-established intervention supported by strong evidence that is used to treat patients affected by chronic respiratory diseases. However, several barriers still affect its spreading in rehabilitation clinical practices. Although chronic respiratory diseases are common age-related disorders, there is still a gap of knowledge regarding the implementation of sustainable strategies integrating PR in the rehabilitation management of frail patients at high risk of respiratory complications. Therefore, in the present study, we characterized the effects of PR in frail patients, highlighting the evidence supporting its role in improving the complex rehabilitative management of these patients. Moreover, we propose a novel organizational model promoting PR programs for frail patients in both inpatient and outpatient settings. Our model emphasizes the role of interdisciplinary care, specifically tailored to patients and environmental characteristics. In this scenario, cutting-edge technology and telemedicine solutions might be implemented as safe and sustainable strategies filling the gap between inpatient and outpatient settings. Future research should focus on large-scale sustainable interventions to improve the quality of life and global health of frail patients. Moreover, evidence-based therapeutic paths should be promoted and taught in training courses promoting multiprofessional PR knowledge to increase awareness and better address its delivery in frail patients. © 2022 by the authors.	https://www.scopus.com/inward/record.uri?eid=2-s	Q2	Scopus	EXC_Scope
Early detection and prediction of acute exacerbation of chronic o	2023	Chronic Obstructive Pulmonary Disease (COPD) is characterized by persistent respiratory symptoms and airflow limitation. Acute exacerbation of COPD (AECOPD) is an acute worsening of respiratory symptoms, which needs additional treatment and can result in worsening health status, increasing risks of hospitalization and mortality. Therefore, it is necessary to early recognize and diagnose exacerbations of COPD. This review introduces the updated definition of COPD exacerbations, the current clinical assessment tools, and the current potential biomarkers. The application of mobile health care in COPD management for early identification and diagnosis is also included in this review. © 2023	https://www.scopus.com/inward/record.uri?eid=2-s	Q2	Scopus	EXC_PubType
Comprehensive care for people living with heart failure and chror	2022	Heart failure (HF) and chronic obstructive pulmonary disease (COPD) are the leading global epidemiological, clinical, social, and economic burden. Due to similar risk factors and overlapping pathophysiological pathways, the coexistence of these two diseases is common. People with severe COPD and advanced chronic HF (CHF) develop similar symptoms that aggravate if evoking mechanisms overlap. The coexistence of COPD and CHF limits the quality of life (QoL) and worsens symptom burden and mortality, more than if only one of them is present. Both conditions progress despite optimal, guidelines directed treatment, frequently exacerbate, and have a similar or worse prognosis in comparison with many malignant diseases. Palliative care (PC) is effective in QoL improvement of people with CHF and COPD and may be a valuable addition to standard treatment. The current guidelines for the management of HF and COPD emphasize the importance of early integration of PC parallel to disease-modifying therapies in people with advanced forms of both conditions. The number of patients with HF and COPD requiring PC is high and will grow in future decades necessitating further attention to research and knowledge translation in this field of practice. Care pathways for people living with concomitant HF and COPD have not been published so far. It can be hypothesized that overlapping of symptoms and similarity in disease trajectories allow to draw a model of care which will address symptoms and problems caused by either condition. Copyright © 2022 Kowalczys, Bohdan, Wilkowska, Pawłowska, Pawłowski, Janowiak, Jassem, Lelonek, Gruchała and Sobański.	https://www.scopus.com/inward/record.uri?eid=2-s	Q2	Scopus	EXC_Scope
A systematic review of effective strategies for chronic disease ma	2022	Large number of people with non-communicable diseases (NCDs) face barriers to adequate healthcare in humanitarian settings. We conducted a systematic literature review in MEDLINE/PubMed, Web of Science, EMBASE/DARE, Cochrane, and grey literature from 1990 to 2021 to evaluate effective strategies in addressing NCDs (diabetes, cardiovascular diseases, COPD, cancer) in humanitarian settings. From 2793 articles, 2652 were eliminated through title/abstract screening; 141 articles were reviewed in full; 93 were eliminated for not meeting full criteria. Remaining 48 articles were reviewed qualitatively to assess populations, settings, interventions, outcome, and efficacy and effectiveness; 38 studies addressed treatments, 9 prevention, and 7 epidemiology. Prevention studies broadly addressed capacity-building. Treatment and epidemiology studies largely addressed hypertension and diabetes. Interventions included web-based/mobile health strategies, pharmacy-level interventions, portable imaging, and capacity building including physical clinics, staff training, forging collaborations, guideline development, point-of-care labs, health promotion activities, EMR, and monitoring interventions. Collaboration between academia and implementing agencies was limited. Models of care were largely not well-described and varied between studies due to contextual constraints. Barriers to interventions included financial, logistical, organizational, sociocultural, and security. Cancer care is significantly understudied. Simplified care models adapted to contexts and program evaluations of implemented strategies could address gaps in applied research. Inherent challenges in humanitarian settings pose unavoidable perils to evidence generation which requires a shift in research mindset to match aspirations with practicality, research collaborations at the inception of projects, reworking of desired conventional level of research evidence considering resource-intense constraints (HR, time, cost), and adapted research tools, methods, and procedures. © 2022	https://www.scopus.com/inward/record.uri?eid=2-s	Q2	Scopus	EXC_Scope

Remote Monitoring for Prediction and Management of Acute Exa	2022	The progression of chronic obstructive pulmonary disease (COPD) is characterized by episodes of acute exacerbation (AECOPD) of symptoms, decline in respiratory function, and reduction in quality-of-life increasing morbi-mortality and often requiring hospitalization. Exacerbations can be triggered by environmental exposures, changes in lifestyle, and/or physiological and psychological factors to greater or lesser extents depending on the individual's COPD phenotype. The prediction and early detection of an exacerbation might allow patients and physicians to better manage the acute phase. We summarize the recent scientific data on remote telemonitoring (TM) for the prediction and management of acute exacerbations in COPD patients. We discuss the components of remote monitoring platforms, including the integration of environmental monitoring data; patient reported outcomes collected via interactive Smartphone apps, with data from wearable devices that monitor physical activity, heart rate, etc.; and data from medical devices such as connected non-invasive ventilators. We consider how telemonitoring and the deluge of data it potentially generates could be combined with electronic health records to provide personalized care and multi-disease management for COPD patients. © 2022 by the authors. Licensee MDPI, Basel, Switzerland.	https://www.scopus.com/inward/record.uri?eid=2-s	Q2	Scopus	EXC_PubType
Fatigue, post-exertional malaise and orthostatic intolerance: a ma	2022	INTRODUCTION: Rehabilitation focuses on impairments, activity limitations and participation restrictions being informed by the underlying health condition. in the current absence of direct "evidence on" rehabilitation interventions for people with post-coVid-19 condition (pcc), we can search and synthesize the indirect "evidence relevant to" coming from interventions effective for the symptoms of pcc in other health conditions. The World Health Organization (WHO) required this information to inform expert teams and provide specific recommendations in their Guidelines. With this overview of reviews with mapping, we aimed to synthesize in a map the cochrane evidence relevant to rehabilitation for fatigue, post-exertional malaise and orthostatic intolerance due to pcc. EVIDENCE ACQUISITION: We searched the last five years' Cochrane Systematic Review (CSRs) using the terms "fatigue," "orthostatic intolerance," "rehabilitation" and their synonyms in the cochrane library. We extracted and summarized the available evidence using a map. We grouped the included csrs for health conditions and interventions, indicating the effect and the quality of evidence. EVIDENCE SYNTHESIS: Out of 1397 CSRs published between 2016 and 2021, we included 32 for fatigue and 4 for exercise intolerance. They provided data from 13 health conditions, with cancer (11 studies), chronic obstructive pulmonary disease (7 studies), fibromyalgia (4 studies), and cystic fibrosis (3 studies) being the most studied. Effective interventions for fatigue included exercise training and physical activities, telerehabilitation and multicomponent and educational interventions. Effective interventions for exercise intolerance included combined aerobic/anaerobic training and integrated disease rehabilitation management. the overall quality of evidence was low to very low and moderate in very few cases. We did not identify CSRs that specifically addressed post-exertional malaise or orthostatic intolerance. CONCLUSIONS: These results are the first step of indirect evidence able to generate helpful hypotheses for clinical practice and future research. they served as the basis for the three recommendations on treatments for these pcc symptoms published in the current Who Guidelines for clinical practice. © 2022 THE AUTHORS.	https://www.scopus.com/inward/record.uri?eid=2-s	Q2	Scopus	EXC_Scope
eHealth in Self-Managing at a Distance Patients with COPD	2022	Worldwide, healthcare delivery for chronic diseases has been challenging due to the current SARS-COV-2 pandemic. The growing use of information and communication technologies via telehealth has gained popularity in all fields of medicine. In chronic respiratory diseases, self-management, defined as a structured but personalized multi-component intervention with the main goal of achieving healthy behavioral change, is an essential element of long-term care. Iterative interventions delivered by a well-trained health coach in order to empower and provide the patient with the tools and skills needed to adopt sustained healthy behaviors have proven to be effective in chronic obstructive pulmonary disease (COPD). Benefits have been shown to both improve patient quality of life and reduce acute exacerbation events and acute healthcare utilization. In COPD, the evidence so far has shown us that remote technologies such as telemonitoring or remote management may improve patient-reported outcomes and healthcare utilization. However, clear limitations are still present and questions remain unanswered. More and better designed studies are therefore necessary to define the place of eHealth in self-managing at a distance in patients with COPD. © 2022 by the authors. Licensee MDPI, Basel, Switzerland.	https://www.scopus.com/inward/record.uri?eid=2-s	Q2	Scopus	EXC_PubType
Methods to assess COPD medications adherence in healthcare (	2023	Background: The Global Initiative for Chronic Obstructive Lung Disease 2023 report recommends medication adherence assessment in COPD as an action item. Healthcare databases provide opportunities for objective assessments; however, multiple methods exist. We aimed to systematically review the literature to describe existing methods to assess adherence in COPD in healthcare databases and to evaluate the reporting of influencing variables. Method: We searched MEDLINE, Web of Science and Embase for peer-reviewed articles evaluating adherence to COPD medication in electronic databases, written in English, published up to 11 October 2022 (PROSPERO identifier CRD42022363449). Two reviewers independently conducted screening for inclusion and performed data extraction. Methods to assess initiation (dispensing of medication after prescribing), implementation (extent of use over a specific time period) and/or persistence (time from initiation to discontinuation) were listed descriptively. Each included study was evaluated for reporting variables with an impact on adherence assessment: inpatient stays, drug substitution, dose switching and early refills. Results: 160 studies were included, of which four assessed initiation, 135 implementation and 45 persistence. Overall, one method was used to measure initiation, 43 methods for implementation and seven methods for persistence. Most of the included implementation studies reported medication possession ratio, proportion of days covered and/or an alteration of these methods. Only 11% of the included studies mentioned the potential impact of the evaluated variables. Conclusion: Variations in adherence assessment methods are common. Attention to transparency, reporting of variables with an impact on adherence assessment and rationale for choosing an adherence cut-off or treatment gap is recommended. © 2023, European Respiratory Society. All rights reserved.	https://www.scopus.com/inward/record.uri?eid=2-s	Q2	Scopus	EXC_Scope
Clinical Approaches to Minimize Readmissions of Patients with C	2023	Chronic Obstructive Pulmonary Disease (COPD) is a progressive disease and also a lead-ing cause of morbidity and mortality worldwide. The frequent readmissions of patients with COPD may reduce lung function, mental health, and quality of life; it also increases the cost of treatment and mortality rate. Some common factors that may increase the readmission frequency of COPD patients include delay of diagnosis, advanced lung function decline, lack of adherence for COPD treatment, ineffective management of comorbidities, acute exacerbation or stable COPD, and infec-tions. However, these factors might be well controlled with appropriate approaches to minimize the readmission of patients with COPD. In this review, we propose a strategy with a seven-step approach to reduce the readmission in COPD patients, including early diagnosis of COPD, optimal treatment for stable COPD, targeted management of comorbidities, adequate therapy for acute ex-acerbations, individualized action plans for COPD patients, effective prevention of bacterial and viral infections, and adaptive program of pulmonary rehabilitation. Thus, implementing this approach may reduce the risk of readmission in patients with COPD. © 2023 Bentham Science Publishers.	https://www.scopus.com/inward/record.uri?eid=2-s	Q2	Scopus	EXC_Scope
Video-Based Educational Interventions for Patients With Chronic	2023	Background: With rising time constraints, health care professionals increasingly depend on technology to provide health advice and teach patients how to manage chronic disease. The effectiveness of video-based tools in improving knowledge, health behaviors, disease severity, and health care use for patients with major chronic illnesses is not well understood. Objective: The aim of this study was to assess the current literature regarding the efficacy of video-based educational tools for patients in improving process and outcome measures across several chronic illnesses. Methods: A systematic review was conducted using CINAHL and PubMed with predefined search terms. The search included studies published through October 2021. The eligible studies were intervention studies of video-based self-management patient education for an adult patient population with the following chronic health conditions: asthma, chronic kidney disease, chronic obstructive pulmonary disease, chronic pain syndromes, diabetes, heart failure, HIV infection, hypertension, inflammatory bowel disease, and rheumatologic disorders. The eligible papers underwent full extraction of study characteristics, study design, sample demographics, and results. Bias was assessed with the Cochrane risk-of-bias tools. Summary statistics were synthesized in Stata SE (StataCorp LLC). Data reporting was conducted per the PRISMA (Preferred Reporting Items for Systematic Reviews and Meta-Analyses) checklist. Results: Of the 112 studies fully extracted, 59 (52.7%) were deemed eligible for inclusion in this review. The majority of the included papers were superiority randomized controlled trials (RCTs; 39/59, 66%), with fewer pre-post studies (13/59, 22%) and noninferiority RCTs (7/59, 12%). The most represented conditions of interest were obstructive lung disease (18/59, 31%), diabetes (11/59, 19%), and heart failure (9/59, 15%). The plurality (28/59, 47%) of video-based interventions only occurred once and occurred alongside adjunct interventions that included printed materials, in-person counseling, and interactive modules. The most frequently studied outcomes were disease severity, health behavior, and patient knowledge. Video-based tools were the most effective in improving patient knowledge (30/40, 75%). Approximately half reported health behavior (21/38, 56%) and patient self-efficacy (12/23, 52%) outcomes were improved by video-based tools, and a minority of health care use (11/28, 39%) and disease severity (23/69, 33%) outcomes were improved by video-based tools. In total, 48% (22/46) of the superiority and noninferiority RCTs and 54% (7/13) of the pre-post trials had moderate or high risk of bias. Conclusions: There is robust evidence that video-based tools can improve patient knowledge across several chronic illnesses. These tools less consistently improve disease severity and health care use outcomes. Additional study is needed to identify features that maximize the efficacy of video-based interventions for patients across the spectrum of digital competencies to ensure optimized and equitable patient education and outcomes. © 2023 Journal of Medical Internet Research. All rights reserved.	https://www.scopus.com/inward/record.uri?eid=2-s	Q2	Scopus	EXC_Scope

Does Telemedicine Promote Physical Activity?	2022	Exercise capacity and physical activity are different concepts: the former refers to what an individual is capable of performing, while the latter refers to what the individual does in daily life. Low levels of physical activity (PA), which are very common in individuals with COPD, are associated with poor health outcomes, including increased symptoms, a more rapid decline in lung function, increased health care utilization and increased mortality risk. Because of these pervasive negative outcomes, attempts have been made to increase physical activity in individuals with COPD, hoping that success in this area will mitigate the negative effects of inactivity. Based on its ability to increase exercise capacity and reduce dyspnea in COPD and other chronic respiratory diseases, pulmonary rehabilitation (PR) would be expected also increase physical activity in these patients. However, accessibility to pulmonary rehabilitation programs is problematic in some areas, and studies testing its effectiveness in this outcome area have had inconsistent results. Using telehealth interventions using technology to provide medical care conveniently over a distance would have the benefit of reaching a larger proportion of individuals with COPD. A systematic review of clinical trials testing telehealth to promote physical activity had mixed results and low-certainty evidence, resulting in the inability to recommend any single type of intervention. Thus, using telehealth interventions to promote physical activity for individuals with chronic respiratory diseases, while promising, remains an area where future investigations are needed to identify its optimal modalities and clarify its benefits. © 2022 by the author. Licensee MDPI, Basel, Switzerland.	https://www.scopus.com/inward/record.uri?eid=2-s	Q2	Scopus	EXC_Scope
Embedding Pulmonary Rehabilitation for Chronic Obstructive Pu	2022	This paper presents a rapid review of the literature for the components, benefits, barriers, and facilitators of pulmonary rehabilitation for chronic obstructive pulmonary disease (COPD) people in-home and community-based settings. seventy-six studies were included: 57 home-based pulmonary rehabilitation (HBPR) studies and 19 community-based pulmonary rehabilitation (CBPR) studies. The benefits of HBPR on exercise capacity and health-related quality of life were observed in one-group studies, studies comparing HBPR to usual care, and studies comparing to hospital-based pulmonary rehabilitation, although the benefits were less pronounced in the latter. HBPR reduced hospital admissions compared to usual care and was more cost-effective than hospital pulmonary rehabilitation. Most HBPRs were designed with low-density or customized equipment, are minimally supervised, and have a low intensity of training. Although the HBPR has flexibility and no travel burden, participants with severe disease, physical frailty, and complex comorbidities had barriers to complying with HBPR. The telerehabilitation program, a facilitator for HBPR, is feasible and safe. CBPR was offered in-person supervision, despite being limited to physical therapists in most studies. Benefits in exercise capacity were shown in almost all studies, but the improvement in health-related quality of life was controversial. Patients reported the benefits that facilities where they attended the CBPR including social support and the presence of an instructor. They also reported barriers, such as poor physical condition, transport difficulties, and family commitments. Despite the minimal infrastructure offered, HBPR and CBPR are feasible, safe, and provide clinical benefits to patients with COPD. Home and community settings are excellent opportunities to expand the offer of pulmonary rehabilitation programs, as long as they follow protocols that ensure quality and safety following current guidelines. Copyright © 2022 de Oliveira, Pereira, Costa, de Souza Mendes, de Almeida, Velloso and Malaguti.	https://www.scopus.com/inward/record.uri?eid=2-s	Q2	Scopus	EXC_Scope
The Current and Future Role of Technology in Respiratory Care	2022	Over the past few decades, technology and improvements in artificial intelligence have dramatically changed major sectors of our day-to-day lives, including the field of healthcare. E-health includes a wide range of subdomains, such as wearables, smart-inhalers, portable electronic spirometers, digital stethoscopes, and clinical decision support systems. E-health has been consistently shown to enhance the quality of care, improve adherence to therapy, and allow early detection of worsening in chronic pulmonary diseases. The present review addresses the current and potential future role of major e-health tools and approaches in respiratory medicine, with the aim of providing readers with trustful and updated evidence to increase their awareness of the topic, and to allow them to optimally benefit from the latest innovation technology. Collected literature evidence shows that the potential of technology tools in respiratory medicine mainly relies on three fundamental interactions: between clinicians, between clinician and patient, and between patient and health technology. However, it would be desirable to establish widely agreed and adopted standards for conducting trials and reporting results in this area, as well as to take into proper consideration potentially relevant pitfalls related to privacy protection and compliance with regulatory procedures. © 2022, The Author(s).	https://www.scopus.com/inward/record.uri?eid=2-s	Q2	Scopus	EXC_PubType
Health literacy in asthma and chronic obstructive pulmonary dise	2022	Respiratory self-care places considerable demands on patients with chronic airways disease (AD), as they must obtain, understand and apply information required to follow their complex treatment plans. If clinical and lifestyle information overwhelms patients' HL capacities, it reduces their ability to self-manage. This review outlines important societal, individual, and healthcare system factors that influence disease management and outcomes among patients with asthma and chronic obstructive pulmonary disease (COPD)—the two most common ADs. For this review, we undertook a comprehensive literature search, conducted reference list searches from prior HL-related publications, and added insights from international researchers and scientists with an interest in HL. We identified methodological limitations in currently available HL measurement tools in respiratory care. We also summarized the issues contributing to low HL and system-level cultural incompetency that continue to be under-recognized in AD management and contribute to suboptimal patient outcomes. Given that impaired HL is not commonly recognized as an important factor in AD care, we propose a three-level patient-centered model (strategies) designed to integrate HL considerations, with the goal of enabling health systems to enhance service delivery to meet the needs of all AD patients. © 2022, The Author(s).	https://www.scopus.com/inward/record.uri?eid=2-s	Q2	Scopus	EXC_Scope
Health Disparities: Interventions for Pulmonary Disease – A Narr	2023	There is expansive literature documenting the presence of health disparities, but there are disproportionately few studies describing interventions to reduce disparity. In this narrative review, we categorize interventions to reduce health disparity in pulmonary disease within the US health care system to support future initiatives to reduce disparity. We identified 211 articles describing interventions to reduce disparity in pulmonary disease related to race, income, or sex. We grouped the studies into the following four categories: biologic, educational, behavioral, and structural. We identified the following five main themes: (1) there were few interventional trials compared with the breadth of studies describing health disparities, and trials involving patients with asthma who were Black, low income, and living in an urban setting were overrepresented; (2) race or socioeconomic status was not an effective marker of individual pharmacologic treatment response; (3) telehealth enabled scaling of care, but more work is needed to understand how to leverage telehealth to improve outcomes in marginalized communities; (4) future interventions must explicitly target societal drivers of disparity, rather than focusing on individual behavior alone; and (5) individual interventions will only be maximally effective when specifically tailored to local needs. Much work has been done to catalog health disparities in pulmonary disease. Notable gaps in the identified literature include few interventional trials, the need for research in diseases outside of asthma, the need for high quality effectiveness trials, and an understanding of how to implement proven interventions balancing fidelity to the original protocol and the need to adapt to local barriers to care. © 2023 American College of Chest Physicians	https://www.scopus.com/inward/record.uri?eid=2-s	Q2	Scopus	EXC_Scope

Educational interventions for health professionals managing chro	2022	Background: Chronic obstructive pulmonary disease (COPD) is a common, preventable and treatable health condition. COPD is associated with substantial burden on morbidity, mortality and healthcare resources. Objectives: To review existing evidence for educational interventions delivered to health professionals managing COPD in the primary care setting. Search methods: We searched the Cochrane Airways Trials Register from inception to May 2021. The Register includes records from the Cochrane Central Register of Controlled Trials (CENTRAL), MEDLINE, Embase, Cumulative Index to Nursing and Allied Health Literature (CINAHL), Allied and Complementary Medicine Database (AMED) and PsycINFO. We also searched online trial registries and reference lists of included studies. Selection criteria: We included randomised controlled trials (RCTs) and cluster-RCTs. Eligible studies tested educational interventions aimed at any health professionals involved in the management of COPD in primary care. Educational interventions were defined as interventions aimed at upskilling, improving or refreshing existing knowledge of health professionals in the diagnosis and management of COPD. Data collection and analysis: Two review authors independently reviewed abstracts and full texts of eligible studies, extracted data and assessed the risk of bias of included studies. We conducted meta-analyses where possible and used random-effects models to yield summary estimates of effect (mean differences (MDs) with 95% confidence intervals (CIs)). We performed narrative synthesis when meta-analysis was not possible. We assessed the overall certainty of evidence for each outcome using Grades of Recommendation, Assessment, Development and Evaluation (GRADE). Primary outcomes were: 1) proportion of COPD diagnoses confirmed with spirometry; 2) proportion of patients with COPD referred to, participating in or completing pulmonary rehabilitation; and 3) proportion of patients with COPD prescribed respiratory medication consistent with guideline recommendations. Main results: We identified 38 studies(22 cluster-RCTs and 16 RCTs) involving 4936 health professionals (reported in 19/38 studies) and 71,085 patient participants (reported in 25/38 studies). Thirty-six included studies evaluated interventions versus usual care; seven studies also reported a comparison between two or more interventions as part of a three- to five-arm RCT design. A range of simple to complex interventions were used across the studies, with common intervention features including education provided to health professionals via training sessions, workshops or online modules (31 studies), provision of practice support tools, tool kits and/or algorithms (10 studies), provision of guidelines (nine studies) and training on spirometry (five studies). Health professionals targeted by the interventions were most commonly general practitioners alone (20 studies) or in combination with nurses or allied health professionals (eight studies), and the majority of studies were conducted in general practice clinics. We identified performance bias as high risk for 33 studies. We also noted risk of selection, detection, attrition and reporting biases, although to a varying extent across studies. The evidence of efficacy was equivocal for all the three primary endpoints evaluated: 1) proportion of COPD diagnoses confirmed with spirometry (of the four studies that reported this outcome, two supported the intervention); 2) proportion of patients with COPD who are referred to, participate in or complete pulmonary rehabilitation (of the four studies that reported this outcome, two supported the intervention); and 3) proportion of patients with COPD prescribed respiratory medications consistent with guideline recommendations (12 studies reported this outcome, the majority evaluated multiple drug classes and reported a mixed effect). Additionally, the low quality of evidence and potential risk of bias make the interpretation more difficult. Moderate-quality evidence (downgraded due to risk of bias concerns) suggests that educational interventions for health professionals probably improve the proportion of patients with COPD vaccinated against influenza (three studies) and probably have little impact on the proportion of patients vaccinated against pneumococcal infection (two studies). Low-quality evidence suggests that educational interventions for health professionals may have little or no impact on the frequency of COPD exacerbations (10 studies). There was a high degree of heterogeneity in the reporting of health-related quality of life (HRQoL). Low-quality evidence suggests that educational interventions for health professionals may have little or no impact on HRQoL overall, and when using the COPD-specific HRQoL instrument, the St George's Respiratory Questionnaire (at six months MD 0.87, 95% CI -2.51 to 4.26; 2 studies, 406 participants, and at 12 months MD -0.43, 95% CI -1.52 to 0.67, 4 studies, 1646 participants; reduction in score indicates better health). Moderate-quality evidence suggests that educational interventions for health professionals may improve patient satisfaction with care (one study). We identified no studies that reported adverse outcomes. Authors' conclusions: The evidence of efficacy was equivocal for educational interventions for health professionals in primary care on the proportion of COPD diagnoses confirmed with spirometry, the proportion of patients with COPD who participate in pulmonary rehabilitation, and the proportion of patients prescribed guideline-recommended COPD respiratory medications. Educational interventions for health professionals may improve influenza vaccination rates among patients with COPD and patient satisfaction with care. The quality of evidence for most outcomes was low or very low due to heterogeneity and methodological limitations of the studies included in the review, which means that there is uncertainty about the benefits of any currently published educational interventions for healthcare professionals to improve COPD management in primary care. Further well-designed RCTs are needed to investigate the effects of educational interventions delivered to health professionals managing COPD in the primary care setting. Copyright © 2022 The Cochrane Collaboration. Published by John Wiley & Sons, Ltd.	https://www.scopus.com/inward/record.uri?eid=2-s	Q2	Scopus	EXC_Scope
A systematic review of methods of scoring inhaler technique	2023	Many inhaler devices are currently used in clinical practice to deliver medication, with each inhaler device offering different benefits to overcome technique issues. Inhaler technique remains poor, contributing to reduced airway drug deposition and consequently poor disease control. Scoring inhaler technique has been used within research as an outcome measure of inhaler technique assessment, and this systematic review collates and evaluates these scoring methods. The review protocol was prospectively registered in PROSPERO (CRD42020218869). A total of 172 articles were screened with 77 included, and the results presented using narrative synthesis due to the heterogeneity of the study design and data. The most frequently used scoring method awarded one point per step in the inhaler technique checklist and was included in 59/77 (77%) of articles; however limited and varied guidance was provided for score interpretation. Other inhaler technique scoring methods included grading the final inhaler technique score, expressing the total score as a percentage/ratio, deducting points from the final score when errors were made, and weighting steps within the checklist depending on how crucial the step was. Vast heterogeneity in the number of steps and content in the inhaler technique checklists was observed across all device types (range 5–19 steps). Only 4/77 (5%) of the inhaler technique measures had undertaken fundamental steps required in the scale development process for use in real world practice. This review demonstrates the demand for a tool that measures inhaler technique and highlights the current unmet need for one that has undergone validation. © 2023 The Authors	https://www.scopus.com/inward/record.uri?eid=2-s	Q2	Scopus	EXC_Scope
Virtual reality for COPD rehabilitation: a technological perspective	2022	Virtual Reality (VR) is a promising technology for implementing personalized, motivating and controlled rehabilitation scenarios. Although its clear potential benefits, VR has been poorly investigated in pulmonary rehabilitation. This review analyses the state of the art, by searching the scientific and grey literature, regarding the use of VR for the rehabilitation of patients with chronic obstructive pulmonary disease, providing a technological perspective. First, the main characteristics of the included systems are presented in terms of visualization devices, way of interaction and type of feedback they provide. Then, results of the selected studies are reported considering feasibility, safety, usability and user experience as outcomes. Finally, the main findings are discussed and future directions for research are outlined. © 2020 Sociedade Portuguesa de Pneumologia	https://www.scopus.com/inward/record.uri?eid=2-s	Q2	Scopus	EXC_Scope
Technological features of smartphone apps for physical activity p	2023	Introduction: Low physical activity (PA) levels have a negative impact on the health status of patients with Chronic Obstructive Pulmonary Disease (COPD). Smartphone applications (apps) focused on PA promotion may mitigate this problem; however, their effectiveness depends on patient adherence, which can be influenced by the technological features of the apps. This systematic review identified the technological features of smartphone apps aiming to promote PA in patients with COPD. Methods: A literature search was performed in the databases ACM Digital Library, IEEE Xplore, PubMed, Scopus and Web of Science. Papers including the description of a smartphone app for PA promotion in patients with COPD were included. Two researchers independently selected studies and scored the apps features based on a previously developed framework (38 possible features). Results: Twenty-three studies were included and 19 apps identified, with an average of 10 technological features implemented. Eight apps could be connected to wearables to collect data. The categories 'Measuring and monitoring' and 'Support and Feedback' were present in all apps. Overall, the most implemented features were 'progress in visual format' (n = 13), 'advice on PA' (n = 14) and 'data in visual format' (n = 10). Only three apps included social features, and two included a web-based version of the app. Conclusions: The existing smartphone apps include a relatively small number of features to promote PA, which are mostly related to monitoring and providing feedback. Further research is warranted to explore the relationship between the presence/absence of specific features and the impact of interventions on patients' PA levels. © 2023 Sociedade Portuguesa de Pneumologia	https://www.scopus.com/inward/record.uri?eid=2-s	Q2	Scopus	EXC_Scope

A scoping review of co-creation practice in the development of nc	2023	Background: Incorporating co-creation processes may improve the quality of outcome interventions. However, there is a lack of synthesis of co-creation practices in the development of Non-Pharmacological Interventions (NPIs) for people with Chronic Obstructive Pulmonary Disease (COPD), that could inform future co-creation practice and research for rigorously improving the quality of care. Objective: This scoping review aimed to examine the co-creation practice used when developing NPIs for people with COPD. Methods: This review followed Arksey and O'Malley scoping review framework and was reported according to the PRISMA-ScR framework. The search included PubMed, Scopus, CINAHL, and Web of Science Core Collection. Studies reporting on the process and/or analysis of applying co-creation practice in developing NPIs for people with COPD were included. Results: 13 articles complied with the inclusion criteria. Limited creative methods were reported in the studies. Facilitators described in the co-creation practices included administrative preparations, diversity of stakeholders, cultural considerations, employment of creative methods, creation of an appreciative environment, and digital assistance. Challenges around the physical limitations of patients, the absence of key stakeholder opinions, a prolonged process, recruitment, and digital illiteracy of co-creators were listed. Most of the studies did not report including implementation considerations as a discussion point in their co-creation workshops. Conclusion: Evidence-based co-creation in COPD care is critical for guiding future practice and improving the quality of care delivered by NPIs. This review provides evidence for improving systematic and reproducible co-creation. Future research should focus on systematically planning, conducting, evaluating, and reporting co-creation practices in COPD care. © 2023 The Author(s)	https://www.scopus.com/inward/record.uri?eid=2-s	Q2	Scopus	EXC_PubType
A systematic review of behaviour change techniques in pharmac	2023	Background: Self-management interventions often employ behaviour change techniques in order to produce desired target behaviours that are necessary for day-to-day living with a chronic disease. Despite the large number of self-management interventions for patients with chronic obstructive pulmonary disease (COPD), previously reported interventions have been typically delivered by healthcare providers other than the pharmacist. Objective: This systematic review examined the components of pharmacists-delivered COPD self-management interventions according to an established taxonomy of behaviour change techniques (BCTs). Methods: A systematic search was conducted on PubMed, ScienceDirect, OVID, and Google Scholar from January 2011 to December 2021 for studies of pharmacist-delivered self-management interventions in COPD patients. Results: A total of seventeen studies of intervention were eligible for inclusion in the narrative review. Interventions were educational and were delivered individually and face-to-face for the first session. Across studies, pharmacists spent an average of 35 min on the first meeting and had an average of 6 follow-up sessions. Recurrent BCTs in pharmacist interventions were "Information on the health consequence", "Feedback on behaviour", "Instruction on how to perform a behaviour", "Demonstration of the behaviour" and "Behavioural practice/rehearsal". Conclusions: Pharmacists have provided interventions towards improving health behaviours, especially on adherence and usage of inhaler devices for patients with COPD. Future self-management interventions should be designed using the identified BCTs for the improvement of COPD self-management and disease outcomes. © 2023 Elsevier Inc.	https://www.scopus.com/inward/record.uri?eid=2-s	Q2	Scopus	EXC_Scope
Consideration of sex, gender, or age on outcomes of digital techr	2023	Background: Several systematic reviews have addressed digital technology use for treatment and monitoring of chronic obstructive pulmonary disease (COPD). Objective: This study aimed to assess if systematic reviews considered the effects of sex, gender, or age on the outcomes of digital technologies for treatment and monitoring of COPD through an overview of such systematic reviews. The objectives of this overview were to (1) describe the definitions of sex or gender used in reviews; (2) determine whether the consideration of sex, gender, or age was planned in reviews; (3) determine whether sex, gender, or age was reported in review results; (4) determine whether sex, gender, or age was incorporated in implications for clinical practice in reviews; and (5) create an evidence map for development of individualized clinical recommendations for COPD based on sex, gender, or age diversity. Methods: MEDLINE, the Cochrane Library, Epistemonikos, Web of Science, and the bibliographies of the included systematic reviews were searched to June 2022. Inclusion was based on the PICOS framework: (1) population (COPD), (2) intervention (any digital technology), (3) comparison (any), (4) outcome (any), and (5) study type (systematic review). Studies were independently selected by 2 authors based on title and abstract and full-Text screening. Data were extracted by 1 author and checked by another author. Data items included systematic review characteristics; PICOS criteria; and variables related to sex, gender, or age. Systematic reviews were appraised using A Measurement Tool to Assess Systematic Reviews, version 2 (AMSTAR 2). Data were synthesized using descriptive statistics. Results: Of 1439 records, 30 systematic reviews published between 2010 and 2022 were included in this overview. The confidence in the results of 25 of the 30 (83%) reviews was critically low according to AMSTAR 2. The reviews focused on user outcomes that potentially depend on sex, gender, or age, such as efficacy or effectiveness (25/30, 83%) and acceptance, satisfaction, or adherence (3/30, 10%) to digital technologies for COPD. Reviews reported sex or gender (19/30 systematic reviews) or age (25/30 systematic reviews) among primary study characteristics. However, only 1 of 30 reviews included age in a subgroup analysis, and 3 of 30 reviews identified the effects of sex, gender, or age as evidence gaps. Conclusions: This overview shows that the effects of sex, gender, or age were rarely considered in 30 systematic reviews of digital technologies for COPD treatment and monitoring. Furthermore, systematic reviews did not incorporate sex, gender, nor age in their implications for clinical practice. We recommend that future systematic reviews should (1) evaluate the effects of sex, gender, or age on the outcomes of digital technologies for treatment and monitoring of COPD and (2) better adhere to reporting guidelines to improve the confidence in review results. © 2023 Journal of Medical Internet Research. All rights reserved.	https://www.scopus.com/inward/record.uri?eid=2-s	Q2	Scopus	EXC_Scope
Content, Mechanism, and Outcome of Effective Telehealth Soluti	2023	Telehealth (TH) solutions for Chronic Obstructive Pulmonary Disease (COPD) are promising behavioral therapeutic interventions and can help individuals living with COPD to improve their health status. The linking content, mechanism, and outcome of TH interventions reported in the literature related to COPD care are unknown. This paper aims to summarize the existing literature about structured TH solutions in COPD care. We conducted an electronic search of the literature related to TH solutions for COPD management up to October 2023. Thirty papers presented TH solutions as an innovative treatment to manage COPD. TH and digital health solutions are used interchangeably in the literature, but both have the potential to improve care, accessibility, and quality of life. To date, current TH solutions in COPD care have a variety of content, mechanisms, and outcomes. TH solutions can enhance education as well as provide remote monitoring. The content of TH solutions can be summarized as symptom management, prompt physical activity, and psychological support. The mechanism of TH solutions is manipulated by factors such as content, mode of delivery, strategy, and intensity. The most common outcome measures with TH solutions were adherence to treatment, health status, and quality of life. Implementing effective TH with a COPD care bundle must consider important determinants such as patient's needs, familiarity with the technology, healthcare professional support, and data privacy. The development of effective TH solutions for COPD management also must consider patient engagement as a positive approach to optimizing implementation and effectiveness. © 2023 by the author.	https://www.scopus.com/inward/record.uri?eid=2-s	Q2	Scopus	EXC_PubType
The Role of Palliative Care in COPD	2022	COPD is the fourth leading cause of death in the United States and is a serious respiratory illness characterized by years of progressively debilitating breathlessness, high prevalence of associated depression and anxiety, frequent hospitalizations, and diminished well-being. Despite the potential to confer significant quality-of-life benefits for patients and their care partners and to improve end-of-life (EOL) care, specialist palliative care is rarely implemented in COPD, and when initiated, it often occurs only at the very EOL. Primary palliative care delivered by frontline clinicians is a feasible model, but is not integrated routinely in COPD. In this review, we discuss the following: (1) the role of specialist and primary palliative care for patients with COPD and the case for earlier integration into routine practice; (2) the domains of the National Consensus Project Guidelines for Quality Palliative Care applied to people living with COPD and their care partners; and (3) triggers for initiating palliative care and practical ways to implement palliative care using case-based examples. This review solidifies that palliative care is much more than hospice and EOL care and demonstrates that early palliative care is appropriate at any point during the COPD trajectory. We emphasize that palliative care should be integrated long before the EOL to provide comprehensive support for patients and their care partners and to prepare them better for the EOL. © 2021 American College of Chest Physicians	https://www.scopus.com/inward/record.uri?eid=2-s	Q2	Scopus	EXC_Scope

Technological advances and digital solutions to improve quality c	2023	Background: Several technological advances and digital solutions have been proposed in the recent years to face the emerging need for tele-monitoring older adults with Chronic Obstructive Pulmonary Disease (COPD). However, several challenges have negatively influenced an evidence-based approach to improve Health-Related Quality of Life (HR-QoL) in these patients. Aim: To assess the effects of tele-monitoring devices on HR-QoL in older adults with COPD. Methods: On November 11, 2022, PubMed, Scopus, Web of Science, and Cochrane were systematically searched for randomized controlled trials (RCTs) consistent with the following PICO model: older people with COPD as participants, tele-monitoring devices as intervention, any comparator, and HR-QoL as the primary outcome. Functional outcomes, sanitary costs, safety, and feasibility were considered secondary outcomes. The quality assessment was performed in accordance with the Jadad scale. Results: A total of 1845 records were identified and screened for eligibility. As a result, 5 RCTs assessing 584 patients (423 males and 161 females) were included in the systematic review. Tele-monitoring devices were ASTRI telecare system, WeChat social media, Pedometer, SweetAge monitoring system, and CHROMED monitoring platform. No significant improvements in terms of HR-QoL were reported in the included studies. However, positive effects were shown in terms of the number of respiratory events and hospitalization in patients telemonitored by SweetAge system and CHROMED platform. Discussion: Although a little evidence supports the role of tele-monitoring devices in improving HR-QoL in older patients, positive effects were reported in COPD exacerbation consequences and functional outcomes. Conclusion: Tele-monitoring solutions might be considered as sustainable strategies to implement HR-QoL in the long-term management of older patients with COPD. © 2023, The Author(s), under exclusive licence to Springer Nature Switzerland AG.	https://www.scopus.com/inward/record.uri?eid=2-s	Q2	Scopus	EXC_Scope
The Impact of eHealth Interventions on the Improvement of Self-	2022	Promoting self-care is one of the most promising strategies for managing chronic conditions. This overview aimed to investigate the effectiveness of eHealth interventions at improving self-care in patients with type-2 diabetes mellitus, cardiovascular disease, and chronic obstructive pulmonary disease when compared to standard care. We carried out a review of systematic reviews on PubMed, Scopus, Cochrane, PsychInfo, and CINAHL. AMSTAR-2 was used for quality appraisal. Eight systematic reviews (six with meta-analysis) were included, involving a total of 41,579 participants. eHealth interventions were categorized into three subgroups: (i) reminders via messaging apps, emails, and apps; (ii) telemonitoring and online operator support; (iii) internet and web-based educational programs. Six systematic reviews showed an improvement in self-care measurements through eHealth interventions, which also led to a better quality of life and clinical outcomes (HbA1C, blood pressure, hospitalization, cholesterol, body weight). This overview provided some implications for practice and research: eHealth is effective in increasing self-care in chronic patients; however, it is required to designate the type of eHealth intervention based on the needed outcome (e.g., implementing telemonitoring to increase self-monitoring of blood pressure). In addition, there is a need to standardize self-care measures through increased use of validated assessment tools. © 2022 by the authors.	https://www.scopus.com/inward/record.uri?eid=2-s	Q2	Scopus	EXC_PubType
The Voice of Patients Really Matters: Using Patient-Reported Ou	2023	Background: The aim of the study is to analyze the prevalence of using patients’ reported outcomes measures and experiences (PROMs and PREMs) in relation to integrated care (IC). Material and methods: To select eligible studies (<10 years, full-text), PubMed was used. The general subject of the articles referring to the type of disease was indicated on the basis of a review of all full-text publications discussing the effectiveness of IC (N = 6518). The final search included MeSH headings related to outcomes measures and IC. Full-text screening resulted in including 73 articles (23 on COPD, 40 on diabetes/obesity and 10 on depression) with 93.391 participants. Results: Analysis indicated that authors used multiple outcome measures, with 54.8% of studies including at least one patient reported. PROMs were more often used than PREMs. Specific (disease or condition/dimension) outcome measures were reported more often than general, especially those dedicated to self-assessment of health in COPD and depression. PROMs and PREMs were most commonly used in studies from the USA and Netherlands. Conclusion: Using PROMS/PREMS is becoming more popular, although it is varied, both due to the place of research and type of disease. © 2022 by the author.	https://www.scopus.com/inward/record.uri?eid=2-s	Q2	Scopus	EXC_Scope
A critical interpretive synthesis of the lived experiences and healt	2023	Aims: To determine the lived experiences of people with COPD who isolated at home during the coronavirus disease 2019 (COVID-19) pandemic, and explore how these experiences affected health and patient-reported outcomes. Methods: Keyword searches were performed in five bibliographic databases. Critical interpretative synthesis (CIS) methods were used to interrogate and understand patterns across studies. Results: 23 studies were identified; three employed qualitative methods and 20 quantitative methods. Application of CIS methods highlighted a core synthetic concept that appeared to underpin experiences and outcomes, that of a heightened perception of risk. Using the Risk Perception Model as a framework, we found that cognitive factors such as knowledge of underlying health status and the transmissibility of COVID-19; experiential factors including previous episodes of breathlessness and hospitalisation; and sociocultural factors such as access to trusted sources of information, influenced perceptions of risk. In turn, this influenced behaviour, which translated to outcomes such as reduced hospitalisations, deconditioning and social isolation as people avoided “high-risk” situations and settings. Conclusions: Patients with COPD who isolated at home during the COVID-19 pandemic had a heightened perception of risk which was influenced by cognitive, experiential and sociocultural factors. The consequences of this were varied and included both positive (reduced exacerbations and hospitalisations) and negative (social isolation, deconditioning, diminished capacity for self-care) outcomes. Understanding risk and the impacts it can have could help clinicians to support people with COPD return to their pre-pandemic way of living and enable better communication of ongoing risk from respiratory viral illness. © The authors 2023.	https://www.scopus.com/inward/record.uri?eid=2-s	Q2	Scopus	EXC_Scope
Smart dry powder inhalers and intelligent adherence manageme	2022	Adherence to inhaled treatments is a complex challenge for patients with chronic obstructive pulmonary disease (COPD) and asthma, it not only involves following the prescribed treatment plans but also administering the medications correctly. When using a dry powder inhaler (DPI), the inhalation flow is particularly critical. Patients frequently fail to use a rapid enough onset and fast enough inhalation when using DPIs. At the same time, there is increasing pressure on physicians to switch patients to DPIs, to minimise the environmental impact of pMDI propellants. This makes it critical to understand whether a patient will maintain or improve disease control by using their new inhaler correctly. However, it is challenging for health care professionals to understand how a patient behaves away from the clinic. Therefore, it would be beneficial to obtain real-world data through the use of monitoring tools, i.e., “smart inhalers”. This paper reviews the technologies used to monitor DPIs, how effective they have been in a clinical setting, and how well these have been adopted by patients and health care providers. © 2022	https://www.scopus.com/inward/record.uri?eid=2-s	Q2	Scopus	EXC_Scope
Telerehabilitation as a Form of Pulmonary Rehabilitation in Chror	2022	Introduction: Tele-rehabilitation is increasingly used to deliver pulmonary rehabilitation. The aim of this systematic review was to compare the effect between tele-pulmonary rehabilitation and classical supervised pulmonary rehabilitation. Method: Three databases were analysed (PubMed, PEDro, Scopus). The selection and evaluation of studies followed the PRISMA guidelines. The risk of bias was evaluated using the PEDro Scale. Results: From the initial selection (n = 245), ten studies were retrieved, including from 10 to 67 patients. All but two (IPF) included patients with COPD. Based on the FEV1, patients with COPD were mainly categorised as moderate and severe. The teleactivities were heterogenous in terms of proposed exercises and way of settings and often not in agreement with the guidelines about pulmonary rehabilitation. Despite this, the effects of the interventions were globally positive on functional exercise capacity, quality of life, anxiety and depression, and impact of COPD on personal life but not on dyspnoea. The PEDro scores varied from 4 to 8. The adherence was higher than 80% when supervision during the exercise was included. Conclusion: This review demonstrated that the telerehabilitation is safe and well accepted by the patients, and could be considered as one option of classical pulmonary rehabilitation to improve the functional exercise capacity, quality of life, anxiety and depression, and the impact of COPD on personal’s life. This conclusion cannot be extrapolated to the other chronic lung diseases due to the lack of data. © 2022 by the authors.	https://www.scopus.com/inward/record.uri?eid=2-s	Q2	Scopus	EXC_Scope
Physical activity promotion interventions in chronic airways disea	2023	Physical inactivity is common in people with chronic airways disease (pwCAD) and associated with worse clinical outcomes and impaired quality of life. We conducted a systematic review and meta-analysis to characterise and evaluate the effectiveness of interventions promoting step-based physical activity (PA) in pwCAD. We searched for studies that included a form of PA promotion and step-count outcome measure. A random-effects model was used to determine the overall effect size using post-intervention values. 38 studies (n=32 COPD; n=5 asthma; n=1 bronchiectasis; study population: n=3777) were included. Overall, implementing a form of PA promotion resulted in a significant increase in step-count: median (IQR) 705 (183–1210) when compared with usual standard care: −64 (−597–229), standardised mean difference (SMD) 0.24 (95% CI: 0.12–0.36), p<0.01. To explore the impact of specific interventions, studies were stratified into subgroups: PA promotion+wearable activity monitor-based interventions (n=17) (SMD 0.37, p<0.01); PA promotion+step-count as an outcome measure (n=9) (SMD 0.18, p=0.09); technology-based interventions (n=12) (SMD 0.16, p=0.01). Interventions promoting PA, particularly those that incorporate wearable activity monitors, result in a significant and clinically meaningful improvement in daily step-count in pwCAD. © The authors 2023.	https://www.scopus.com/inward/record.uri?eid=2-s	Q2	Scopus	EXC_Scope

A meta-analysis on the structure of pulmonary rehabilitation mair	2022	Pulmonary rehabilitation (PR) improves functional capacity, health-related quality of life (HRQoL) in COPD patients, and maintenance programmes are relevant in preserving those improvements. However, little is known about the structure of maintenance programmes after PR. We performed a systematic review and meta-analysis of experimental and quasi-experimental studies evaluating individuals with COPD admitted to a maintenance PR programme, delivered after an initial PR programme. We reported functional capacity evaluation (6-minute-walking-test), HRQoL, dyspnoea and symptom control. Searches were performed on the 11th April 2021 using MEDLINE, Embase, EBSCO, CINAHL, Web of Science and Cochrane Library. We extracted summary-level data from trial publications and used a random-effects model, predicting that severe heterogeneity was detected. The protocol was registered in PROSPERO (CRD42021247724). Fifteen studies were included in the meta-analysis, with 1151 participants. Maintenance programmes were associated with a pooled mean increase of 27.08 meters in 6mWT (CI: 10.39 to 43.77; I2 = 93%; p < 0.0001), being better in supervised, long (>12 month) home-based programmes; and having a potential MD of -4.20 pts in SGRQ (CI: -4.49 to -3.91; I2 = 0%; p = 0.74). Regarding dyspnoea and exacerbations, we found a nonsignificant trend for improvement after maintenance PR programmes. Severe COPD patients showed smaller improvements in programmes up to a year. Overall, the strength of the underlying evidence was moderate. Despite limitations of risk of bias and heterogeneity, our results support that home-based, supervised, long-term maintenance PR programmes may significantly improve functional capacity in COPD patients and HRQoL. © 2022, The Author(s).	https://www.scopus.com/inward/record.uri?eid=2-s	Q2	Scopus	EXC_Scope
Evaluating the implementation of a remote-monitoring program fc	2020	Background: Implementing digital health technologies is complex but can be facilitated by considering the features of the tool that is being implemented, the team that will use it, and the routines that will be affected. Objective: The goal of this study was to assess the implementation of a remote-monitoring initiative for patients with chronic obstructive pulmonary disease in Ontario, Canada using the Tool+Team+Routine framework and to refine this approach to conceptualize the adoption of technologies in health care. Methods: This study was a qualitative research project that took place alongside a randomized controlled trial comparing a technology-enabled self-monitoring program with a technology-enabled self- and remote-monitoring program in patients with chronic obstructive pulmonary disease and with standard care. This study included interviews with 5 remote-monitoring patients, 3 self-monitoring patients, 2 caregivers, 5 health care providers, and 3 hospital administrators. The interview questions were structured around the 3 main concepts of the Tool+Team+Routine framework. Results: Findings emphasized that (1) technologies can alter relationships between providers and patients, and that these relationships drove the development of a new service arising from the technology, in our case, and (2) technologies can create additional work that is not visible to management as a result of not being considered within the scope of the service. Conclusions: Literature on the implementation of digital health technologies has still not reconciled the importance of interpersonal relationships to conventional implementation strategies. By acknowledging the centrality of such relationships, implementation teams can better plan for the adaptations required in order to make new technologies work for patients and health care providers. Further work will need to address how specific individuals administering a remote-monitoring program work to build relationships, and how these relationships and other sources of activity might lead to technological scope creep-an unanticipated expanding scope of work activities in relation to the function of the tool. © 2020 Journal of Medical Internet Research. All rights reserved.	https://www.scopus.com/inward/record.uri?eid=2-s	Q2	Scopus	EXC_Scope
Pulmonary rehabilitation in a post-covid-19 world: Telerehabilitati	2021	Pulmonary rehabilitation (PR) is effective in reducing symptoms and improving health status, and exercise tolerance of patients with chronic obstructive pulmonary disease (COPD). The coronavirus disease 19 (COVID-19) pandemic has greatly impacted PR programs and their delivery to patients. Owing to fears of viral transmission and resultant outbreaks of COVID-19, institution-based PR programs have been forced to significantly reduce enrolment or in some cases completely shut down during the pandemic. As a majority of COPD patients are elderly and have multiple co-morbidities including cardiovascular disease and diabetes, they are notably susceptible to severe complications of COVID-19. As such, patients have been advised to stay at home and avoid social contact to the maximum extent possible. This has increased patients' vulnerability to physical deconditioning, depres-sion, and social isolation. To address this major gap in care, some traditional hospital or clinic-centered PR programs have converted some or all of their learning contents to home-based telerehabilitation during the pandemic. There are, however, some significant barriers to this approach that have impeded its implementation in the community. These include variable access and use of technology (by patients), a lack of standardization of methods and tools for evaluation of the program, and inadequate training and resources for health professionals in optimally delivering telerehabilitation to patients. There is a pressing need for high-quality studies on these modalities of PR to enable the successful implementation of PR at home and via teleconferencing technologies. Here, we highlight the importance of telerehabilitation of patients with COPD in the post-COVID world and discuss various strategies for clinical implementation. © 2021 Tsutsui et al.	https://www.scopus.com/inward/record.uri?eid=2-s	Q2	Scopus	EXC_Scope
A Detailed Description of Physical Activity Counseling Interventio	2022	Physical activity interventions are recommended for people with chronic obstructive pulmonary disease (COPD). However, adherence is low. This scoping review aimed to provide detailed descriptions of physical activity counseling interventions for people with COPD that have been studied in randomized controlled trials. Common components included the use of pedometers/activity monitors, goal setting, and recording physical activity in a written or electronic diary. Clinicians could consider including these components within their management of people with COPD to promote increased physical activity levels. Additional research is needed to determine the key components that drive physical activity behavior change in this population. Copyright © 2022 Wolters Kluwer Health, Inc.	https://www.scopus.com/inward/record.uri?eid=2-s	Q2	Scopus	EXC_PubType
Chronic obstructive pulmonary disease: an urgent problem of hea	2021	Chronic obstructive pulmonary disease is one of the most urgent problems of the modern medical community. Despite the introduction of constantly updated schemes of early diagnosis and treatment into practical medicine, there is still an upward trend in the number of cases, under-examined persons, and prognostically unfavorable outcomes. The review highlights the findings of epidemiological studies confirming the widespread prevalence of the disease, accompanied by rapid disability progression, high mortality, and significant economic damage. The review lists the main exogenous and endogenous risk factors for the development of chronic obstructive pulmonary disease, including occupational etiology. Attention is drawn to the possibility of disease prevention in a professional environment with the proper motivation of the patient and the application of economic efforts. The article discusses the main causes of underdiagnosis and late diagnosis of the disease. Lifestyle modification makes an undeniable contribution to the prevention of chronic obstructive pulmonary disease and improving prognosis in the developed disease. Certain psychological characteristics that reduce adherence to treatment of such patients should be considered in organizing the management of this category of persons and creating special schools. It is important to create a classification of "endotypes" of chronic obstructive pulmonary disease, as well as sufficient public awareness about this disease with the aim of the earliest possible diagnosis. © Kazan Medical Journal. All rights reserved.	https://www.scopus.com/inward/record.uri?eid=2-s	Q2	Scopus	EXC_Scope
Effectiveness of Self-Monitoring Approach Using Fitness Tracker	2021	Background: A self-monitoring approach utilizing fitness trackers that provide feedback regarding physical activities has been recently applied to rehabilitation patients to promote voluntary walking activities. Although this approach has been proven to increase physical activity, it is uncertain whether the intervention improves walking ability. Aim: This review investigated whether the additional self-monitoring approach using activity trackers would improve walking ability in any type of rehabilitation setting. Methods: A systematic search was performed in four databases [PubMed (MEDLINE), The Cochrane Library, SPORTDiscus, and Cumulative Index to Nursing and Allied Health Literature] to identify studies that examined the self-monitoring approach combined with rehabilitative intervention vs. the same rehabilitative intervention only in participants with any unhealthy conditions. Two review authors independently assessed the eligibility of all the retrieved English literature published from 2009 to 2019, then discussed the final inclusion. The risk of bias was assessed referring to the criteria of the Cochrane Risk of Bias tool. The key findings were synthesized using narrative synthesis. In addition, a quantitative synthesis was conducted when more than two studies investigating the same disease were identified. Results: Eleven randomized controlled trials satisfied the eligibility criteria, nine of which had a lower risk of bias. The types of diseases included stroke, chronic obstructive pulmonary disease (COPD), cancer, Parkinson's disease, hemophilia, peripheral artery disease, post-total knee arthroplasty, and geriatric rehabilitation. Eight studies reported measures of walking endurance and four reported measures of gait speed. In the quantitative synthesis of two studies investigating COPD, there was a significant between-group difference in terms of changes in the 6-min walking distance from the baseline, which was favorable to the additional self-monitoring intervention group (mean difference: 13.1 m; 95% confidence interval, 1.8–24.5; 2 studies, 124 participants; p = 0.02; I2 = 0%). Other available data revealed no consistent evidence regarding effectiveness of the intervention. Conclusions: The findings indicate that there is little evidence suggesting the effectiveness of the self-monitoring approach in improving walking ability in rehabilitation settings. However, a weak recommendation for patients with stable COPD was implicated in the quantitative synthesis. Further research would be required to explore the best indications for this self-monitoring approach. Systematic Review Registration: CRD 42020157695. Copyright © 2021 Otaka, Oguchi, Kondo and Otaka.	https://www.scopus.com/inward/record.uri?eid=2-s	Q2	Scopus	EXC_Scope

Improving Physiological, Physical, and Psychological Health Out	2022	The Veterans Health Administration (VHA) is the largest integrated healthcare system in the United States (US) providing healthcare to an increasing number of middle-aged and older adults who remain at greater risk for chronic obstructive pulmonary disease (COPD) compared to their civilian counterparts. The VHA has obligated research funds, drafted clinical guidelines, and built programmatic infrastructure to support the diagnosis, treatment, and care management of Veterans with COPD. Despite these efforts, COPD remains a leading cause of morbidity and mortality in Veterans. This paper provides a narrative review of research conducted with US Veteran samples targeting improvement in COPD outcomes. We review key physiological, physical, and psychological health outcomes and intervention research that included US Veteran samples. We conclude with a discussion of directions for future research to continue advancing the treatment of COPD in Veterans and inform advancements in COPD research within and outside the VHA. © 2022 Bamonti et al.	https://www.scopus.com/inward/record.uri?eid=2-s	Q2	Scopus	EXC_PubType
Titration and follow-up for home noninvasive positive pressure ve	2021	Introduction: Home noninvasive positive pressure ventilation (NIPPV) has become a well-established treatment for stable hypercapnic chronic obstructive pulmonary disease (COPD) patients. There are still other challenges including appropriate titration of ventilator parameters, adequacy of follow-up, monitoring, and management at home to ensure effectiveness and security, and to improve quality of life. The Internet of Things (IoT) is the name given to the network of devices and other "things" with built-in sensors, software, electronics, and network connectivity, which can communicate these objects over wireless networks and then send data to a cloud platform. Reliable tele-monitoring and transmission of clinical parameters from home to hospitals have prompted the development of IoT-based home NIPPV. Objectives: This review provides an overview of titration and follow-up of home NIPPV and focuses on different technologies, modalities, managements, and cost-effectiveness used in IoT-based tele-monitoring of home mechanical ventilation. Data Source: Literature search of Web of Science, PubMed, and EMBASE was made to find relevant articles about tele-monitoring and the IoT in home mechanical ventilation over the last 15 years. We used the following search terms: NIPPV, COPD, home mechanical ventilation, telemedicine, tele-monitoring, and management. Conclusion: IoT-based management of home NIPPV, such as home titration and follow-up with the use of tele-monitoring, are emerging and yielding positive findings. However, clear conclusions based on RCT of tele-monitoring in COPD patients with NIPPV at home are only a few and large-scale multicenter studies are required for replication and further validation. © 2021 John Wiley & Sons Ltd.	https://www.scopus.com/inward/record.uri?eid=2-s	Q2	Scopus	EXC_Scope
Can self-management programmes change healthcare utilisation	2021	Objective: The study aims to evaluate the ability of self-management programmes to change the healthcare-seeking behaviours of people with Chronic Obstructive Pulmonary Disease (COPD), and any associations between programme design and outcomes. Methods: A systematic search of the literature returned randomised controlled trials of SMPs for COPD. Change in healthcare utilisation was the primary outcome measure. Programme design was analysed using the Theoretical Domains Framework (TDF). Results: A total of 26 papers described 19 SMPs. The most common utilisation outcome was hospitalisation (n = 22). Of these, 5 showed a significant decrease. Two theoretical domains were evidenced in all programmes: skills and behavioural regulation. All programmes evidenced at least 5 domains. However, there was no clear association between TDF domains and utilisation. Overall, study quality was moderate to poor. Conclusion: This review highlights the need for more alignment in the goals, design, and evaluation of SMPs. Specifically, the TDF could be used to guide programme design and evaluation in future. Practice implications: Practices have a reasonable expectation that interventions they adopt will provide patient benefit and value for money. Better design and reporting of SMP trials would address their ability to do so. © 2020 The Authors	https://www.scopus.com/inward/record.uri?eid=2-s	Q2	Scopus	EXC_Scope
Models of care across the continuum of exacerbations for patient	2020	Exacerbations of chronic obstructive pulmonary disease (COPD) are associated with significant morbidity and mortality, and treatments require a multidisciplinary approach to address patient needs. This review considers different models of care across the continuum of exacerbations (1) chronic care and self-management interventions with the action plan, (2) domiciliary care for severe exacerbation and the impact on readmission prevention and (3) the discharge care bundle for management beyond the acute exacerbation episode. Self-management strategies include written action plans and coaching with patient and family support. Self-management interventions facilitate the delivery of good care, can reduce exacerbations associated with admission, be cost-effective and improve quality of life. Hospitalization as a complication of exacerbation is not always unavoidable. Domiciliary care has been proposed as a solution to replace part, and perhaps even all, of the patient's in-hospital stay, and to reduce hospital bed days, readmission rates and costs; low-risk patients can be identified using risk stratification tools. A COPD discharge bundle is another potentially important approach that can be considered to improve the management of COPD exacerbations complicated by hospital admission; it comprised treatments that have demonstrated efficacy, such as smoking cessation, personalized pharmacotherapy and non-pharmacotherapy such as pulmonary rehabilitation. COPD bundles may also improve the transition of care from the hospital to the community following exacerbation and may reduce readmission rates. Future models of care should be personalized – providing patient education aiming at behaviour changes, identifying and treating co-morbidities, and including outcomes that measure quality of care rather than focusing only on readmission quantity within 30 days. © The Author(s) 2020.	https://www.scopus.com/inward/record.uri?eid=2-s	Q2	Scopus	EXC_PubType
ERS international congress, madrid, 2019: Highlights from the ai	2020	The European Respiratory Society (ERS) International Congress 2019 in Madrid, Spain, was a platform for scientific discussion of the highest quality scientific research, cutting-edge techniques and innovative new therapies within the respiratory field. This article discusses some of the high-quality research studies presented at that Congress, with a focus on airway diseases, including asthma, COPD, small airways, bronchiectasis and cough, presented through the Airway Diseases, Asthma and COPD Assembly (Assembly 5) of the ERS. The authors establish the key take-home messages of these studies, compare their findings and place them into context of current understanding. © ERS 2020.	https://www.scopus.com/inward/record.uri?eid=2-s	Q2	Scopus	EXC_PubType
Hospitalization risk factors of older cohorts of home health care p	2019	Nearly one million Medicare home health care beneficiaries are hospitalized annually of which one-quarter are considered preventable. Older hospitalized patients are at risk for nosocomial complications and poorer outcomes and incur higher health care costs. This paper reports the results of a systematic review of 28 studies on hospitalization risk factors of older home health care patients. It found that males, Blacks, and non-Asian minorities are at greater hospitalization risk. Factors associated with higher risk included skin ulcers, psychiatric conditions, dyspnea/COPD, cardiovascular conditions, diabetes, functional deficits, more comorbidities, and higher medication usage. These findings can inform practice, research, and policy. © 2019, © 2019 Taylor & Francis.	https://www.scopus.com/inward/record.uri?eid=2-s	Q2	Scopus	EXC_Scope
Acutely decompensated versus acute heart failure: two different	2020	Heart failure (HF) has been classified in chronic HF (CHF) and acute HF (AHF). The latter has been subdivided in acutely decompensated chronic HF (ADCHF) defined as the deterioration of preexisting CHF and de novo AHF defined as the rapid development of new symptoms and signs of HF that requires urgent medical attention. However, ADCHF and de novo AHF have fundamental pathophysiological differences. Most importantly, the typical illness trajectory of HF, which is similar to that of other chronic organ diseases including lung, renal, and liver failure, features a gradual decline, with acute episodes usually related to disease evolution followed by partial recovery. Thus, ADCHF should be considered part of the natural history of CHF and renamed CHF exacerbation (CHFE) in accordance with the appropriate terminology used in chronic obstructive pulmonary disease. AHF, in turn, should include only acute de novo HF. The clinical implications of this paradigm shift will be in CHFE the change in focus from in-hospital to optimal ambulatory CHF management aiming at primary and secondary CHFE prevention, while in AHF, the institution of measures for in-hospital limitation of cardiac injury and prevention or retardation of symptomatic CHF development. © 2019, Springer Science+Business Media, LLC, part of Springer Nature.	https://www.scopus.com/inward/record.uri?eid=2-s	Q2	Scopus	EXC_Scope
The effects of nurse-driven self-management programs on chron	2020	Aims: To analyse the effects of nurse-driven self-management (SM) programs on physical and psychosocial health variables in people with chronic obstructive pulmonary disease (COPD). Design: A systematic review and meta-analysis. Data Sources: An exhaustive scanning of PubMed, Cochrane Controlled Register of Trials, CINAHL, ScienceDirect and Medline databases between January 2010–December 2019 was conducted for this meta-analysis. Review Methods: Randomized controlled trials (RCTs) related to nurse-driven SM programs in COPD population were included. The standardized mean differences with 95% confidence intervals were determined for the main variables and heterogeneity was analysed using the I2 test. The Preferred Reporting Items for Systematic Reviews and Meta-Analysis (PRISMA) was used. Results: Twelve studies were included. The results indicated that significant difference in physical health scores based on COPD Assessment Tool (CAT) and walking distance according to the 6-min walk distance (6MWD) test in the intervention groups compared with the control groups. About psychosocial health findings, the quality of life increased and the Hospital Anxiety and Depression Scale (HADS) scores decreased following SM programs. All of the studies had good quality (varying from 5–8 points) according to The Modified Jadad Scale. Conclusion: Nurse-driven SM programs may contribute to prognosis in patients with COPD. Due to methodological weaknesses in the included trials, high-quality RCTs are needed to better determine the effects of nurse-driven SM programs in the management of COPD. Nurse-driven SM programs may be employed as a useful strategy to improve health status and QOL and psychosocial health in the COPD population, as well. Impact: Current evidence shows that nurse-driven SM programs could be safely integrated into the clinical practice for patients with COPD. Future studies are warranted that evaluating the effects of nurse-driven SM programs on other frequently observed COPD symptoms such as dyspnoea, fatigue and sleep disturbance. © 2020 John Wiley & Sons Ltd	https://www.scopus.com/inward/record.uri?eid=2-s	Q2	Scopus	EXC_Scope

Objectively measured physical activity in patients with COPD: Re	2021	Physical activity (PA) is of key importance for health among healthy persons and individuals with chronic obstructive pulmonary disease (COPD). PA has multiple dimensions that can be assessed and quantified objectively using activity monitors. Moreover, as shown in the published literature, variable methodologies have been used to date to quantify PA among individuals with COPD, precluding clear comparisons of outcomes across studies. The present paper aims to provide a summary of the available literature for the rationale behind using objectively measured PA and proposes a standardized methodology for assessment, including standard operating procedures for future research. The present paper, therefore, describes the concept of PA, reports on the importance of PA, summarizes the dimensions of PA, provides a standard operating procedure on how to monitor PA using objective assessments, and describes the psychometric properties of objectively measured PA. The present international task force recommends implementation of the standard operating procedure for PA data collection and reporting in the future. This should further clarify the relationship between PA and clinical outcomes, test the impact of treatment interventions on PA in individuals with COPD, and successfully propose a PA endpoint for regulatory qualification in the future. © 2021 COPD Foundation. All rights reserved.	https://www.scopus.com/inward/record.uri?eid=2-s	Q2	Scopus	EXC_Scope
Self-efficacy of older people using technology to self-manage CC	2021	Background and Objectives: Although telehealth research among the general population is voluminous, the quality of studies is low and results are mixed. Little is known specifically concerning older people and their self-efficacy to engage with and benefit from such technologies. This article reviews the evidence for which self-care telehealth technology supports the self-efficacy of older people with long-term conditions (LTCs) living at home. Research Design and Methods: Following Preferred Reporting Items for Systematic Reviews and Meta-Analyses Statement (PRISMA) guidelines, this overview of systematic reviews focused on four LTCs and the concept of "self-efficacy." Quality was appraised using R-AMSTAR and study evaluation was guided by the PRISMS taxonomy for reporting of self-management support. Heterogeneous data evidencing technology-enhanced self-efficacy were narratively synthesized. Results: Five included articles contained 74 primary studies involving 9,004 participants with chronic obstructive pulmonary disease, hypertension, heart failure, or dementia. Evidence for self-care telehealth technology supporting the self-efficacy of older people with LTCs living at home was limited. Self-efficacy was rarely an outcome, also attrition and dropout rates and mediators of support or education. The pathway from telehealth to self-efficacy depended on telehealth modes and techniques promoting healthy lifestyles. Increased self-care and self-monitoring empowered self-efficacy, patient activation, or mastery. Discussion and Implications: Future research needs to focus on the process by which the intervention works and the effects of mediating variables and mechanisms through which self-management is achieved. Self-efficacy, patient activation, and motivation are critical components to telehealth's adoption by the patient and hence to the success of self-care in self-management of LTCs. Their invisibility as outcomes is a limitation. © 2020 The Author(s) 2020. Published by Oxford University Press on behalf of The Gerontological Society of America. All rights reserved. For permissions, please e-mail: journals.permissions@oup. com.	https://www.scopus.com/inward/record.uri?eid=2-s	Q2	Scopus	EXC_Scope
Effectiveness of tele-monitoring by patient severity and interventi	2019	Background: Chronic obstructive pulmonary disease is a major burden on healthcare systems worldwide. Tele-monitoring has recently been used for management of chronic obstructive pulmonary disease patients. Objectives: We analyzed the effect of tele-monitoring on chronic obstructive pulmonary disease patients and performed subgroup analysis by patient severity and intervention type. Design: Systematic review. Data source: Electronic databases including Ovid-Medline, Ovid-Embase, and the Cochrane Library. Review methods: We conducted a meta-analysis of randomized controlled trials published up to April 2017. Three databases were searched, two investigators independently extracted data and assessed study quality using risk of bias. Results: Out of 1,185 studies, 27articles were identified to be relevant for this study. The included studies were divided by intervention: 15studies used tele-monitoring only, 4studies used integrated tele-monitoring (pure control), and 8studies used integrated tele-monitoring (not pure control). We also divided the studies by patient severity: 16studies included severely ill patients, 8studies included moderately ill patients, and 3studies did not discuss the severity of the patients' illness. Meta-analysis showed that tele-monitoring reduced the emergency room visits (risk ratio 0.63, 95% confidence interval 0.55-0.72) and hospitalizations (risk ratio 0.88, 95% confidence interval 0.80–0.97). The subgroup analysis of patient severity showed that tele-monitoring more effectively reduced emergency room visits in patients with severe vs. moderate disease (risk ratio 0.48, 95% confidence interval 0.31–0.74; risk ratio 1.28, 95% confidence interval 0.61–2.69, retrospectively) and hospitalizations (risk ratio 0.92, 95% confidence interval 0.82–1.02; risk ratio 1.24, 95% confidence interval 0.57–2.70, retrospectively). The mental health quality of life score (mean difference 3.06, 95% confidence interval 2.15–3.98) showed more improved quality of life than the physical health quality of life score (mean difference -0.11, 95% confidence interval -0.83–0.61). Conclusions: Tele-monitoring reduced rates of emergency room visits and hospitalizations and improved the mental health quality of life score. Integrated tele-monitoring including the delivery of coping skills or education by online methods including pulmonary rehabilitation is recommended to produce significant improvement. This application of integrated tele-monitoring (the delivery of education, exercise etc. in addition to tele-monitoring) is more useful for patients with (very) severe chronic obstructive pulmonary disease than those with moderate disease. Tele-monitoring might be a useful application of information and communication technologies, if the intervention includes the appropriate intervention components for eligible patients. Further studies such as large size randomized controlled trials with sub-group by patient severity and intervention type is needed to confirm these finding. © 2018	https://www.scopus.com/inward/record.uri?eid=2-s	Q2	Scopus	EXC_Scope
Recent Advances in the Management of Acute Exacerbations of	2020	Chronic obstructive pulmonary disease is a chronic, irreversible obstructive lung disease that results from exposure to noxious stimuli. Acute exacerbations of chronic obstructive pulmonary disease (AECOPD) usually result from viral or bacterial respiratory infections, but may also result from exposure to environmental pollution. AECOPD are associated with functional decline, increased risk of subsequent exacerbations, and death. Despite the poor prognosis of AECOPD, patients are empowered through self-management programs in their battle against this lethal disease. Morbidity and mortality of chronic obstructive pulmonary disease hospitalizations are reduced by implementing standardized treatment modalities outlined in this article throughout the hospitalization and beyond. © 2020 Elsevier Inc.	https://www.scopus.com/inward/record.uri?eid=2-s	Q2	Scopus	EXC_Scope
Noninvasive positive pressure ventilation in stable patients with (	2020	Purpose of reviewLong-term noninvasive positive pressure ventilation (NIV) used to be a controversial form of therapy for patients with stable hypercapnic chronic obstructive pulmonary disease (SH-COPD). New evidence described in this review defines the optimal settings, timing and target population for NIV utilization in SH-COPD necessary to maximize its benefit.Recent findingsNIV, when titrated appropriately, leads to improved clinical outcomes. High inspiratory positive airway pressures aimed at decreasing CO2 levels can ensure NIV success in SH-COPD. NIV initiated when patients remain hypercapnic whereas in a clinical stable state following an acute exacerbation can prolong the time to a readmission. Technological advances in NIV algorithms and remote monitoring have the potential to improve use and titration. NIV and portable NIV improve exercise tolerance and may accentuate the benefits derived from pulmonary rehabilitation alone.SummaryUse of high-intensity NIV in SH-COPD is beneficial yet appropriate patient selection and implementation is paramount. © 2020 Lippincott Williams and Wilkins. All rights reserved.	https://www.scopus.com/inward/record.uri?eid=2-s	Q2	Scopus	EXC_Scope
Strategies to Increase Physical Activity in Chronic Respiratory Di	2019	Physical activity is important to maintain health. Patients who reduce their physical activity are at increased risk of developing comorbidities and faster decline in health. Interventions to enhance physical activity require a behavior change from patients and these interventions have become increasingly popular in chronic obstructive pulmonary disease. However, few interventions have shown long-term effects and all focused on enhancing physical activity rather than the maintenance thereof. In patients with very low exercise tolerance or with significant symptom burden, enhancing physical activity be difficult and interventions should first focus on enhancing exercise tolerance. © 2019 Elsevier Inc.	https://www.scopus.com/inward/record.uri?eid=2-s	Q2	Scopus	EXC_Scope
COPD Exacerbation Syndrome: The Spanish Perspective on an	2022	The definition of exacerbation of COPD as a syndrome, as proposed by the Spanish COPD guidelines (GesEPOC) 2021 update, and the consequences that this implies, have direct implications on patient care. This review analyzes this novel vision of the COPD exacerbation syndrome, its rationale, and its clinical implications, as opposed to the traditional symptoms-based or event-based definitions. An exacerbation conceived as a syndrome provides us with an umbrella term to include a set of diverse alterations, which, either in isolation or more frequently in combination, are clinically expressed in a similar way in patients with COPD. In patients with COPD, this occurs as a consequence of worsening expiratory airflow limitation or the underlying inflammatory process, producing a worsening in symptoms with respect to the baseline situation. This definition therefore assumes a worsening in at least one of the two key physiopathological markers, lung function and inflammation. The main features of this new physiopathological proposal include a syndromic approach with narrower differential diagnosis, the use of several biomarkers, treatable traits to better guide treatment, and a new severity classification. Further research is needed to examine the role of eosinophils in this context, but currently, the early results are promising. The evaluation of severity is key in the multidimensional characterization of exacerbation and the GesEPOC 2021 proposes new approaches and also recommends the use of multidisciplinary scores for severity categorization in patients. Finally, another innovation in the GesEPOC 2021 refers to the recurrence of exacerbations, which has implications for disease prognosis or long-term clinical impact which need to be elucidated in further studies. © 2022 Soler-Cataluña and Lopez-Campos.	https://www.scopus.com/inward/record.uri?eid=2-s	Q2	Scopus	EXC_Scope

The Role of Health Literacy on the Self-Management of Chronic	2020	The effects of health literacy in developing self-management skills among people suffering from Chronic Obstructive Pulmonary Disease (COPD) is a topic that has been lightly tread upon. The advent of tobacco smoking and air pollution caused by the industrialisation era has caused a startling increase in the rates of incidence and prevalence of those diagnosed with COPD. Despite advancement in medical treatment, prevention and health care systems COPD poses a great challenge to public health now than ever before. This systematic review examines eight articles that have dealt with the role health literacy plays in developing self-management skills. This study found that there is no relationship between the adequacy of health literacy and the knowledge or learning of a self-management skill. The relationship between heath literacy and developing skills such as correct technique of inhaler use, awareness of an exacerbation, usage of home-based technological support (telehomecare) needs further delving. Remarkably, it also revealed that health literacy sensitive materials improved self-management skills in all the levels of health literacy. More research is required in identifying literacy sensitive methods that would be beneficial to all disregarding of the level health literary. A wider range of self-management skills pertaining to prevention, maintenance and control needs to be explored. © 2020, © 2020 Taylor & Francis Group, LLC.	https://www.scopus.com/inward/record.uri?eid=2-s	Q2	Scopus	EXC_Scope
Perceptions of patients with chronic obstructive pulmonary disea	2021	Background: There are some qualitative studies on the views of patients with chronic obstructive pulmonary disease (COPD) on telemedicine, however, there are few related qualitative systematic reviews. Objectives: To systematically review and synthesize qualitative studies involving the perceptions of patients with COPD about telemedicine to understand patients' attitudes and expectations for telemedicine and determine the obstacles and stimulus in the use of telemedicine. Methods: We searched PubMed, Web of Science, MEDLINE, Embase and CINAHL for articles published from January 2000 to December 2020. The data were analysed using thematic synthesis. Results: We included 20 articles involving 19 studies and 301 patients, and we identified four themes: perceived ease of use, perceived usefulness, perceived difficulty of use, and perceived uselessness. We found that although patients have different views on telemedicine, most of them have a positive attitude towards it. Conclusions: The synthesis of views will help us determine the factors that promote or hinder the application of telemedicine and guide the design and implementation of telemedicine in the future. © 2021	https://www.scopus.com/inward/record.uri?eid=2-s	Q2	Scopus	EXC_Scope
A systematic map and in-depth review of European telehealth int	2019	Background: Evidence to support the implementation of telehealth (TH) interventions in the management of chronic obstructive pulmonary disease (COPD) varies throughout Europe. Despite more than ten years of TH research in COPD management, it is still not possible to define which TH interventions are beneficial to which patient group. Therefore, informing policymakers on TH implementation is complicated. We aimed to examine the provision and efficacy of TH for COPD management to guide future decision-making. Methods: A mapping study of twelve systematic reviews of TH interventions for COPD management was conducted. This was followed by an in-depth review of fourteen clinical trials performed in Europe extracted from the systematic reviews. Efficacy outcomes for COPD management were synthesized. Results: The mapping study revealed that systematic reviews with a meta-analysis often report positive clinical outcomes. Despite this, we identified a lack of pragmatic trial design affecting the synthesis of reported outcomes. The in-depth review visualized outcomes for three TH categories, which revealed a plethora of heterogeneous outcomes. Suggestions for reporting within these three outcomes are synthesized as targets for future empirical research reporting. Conclusion: The present study indicates the need for more standardized and updated systematic reviews. Policymakers should advocate for improved TH trial designs, focusing on the entire intervention's adoption process evaluation. One of the policymakers' priorities should be the harmonization of the outcome sets, which would be considered suitable for deciding about subsequent reimbursement. We propose possible outcome sets in three TH categories which could be used for discussion with stakeholders. © 2019 Elsevier Ltd	https://www.scopus.com/inward/record.uri?eid=2-s	Q2	Scopus	EXC_PubType
The role of telemedicine in extending and enhancing medical ma	2021	Medical management of a chronic obstructive pulmonary disease (COPD) patient must incorporate a broadened and holistic approach to achieve optimal outcomes. This is best achieved with integrated care, which is based on the chronic care model of disease management, proactively addressing the patient’s unique medical, social, psychological, and cognitive needs along the trajectory of the disease. While conceptually appealing, integrated care requires not only a different approach to disease management, but considerably more health care resources. One potential way to reduce this burden of care is telemedicine: technology that allows for the bidirectional transfer of important clinical information between the patient and health care providers across distances. This not only makes medical services more accessible; it may also enhance the efficiency of delivery and quality of care. Telemedicine includes distinct, often overlapping interventions, including telecommunication (enhancing lines of communication), telemonitoring (symptom reporting or the transfer of physiological data to health care providers), physical activity monitoring and feedback to the patient and provider, remote decision support systems (identifying “red flags,” such as the onset of an exacerbation), tele-consultation (directing assessment and care from a distance), tele-education (through web-based educational or self-management platforms), tele-coaching, and tele-rehabilitation (providing educational material, exercise training, or even total pulmonary rehabilitation at a distance when standard, center-based rehabilitation is not feasible). While the above components of telemedicine are conceptually appealing, many have had inconsistent results in scientific trials. Interventions with more consistently favorable results include those potentially modifying physical activity, non-invasive ventilator management, and tele-rehabilitation. More inconsistent results in other telemedicine interventions do not necessarily mean they are ineffective; rather, more data on refining the techniques may be necessary. Until more outcome data are available clinicians should resist being caught up in novel technologies simply because they are new. © 2021 by the authors. Licensee MDPI, Basel, Switzerland.	https://www.scopus.com/inward/record.uri?eid=2-s	Q2	Scopus	EXC_PubType
Addressing Reduced Laboratory-Based Pulmonary Function Tes	2020	To reduce the spread of the severe acute respiratory syndrome coronavirus 2, many pulmonary function testing (PFT) laboratories have been closed or have significantly reduced their testing capacity. Because these mitigation strategies may be necessary for the next 6 to 18 months to prevent recurrent peaks in disease prevalence, fewer objective measurements of lung function will alter the diagnosis and care of patients with chronic respiratory diseases. PFT, which includes spirometry, lung volume, and diffusion capacity measurement, is essential to the diagnosis and management of patients with asthma, COPD, and other chronic lung conditions. Both traditional and innovative alternatives to conventional testing must now be explored. These may include peak expiratory flow devices, electronic portable spirometers, portable exhaled nitric oxide measurement, airwave oscillometry devices, and novel digital health tools such as smartphone microphone spirometers and mobile health technologies along with integration of machine learning approaches. The adoption of some novel approaches may not merely replace but could improve existing management strategies and alter common diagnostic paradigms. With these options comes important technical, privacy, ethical, financial, and medicolegal barriers that must be addressed. However, the coronavirus disease 19 pandemic also presents a unique opportunity to augment conventional testing by including innovative and emerging approaches to measuring lung function remotely in patients with respiratory disease. The benefits of such an approach have the potential to enhance respiratory care and empower patient self-management well beyond the current global pandemic. © 2020 American College of Chest Physicians	https://www.scopus.com/inward/record.uri?eid=2-s	Q2	Scopus	EXC_Scope
The Perspectives of Patients with Chronic Diseases and Their Ca	2021	Background: Self-management (SM) interventions are supportive interventions systematically provided by healthcare professionals, peers, or laypersons to increase the skills and confidence of patients in their ability to manage chronic diseases. We had two objectives: (1) to summarise the preferences and experiences of patients and their caregivers (informal caregivers and healthcare professionals) with SM in four chronic diseases and (2) to identify and describe the relevant outcomes for SM interventions from these perspectives. Methods: We conducted a mixed-methods scoping review of reviews. We searched three databases until December 2020 for quantitative, qualitative, or mixed-methods reviews exploring patients’ and caregivers’ preferences or experiences with SM in type 2 diabetes mellitus (T2DM), obesity, chronic obstructive pulmonary disease (COPD), and heart failure (HF). Quantitative data were narratively synthesised, and qualitative data followed a three-step descriptive thematic synthesis. Identified themes were categorised into outcomes or modifiable factors of SM interventions. Results: We included 148 reviews covering T2DM (n = 53 [35.8%]), obesity (n = 20 [13.5%]), COPD (n = 32 [21.6%]), HF (n = 38 [25.7%]), and those with more than one disease (n = 5 [3.4%]). We identified 12 main themes. Eight described the process of SM (disease progression, SM behaviours, social support, interaction with healthcare professionals, access to healthcare, costs for patients, culturally defined roles and perceptions, and health knowledge), and four described their experiences with SM interventions (the perceived benefit of the intervention, individualised care, sense of community with peers, and usability of equipment). Most themes and subthemes were categorised as outcomes of SM interventions. Conclusion: The process of SM shaped the perspectives of patients and their caregivers on SM interventions. Their perspectives were influenced by the perceived benefit of the intervention, the sense of community with peers, the intervention’s usability, and the level of individualised care. Our findings can inform the selection of patient-important outcomes, decision-making processes, including the formulation of recommendations, and the design and implementation of SM interventions. © 2021, The Author(s).	https://www.scopus.com/inward/record.uri?eid=2-s	Q2	Scopus	EXC_PubType

Contemporary perspectives in COPD: Patient burden, the role of	2021	An individual's experience of COPD is determined by many factors in addition to the pathological features of chronic bronchitis and emphysema and the symptoms that derive directly from them. Multimorbidity is the norm rather than the exception, so most people with COPD are living with a range of other medical problems which can decrease overall quality of life. COPD is caused by the inhalation of noxious particles or gases, in particular tobacco smoke, but also by early life disadvantage impairing lung development and by occupations where inhaled exposures are common (e.g. industrial, farming and cleaning work). Wealthy people are therefore relatively protected from developing COPD and people who do develop the condition may have reduced resources to cope. COPD is also no longer a condition that predominantly affects men. The prevalence of COPD among women has equalled that of men since 2008 in many high-income countries, due to increased exposure to tobacco, and in low-income countries due to biomass fuels. COPD is one of the leading causes of death in women in the USA, and death rates attributed to COPD in women in some countries are predicted to overtake those of men in the next decade. Many factors contribute to this phenomenon, but in addition to socioeconomic and occupational factors, there is increasing evidence of a higher susceptibility of females to smoking and pollutants. Quality of life is also more significantly impaired in women. Although most medications (bronchodilators and inhaled corticosteroids) used to treat COPD demonstrate similar trends for exacerbation prevention and lung function improvement in men and women, this is an understudied area and clinical trials frequently have a preponderance of males. A better understanding of gender-based predictors of efficacy of all therapeutic interventions is crucial for comprehensive patient care. There is an urgent need to recognize the increasing burden of COPD in women and to facilitate global improvements in disease prevention and management in this specific population. Many individuals with COPD follow a trajectory of both lung function decline and also multimorbidity. Unfavourable lung function trajectories throughout life have implications for later development of other chronic diseases. An enhanced understanding of the temporal associations underlying the development of coexisting diseases is a crucial first step in unravelling potential common disease pathways. Lessons can be learned from exploring disease trajectories of other NCD as well as multimorbidity development. Further research will be essential to explain how early life risk factors commonly influence trajectories of COPD and other diseases, how different diseases develop in relation to each other in a temporal way and how this ultimately leads to different multimorbidity patterns in COPD. This review integrates new knowledge and ideas pertaining to three broad themes (i) the overall burden of disease in COPD, (ii) an unappreciated high burden in women and (iii) the contrast of COPD trajectories and different multimorbidity patterns with trajectories of other NCD. The underlying pathology of COPD is largely irreversible, but many factors noted in the review are potentially amenable to intervention. Health and social care systems need to ensure that effective treatment is accessible to all people with the condition. Preventive strategies and treatments that alter the course of disease are crucial, particularly for patients with COPD as one of many problems. © 2021 Asian Pacific Society of Respirology.	https://www.scopus.com/inward/record.uri?eid=2-s	Q2	Scopus	EXC_Scope
Remote patient monitoring technologies for predicting chronic ob	2020	Background: Chronic obstructive pulmonary disease (COPD) is the third leading cause of death by disease worldwide and has a 30-day readmission rate of 22.6%. In 2015, COPD was added to the Medicare Hospital Readmission Reductions Program. Objective: The objective of this paper was to survey the current medical technologies for remote patient monitoring (RPM) tools that forecast COPD exacerbations in order to reduce COPD readmissions. Methods: We searched literature and digital health news to find commercially available RPM devices focused on predicting COPD exacerbations. These technologies were reviewed and compared according to four criteria: Forecasting ability, cost, ease of use, and appearance. A rating system was developed to facilitate the evaluation process. Results: As of June 2019, a list of handheld and hands-free devices was compiled. We compared features and found substantial variations. Devices that ranked higher on all criteria tended to have a high or unlisted price. Commonly mass-marketed devices like the pulse oximeter and spirometer surprisingly fulfilled the least criteria. Conclusions: The COPD RPM technologies with most technological promise and compatibility with daily living appear to have high or unlisted prices. Consumers and providers need better access to product information to make informed decisions. © 2020 JMIR Publications. All rights reserved.	https://www.scopus.com/inward/record.uri?eid=2-s	Q2	Scopus	EXC_PubType
The Utility of Electronic Inhaler Monitoring in COPD Managemen	2020	COPD is a common respiratory disorder that poses a major health-care burden with societal and financial ramifications. Although effective inhaled therapies are available, nonadherence is common among patients with COPD and potentially contributes to the burden of this disease. Electronic inhaler monitoring (EIM) is a novel modality that enables real-time assessment of adherence to inhaled therapy and informs the assessment of treatment effectiveness. EIM can be combined with physician feedback, automated audiovisual reminders, and text messaging to bolster adherence. Clinical studies have suggested that EIM can diagnose nonadherence, improve adherence, and predict exacerbations. Using an EIM-guided protocol has the potential to avoid treatment escalation in the nonadherent. Coupling EIM to behavioral intervention is an area of ongoing research with mixed results, with some studies showing benefit and others showing minimal or no significant change in clinical outcomes. Further investigation is necessary to understand the incremental benefits of EIM features, delineate optimal program implementation, and target patient populations that would benefit the most from monitoring. © 2020 American College of Chest Physicians	https://www.scopus.com/inward/record.uri?eid=2-s	Q2	Scopus	EXC_Scope
Efficacy of web-based supportive interventions in quality of life in	2021	Background: Adults living with Chronic Obstructive Pulmonary Disease (COPD) often have difficulties when trying to access health care services. Interactive communication technologies are a valuable tool to enable patients to access supportive interventions to cope with their disease. The aim of this revision and meta-analysis is to analyze the content and efficacy of web-based supportive interventions in quality of life in COPD. Methods: Medline (via PubMed), Web of Science, and Scopus were the databases used to select the studies for this systematic review. A screening, analysis, and assessment of the methodological quality was carried out by two independent researchers. A meta-analysis of the extracted data was performed. Results: A total of 9 of the 3089 studies reviewed met the inclusion criteria. Most repeated web content elements were educational and involved communication with healthcare professional content. Finally, seven of the nine studies were included in a quantitative analysis. Web-based supportive interventions significantly improved quality of life when added to usual care (SMD = −1.26, 95% CI = −1.65, −0.86; p < 0.001) but no significant differences were found when compared with an autonomous pedometer walking intervention (p = 0.64) or a face-to-face treatment (p = 0.82). Conclusion: This systematic review and meta-analysis suggests that web-based supportive interventions may complement or accompany treatments in COPD patients due to the advantages of online interventions. The results obtained should be treated with caution due to the limited number of studies in this area and methodological weaknesses. © 2021 by the authors. Licensee MDPI, Basel, Switzerland.	https://www.scopus.com/inward/record.uri?eid=2-s	Q2	Scopus	EXC_Scope
Key toolkits of non-pharmacological management in COPD: Duri	2021	Individuals with COPD are at higher risk of severe disease and mortality if they contract COVID-19. Shielding and social distancing have negatively impacted the delivery of routine care for COPD patients, which should be maintained to avoid further deterioration. We aimed to review the literature about the key toolkits of non-pharmacological treatments of COPD patients before and during the COVID-19 pandemic. In particular, we focused on smoking cessation, pulmonary rehabilitation, and telehealth delivery approaches during the COVID-19 crisis. Smoking cessation services are important to mitigate the spread of the virus, especially in people with chronic lung disease; the pandemic, in one way or another, has helped to enhance people's motivation to quit smoking. Also, tele-rehabilitation is considered as effective as conventional pulmonary rehabilitation in controlling symptoms of disease, promoting physical activity, and enhancing self-management of COPD. Telerehabilitation offers flexibility and it could be the dominant mode for providing a pulmonary rehabilitation programme. Finally, the use of telehealth (TH) modes has trended during the pandemic. Consensus about the effectiveness of TH in reducing exacerbation events is still inconclusive. In the context of COPD, further clinical research must concentrate on understanding attitudes, behaviours, and motivations towards smoking cessation. Further recommendations include gauging the feasibility of a long-term tele-rehabilitation programme in large COPD populations, designing more COPD-related mobile apps, and evaluating the feasibility of tele-rehabilitation in clinical practice. © 2021 The Author(s). Published by BRI.	https://www.scopus.com/inward/record.uri?eid=2-s	Q2	Scopus	EXC_Scope
Use of pedometers as a tool to promote daily physical activity lev	2019	The aim of this study was to examine the use of pedometers as a tool to promote daily physical activity levels in patients with COPD. A systematic review meta-analysis of pedometer physical activity promotion in patients with COPD was conducted. Medline/PubMed, Cochrane Library, Web of Science and CINAHL were searched from inception to January 2019. The search strategy included the following keywords: physical activity promotion, pulmonary rehabilitation and daily physical activity. The eligibility criteria for selecting studies were randomised controlled trials reporting pedometer physical activity promotion in patients with COPD. Improvements in steps per day were found with pedometer physical activity promotion either standalone (n=12, mean 0.53 (95% CI 0.29-0.77); p=0.00001) or alongside pulmonary rehabilitation (n=7, 0.51 (0.13-0.88); p=0.006). A subgroup analysis reported significant differences in the promotion of physical activity based on baseline physical activity levels and the type of instrument used to assess levels of physical activity. Future trials should consider the way in which pedometers are used to promote physical activity to inform clinical practice in the setting of pulmonary rehabilitation. © 2019, European Respiratory Society. All rights reserved.	https://www.scopus.com/inward/record.uri?eid=2-s	Q2	Scopus	EXC_Scope

Active video games for rehabilitation in respiratory conditions: Sy	2019	Background: Exercise and physical activity are key components of treatment for chronic respiratory diseases. However, the level of physical activity and adherence to exercise programs are low in people with these diseases. Active video games (AVGs) may provide a more engaging alternative to traditional forms of exercise. Objective: This review examines the effectiveness of game-based interventions on physiological outcome measures, as well as adherence and enjoyment in subjects with chronic respiratory diseases. Methods: A systematic search of the literature was conducted, with full texts and abstracts included where they involved an AVG intervention for participants diagnosed with respiratory conditions. A narrative synthesis of included studies was performed. Additionally, meta-analysis comparing AVGs with traditional exercise was undertaken for 4 outcome measures: Mean heart rate (HR) during exercise, peripheral blood oxygen saturation (SpO2) during exercise, dyspnea induced by the exercise, and enjoyment of the exercise. Results: A total of 13 full-text papers corresponding to 12 studies were included in the review. Interventions predominantly used games released for the Nintendo Wii (8 studies) and Microsoft Xbox Kinect (3 studies). There were 5 studies that examined the acute effects of a single session of AVGs and 7 studies that examined the long-term effects after multiple sessions of AVGs. Trials conducted over more than 1 session varied in duration between 3 and 12 weeks. In these, AVG interventions were associated with either similar or slightly greater improvements in outcomes such as exercise capacity when compared with a traditional exercise control, and they also generally demonstrated improvements over baseline or nonintervention comparators. There were a few studies of unsupervised AVG interventions, but the reported adherence was high and maintained throughout the intervention period. Additionally, AVGs were generally reported to be well liked and considered feasible by participants. For outcome measures measured during a single exercise session, there was no significant difference between an AVG and traditional exercise for HR (mean difference 1.44 beats per minute, 95% CI -14.31 to 17.18), SpO2 (mean difference 1.12 percentage points, 95% CI -1.91 to 4.16), and dyspnea (mean difference 0.43 Borg units, 95% CI -0.79 to 1.66), but AVGs were significantly more enjoyable than traditional exercise (Hedges g standardized mean difference 1.36, 95% CI 0.04-2.68). Conclusions: This review provides evidence that AVG interventions, undertaken for several weeks, can provide similar or greater improvements in exercise capacity and other outcomes as traditional exercise. Within a single session of cardiovascular exercise, an AVG can evoke similar physiological responses as traditional exercise modalities but is more enjoyable to subjects with chronic respiratory diseases. However, there is very limited evidence for adherence and effectiveness in long-term unsupervised trials, which should be the focus of future research. © JMIR Serious Games.All right reserved.	https://www.scopus.com/inward/record.uri?eid=2-s	Q2	Scopus	EXC_Scope
Telerehabilitation in subjects with respiratory disease: A scoping	2021	Considering the current coronavirus disease (COVID-19) pandemic, telerehabilitation may be a viable first-line option for patients with respiratory tract disease. To date, there has been no systematic review on telerehabilitation for respiratory tract diseases, including COVID-19. Therefore, this scoping review aimed to determine what telerehabilitation for patients with respiratory tract diseases consists of, how safe telerehabilitation is for patients with respiratory tract diseases, and how feasible telerehabilitation is for hospitalized patients with COVID-19. In May 2020, we conducted a search of the following publication databases on the use of telerehabilita-tion in the treatment of respiratory tract diseases: Medical Literature Analysis and Retrieval System Online, Embase, Cochrane Central Register of Controlled Trials, Cumulative Index to Nursing and Allied Literature, and Physiotherapy Evidence Database. Of the 208 articles identi-fied, 23 studies were subsequently included in this scoping review. In 22 of the included studies, subjects had stable COPD and underwent telerehabilitation at home. The final included study was a case series of subjects with severe acute respiratory syndrome coronavirus 2 infection who underwent telerehabilitation in-hospital. Most telerehabilitation programs consisted of aerobic exercises using a cycle ergometer or a treadmill, walking, and muscle-strengthening exercises. The reported number of adverse events was low, and most studies reported that the average ses-sion adherence rate was > 70%. The majority of the telerehabilitation programs included a face-to-face rehabilitation assessment. Our findings indicate that, in its current state, telerehabilita-tion may be safe and feasible and may lead to reduced face-to-face rehabilitation therapy; in addition, remote rehabilitation assessment should be considered during the COVID-19 pandemic. Further research that targets a more diverse range of respiratory tract diseases and considers telerehabilitation in a hospital setting is required. © 2021 Daedalus Enterprises.	https://www.scopus.com/inward/record.uri?eid=2-s	Q2	Scopus	EXC_PubType
Web Portals for Patients with Chronic Diseases: Scoping Review	2022	Background: The COVID-19 pandemic has required an increased need for rehabilitation activities applicable to patients with chronic diseases. Telerehabilitation has several advantages, including reducing clinic visits by patients vulnerable to infectious diseases. Digital platforms are often used to assist rehabilitation services for patients in remote settings. Although web portals for medical use have existed for years, the technology in telerehabilitation remains a novel method. Objective: This scoping review investigated the functional features and theoretical approaches of web portals developed for telerehabilitation in patients with chronic diseases. Methods: PubMed and Web of Science were reviewed to identify articles associated with telerehabilitation. Of the 477 nonduplicate articles reviewed, 35 involving 14 portals were retrieved for the scoping review. The functional features, targeted diseases, and theoretical approaches of these portals were studied. Results: The 14 portals targeted patients with chronic obstructive pulmonary disease, cardiovascular, osteoarthritis, multiple sclerosis, cystic fibrosis diseases, and stroke and breast cancer survivors. Monitoring/data tracking and communication functions were the most common, followed by exercise instructions and diary/self-report features. Several theoretical approaches, behavior change techniques, and motivational techniques were found to be utilized. Conclusions: The web portals could unify and display multiple types of data and effectively provide various types of information. Asynchronous correspondence was more favorable than synchronous, real-time interactions. Data acquisition often required assistance from other digital tools. Various functions with patient-centered principles, behavior change strategies, and motivational techniques were observed for better support shifting to a healthier lifestyle. These findings suggested that web portals for telerehabilitation not only provided entrance into rehabilitation programs but also reinforced participant-centered treatment, adherence to rehabilitation, and lifestyle changes over time. ©Yuh Morimoto, Tetsuya Takahashi, Ryuichi Sawa, Masakazu Saitoh, Tomoyuki Morisawa, Nobuyuki Kagiyama, Takatoshi Kasai, Birthe Dinesen, Malene Hollingdal, Jens Refsgaard, Hiroyuki Daida.	https://www.scopus.com/inward/record.uri?eid=2-s	Q2	Scopus	EXC_PubType
Remote monitoring of chronic noncommunicable diseases: poter	2022	Aim. To review the current progress in the use of remote health monitoring (RHM) technologies for chronic noncommunicable diseases (CNCD). To search for data, we used Web of Science, Scopus, Russian Science Citation Index, Academic Search Complete (EBSCO), Cochrain, and PubMed databases. The date range was 5-10 years. The importance of development of RHM technologies and their further study was shown to confirm the evidence of effect of certain RHM systems. New approaches to the integration of the medical community into the international telemedicine strategy are considered. It was established that RHM can potentially decrease treatment costs and reduce the burden on medical organizations. The review analyzes the experience in using RHM in patients with cardiovascular diseases, as well as respiratory and endocrine disorders. The review also summarizes and systematizes the findings of studies on assessing the effectiveness of RHM technologies in clinical practice, including their use in the COVID-19 pandemic. It is noted that despite high interest of the scientific community in the study of RHM technologies, unambiguous results demonstrating the effectiveness of such developments in clinical practice have not been presented. © 2022 Siberian State Medical University. All rights reserved.	https://www.scopus.com/inward/record.uri?eid=2-s	Q2	Scopus	EXC_Scope
Digital Inhalers for Asthma or Chronic Obstructive Pulmonary Dis	2021	Impressive advances in inhalation therapy for patients with asthma and chronic obstructive pulmonary disease (COPD) have occurred in recent years. However, important gaps in care remain, particularly relating to poor adherence to inhaled therapies. Digital inhaler health platforms which incorporate digital inhalers to monitor time and date of dosing are an effective disease and medication management tool, promoting collaborative care between clinicians and patients, and providing more in-depth understanding of actual inhaler use. With advances in technology, nearly all inhalers can be digitalized with add-on or embedded sensors to record and transmit data quantitating inhaler actuations, and some have additional capabilities to evaluate inhaler technique. In addition to providing an objective and readily available measure of adherence, they allow patients to interact with the device directly or through their self-management smartphone application such as via alerts and recording of health status. Clinicians can access these data remotely and during patient encounters, to better inform them about disease status and medication adherence and inhaler technique. The ability for remote patient monitoring is accelerating interest in and the use of these devices in clinical practice and research settings. More than 20 clinical studies of digital inhalers in asthma or COPD collectively show improvement in medication adherence, exacerbation risk, and patient outcomes with digital inhalers. These studies support previous findings about patient inhaler use and behaviors, but with greater granularity, and reveal some new findings about patient medication-taking behaviors. Digital devices that record inspiratory flows with inhaler use can guide proper inhaler technique and may prove to be a clinically useful lung function measure. Adoption of digital inhalers into practice is still early, and additional research is needed to determine patient and clinician acceptability, the appropriate place of these devices in the therapeutic regimen, and their cost effectiveness. [MediaObject not available: see fulltext.]. © 2021, The Author(s).	https://www.scopus.com/inward/record.uri?eid=2-s	Q2	Scopus	EXC_Scope

Management challenges in chronic obstructive pulmonary diseas	2021	For the treatment of chronic obstructive pulmonary disease (COPD), early diagnosis and unconditionally correct management at the initial stage of the disease are very important when the symptoms are not yet too worrying. In this way, the progress of the disease can be slowed down, as can the occurrence of late, life-threatening symptoms. Pulmonary rehabilitation is an essential component of the management of COPD. The selection of appropriate exercises, which are determined during the classification of patients into a suitable improvement program, is of key importance in the process of rehabilitation. The coronavirus disease 2019 (COVID-19) pandemic has resulted in major limitations to public health care. Health systems were largely unprepared for an outbreak of this magnitude. Searching for new, attractive technologies that help patients with chronic diseases seems to be justified. This may be driven by telehealth platforms, likewise with the use of virtual reality (VR). Analysis of the available literature indicates promising effectiveness, high patient acceptance, and high motivations to undertake physical activity with the use of such a solution. Thus, the management of patients with COPD during the COVID-19 pandemic should include options for remote delivery of pulmonary rehabilitation, including home-based, telerehabilitation, and computer-based virtual programs. © 2021 by the author. Licensee MDPI, Basel, Switzerland.	https://www.scopus.com/inward/record.uri?eid=2-s	Q2	Scopus	EXC_PubType
Mindfulness-Based Programs for People with Chronic Obstructiv	2020	Objectives: To identify, summarize, and aggregate the quantitative and qualitative evidence on the use of mindfulness-based programs in people with chronic obstructive pulmonary disease (COPD), to describe the possible barriers and facilitators and derive recommendations for the implementation of mindfulness-based programs in people with COPD. Methods: A mixed methods review was conducted following the Joanna Briggs Institute methodology. After a systematic search in eight relevant databases, seven papers presenting five studies were included. Two researchers independently extracted the data and assessed the methodological quality of the studies. Results: No significant changes in levels of anxiety, stress, respiratory symptoms, or other physiological outcomes were found, despite the perception of most participants that mindfulness had a positive influence on their psychological and physical well-being. Only one study showed the effectiveness of mindfulness-based programs in reducing depressive symptoms when combined with pulmonary rehabilitation. Participation in and completion of mindfulness-based programs were hampered by personal beliefs, psychological factors, and practical aspects. Furthermore, the characteristics of the mindfulness-based protocols and the different methods of provision could encourage or discourage program attendance. Conclusions: The limited published studies to date have not demonstrated the efficacy of mindfulness-based programs in COPD. Further methodologically sound studies with bigger sample sizes and with consistent outcome measures are needed to verify their effectiveness. Due to the fluctuations in symptoms of the disease, and patients' difficulties in leaving the house, home-based, web-delivered, and shorter protocols could be further tested as they could facilitate the adherence of people with COPD to mindfulness practice. © 2020, Springer Science+Business Media, LLC, part of Springer Nature.	https://www.scopus.com/inward/record.uri?eid=2-s	Q2	Scopus	EXC_Scope
Telemonitoring Interventions in COPD Patients: Overview of Sys	2020	Objective. The role of telemonitoring interventions (TIs) for chronic obstructive pulmonary disease (COPD) has been studied in many systematic reviews (SRs) and meta-analyses (MAs), but robust conclusions have not been reached due to wide variations in scopes, qualities, and outcomes. The aim of this overview was to determine the effectiveness of TIs on COPD patients. Methods. PubMed, EMBASE, Web of Science, and Cochrane Library were searched for all reviews on the topic of TI in treating COPD from inception to July 8, 2019, without restrictions on language. According to the inclusion and exclusion criteria, the retrieved literature studies were screened to select SRs and MAs of randomized control trials (RCTs) that evaluated the effects of TIs in COPD patients. The methodological quality of SRs and MAs was assessed with the AMSTAR-2 tool, and the strength of evidence was assessed with the grades of recommendations, assessment, development, and evaluation (GRADE) system for concerned outcomes in terms of mortality, quality of life (SGRQ total scores), exercise capacity (6MWD), and exacerbation-related outcomes (hospitalizations, exacerbation rate, and emergency room visits). Results. Our overview included eight SRs and MAs published in 2011 to 2019, from 95 RCTs involving 10632 participants. After strict evaluation by the AMSTAR-2 tool, 75% of the SRs and MAs in this overview had either low or critically low methodological quality. The effects of TIs for COPD on mortality, quality of life, exercise capacity, and exacerbation-related outcomes are limited, and all of these outcomes scored either low or very low quality of evidence on the GRADE system. Conclusions. There might be insufficient evidence to support the effectiveness of TIs for COPD currently, but the results of this overview should be interpreted dialectically and prudently, and the role of TIs in COPD needs further exploration. © 2020 Xuanlin Li et al.	https://www.scopus.com/inward/record.uri?eid=2-s	Q2	Scopus	EXC_PubType
Transitions of care interventions to improve quality of life among	2021	Background: Although transitional care interventions can improve health among patients hospitalized with acute conditions, few interventions use patient quality of life (QOL) as the primary outcome. Existing interventions use a variety of intervention components, are not effective for patients of all races and ethnicities, do not address age-related patient needs, and do not incorporate the needs of families. The purpose of this study was to systematically review characteristics of transitional care intervention studies that aimed to improve QOL for younger adult patients of all race and ethnicities who were hospitalized with acute conditions. Methods: A systematic review was conducted of empirical literature available in PubMed, Embase, CINAHL, and PsycINFO by November 19, 2019 to identify studies of hospital to home care transitions with QOL as the primary outcome. Data extraction on study design and intervention components was limited to studies of patients aged 18–64. Results: Nineteen articles comprising 17 studies met inclusion criteria. There were a total of 3,122 patients across all studies (range: 28–536). Populations of focus included cardiovascular disease, chronic obstructive pulmonary disease, stroke, breast cancer, and kidney disease. Seven QOL instruments were identified. All interventions were multi-component with a total of 31 different strategies used. Most interventions were facilitated by a registered nurse. Seven studies discussed intervention facilitator training and eight discussed intervention materials utilized. No studies specified cultural tailoring of interventions or analyzed findings by racial/ethnic subgroup. Conclusions: Future research is needed to determine which intervention components, either in isolation or in combination, are effective in improving QOL. Future studies should also elaborate on the background and training of intervention facilitators and on materials utilized and may also consider incorporating differences in culture, race and ethnicity into all phases of the research process in an effort to address and reduce any health disparities. © 2021, The Author(s).	https://www.scopus.com/inward/record.uri?eid=2-s	Q2	Scopus	EXC_Scope
Digital health for COPD care: The current state of play	2019	Chronic obstructive pulmonary disease (COPD) imposes a huge burden to our healthcare systems and societies. To alleviate the burden, digital health—"the use of digital technologies for health"—has been recognized as a potential solution for improving COPD care at scale. The aim of this review is to provide an overview of digital health interventions in COPD care. We accordingly reviewed recent and emerging evidence on digital transformation approaches for COPD care focusing on (I) self-management, (II) in-hospital care, (III) post-discharge care, (IV) hospital-at-home, (V) ambient environment, and (VI) public health surveillance. The emerging approaches included digital-technology-enabled homecare programs, electronic records, big data analytics, and environment-monitoring applications. The digital health approaches of telemonitoring, telehealth and mHealth support the self-management, post-discharge care, and hospital-at-home strategy, with prospective effects on reducing acute COPD exacerbations and hospitalizations. Electronic records and classification tools have been implemented; and their effectiveness needs to be further evaluated in future studies. Air pollution concentrations in the ambient environment are associated with declined lung functions and increased risks for hospitalization and mortality. In all the digital transformation approaches, clinical evidence on reducing mortality, the ultimate goal of digital health intervention, is often inconsistent or insufficient. Digital health transformation provides great opportunities for clinical innovations and discovery of new intervention strategies. Further research remains needed for achieving reliable improvements in clinical outcomes and cost-benefits in future studies. © Journal of Thoracic Disease. All rights reserved.	https://www.scopus.com/inward/record.uri?eid=2-s	Q2	Scopus	EXC_PubType
Objectively Measured Physical Activity as a COPD Clinical Trial (	2021	Background: Reduced physical activity is common in COPD and is associated with poor outcomes. Physical activity is therefore a worthy target for intervention in clinical trials; however, trials evaluating physical activity have used heterogeneous methods. Research Question: What is the available evidence on the efficacy and/or effectiveness of various interventions to enhance objectively measured physical activity in patients with COPD, taking into account the minimal preferred methodologic quality of physical activity assessment? Study Design and Methods: In this narrative review, the COPD Biomarker Qualification Consortium (CBQC) task force searched three scientific databases for articles that reported the effect of an intervention on objectively measured physical activity in COPD. Based on scientific literature and expert consensus, only studies with ≥ 7 measurement days and ≥ 4 valid days of ≥ 8 h of monitoring were included in the primary analysis. Results: Thirty-seven of 110 (34%) identified studies fulfilled the criteria, investigating the efficacy and/or effectiveness of physical activity behavior change programs (n = 7), mobile or electronic-health interventions (n = 9), rehabilitative exercise (n = 9), bronchodilation (n = 6), lung volume reduction procedures (n = 3), and other interventions (n = 3). Results are generally variable, reflecting the large differences in study characteristics and outcomes. Few studies show an increase beyond the proposed minimal important change of 600 to 1100 daily steps, indicating that enhancing physical activity levels is a challenge. Interpretation: Only one-third of clinical trials measuring objective physical activity in people with COPD fulfilled the preset criteria regarding physical activity assessment. Studies showed variable effects on physical activity even when investigating similar interventions. © 2021 American College of Chest Physicians	https://www.scopus.com/inward/record.uri?eid=2-s	Q2	Scopus	EXC_Scope

Using telemedicine to monitor the patient with chronic respiratory	2021	Background: Advances in management have improved mortality of individuals with chronic respiratory failure (CRF), leading to an increase in need for long-term oxygen therapy and/or ventilatory support. These individuals require frequent visits and monitoring of their physiological parameters as well as of the functioning of their devices, such as ventilators or oxygen concentrators. Telemedicine is a clinical application of Information Communication Technology connecting patients to specialised care consultants. This narrative review aims to explore the current available telemoni-toring options for individuals with CRF and reported or potential results. Methods: The research focused on EMBASE, CINALH, PubMed, and Scopus databases. Papers published between 2003 and 2021 in English were considered. Results: Different sensors, transmission devices and systems, and interventions are used with promising but not conclusive clinical results. However, legal problems are still unsolved, and economic advantages for health care systems, although potentially high, are still under debate. Conclusions: Telemonitoring systems for individuals with CRF are increasingly used; with promising results still to be clarified, legal, economical and organisational issues must be defined. © 2021 by the authors. Licensee MDPI, Basel, Switzerland.	https://www.scopus.com/inward/record.uri?eid=2-s	Q2	Scopus	EXC_Scope
The Performance of Digital Monitoring Devices for Oxygen Satur	2021	Healthcare access and delivery for individuals with chronic obstructive pulmonary disease (COPD) who live in remote areas or who are susceptible to contracting communicable diseases, such as COVID-19, may be a challenge. Telehealth and remote monitoring devices can be used to overcome this issue. However, the accuracy of these devices must be ensured before forming healthcare decisions based on their outcomes. Therefore, a systematic review was performed to synthesize the evidence on the reliability, validity and responsiveness of digital devices used for tracking oxygen saturation (SpO2) and/or respiratory rate (RR) in individuals with COPD, in remote settings. Three electronic databases were searched: MEDLINE (1996 to October 8, 2020), EMBASE (1996 to October 8, 2020) and CINAHL (1998 to October 8, 2020). Studies were included if they aimed to evaluate one or more measurement properties of a digital device measuring SpO2 or RR in individuals with COPD. Six-hundred and twenty-five articles were identified and after screening, 7 studies matched the inclusion criteria; covering 11 devices measuring SpO2 and/or RR. Studies reported on the reliability (n = 1), convergent validity (n = 1), concurrent validity (n = 2) and predictive validity (n = 2) of SpO2 devices and on the convergent validity (n = 1), concurrent validity (n = 1) and predictive validity (n = 1) of RR devices. SpO2 and RR devices were valid when compared against other respiration monitoring devices but were not precise in predicting exacerbation events. More well-designed measurement studies are needed to make firm conclusions about the accuracy of such devices. Supplemental data for this article is available online at https://doi.org/10.1080/15412555.2021.1945021. © 2021 Taylor & Francis Group, LLC.	https://www.scopus.com/inward/record.uri?eid=2-s	Q2	Scopus	EXC_Scope
The Role of Technology in Adherence to Physical Activity Prograi	2019	Background: The beneficial role of physical activity (PA) to manage the health condition of patients with chronic diseases is well known. However, adherence to PA guidelines in this group is still low. Monitoring and user-interface technology could represent a significant tool to increase exercise adherence to those particular groups who experience difficulties in adhering to regular and substantial physical activity, and could be supportive in increasing the success of PA programs and interventions. This systematic review aimed at evaluating the effect of physical activity monitoring technology in improving adherence to a PA program in patients with chronic diseases experiencing fatigue. Methods: This systematic review was conducted according to PRISMA guidelines. The literature search was performed in Embase, Medline, Biosis, Scopus, and SPORTDiscus. We filtered the literature according to the question: "Does monitoring technology affect adherence to physical activity and exercise programs in patients with chronic diseases perceiving fatigue?". Results: The search resulted in 1790 hits; finally, eight studies were included, with a total number of 205 patients. Study quality was moderate except for one study of high quality. Only three disease types emerged, COPD, HF, and cancer. PA programs were rather short (from 8 to 13 weeks) except for one 3-year-long study. Five studies employed pedometers and two an activity monitor. Three studies based their adherence on steps, the remaining studies focused on active minutes. Adherence was explicitly reported in two studies, and otherwise derived. Four studies showed high adherence levels (85% week-10, 89% week-8, 81% week-13, 105% week-13, 83% average week-1–12) and three low levels (56% week-12, 41% year-2, 14 year-3). Conclusion: The small number of studies identified did not allow to establish whether the use of monitoring technology could improve adherence to PA programs in patients with chronic diseases experiencing fatigue, but the current evidence seems to suggest that this is a field warranting further study, particularly into how monitoring technology can help to engage patients to adhere to PA programs. © 2019, The Author(s).	https://www.scopus.com/inward/record.uri?eid=2-s	Q2	Scopus	EXC_Scope
Vilnius Declaration on chronic respiratory diseases: Multisectoral	2019	Background: Over 1 billion people suffer from chronic respiratory diseases such as asthma, COPD, rhinitis and rhinosinusitis. They cause an enormous burden and are considered as major non-communicable diseases. Many patients are still uncontrolled and the cost of inaction is unacceptable. A meeting was held in Vilnius, Lithuania (March 23, 2018) under the patronage of the Ministry of Health and several scientific societies to propose multisectoral care pathways embedding guided self-management, mHealth and air pollution in selected chronic respiratory diseases (rhinitis, chronic rhinosinusitis, asthma and COPD). The meeting resulted in the Vilnius Declaration that was developed by the participants of the EU Summit on chronic respiratory diseases under the leadership of Euforea. Conclusion: The Vilnius Declaration represents an important step for the fight against air pollution in chronic respiratory diseases globally and has a clear strategic relevance with regard to the EU Health Strategy as it will bring added value to the existing public health knowledge. © 2019 The Author(s).	https://www.scopus.com/inward/record.uri?eid=2-s	Q2	Scopus	EXC_Scope
Understanding factors critical to the implementation of ehealth in	2021	Introduction Canadians are living longer, many with multiple chronic conditions. This population of older, frail Canadians continues to grow in size as do concurrent demands for community-based, outpatient and ambulatory models of care. Ideally, a multifaceted, proactive, planned and integrated care model includes ehealth. Although several factors are known to facilitate the implementation of ehealth in chronic disease management (CDM), for example, adequate support, usability, alignment of programme objectives, there is a growing body of inconclusive evidence on what is critical for implementation. We aim to achieve a fulsome understanding of factors critical to implementation by conducting a realist review - an approach suitable for understanding complex interventions. Our proposed review will identify factors critical to the implementation of ehealth in CDM (heart failure, chronic obstructive pulmonary disease, chronic kidney disease and/or diabetes (type 1 or 2)) without limitations to care setting, language, publication year or geography. Findings will be presented in configurations of contexts, mechanisms and outcomes (CMOs). Methods and analysis A search strategy will be iteratively developed based on the concepts of 'implementation' and 'adoption' of 'ehealth' interventions used within 'CDM' to identify the peer-reviewed and grey literature published before 31 March 2021 from five databases (Medline, Embase, Cochrane, CINAHL and PsychInfo) on ehealth interventions actively involving a healthcare provider for CDM among adults. Data extraction and synthesis will be guided by Realist and Meta-review Evidence Synthesis: Evolving Standards (RAMESES) guidelines informing core concepts of CMOs, and a study output will include a middle-range-theory describing the implementation of ehealth in CDM. Ethics and dissemination Findings will be published in an open-access peer-reviewed journal and presented at relevant conferences. A multistakeholder (patients, caregivers, healthcare providers and practitioners, decision-makers and policy-makers) perspective will be used in our dissemination approach. No formal ethics approval is required for this review. PROSPERO registration number CRD42020208275. ©	https://www.scopus.com/inward/record.uri?eid=2-s	Q2	Scopus	EXC_PubType
Machine Learning for Pulmonary and Critical Care Medicine: A N	2020	Machine learning (ML) is a discipline of computer science in which statistical methods are applied to data in order to classify, predict, or optimize, based on previously observed data. Pulmonary and critical care medicine have seen a surge in the application of this methodology, potentially delivering improvements in our ability to diagnose, treat, and better understand a multitude of disease states. Here we review the literature and provide a detailed overview of the recent advances in ML as applied to these areas of medicine. In addition, we discuss both the significant benefits of this work as well as the challenges in the implementation and acceptance of this non-traditional methodology for clinical purposes. © 2020, The Author(s).	https://www.scopus.com/inward/record.uri?eid=2-s	Q2	Scopus	EXC_PubType
Walking-related digital mobility outcomes as clinical trial endpoin	2020	Introduction Advances in wearable sensor technology now enable frequent, objective monitoring of real-world walking. Walking-related digital mobility outcomes (DMOs), such as real-world walking speed, have the potential to be more sensitive to mobility changes than traditional clinical assessments. However, it is not yet clear which DMOs are most suitable for formal validation. In this review, we will explore the evidence on discriminant ability, construct validity, prognostic value and responsiveness of walking-related DMOs in four disease areas: Parkinson's disease, multiple sclerosis, chronic obstructive pulmonary disease and proximal femoral fracture. Methods and analysis Arksey and O'Malley's methodological framework for scoping reviews will guide study conduct. We will search seven databases (Medline, CINAHL, Scopus, Web of Science, EMBASE, IEEE Digital Library and Cochrane Library) and grey literature for studies which (1) measure differences in DMOs between healthy and pathological walking, (2) assess relationships between DMOs and traditional clinical measures, (3) assess the prognostic value of DMOs and (4) use DMOs as endpoints in interventional clinical trials. Two reviewers will screen each abstract and full-text manuscript according to predefined eligibility criteria. We will then chart extracted data, map the literature, perform a narrative synthesis and identify gaps. Ethics and dissemination As this review is limited to publicly available materials, it does not require ethical approval. This work is part of Mobilise-D, an Innovative Medicines Initiative Joint Undertaking which aims to deliver, validate and obtain regulatory approval for DMOs. Results will be shared with the scientific community and general public in cooperation with the Mobilise-D communication team. Registration Study materials and updates will be made available through the Center for Open Science's OSFRegistry (https://osf.io/k7395). © Author(s) (or their employer(s)) 2020. Re-use permitted under CC BY-NC. No commercial re-use. See rights and permissions. Published by BMJ.	https://www.scopus.com/inward/record.uri?eid=2-s	Q2	Scopus	EXC_PubType

Use, utility and methods of telehealth for patients with COPD in E	2019	Introduction Although the effectiveness of domiciliary monitoring (telehealth) to improve outcomes in chronic obstructive pulmonary disease (COPD) is controversial, it is being used in the National Health Service (NHS). Aim To explore the use of teleheath for COPD across England and Wales, to assess the perceptions of clinicians employing telehealth in COPD and to summarise the techniques that have been used by healthcare providers to personalise alarm limits for patients with COPD enrolled in telehealth programmes. Methods A cross-sectional survey consisting of 14 questions was sent to 230 COPD community services in England and Wales. Questions were designed to cover five aspects of telehealth in COPD: Purpose of use, equipment type, clinician perceptions, variables monitored and personalisation of alarm limits. Results 65 participants completed the survey from 52 different NHS Trusts. 46% of Trusts had used telehealth for COPD, and currently, 31% still provided telehealth services to patients with COPD. Telehealth is most commonly used for baseline monitoring and to allow early detection of exacerbations, with 54% believing it to be effective. The three most commonly monitored variables were oxygen saturation, heart rate and breathlessness. A variety of methods were used to set alarm limits with the majority of respondents believing that at least 40% of alarms were false. Conclusion Around one-third of responded community COPD services are using telehealth, believing it to be effective without robust evidence, with a variety of variables monitored, a variety of hardware and varying techniques to set alarm limits with high false alarm frequencies. © Author(s) (or their employer (s)) 2019.	https://www.scopus.com/inward/record.uri?eid=2-s	Q2	Scopus	EXC_PubType
Comprehensive care for chronic obstructive pulmonary disease	2019	Chronic obstructive pulmonary disease (COPD) is a common chronic disease worldwide and incurs heavy utilization of healthcare resources. Many COPD patients have comorbidities and experience exacerbations in the course of the disease. Correct diagnosis and appropriate disease assessment are essential for clinical management. Comprehensive care for patients with different severity of disease aims to offer personalized treatment to suit individual needs. Patients with recent exacerbations also need extra care for the post-acute and rehabilitation phases. Comprehensive care consists of self-management and pulmonary rehabilitation and involves multiple healthcare providers working together closely to provide formal structured programmes for patients. The setting, professionals involved, content and the duration of programme vary a lot among different comprehensive care models. Some randomized controlled trials suggested there was improvement in quality of life, exercise capacity and reduced hospital admissions for participants in comprehensive care programmes compared with controls. However, other studies showed that such programmes might not confer benefits and might even bring harm. The reason for the differences in clinical effect of programmes might be due to differences in study design, components and subjects involved in the studies. Careful evaluation of each programme is thus mandatory. Further research is needed to evaluate the safety and effectiveness of comprehensive care management for COPD patients, both at the stable and post-acute exacerbation state. © Journal of Thoracic Disease. All rights reserved.	https://www.scopus.com/inward/record.uri?eid=2-s	Q2	Scopus	EXC_Scope
Will remotely based pulmonary rehabilitation water down its effec	2021	Despite numerous benefits, traditional Pulmonary Rehabilitation (PR) as a resource remains underutilized in chronic lung disease. Less than 3% of eligible candidates for PR attend one or more sessions after hospitalization due to many barriers, including the ongoing COVID-19 pandemic. Emerging alternative models of PR delivery such as home-based PR, tele-rehabilitation, web-based PR, or hybrid models could help address these barriers. Numerous studies have tested the feasibility, safety, and efficacy of these methods, but there is wide variability across studies and methods. We conducted a literature review to help determine if these alternative delivery methods watered down the effectiveness of PR. To evaluate the effectiveness of remotely based PR, the authors performed a literature search for randomized controlled trials (RCTs), cohort studies, and case series using PubMed, CINAHL, and Medline to identify relevant articles through 1 May 2021. Twenty-six applicable studies were found in which 11 compared tele-rehabilitation to conventional clinic-based PR; 11 evaluated tele-rehabilitation using the patient's baseline status as control; and four compared tele-rehabilitation to no rehabilitation. Despite the different technologies used across studies, telerehabilitation was found to be both a feasible and an efficacious option for select patients with lung disease. Outcomes across these studies demonstrated similar benefits to traditional PR programs. Thus the existing data does not show that remotely based PR waters down the effectiveness of conventional PR. Use of remotely based PR is a feasible and effective option to deliver PR, especially for patients with significant barriers to conventional clinic-based PR. Additional, well-conducted RCTs are needed to answer the questions regarding its efficacy, safety, cost-effectiveness and who, among patients with COPD and other lung diseases, will derive the maximum benefit. © 2021 by the authors. Licensee MDPI, Basel, Switzerland.	https://www.scopus.com/inward/record.uri?eid=2-s	Q2	Scopus	EXC_Scope
A Systematic Review of the Effectiveness of Telerehabilitation Int	2020	Background: Many elderly people suffer from chronic health conditions and mobility limitations. Therefore, they may benefit from traditional rehabilitation or telerehabilitation interventions as an alternative for this type of services. Objective: The purpose of this study was to compare the effectiveness of telerehabilitation interventions with traditional rehabilitation services for therapeutic purposes in the elderly. Methods: This systematic review was conducted in 2018. The searched databases were Cochrane Library, PubMed, Scopus, Web of Science, Embase, and ProQuest. The search was conducted with no time or language limitation. The selected papers included the randomized clinical trial studies in which elderly people aged 60 and over used telerehabilitation services for treatment purposes. The quality of the studies was evaluated by using the physiotherapy evidence database (PEDro) scale. Data were extracted by using a data extraction form and findings were narratively synthesized. Results: After screening the retrieved papers, eight articles were selected to be included in the study. According to the findings, telerehabilitation was used for the elderly after stroke, chronic obstructive pulmonary disease (COPD), total knee replacement, and in patients with the comorbidity of COPD and chronic heart failure. Overall, in most studies, there was no significant difference between the intervention and control groups and the level of improvements was similar for most outcomes. Conclusion: Telerehabilitation services can be regarded as an alternative to traditional rehabilitation approaches to reduce outpatient resource utilization and improve quality of life. However, more rigorous studies are suggested to investigate the effectiveness of telerehabilitation services for specific diseases or health conditions. © 2020 Georg Thieme Verlag KG Stuttgart New York.	https://www.scopus.com/inward/record.uri?eid=2-s	Q2	Scopus	EXC_Scope
Understanding end-user perspectives of mobile pulmonary rehab	2019	Background: Pulmonary rehabilitation (PR) is an effective intervention for the management of people with chronic respiratory diseases, but the uptake of and adherence to PR programs is low. There is potential for mobile health (mHealth) to provide an alternative modality for the delivery of PR, overcoming many of the barriers contributing to poor attendance to current services. Objective: The objective of this study was to understand the needs, preferences, and priorities of end users for the development of an adaptive mobile PR (mPR) support program. Methods: A mixed methods (qualitative and quantitative) approach was used to assess the needs, preferences, and priorities of the end users (ie, patients with chronic respiratory disorders) and key stakeholders (ie, clinicians working with patients with chronic respiratory disorders and running PR). The formative studies included the following: (1) a survey to understand the preferences and priorities of patients for PR and how mobile technology could be used to provide PR support, (2) ethnographic semistructured interviews with patients with chronic respiratory disorders to gain perspectives on their understanding of their health and potential features that could be included in an mPR program, and (3) key informant interviews with health care providers to understand the needs, preferences, and priorities for the development of an mPR support program. Results: Across all formative studies (patient survey, n=30; patient interviews, n=8; and key stakeholder interviews, n=8), the participants were positive about the idea of an mPR program but raised concerns related to digital literacy and confidence in using technology, access to technology, and loss of social support currently gained from traditional programs. Key stakeholders highlighted the need for patient safety to be maintained and ensuring appropriate programs for different groups within the population. Finding a balance between ensuring safety and maximizing access was seen to be essential in the success of an mPR program. Conclusions: These formative studies found high interest in mHealth-based PR intervention and detailed the potential for an mPR program to overcome current barriers to accessing traditional PR programs. Key considerations and features were identified, including the importance of technology access and digital literacy being considered in utilizing technology with this population. © Rosie Dobson, Pauline Herbst, Sarah Candy, Tamzin Brott, Jeffrey Garrett, Gayl Humphrey, Julie Reeve, Merryn Tawhai, Denise Taylor, Jim Warren, Robyn Whittaker. Originally published in JMIR Formative Research (http://formative.jmir.org),20.12.2019. This is an open-access article distributed under the terms of the Creative Commons Attribution License (https://creativecommons.org/licenses/by/4.0/), which permits unrestricted use, distribution, and reproduction in any medium, provided the original work, first published in JMIR Formative Research, is properly cited. The complete bibliographic information, a link to the original publication on http: //formative.jmir.org, as well as this copyright and license information must be included.	https://www.scopus.com/inward/record.uri?eid=2-s	Q2	Scopus	EXC_PubType
COVID-19 and COPD	2020	[No abstract available]	https://www.scopus.com/inward/record.uri?eid=2-s	Q2	Scopus	EXC_Scope

Developments in smoking cessation interventions for patients wit	2022	Introduction: Smoking cessation is the most effective strategy for slowing the progression of chronic obstructive pulmonary disease (COPD). However, COPD patients find it difficult to quit smoking with standard cessation interventions. Areas covered: A scoping review of smoking cessation for COPD patients was conducted by searching the MEDLINE, Embase, and Cochrane Library databases for all studies published between 1 January 2016 and 22 September 2021. Four themes were set up and 47 studies were included eventually. The majority of the included studies (61.7%, 29/47) investigated efficacy and effectiveness, including new strategies for extended treatment and mobile health (mHealth) delivery approach. Studies examining accessibility and utilization (31.9%, 15/47), safety (10.6%, 5/47), and health economics (6.4%, 3/47) were also reviewed. The quality of the included randomized controlled trials was also evaluated. Expert opinion: Pharmacotherapy combined with behavioral interventions delivered via mHealth may be a promising strategy to help COPD smokers quit. However, the overall quality of the current studies is poor, making it challenging for clinicians to make informed decisions. Future high-quality studies are needed to provide conclusive evidence on the optimal pharmacotherapies and the most cost-effective comprehensive smoking cessation interventions, particularly those integrated into disease management for smokers with COPD. © 2022 Informa UK Limited, trading as Taylor & Francis Group.	https://www.scopus.com/inward/record.uri?eid=2-s	Q2	Scopus	EXC_PubType
Walking on common ground: a cross-disciplinary scoping review	2021	Physical mobility is essential to health, and patients often rate it as a high-priority clinical outcome. Digital mobility outcomes (DMOs), such as real-world gait speed or step count, show promise as clinical measures in many medical conditions. However, current research is nascent and fragmented by discipline. This scoping review maps existing evidence on the clinical utility of DMOs, identifying commonalities across traditional disciplinary divides. In November 2019, 11 databases were searched for records investigating the validity and responsiveness of 34 DMOs in four diverse medical conditions (Parkinson's disease, multiple sclerosis, chronic obstructive pulmonary disease, hip fracture). Searches yielded 19,672 unique records. After screening, 855 records representing 775 studies were included and charted in systematic maps. Studies frequently investigated gait speed (70.4% of studies), step length (30.7%), cadence (21.4%), and daily step count (20.7%). They studied differences between healthy and pathological gait (36.4%), associations between DMOs and clinical measures (48.8%) or outcomes (4.3%), and responsiveness to interventions (26.8%). Gait speed, step length, cadence, step time and step count exhibited consistent evidence of validity and responsiveness in multiple conditions, although the evidence was inconsistent or lacking for other DMOs. If DMOs are to be adopted as mainstream tools, further work is needed to establish their predictive validity, responsiveness, and ecological validity. Cross-disciplinary efforts to align methodology and validate DMOs may facilitate their adoption into clinical practice. © 2021, The Author(s).	https://www.scopus.com/inward/record.uri?eid=2-s	Q2	Scopus	EXC_PubType
Adherence to Pulmonary Rehabilitation in COPD: A QUALITATIV	2019	Purpose: Adherence to pulmonary rehabilitation (PR) is low. This qualitative study used the PRECEDE model to identify predisposing (intrapersonal), reinforcing (interpersonal), and enabling (structural) factors acting as barriers or facilitators of adherence to PR, and elicit recommendations for solutions from patients with chronic obstructive pulmonary disease (COPD). Methods: Focus groups with COPD patients who had attended PR in the past year were conducted. Sessions were recorded, transcribed verbatim, and coded independently by 2 coders, who then jointly decided on the final coding scheme. Data were summarized across groups, and analysis was used a thematic approach with constant comparative method to generate categories. Results: Five focus groups with 24 participants each were conducted. Participants (mean age 62 yr) were 54% male, and 67% black. More than half had annual income less than $20 000, 17% were current smokers, and 54% had low adherence (less than 35% of prescribed PR sessions). The most prominent barriers included physical ailments and lack of motivation (intrapersonal), no support system (interpersonal), transportation difficulties, and financial burden (structural). The most prominent facilitators included health improvement, personal determination (intrapersonal), support from peers, family, and friends (interpersonal), and program features such as friendly staff and educational component of sessions (structural). Proposed solutions included incentives to maintain motivation, tobacco cessation support (intrapersonal), educating the entire family (interpersonal), transportation assistance, flexible program scheduling, and financial assistance (structural). Conclusion: Health limitations, social support, transportation and financial difficulties, and program features impact ability of patients to attend PR. Interventions addressing these interpersonal, intrapersonal, and structural barriers are needed to facilitate adherence to PR. Copyright © 2019 Wolters Kluwer Health, Inc. All rights reserved.	https://www.scopus.com/inward/record.uri?eid=2-s	Q2	Scopus	EXC_PubType
Older Patients' Perspectives of Online Health Approaches in Chr	2019	Background: Chronic obstructive pulmonary disease (COPD) is a complex, chronic condition. Patients commonly have limited access to face-to-face support due to decreased mobility, symptom burden, and availability of services. Online health care approaches provide the potential for increased access to self-management education and support. This study sought to understand older patients with COPD's perspectives of online approaches to health care. Materials and Methods: Participants older than 65 years were recruited from a respiratory service at an academic medical center. Qualitative, focus groups were used and recorded, transcribed verbatim, and analyzed using thematic analysis to identify key and repeated emergent themes. Results: Focus groups were undertaken between January and May 2014. Thematic analysis resulted in five overall themes: (1) concern over risks in the online environment; (2) multimedia and technology use as part of everyday life; (3) online resources as an opportunity for revision of forgotten knowledge; (5) potential for facilitation of decision-making support across geographical and physical barriers; and (4) perceived benefits of online peer support for people with COPD. Conclusions: Overall, these older participants with COPD had positive views of online health information, but did raise the need for guidance to ensure valid and reliable online sources. The capacity for online sources to increase access to decision support and up-to-date information was viewed positively, as was the ability to interact with peers who had similar experiences. Telecommunication tools and approaches are already being utilized in health care interactions. Further research is required into the most appropriate, feasible, and sustainable online health approaches to support patients with chronic illnesses such as COPD. © Copyright 2019, Mary Ann Liebert, Inc., publishers 2019.	https://www.scopus.com/inward/record.uri?eid=2-s	Q2	Scopus	EXC_Scope
The Importance of Self-Management in the Context of Personaliz	2022	Despite current guidelines and decades of evidence on the benefits of a self-management approach, self-management of COPD remains relatively under-utilized in clinical care compared with other chronic diseases. However, self-management interventions can play a valuable role in supporting people with COPD to respond to changing symptoms, and thereby make appropriate decisions regarding the management of their own chronic condition. In this review, we discuss the history and evolution of the concept of self-management, assess current multidisciplinary support programs and clinical interactions designed to optimize self-management, and reflect on how effective these are in terms of clinical and humanistic outcomes. We also evaluate the mechanisms for encouraging change from protocol-based care towards a more personalized care approach, and discuss the role of digital self-management interventions and the importance of addressing health inequalities in COPD treatment, which have been accelerated by the COVID-19 pandemic. Reflecting on the importance of self-management in the context of symptom monitoring and provision of educational support, including information from patient organizations and charities, we discuss the ideal components of a self-management plan for COPD and provide six key recommendations for its implementation: 1) better education for healthcare professionals on disease management and consultation skills; 2) new targets and priorities for patient-focused outcomes; 3) skills gap audits to identify barriers to self-management; 4) best practice sharing within primary care networks and ongoing professional development; 5) enhanced initial consultations to establish optimal self-management from the outset; and 6) negotiation and sharing of self-management plans at the point of diagnosis. © 2022 Cravo et al.	https://www.scopus.com/inward/record.uri?eid=2-s	Q2	Scopus	EXC_PubType
Effectiveness of Health Literacy Interventions on COPD Self-Mar	2021	Chronic obstructive pulmonary disease (COPD) is a chronic progressive lung disease which imposes significant health and economic burdens on societies. Self-management is beneficial in controlling and managing COPD and health literacy (HL) is a major driver of COPD self-management. This review aims to summarize the most recent evidence on the effectiveness of HL driven COPD self-management interventions using randomized controlled trials (RCTs). Eight data bases including Science Citation Index, Academic Search Complete, Social Sciences Citation Index, CINAHL Plus, APA PsycInfo, MEDLINE, Scopus and ScienceDirect were searched to find eligible RCTs assessing the effectiveness of HL interventions on COPD self-management outcomes in outpatient settings between 2008 and February 2020. Ten RCTs met the eligibility criteria. The review found that HL interventions led to moderate improvements in physical activity levels (four out of seven trials) and COPD knowledge (three out of six trials). Surprisingly, none of the RCTs led to significant improvement in medication adherence, which warrants further studies. Furthermore, there were inconclusive findings regarding other COPD self-management outcomes such as smoking cessation, medication adherence, dyspnea, mental health, hospital admissions and health related quality of life. © 2021 Taylor & Francis Group, LLC.	https://www.scopus.com/inward/record.uri?eid=2-s	Q2	Scopus	EXC_Scope

Breathing exercises in people with COPD: A realist review	2021	Aims: To determine the theoretical framework that explains the mechanisms of the success of breathing exercise interventions in people with chronic obstructive pulmonary disease. Design: A realist review. Data sources: Seven bibliographic databases and the grey literature were searched from 2015–January 2020 to identify the studies of breathing exercises. Review Methods: The evaluation criteria of realist review and the mixed method appraisal tool were both used to evaluate the included studies. We extracted and integrated the context–mechanism–outcome strings of each study to form the theoretical framework. Results: Six theoretical mechanisms that affected the success of the intervention were articulated: Wide acceptance of training methods, Integration of the intervention with life, Self-management of the participants, Confidence in controlling symptoms, Participation and support of practitioners, Motivation for intervention. Conversely, the other two mechanisms including the gap between implementation and training and the duration of the intervention, had negative impacts on the implementation of breathing exercises. Conclusion: This review updates and expands the previous literature review on the impact of breathing exercises in people and provides researchers and clinical practitioners with theoretical mechanisms to ensure that the interventions achieve expected effects. Impact: When formulating or selecting breathing exercise interventions, our theoretical framework will guide researchers and clinical practitioners to ensure that the intervention will have practical effects. © 2020 John Wiley & Sons Ltd	https://www.scopus.com/inward/record.uri?eid=2-s	Q2	Scopus	EXC_PubType
Telemedicine Services in Chronic Obstructive Pulmonary Diseas	2021	Background: The current systematic review aimed to determine the effect of telemedicine services on adherence in patients with chronic obstructive pulmonary disease (COPD) and to describe the type of adherence and applied devices and modules. Materials and Methods: We reviewed PubMed, Scopus, Web of Science, and Embase databases to identify relevant studies from the time of inception of these databases to March 10, 2019, using three groups of keywords. The first group comprised words describing COPD, the second group included words describing types of telemedicine interventions, and the third group contained words describing adherence. The reference list of identified articles was also hand-searched to retrieve possibly relevant articles. Results: In total 21 articles were included, in which 13 reported a positive effect for telemedicine on patients' adherence. Adherence to treatment was classified under six categories. The highest frequency belongs to the adherence to performing exercises and participation in training sessions, using the system, using devices, measuring (like blood pressure, oxygen saturation, heart rate, weight, temperature, sputum volume) and reporting symptoms and the results of measurements, completing tasks, and medication. Conclusion: This study demonstrated the effectiveness of telemedicine services on adherence to treatment plans in patients with COPD. The following factors contribute to the effectiveness of telemedicine services: patient support by healthcare professionals and easy access to them, uninterrupted execution of telemedicine programs, follow-up and supervision of providers, creating and maintaining motivation in patients, and provision of different self-management modules. © 2021 NRITLD, National Research Institute of Tuberculosis and Lung Disease, Iran.	https://www.scopus.com/inward/record.uri?eid=2-s	Q2	Scopus	EXC_Scope
Can vital signs recorded in patients' homes aid decision making i	2021	Aim: Use of tele-health programs and wearable sensors that allow patients to monitor their own vital signs have been expanded in response to COVID-19. We aimed to explore the utility of patient-held data during presentation as medical emergencies. Methods: We undertook a systematic scoping review of two groups of studies: studies using non-invasive vital sign monitoring in patients with chronic diseases aimed at preventing unscheduled reviews in primary care, hospitalization or emergency department visits and studies using vital sign measurements from wearable sensors for decision making by clinicians on presentation of these patients as emergencies. Only studies that described a comparator or control group were included. Studies limited to inpatient use of devices were excluded. Results: The initial search resulted in 896 references for screening, nine more studies were identified through searches of references. 26 studies fulfilled inclusion and exclusion criteria and were further analyzed. The majority of studies were from telehealth programs of patients with congestive heart failure or Chronic Obstructive Pulmonary Disease. There was limited evidence that patient held data is currently used to risk-stratify the admission or discharge process for medical emergencies. Studies that showed impact on mortality or hospital admission rates measured vital signs at least daily. We identified no interventional study using commercially available sensors in watches or smart phones. Conclusions: Further research is needed to determine utility of patient held monitoring devices to guide management of acute medical emergencies at the patients' home, on presentation to hospital and after discharge back to the community. © 2021 The Author(s)	https://www.scopus.com/inward/record.uri?eid=2-s	Q2	Scopus	EXC_Scope
Treating copd patients with inhaled medications in the era of covi	2021	COVID-19 has affected millions of patients, caregivers, and clinicians around the world. Severe acute respiratory syndrome coronavirus 2 (SARS-CoV-2) spreads via droplets and close contact from person to person, and there has been an increased concern regarding aerosol drug delivery due to the potential aerosolizing of viral particles. To date, little focus has been given to aerosol drug delivery to patients with COVID-19 treated at home to minimize their hospital utilization. Since most hospitals were stressed with multiple admissions and experienced restricted healthcare resources in the era of COVID-19 pandemic, treating patients with COPD at home became essential to minimize their hospital utilization. However, guidance on how to deliver aerosolized medications safely and effectively to this patient population treated at home is still lacking. In this paper, we provide some strategies and rationales for device and interface selection, delivery technique, and infection control for patients with COPD who are being treated at home in the era of COVID-19 and beyond. © 2021 Ari et al.	https://www.scopus.com/inward/record.uri?eid=2-s	Q2	Scopus	EXC_Scope
Revisiting pulmonary rehabilitation during COVID-19 pandemic: /	2021	There has been an apparent association between the risks of complications with severe acute respiratory syndrome coronavirus 2 (SARSCoV-2) infection in patients with a history of existing chronic respiratory diseases during the pandemic of coronavirus disease 2019 (COVID-19). SARS-CoV-2poses a severe risk in cardiopulmonary management. Moreover, chronic respiratory diseases may further amplify the risk of morbidity and mortality among the afflicted population in the pandemic era. The present review outlines the importance of pulmonary rehabilitation (PR) in persons with chronic respiratory diseases (Chronic obstructive pulmonary disease (COPD) and Asthma) during the COVID-19 era. In this context, amongst the population with a pre-existing pulmonary diagnosis who have contracted SARS-CoV-2, following initial medical management and acute recovery, exercise-based pulmonary rehabilitation (PR) may play a crucial role in long-term management and recovery. The energy conservation techniques will play a pragmatic role in PR of mild to moderate severity cases to counter post-COVID-19 fatigue. Moreover, there is also an urgent need to effectively address post-COVID-19 anxiety and depression, affecting the PR delivery system. © 2021 The Author(s). Published by IMR Press.	https://www.scopus.com/inward/record.uri?eid=2-s	Q2	Scopus	EXC_PubType
Digital interventions for psychological comorbidities in chronic dis	2021	Chronic diseases represent one of the main causes of death worldwide. The integration of digital solutions in clinical interventions is broadly diffused today; however, evidence on their efficacy in addressing psychological comorbidities of chronic diseases is sparse. This systematic review analyzes and synthesizes the evidence about the efficacy of digital interventions on psychological comorbidities outcomes of specific chronic diseases. According to the Preferred Reporting Items for Systematic Reviews and Meta-Analyses (PRISMA) guidelines, a systematic search of PubMed, PsycInfo, Scopus and Web of Science databases was conducted. Only Randomized Controlled Trials (RCTs) were considered and either depression or anxiety had to be assessed to match the selection criteria. Of the 7636 identified records, 17 matched the inclusion criteria: 9 digital interventions on diabetes, 4 on cardiovascular diseases, 3 on Chronic Obstructive Pulmonary Disease (COPD) and one on stroke. Of the 17 studies reviewed, 14 found digital interventions to be effective. Quantitative synthesis highlighted a moderate and significant overall effect of interventions on depression, while the effect on anxiety was small and non-significant. Design elements making digital interventions effective for psychological comorbidities of chronic diseases were singled out: (a) implementing a communication loop with patients and (b) providing disease-specific digital contents. This focus on "how" to design technologies can facilitate the translation of evidence into practice. © 2021 by the authors. Li-censee MDPI, Basel, Switzerland.	https://www.scopus.com/inward/record.uri?eid=2-s	Q2	Scopus	EXC_Scope

Implementation of e-mental health interventions for informal care	2020	Introduction Informal caregivers provide the majority of care to individuals with chronic health conditions, benefiting the care recipient and reducing use of formal care services. However, providing informal care negatively impacts the mental health of many caregivers. E-mental health interventions have emerged as a way to provide accessible mental healthcare to caregivers. Much attention has been given to reviewing the effectiveness and efficacy of such interventions, however, factors related to implementation have received less consideration. Therefore, this mixed-methods systematic review will aim to examine factors associated with the effectiveness and implementation of e-mental health interventions for caregivers. Methods and analysis Eligible studies published since 1 January 2007 will be searched for in several electronic databases (CINAHL Plus with Full Text, the Cochrane Library, EMBASE, PsycINFO, PubMed and Web of Science), clinical trial registries and OpenGrey, with all screening steps conducted by two independent reviewers. Studies will be included if they focus on the implementation or effectiveness of e-mental health interventions designed for informal adult caregivers of adults with cancer, heart disease, stroke, diabetes, dementia or chronic obstructive pulmonary disease. Pragmatic randomised controlled trials quantitatively reporting on caregiver anxiety, depression, psychological distress or stress will be used for a qualitative comparative analysis to identify combinations of conditions that result in effective interventions. Qualitative and quantitative data on implementation of e-mental health interventions for caregivers will be integrated in a thematic synthesis to identify barriers and facilitators to implementation. These results will inform future development and implementation planning of e-mental health interventions for caregivers. Ethics and dissemination Ethical approval is not required for this study as no primary data will be collected. Results will be disseminated in the form of a scientific publication and presentations at academic conferences and plain language summaries for various stakeholders. PROSPERO registration number CRD42020155727. © Author(s) (or their employer(s)) 2020. Re-use permitted under CC BY. Published by BMJ.	https://www.scopus.com/inward/record.uri?eid=2-s	Q2	Scopus	EXC_PubType
Pulmonary Rehabilitation	2020	Pulmonary rehabilitation (PR) is an essential intervention in the management of patients with chronic obstructive pulmonary disease. To guide health care professionals in the implementation and evaluation of a PR program, this article discusses the current key concepts regarding exercise testing, prescription, and training, as well as self-management intervention as essential parts of PR and post-rehabilitation maintenance. Moreover, new approaches (alternative forms of organization and delivery, tele-rehabilitation, exercise adjuncts) and unique and challenging situations (patients experiencing acute exacerbations, advanced disease) are thoroughly reviewed. Finally, validated point-of-care resources and online tools are provided. © 2020	https://www.scopus.com/inward/record.uri?eid=2-s	Q2	Scopus	EXC_Scope
A sense of belonging: A meta-ethnography of the experience of p	2019	Aim: To synthesize the qualitative research in the literature addressing how patients with chronic obstructive pulmonary disease experience care received by telemedicine. Design: Meta-ethnography. Data Sources: Twelve studies, published from 2013 – 2018, were identified by a search of relevant systematic databases in June 2017, including updated searches performed in June 2018. Review Methods: The studies were reviewed and critically appraised independently by three researchers. The review followed the seven steps of meta-ethnography developed by Noblit and Hare, including a line-of-argument synthesis. Results: The synthesis revealed three second-order constructs: presence, transparency, and ambivalence. Using a line-of-argument synthesis, a model was developed that showed patients' experience of a sense of belonging when receiving care by telemedicine. Conclusion: This meta-ethnography contributes to the existing and contradictory evidence base of telemedicine to chronic obstructive pulmonary disease patients. It addresses and adds renewed understanding of who would benefit from telemedicine and why, by illustrating the interrelationship between the conditions of telemedicine care, the severity of COPD, and the need for connectedness and emphasizes that the need to belong in telemedicine care increases with the progression of illness burden and severity. Impact: The present study endorses the view that the patients with a severe illness burden are likely to benefit the most when receiving care by telemedicine. However, the benefits rely on the fact that the telemedicine interventions involve emotional, social, and clinical support, including regular contact with healthcare professionals, to meet the requirements to belong. © 2019 John Wiley & Sons Ltd	https://www.scopus.com/inward/record.uri?eid=2-s	Q2	Scopus	EXC_PubType
Effects of Home-Based Pulmonary Rehabilitation on Dyspnea, E	2021	Conventional pulmonary rehabilitation programs are used as therapies for the treatment of chronic obstructive pulmonary disease (COPD). However, this modality presents barriers that make rehabilitation difficult. For this reason, home-based pulmonary rehabilitation (HBPR) has been used to overcome these barriers. The objective was to systematically compare a structured program with HBPR or a control group for participants with COPD. The primary outcome was an improvement in symptoms in the level of dyspnea and secondary outcomes were parameters in lung function, exercise capacity, health-related quality of life (HRQoL) and the impact of the disease on the individual. The Medline (via PubMed), Virtual Health Library and Cochrane Library databases were searched until May 10, 2021. Randomized controlled trials were included without restrictions on the year of publication or language. The risk of bias was evaluated using the Cochrane risk-of-bias tool for randomized trials (RoB). Our results showed that there was a significant decrease in the level of dyspnea, (MD: 5.46; 95% CI: 1.97 to 8.96), increased distance covered (MD: 61.75; 95% CI: 42, 94 to 80.56, significant improvement in HRQoL (MD: −11.30; 95% CI: −19.81 to −2.79) and reduction in the impact of the disease (DM: −4.71; 95% CI: −7.95 to −1.47). All results found were comparing the intervention group versus the control group. To conclude we found a reduction in the levels of dyspnea, an increase in the distance covered on the six-minute walk test, improving HRQoL and decreasing the impact of the disease in COPD patients in home-based pulmonary rehabilitation. © 2022 The Author(s). Published with license by Taylor & Francis Group, LLC.	https://www.scopus.com/inward/record.uri?eid=2-s	Q2	Scopus	EXC_Scope
Role for artificial intelligence in respiratory diseases-chronic obst	2021	Chronic obstructive pulmonary disease (COPD) is a global healthcare challenge. It is highly prevalent in low-income countries, causes 3 million death per year and is projected to be the leading cause of death globally by 2030. Challenges in COPD management result in care quality gaps which impair timely and accurate diagnosis and limit patient stratification and provision of evidence-based interventions. COPD exacerbations are responsible for a large proportion of the disease-burden, adverse outcomes and healthcare costs. There is a requirement to re-orientate COPD exacerbation care from failure-driven reactive approach to one based on proactive preventative management. Service model adaptation supported by artificial intelligence (AI) tools offer the prospect of addressing these care-quality gaps and achieving this practice re-orientation. Progress with clinical applications of AI for COPD is accelerating. Evidence available demonstrates the potential of AI techniques to facilitate early and precise COPD case-finding and diagnosis, allow stratification with clinical decision support to prioritise management, and achieve accurate exacerbation detection/prediction to allow proactive interventions. In this narrative review, we will summarise current evidence for the application of AI to these COPD challenges, outline the barriers to implementation of AI models and present our opinion on the required next steps to realise the potential role for AI in COPD management. © Journal of Hospital Management and Health Policy. All rights reserved.	https://www.scopus.com/inward/record.uri?eid=2-s	Q2	Scopus	EXC_Scope
Beyond forest plots: Clinical gestalt and its influence on COPD te	2019	Background Chronic obstructive pulmonary disease (COPD) is a progressive chronic condition. Improvements in therapies have resulted in better patient outcomes. The use of technology such as telemonitoring as an additional intervention is aimed at enhancing care and reducing unnecessary acute hospital service use. The influence of verbal communication between health staff and patients to inform decision making regarding use of acute hospital services within telemonitoring studies has not been assessed. Method A systematic overview of published systematic reviews of COPD and telemonitoring was conducted using an a priori protocol to ascertain the impact of verbal communication in telemonitoring studies on health service outcomes such as emergency department attendances, hospitalisation and hospital length of stay. The search of the following electronic databases: Cochrane Library, Medline, Pubmed, CINAHL, Embase, TROVE, Australian Digital Thesis and Proquest International Dissertations and Theses was conducted in 2017 and updated in September 2019. Results Six systematic reviews were identified. All reviews involved home monitoring of COPD symptoms and biometric data. Included reviews reported 5-28 studies with sample sizes ranging from 310 to 2891 participants. Many studies reported in the systematic reviews were excluded as they were telephone support, cost effectiveness studies, and/or did not report the outcomes of interest for this overview. Irrespective of group assignment, verbal communication with the health or research team did not alter the emergency attendance or hospitalisation outcome. The length of stay was longer for those who were assigned home telemonitoring in the majority of studies. Conclusion This overview of telemonitoring for COPD had small sample sizes and a wide variety of included studies. Communication was not consistent in all included studies. Understanding the context of communication with study participants and the decision-making process for referring patients to various health services needs to be reported in future studies of telemonitoring and COPD. © © Author(s) (or their employer (s)) 2019. Re-use permitted under CC BY-NC. No commercial re-use. See rights and permissions. Published by BMJ.	https://www.scopus.com/inward/record.uri?eid=2-s	Q2	Scopus	EXC_PubType
Pulmonary Rehabilitation: From Theory to Practice; [Rehabilitaci	2022	Pulmonary rehabilitation (PR) has been seen to be useful not only in chronic obstructive pulmonary disease, but also in other non-chronic obstructive pulmonary disease respiratory diseases. It is highly effective in improving dyspnea, exercise tolerance, and health-related quality of life, and is supported by a high level of evidence and grade of recommendation. In recent years, PR has been shown to be equally effective in both the hospital and home setting, and can even be used in telemedicine. The recommended timing of PR after an exacerbation has also changed following evidence that early intervention is more beneficial and has no negative impact on side effects. However, to achieve maximum effect while avoiding risk, each patient must be evaluated by the PR team, and rehabilitation must be tailored to their needs and capabilities. In recent years, new, simpler strategies have been put forward to give all potential candidates access to PR. This approach should help achieve greater adherence to rehabilitation programs and maintain long-term benefits, primarily by influencing patient lifestyles and encouraging physical activity. © 2021 Sociedad Española de Neumología y Cirugía Torácica (SEPAR)	https://www.scopus.com/inward/record.uri?eid=2-s	Q2	Scopus	EXC_Scope

Role of new digital technologies and telemedicine in pulmonary r	2021	Background: Asthma and chronic obstructive pulmonary diseases are conditions characterized by a variable progression. Some individuals experience longer asymptomatic periods while others acute worsening periods and/or exacerbations triggered by symptom multiplication factors. Medications are adjusted to the patients' respiratory function, self-assessment of health and emerging certain physical changes. A more effective treatment may be applied by real-time data registered during the patient's everyday life. Aim and methods: Introducing new modern digital technology in pulmonary rehabilitation (PR) to help tracking the patients' medication, thus we systematically reviewed the latest publications on telemedicine and pulmonary telerehabilitation. Conclusion: The use of the latest digital technologies in PR is very exciting and offers great opportunities while treating patients affected by specific conditions. On the one hand, adherence to medication can be improved in patients with chronic respiratory diseases by using these new state of the art devices; on the other hand, digital devices will also be able to monitor various physiological parameters of patients during their usual everyday activities. Data can be stored on a smartphone and shared with the provider. Relying on this information, physicians will be able to tailor medications and dosage to the specific needs of individual patients. Telerehabilitation may be a sustainable solution to the growing burden of chronic respiratory disease worldwide. However, PR must keep its cornerstones, such as education and motivations, which are most successful when conducted in person. Many issues remain to be resolved in the future, e.g. cybersecurity while using smart devices since they offer unique opportunities for PR. © 2021, The Author(s).	https://www.scopus.com/inward/record.uri?eid=2-s	Q2	Scopus	EXC_PubType
Pre-hospital and emergency department pathways of care for ex	2019	Exacerbations are serious complications of chronic obstructive pulmonary disease (COPD) that often require acute care from pre-hospital and emergency department (ED) services. Despite being a frequent cause of emergency presentations, gaps remain in both literature and practice for emergency care pathways of COPD exacerbations. This review seeks to address these gaps and focuses on the literature of prehospital and ED systems of care and how these intersect with patients experiencing an exacerbation of COPD. The literature in this area is expanding rapidly; however, more research is required to further understand exacerbations and how they are addressed by emergency medical services worldwide. For the purpose of this review, the pre-hospital domain includes ambulance and other emergency transport services, and encompasses medical interventions delivered prior to arrival at an ED or hospital. The ED domain is defined as the area of a hospital or free-standing centre where patients arrive to receive emergent medical care prior to admission. In many studies there is a significant overlap between these two domains and frequent intersection and collaboration between services. In both of these domains, for the management of COPD exacerbations, several overarching themes have been identified in the literature. These include: the appropriate delivery of oxygen in the emergency setting; strategies to improve the provision of care in accordance with diagnostic and treatment guidelines; strategies to reduce the requirement for emergency presentations; and, technological advances including machine learning which are helping to improve emergency healthcare systems. © Journal of Thoracic Disease. All rights reserved.	https://www.scopus.com/inward/record.uri?eid=2-s	Q2	Scopus	EXC_Scope
What Are the Experiences of People with COPD Using Activity M	2022	Physical activity monitoring technology (e.g. smartphone apps or wearables) can objectively record physical activity levels, potentially support interventions to increase activity levels, and support the self-management of Chronic Obstructive Pulmonary Disease (COPD). Insight into patients' experiences of monitoring physical activity is needed to inform future healthcare practice and policy utilizing this technology to support long-term positive health behavior change. This scoping review aimed to explore the experiences of using technology for monitoring physical activity among people with COPD. The Joanna Briggs Institute scoping review methodological framework was used. Relevant scientific databases (CINAHL Complete, MEDLINE, PsycINFO, SPORTDiscus, Cochrane Library and Scopus) were searched from 1st January 2016 to 16th March 2021. Thematic synthesis was used to analyze the data. Twelve studies exploring the experiences of people with COPD using technology for monitoring physical activity were included in the synthesis. Seven themes were developed and summarize experiences: 1) Monitoring and keeping track of their activity and health, 2) Supporting motivation to be active, 3) Acceptability of the device, 4) Experiencing technical issues with the device, 5) Setting appropriate and achievable goals for their health condition, 6) Integrating the device into their life and daily routine, and 7) Perceived physical and psychological benefits of using the device. Further high-quality research is needed to understand the experiences of people with COPD using technology to monitor physical activity in everyday life and better self-manage their health condition. Supporting people with COPD to monitor their physical activity could enable them to better self-manage their health condition. © 2022 The Author(s). Published with license by Taylor & Francis Group, LLC.	https://www.scopus.com/inward/record.uri?eid=2-s	Q2	Scopus	EXC_PubType
Telemedicine in COPD: An Overview by Topics	2020	COPD is a major cause of morbidity and mortality worldwide and carries a huge and growing economic and social burden. Telemedicine might allow the care of patients with limited access to health services and improve their self-management. During the COVID-19 pandemic, patient's safety represents one of the main reasons why we might use these tools to manage our patients. The authors conducted a literature search in MEDLINE database. The retrieval form of the Medical Subject Headings (Mesh) was ((Telemedicine OR Tele-rehabilitation OR Telemonitoring OR mHealth OR Ehealth OR Telehealth) AND COPD). We only included systematic reviews, reviews, meta-analysis, clinical trials and randomized-control trials, in the English language, with the selected search items in title or abstract, and published from January 1st 2015 to 31st May 2020 (n = 56). There was a positive tendency toward benefits in tele-rehabilitation, health-education and self-management, early detection of COPD exacerbations, psychosocial support and smoking cessation, but the heterogeneity of clinical trials and reviews limits the extent to which this value can be understood. Telemonitoring interventions and cost-effectiveness had contradictory results. The literature on teleconsultation was scarce during this period. The non-inferiority tendency of telemedicine programmes comparing to conventional COPD management seems an opportunity to deliver quality healthcare to COPD patients, with a guarantee of patient's safety, especially during the COVID-19 outbreak. © 2020 Taylor & Francis Group, LLC.	https://www.scopus.com/inward/record.uri?eid=2-s	Q2	Scopus	EXC_PubType
Digital health interventions for chronic diseases: A scoping reviev	2020	Background Monitoring and evaluations of digital health (DH) solutions for the management of chronic diseases are quite heterogeneous and evidences around evaluating frameworks are inconsistent. An evidenced-based framework is needed to inform the evaluation process and rationale of such interventions. We aimed to explore the nature, extent and components of existing DH frameworks for chronic diseases. Methods This review was conducted based on the five steps of Arksey and O'Malley's scoping review methodology. Out of 172 studies identified from, PubMed, Embase and Web of Science, 11 met our inclusion criteria. The reviewed studies developed DH frameworks for chronic diseases and published between 2010 and 2018. Results According to WHO guidelines for monitoring and evaluation of DH interventions, we identified seven Conceptual frameworks, two Results frameworks, one Logical framework and one Theory of change. The frameworks developed for providing interventions such as self-management, achieving personal goals and reducing relapse for cardiovascular disease, diabetes, chronic obstructive pulmonary disease and severe mental health. A few studies reported evaluation of the frameworks using randomised clinical trials (n=3) and feasibility testing via Likert scale survey (n=2). A wide range of outcomes were reported including access to care, cost-effectiveness, behavioural outcomes, patient-provider communications, technology acceptance and user experience. Conclusion There is a lack of evidence on the application of consistent DH frameworks. Future research should address the use of evidence-based frameworks into the research design, monitoring and evaluation process. This review explores the nature of DH frameworks for the management of chronic diseases and provides examples to guide monitoring and evaluation of interventions. Author(s) (or their employer(s)) 2020. © 2020 Copernicus GmbH. All rights reserved.	https://www.scopus.com/inward/record.uri?eid=2-s	Q2	Scopus	EXC_PubType
Chronic obstructive pulmonary disease treatment and pharmacis	2021	Chronic obstructive pulmonary disease (COPD) is the fourth leading cause of death across the globe. Its repeated exacerbation will seriously worsen the quality of life, aggravate the patients' symptoms, and bring a heavy burden on the patients and the society. Understanding the current status of drug therapy and the role of pharmaceutical care is essential for the management of COPD. In addition to the drugs already on the market, recent clinical trials also show that emerging novel drugs for treating COPD are being developed to prevent the symptoms, reduce the frequency of acute exacerbation, and improve the quality of life. Recent progress in new drug research should lead to novel treatment options for COPD patients in future clinical practice. The pharmaceutical care has shown significantly favourable impacts on addressing drug-related problems, supporting its vital role in the management of COPD, especially when there are a wide range of therapeutic agents. This review not only provides an overview of current treatment strategies but also further underlines the importance of new drug development and pharmaceutical care for patients with COPD. © 2021 Li et al.	https://www.scopus.com/inward/record.uri?eid=2-s	Q2	Scopus	EXC_Scope

Telehealth in chronic disease management and the role of the In	2020	Introduction: Telehealth is used to remotely and timely deliver clinical care, and its effectiveness for managing the most common chronic diseases has been proved by several studies. Areas covered: Tholomeus® is a web-based clinically validated and certified telehealth solution operating in the context of the Internet-of-Medical-Things. It favors closed-loop connectivity between patients and caregivers, according to multidisciplinary and multifaceted interventions. Evidence collected in the last decade in 1,471 healthcare facilities and 135,333 patients has documented the usefulness of the service for improving access to care, and enhance screening and management of arterial hypertension, heart disease, chronic obstructive pulmonary disease, and obstructive sleep apnea. In addition to professional diagnostic tests, an AndroidTM Tholomeus® app used by 3,654 consumers in the last three years has helped document a high prevalence of impaired glucose tolerance, overweight or obesity, dyslipidemia, or uncontrolled blood pressure among users. Expert opinion: The telehealth approach to chronic disease management is currently characterized by a high heterogeneity of solutions, often not supported by robust evidence for clinical efficacy and safety. The Tholomeus® solution seems to satisfy the current recommendations of software as a medical device solution, although further clinical evidence needs to be collected in prospective studies. © 2020 Informa UK Limited, trading as Taylor & Francis Group.	https://www.scopus.com/inward/record.uri?eid=2-s	Q2	Scopus	EXC_PubType
Rehabilitation, the great absentee of virtual coaching in medical c	2019	Background: In the last few years, several studies have focused on describing and understanding how virtual coaches (ie, coaching program or smart device aiming to provide coaching support through a variety of application contexts) could be key drivers for health promotion in home care settings. As there has been enormous technological progress in the field of artificial intelligence and data processing in the past decade, the use of virtual coaches gains an augmented attention in the considerations of medical innovations. Objective: This scoping review aimed at providing an overview of the applications of a virtual coach in the clinical field. In particular, the review focused on the papers that provide tangible information for coaching activities with an active implication for engaging and guiding patients who have an ongoing plan of care. Methods: We aimed to investigate the use of the term virtual coach in the clinical field performing a methodical review of the relevant literature indexed on PubMed, Scopus, and Embase databases to find virtual coach papers focused on specific activities dealing with clinical or medical contexts, excluding those aimed at surgical settings or electronic learning purposes. Results: After a careful revision of the inclusion and exclusion criteria, 46 records were selected for the full-text review. Most of the identified articles directly or indirectly addressed the topic of physical activity. Some papers were focused on the use of virtual coaching (VC) to manage overweight or nutritional issues. Other papers dealt with technological interfaces to facilitate interactions with patients suffering from different chronic clinical conditions such as heart failure, chronic obstructive pulmonary disease, depression, and chronic pain. Conclusions: Although physical activity is a healthy practice that is most encouraged by a virtual coach system, in the current scenario, rehabilitation is the great absentee. This paper gives an overview of the tangible applications of this tool in the medical field and may inspire new ideas for future research on VC. © Peppino Tropea, Hannes Schlieter, Irma Sterpi, Elda Judica, Kai Gand, Massimo Caprino, Inigo Gabilondo, Juan Carlos Gomez-Esteban, Stefan Busnatu, Crina Sinescu, Sofoklis Kyriazakos, Sadia Anwar, Massimo Corbo.	https://www.scopus.com/inward/record.uri?eid=2-s	Q2	Scopus	EXC_PubType
Effectiveness of telemonitoring for respiratory and systemic symp	2021	Asthma and chronic obstructive pulmonary diseases (COPD) are highly prevalent chronic lung diseases that require ongoing self-management, which itself is often suboptimal. Therefore, telemonitoring has been used to help patients measure their symptoms, share data with healthcare providers and receive education and feedback to improve disease management. In this study, we conducted a narrative review of recent evidence on the effectiveness of telemonitoring for asthma and COPD in adults. Of the thirteen identified studies, eleven focused on COPD and two focused on asthma. All studies were reviewed, and effects were compared between intervention and care as usual groups. Of the study interventions, seven showed a positive outcome on at least one outcome measure, and six had no significant results on any of the outcome measures. All of the interventions with a positive outcome included an educational component, while only one of the six interventions without positive outcomes included an educational component. We conclude that telemonitoring interventions for asthma and COPD seem more effective if they included an educational component regarding different aspects of self-management. © 2021 by the authors. Licensee MDPI, Basel, Switzerland.	https://www.scopus.com/inward/record.uri?eid=2-s	Q2	Scopus	EXC_PubType
Pulmonary rehabilitation for COPD: A narrative review and call fo	2021	Chronic obstructive pulmonary disease (COPD) is a common, preventable, and treatable condition, in which outcomes can be improved with careful management. Pulmonary rehabilitation (PR) comprises exercise and education, delivered by multidisciplinary teams. PR is a cost-effective management strategy in COPD patients which improves exercise performance, reduces dyspnea, reduces the risk of exacerbation, and improves health-related quality of life. All COPD patients appear to benefit irrespective of their baseline function, and PR has also been shown to be a clinically and cost-effective management approach following an acute exacerbation. COPD patients with greater disability and those recovering postexacerbation should be specifically targeted for PR. Due to limited current capacity, the latter group may not currently be able to benefit from PR. Therefore, there is a need for the wider implementation of PR services in Saudi Arabia, requiring us to address challenges including capacity and workforce competency. © 2021 Wolters Kluwer Medknow Publications. All rights reserved.	https://www.scopus.com/inward/record.uri?eid=2-s	Q2	Scopus	EXC_PubType
Self-management interventions in COPD patients with multimorb	2019	[No abstract available]	https://www.scopus.com/inward/record.uri?eid=2-s	Q2	Scopus	EXC_PubType
Using temporal features to provide data-driven clinical early warr	2019	Background: Both chronic obstructive pulmonary disease (COPD) and asthma incur heavy health care burdens. To support tailored preventive care for these 2 diseases, predictive modeling is widely used to give warnings and to identify patients for care management. However, 3 gaps exist in current modeling methods owing to rarely factoring in temporal aspects showing trends and early health change: (1) existing models seldom use temporal features and often give late warnings, making care reactive. A health risk is often found at a relatively late stage of declining health, when the risk of a poor outcome is high and resolving the issue is difficult and costly. A typical model predicts patient outcomes in the next 12 months. This often does not warn early enough. If a patient will actually be hospitalized for COPD next week, intervening now could be too late to avoid the hospitalization. If temporal features were used, this patient could potentially be identified a few weeks earlier to institute preventive therapy; (2) existing models often miss many temporal features with high predictive power and have low accuracy. This makes care management enroll many patients not needing it and overlook over half of the patients needing it the most; (3) existing models often give no information on why a patient is at high risk nor about possible interventions to mitigate risk, causing busy care managers to spend more time reviewing charts and to miss suited interventions. Typical automatic explanation methods cannot handle longitudinal attributes and fully address these issues. Objective: To fill these gaps so that more COPD and asthma patients will receive more appropriate and timely care, we will develop comprehensible data-driven methods to provide accurate early warnings of poor outcomes and to suggest tailored interventions, making care more proactive, efficient, and effective. Methods: By conducting a secondary data analysis and surveys, the study will: (1) use temporal features to provide accurate early warnings of poor outcomes and assess the potential impact on prediction accuracy, risk warning timeliness, and outcomes; (2) automatically identify actionable temporal risk factors for each patient at high risk for future hospital use and assess the impact on prediction accuracy and outcomes; and (3) assess the impact of actionable information on clinicians' acceptance of early warnings and on perceived care plan quality. Results: We are obtaining clinical and administrative datasets from 3 leading health care systems' enterprise data warehouses. We plan to start data analysis in 2020 and finish our study in 2025. Conclusions: Techniques to be developed in this study can boost risk warning timeliness, model accuracy, and generalizability; improve patient finding for preventive care; help form tailored care plans; advance machine learning for many clinical applications; and be generalized for many other chronic diseases. © Gang Luo, Bryan L Stone, Corinna Koebnick, Shan He, David H Au, Xiaoming Sheng, Maureen A Murtaugh, Katherine A Sward, Michael Schatz, Robert S Zeiger, Giana H Davidson, Flory L Nkoy. Originally published in JMIR Research Protocols (http://www.researchprotocols.org), 06.06.2019.	https://www.scopus.com/inward/record.uri?eid=2-s	Q2	Scopus	EXC_PubType

Health equity in the effectiveness of web-based health interventic	2020	Background: Web-based self-care interventions have the potential to reduce health inequalities by removing barriers to access to health care. However, there is a lack of evidence about the equalizing effects of these interventions on chronic conditions. Objective: This study investigated the differences in the effectiveness of web-based behavioral change interventions for the self-care of high burden chronic health conditions (eg, asthma, chronic obstructive pulmonary disease [COPD], diabetes, and osteoarthritis) across socioeconomic and cultural groups. Methods: A systematic review was conducted, following Cochrane review guidelines. We conducted searches in Ovid Medical Literature Analysis and Retrieval System Online and Cumulative Index to Nursing and Allied Health Literature databases. Studies with any quantitative design were included (published between January 1, 2006, and February 20, 2019) if they investigated web-based self-care interventions targeting asthma, COPD, diabetes, and osteoarthritis; were conducted in any high-income country; and reported variations in health, behavior, or psychosocial outcomes across social groups. Study outcomes were investigated for heterogeneity, and the possibility of a meta-analysis was explored. A narrative synthesis was provided together with a novel figure that was developed for this review, displaying heterogeneous outcomes. Results: Overall, 7346 records were screened and 18 studies were included, most of which had a high or critical risk of bias. Important study features and essential data were often not reported. The meta-analysis was not possible due to the heterogeneity of outcomes. There was evidence that intervention effectiveness was modified by participants' social characteristics. Minority ethnic groups were found to benefit more from interventions than majority ethnic groups. Single studies with variable quality showed that those with higher education, who were employed, and adolescents with divorced parents benefited more from interventions. The evidence for differences by age, gender, and health literacy was conflicting (eg, in some instances, older people benefited more, and in others, younger people benefited more). There was no evidence of differences in income, numeracy, or household size. Conclusions: There was evidence that web-based self-care interventions for chronic conditions can be advantageous for some social groups (ie, minority ethnic groups, adolescents with divorced parents) and disadvantageous for other (ie, low education, unemployed) social groups who have historically experienced health inequity. However, these findings should be treated with caution as most of the evidence came from a small number of low-quality studies. The findings for gender and health literacy were mixed across studies on diabetes, and the findings for age were mixed across studies on asthma, COPD, and diabetes. There was no evidence that income, numeracy, or the number of people living in the household modified intervention effectiveness. We conclude that there appear to be interaction effects, which warrant exploration in future research, and recommend a priori consideration of the predicted interaction effects. © Sophie Turnbull, Christie Cabral, Alastair Hay, Patricia J Lucas. Originally published in the Journal of Medical Internet Research (http://www.jmir.org), 05.06.2020. This is an open-access article distributed under the terms of the Creative Commons Attribution License (https: //creativecommons.org/licenses/by/4.0/), which permits unrestricted use, distribution, and reproduction in any medium, provided the original work, first published in the Journal of Medical Internet Research, is properly cited. The complete bibliographic information, a link to the original publication on http://www.jmir.org/, as well as this copyright and license information must be included.	https://www.scopus.com/inward/record.uri?eid=2-s	Q2	Scopus	EXC_Scope
Effectiveness of telemedicine intervention for chronic obstructive	2020	Background: Telemedicine market in China has been rapidly developing. However, no systematic review has been published in China. Details of the implementation of telemedicine interventions in the chronic obstructive pulmonary disease (COPD) in China have not been described, and the effectiveness of telemedicine interventions is still unclear. Therefore, in this review, we describe the implementation details of telemedicine intervention in China and access the efficacy of telemedicine. Materials and Methods: A literature search was conducted in Embase, Cochrane Library, PubMed, China National Knowledge Infrastructure (CNKI), Wan Fang Data, and China Science and Technology Journal Database by July 9, 2018. Results: A total number of 24 studies were meta-analyzed. There are many differences during the implementation of telemedicine in China. Quality of life in the group of the telemedicine intervention was better than that in the control group (mean difference = -4.93 [95% confidence interval; CI -6.86 to -3.01], p < 0.00001), but the heterogeneity is high (I2 = 86%, p = 0.0001). The rates of hospitalization were lower than those in the control group (odds ratio = 0.24 [95% CI 0.20-0.29], p < 0.00001), and the heterogeneity was low (I2 = 25%, p = 0.14). Conclusion: The implementation of telemedicine in China has not yet been standardized. Nonetheless, results of our review indicated that telemedicine in China can improve the quality of life and reduce the rates of hospitalization in COPD patients. © Copyright 2020, Mary Ann Liebert, Inc., publishers 2020.	https://www.scopus.com/inward/record.uri?eid=2-s	Q2	Scopus	EXC_Scope
Assessing the usability of wearable devices to measure gait and	2021	Background: The World Health Organisation's global strategy for digital health emphasises the importance of patient involvement. Understanding the usability and acceptability of wearable devices is a core component of this. However, usability assessments to date have focused predominantly on healthy adults. There is a need to understand the patient perspective of wearable devices in participants with chronic health conditions. Methods: A systematic review was conducted to identify any study design that included a usability assessment of wearable devices to measure mobility, through gait and physical activity, within five cohorts with chronic conditions (Parkinson's disease [PD], multiple sclerosis [MS], congestive heart failure, [CHF], chronic obstructive pulmonary disorder [COPD], and proximal femoral fracture [PFF]). Results: Thirty-seven studies were identified. Substantial heterogeneity in the quality of reporting, the methods used to assess usability, the devices used, and the aims of the studies precluded any meaningful comparisons. Questionnaires were used in the majority of studies (70.3%; n = 26) with a reliance on intervention specific measures (n = 16; 61.5%). For those who used interviews (n = 17; 45.9%), no topic guides were provided, while methods of analysis were not reported in over a third of studies (n = 6; 35.3%). Conclusion: Usability of wearable devices is a poorly measured and reported variable in chronic health conditions. Although the heterogeneity in how these devices are implemented implies acceptance, the patient voice should not be assumed. In the absence of being able to make specific usability conclusions, the results of this review instead recommends that future research needs to: (1) Conduct usability assessments as standard, irrespective of the cohort under investigation or the type of study undertaken. (2) Adhere to basic reporting standards (e.g. COREQ) including the basic details of the study. Full copies of any questionnaires and interview guides should be supplied through supplemental files. (3) Utilise mixed methods research to gather a more comprehensive understanding of usability than either qualitative or quantitative research alone will provide. (4) Use previously validated questionnaires alongside any intervention specific measures. © 2021, The Author(s).	https://www.scopus.com/inward/record.uri?eid=2-s	Q2	Scopus	EXC_Scope
COPD: Rethinking Patient Management - How to Approach a Ch	2019	In comparison to other chronically ill people, patients suffering from chronic obstructive pulmonary disease (COPD) have many additional difficulties to face and conquer. Due to the contribution of avoidable causes of their illness ("smokers' lung"), society holds people with COPD responsible for their disease, which in return often leads to stigmatization and social isolation. In addition, COPD patients commonly belong to a less privileged social class, own a low socioeconomic status, and lower education. Their physical symptoms are easily observable and - by employing moderate adherence - treatable. Nonetheless, the influence of COPD on a patient's psyche often plays an overly prominent role during therapy. "There is only half a patient laying on the examination table," a revelation that sums up the current state of COPD research and the result of the expert meeting "Luftschlösser" ("castles in the clouds"), which took place in spring 2018. Within the limits of the meeting, participants identified practically applicable approaches aiming to enhance the patient management of this challenging patient group. These considerations are supposed to support healthcare professionals in their daily work and aim to improve the therapy as well as the outcome for COPD patients. © 2019 S. Karger AG, Basel.	https://www.scopus.com/inward/record.uri?eid=2-s	Q2	Scopus	EXC_Scope
Telemedicine in chronic obstructive pulmonary disease: Clinical,	2017	Background: Chronic obstructive pulmonary disease (COPD) is a complex disease that requires multiple care providers working together closely. Recent advances in telemedicine technologies have generated enormous potential benefits for COPD management and care. This review aims to explore the clinical, economic, organizational and patient-quality-of-life impact from using telemedicine systems for COPD and to provide a comprehensive description of these methodologies. Methods: We conducted a structured search of bibliographic databases for peer-reviewed research literature using a focused review question. We include quantitative and qualitative studies. Data extraction and quality assessment of the reviewed studies were done with standardized forms and checklists. Results: The support of evidence from studies points to beneficial results of telemedicine in its various manifestations. Benefits include the reduction of emergency department attendances and hospitalizations, but little evidence has been found to make a strong case for telemedicine regarding the quality of life or economic improvements. Internal organizational consequences of telemedicine are frequent and efficient use of the technology requires organizational changes. Telemedicine modifies traditional practices and can help patients better engage in managing their health. Nevertheless, telemedicine must be integrated into a care process and within a care model. Conclusion: COPD telemedicine interventions could significantly reduce the risk of emergency department attendance and hospitalization. It is an important area of research, and further studies of the effect of telemedicine for patients with COPD would be beneficial. © 2017 Bentham Science Publishers.	https://www.scopus.com/inward/record.uri?eid=2-s	Q2	Scopus	EXC_Scope

Advances in Psychotherapy for Depressed Older Adults	2017	Purpose of Review: We review recent advances in psychotherapies for depressed older adults, in particular those developed for special populations characterized by chronic medical illness, acute medical illness, cognitive impairment, and suicide risk factors. We review adaptations for psychotherapy to overcome barriers to its accessibility in non-specialty settings such as primary care, homebound or hard-to-reach older adults, and social service settings. Recent Findings: Recent evidence supports the effectiveness of psychotherapies that target late-life depression in the context of specific comorbid conditions including COPD, heart failure, Parkinson's disease, stroke and other acute conditions, cognitive impairment, and suicide risk. Growing evidence supports the feasibility, acceptability, and effectiveness of psychotherapy modified for a variety of health care and social service settings. Summary: Research supports the benefits of selecting the type of psychotherapy based on a comprehensive assessment of the older adult's psychiatric, medical, functional, and cognitive status, and tailoring psychotherapy to the settings in which older depressed adults are most likely to present. © 2017, Springer Science+Business Media, LLC.	https://www.scopus.com/inward/record.uri?eid=2-s	Q2	Scopus	EXC_Scope
Exercise training alone or with the addition of activity counseling	2016	Physical inactivity is associated with poor outcomes in COPD, and as a result, interventions to improve physical activity (PA) are a current research focus. However, many trials have been small and inconclusive. Objective: The aim of this systematic review and meta-analysis was to study the effects of randomized controlled trials (RCTs) targeting PA in COPD. Methods: Databases (Physiotherapy Evidence Database [PEDro], Embase, MEDLINE, CINAHL and the Cochrane Central Register for Controlled Trials) were searched using the following keywords: "COPD", "intervention" and "physical activity" from inception to May 20, 2016; published RCTs that aimed to increase PA in individuals with COPD were included. The PEDro scale was used to rate study quality. Standardized mean differences (effect sizes, ESs) with 95% confidence intervals (CIs) were determined. Effects of included interventions were also measured according to the minimal important difference (MID) in daily steps for COPD (599 daily steps). Results: A total of 37 RCTs with 4,314 participants (mean forced expiratory volume in one second (FEV1) % predicted 50.5 [SD=10.4]) were identified. Interventions including exercise training (ET; n=3 studies, 103 participants) significantly increased PA levels in COPD compared to standard care (ES [95% CI]; 0.84 [0.44–1.25]). The addition of activity counseling to pulmonary rehabilitation (PR; n=4 studies, 140 participants) showed important effects on PA levels compared to PR alone (0.47 [0.02–0.92]), achieving significant increases that exceeded the MID for daily steps in COPD (mean difference [95% CI], 1,452 daily steps [549–2,356]). Reporting of methodological quality was poor in most included RCTs. Conclusion: Interventions that included ET and PA counseling during PR were effective strategies to improve PA in COPD. � 2016 Lahham et al.	https://www.scopus.com/inward/record.uri?eid=2-s	Q2	Scopus	EXC_Scope
Health State Utility Value in Chronic Obstructive Pulmonary Dise	2016	Chronic obstructive pulmonary disease (COPD) has a considerable impact on quality of life and well-being of patients. Health state utility value (HSUV) is a recognized measure for health economic appraisals and is extensively used as an indicator for decision-making studies. This study is a systematic review of literature aimed to estimate mean utility value in COPD using meta-analysis and explore degree of heterogeneity in the utility values across a variety of clinical and study characteristic. The literature review covers studies that used EQ-5D to estimate utility value for patient level research in COPD. Studies that reported utility values elicited by EQ-5D in COPD patients were selected for random-effect meta-analysis addressing inter-study heterogeneity and subgroup analyses. Thirty-two studies were included in the general utility meta-analysis. The estimated general utility value was 0.673 (95% CI 0.653 to 0.693). Meta-analyses of COPD stages utility values showed influence of airway obstruction on utility value. The utility values ranged from 0.820 (95% CI 0.767 to 0.872) for stage I to 0.624 (95% CI 0.571 to 0.677) for stage IV. There was substantial heterogeneity in utility values: I2 = 97.7%. A more accurate measurement of utility values in COPD is needed to refine valid and generalizable scores of HSUV. Given the limited success of the factors studied to reduce heterogeneity, an approach needs to be developed how best to use mean utility values for COPD in health economic evaluation. © 2016 Taylor & Francis Group, LLC.	https://www.scopus.com/inward/record.uri?eid=2-s	Q2	Scopus	EXC_Scope
Incorporating telemedicine into the integrated care of the COPD	2018	This report is a summary of a workshop focusing on using telemedicine to facilitate the integrated care of chronic obstructive pulmonary disease (COPD). Twenty-five invited participants from 8 countries met for one and one-half days in Stresa, Italy on 7–8 September 2017, to discuss this topic. Participants included physiotherapists, nurses, a nurse practitioner, and physicians. While evidence-based data are always at the center of sound inference and recommendations, at this point in time the science behind telemedicine in COPD remains under-developed; therefore, this document reflects expert opinion and consensus. While telemedicine has great potential to expand and improve the care of our COPD patients, its application is still in its infancy. While studies have demonstrated its effectiveness in some patient-centered outcomes, the results are by no means consistently positive. Whereas this tool may potentially reduce health care costs by moving some medical interventions from centralized locations in to patient's home, its cost-effectiveness has had mixed results and telemonitoring has yet to prove its worth in the COPD population. These discordant results should not be unexpected in view of patient complexity and the heterogeneity of telemedicine. This is reflected in the very limited support offered by the National Health Services to a wider application of telemedicine in the integrated care of COPD patients. However, this situation should challenge us to develop the necessary science to clarify the role of telemedicine in the medical management of our patients, providing a better and definitive scientific basis to this approach. © 2018	https://www.scopus.com/inward/record.uri?eid=2-s	Q2	Scopus	EXC_PubType
Continuous remote monitoring of COPD patients—justification ar	2018	Remote patient monitoring should reduce mortality rates, improve care, and reduce costs. We present an overview of the available technologies for the remote monitoring of chronic obstructive pulmonary disease (COPD) patients, together with the most important medical information regarding COPD in a language that is adapted for engineers. Our aim is to bridge the gap between the technical and medical worlds and to facilitate and motivate future research in the field. We also present a justification, motivation, and explanation of how to monitor the most important parameters for COPD patients, together with pointers for the challenges that remain. Additionally, we propose and justify the importance of electrocardiograms (ECGs) and the arterial carbon dioxide partial pressure (PaCO2) as two crucial physiological parameters that have not been used so far to any great extent in the monitoring of COPD patients. We cover four possibilities for the remote monitoring of COPD patients: continuous monitoring during normal daily activities for the prediction and early detection of exacerbations and life-threatening events, monitoring during the home treatment of mild exacerbations, monitoring oxygen therapy applications, and monitoring exercise. We also present and discuss the current approaches to decision support at remote locations and list the normal and pathological values/ranges for all the relevant physiological parameters. The paper concludes with our insights into the future developments and remaining challenges for improvements to continuous remote monitoring systems. © 2018, The Author(s).	https://www.scopus.com/inward/record.uri?eid=2-s	Q2	Scopus	EXC_Scope
What else should we know about experiencing COPD? A narrativ	2016	The present paper is a narrative review focusing on the psychological impact, identification of protective factors, and interventions minimizing the psychological burdens of chronic obstructive pulmonary disease (COPD). The research reviews studies on neurocognitive functions, personality, emotional problems, and health-related quality of life. This is done with regard to resources as well as activities enabling or enhancing a patient's adaptation. PubMed and PsychArticles databases were searched for relevant medical (eg, CODP, emphysema), psychopathology (eg, depression), and psychological (eg, personality) keywords, followed by hand search. After application of the inclusion and exclusion criteria, the search resulted in 82 articles and book chapters. The choice was based on evidence accepted by evidence-based medicine, although at different levels of strength. Psychological experiencing of COPD appears to be very unequally represented with scientific research on emotional problems and functioning decrease significantly outnumbering those addressing resources or effective interventions. As our initial literature search called for an urgent need for further exploration, we have carefully pointed out numerous areas where the knowledge on how to protect or restore psychological well-being among COPD patients should be broadened. © 2016 Rzadkiewicz et al.	https://www.scopus.com/inward/record.uri?eid=2-s	Q2	Scopus	EXC_PubType
Chronic obstructive pulmonary disease: Perspectives for primary	2019	Background: Chronic obstructive pulmonary disease (COPD) has become a major health challenge worldwide due to its increasing incidence and mortality, which have serious repercussions for health-care systems. Methods: We conducted a review of international efforts to control COPD in primary care. Results: The WHO created the Alma-Ata declaration which established for the first time, access to health care as a human right. This precept led to the implementation of numerous programs including practical approach to Lung Health and variants in several countries; schemes designed to centralize medical care; and resources to improve attention of respiratory diseases by adapting approaches to the health-care needs of local populations. Primary respiratory health care should include actions for timely detection, health education, and targeted treatment, but the challenge for all health systems is to ensure that their programs function adequately, for they still show shortcomings in terms of their application. Conclusions: We conclude that offering primary health care based on models that combine opportune diagnoses with suitable treatment can positively influence the course of COPD by treating early stages, thus slowing its progression. However, more extensive education and broader dissemination of information are necessary to achieve this goal. © 2019 current edition National Institutes of Health of Mexico.	https://www.scopus.com/inward/record.uri?eid=2-s	Q2	Scopus	EXC_Scope

Oxygen saturation measurements in telemonitoring of patients w	2018	Introduction: Telemonitoring applications are expected to become a key component in future healthcare. Despite the frequent use of SpO2 measurements in telemonitoring of patients with chronic obstructive pulmonary disease (COPD), no profound overview is available about these measurements. Areas covered: A systematic search identified 71 articles that performed SpO2 measurements in COPD telemonitoring. The results indicate that long-term follow-up of COPD patients using daily SpO2 spot checks is practically feasible. Very few studies specified protocols for performing these measurements. In many studies, deviating SpO2 values were used to raise alerts that led to immediate action from healthcare professionals. However, little information was available about the exact implementation and performance of these alerts. Therefore, no firm conclusions can be drawn about the real value of SpO2 measurements. Future research could optimize performance of alerts using individualized, time-dependent thresholds or predictive algorithms to account for individual differences and SpO2 baseline changes. Additionally, the value of performing continuous measurements should be examined. Expert commentary: Standardization of the measurements, data science techniques and advancing technology can still boost performance of telemonitoring applications. All these opportunities should be thoroughly explored to assess the real value of SpO2 in COPD telemonitoring. © 2017 Informa UK Limited, trading as Taylor & Francis Group.	https://www.scopus.com/inward/record.uri?eid=2-s	Q2	Scopus	EXC_Scope
mHealth application areas and technology combinations: A comp	2017	Background: With the continuous and enormous spread of mobile technologies, mHealth has evolved as a new subfield of eHealth. While eHealth is broadly focused on information and communication technologies, mHealth seeks to explore more into mobile devices and wireless communication. Since mobile phone penetration has exceeded other infrastructure in low and middle-income countries (LMICs), mHealth is seen as a promising component to provide pervasive and patient-centered care. Objectives: The aim of our research work for this paper is to examine the mHealth literature to identify application areas, target diseases, and mHealth service and technology types that are most appropriate for LMICs. Methods: Based on the 2011 WHO mHealth report, a combination of search terms, all including the word "mHealth", was identified. A literature review was conducted by searching the PubMed and IEEE Xplore databases. Articles were included if they were published in English, covered an mHealth solution/ intervention, involved the use of a mobile communication device, and included a pilot evaluation study. Articles were excluded if they did not provide sufficient detail on the solution covered or did not focus on clinical ef-ficacy/effectiveness. Cross-referencing was also performed on included articles. Results: 842 articles were retrieved and analyzed, 255 of which met the inclusion criteria. North America had the highest number of applications (n=74) followed by Europe (n=50), Asia (n=44), Africa (n=25), and Australia (n=9). The Middle East (n=5) and South America (n=3) had the least number of studies. The majority of solutions addressed diabetes (n=51), obesity (n=25), CVDs (n=24), HIV (n=18), mental health (n=16), health behaviors (n=16), and maternal and child's health (MCH) (n=11). Fewer solutions addressed asthma (n=7), cancer (n=5), family health planning (n=5), TB (n=3), malaria (n=2), chronic obtrusive pulmonary disease (COPD) (n=2), vision care (n=2), and dermatology (n=2). Other solutions targeted stroke, dental health, hepatitis vaccination, cold and flu, ED prescribed antibiotics, iodine deficiency, and liver transplantation (n=1 each). The remainder of solutions (n=14) did not focus on a certain disease. Most applications fell in the areas of health monitoring and surveillance (n=93) and health promotion and raising awareness (n=88). Fewer solutions addressed the areas of communication and reporting (n=11), data collection (n=6), telemedicine (n=5), emergency medical care (n=3), point of care support (n=2), and decision support (n=2). The majority of solutions used SMS messaging (n=94) or mobile apps (n=71). Fewer used IVR/phone calls (n=8), mobile website/email (n=5), videoconferenc-ing (n=2), MMS (n=2), or video (n=1) or voice messages (n=1). Studies were mostly RCTs, with the majority suffering from small sample sizes and short study durations. Problems addressed by solutions included travel distance for reporting, self-management and disease monitoring, and treatment/medica-tion adherence. Conclusions: SMS and app solutions are the most common forms of mHealth applications. SMS solutions are prevalent in both high and LMICs while app solutions are mostly used in high income countries. Common application areas include health promotion and raising awareness using SMS and health monitoring and surveillance using mobile apps. Remaining application areas are rarely addressed. Diabetes is the most commonly targeted medical condition, yet remains deficient in LMICs. © Schattauer 2017.	https://www.scopus.com/inward/record.uri?eid=2-s	Q2	Scopus	EXC_Scope
Interactive monitoring service and COPD: Is it possible to reduce	2015	Chronic obstructive pulmonary disease (COPD) represents one of the main causes of death worldwide. It affects hundreds of millions of people and is likely to spread further in the coming years. Despite the chronic nature of the disease and the proven efficacy of current therapies, treatment nonadherence is unfortunately common and too often related to treatment failure, disease exacerbations, hospitalizations, and high healthcare costs. At present, studies aimed to assess and improve patients' adherence in chronic respiratory diseases-and especially in COPD-are limited, but a review of the few data available makes it clear that there is a need for an innovative approach that leverages health technology to encourage patients to adhere to prescribed chronic treatments. © 2015 Informa Healthcare USA, Inc.	https://www.scopus.com/inward/record.uri?eid=2-s	Q2	Scopus	EXC_PubType
Meeting the challenge of COPD care delivery in the USA: A multi	2016	The burden of chronic obstructive pulmonary disease (COPD) in the USA continues to grow. Although progress has been made in the the development of diagnostics, therapeutics, and care guidelines, whether patients' quality of life is improved will ultimately depend on the actual implementation of care and an individual patient's access to that care. In this Commission, we summarise expert opinion from key stakeholders-patients, caregivers, and medical professionals, as well as representatives from health systems, insurance companies, and industry-to understand barriers to care delivery and propose potential solutions. Health care in the USA is delivered through a patchwork of provider networks, with a wide variation in access to care depending on a patient's insurance, geographical location, and socioeconomic status. Furthermore, Medicare's complicated coverage and reimbursement structure pose unique challenges for patients with chronic respiratory disease who might need access to several types of services. Throughout this Commission, recurring themes include poor guideline implementation among health-care providers and poor patient access to key treatments such as affordable maintenance drugs and pulmonary rehabilitation. Although much attention has recently been focused on the reduction of hospital readmissions for COPD exacerbations, health systems in the USA struggle to meet these goals, and methods to reduce readmissions have not been proven. There are no easy solutions, but engaging patients and innovative thinkers in the development of solutions is crucial. Financial incentives might be important in raising engagement of providers and health systems. Lowering co-pays for maintenance drugs could result in improved adherence and, ultimately, decreased overall health-care spending. Given the substantial geographical diversity, health systems will need to find their own solutions to improve care coordination and integration, until better data for interventions that are universally effective become available. © 2016 Elsevier Ltd.	https://www.scopus.com/inward/record.uri?eid=2-s	Q2	Scopus	EXC_Scope
Exercise telemonitoring and telerehabilitation compared with trad	2016	Background: Despite exercise capacity and quality-of-life benefits, pulmonary rehabilitation (PR) and cardiac rehabilitation (CR) programmes are not easily accessed because of several barriers. A solution may be telerehabilitation (TR), in which patients exercise in their communities while they are monitored via teletechnology. However, the benefits of TR for the purposes of PR and CR have not been systematically reviewed. Objective: To determine whether the benefits of the exercise component of PR and CR using TR are comparable to usual-care (UC) programmes. Methods: A comprehensive literature search was performed of the Medline, Embase, and CINAHL databases up to July 13, 2015. Meta-analyses were performed for peak oxygen consumption, peak workload, exercise test duration, and 6-minute walk test (6MWT) distance using the I2 statistic and forest plots displaying standardized mean difference (SMD). Results: Of 1,431 citations found, 8 CR studies met the inclusion criteria. No differences were found in exercise outcomes between UC and TR groups for CR studies, except in exercise test duration, which slightly favoured UC (SMD 0.268, 95% CI: 0.002, 0.534, $p < 0.05$). Only 1 PR study was included, and it showed similar improvements on the 6MWT between the UC and TR groups. Conclusion: TR for patients with cardiac conditions provided benefits similar to UC with no adverse effects reported. Similar studies of TR for patients with pulmonary conditions need to be conducted. © University of Toronto Press Inc. All rights reserved.	https://www.scopus.com/inward/record.uri?eid=2-s	Q2	Scopus	EXC_Scope
Telemonitoring to manage chronic obstructive pulmonary disease	2019	Background: Chronic obstructive pulmonary disease (COPD) is a leading cause of death throughout the world. Telemedicine has been utilized for many diseases and its prevalence is increasing in the United States. Telemonitoring of patients with COPD has the potential to help patients manage disease and predict exacerbations. Objective: The objective of this review is to evaluate the effectiveness of telemonitoring to manage COPD. Researchers want to determine how telemonitoring has been used to observe COPD and we are hoping this will lead to more research in telemonitoring of this disease. Methods: This review was conducted in accordance with the Assessment for Multiple Systematic Reviews (AMSTAR) and reported in accordance with the Preferred Reporting Items for Systematic Reviews and Meta-Analyses (PRISMA). Authors performed a systematic review of the PubMed and Cumulative Index to Nursing and Allied Health Literature (CINAHL) databases to obtain relevant articles. Articles were then accepted or rejected by group consensus. Each article was read and authors identified barriers and facilitators to effectiveness of telemonitoring of COPD. Results: Results indicate that conflicting information exists for the effectiveness of telemonitoring of patients with COPD. Primarily, 13 out of 29 (45%) articles stated that patient outcomes were improved overall with telemonitoring, while 11 of 29 (38%) indicated no improvement. Authors identified the following facilitators: Reduced need for in-person visits, better disease management, and bolstered patient-provider relationship. Important barriers included low-quality data, increased workload for providers, and cost. Conclusions: The high variability between the articles and the ways they provided telemonitoring services created conflicting results from the literature review. Future research should emphasize standardization of telemonitoring services and predictability of exacerbations. © 2019 JMIR Publications Inc.. All right reserved.	https://www.scopus.com/inward/record.uri?eid=2-s	Q2	Scopus	EXC_Scope

Systematic Review of Platforms Used for Remote Monitoring of V	2019	The research in telemedicine has been evolving for several years, nevertheless, there isn't a clear designation of which hardware and/or software tools should those who intend to research on this topic use. Given this situation, the objective of this study is to review and established the most used electronic cards and microcontrollers in vital signs remote monitoring systems for specific chronic patients. Also, this research intended to identify which are the motivations, challenges and recommendations to serve this study as support for further research in this area. To accomplish the above, four databases: ScienceDirect, PubMed, Science AAAS and IEEE Xplore, as well as the search engine Google Scholar academic referents, were used for developed literature review. Likewise, among 330 abstracts were searched to determine their relevance and affinity regarding the targeted scope. At the end, 35 documents were selected due to their high level of importance. According to the information extracted from such scientific papers, Arduino ONE electronic development platform has been the most used (considering low-end embedded devices) for the creation of this type of systems since 2016. Further into the review, the followed process and conclusions will be detailed and further explored. © 2019 IEEE.	https://www.scopus.com/inward/record.uri?eid=2-s	Q2	Scopus	EXC_Scope
Time for a longer and better life for patients with COPD	2018	[No abstract available]	https://www.scopus.com/inward/record.uri?eid=2-s	Q2	Scopus	EXC_Scope
Advances in rehabilitation for chronic diseases: Improving health	2019	Much of the burden on healthcare systems is related to the management of chronic conditions such as cardiovascular disease and chronic obstructive pulmonary disease. Although conventional outpatient cardiopulmonary rehabilitation programs significantly decrease morbidity and mortality and improve function and health related quality of life for people with chronic diseases, rehabilitation programs are underused. Barriers to enrollment are multifactorial and include failure to recommend and refer patients to these services; poor communication with patients about potential benefits; and patient factors including logistical and financial barriers, comorbidities, and competing demands that make participation in facility based programs difficult. Recent advances in rehabilitation programs that involve remotely delivered technology could help deliver services to more people who might benefit. Problems with intensity, adherence, and safety of home based programs have been investigated in recent clinical trials, and larger dissemination and implementation trials are under way. This review summarizes the evidence for benefit of in-person cardiac and pulmonary rehabilitation programs. It also reviews the literature on newer developments, such as home based remotely mediated exercise programs developed to decrease cost and improve accessibility, high intensity interval training in cardiac rehabilitation, and alternative therapies such as tai chi and yoga for people with chronic obstructive pulmonary disease. © Published by the BMJ Publishing Group Limited. For permission to use (where not already granted under a licence) please go to.	https://www.scopus.com/inward/record.uri?eid=2-s	Q2	Scopus	EXC_Scope
Why don't our patients with chronic obstructive pulmonary diseas	2016	Nonadherence-not taking pharmacologic or nonpharmacologic treatments according to agreed recommendations from a health care provider-is common in patients with chronic obstructive pulmonary disease. Nonadherence in taking maintenance medications, smoking cessation, maintaining regular physical activity and exercise, starting and staying in pulmonary rehabilitation and continuing on with the postrehabilitation exercise/activity prescription, and successfully following self-management directions results in adverse outcomes across multiple areas. These include a faster decline in airway function, higher symptom burden, impaired health status, and increased health care use and mortality risk. Although nonadherence can also occur in health care providers (not following established treatment guidelines), this perspective focuses on patient nonadherence. Factors such as social/economic, health system, therapy-related, patient-related, and conditionrelated factors all impact this problem. To improve patient adherence, we need to consider these factors in the context of people with chronic obstructive pulmonary disease and implement strategies directly targeting underlying issues. Strategies may include customizing and simplifying learning and intervention regimes, identifying barriers to adherence and addressing them, ensuring patient support structures are in place, and improving self-efficacy. Future directions should focus on research and development in educational design; use of technology to assist education; psychological intervention strategies to support learning, motivation, self-efficacy and behavior change; and ways to improve healthcare providers' engagement with patients. Copyright © 2016 by the American Thoracic Society.	https://www.scopus.com/inward/record.uri?eid=2-s	Q2	Scopus	EXC_Scope
How does it work? Factors involved in telemedicine home-interve	2018	Introduction Definitive evidence of the effectiveness and cost-effectiveness of telemedicine home-interventions for the management of chronic diseases is still lacking. This study examines whether and how published reviews consider and discuss the influence on outcomes of different factors, including: setting, target, and intensity of intervention; patient engagement; the perspective of patients, caregivers and health professionals; the organizational model; patient education and support. Included reviews were also assessed in terms of economic and ethical issues. Methods Two search algorithms were developed to scan PubMed for reviews published between 2000 and 2015, about ICT-based interventions for the management of hypertension, diabetes, heart failure, asthma, chronic obstructive pulmonary disease, or for the care of elderly patients. Based on our inclusion criteria, 25 reviews were selected for analysis. Results None of the included reviews covered all the above-mentioned factors. They mostly considered target (44%) and intervention intensity (24%). Setting, ethical issues, patient engagement, and caregiver perspective were the most neglected factors (considered in 0–4% of the reviews). Only 4 reviews (16%) considered at least 4 of the 11 factors, the maximum number of factors considered in a review is 5. Conclusions Factors that may be involved in ICT-based interventions, affecting their effectiveness or cost-effectiveness, are not enough studied in the literature. This research suggests to consider mostly the role of each one, comparing not only disease-related outcomes, but also patients and healthcare organizations outcomes, and patient engagement, in order to understand how interventions work. © 2018 Bertoncello et al. This is an open access article distributed under the terms of the Creative Commons Attribution License, which permits unrestricted use, distribution, and reproduction in any medium, provided the original author and source are credited.	https://www.scopus.com/inward/record.uri?eid=2-s	Q2	Scopus	EXC_PubType
Tele-medicine in respiratory diseases	2017	Information and Communication Technologies applied to health care and advances in sensor and data transmission technology allowed tele-medicine based programs of care also for patients with respiratory diseases. Different sensors, transmission devices and interventions are used in tele-medicine for some indications. Patients suffering from Chronic Obstructive Pulmonary Disease, asthma, neuromuscular diseases, ventilator assisted individuals and those undergoing pulmonary rehabilitation programs may benefit from this approach. The legal problems are still unsolved. Economic advantages for health care systems, though potentially high, are still poorly investigated. Despite the hopes, we need more evidence before this modality can be considered as a real progress in the management of patients with respiratory diseases. On one hand, these technologies can improve the care of patients with difficult access to services, particularly those in rural/remote areas, on the other hand, there is the risk that they will be used only to reduce standard services in health systems of developed countries. © 2017 The Author(s).	https://www.scopus.com/inward/record.uri?eid=2-s	Q2	Scopus	EXC_Scope

Psychological therapies for the treatment of anxiety disorders in (	2017	Background: Chronic obstructive pulmonary disease (COPD) (commonly referred to as chronic bronchitis and emphysema) is a chronic lung condition characterised by the inflammation of airways and irreversible destruction of pulmonary tissue leading to progressively worsening dyspnoea. It is a leading international cause of disability and death in adults. Evidence suggests that there is an increased prevalence of anxiety disorders in people with COPD. The severity of anxiety has been shown to correlate with the severity of COPD, however anxiety can occur with all stages of COPD severity. Coexisting anxiety and COPD contribute to poor health outcomes in terms of exercise tolerance, quality of life and COPD exacerbations. The evidence for treatment of anxiety disorders in this population is limited, with a paucity of evidence to support the efficacy of medication-only treatments. It is therefore important to evaluate psychological therapies for the alleviation of these symptoms in people with COPD. Objectives: To assess the effects of psychological therapies for the treatment of anxiety disorders in people with chronic obstructive pulmonary disease. Search methods: We searched the specialised registers of two Cochrane Review Groups: Cochrane Common Mental Disorders (CCMD) and Cochrane Airways (CAG) (to 14 August 2015). The specialised registers include reports of relevant randomised controlled trials from The Cochrane Library, MEDLINE, Embase, and PsycINFO. We carried out complementary searches on PsycINFO and CENTRAL to ensure no studies had been missed. We applied no date or language restrictions. Selection criteria: We considered all randomised controlled trials (RCTs), cluster-randomised trials and cross-over trials of psychological therapies for people (aged over 40 years) with COPD and coexisting anxiety disorders (as confirmed by recognised diagnostic criteria or a validated measurement scale), where this was compared with either no intervention or education only. We included studies in which the psychological therapy was delivered in combination with another intervention (co-intervention) only if there was a comparison group that received the co-intervention alone. Data collection and analysis: Two review authors independently screened citations to identify studies for inclusion and extracted data into a pilot-tested standardised template. We resolved any conflicts that arose through discussion. We contacted authors of included studies to obtain missing or raw data. We performed meta-analyses using the fixed-effect model and, if we found substantial heterogeneity, we reanalysed the data using the random-effects model. Main results: We identified three prospective RCTs for inclusion in this review (319 participants available to assess the primary outcome of anxiety). The studies included people from the outpatient setting, with the majority of participants being male. All three studies assessed psychological therapy (cognitive behavioural therapy) plus co-intervention versus co-intervention alone. We assessed the quality of evidence contributing to all outcomes as low due to small sample sizes and substantial heterogeneity in the analyses. Two of the three studies had prespecified protocols available for comparison between prespecified methodology and outcomes reported within the final publications. We observed some evidence of improvement in anxiety over 3 to 12 months, as measured by the Beck Anxiety Inventory (range from 0 to 63 points), with psychological therapies performing better than the co-intervention comparator arm (mean difference (MD) -4.41 points, 95% confidence interval (CI) -8.28 to -0.53; P = 0.03). There was however, substantial heterogeneity between the studies (I2 = 62%), which limited the ability to draw reliable conclusions. No adverse events were reported. Authors' conclusions: We found only low-quality evidence for the efficacy of psychological therapies among people with COPD with anxiety. Based on the small number of included studies identified and the low quality of the evidence, it is difficult to draw any meaningful and reliable conclusions. No adverse events or harms of psychotherapy intervention were reported. A limitation of this review is that all three included studies recruited participants with both anxiety and depression, not just anxiety, which may confound the results. We downgraded the quality of evidence in the 'Summary of findings' table primarily due to the small sample size of included trials. Larger RCTs evaluating psychological interventions with a minimum 12-month follow-up period are needed to assess long-term efficacy. © 2017 The Cochrane Collaboration. Published by John Wiley & Sons, Ltd.	https://www.scopus.com/inward/record.uri?eid=2-s	Q2	Scopus	EXC_Scope
Coincidence versus consequence: Opportunities in multi-morbidi	2017	Introduction: Management of co-and multi-morbidity continues to represent a major clinical challenge. There remains a lack of understanding of how multiple pathologies or conditions co-evolve or interact and importantly how treatments should be combined to effectively improve outcomes. This review highlights the challenges presented to the clinical community in managing co-and multi-morbidity. Areas covered: The review focuses on frequently co-existing chronic conditions characterised by prolonged inflammation; these conditions include chronic obstructive pulmonary disease, rheumatoid arthritis, cardiovascular disease and certain forms of cancer. Literature searches were performed to retrieve relevant articles, particularly those published in the last 10–15 years. Within the review, current management strategies and opportunities for improvements are discussed. Problems with current clinical trial designs are described and the need emphasized for novel treatments and diagnostics for exemplar co-and multi-morbidities. We provide an overview of how precision medicine approaches could offer opportunities to treat multiple inflammation-driven disorders in a more comprehensive fashion. Expert commentary: This section emphasizes the need for further research on individual gene-environment interactions, with the view that environmental risk factors could offer a viable route to prevention of multi-morbidities. © 2017 Informa UK Limited, trading as Taylor & Francis Group.	https://www.scopus.com/inward/record.uri?eid=2-s	Q2	Scopus	EXC_Scope
Improving the Efficiency of Respiratory Drug Delivery: A Review (	2017	Asthma and chronic obstructive pulmonary disease (COPD) are heterogeneous airway diseases associated with significant morbidity and mortality. Pharmacological treatment is delivered primarily through the inhalation route using various devices. Optimal disease control is highly dependent upon patient adherence. Both patients with asthma and COPD are prone to exacerbations leading to hospitalization, which can significantly impact quality of life. Poor adherence is a complex and multifactorial problem that does not have one simple solution. However, it is the biggest risk factor for exacerbations and consequently high healthcare utilization. This review discusses the complex and multifactorial obstacles that impact patient adherence as well as the effect on overall treatment outcomes and healthcare utilization. We also critically examined and compared relatively recent improvements in breath-activated pressurized metered dose inhalers, dry powder inhalers, and e-technology in asthma and COPD. Finally, future treatment strategies for better patient compliance such as personalized medicine and the importance of decision-making between patients and physicians were highlighted. © 2017, The Author(s).	https://www.scopus.com/inward/record.uri?eid=2-s	Q2	Scopus	EXC_Scope
Promise of wearable physical activity monitors in oncology practi	2017	Commercially available physical activity monitors provide clinicians an opportunity to obtain oncology patient health measures to an unprecedented degree. These devices can provide objective and quantifiable measures of physical activity, which are not subject to errors or bias of self-reporting or shorter duration of formal testing. Priorworkonso-called quantified-self datawasbasedonolder-generation, research-grade accelerometers, which laid the foundation for consumer-based physical activity monitoring devices to be validated as a feasible and reliable tool in patients with cancer. Physical activity monitors are being used in chronic conditions including chronic obstructive pulmonary disease, congestive heart failure, diabetes mellitus, and obesity. Differing demographics, compounded with higher symptom and treatment burdens in patients with cancer, imply that additional work is needed to understand the unique strengths and weaknesses of physical activity monitors in this population. Oncology programs can systematically implement these tools into their workflows in an adaptable and iterative manner. Translating large amounts of data collected from an individual physical activity monitoring device into clinically relevant information requires sophisticated data compilation and reduction. In this article,wesummarize the characteristics of older-And newer-generation physical activity monitors, review the validation of physical activity monitors with respect to health-related quality-of-life assessments, and describe the current role of these devices for the practicing oncologist. We also highlight the challenges and next steps needed for physical activity monitors to provide relevant information that can change the current state of oncology practice. Copyright © 2017 American Society of Clinical Oncology. All rights reserved.	https://www.scopus.com/inward/record.uri?eid=2-s	Q2	Scopus	EXC_Scope
Acute exacerbation of COPD	2016	The literature of acute exacerbation of chronic obstructive pulmonary disease (COPD) is fast expanding. This review focuses on several aspects of acute exacerbation of COPD (AECOPD) including epidemiology, diagnosis and management. COPD poses a major health and economic burden in the Asia-Pacific region, as it does worldwide. Triggering factors of AECOPD include infectious (bacteria and viruses) and environmental (air pollution and meteorological effect) factors. Disruption in the dynamic balance between the 'pathogens' (viral and bacterial) and the normal bacterial communities that constitute the lung microbiome likely contributes to the risk of exacerbations. The diagnostic approach to AECOPD varies based on the clinical setting and severity of the exacerbation. After history and examination, a number of investigations may be useful, including oximetry, sputum culture, chest X-ray and blood tests for inflammatory markers. Arterial blood gases should be considered in severe exacerbations, to characterize respiratory failure. Depending on the severity, the acute management of AECOPD involves use of bronchodilators, steroids, antibiotics, oxygen and noninvasive ventilation. Hospitalization may be required, for severe exacerbations. Nonpharmacological interventions including disease-specific self-management, pulmonary rehabilitation, early medical follow-up, home visits by respiratory health workers, integrated programmes and telehealth-assisted hospital at home have been studied during hospitalization and shortly after discharge in patients who have had a recent AECOPD. Pharmacological approaches to reducing risk of future exacerbations include long-acting bronchodilators, inhaled steroids, mucolytics, vaccinations and long-term macrolides. Further studies are needed to assess the cost-effectiveness of these interventions in preventing COPD exacerbations. © 2016 Asian Pacific Society of Respirology	https://www.scopus.com/inward/record.uri?eid=2-s	Q2	Scopus	EXC_PubType

Populations and Interventions for Palliative and End-of-Life Care	2016	Importance: Evidence supports palliative care effectiveness. Given workforce constraints and the costs of new services, payers and providers need help to prioritize their investments. They need to know which patients to target, which personnel to hire, and which services best improve outcomes. Objective: To inform how payers and providers should identify patients with "advanced illness" and the specific interventions they should implement, we reviewed the evidence to identify (1) individuals appropriate for palliative care and (2) elements of health service interventions (personnel involved, use of multidisciplinary teams, and settings of care) effective in achieving better outcomes for patients, caregivers, and the healthcare system. Evidence Review: Systematic searches of MEDLINE, EMBASE, PsycINFO, Web of Science, and Cochrane Database of Systematic Reviews databases (1/1/2001-1/8/2015). Results: Randomized controlled trials (124) met inclusion criteria. The majority of studies in cancer (49%, 38 of 77 studies) demonstrated statistically significant patient or caregiver outcomes (e.g., $p < 0.05$), as did those in congestive heart failure (CHF) (62%, 13 of 21), chronic obstructive pulmonary disease (COPD; 58%, 11 of 19), and dementia (60%, 15 of 25). Most prognostic criteria used clinicians' judgment (73%, 22 of 30). Most interventions included a nurse (70%, 69 of 98), and many were nurse-only (39%, 27 of 69). Social workers were well represented, and home-based approaches were common (56%, 70 of 124). Home interventions with visits were more effective than those without (64%, 28 of 44; vs. 46%, 12 of 26). Interventions improved communication and care planning (70%, 12 of 18), psychosocial health (36%, 12 of 33, for depressive symptoms; 41%, 9 of 22, for anxiety), and patient (40%, 8 of 20) and caregiver experiences (63%, 5 of 8). Many interventions reduced hospital use (65%, 11 of 17), but most other economic outcomes, including costs, were poorly characterized. Palliative care teams did not reliably lower healthcare costs (20%, 2 of 10). Conclusions: Palliative care improves cancer, CHF, COPD, and dementia outcomes. Effective models include nurses, social workers, and home-based components, and a focus on communication, psychosocial support, and the patient or caregiver experience. High-quality research on intervention costs and cost outcomes in palliative care is limited. © Copyright 2016, Mary Ann Liebert, Inc. 2016.	https://www.scopus.com/inward/record.uri?eid=2-s	Q2	Scopus	EXC_Scope
Informal caregiving in COPD: A systematic review of instruments	2017	Background Increasing symptoms and activity restriction associated with COPD progression greatly impact on the lives of their informal caregivers, who play a vital role in maintaining their health. An understanding of this impact is important for clinicians to support caregivers and maintain a viable patient environment at home. This systematic review aimed to identify the instruments commonly used to assess informal caregiving in COPD and describe their measurement properties in this population. Methods Searches were conducted in PubMed, Scopus, Web of Science, CINAHL and PsycINFO and in references of key articles, until November 2016 (PROSPERO: CRD42016041401). Instruments used to assess the impact of COPD on caregivers were identified and their properties described. Quality of studies was rated using the COnsensus-based Standards for the selection of health Measurement INstruments (COSMIN) checklist. Quality of the measurement properties of instruments was rated as ‘positive’, ‘negative’ or ‘indeterminate’. Results Patients cared for, had moderate to very severe COPD and the sample of caregivers ranged from 24 to 406. Thirty-five instruments were used in fifty studies to assess caregivers’ psychological status and mood (9 instruments), burden/distress (12 instruments), quality of life (5 instruments) or other (9 instruments). Eighteen studies assessed the measurement properties of 21 instruments, most commonly hypothesis testing (known validity) and internal consistency. Study quality varied from ‘poor’ to ‘fair’ and with many properties rated as ‘indeterminate’. Conclusions Although several instruments have been used to assess the impact of COPD on caregivers, an increased understanding of their properties is needed before their widespread implementation. © 2017 Elsevier Ltd	https://www.scopus.com/inward/record.uri?eid=2-s	Q2	Scopus	EXC_Scope
Identifying components of self-management interventions that im	2016	Objective To quantify diversity in components of self-management interventions and explore which components are associated with improvement in health-related quality of life (HRQoL) in patients with chronic heart failure (CHF), chronic obstructive pulmonary disease (COPD), or type 2 diabetes mellitus (T2DM). Methods Systematic literature search was conducted from January 1985 through June 2013. Included studies were randomised trials in patients with CHF, COPD, or T2DM, comparing self-management interventions with usual care, and reporting data on disease-specific HRQoL. Data were analysed with weighted random effects linear regression models. Results 47 trials were included, representing 10,596 patients. Self-management interventions showed great diversity in mode, content, intensity, and duration. Although self-management interventions overall improved HRQoL at 6 and 12 months, meta-regression showed counterintuitive negative effects of standardised training of interventionists (SMD = −0.16, 95% CI: −0.31 to −0.01) and peer interaction (SMD = −0.23, 95% CI: −0.39 to 0.06) on HRQoL at 6 months. Conclusion Self-management interventions improve HRQoL at 6 and 12 months, but interventions evaluated are highly heterogeneous. No components were identified that favourably affected HRQoL. Standardised training and peer interaction negatively influenced HRQoL, but the underlying mechanism remains unclear. Practice implications Future research should address process evaluations and study response to self-management on the level of individual patients. © 2016 Elsevier Ireland Ltd	https://www.scopus.com/inward/record.uri?eid=2-s	Q2	Scopus	EXC_Scope
Continuity of Care to Prevent Readmissions for Patients with Chr	2017	Readmissions of patients with chronic obstructive pulmonary disease (COPD) to hospitals cast a heavy burden to health care systems. This meta-analysis was aimed to assess the efficacy of continuity of care as interventions, which reduced readmission and mortality rates of such patients. PubMed, Cochrane Library and Embase were searched for articles published before July 2015. A total of 31 reports with randomized controlled trials (RCTs) were finally included in this meta-analysis. The results showed that health education reduced all-cause readmission at 3 months. In addition, health education, comprehensive nursing intervention (CNI) and telemonitoring reduced all-cause readmissions over 6–12 months, and the effect of CNI was best because CNI also reduced COPD-specific readmissions. Home visits also reduced COPD-specific readmissions (the quality more than moderate), but it did not reduce the risk for all-cause readmissions (risk ratios (RRs), 0.92 [95% CI, 0.82–1.04]; moderate quality). There was no statistically significant difference in reducing mortality and quality of life (QOL) among various continued cares. In conclusion, CNI, telemonitoring, health education and home visits should receive more consideration than other interventions by caregivers seeking to implement continued care interventions for patients with COPD. © 2017 Taylor & Francis Group, LLC.	https://www.scopus.com/inward/record.uri?eid=2-s	Q2	Scopus	EXC_Scope
How will telemedicine change clinical practice in chronic obstruct	2018	Within telehealth there are a number of domains relevant to pulmonary care: telemonitoring, teleassistance, telerehabilitation, teleconsultation and second opinion calls. In the last decade, several studies focusing on the effects of various telemanagement programs for patients with chronic obstructive pulmonary disease (COPD) have been published but with contradictory findings. From the literature, the best telemonitoring outcomes come from programs dedicated to aged and very sick patients, frequent exacerbators with multimorbidity and limited community support; programs using third-generation telemonitoring systems providing constant analytical and decisionmaking support (24 h/day, 7 days/week); countries where strong community links are not available; and zones where telemonitoring and rehabilitation can be delivered directly to the patient’s location. In the near future, it is expected that telemedicine will produce changes in work practices, cultural attitudes and organization, which will affect all professional figures involved in the provision of care. The key to optimizing the use of telemonitoring is to correctly identify who the ideal candidates are, at what time they need it, and for how long. The time course of disease progression varies from patient to patient; hence identifying for each patient a ‘correct window’ for initiating telemonitoring could be the correct solution. In conclusion, as clinicians, we need to identify the specific challenges we face in delivering care, and implement flexible systems that can be customized to individual patients’ requirements and adapted to our diverse healthcare contexts. © The Author(s), 2018.	https://www.scopus.com/inward/record.uri?eid=2-s	Q2	Scopus	EXC_Scope
Perspectives from the kidney health initiative on advancing techr	2017	Telehealth and remote monitoring of a patient’s health status has become more commonplace in the last decade and has been applied to conditions such as heart failure, diabetes mellitus, hypertension, and chronic obstructive pulmonary disease. Conversely, uptake of these technologies to help engender and support home RRTs has lagged. Although studies have looked at the role of telehealth in RRT, they are small and single-centered, and both outcome and cost-effectiveness data are needed to inform future decision making. Furthermore, alignment of payer and government (federal and state) regulations with telehealth procedures is needed along with a better understanding of the viewpoints of the various stakeholders in this process (patients, caregivers, clinicians, payers, dialysis organizations, and government regulators). Despite these barriers, telehealth has great potential to increase the acceptance of home dialysis, and improve outcomes and patient satisfaction while potentially decreasing costs. The Kidney Health Initiative convened a multidisciplinary workgroup to examine the current state of telehealth use in home RRTs as well as outline potential benefits and drawbacks, impediments to implementation, and key unanswered questions. © 2017 by the American Society of Nephrology.	https://www.scopus.com/inward/record.uri?eid=2-s	Q2	Scopus	EXC_Scope

Interventions to modify physical activity in patients with COPD: A	2016	The broad range of interventions to increase physical activity (PA) in patients with chronic obstructive pulmonary disease (COPD) has not been systematically assessed. We aimed to perform a systematic review of the interventional studies that have assessed PA as an outcome in patients with COPD. A systematic search in five different databases (Medline, Embase, PsycINFO, CINAHL and Web of Science) was performed in March 2015. Two independent reviewers analysed the studies against the inclusion criteria (COPD defined by spirometry; prospective, randomised/nonrandomised studies, cohort and experimental studies with interventions using PA as an outcome), extracted the data and assessed the quality of evidence. 60 studies were included. Seven intervention groups were identified. PA counselling increased PA levels in COPD, especially when combined with coaching. 13 studies showed positive effects of pulmonary rehabilitation (PR) on PA, while seven studies showed no changes. All three PR programmes >12 weeks in duration increased PA. Overall, the quality of evidence was graded as very low. Interventions focusing specifically on increasing PA, and longer PR programmes, may have greater impacts on PA in COPD. Well-designed clinical trials with objective assessment of PA in COPD patients are needed. Copyright © ERS 2016.	https://www.scopus.com/inward/record.uri?eid=2-s	Q2	Scopus	EXC_Scope
Telehealth for patients with chronic obstructive pulmonary diseas	2018	Introduction Chronic obstructive pulmonary disease (COPD) is a highly prevalent chronic disease characterised by persistent respiratory symptoms. A focus of COPD interventional studies is directed towards prevention of exacerbations leading to hospital readmissions. Telehealth as a method of remote patient monitoring and care delivery may be implemented to reduce hospital readmissions and improve self-management of disease. Prior reviews have not systematically assessed the efficacies of various telehealth functionalities in patients with COPD at different stages of disease severity. We aim to evaluate which COPD telehealth interventions, classified by their functionalities, are most effective in improving patient with COPD management measured by both clinical and resource utilisation outcomes. Methods and analysis We will conduct a systematic review which will include randomised controlled trials comparing the efficacy of telehealth interventions versus standard care in patients with COPD with confirmed disease severity based on forced expiratory volume(%) levels. An electronic search strategy will be used to identify trials published since 2000 in MEDLINE, EMBASE, the Cochrane Central Register of Controlled Trials, CINHAL. Telehealth is described as remote monitoring and delivery of care where patient data/clinical information is routinely or continuously collected and/or processed, presented to the patient and transferred to a clinical care institution for feedback, triage and intervention by a clinical specialist. Two authors will independently screen articles for inclusion, assess risk of bias and extract data. We will merge studies into a meta-analysis if the interventions, technologies, participants and underlying clinical questions are homogeneous enough. We will use a random-effects model, as we expect some heterogeneity between interventions. In cases where a meta-analysis is not possible, we will synthesise findings narratively. We will assess the quality of the evidence for the main outcomes using GRADE. Ethics and Dissemination Research ethics approval is not required. The findings will be disseminated through publication in a peer-reviewed journal. © Author(s) (or their employer(s)) 2018.	https://www.scopus.com/inward/record.uri?eid=2-s	Q2	Scopus	EXC_PubType
Practical Insight to Monitor Home NIV in COPD Patients	2017	Home noninvasive ventilation (NIV) is used in COPD patients with concomitant chronic hypercapnic respiratory failure in order to correct nocturnal hypoventilation and improve sleep quality, quality of life, and survival. Monitoring of home NIV is needed to assess the effectiveness of ventilation and adherence to therapy, resolve potential adverse effects, reinforce patient knowledge, provide maintenance of the equipment, and readjust the ventilator settings according to the changing condition of the patient. Clinical monitoring is very informative. Anamnesis focuses on the improvement of nocturnal hypoventilation symptoms, sleep quality, and side effects of NIV. Side effects are major cause of intolerance. Screening side effects leads to modification of interface, gas humidification, or ventilator settings. Home care providers maintain ventilator and interface and educate patients for correct use. However, patient's education should be supervised by specialized clinicians. Blood gas measurement shows a significant decrease in PaCO2 when NIV is efficient. Analysis of ventilator data is very useful to assess daily use, unintentional leaks, upper airway obstruction, and patient ventilator synchrony. Nocturnal oximetry and capnography are additional monitoring tools to assess the impact of NIV on gas exchanges. In the near future, telemonitoring will reinforce and change the organization of home NIV for COPD patients. © 2017 Taylor & Francis Group, LLC.	https://www.scopus.com/inward/record.uri?eid=2-s	Q2	Scopus	EXC_Scope
Improvement of physical activity in chronic obstructive pulmonary	2018	Physical activity (PA) is defined as bodily movement produced by skeletal muscles with energy expenditure beyond resting levels. PA is closely related to reduced morbidity and mortality in chronic obstructive pulmonary disease (COPD). Self-report questionnaires are often subject to recall bias, correlating poorly with objectively qualified PA, and do not provide an accurate estimate of free-living energy expenditure. PA may be objectively evaluated by newly developed tri-axial accelerometers by quantifying steps or body movements over a period of time. Low-intensity, home-based pulmonary rehabilitation (PR) using pedometer feedback improves PA. Improvement in physiological factors correlates with increased walking time in stable elderly COPD patients. This review focuses on the effects of PR and pharmacological treatment on PA in COPD patients. We selected 32 studies from our literature search evaluating the effects of PR and 11 studies examining the effects of pharmacological treatment on PA. Findings in both categories were inconsistent. Nineteen studies showed a positive effect with PR whereas 13 showed no effect. Eight studies showed a positive effect, while three revealed no effect from pharmacological intervention. As both interventions increase exercise capacity without a consistent effect on PA, counseling with behavioral changes may be necessary to achieve a significant and lasting increase in PA. Changing PA behavior in COPD patients requires an interdisciplinary approach involving specialists in respiratory medicine, rehabilitation, social, and behavioral sciences. Future research in this area is warranted to advance our knowledge in this area, specifically with regard to the interaction of pharmacological and non-pharmacological interventions. © 2018	https://www.scopus.com/inward/record.uri?eid=2-s	Q2	Scopus	EXC_Scope
Home hospitalization for acute decompensated heart failure: Opp	2018	Importance: Heart failure (HF) is the leading cause of hospitalization among patients over the age of 65 in the United States and developed countries, posing a significant economic burden to the health care systems. More than half of the patients with HF will be readmitted to the hospital within 6 months from discharge, leading not only to increased health care related expenses but also functional decline, iatrogenic injuries and in-hospital infections. With the increasing prevalence of HF, there is a substantial need for innovative delivery care models that can provide hospital level of care at a patient's home. Observations: Home hospitalization was originally used to safely manage chronically ill patients with general medical (stroke, chronic obstructive pulmonary disease, deep vein thrombosis, community acquired pneumonia) and surgical conditions and was associated with improved patient satisfaction and improvement in activity of daily living status. This had no clear effect on readmission or cost. When hospital at home care model was applied to HF patients it demonstrated increased time to readmission, reduced index costs and improved health related quality of life, with no significant differences in adverse events. Eligible patients should be selected based on multiple factors taking into consideration applicable limitations and comorbidities. Conclusions and Relevance: Providing in-hospital level care to the patient's house presents a reliable alternative, yielding multiple benefits both for the patient, as well as the health care system. Formulating a well-defined model is necessary before wide implementation. © 2018 by the authors. Licensee MDPI, Basel, Switzerland.	https://www.scopus.com/inward/record.uri?eid=2-s	Q2	Scopus	EXC_Scope
Integrated Care in Chronic Obstructive Pulmonary Disease and F	2018	Individuals with advanced chronic obstructive pulmonary disease (COPD) often have complex medical problems that require more than simple pharmacological therapy to optimize outcomes. Comprehensive care is necessary to meet the substantial burdens, not just from the primary respiratory disease process itself, but also those imposed by its systemic manifestations and comorbidities. These problems are intensified in the peri-exacerbation period, especially for newly discharged patients. Pulmonary rehabilitation, with its interdisciplinary, patient-centered and holistic approach to management, and integrated care, adding coordination or transition of care to the chronic care model, are useful approaches to meeting these complex issues. © 2018, © 2018 Taylor & Francis Group, LLC.	https://www.scopus.com/inward/record.uri?eid=2-s	Q2	Scopus	EXC_Scope
The role of tele-medicine in patients with respiratory diseases	2017	Introduction: Tele-medicine is a clinical application connecting a patient with specialized care consultants by means of electronic platforms, potentially able to improve patients' self-management and allow for the care of patients with limited access to health services. This article summarizes the use of tele-medicine as a tool in managing patients suffering from some pathological respiratory conditions. Areas covered: We searched papers published between 1990 and 2017 dealing with tele-medicine and respiratory diseases, chronic obstructive pulmonary disease, asthma, interstitial lung disease, chronic respiratory failure, neuromuscular diseases, critical illness, home mechanical ventilation, and also legal and economic issues. Controlled trials report different results on feasibility, cost-effectiveness, and safety of tele-medicine. Expert commentary: Progress in tele-medicine widens the horizons in respiratory medicine: this tool may potentially reduce health care costs by moving some medical interventions from centralized locations in to patient's home, also allowing for the delivery of care in countries with limited access to it. Legal, safety, and privacy problems, as well as reimbursement issues, must still be defined and solved. At present time, we still need much more evidence to consider this modality as a real option in the management of these patients. © 2017 Informa UK Limited, trading as Taylor & Francis Group.	https://www.scopus.com/inward/record.uri?eid=2-s	Q2	Scopus	EXC_Scope

Qualitative investigation into a wearable system for chronic obstr	2016	Objectives To ascertain the stakeholders' views and devise recommendations for further stages of the Wearable Sensing and Smart Cloud Computing for Integrated Care to Chronic Obstructive Pulmonary Disease (COPD) Patients with Co-morbidities (WELCOME) system development. This system aims to create a wearable vest to monitor physiological signals for patients concerned incorporating an inhaler adherence monitoring, weight, temperature, blood pressure and glucose metres, and a mobile health application for communication with healthcare professionals (HCPs). Design A study of qualitative data derived from focus groups and semistructured interviews. Setting 4 participating clinical sites in Greece, the UK, Ireland and the Netherlands. Participants Purposive sampling was used to recruit 32 patients with COPD with heart failure, diabetes, anxiety or depression, 27 informal carers and 23 HCPs from 4 European Union (EU) countries for focus groups and interviews. Results Most patients and HCPs described the WELCOME system as € brilliant and creative' and felt it gave a sense of safety. Both users and HCPs agreed that the duration and frequency of vest wear should be individualised as should the mobile application functions. The parameters and frequency of monitoring should be personalised using a multidisciplinary approach. A € traffic light' alert system was proposed by HCPs for abnormal results. Patients were happy to take actions in response. Conclusions WELCOME stakeholders provided valuable views on the development of the system, which should take into account patient's individual comorbidities, circumstances and concerns. This will enable the development of the individualised system in each member state concerned. © Published by the BMJ Publishing Group Limited.	https://www.scopus.com/inward/record.uri?eid=2-s	Q2	Scopus	EXC_PubType
Recovery Following Acute Exacerbations of Chronic Obstructive	2019	Acute exacerbations are associated with disease progression, hospital admission and death in people with chronic obstructive pulmonary disease (COPD). The detrimental outcomes associated with acute exacerbations highlights a need to understand the time course of recovery following acute exacerbation of COPD (AECOPD) so that effective and timely interventions can be provided. The aim of this narrative review was to describe the natural recovery in physiology, symptoms and function following AECOPD. Substantial recovery of lung function and airway inflammation occurs in the first week after onset of an AECOPD, whilst systemic inflammatory markers may take up to two weeks to recover. Symptoms generally improve over the first 14 days, however marked variation is evident between studies and individuals. There are limited data regarding the time course of recovery for functional capacity, quality of life and strength. In a small number of patients (<10%) recovery of lung function and symptoms has not occurred by three months. Features of patients at risk of a prolonged recovery following AECOPD include older age, more severe lung disease, presence of chronic bronchitis, lower body mass index and more chronic dyspnoea. Exacerbation features associated with prolonged recovery are symptoms of the common cold at exacerbation onset, evidence of viral infection, more severe dyspnoea during the exacerbation and persistent systemic inflammation. In clinical practice efforts should be made to recognise prolonged recovery, which puts patients at risk of poor outcomes, and to address the consequences of AECOPD including physical inactivity and skeletal muscle weakness. Whether delivery of specific interventions at distinct time points in the recovery process can enhance recovery remains to be determined. © 2019 Taylor & Francis Group, LLC.	https://www.scopus.com/inward/record.uri?eid=2-s	Q2	Scopus	EXC_Scope
Self-management interventions including action plans for exacerl	2017	Background: Chronic Obstructive Pulmonary Disease (COPD) self-management interventions should be structured but personalised and often multi-component, with goals of motivating, engaging and supporting the patients to positively adapt their behaviour(s) and develop skills to better manage disease. Exacerbation action plans are considered to be a key component of COPD self-management interventions. Studies assessing these interventions show contradictory results. In this Cochrane Review, we compared the effectiveness of COPD self-management interventions that include action plans for acute exacerbations of COPD (AECOPD) with usual care. Objectives: To evaluate the efficacy of COPD-specific self-management interventions that include an action plan for exacerbations of COPD compared with usual care in terms of health-related quality of life, respiratory-related hospital admissions and other health outcomes. Search methods: We searched the Cochrane Airways Group Specialised Register of trials, trials registries, and the reference lists of included studies to May 2016. Selection criteria: We included randomised controlled trials evaluating a self-management intervention for people with COPD published since 1995. To be eligible for inclusion, the self-management intervention included a written action plan for AECOPD and an iterative process between participant and healthcare provider(s) in which feedback was provided. We excluded disease management programmes classified as pulmonary rehabilitation or exercise classes offered in a hospital, at a rehabilitation centre, or in a community-based setting to avoid overlap with pulmonary rehabilitation as much as possible. Data collection and analysis: Two review authors independently assessed trial quality and extracted data. We resolved disagreements by reaching consensus or by involving a third review author. Study authors were contacted to obtain additional information and missing outcome data where possible. When appropriate, study results were pooled using a random-effects modelling meta-analysis. The primary outcomes of the review were health-related quality of life (HRQoL) and number of respiratory-related hospital admissions. Main results: We included 22 studies that involved 3,854 participants with COPD. The studies compared the effectiveness of COPD self-management interventions that included an action plan for AECOPD with usual care. The follow-up time ranged from two to 24 months and the content of the interventions was diverse. Over 12 months, there was a statistically significant beneficial effect of self-management interventions with action plans on HRQoL, as measured by the St. George's Respiratory Questionnaire (SGRQ) total score, where a lower score represents better HRQoL. We found a mean difference from usual care of -2.69 points (95% CI -4.49 to -0.90; 1,582 participants; 10 studies; high-quality evidence). Intervention participants were at a statistically significant lower risk for at least one respiratory-related hospital admission compared with participants who received usual care (OR 0.69, 95% CI 0.51 to 0.94; 3,157 participants; 14 studies; moderate-quality evidence). The number needed to treat to prevent one respiratory-related hospital admission over one year was 12 (95% CI 7 to 69) for participants with high baseline risk and 17 (95% CI 11 to 93) for participants with low baseline risk (based on the seven studies with the highest and lowest baseline risk respectively). There was no statistically significant difference in the probability of at least one all-cause hospital admission in the self-management intervention group compared to the usual care group (OR 0.74, 95% CI 0.54 to 1.03; 2467 participants; 14 studies; moderate-quality evidence). Furthermore, we observed no statistically significant difference in the number of all-cause hospitalisation days, emergency department visits, General Practitioner visits, and dyspnoea scores as measured by the (modified) Medical Research Council questionnaire for self-management intervention participants compared to usual care participants. There was no statistically significant effect observed from self-management on the number of COPD exacerbations and no difference in all-cause mortality observed (RD 0.0019, 95% CI -0.0225 to 0.0263; 3296 participants; 16 studies; moderate-quality evidence). Exploratory analysis showed a very small, but significantly higher respiratory-related mortality rate in the self-management intervention group compared to the usual care group (RD 0.028, 95% CI 0.0049 to 0.0511; 1219 participants; 7 studies; very low-quality evidence). Subgroup analyses showed significant improvements in HRQoL in self-management interventions with a smoking cessation programme (MD -4.98, 95% CI -7.17 to -2.78) compared to studies without a smoking cessation programme (MD -1.33, 95% CI -2.94 to 0.27, test for subgroup differences: Chi2 = 6.89, df = 1, P = 0.009, I2 = 85.5%). The number of behavioural change techniques clusters integrated in the self-management intervention, the duration of the intervention and adaptation of maintenance medication as part of the action plan did not affect HRQoL. Subgroup analyses did not detect any potential variables to explain differences in respiratory-related hospital admissions among studies. Authors' conclusions: Self-management interventions that include a COPD exacerbation action plan are associated with improvements in HRQoL, as measured with the SGRQ, and lower probability of respiratory-related hospital admissions. No excess all-cause mortality risk was observed, but exploratory analysis showed a small, but significantly higher respiratory-related mortality rate for self-management compared to usual care. For future studies, we would like to urge only using action plans together with self-management interventions that meet the requirements of the most recent COPD self-management intervention definition. To increase transparency, future study authors should provide more detailed information regarding interventions provided. This would help inform further subgroup analyses and increase the ability to provide stronger recommendations regarding effective self-management interventions that include action plans for AECOPD. For safety reasons, COPD self-management action plans should take into account comorbidities when used in the wider population of people with COPD who have comorbidities. Although we were unable to evaluate this strategy in this review, it can be expected to further increase the safety of self-management interventions. We also advise to involve Data and Safety Monitoring Boards for future COPD self-management studies. © 2017 The Cochrane Collaboration.	https://www.scopus.com/inward/record.uri?eid=2-s	Q2	Scopus	EXC_Scope
Moving Pulmonary Rehabilitation into the Home: A CLINICAL RE	2018	Pulmonary rehabilitation (PR) is the standard of care for persons with chronic, symptomatic lung disease. The availability of PR is limited, particularly in rural areas. In addition, barriers to PR include the lack of transportation, patient inconvenience, inadequate insurance coverage, and cost. Technology has the potential to overcome several barriers to PR by enhancing the availability and uptake of PR principles through the development of technology-supported, home-based PR programs. For technology-supported, home-based PR, or telehealth PR, to be effective, key components of traditional PR must be present including appropriate individualized exercise prescription, self-management education, outcome measurements, and patient support. This clinical review summarizes the current practice of PR, describes limitations to the availability of PR, describes key principles that technology should feature to ensure best practices are met, and proposes current and future technology options as an emerging strategy for home delivery of PR and its components. © 2018 Wolters Kluwer Health, Inc. All rights reserved.	https://www.scopus.com/inward/record.uri?eid=2-s	Q2	Scopus	EXC_PubType

The efficacy of telehealth delivered educational approaches for p	2018	Objective: The virtual delivery of patient education and other forms of telehealth have been proposed as alternatives to providing needed care for patients with chronic diseases. The purpose of this systematic review was to compare the efficacy of virtual education delivery on patient outcomes compared with usual care. Methods: The review examined citations from 3 databases, MEDLINE, CINAHL, and EMBASE using the search words telehealth, chronic disease, patient education, and related concepts. From 2447 records published between 2006 and 2017, 16 high to moderate quality studies were selected for review. Eligible papers compared virtual education to usual care using designs allowing for assessment of causality. Results: Telehealth modalities included the web, telephone, videoconference, and television delivered to patients with diabetes, chronic obstructive pulmonary disease, irritable bowel syndrome and heart failure. In 11 of 16 studies, virtually delivered interventions significantly improved outcomes compared to control conditions. In the remaining 5 studies, virtual education showed comparable outcomes to the control conditions. Conclusions: Findings demonstrated that virtual education delivered to patients with chronic diseases was comparable, or more effective, than usual care. Research implications: Despite its benefits, there is potential for further research into the individual components which improve effectiveness of virtually delivered interventions © 2018 Elsevier B.V.	https://www.scopus.com/inward/record.uri?eid=2-s	Q2	Scopus	EXC_Scope
Airway diseases and health literacy (HL) measurement tools: A s	2018	Objective: To identify and evaluate asthma/COPD measurement tools that assess any of the five health literacy (HL) domains: (1) access, (2) understand, (3) evaluate, (4) communicate, and (5) use, as well as numeracy. Methods: MEDLINE/Embase (via Ovid) databases from 1974 to 2016 were searched and complimented by grey literature. Study selection and data extraction were conducted by two reviewers independently. Results: We identified 65 tools including 40 asthma, 22 COPD, and 3 asthma/COPD focused tools. Thirty tools had been validated and two assessed all five domains. The 'understand' domain was captured in 49 tools, followed by 'access' in 29 tools, 'use' in 24 tools, 'evaluate' in 20 tools, and 'communicate' in 10 tools. Two tools assessed 'numeracy'. Tool content comprised disease physiology, triggers, symptoms, inhaler technique, self-management practices, and rehab programs. Conclusions: This review highlights paucity of HL tools that have been validated and/or assess the 'communicate' domain and makes a valuable contribution to filling an existing research gap in the field of HL by determining the deficiencies of such tools. Practice implications: Our review uncovers which HL domains are under-measured, justifying the need to develop an airways HL measurement tool which applies the 5-domain model for asthma/COPD management. © 2017 Elsevier B.V.	https://www.scopus.com/inward/record.uri?eid=2-s	Q2	Scopus	EXC_Scope
Global strategy for the diagnosis, management, and prevention c	2017	This Executive Summary of the Global Strategy for the Diagnosis, Management, and Prevention ofCOPD, Global Initiative for Chronic Obstructive Lung Disease (GOLD) 2017 report focuses primarily on the revised and novel parts of the document. The most significant changes include: (1) the assessment of chronic obstructive pulmonary disease has been refined to separate the spirometric assessment from symptom evaluation. ABCD groups are now proposed to be derived exclusively from patient symptoms and their history of exacerbations; (2) for each of the groups A to D, escalation strategies for pharmacologic treatments are proposed; (3) the concept of deescalation of therapy is introduced in the treatment assessment scheme; (4) nonpharmacologic therapies are comprehensively presented; and (5) the importance of comorbid conditions in managing chronic obstructive pulmonary disease is reviewed. Copyright © 2017 by the American Thoracic Society.	https://www.scopus.com/inward/record.uri?eid=2-s	Q2	Scopus	EXC_PubType
Is there room for further innovation in inhaled therapy for airways	2018	Inhaled medication is the cornerstone in the treatment of patients across a spectrum of respiratory diseases including asthma and chronic obstructive pulmonary disease. The benefits of inhaled therapy have long been recognised but the most important innovations have occurred over the past 60 years, beginning with the invention of the pressurised metered dose inhaler. However, despite over 230 different device and drug combinations currently being available, disease control is far from perfect. Here we look at how innovation in inhaler design may improve treatments for respiratory diseases and how new formulations may lead to treatments for diseases beyond the lungs. We look at the three main areas where innovation in inhaled therapy is most likely to occur: 1) device engineering and design; 2) chemistry and formulations; and 3) digital technology associated with inhalers. Inhaler design has improved significantly but considerable challenges still remain in order to continually innovate and improve targeted drug delivery to the lungs. Healthcare professionals want see innovations that motivate their patients to achieve their goal of improving their health, through better adherence to treatment. Patients want devices that are easy to use and to see that their efforts are rewarded by improvements in their condition. © ERS 2018.	https://www.scopus.com/inward/record.uri?eid=2-s	Q2	Scopus	EXC_PubType

Automated telephone communication systems for preventive hea	2016	Background: Automated telephone communication systems (ATCS) can deliver voice messages and collect health-related information from patients using either their telephone's touch-tone keypad or voice recognition software. ATCS can supplement or replace telephone contact between health professionals and patients. There are four different types of ATCS: unidirectional (one-way, non-interactive voice communication), interactive voice response (IVR) systems, ATCS with additional functions such as access to an expert to request advice (ATCS Plus) and multimodal ATCS, where the calls are delivered as part of a multicomponent intervention. Objectives: To assess the effects of ATCS for preventing disease and managing long-term conditions on behavioural change, clinical, process, cognitive, patient-centred and adverse outcomes. Search methods: We searched 10 electronic databases (the Cochrane Central Register of Controlled Trials; MEDLINE; Embase; PsycINFO; CINAHL; Global Health; WHOLIS; LILACS; Web of Science; and ASSIA); three grey literature sources (Dissertation Abstracts, Index to Theses, Australasian Digital Theses); and two trial registries (www.controlled-trials.com; www.clinicaltrials.gov) for papers published between 1980 and June 2015. Selection criteria: Randomised, cluster- and quasi-randomised trials, interrupted time series and controlled before-and-after studies comparing ATCS interventions, with any control or another ATCS type were eligible for inclusion. Studies in all settings, for all consumers/carers, in any preventive healthcare or long term condition management role were eligible. Data collection and analysis: We used standard Cochrane methods to select and extract data and to appraise eligible studies. Main results: We included 132 trials (N = 4,669,689). Studies spanned across several clinical areas, assessing many comparisons based on evaluation of different ATCS types and variable comparison groups. Forty-one studies evaluated ATCS for delivering preventive healthcare, 84 for managing long-term conditions, and seven studies for appointment reminders. We downgraded our certainty in the evidence primarily because of the risk of bias for many outcomes. We judged the risk of bias arising from allocation processes to be low for just over half the studies and unclear for the remainder. We considered most studies to be at unclear risk of performance or detection bias due to blinding, while only 16% of studies were at low risk. We generally judged the risk of bias due to missing data and selective outcome reporting to be unclear. For preventive healthcare, ATCS (ATCS Plus, IVR, unidirectional) probably increase immunisation uptake in children (risk ratio (RR) 1.25, 95% confidence interval (CI) 1.18 to 1.32; 5 studies, N = 10,454; moderate certainty) and to a lesser extent in adolescents (RR 1.06, 95% CI 1.02 to 1.11; 2 studies, N = 5725; moderate certainty). The effects of ATCS in adults are unclear (RR 2.18, 95% CI 0.53 to 9.02; 2 studies, N = 1743; very low certainty). For screening, multimodal ATCS increase uptake of screening for breast cancer (RR 2.17, 95% CI 1.55 to 3.04; 2 studies, N = 462; high certainty) and colorectal cancer (CRC) (RR 2.19, 95% CI 1.88 to 2.55; 3 studies, N = 1013; high certainty) versus usual care. It may also increase osteoporosis screening. ATCS Plus interventions probably slightly increase cervical cancer screening (moderate certainty), but effects on osteoporosis screening are uncertain. IVR systems probably increase CRC screening at 6 months (RR 1.36, 95% CI 1.25 to 1.48; 2 studies, N = 16,915; moderate certainty) but not at 9 to 12 months, with probably little or no effect of IVR (RR 1.05, 95% CI 0.99, 1.11; 2 studies, 2599 participants; moderate certainty) or unidirectional ATCS on breast cancer screening. Appointment reminders delivered through IVR or unidirectional ATCS may improve attendance rates compared with no calls (low certainty). For long-term management, medication or laboratory test adherence provided the most general evidence across conditions (25 studies, data not combined). Multimodal ATCS versus usual care showed conflicting effects (positive and uncertain) on medication adherence. ATCS Plus probably slightly (versus control; moderate certainty) or probably (versus usual care; moderate certainty) improves medication adherence but may have little effect on adherence to tests (versus control). IVR probably slightly improves medication adherence versus control (moderate certainty). Compared with usual care, IVR probably improves test adherence and slightly increases medication adherence up to six months but has little or no effect at longer time points (moderate certainty). Unidirectional ATCS, compared with control, may have little effect or slightly improve medication adherence (low certainty). The evidence suggested little or no consistent effect of any ATCS type on clinical outcomes (blood pressure control, blood lipids, asthma control, therapeutic coverage) related to adherence, but only a small number of studies contributed clinical outcome data. The above results focus on areas with the most general findings across conditions. In condition-specific areas, the effects of ATCS varied, including by the type of ATCS intervention in use. Multimodal ATCS probably decrease both cancer pain and chronic pain as well as depression (moderate certainty), but other ATCS types were less effective. Depending on the type of intervention, ATCS may have small effects on outcomes for physical activity, weight management, alcohol consumption, and diabetes mellitus. ATCS have little or no effect on outcomes related to heart failure, hypertension, mental health or smoking cessation, and there is insufficient evidence to determine their effects for preventing alcohol/substance misuse or managing illicit drug addiction, asthma, chronic obstructive pulmonary disease, HIV/AIDS, hypercholesterolaemia, obstructive sleep apnoea, spinal cord dysfunction or psychological stress in carers. Only four trials (3%) reported adverse events, and it was unclear whether these were related to the interventions. Authors' conclusions: ATCS interventions can change patients' health behaviours, improve clinical outcomes and increase healthcare uptake with positive effects in several important areas including immunisation, screening, appointment attendance, and adherence to medications or tests. The decision to integrate ATCS interventions in routine healthcare delivery should reflect variations in the certainty of the evidence available and the size of effects across different conditions, together with the varied nature of ATCS interventions assessed. Future research should investigate both the content of ATCS interventions and the mode of delivery; users' experiences, particularly with regard to acceptability; and clarify which ATCS types are most effective and cost-effective. © 2016 The Cochrane Collaboration. Published by John Wiley & Sons, Ltd.	https://www.scopus.com/inward/record.uri?eid=2-s	Q2	Scopus	EXC_Scope
Year in review 2016: Chronic obstructive pulmonary disease and	2017	[No abstract available]	https://www.scopus.com/inward/record.uri?eid=2-s	Q2	Scopus	EXC_PubType
Personalised medicine in asthma: From curative to preventive m	2017	The concept of asthma has changed substantially in recent years. Asthma is now recognised as a heterogeneous entity that is complex to treat. The subdivision of asthma, provided by "cluster" analyses, has revealed various groups of asthma patients who share phenotypic features. These phenotypes underlie the need for personalised asthma therapy because, in contrast to the previous approach, treatment must be tailored to the individual patient. Determination of the patient's asthma phenotype is therefore essential but sometimes challenging, particularly in elderly patients with a multitude of comorbidities and a complex exposure history. This review first describes the various asthma phenotypes, some of which were defined empirically and others through cluster analysis, and then discusses personalisation of the patient's diagnosis and therapy, addressing in particular biological therapies and patient education. This personalised approach to curative medicine should make way in the coming years for personalised preventive and predictive medicine, focused on subjects at risk who are not yet ill, with the aim of preventing asthma before it occurs. The concept of personalised preventive medicine may seem a long way off, but is it really?. ©ERS 2017.	https://www.scopus.com/inward/record.uri?eid=2-s	Q2	Scopus	EXC_Scope
Smart homes and home health monitoring technologies for older	2016	Background: Around the world, populations are aging and there is a growing concern about ways that older adults can maintain their health and well-being while living in their homes. Objectives: The aim of this paper was to conduct a systematic literature review to determine: (1) the levels of technology readiness among older adults and, (2) evidence for smart homes and home-based health-monitoring technologies that support aging in place for older adults who have complex needs. Results: We identified and analyzed 48 of 1863 relevant papers. Our analyses found that: (1) technology-readiness level for smart homes and home health monitoring technologies is low; (2) the highest level of evidence is 1b (i.e., one randomized controlled trial with a PEDro score ≥6); smart homes and home health monitoring technologies are used to monitor activities of daily living, cognitive decline and mental health, and heart conditions in older adults with complex needs; (3) there is no evidence that smart homes and home health monitoring technologies help address disability prediction and health-related quality of life, or fall prevention; and (4) there is conflicting evidence that smart homes and home health monitoring technologies help address chronic obstructive pulmonary disease. Conclusions: The level of technology readiness for smart homes and home health monitoring technologies is still low. The highest level of evidence found was in a study that supported home health technologies for use in monitoring activities of daily living, cognitive decline, mental health, and heart conditions in older adults with complex needs. © 2016.	https://www.scopus.com/inward/record.uri?eid=2-s	Q2	Scopus	EXC_Scope
Living with asthma and chronic obstructive airways disease: Usir	2017	Long-term respiratory conditions such as asthma and chronic obstructive pulmonary disease (COPD) are common, and cause high levels of morbidity and mortality. Supporting self-management is advocated for both asthma and increasingly so for COPD, and there is growing interest in the potential role of a range of new technologies, such as smartphone apps, the web or telehealth to facilitate and promote self-management in these conditions. Treatment goals for both asthma and COPD include aiming to control symptoms, maintain activities, achieve the best possible quality of life and minimize risks of exacerbation. To do this, health professionals should be (a) helping patients to recognize deteriorating symptoms and act appropriately; (b) promoting adherence to maintenance therapy; (c) promoting a regular review where triggers can be established, and strategies for managing such triggers discussed; and (d) promoting healthy lifestyles and positive self-management of symptoms. In particular, low uptake of asthma action plans is a modifiable contributor to morbidity and possibly also to mortality in those with asthma and should be addressed as a priority. Using technology to support self-management is an evolving strategy that shows promise. This review provides an overview of self-management support and discusses how newer technologies may help patients and health professionals to meet key treatment goals. © 2016, © The Author(s) 2016.	https://www.scopus.com/inward/record.uri?eid=2-s	Q2	Scopus	EXC_PubType

When is dual bronchodilation indicated in COPD?	2017	Inhaled bronchodilator medications are central to the management of COPD and are frequently given on a regular basis to prevent or reduce symptoms. While short-acting bronchodilators are a treatment option for people with relatively few COPD symptoms and at low risk of exacerbations, for the majority of patients with significant breathlessness at the time of diagnosis, long-acting bronchodilators may be required. Dual bronchodilation with a long-acting β2-agonist and long-acting muscarinic antagonist may be more effective treatment for some of these patients, with the aim of improving symptoms. This combination may also reduce the rate of exacerbations compared with a bronchodilator-inhaled corticosteroid combination in those with a history of exacerbations. However, there is currently a lack of guidance on clinical indicators suggesting which patients should step up from mono- to dual bronchodilation. In this article, we discuss a number of clinical indicators that could prompt a patient and physician to consider treatment escalation, while being mindful of the need to avoid unnecessary polypharmacy. These indicators include insufficient symptomatic response, a sustained increased requirement for rescue medication, suboptimal 24-hour symptom control, deteriorating symptoms, the occurrence of exacerbations, COPD-related hospitalization, and reductions in lung function. Future research is required to provide a better understanding of the optimal timing and benefits of treatment escalation and to identify the appropriate tools to inform this decision. © 2017 Thomas et al.	https://www.scopus.com/inward/record.uri?eid=2-s	Q2	Scopus	EXC_Scope
The role of physical activity in the context of pulmonary rehabilita	2018	Pulmonary rehabilitation is an important treatment for patients with chronic obstructive pulmonary disease (COPD). Although this intervention leads to large and clinically meaningful improvements in exercise capacity and quality of life, the effect of pulmonary rehabilitation on physical activity is controversial. Physical activity is lower in patients with COPD as compared to healthy age-matched controls and it is related to important health outcomes (e.g. increased risk of mortality and hospitalization). It is an important goal for rehabilitation programs to enhance physical activity to more normal levels in order to achieve the ultimate goal of rehabilitation 'to improve adherence to health enhancing behaviors'. This review discusses the role of physical activity in the context of pulmonary rehabilitation and possible ways to embed interventions geared to behavior change (i.e. to enhance physical activity) and exercise training (i.e. to enhance physical fitness) into comprehensive rehabilitation programs for patients with COPD. © 2019, © 2019 Taylor & Francis Group, LLC.	https://www.scopus.com/inward/record.uri?eid=2-s	Q2	Scopus	EXC_PubType
Making sense of telemedicine in the management of COPD	2018	[No abstract available]	https://www.scopus.com/inward/record.uri?eid=2-s	Q2	Scopus	EXC_PubType
New and emerging technologies for the diagnosis and monitoring	2016	There is a need for straightforward, novel diagnostic and monitoring technologies to enable the early diagnosis of COPD and its differentiation from other respiratory diseases, to establish the cause of acute exacerbations and to monitor disease progression. We sought to establish whether technologies already in development could potentially address these needs. A systematic horizon scanning review was undertaken to identify technologies in development from a wide range of commercial and non-commercial sources. Technologies were restricted to those likely to be available within 18 months, and then evaluated for degree of innovation, potential for impact, acceptability to users and likelihood of adoption by clinicians and patients with COPD. Eighty technologies were identified, of which 25 were considered particularly promising. Biomarker tests, particularly those using sputum or saliva samples and/or available at the point of care, were positively evaluated, with many offering novel approaches to early diagnosis and to determining the cause for acute exacerbations. Several wrist-worn devices and smartphone-based spirometers offering the facility for self-monitoring and early detection of exacerbations were also considered promising. The most promising identified technologies have the potential to improve COPD care and patient outcomes. Further research and evaluation activities should be focused on these technologies. © The Author(s) 2016.	https://www.scopus.com/inward/record.uri?eid=2-s	Q2	Scopus	EXC_PubType
eHealth for people with COPD in the netherlands: A scoping revi	2019	Background: In the Netherlands, almost 600,000 people had chronic obstructive pulmonary disease (COPD) in 2017. This decreases quality of life for many and each year, COPD leads to approximately 6,800 deaths and about one billion health care expenditures. It is expected that eHealth may improve access to care and reduce costs. However, there is no conclusive scientific evidence available of the added value of eHealth in COPD care. We conducted a scoping review into the use of eHealth in Dutch COPD care. The aim of the research was to provide an overview of all eHealth applications used in Dutch COPD care and to assess these applications on a number of relevant criteria. Methods: In order to make an overview of all eHealth applications aimed at COPD patients in the Netherlands, literature was searched in the electronic databases PubMed and Google Scholar. In addition, Dutch health care websites were searched for applications that have been evaluated for effectiveness and reliability. The identified eHealth applications were assessed according to five relevant quality criteria, eg, whether research has been conducted on the effectiveness. Results: Thirteen health care programs and patient platforms in COPD care have been found that use eHealth. In addition, 13 self-care and informative websites and 15 mobile apps were found that are available to citizens and patients. Five of 13 care programs and patient platforms were found to be effective in improving quality of life or reducing hospital admissions in small pilot studies. The effectiveness of these and the other eHealth applications should be established in larger studies in the future. Discussion: More research into the effectiveness of eHealth applications for COPD patients is needed. We recommend to develop a nationwide open source platform where well-evaluated eHealth applications can be showcased for patients and health care providers to improve COPD care. © 2019 Hallensleben et al.	https://www.scopus.com/inward/record.uri?eid=2-s	Q2	Scopus	EXC_PubType
Non-pharmacological treatments for COPD	2016	Chronic obstructive pulmonary disease (COPD) affects roughly 10% of the global population and is growing in prevalence annually. COPD is characterized by progressive non-reversible narrowing of airways mainly due to cigarette smoking. Therapeutic interventions aimed at altering this progressive disease course can largely be grouped into pharmacological or non-pharmacological therapies. The focus of this paper is on the non-pharmacological aspects of COPD management, reviewing the current literature to provide an evidence-based management approach. Non-pharmacological therapies reviewed in this article include the implementation of comprehensive care models utilizing a coordinated multidisciplinary team, tele-monitoring and patient-centred approach to optimize COPD care and improve compliance. Preventing progression of COPD via smoking cessation remains of paramount importance, and newer therapeutic options including electronic cigarettes show promise in small studies as cessation aids. COPD has systemic manifestations that can be ameliorated with the enrollment in pulmonary rehabilitation programmes, which focus on exercise endurance to improve dyspnoea and quality of life. Advanced therapeutics for COPD includes lung volume reduction surgery for a pre-specified cohort and minimally invasive bronchoscopic valves that in recent reviews show promise. Lastly, patients on maximal COPD therapy with progressive disease can be referred for lung transplantation; however, this often requires a highly selected and motivated patient and care team. Survival rates for lung transplantation are improving; thus, this procedure remains a viable option as more expertise and experience are gained. © 2016 Asian Pacific Society of Respirology	https://www.scopus.com/inward/record.uri?eid=2-s	Q2	Scopus	EXC_PubType
The Nurse Practitioners' Perspective on Inhaler Education in Astl	2018	Asthma and chronic obstructive pulmonary disease (COPD) can be debilitating conditions adversely affecting a person's quality of life. Effective treatments are available, but common errors in the use of inhalers compound the issue of disease control. The beliefs and concerns of a patient can also have an impact on treatment adherence, the consequences of which are diminished disease control and the occurrence of exacerbations. Once a treatment has been prescribed, it is often nurses who manage the patient long-term, and they may even be the main care provider. This puts nurses in a key position to monitor inhaler technique, communicate with the patient to improve adherence, and even suggest alternative treatments if the patient and therapy are incompatible. This review examines the central role that nurses play in disease management and emphasizes how effective inhaler education can make a difference to disease control. Good communication between the nurse and patient is vital if this is to be achieved. Recent updates to asthma and COPD guidelines are reviewed, and key resources available to help manage patients are highlighted. Finally, with regard to inhaler education, we reconsider the nursing keystones of "Know it," "Show it," "Teach it," and "Review it." © 2018 Jane Scullion.	https://www.scopus.com/inward/record.uri?eid=2-s	Q2	Scopus	EXC_Scope

Integrated Telehealth and Telecare for Monitoring Frail Elderly wi	2018	Objective: To investigate the potential of an integrated care system that acquires vital clinical signs and habits data to support independent living for elderly people with chronic disease. Materials and Methods: We developed an IEEE 11073 standards-based telemonitoring platform for monitoring vital signs and activity data of elderly living alone in their home. The platform has important features for monitoring the elderly: unobtrusive, simple, elderly-friendly, plug and play interoperable, and self-integration of sensors. Thirty-six (36) patients in a primary care practice in the United Kingdom (mean [standard deviation] age, 82 [10] years) with congestive heart failure (CHF) or chronic obstructive pulmonary disease (COPD) were provided with clinical sensors to measure the vital signs for their disease (blood pressure [BP] and weight for CHF, and oxygen saturation for COPD) and one passive infrared (PIR) motion sensor and/or a chair/bed sensor were installed in a patient's home to obtain their activity data. The patients were asked to take one measurement each day of their vital signs in the morning before breakfast. All data were automatically transmitted wirelessly to the remote server and displayed on a clinical portal for clinicians to monitor each patient. An alert algorithm detected outliers in the data and indicated alerts on the portal. Patient data have been analyzed retrospectively following hospital admission, emergency room visit or death, to determine whether the data could predict the event. Results: Data of patients who were monitored for a long period and had interventions were analyzed to identify useful parameters and develop algorithms to define alert rules. Twenty of the 36 participants had a clinical referral during the time of monitoring; 16 of them received some type of intervention. The most common reason for intervention was due to low oxygen levels for patients with COPD and high BP levels for CHF. Activity data were found to contain information on the well-being of patients, in particular for those with COPD. During exacerbation the activity level from PIR sensors increased slightly, and there was a decrease in bed occupancy. One subject with CHF who felt unwell spent most of the day in the bedroom. Conclusions: Our results suggest that integrated care monitoring technologies have a potential for providing improved care and can have positive impact on well-being of the elderly by enabling timely intervention. Long-term BP and pulse oximetry data could indicate exacerbation and lead to effective intervention; physical activity data provided important information on the well-being of patients. However, there remains a need for better understanding of long-term variations in vital signs and activity data to establish intervention protocols for improved disease management. © Hulya Gokalp, et al., 2018; Published by Mary Ann Liebert, Inc. 2018.	https://www.scopus.com/inward/record.uri?eid=2-s	Q2	Scopus	EXC_PubType
The impact of self-monitoring in chronic illness on healthcare utili	2015	Background: Self-management interventions have been found to reduce healthcare utilisation in people with long-term conditions, but further work is needed to identify which components of these interventions are most effective. Self-monitoring is one such component and is associated with significant clinical benefits. The aim of this systematic review of reviews is to assess the impact of self-monitoring interventions on healthcare utilisation across a range of chronic illnesses. Methods: An overview of published systematic reviews and meta-analyses. Multiple databases were searched (MEDLINE, CINAHL, PsycINFO, EMBASE, AMED, EBM and HMIC) along with the reference lists of included reviews. A narrative synthesis was performed, accompanied by calculation of the Corrected Cover Area to understand the impact of overlapping primary research papers. Results: A total of 17 systematic reviews and meta-analyses across three chronic conditions, heart failure, hypertension and chronic obstructive pulmonary disease, were included. Self-monitoring was associated with significant reductions in hospitalisation and re-admissions to hospital. Conclusions: Self-monitoring has the potential to reduce the pressure placed on secondary care services, but this may lead to increase in services elsewhere in the system. Further work is needed to determine how these findings affect healthcare costs. © 2015 McBain et al.	https://www.scopus.com/inward/record.uri?eid=2-s	Q2	Scopus	EXC_Scope
Update on COPD exacerbation	2015	The study was approved by the committee on research ethics at the institution in which the research was conducted and any informed consent from human subjects was obtained as required. COPD exacerbations represent a growing healthcare burden. During the last few years, new data have been emerging on the diagnosis, evaluation, management and prevention of COPD exacerbation, some of which may change the management of this condition. We now have more evidence concerning duration of glucocorticoids treatment, beneit of antibiotics and markers that can guide antibiotic therapy, noninvasive ventilation (NIV) indications, the role of mucoactive medications, and measures for prevention of COPD exacerbations like prophylactic antibiotic, chronic non-invasive ventilation, mucolytic agents, PDE-4 inhibitors, pulmonary rehabilitation or patient education. We also have evidence showing that some approaches don't seem to provide signiicant beneits, like statin treatment in the absence of cardiovascular or metabolic indication, or vitamin D supplementation when there's no vitamin D deiciency. Further studies with thorough characterization of exacerbations' and underlying patients' characteristics are needed to develop a more tailored strategy depending on patients' and exacerbations' phenotypes.	https://www.scopus.com/inward/record.uri?eid=2-s	Q2	Scopus	EXC_PubType
Telehealth pulmonary rehabilitation: A review of the literature and	2018	Several different applications of telehealth technologies have been used in the care of respiratory patients, including telemonitoring, teleconsultations, tele-education, and telehealth-pulmonary rehabilitation (PR). Telehealth technology provides an opportunity to assist in the management of chronic respiratory diseases and improve access to PR programs. While there is inconclusive evidence as to the effectiveness of telemonitoring to reduce healthcare utilization and detection of exacerbations, teleconsultations have been shown to be an effective means to assess patients' disease prior to the initiation of PR, and telehealth PR has been shown to be as effective as institution-based PR at improving functional exercise capacity and health-related quality of life. To improve PR access across Canada and ensure a high standard of program quality, a team of clinicians and researchers has developed and begun to implement a national standardized PR program that can be delivered across different settings of practice, including remote satellite sites via telehealth PR. The program has adapted the "Living Well with COPD" self-management program and includes standardized reference guides and resources for patients and practitioners. A progressive and iterative process will evaluate the success of program implementation and outcomes. This initiative will address nationwide accessibility challenges and provide PR content as well as evaluations that are in accordance with clinical standards and established self-management practices. © 2017, © The Author(s) 2017.	https://www.scopus.com/inward/record.uri?eid=2-s	Q2	Scopus	EXC_PubType
Telerehabilitation for chronic obstructive pulmonary disease patie	2018	Pulmonary rehabilitation (PR) is proved to be best supportive management in chronic obstructive pulmonary disease (COPD) individuals. The literature claims the reduction of dyspnea, fatigue, exacerbations, and improved functional capacity and quality of life. Home-based PR is being prescribed widely than hospital-based rehab due to be less cost and ease of caregiver burden, but efficacy is usually questioned. The poor efficacy may be probably due to recurrent exacerbation and poor quality of life even after years of home rehabilitation. Telerehabilitation is an excellent rehab measure where the COPD patients exercise at his home, while expertise from the tertiary care centers monitors the rehab sessions remotely. In India, the tele-PR is at its budding state. This review shall enable the readers with the basics of telerehabilitation in comparison with the other available rehab measures and evidence in the management of COPD. © 2018 Indian Journal of Palliative Care | Published by Wolters Kluwer - Medknow.	https://www.scopus.com/inward/record.uri?eid=2-s	Q2	Scopus	EXC_Scope
Innovations in health information technologies for chronic pulmor	2016	Asthma and chronic obstructive pulmonary disease (COPD) are common chronic obstructive lung disorders in the US that affect over 49 million people. There is no cure for asthma or COPD, but clinical guidelines exist for controlling symptoms that are successful in most patients that adhere to their treatment plan. Health information technologies (HITs) are revolutionizing healthcare by becoming mainstream tools to assist patients in self-monitoring and decision-making, and subsequently, driving a shift toward a care model increasingly centered on personal adoption and use of digital and web-based tools. While the number of chronic pulmonary disease HITs is rapidly increasing, most have not been validated as clinically effective tools for the management of disease. Online communities for asthma and COPD patients are becoming sources of empowerment and support, as well as facilitators of patient-centered research efforts. In addition to empowering patients and facilitating disease self-management, HITs offer promise to aid researchers in identifying chronic pulmonary disease endotypes and personalized treatments based on patient-specific profiles that integrate symptom occurrence and medication usage with environmental and genomic data. © 2016 Himes and Weitzman.	https://www.scopus.com/inward/record.uri?eid=2-s	Q2	Scopus	EXC_PubType
Digital Interventions for Psychological Comorbidities in Chronic D	2021	Chronic diseases represent one of the main causes of death worldwide. The integration of digital solutions in clinical interventions is broadly diffused today; however, evidence on their efficacy in addressing psychological comorbidities of chronic diseases is sparse. This systematic review analyzes and synthesizes the evidence about the efficacy of digital interventions on psychological comorbidities outcomes of specific chronic diseases. According to the Preferred Reporting Items for Systematic Reviews and Meta-Analyses (PRISMA) guidelines, a systematic search of PubMed, PsycInfo, Scopus and Web of Science databases was conducted. Only Randomized Controlled Trials (RCTs) were considered and either depression or anxiety had to be assessed to match the selection criteria. Of the 7636 identified records, 17 matched the inclusion criteria: 9 digital interventions on diabetes, 4 on cardiovascular diseases, 3 on Chronic Obstructive Pulmonary Disease (COPD) and one on stroke. Of the 17 studies reviewed, 14 found digital interventions to be effective. Quantitative synthesis highlighted a moderate and significant overall effect of interventions on depression, while the effect on anxiety was small and non-significant. Design elements making digital interventions effective for psychological comorbidities of chronic diseases were singled out: (a) implementing a communication loop with patients and (b) providing disease-specific digital contents. This focus on ``how'' to design technologies can facilitate the translation of evidence into practice.	http://dx.doi.org/10.3390/jpm11010030	Q2	WoS	EXC_Scope

Effectiveness of telemonitoring versus usual care for chronic obs	2020	Aims The purpose of this research was to investigate the effectiveness of telemonitoring for chronic obstructive pulmonary disease. Methods We searched MEDLINE, EMBASE, the Cochrane Central Register of Controlled Trials and CINAHL up to September 2018. We selected randomised controlled trials comparing telemonitoring and control groups for chronic obstructive pulmonary disease management. Two reviewers independently examined articles based on eligibility, extracted data and evaluated the risk of bias. The Cochrane tool was applied for assessing the risk of bias. The 95% confidence interval was calculated. Results A total of 28 randomised controlled trials were included. Meta-analysis revealed that there were no variables showing a statistically significant difference between telemonitoring and control groups. Chronic obstructive pulmonary disease exacerbation rate (six studies) was not different between two groups (risk ratio 0.67, 95% confidence interval 0.31-1.42). Subgroup analysis showed that telemonitoring reduced exacerbation rates when the intervention continued for longer than six months or pulmonary function was monitored. No differences between groups were noticed for mortality (seven studies, risk ratio 0.89, 95% confidence interval 0.60-1.34). Similarly, no differences between groups were observed in the patient-reported outcomes (St George's Respiratory Questionnaire, Chronic Respiratory Disease Questionnaire-Dyspnea score) and for health service utilization (length of hospital stay, number of hospital admissions, number of emergency room visits). Conclusions Telemonitoring for chronic obstructive pulmonary disease was unlikely to result in statistically significant improvements in health outcomes. However, our novel finding was that at least six months of intervention duration and monitoring of pulmonary function play roles in activating the effects of telemonitoring.	http://dx.doi.org/10.1177/1357633X18811757	Q2	WoS	EXC_Scope
COPD Self-Management for Adults Living in Rural Areas: Systen	2022	Background: Chronic Obstructive Pulmonary Disease (COPD) is more prevalent in rural areas than in non-rural areas due to factors such as limited access to healthcare; however, no known studies have systematically reviewed evidence related to the impact of self-management interventions on rural adults with COPD. Purpose: Systematically review telehealth and non-telehealth self-management interventions delivered to adults with COPD in rural areas. Methods: Six electronic databases were searched to identify eleven eligible articles. Results: Six studies delivered telehealth interventions, while five delivered non-telehealth interventions. Six interventions (54.5%) reported the use of a behavioral theory, and four (36.4%) employed a randomized control trial (RCT) design. Three studies (n = 2 telehealth; n = 1 nontelehealth) reported significant improvements in self-management efficacy, five (n = 3 telehealth; n = 2 non-telehealth) reported improvements in quality of life, four (n = 1 telehealth; n = 3 nontelehealth) demonstrated improved exercise capacity, and four (n = 2 telehealth; n = 2 nontelehealth) reported improved COPD knowledge. Discussion: There is promising evidence that telehealth interventions could be as beneficial as nontelehealth interventions for improving COPD self-management in rural areas. Translation to Health Education Practice: Community-based needs assessments in rural areas may help determine the optimal method of delivery (i.e., telehealth and/or non-telehealth strategies) for local COPD self-management interventions.	http://dx.doi.org/10.1080/19325037.2022.2100525	Q2	WoS	EXC_PubType
Videoconferencing interventions and COPD patient outcomes: A	2024	Introduction Videoconferencing circumvents various physical and financial barriers associated with in-person care. Given this technology's potential benefits and timely nature, we conducted a systematic review to understand how videoconferencing for chronic obstructive pulmonary disease (COPD) follow-up care affects patient-related outcomes. Methods We included primary research evaluating the use of bidirectional videoconferencing for COPD patient follow-up. The outcomes of interest were resource utilization, mortality, lifestyle factors, patient satisfaction, barriers, and feasibility. We searched MEDLINE, EMBASE, EBM Reviews, and CINAHL databases for articles published from January 1, 2010, to August 2, 2021. Relevant information was extracted and presented descriptively and common themes and patterns were identified. The risk of bias for each study was assessed using design-specific validated tools. Results We included 39 studies of 18,194 patients (22 quantitative, 12 qualitative, and 5 mixed methods). The included studies were grouped by type of intervention; 18 studies explored videoconferencing for exercise, 19 explored videoconferencing for clinical assessment/monitoring, and 2 examined videoconferencing for education. Generally, videoconferencing was associated with high levels of patient satisfaction. There were mixed results in terms of its effects on resource utilization and lifestyle-related factors. Additionally, 12 studies were at high risk of bias, indicating that these results should be interpreted with caution. Conclusions The videoconferencing interventions resulted in high levels of patient satisfaction, despite facing technological issues. Overall, more research is needed to better understand the effects of videoconferencing interventions on resource utilization and other patient outcomes, quantifying their advantages over in-person care.	http://dx.doi.org/10.1177/1357633X231158140	Q2	WoS	EXC_Scope
Asessment of the impact of telecoaching and mobile applications	2024	ntroduction: Chronic obstructive pulmonary disease (COPD) is a leading cause of morbidity and mortality worldwide, with significant economic and social burdens. Smoking and lack of physical activity are key modifiable risk factors. Reduced physical activity in daily life has been identified as a significant predictor of mortality in these patients. Therefore, numerous attempts have been made to increase physical activity to prevent associated harms. Telemedicine interventions, such as telecoaching or those based on mHealth, may be useful in promoting physical activity in COPD patients, although their effectiveness largely depends on patient adherence and the technological characteristics of the interventions. Objective: To assess the impact of telecoaching and mHealth interventions on improving physical activity in COPD patients. Method: A literature search was conducted in the Web of Science and PubMed databases, following PRISMA guidelines. A manual search was then performed in Google Scholar. Studies including the use of information and communication technologies (ICT) to increase physical activity in COPD patients were included. Results: Fifteen studies were included, revealing varied results on different telemedicine interventions in COPD patients. Some show that telecoaching and mobile applications can increase physical activity, supporting their utility in disease management. However, others did not observe significant improvements. Although most patients are satisfied with these interventions, it is important to consider individual needs and preferences. Additionally, the combined use of telecoaching and applications may enhance long-term adherence. Conclusions: It is essential to tailor interventions according to individual needs: some require physical training to improve tolerance, while others need motivational strategies to incorporate physical activity into their daily routine. Simplification of applications and consideration of individual factors are key to long-term adherence. Further research with larger samples and longer follow-up periods is recommended to better understand long-term impacts and improve patient participation in self-care and physical activity	http://dx.doi.org/10.3306/AJHS.2024.39.05.158	Q2	WoS	EXC_Language
A sense of belonging: A meta-ethnography of the experience of p	2019	Aim To synthesize the qualitative research in the literature addressing how patients with chronic obstructive pulmonary disease experience care received by telemedicine. Design Meta-ethnography. Data Sources Twelve studies, published from 2013 - 2018, were identified by a search of relevant systematic databases in June 2017, including updated searches performed in June 2018. Review Methods The studies were reviewed and critically appraised independently by three researchers. The review followed the seven steps of meta-ethnography developed by Noblit and Hare, including a line-of-argument synthesis. Results The synthesis revealed three second-order constructs: presence, transparency, and ambivalence. Using a line-of-argument synthesis, a model was developed that showed patients' experience of a sense of belonging when receiving care by telemedicine. Conclusion This meta-ethnography contributes to the existing and contradictory evidence base of telemedicine to chronic obstructive pulmonary disease patients. It addresses and adds renewed understanding of who would benefit from telemedicine and why, by illustrating the interrelationship between the conditions of telemedicine care, the severity of COPD, and the need for connectedness and emphasizes that the need to belong in telemedicine care increases with the progression of illness burden and severity. Impact The present study endorses the view that the patients with a severe illness burden are likely to benefit the most when receiving care by telemedicine. However, the benefits rely on the fact that the telemedicine interventions involve emotional, social, and clinical support, including regular contact with healthcare professionals, to meet the requirements to belong.	http://dx.doi.org/10.1111/jan.14117	Q2	WoS	EXC_PubType
Remote Care Technology: A Systematic Review of Reviews and	2018	Objectives-To identify the technologies that are being used in the remote care of patients with chronic conditions, and their most relevant outcomes. Methods-A systematic review of reviews and meta-analyses. Results-Fifty-one systematic reviews and meta-analyses related to diabetes, congestive heart failure, chronic obstructive pulmonary disease, mental and behavioral diseases, cancer, hypertension, asthma, multiple sclerosis, chronic renal disease, and obesity were retrieved; these studies compared the use of remote care technology with usual care. Conclusion-Remote care technology has positive effects in various health-related outcomes, but further research is required to allow its use in clinical practice.	http://dx.doi.org/10.3390/technologies6010022	Q2	WoS	EXC_Scope

Psychosocial Interventions for Patients with Severe COPD-An U	2019	Background and Objectives: Chronic obstructive pulmonary disease (COPD) is a life limiting condition with a long list of serious psychosocial consequences, aggravating with illness progression. In advanced stages, chronic respiratory failure often develops, which might undermine mental health and reduce activity. The study objective was to review the recent studies concerning psychosocial interventions dedicated to patients with severe COPD. Materials and Methods: The PubMed database was searched for terms, such as `COPD and long-term oxygen therapy, non-invasive ventilation, severe or respiratory failure' and `psychological or psychosocial or mental health and intervention.' Studies were included that described patients with stable, severe COPD and the outcomes of psychosocial interventions. Results and Conclusions: Thirty-four studies were identified and divided into four thematic groups: home medical support, exercise, self-management and mental health. The number of studies that focused on mental health preservation in severe COPD was very limited; i.e., none refer directly to those treated with respiratory failure. Improving patients' self-efficacy gave promising effects to the acceptance of palliative care, pulmonary rehabilitation completion and mental health. Physical activity might be recommended to be included in interventions for mental health enhancement, although little is known about the role of the particular forms of exercise. An increasing beneficial use of new technologies for psychosocial interventions was noted. Psychosocial interventions applied in advanced COPD underline the roles of self-efficacy, telehealth and physical activity in physical and mental health preservation. However, all of the above elements need to be independently tested on more homogenous groups of patients and have the possible modes of their treatment analysed.	http://dx.doi.org/10.3390/medicina55090597	Q2	WoS	EXC_PubType
Utilities for asthma and COPD according to category of severity:	2015	Background: Asthma and chronic obstructive pulmonary disease (COPD) are incurable diseases that impact quality-oflife. Objective: To summarize original research articles that measured or utilized preference-based utilities or disutilities according to disease severity. Methods: Medline and Embase were searched from inception until the end of November 2014. Two reviewers independently searched the literature with differences settled through discussion. Data extracted included utility scores as determined in original research categorized according to disease severity as well as disutilities associated with exacerbations or comorbidities. Data were tabulated and analyzed descriptively. Results: In total, 862 articles were identified, 790 were rejected, and 69 analyzed. There were 44 dealing with COPD and 25 with asthma. Average utilities determined by research were 0.828 +/- 0.062, 0.765 +/- 0.090, 0.711 +/- 0.120, and 0.607 +/- 0.120 for mild, moderate, severe, and very severe COPD, respectively. Utilities used in economic analyses were 0.866 +/- 0.038, 0.770 +/- 0.024, 0.739 +/- 0.045, and 0.596 +/- 0.075, respectively. Disutilities (annual) ranged from 0.002-0.378; major and minor exacerbations had respective disutilities of 0.287 and 0.108. For asthma patients, utilities were for 0.86 +/- 0.32, 0.83 +/- 0.065, and 0.74 +/- 0.029, for mild, moderate, and severe disease, respectively. Conclusions: Utilities have been summarized according to severity category of asthma and COPD. These values should be useful for researchers undertaking economic analyses of these diseases.	http://dx.doi.org/10.3111/13696998.2015.1025793	Q2	WoS	EXC_Scope
Interventions Including Smart Technology Compared With Face-t	2022	Background: This is a systematic review of randomized controlled trials and a meta-analysis comparing smart technology with face-to-face physical activity (PA) interventions in community-dwelling older adults (mean age 60 years). Objective: This study aims to determine the effect of interventions including smart technology components compared with face-to-face PA interventions on PA and physical function in older adults. The secondary outcomes are depression, anxiety, and health-related quality of life. Methods: We searched MEDLINE, Embase, CINAHL, and AMED electronic databases from inception to February 2021. Two independent reviewers screened titles, abstracts, and full texts and performed data extraction and risk of bias assessments using the Cochrane risk of bias tool. The Grading of Recommendations Assessment, Development and Evaluation was used to evaluate the quality of the evidence. We provided a narrative synthesis on all included studies and, where possible, performed meta-analyses for similar outcomes. Results: This review included 19 studies with a total of 3455 participants. Random effects meta-analyses showed that interventions with smart technology components resulted in improved step count (mean difference 1440 steps, 95% CI 500-2390) and total PA (standardized mean difference 0.17, 95% CI 0.02-0.32) compared with face-to-face alone. There was no difference between groups in terms of the measures of physical function. Smart technology alone did not show significant differences between groups in any outcome. The quality of the evidence was very low based on the Grading of Recommendations Assessment, Development and Evaluation criteria. Conclusions: Interventions that include smart technology may improve daily step counts by an average of 1440 steps in community-dwelling older adults; however, the quality of the evidence was very low. Future studies are needed to improve the certainty of these results.	http://dx.doi.org/10.2196/36134	Q2	WoS	EXC_Scope
Nurse-led remote digital support for adults with chronic condition	2025	AimThe systematic review aims to synthesize the literature examining the effectiveness of nurse-led remote digital support on health outcomes in adults with chronic conditions.BackgroundAdults with chronic diseases have increased rates of mortality and morbidity and use health care resources at a higher intensity than those without chronic conditions-placing strain on the patient, their caregivers and health systems. Nurse-led digital health disease self-management interventions have potential to improve outcomes for patients with chronic conditions by facilitating care in environments other that the hospital setting.Design and MethodsWe searched PubMed/MEDLINE, Embase, PsycINFO and Cochrane Central databases from inception to 7 December 2022. We included randomized controlled trials assessing the impact of nurse-led remote digital support interventions compared to usual care on health-related outcomes in adults with chronic illness. The Cochrane risk-of-bias tool was used to assess bias in studies. Outcomes were organized into four categories: self-management, clinical outcomes, health care resource use and satisfaction with care. Results are presented narratively based on statistical significance.ResultsForty-four papers pertaining to 40 unique studies were included. Interventions most targeted diabetes (n = 11) and cardiovascular disease (n = 8). Websites (n = 10) and mobile applications (n = 10) were the most used digital modalities. Nurses supported patients either in response to incoming patient health data (n = 14), virtual appointment (n = 8), virtual health education (n = 5) or through a combination of these approaches (n = 13). Positive impacts of nurse-led digital chronic disease support were identified in each outcome category. Mobile applications were the most effective digital modality.Conclusion and Relevance to Clinical PracticeResults show that nurse-led remote digital support interventions significantly improve self-management capacity, clinical health outcomes, health care resource use and satisfaction with care. Such interventions have potential to support overall health for adults with chronic conditions in their home environments.	http://dx.doi.org/10.1111/jocn.17226	Q2	WoS	EXC_Scope
mHealth Application Areas and Technology Combinations	2017	Background: With the continuous and enormous spread of mobile technologies, mHealth has evolved as a new subfield of eHealth. While eHealth is broadly focused on information and communication technologies, mHealth seeks to explore more into mobile devices and wireless communication. Since mobile phone penetration has exceeded other infrastructure in low and middle-income countries (LMICs), mHealth is seen as a promising component to provide pervasive and patient-centered care. Objectives: The aim of our research work for this paper is to examine the mHealth literature to identify application areas, target diseases, and mHealth service and technology types that are most appropriate for LMICs. Methods: Based on the 2011 WHO mHealth report, a combination of search terms, all including the word ``mHealth'', was identified. A literature review was conducted by searching the PubMed and IEEE Xplore databases. Articles were included if they were published in English, covered an mHealth solution/intervention, involved the use of a mobile communication device, and included a pilot evaluation study. Articles were excluded if they did not provide sufficient detail on the solution covered or did not focus on clinical efficacy/effectiveness. Cross-referencing was also performed on included articles. Results: 842 articles were retrieved and analyzed, 255 of which met the inclusion criteria. North America had the highest number of applications (n=74) followed by Europe (n=50), Asia (n=44), Africa (n=25), and Australia (n=9). The Middle East (n=5) and South America (n=3) had the least number of studies. The majority of solutions addressed diabetes (n=51), obesity (n=25), CVDs (n=24), HIV(n=18), mental health (n=16), health behaviors (n=16), and maternal and child's health (MCH) (n=11). Fewer solutions addressed asthma (n=7), cancer (n=5), family health planning (n=5), TB (n=3), malaria (n=2), chronic obtrusive pulmonary disease (COPD) (n=2), vision care (n=2), and dermatology (n=2). Other solutions targeted stroke, dental health, hepatitis vaccination, cold and flu, ED prescribed antibiotics, iodine deficiency, and liver transplantation (n=1 each). The remainder of solutions (n=14) did not focus on a certain disease. Most applications fell in the areas of health monitoring and surveillance (n=93) and health promotion and raising awareness (n=88). Fewer solutions addressed the areas of communication and reporting (n=11), data collection (n=6), telemedicine (n=5), emergency medical care (n=3), point of care support (n=2), and decision support (n=2). The majority of solutions used SMS messaging (n=94) or mobile apps (n=71). Fewer used IVR/phone calls (n=8), mobile website/email (n=5), videoconferencing (n=2), MMS (n=2), or video (n=1) or voice messages (n=1). Studies were mostly RCTs, with the majority suffering from small sample sizes and short study durations. Problems addressed by solutions included travel distance for reporting, self-management and disease monitoring, and treatment/medica tion adherence. Conclusions: SMS and app solutions are the most common forms of mHealth applications. SMS solutions are prevalent in both high and LMICs while app solutions are mostly used in high income countries. Common application areas include health promotion and raising awareness using SMS and health monitoring and surveillance using mobile apps. Remaining application areas are rarely addressed. Diabetes is the most commonly targeted medical condition, yet remains deficient in LMICs.	http://dx.doi.org/10.3414/ME17-05-0003	Q2	WoS	EXC_Scope
