# Supplementary material for: Artificial Intelligence Methods and Digital Intervention Strategies for Predicting and Managing Chronic Obstructive Pulmonary Disease Exacerbations: An Umbrella Review
Source: Healthcare (Basel). 2025 Nov 24;13(23):3037. doi: 10.3390/healthcare13233037 (PMC12691994; doi:10.3390/healthcare13233037)
Supplement: Supplementary file 1 [file healthcare-13-03037-s001.zip › s4_RQ2_full_table_data_extraction.pdf]

Table S9. Data extraction table for RQ2 studies with study design, PICOs, statistical findings, and main conclusions.

Study	Year	Type of Review	Number of Included Studies	Study Design	P – Population	I – Digital Intervention	C – Comparator	O – Outcomes	Statistical Findings	Heterogeneity Reported	Main Conclusions	Limitations
Effectiveness of Mobile Health Intervention in Medication Adherence: a Systematic Review and Meta-Analysis	2025	Systematic review and meta-analysis	26	RCTs	Patients with chronic diseases require regular medication.<br>Chronic diseases include:<br>> Cardiovascular disease (most common)<br>> Diabetes<br>> COPD (2 studies)<br>> Other: stroke, asthma, AIDS, Chronic kidney disease, cancer<br><br>**Demographics**<br>> Older adults with mean age in the 50s and 60s<br>> Gender: not consistently reported<br>> Regions/Countries: Multinational studies conducted in the U.S., China, Australia, Austria, Brazil, Canada, Denmark, Ghana, India, Indonesia, Palestine, Spain, and Switzerland	Mobile health (mHealth) apps with features like reminders (simple/advanced), education, symptom tracking, data sharing, pill dispensers; durations ranged from 1 to 12 months<br><br>mHealth applications delivered via smartphones. These apps included key components such as:<br>> Reminders<br>> Education for disease-specific information or medication adherence (video training)<br>> Monitoring via self-reports or data collection using Bluetooth-enabled devices<br>> Feedback and alerts<br>> Data sharing<br>> Interaction through chat, dashboards, and healthcare professionals<br><br>**Duration**<br>> Ranged from 1 to 12 months, with most studies running for 3-6 months.	Control group received standard clinical follow-up without mobile apps<br>> In-person clinic visits<br>> Verbal or printed health education from healthcare providers<br>> Standard medication prescription and follow-up<br>> Non-digital adherence strategies (e.g., brochures, phone calls in some cases)<br>> Educational materials (non-digital)	• **Primary outcomes** across all studies were medication adherence<br><br>• **Quality of life measures** were not assessed or reported in the included studies<br><br>• **Medication adherence** assessed with self-report tools and electronic monitoring<br>> Self-report instruments: MMAS-8, Hill-Bone CHBPT , MAQ, Voils Extent Scale, MG Test<br>> Electronic tools: pill count from devices, dispenser logs<br><br>• **Disease management** was not systematically reported, but some apps allowed tracking of vitals and symptoms like blood pressure, heart rate, and fatigue<br><br>• **Direction/magnitude of change**:<br>> OR = 2.37 (95% CI: 1.98–2.84) – higher adherence in intervention groups<br>> SMD = 0.28 (95% CI: 0.10–0.46) – overall effect size<br>> Enhanced effects with: advanced reminders (SMD = 0.455), interactive apps, data sharing, medication dispensers	Meta-analysis showed a significant improvement in medication adherence among intervention groups (OR = 2.371, 95% CI: 1.98–2.84; SMD = 0.279, 95% CI: 0.10–0.46). Subgroup analyses demonstrated larger effects when interventions included interactive features (SMD = 0.283), advanced reminders (SMD = 0.455), data sharing with providers (SMD = 0.346), and the use of medication dispensers (OR = 2.453). Publication bias was detected (Egger's p = 0.001), but the results were considered robust (Fail-safe N = 878).	Assessed using the I^2 and Q statistics.<br>While some pooled outcomes showed low heterogeneity (I^2 = 0%), others showed moderate to high variability (I^2 up to 85%).<br>Substantial heterogeneity was observed in self-reported adherence scores. Subgroup analyses indicated that heterogeneity was reduced in studies using advanced reminders, interactive features, or provider data-sharing. A random-effects model was applied throughout.	**Overall effectiveness**<br>> Mobile health interventions significantly improved medication adherence among patients with chronic diseases compared to standard care<br>> The effects were statistically significant and clinically meaningful<br><br>**Drivers of effectiveness**<br>> Interactive features: chat, dashboards and feedbacks<br>> Advanced reminders: context-aware or personalized alerts<br>> Data sharing with healthcare providers<br>> Use of medication dispensers like smart inhalers and electronic pillboxes<br><br>**Clinical Implications**<br>> Apps with comprehensive, tailored features can address multiple adherence barriers (behavioral, cognitive, environmental).<br>> Stakeholder involvement (e.g., healthcare providers, patients) was highlighted as a critical factor in app effectiveness.<br>> Mobile apps support long-term disease management and offer potential for scalable, personalized care interventions.<br><br>**Future Directions**<br>> Emphasis on integrating advanced technologies (e.g., AI, sensors)<br>> Need for user-centered design considering acceptability and usability<br>> Future interventions should prioritize features that support self-management, real-time feedback, and professional monitoring	**Language and Publication Bias**<br>> Only studies published in English or Korean were included, potentially excluding relevant data in other languages<br>> Grey literature and unpublished studies were not searched, increasing the risk of publication bias<br>> Egger's test confirmed publication bias (p = 0.001), although the results were considered statistically robust<br><br>**Measurement Inconsistencies**<br>There were variations in adherence measurement tools:<br>> Some studies used self-reported questionnaires (e.g., MMAS-8, MAQ), while others used electronic pill counts<br>> This led to difficulty in pooling all 26 studies for a single effect estimate.<br><br>**Limited Outcome Scope**<br>> The review focused solely on medication adherence.<br>> It did not assess quality of life or disease management outcomes, limiting the scope of impact assessment<br><br>**Study Quality Concerns**<br>> Many included RCTs showed methodological weaknesses: lack of blinding of participants and outcome assessors, and inadequate reporting of randomization and allocation concealment<br>> These issues contributed to a high risk of bias in nearly half (12/26) of the studies<br><br>**Heterogeneity**<br>> Some analyses, particularly those involving self-reported adherence scores, showed substantial statistical heterogeneity (e.g., I^2 > 60–70%)<br>> This reduced the certainty in pooled estimates and limited generalizability
Digital interventions for the management of chronic obstructive pulmonary disease	2021	Systematic review and meta-analysis	14	RCTs	> Adults with a clinical diagnosis of COPD<br>> Severity ranged from mild to very severe<br>> Mean age across studies was 65–72 years<br>> Gender distribution: male participants ranged from 48% to 100%, indicating a male-predominant sample in several trials<br>> Country/Regions: USA, UK, Canada, Netherlands, Spain, China, Taiwan, Belgium, Europe, South Korea<br>> Other: most participants were from high-income countries; only studies conducted in East Asia included patients from low and middle-income countries.	Digital intervention categorized into two groups<br><br>**Single-component digital intervention**<br>> Mobile health apps for self monitoring, reminders or education<br>> Web-based platforms for symptom tracking, action planning, or exercise modules<br>> Smartphone/tablet-based coaching or breathing retraining<br>> Skype video sessions for training breathing techniques<br>> Clinical/educational videos for tailored audiovisual content<br><br>**Multi-component digital literventions** that combines digital tools and other components like in-person support or care coordination<br><br>**Duration on the interventions**<br>> Ranged between 13 to 52 weeks<br>> Shorter durations (13–26 weeks) were common for breathing retraining or app-based trials<br>> Longer trials (12 months) were typically multi-component or included ongoing feedback loops	Control group received standard care or minimal non-digital intervention<br>> Usual Care: regular follow-up, medication, emergency visits, and patient-initiated contact with healthcare providers.<br>> Standard Care: includes structured care pathways, but without added digital support and delivered in person<br>> Face-to-Face interventions: digital interventions were compared with traditional face-to-face self-management programs, including in-person education or support<br>> Paper-based Action Plans; control groups were provided with paper-based COPD action plans for exacerbation management or symptom tracking<br>> Access to Educator/Clinic Only: some controls included access to a certified respiratory educator or clinic care, but not the digital component	• **Primary outcomes** across the studies were functional capacity (6MWD), quality of life and self-efficacy.<br>> 6MWD: Mixed and uncertain effects; 52-week result exceeded MID of 25 meters but had high heterogeneity; very low certainty.<br>> Self-efficacy for managing chronic disease: No consistent or significant changes; very low certainty. Assessed with PRAISE, SEMCD<br><br>• **Quality of life** measures were assessed using:<br>> CRQ: Small improvements but below MID of 0.5 points; low to very low certainty. Assessed with CRQ total<br>>SGRQ: Large, clinically meaningful improvement (>4 points MID); low certainty. Assessed with SGRQ total<br>> CAT: Not clinically significant; very low certainty<br>> Other measures (not pooled in meta-analysis): EQ-5D, CCQ used in some studies; results generally mixed or not reported in pooled data<br><br>• **Medication adherence**: Not systematically reported<br><br>• **Disease management metrics**<br>> Dyspnoea (CRQ-dyspnoea): Possibly clinically significant at 52 weeks (MID = 0.5); low certainty. Assessed with CRQ-dyspnoea<br>> Exacerbation frequency: No consistent benefit; low to very low certainty. Assessed with self-reports or medically confirmed exacerbations<br>> Hospital admissions and ED visits: Mixed findings; some reduction in hospital readmissions but not consistent<br>> Patient satisfaction: No strong preference for digital interventions. It shows mixed results; e.g., CSQ-8 MD –3.6 (95% CI –7.32 to 0.12) assessed with CSQ-8, satisfaction ratings<br><br>• **Direction/magnitude of change**:<br>> 6MWD: MD = 54.33 meters (95% CI: –35.47 to 144.12) at 52 weeks – large but uncertain improvement<br>> Self-efficacy (SEMCD): MD = 0.2 (95% CI: –1.03 to 1.43) – minimal change<br>> Quality of life (SGRQ): MD = –26.57 (95% CI: –34.09 to –19.05) – substantial improvement, exceeding MID<br>> Dyspnoea (CRQ-dyspnoea): MD = 0.64 (95% CI: 0.06 to 1.22) – clinically meaningful improvement<br>> Hospitalization (COPD-related): OR = 0.68 (95% CI: 0.46 to 0.99) – lower risk in intervention group<br>> Exacerbations (26 weeks): MD = –0.17 (95% CI: –0.50 to 0.16) – no consistent difference	Meta-analyses showed mixed but occasionally significant effects of digital interventions on clinical and behavioral outcomes in COPD patients. The largest effect was observed in quality of life as measured by SGRQ (MD = –26.57, 95% CI: –34.09 to –19.05), exceeding the clinical threshold (MID = 4 points). Functional capacity (6MWD) showed improvement at 52 weeks (MD = 54.33 meters, 95% CI: –35.47 to 144.12), though results were imprecise and highly heterogeneous (I^2 = 87%).<br><br>Subgroup and single-study results indicated potential benefits in dyspnoea management (CRQ-dyspnoea MD = 0.64, 95% CI: 0.06 to 1.22) and hospital admission reduction (OR = 0.68, 95% CI: 0.46 to 0.99). However, other outcomes like self-efficacy (SEMCD MD = 0.2, 95% CI: –1.03 to 1.43) and exacerbation frequency (MD = –0.17, 95% CI: –0.50 to 0.16) did not reach statistical or clinical significance.<br><br>Adverse event rates were comparable between groups (OR for serious AEs = 0.85, 95% CI: 0.55 to 1.34). Due to heterogeneity and small sample sizes, no consistent subgroup effects were identified, and publication bias was not formally assessed.	Assessed using the I^2 and Q statistics.<br>Heterogeneity was present and quantified for nearly all meta-analyses, with I^2 values ranging from 0% to 87%. It was highest in long-term physical function and QoL outcomes, and attributed to differences in intervention intensity, delivery, and patient characteristics. Subgroup analyses did not identify clear moderators of the treatment effect.<br><br>> 6MWD (52 weeks): I^2 87%<br>> CRQ total (13 weeks): I^2 0%<br>> CRQ total (52 weeks): I^2 68%<br>> Self-efficacy (SEMCD): I^2 0%<br>> SGRQ (52 weeks): Not pooled (single study)<br>> Exacerbations (≥1 event): I^2 0%<br>> Dyspnoea (CRQ-dyspnoea): I^2 0–37%<br>> Hospital admissions: Not pooled consistently	**Overall effectiveness**<br>> Digital health interventions showed modest effectiveness in improving quality of life, physical activity (6MWD), and dyspnoea symptoms in COPD patients when compared to standard care<br>> Effects were statistically significant in some studies (e.g., SGRQ, CRQ-dyspnoea) and clinically meaningful when interventions were longer and behaviourally enriched<br>> Outcomes related to self-efficacy and exacerbation reduction were inconsistent, and medication adherence was not adequately measured<br><br>**Drivers of effectiveness**<br>> Inclusion of behaviour change techniques: goal-setting, self-monitoring, feedback<br>> Multi-component design combining education, remote monitoring, and provider communication<br>> Interventions with durations ≥6 months had more sustained effects<br>> Personalized feedback and integration with healthcare systems supported greater engagement<br><br>**Clinical Implications**<br>> Digital tools may serve as a valuable adjunct to usual care in COPD self-management, especially for enhancing QoL and activity levels<br>> Benefits are most likely when tools are interactive, tailored to individual needs, and connected to clinical workflows<br>> Healthcare provider involvement and coaching improved patient engagement and program adherence<br><br>**Future Directions**<br>> More high-quality RCTs are needed to assess digital tools on exacerbations and medication adherence<br>> Interventions should prioritize usability, accessibility, and long-term engagement strategies<br>> Future designs should isolate active components (e.g., reminders, feedback) and assess their independent contributions<br>> Integration with electronic health records and remote care platforms will be important for scalability	**Risk of bias**<br>> Many included RCTs were rated as unclear or high risk of bias due to: lack of blinding, incomplete outcome data, selective reporting of results<br>> Especially relevant for self-reported outcomes like QoL and self-efficacy, which are prone to performance and detection bias<br><br>**Small sample sizes and limited power**<br>> Several studies had small populations, limiting their ability to detect statistically significant effects<br>> Underpowered trials also contributed to wide confidence intervals in meta-analyses<br><br>**Heterogeneity**<br>> Considerable variation in: type of digital intervention (apps, web tools, video, etc.), components included (education, feedback, monitoring, etc.), duration and intensity of interventions, and outcome measures used<br>> This heterogeneity made meta-analyses challenging and weakened generalizability<br><br>**Inconsistent or Missing Outcome Reporting**<br>> Medication adherence, though a key behaviour target, was not systematically assessed<br>> Tools for QoL, dyspnoea, and exacerbation varied across studies, reducing comparability<br><br>**Multi-component Interventions Limit Attribution**<br>> Most digital interventions included multiple behaviour change techniques, making it difficult to identify which component were responsible for observed effects<br>> Lack of standardization across studies prevented analysis<br><br>**Limited Generalizability**<br>> Most studies were conducted in high-income countries with participants who were technologically literate and had access to the internet and smartphones. This may not reflect populations in low-resource settings, or those with limited digital access or skills
Are COPD self-management mobile applications effective? A systematic review and meta-analysis	2020	Systematic review and meta-analysis	13	RCTs	Older adults with COPD clinical diagnosis<br>> COPD condition ranged between moderate to severe<br>> Mean age ranged from 57 to 74 years old<br>> In most study the gender distribution was balanced<br>> Studies were conducted in diverse locations, including the Netherlands, United Kingdom, United States, and China.	Mobile application to support self-management of COPD<br>**Technology**<br>> Smartphone apps: 11 trials<br>> Tablet-based apps: 2 trials<br>> Wearables or connected devices: 5 trials included peripheral monitoring devices, such as pedometers and pulse oximeters<br><br>**Core components**<br>> Symptom monitoring: Users logged daily symptoms via the app.<br>> Feedback: Automated or clinician-generated feedback based on input data.<br>> Alerts/notifications: Some apps triggered alerts for deteriorating symptoms or adherence lapses.<br>> Education: Information modules on disease management, inhaler use, etc.<br>> Self-management support: Goal setting, prompts to encourage behavior changes like increased activity.<br>> Telemonitoring: 7 trials involved remote clinician oversight of the app-entered data.<br><br>**Duration of the interventions**<br>> Ranged from 2 weeks up to 12 months	The control group went under usual care<br><br>> Regular in-person visits to primary or secondary care providers<br>> Prescribed medications<br>> Pulmonary rehabilitation programs<br>> Standard educational materials (e.g., leaflets, verbal guidance)<br>> No access to digital apps or telemonitoring tools used in the intervention group<br>> Some control groups had basic phone contact but not app-based features<br><br>But the specifics varied depending on the setting and country of each study	• **Primary outcomes** across the studies were exacerbation frequency and hospital admissions.<br>> Exacerbation frequency: Mixed and generally non-significant effects; only one study reported pre/post differences; low certainty. Assessed via self-report or clinical intervention records (hospitalization, community-managed).<br>> Hospital admissions: Not consistently reported; findings were inconclusive across trials; low to very low certainty.<br><br>• **Quality of life** measures were assessed using<br>> SGRQ: Small, non-significant changes across 3 trials; improvements did not exceed > MID of 4 points; very low certainty.<br>> CAT: Reported in 2 trials; no clinically meaningful differences; very low certainty.<br>> CRQ: Small improvements (3–8 points) in total and domain scores, but did not meet > MID of 10 points; very low certainty.<br>> CCQ: Functional domain showed statistically significant improvement in one trial; total score changes non-significant; low certainty.<br>> SF-12: One study showed significant PCS improvement (+9.2 points), exceeding MID of 3–3.5; low certainty.<br>> SF-36: Used in 2 trials; non-significant results; very low certainty.<br>Other measures (e.g., EQ-5D) not used.<br><br>• **Medication adherence**<br>Not reported or assessed in any of the included trials.<br><br>• **Disease management metrics**<br>> Dyspnoea (CRQ-dyspnoea, MRC): One study showed improvement; others showed no significant changes; low to very low certainty.<br>> Fatigue (CRQ, MFI, FACIT): No significant improvements reported; very low certainty.<br>> Physical activity (accelerometers/pedometers): One study reported a statistically significant increase in daily activity (+9.5 min/day); others reported non-significant increases; low certainty.<br>> Self-efficacy: Assessed using various scales (SECD6, General/Exercise SE); no consistent or significant effects; very low certainty.<br>> Anxiety and depression (HADS): Reported in 2 studies; no statistically significant findings; very low certainty.<br><br>• **Direction/magnitude of change**<br>> QoL (SGRQ): MD = −2 to −4.5 points – small, non-significant improvement; below MID<br>> QoL (CRQ): MD = +3 to +8 points – below MID of 10<br>> QoL (SF-12): MD = +9.2 (PCS) in one trial – clinically significant improvement<br>> Physical Function (6MWT): MD = +8.38 meters (95% CI: −4.40 to 21.17); not statistically significant<br>> QoL meta-analysis: SMD = −0.4 (95% CI: −0.86 to 0.05); p = 0.08 – no significant overall effect<br>> Physical Activity: MD = +9.5 min/day; p < 0.01 – significant improvement in one trial<br>> Dyspnoea: Significant improvement; details not quantified in pooled format<br>> Exacerbations: 4 vs. 26 community-managed events; not statistically significant	Meta-analyses showed no statistically significant effects of mobile health applications on primary clinical outcomes in COPD patients.<br><br>> Quality of life, measured across eight trials using various instruments (SGRQ, CAT, CRQ, CCQ, SF-12/36), showed a small, non-significant benefit (SMD = –0.40, 95% CI: –0.86 to 0.05, p = 0.08). Results were highly heterogeneous (I^2 = 83%), limiting interpretability<br>> Physical function, measured via 6-minute walk test in five trials, also showed no significant difference between groups (MD = 8.38 meters, 95% CI: –4.40 to 21.17, p = 0.20; I^2 = 52%)<br>Single-study results suggested possible benefits for physical activity and dyspnoea.<br>> One trial reported a significant increase in physical activity (MD = +9.5 min/day, p < 0.01)<br>> Another trial showed improved dyspnoea scores, though the effect size was not pooled<br>> Exacerbation frequency and hospital admissions were inconsistently reported and showed no clear benefit<br><br>Self-efficacy, fatigue, and mood outcomes did not show consistent or significant effects.<br>> All trials assessing these domains (using SECD6, MFI, FACIT, and HADS) reported non-significant results.<br>> No adverse events related to digital intervention use were reported, and heterogeneity, small sample sizes, and variation in outcome measures limited the reliability of pooled estimates. No formal assessment of publication bias was performed.	Assessed using the I^2 statistic.<br>Heterogeneity was reported and quantified for the main meta-analyses. I^2 values ranged from 52% to 83%, indicating moderate to considerable heterogeneity in pooled outcomes. Heterogeneity was highest for quality of life outcomes, attributed to differences in intervention types (e.g., apps with vs. without clinician input), outcome measurement tools (e.g., SGRQ vs. CCQ), follow-up durations, and study settings. No subgroup analyses were conducted to explore potential effect modifiers.<br><br>> QoL (8 studies, multiple tools): I^2 = 83%<br>> Physical function (6MWT, 5 studies): I^2 = 52%<br>> For exacerbations, dyspnoea (CRQ-dyspnoea, MRC), physical activity, self-efficacy, fatigue, anxiety, and depression was not pooled and heterogeneity not calculated<br>> Hospital admissions: Not pooled consistently across studies<br><br>No formal Q statistics or publication bias analyses were reported.	**Overall Effectiveness**<br>Mobile health applications for COPD self-management showed inconclusive effectiveness compared to usual care.<br>Meta-analyses revealed no statistically or clinically significant improvements in primary outcomes such as exacerbation rates, physical function, or quality of life.<br>Only a few individual trials demonstrated positive results, which were not consistent across studies.<br><br>**Drivers of Effectiveness**<br>> Use of smartphone-based apps with symptom monitoring and feedback<br>> Integration of wearable devices (e.g., pedometers, pulse oximeters) that support patient tracking<br>> Involvement of healthcare professionals in data review and intervention when symptoms worsen<br>> Inclusion of educational content and behavior change components (e.g., activity goals)<br><br>**Clinical Implications**<br>Digital interventions have potential to support COPD self-management, but current evidence does not justify routine clinical implementation<br>The lack of standardized outcome measures hinders comparability and clinical interpretation<br>Apps that include active monitoring, personalized feedback, and professional oversight may offer greater value<br><br>**Future Directions**<br>> Development of a core outcome set for evaluating digital interventions in COPD<br>> Design of larger, high-quality RCTs with consistent methodologies and adequate follow-up<br>> Emphasis on tailored interventions that integrate patient needs, usability, and technological features<br>> Consideration of cost-effectiveness and scalability in implementation research	> Heterogeneity in interventions and outcomes: Included trials varied widely in their design, components (e.g., educational content, monitoring, professional input), and the outcomes assessed. This variability limited the ability to perform consistent and meaningful meta-analyses.<br><br>> Small sample sizes: The majority of trials (8 out of 13) enrolled fewer than 100 participants, reducing statistical power and increasing the risk of type II error.<br><br>> Risk of bias: Many trials lacked adequate blinding (participants and outcome assessors), and several had unclear allocation concealment. This introduces potential bias, particularly in subjective outcomes like quality of life.<br><br>> Inconsistent outcome measures: Trials used a wide range of tools to measure the same domains (e.g., different QoL questionnaires), complicating comparisons and pooling of results.<br><br>> Short follow-up durations: Some studies had very short intervention periods (as little as 2 weeks), which may not be sufficient to observe meaningful changes in COPD management outcomes.<br><br>> Lack of standardization: The absence of a core outcome set for COPD digital interventions contributed to reporting inconsistencies and limited synthesis.<br><br>> No assessment of medication adherence: Despite being a key dimension of self-management, none of the included studies systematically evaluated medication adherence.<br><br>> Potential for publication bias: Only published trials were included, and no formal publication bias assessments (e.g., funnel plots) were conducted.<br><br>> Technology evolution: The rapid pace of digital health innovation may limit the applicability of findings to newer or more advanced apps not represented in the review.
Effectiveness of eHealth Interventions in improving medication adherence for patients with chronic obstructive pulmonary disease or asthma: Systematic review	2021	Systematic Review	13 (6 COPD, 7 asthma)	RCTs	Adults diagnosed with COPD or asthma<br><br>> Age: Asthma studies generally involved younger adult populations (median/mean ~30–50 years), while COPD studies involved older adults (~65–75 years).<br>> Gender: Proportion of female participants varied widely, ranging from 6.7% to 76.5%, often higher in asthma trials.<br>> Condition severity: COPD patients tended to have moderate to severe disease, often post-discharge or under chronic management. Asthma patients were mostly mild to moderate persistent, generally stable, but with adherence issues.<br>> Geographic coverage: Studies were concentrated in Europe (esp. UK, Denmark), the US, and China, with only one in Oceania and none from low-income or underrepresented regions like Africa or South America.	Digital interventions included telemonitoring, interactive voice response systems, internet-based tools, and SMS/text messaging systems.<br><br>These eHealth solutions were delivered via<br>> Telephone (live or automated)<br>> Internet platforms (web portals, tablets, robots)<br>> Mobile-based SMS<br>> Interactive voice response (IVR)<br>> Email (in one study)<br><br>**Key components included**<br>> Monitoring of symptoms and/or medication adherence (via self-report, devices, > or clinician input)<br>> Education and counseling (e.g., inhaler technique, disease understanding, > smoking cessation)<br>> Behavioral and motivational support (based on models like > Information-Motivation-Behavioral Skills)<br>> Automated reminders (for medication intake or refills)<br>> Feedback and treatment adjustment support<br>> Self-support modules (e.g., psychological well-being, action planning)<br>> Pharmacist or clinician interaction (via phone or platform)<br><br>**Duration**<br>> Ranged from 2 to 18 months, with most interventions lasting 3 to 12 months.<br>> Shorter durations (2–4 months) were common in pilot and feasibility trials; longer durations (>12 months) were used in large-scale pragmatic or integrated care trials.	Comparators primarily involved usual care without digital components. These included:<br><br>> Routine clinical management by general practitioners or specialists<br>> Printed or verbal education on asthma/COPD and medication use<br>> Face-to-face counseling (in some cases)<br>> Standard discharge instructions or mailed prescription refills<br>> Monitoring and treatment as per national or clinical guidelines<br><br>No comparator groups included digital or automated elements, ensuring a clear contrast with eHealth interventions.	• **Primary outcomes**: Most studies measured medication adherence using self-report scales (e.g., MARS, Morisky), electronic tracking devices, pill counts, or pharmacy data. Several studies showed significant improvement in adherence, while others reported mixed or nonsignificant findings<br><br>• **Quality of life**: Not consistently assessed; no use of standardized QoL instruments like SGRQ, CAT, or EQ-5D.<br><br>• **Medication adherence**: was the central outcome of all included studies. Improvements ranged from +13% to +49% depending on the metric and population. Tools used included MARS, pill count, adherence ratios, and inhaler sensor tracking.<br><br>• **Disease management**: limited and mostly indirect.<br><br>• **Direction and magnitude**: Digital interventions showed a generally positive effect on medication adherence. About half of the studies reported statistically significant improvements, with p-values ranging from .04 to <.001, indicating small to moderate effects. Some studies showed mixed results, with significant improvements limited to specific adherence metrics (e.g., inhaler technique or controller use). A few studies reported no significant differences. Overall, effectiveness varied depending on adherence definitions, measurement methods, and intervention intensity.<br>Significant effects included:<br>>Pill count adherence in COPD (p = .04)<br>>Inhaler adherence rates in COPD (71% vs. 37%; p = .009)<br>>Self-reported ICS use in asthma (87% vs. 54%; p < .001)<br>>Objective inhaler tracking in asthma (49.1% vs. 64.5% missed doses; p = .003)	No meta-analysis was conducted due to substantial heterogeneity in study design, intervention types, adherence definitions, and measurement tools.<br><br>Statistically significant improvements in medication adherence were reported in approximately half of the included studies, with between-group p-values ranging from .04 to <.001. Effect directions favored the intervention group across various adherence metrics (e.g., pill count, inhaler tracking, self-report). Some studies reported mixed effects, with improvements seen only in specific adherence measures (e.g., inhaler technique). Several studies showed no significant differences. No pooled effect sizes or confidence intervals were reported.	Heterogeneity was reported and identified as a major limitation. No meta-analysis was performed due to variations in study design, intervention type, adherence measures, and statistical reporting. No formal heterogeneity statistics (e.g., I^2) were calculated. Differences in delivery format, intensity, and operational definitions of adherence were considered key sources of heterogeneity.	Digital health interventions show promise in improving medication adherence among patients with COPD and asthma, but the overall evidence is mixed and inconclusive.<br><br>While some interventions led to statistically significant improvements, others had limited or no effects, and many results were highly dependent on how adherence was defined and measured. The variation in intervention type, delivery mode, intensity, and context contributed to these inconsistencies.<br><br>Emphasis on<br>> The potential of eHealth is evident, particularly for tailored, interactive, and multicomponent interventions.<br>> However, more high-quality, standardized research is needed to determine what works best, for whom, and under what conditions.<br>> No definitive conclusions could be drawn about which digital interventions are most effective for medication adherence in these populations.	**Heterogeneity in Study Design**<br>Significant variability across included RCTs in:<br>> Study populations (age, severity, setting)<br>> Intervention types (e.g., SMS vs. multicomponent systems)<br>> Adherence definitions and metrics<br>> Duration and intensity of interventions<br>This precluded pooling of data and prevented meta-analysis.<br><br>**Inconsistent Measurement of Adherence**<br>Studies used a wide range of adherence tools, including:<br>> Self-report questionnaires (e.g., MARS, Morisky)<br>> Electronic monitoring (e.g., inhaler sensors)<br>> Pill counts or refill data<br>These tools vary in objectivity, accuracy, and sensitivity, complicating cross-study comparisons.<br><br>**Risk of Bias**<br>Many studies had a high risk of performance and detection bias due to:<br>> Lack of blinding<br>> Self-reported outcomes (subject to social desirability and recall bias)<br>Some studies also had attrition bias from incomplete follow-up or per-protocol analysis.<br><br>**Small Sample Sizes and Limited Power**<br>> Several trials had small sample sizes, limiting their statistical power to detect meaningful effects.<br>> Lack of subgroup analysis further constrained interpretation of which populations benefit most.<br><br>**Lack of Standardized QoL or Clinical Outcomes**<br>> Few studies assessed clinical endpoints like exacerbations, ER visits, or quality of life.<br>> Absence of standardized, validated outcome sets limits clinical relevance and decision-making.
Benefits and Harms of Digital Health Interventions Promoting Physical Activity in People With Chronic Conditions: Systematic Review and Meta-Analysis	2023	Systematic review and meta-analysis	130	RCTs	Adults with one or more chronic conditions<br>> Only 26 RCTs focused on COPD patients<br>Major conditions are<br>> Ischemic Heart Disease or Heart Failure: 41 RCTs<br>> Type 2 Diabetes (41 RCTs)<br>Minor conditions are<br>> Knee or Hip Osteoarthritis, Mental Health Conditions, Hypertension<br><br>**Demographics**<br>> Mean age: 61 years<br>> Gender distribution: 41.6% female<br><br>**Top contributing countries**<br>> United States: 24 RCTs<br>> Australia: 21 RCTs	Digital health interventions were delivered via a variety of technologies and categorized as:<br>> mHealth (e.g., mobile apps, SMS, phone calls) – 56 RCTs<br>> eHealth (e.g., websites, emails, web portals) – 29 RCTs<br>> Digital Devices (e.g., wearables, pedometers, Bluetooth devices) – 14 RCTs<br>Combinations used:<br>> mHealth + device: 22 RCTs<br>> eHealth + device: 6 RCTs<br>> All 3 combined: 2 RCTs<br><br>The interventions included various behavior change and support strategies such as:<br>> Physical activity monitoring (e.g., steps, intensity)<br>> Feedback and goal setting (automated or personalized)<br>> Educational modules (condition-specific guidance, PA advice)<br>> Reminders and prompts (SMS, app notifications)<br>> Motivational messages, self-management tools, and adherence tracking<br>> Often guided by a theoretical framework (used in 52.6% of RCTs)<br><br>Interventions varied widely in length<br>> On average, interventions lasted about 23 weeks, but some were as short as 4 weeks and others extended up to 2 years (104 weeks)<br>> Participants typically received around 98 digital sessions per intervention, though this ranged from as few as 2 sessions to as many as 377<br>> When in-person sessions were included, the average number was just over 3 sessions, with some studies offering none and others up to 18 sessions	Digital intervention compared with standard care approaches<br>> Usal care included standard medical management, follow-up with healthcare providers, or access to in-person services without any digital enhancement<br>> Minima interventions included educational materials (booklets, leaflets, videos) and blinded wearables (pedometers)<br>> Wait-list control or delayed intervention: in a few trials, control participants were placed on a wait-list and offered the digital intervention after the study period ended	• **Primary outcomes**: objectively measured physical activity<br>> Tools: accelerometers, pedometers, wearables<br>> Metrics reported: Steps/day, moderate-to-vigorous physical activity (MVPA)<br>> Effect at intervention end: SMD: 0.29 (95% CI 0.21–0.37), Mean increase: +971 steps/day (CI 657 to 1284)<br>>Follow-up: SMD 0.17 (CI 0.04–0.31); effect largely sustained<br><br>• **Quality of life measures**<br>> Instruments Used: EQ-5D – Generic measure of health-related quality of life (HRQoL) and disease-specific tools (not always specified per condition)<br>> Effect (End of Intervention): SMD: 0.18 (95% CI 0.07–0.29) – in favor of digital health interventions. Certainty: Low, due to heterogeneity and possible small study bias<br>> Effect (Follow-Up): No statistically significant between-group difference sustained<br><br>• **Medication adherence**: not systematically assessed<br><br>• **Disease management metrics**<br>> Physical activity levels: daily steps and MVPA<br>> Self-care behaviors and self-management: implicitly addressed via behavior change strategies, but not quantified across studies	Digital health interventions showed small but significant effects compared to control.<br>> Objectively measured physical activity: SMD = 0.29 (95% CI 0.21–0.37); ~+971 steps/day<br>> Physical function (6MWT): SMD = 0.36 (95% CI 0.12–0.59); ~+19.8m<br>> Subjective physical activity: SMD = 0.29; sustained at follow-up (SMD = 0.36)<br>> Depression: SMD = –0.25 (CI –0.35 to –0.16); Anxiety: SMD = –0.17<br>> HRQoL: SMD = 0.18; effect not sustained at follow-up<br>> Non-serious AEs: RR = 1.31 (↑ risk); Serious AEs: RR = 0.89 (NS)	Assessed using I^2 statistics, with values ranging from moderate to high across most outcomes:<br>> Physical activity (steps/day): I^2 = 54.0%<br>> Physical function (6MWT): I^2 = 75.9%<br>> Subjective physical activity: I^2 = 81.7%<br>> HRQoL: I^2 = 84%<br><br>Possible sources:<br>> Age and BMI as moderators (older age and higher BMI = smaller effects)<br>> Intervention characteristics such as duration and use of theoretical frameworks also influenced outcomes<br><br>Much of the heterogeneity remained unexplained	Digital health interventions promoting physical activity were found to be modestly effective across multiple chronic conditions, including COPD. Specifically:<br><br>> Objectively measured physical activity and physical function showed small but significant improvements compared to usual care or minimal intervention, both at the end of the intervention and, though attenuated, at follow-up.<br><br>> Secondary outcomes, such as depression, anxiety, and health-related quality of life, also improved at the end of the intervention, but these effects were not sustained over time.<br><br>> The risk of non-serious adverse events (e.g., soreness, pain) was higher in intervention groups, but no increase in serious adverse events (e.g., hospitalization, disability) was observed.<br><br>> Heterogeneity in effects was substantial, and although some variance was explained by age, BMI, intervention format, and duration, a large portion remained unexplained.<br><br>> The evidence quality ranged from moderate to very low, and small study bias may have influenced some of the observed effects.<br><br>> Despite modest effects, digital interventions are considered safe, scalable, and potentially cost-effective options for increasing physical activity and supporting disease management.<br><br>> Authors emphasize that intervention effects largely diminish post-intervention, highlighting the need for long-term engagement strategies.<br><br>Future research should focus on:<br>> Comparing specific digital interventions modalities<br>> Improving reporting quality<br>> Evaluating effects in multimorbidity<br>> Understanding the impact of digital health literacy and the digital divide	**High heterogeneity** across studies in terms of populations, intervention types, delivery methods, and outcome measures; much of this variability remained unexplained despite subgroup and meta-regression analyses.<br><br>**Risk of bias** was common, with many studies rated as having "some concerns" or "high risk" due to issues in randomization, blinding, outcome reporting, and handling of missing data.<br><br>**Short follow-up durations** limited the ability to assess long-term effectiveness; most positive effects were observed only at the end of the intervention and were not sustained.<br><br>**Important outcomes** such as medication adherence, hospital admissions, ER visits, and COPD-specific measures were **not consistently reported** or analyzed.<br><br>**Small study effects** and potential publication bias were detected, especially for subjective outcomes like self-reported physical activity and quality of life, potentially leading to inflated effect sizes.<br><br>**Poor reporting** on critical participant characteristics such as digital literacy, socioeconomic status, and comorbidities limits insights into equity, access, and generalizability.<br><br>**Multimorbidity** and digital health equity were **rarely addressed**, despite their importance for real-world application and implementation of digital interventions.
Clinical Efficacy of Mobile App-Based, Self-Directed Pulmonary Rehabilitation for Patients With Chronic Obstructive Pulmonary Disease: Systematic Review and Meta-Analysis	2024	Systematic review and meta-analysis	10 (9 used in the meta-analysis)	RCTs	Patients diagnosed with COPD with different levels of severity or diagnostic criteria (depending on the studies)<br>> FEV1/FVC < 70%<br>> Restricted to specific GOLD stages or stability<br><br>Mean age: older adults between 62 and 72 years<br>Gender distribution: mostly male<br>Country represented: Romania, United Kingdom, Belgium, China, South Korea, Switzerland, Netherlands, and Taiwan	**Type of digital intervention**<br>The digital intervention used were custom developed apps for COPD rehabilitation (7/10 studies).<br>Some studies applied commercial apps<br>> myCOPD (2 studies)<br>> WeChat (1 study)<br><br>**Components** commonly used<br>> Exercise Program: core feature across all studies. Some apps allowed adjustable levels<br>> Monitoring: activity tracking, symptom reporting, or exercise feedback<br>> Disease Education: educational content on COPD management and inhaler use<br>> Professional Contact: direct communication with healthcare providers during the program<br>> Self-management Support: tools for self-care behavior, goal setting, or inhaler technique improvement<br>> Incentives & Social Support: included in 1 study (WeChat – users earned mall rewards, had peer interaction)<br><br>**Durations** ranged from 3 weeks to 12 months, with the majority falling in the 3- to 6-month range	All studies used standard care or conventional pulmonary rehabilitation as the comparator. The majority (9 out of 10) involved in-person or clinic-based programs without any digital component (structured exercise regimens, sometimes combined with education or follow-up care).<br><br>> Center-based Pulmonary Rehabilitation: Traditional, supervised sessions in hospital or clinic settings<br>> Educational Programs: In-person COPD management education without app support<br>> Usual/Standard Care: Routine post-exacerbation or outpatient follow-up without structured rehab	**Primary outcomes**<br>Mobile app–based interventions were associated with a reduction in COPD exacerbations and hospitalizations, though statistical significance varied:<br><br>> Exacerbation Rates: One study reported a reduction in mean exacerbations from 2.9 to 1.06 in the intervention group versus 3.2 to 1.88 in controls. Other studies also showed fewer exacerbations, but not consistently statistically significant.<br>> Hospital Admissions: One study observed 1 hospitalization in the app group versus 2 in controls; another reported a lower readmission rate (0.24 vs. 0.39).<br>Overall, these outcomes favored the digital intervention group but lacked statistical power for firm conclusions.<br><br>**Quality of life**<br>Was commonly assessed and showed consistent improvement in the digital intervention arms:<br><br>> CAT was reported in 9 studies and showed statistically significant improvement in the intervention group. The meta-analysis revealed a mean difference of –1.29 points (95% CI –2.39 to –0.20; P = .02).<br>> SGRQ, reported in 3 studies, showed non-significant trends toward improvement (e.g., Jiang: 50.2 → 39.7 vs. 49.6 → 44.2).<br>> EQ-5D was reported in one study with a slight non-clinical improvement in the app group (0.6 → 0.7 vs. 0.7 → 0.7).<br><br>Other instruments included the CCQ, HAD, WPAI, and Ex-SRES, each showing modest improvements without strong statistical support.<br><br>**Medication Adherence**<br>Was poorly reported, with just one study evaluating the adherence using SEAMS, with a minor improvement in the intervention group.<br><br>**Disease Management Metrics**<br>Several studies evaluated metrics reflecting functional independence, symptom control, and patient engagement:<br><br>> Physical Activity (steps/day): One study reported an increase of ~462 steps/day in the intervention group, while other studies tracked step counts and walking time.<br>> Inhaler Technique: Two studies reported improved inhaler usage skills, with fewer critical errors in the intervention group.<br>> Self-Care Behavior: One study noted an improvement in self-care scores (112.9 to 122.3 in the intervention group vs. minimal change in controls).<br>> Symptom Scores (mMRC Dyspnea Scale): Reported in 5 studies, with slight improvements in dyspnea but no statistically significant effect (meta-analysis MD = –0.08; P = .45).<br>> Exercise Self-Regulation: One study reported gains in Ex-SRES scores, indicating increased confidence in managing activity levels.	CAT scores significantly improved in the intervention group (MD = –1.29; 95% CI –2.39 to –0.20; P = .02).<br><br>6MWD showed a non-significant improvement (MD = +9.52 m; 95% CI –3.05 to 22.08; P = .14).<br><br>mMRC dyspnea scale and SGRQ scores showed no significant differences (MD = –0.08 and –3.62 respectively; P > .05).<br><br>Hospitalizations were reduced but not significantly (RR = 0.65; 95% CI 0.27–1.53; P = .32).<br><br>Subgroup analysis revealed greater CAT improvement in patients with baseline CAT ≥ 20 (MD = –2.16; P = .02) and in those receiving both exercise and self-management interventions.	Statistical heterogeneity was assessed using the I^2 statistic and Chi-square tests<br>> For all pooled outcomes (6MWD, CAT, mMRC, SGRQ, hospitalizations), no important heterogeneity was detected. I^2 values were low or not substantial, suggesting consistent findings across studies.<br>> Funnel plots for 6MWD, CAT, mMRC, SGRQ, and hospitalization outcomes appeared symmetrical, indicating low risk of publication bias.<br><br>Subgroup analyses were performed to explore heterogeneity by:<br>> Baseline severity (e.g., CAT ≥ 20 vs. < 20; 6MWD ≥ 400m vs. < 400m)<br>> Intervention type (exercise only vs. exercise + self-management)	The review concluded that mobile app–based, self-rehabilitation is a viable and promising alternative to conventional rehabilitation for patients with COPD, especially when traditional programs are inaccessible or impractical. Despite the heterogeneity, the evidence showed that:<br><br>> Exercise capacity, symptom burden, quality of life, and hospitalization outcomes generally favored the digital interventions.<br>> A statistically significant improvement was observed in CAT scores, indicating reduced symptom burden in patients using mobile apps.<br>> While improvements in 6MWD, SGRQ, and hospitalizations were noted, these did not reach statistical significance, suggesting positive trends that warrant further exploration.<br>> Apps that combined exercise training with self-management education and monitoring showed more pronounced effects than exercise-only apps.<br>> In the context of barriers to conventional rehab (e.g., cost, travel, COVID-19 limitations), mobile solutions were considered feasible, scalable, and clinically beneficial.<br><br>Moreover, mobile app–based programs that are interactive and customizable can enhance patient engagement and compliance	**Heterogeneity Across Studies**<br>There was considerable variation in:<br>> Participant characteristics (e.g., baseline disease severity, post-exacerbation status, previous rehab experience).<br>> Intervention components (some apps included only exercise, while others added education, monitoring, or social support).<br>> Outcome measures (different tools and timeframes used across studies).<br>These discrepancies complicated pooled analyses and reduced comparability across trials.<br><br>**Limited Sample Sizes**<br>Many of the included studies had small participant numbers (6 out of 10 had <100 participants). This reduced statistical power and may have contributed to non-significant findings in several outcome domains (e.g., 6MWD, SGRQ, hospitalizations).<br><br>**Short Follow-up Durations**<br>Follow-up periods ranged from 3 weeks to 12 months, with most being 3–6 months. This limited the ability to detect long-term effects on exacerbation rates, hospitalizations, and disease progression.<br><br>**Incomplete Reporting on App Use and Adherence**<br>Most studies did not report detailed data on app usage, such as frequency of engagement, dropout rates, or patient adherence. This hindered the ability to analyze the relationship between app engagement and clinical outcomes.<br><br>**Performance Bias**<br>Blinding was not feasible due to the nature of digital interventions. All studies were assessed as having a high risk of performance bias, which may have introduced expectation-related effects.<br><br>**Limited Evidence on Medication Adherence**<br>Only one study assessed medication adherence using the SEAMS scale, and no objective adherence metrics (e.g., refill rates) were used.

Comparative effectiveness of eHealth interventions on the exercise endurance and quality of life of patients with COPD: A systematic review and network meta-analysis	2024	Systematic review and network meta-analysis	51	RCTs	Adults with stable COPD not in acute exacerbation phase<br>> Age range from 51 to 83 years, with a mean age between 60 and 75 years<br>> Majority of the participants were male, but sex ratios ranged broadly (e.g., some samples had ≥90% male, while others had a more balanced)<br><br>The studies were multinational, conducted across countries including China, UK, Australia, Spain, USA, and Denmark.<br>Settings varied and included hospitals, outpatient clinics, home environments, and community settings.	The review categorized six main types of digital health interventions.<br><br>**Telemonitoring**<br>Remote monitoring of physiological or health status data by healthcare providers.<br>Key Components:<br>> Electronic devices to transmit data<br>> Two-way communication with clinicians<br>> Alerts and automated feedback<br>> Patient education and self-management support<br>Duration: Ranged from 12 to 56 weeks.<br><br>**Application**<br>Smartphone/mobile health applications used by patients for COPD management.<br>Key Components:<br>> Educational content (videos, diagrams).<br>> Exercise tracking and reminders.<br>> Breathing techniques and symptom diaries.<br>> Medication tracking or guidance.<br>Duration: Typically 12 to 48 weeks.<br><br>**Web-based or Internet-based Interventions**<br>Interventions delivered via websites or online portals (often computer or tablet-based).<br>Key Components:<br>> Structured modules (e.g., pulmonary rehab).<br>> Online coaching or support.<br>> Health information and videos.<br>> Monitoring and feedback tools.<br>Duration: Broad range, 6 to 48 weeks.<br><br>**Telephone Support**<br>Interventions involving structured support via regular phone contact.<br>Key Components:<br>> Scheduled calls for health coaching or symptom check-ins.<br>> Motivational interviewing or behavioral support.<br>> Reinforcement of rehabilitation adherence.<br>Duration: Generally 6 to 48 weeks.<br><br>**Virtual Reality**<br>Use of immersive or semi-immersive VR technologies to support pulmonary rehabilitation.<br>Key Components:<br>> Interactive breathing exercises.<br>> Gamified physical activity.<br>> Visual and audio simulation for motivation.<br>Duration: 2 to 20 weeks.<br><br>**Combined Interventions** (≥2 types)<br>Integrations of two or more digital modalities (e.g., APP + phone, web + telemonitoring).<br>Key Components: Varying combinations of the elements listed above.<br>Duration: Most commonly 12 to 48 weeks.<br><br>**General**<br>Designed to improve exercise adherence, enhance disease self-management, monitor symptoms and physiological signs, and support emotional well-being and reduce dyspnea-related anxiety.<br>Some incorporated tailored feedback or interactive features	The comparator groups across the included studies were all non-digital interventions, primarily consisting of:<br>> Usual care, which included standard pharmacological management, routine clinical follow-ups, and unstructured advice without digital support.<br>> Traditional face-to-face pulmonary rehabilitation, offered in some studies as in-person supervised exercise and education programs.<br>> Phone call-based support, used in a few studies as a lower-intensity control when compared to more complex digital interventions.<br>> Waiting list control, where participants received no active intervention during the study period.<br>> Printed educational materials, occasionally used to provide minimal intervention for control group participants.	**Primary outcomes**<br>The review focused on two primary outcomes: exercise endurance and quality of life.<br>Exercise endurance was consistently measured using the 6-Minute Walk Test (6MWT).<br>eHealth interventions showed significant improvements in 6MWT compared to control:<br>> Telemonitoring: MD = +43.03 meters (95% CI: 14.55 to 83.53)<br>> APP: MD = +25.74 meters (95% CI: 17.48 to 33.99)<br>> VR: MD = +18.43 meters (95% CI: 3.09 to 33.77)<br>> Combined interventions: MD = +25.67 meters (95% CI: 10.16 to 41.18)<br><br>**Quality of life measures**<br>Measured using the CAT SGRQ<br>Significant improvements were found in quality of life scores with:<br>> APP: SMD = −0.47 (95% CI: −0.68 to −0.26)<br>> Web-based interventions: SMD = −1.49 (95% CI: −2.51 to −0.46)<br>> VR: SMD = −0.47 (95% CI: −0.90 to −0.04)<br>Negative SMD indicates improvement in QoL (lower scores = better health status).<br>Web-based interventions ranked highest for QoL enhancement (SUCRA: 94.3%).<br><br>**Medication adherence**: not directly measured or reported<br><br>**Disease management metrics**: not meta-analyzed, but discussed qualitatively. Improvements in self-management, patient engagement, and potential reductions in readmissions or exacerbations were suggested in the discussion section.<br><br>**Instruments used and direction/magnitude of change**<br>> 6MWT: All effective interventions (telemonitoring, APP, VR, combined) showed positive change (increase in distance walked).<br>> CAT & SGRQ: Significant reduction in scores indicates improved quality of life.<br>> Direction of change was consistently favorable for eHealth interventions over standard care.	Exercise Endurance (6MWT):<br>> Telemonitoring: MD = +43.03 meters (95% CI: 14.55 to 83.53), I^2 = 12%<br>> APP: MD = +25.74 meters (95% CI: 17.48 to 33.99), I^2 = 32.3%<br>> VR: MD = +18.43 meters (95% CI: 3.09 to 33.77), I^2 = 0%<br>> Combined interventions: MD = +25.67 meters (95% CI: 10.16 to 41.18), I^2 = 68.8%<br>> Web-based interventions: MD = 18.44 (95% CI: -19.32 to 56.20), I^2 = 96.4% (not significant)<br><br>Quality of Life (SGRQ, CAT):<br>> APP: SMD = -0.47 (95% CI: -0.68 to -0.26), I^2 = 68.9%<br>> Web-based interventions: SMD = -1.49 (95% CI: -2.51 to -0.46), I^2 = 97.8%<br>> VR: SMD = -0.47 (95% CI: -0.90 to -0.04), I^2 = 0%<br>> Telemonitoring, Phone calls, and Combined: Not statistically significant<br><br>Network Meta-Analysis Rankings (SUCRA):<br>> Exercise Endurance: Telemonitoring (86.2%), Combined (76.2%), APP (59.8%)<br>> Quality of Life: Web-based (94.3%), APP (73.4%), VR (63.1%)<br><br>Inconsistency & Sensitivity Analyses:<br>> No significant inconsistency (global p > 0.05).<br>> Sensitivity analysis confirmed robustness.<br>> Publication bias detected for exercise endurance (Egger's p = 0.019); not for QoL (p = 0.102)	Heterogeneity was assessed using I^2 statistics, Cochrane's Q test, and network meta-analysis inconsistency models.<br><br>> Exercise endurance heterogeneity ranged from low (Telemonitoring: I^2 = 12%) to very high (Web-based: I^2 = 96.4%).<br>> Quality of life heterogeneity was substantial to very high, particularly for Web-based (I^2 = 97.8%), Combined (I^2 = 97.1%), and Telemonitoring (I^2 = 72.2%).<br><br>Global network consistency tests showed no significant inconsistency:<br>> Exercise endurance: p = 0.839<br>> Quality of life: p = 0.779<br><br>Side-splitting (local inconsistency) analysis also found no significant inconsistencies (all p > 0.65).<br><br>Sources of heterogeneity included variations in intervention design, duration, measurement instruments (SGRQ vs. CAT), and population characteristics.<br><br>Sensitivity analyses excluding high-risk-of-bias trials showed consistent findings, supporting the robustness of the pooled results.	The review concluded that eHealth interventions are effective in improving exercise endurance and quality of life in COPD patients.<br><br>Telemonitoring was found to be the most effective for enhancing exercise capacity, while web-based interventions were most effective for improving quality of life.<br><br>Other effective interventions included APPs, VR, and combined digital strategies.<br><br>eHealth interventions can overcome limitations of traditional rehabilitation (e.g., access, cost, logistics), offering personalized and remote support.<br><br>The authors emphasized the importance of tailoring digital tools to patient capabilities, particularly older adults, and highlighted the need for provider training and system-level support.<br><br>They recommended further large-scale, high-quality RCTs to optimize digital strategies and validate long-term benefits.	High heterogeneity was observed in some intervention comparisons, especially for web-based and combined modalities (e.g., I^2 > 90%), limiting generalizability.<br><br>Many included studies had some concerns or high risk of bias, particularly related to missing data and deviations from intended interventions.<br><br>Several effect estimates were derived through data transformations (e.g., from p-values), introducing possible estimation bias.<br><br>The review did not meta-analyze medication adherence or hospital readmissions, restricting the scope of conclusions to exercise endurance and quality of life.<br><br>There was no subgroup analysis by COPD severity or disease duration due to inconsistent reporting across trials.<br><br>The review did not directly evaluate digital literacy or usability, which may affect real-world applicability for older COPD patients.
The Effects of Digital Health Interventions for Pulmonary Rehabilitation in People with COPD: A Systematic Review of Randomized Controlled Trials	2024	Systematic review	13	RCTs	Patients diagnosed with COPD with severity ranged from mild to very severe.<br><br>Individual study sample sizes ranged from 34 to 409 participants.<br><br>Adults aged between 44 to 75 years, with mean ages typically in the 60s to early 70s<br><br>Most studies had a slightly higher proportion of male participants, though some were close to gender-balanced<br><br>Geographic distribution:<br>>Europe: 8 studies (Netherlands, UK, Greece, Denmark ×2, Spain, Switzerland, Norway)<br>> Asia: 2 studies (China, Iran)<br>> North America: 2 studies (USA, Canada)<br>> Australia: 1 study	Digital intervention designed for pulmonary rehabilitation categorized into:<br><br>> Web-based platforms (e.g., coaching programs, self-management systems)<br>> Smartphone applications (used for exercise guidance, progress tracking, and education)<br>> Videoconferencing systems (delivering live PR sessions with supervision and education)<br>> Online PR programs (structured digital versions of traditional PR, e.g., "myPR")<br><br>Key Features of Interventions<br>> Exercise training (endurance, strength, flexibility, breathing)<br>> Educational content (disease management, symptom awareness)<br>> Self-management tools (goal setting, monitoring, motivation)<br>> Personalization (intensity adjusted via Borg scale or clinical indicators)<br><br>Duration: Ranged from 6 weeks to 12 months, with frequency between 2–5 sessions/week.	Digital interventions compared with:<br><br>Standard/In-person pulmonary rehabilitation<br>Conventional face-to-face rehabilitation delivered in hospital or clinic settings.<br>> Frequency: Typically 2–3 sessions per week, involving aerobic, strength, and breathing exercises along with education.<br><br>Usual medical or standard care<br>Routine outpatient care involving pharmacotherapy, lifestyle advice, and follow-ups. No structured PR component.<br>> Frequency: Daily routine management, no structured sessions<br><br>Unsupervised home exercise or activity monitoring<br>Patients used wearable devices or received basic instructions but had no access to structured digital PR tools.<br>> Frequency: Daily activity tracking, no guided exercise, 6 weeks<br><br>Face-to-face education<br>In-person education and self-management without the use of digital support.<br>> Frequency: 3–6 months with weekly self-managed exercise and educational sessions.<br><br>Comprehensive clinical follow-up<br>Includes pharmacotherapy, oxygen therapy, vaccinations, and regular hospital visits.<br>> Frequency: Year-long routine care, no PR, hospital-based support only	**Primary Outcomes**<br>Digital health interventions consistently demonstrated improvements in functional exercise capacity, often matching or surpassing traditional pulmonary rehabilitation.<br>> 6-Minute Walk Test (6MWT): Significant improvements observed in multiple studies, particularly in interventions with structured exercise and feedback components (e.g., P < 0.001 in several trials).<br>> 12MWT and ESWT: Also showed gains, especially in digitally guided, personalized exercise settings.<br>> Steps/day (PAL): Increased significantly in digital PR groups (P = 0.007), indicating better physical activity adherence.<br><br>Other tests (e.g., Sit-to-Stand, TUG): Less frequently used but suggested functional improvements.<br><br>Direction of change: Improved physical capacity in most digital PR groups.<br><br>**Quality of Life Measures**<br>Was widely assessed, with multiple validated tools demonstrating positive effects.<br><br>> SGRQ: Improved in both digital and standard PR groups; in some trials, greater improvement was seen with digital interventions.<br>> CAT: Frequently decreased (improvement) in intervention groups (e.g., P = 0.02, P = 0.04).<br>> CRQ: Gains noted in dyspnoea and fatigue domains; less consistent in emotional function.<br>> SF-36: Both physical and mental composite scores improved significantly in digital PR participants (e.g., PCS P = 0.039; MCS P = 0.001).<br>> EQ-5D: No group exceeded MCID in at least one study, suggesting limited sensitivity or marginal clinical benefit in some settings.<br><br>Direction of change: Most digital interventions led to improved QoL, especially in physical health domains.<br><br>**Medication adherence**<br>Not directly and systematically evaluated<br><br>**Disease Management Metrics**<br>Digital PR demonstrated benefits in broader disease management outcomes.<br><br>> Exacerbations, ED visits, hospitalizations: Several studies reported significant reductions (P < 0.001 to P = 0.0008).<br>> Dyspnoea (mMRC): Improved in digital groups; often better than usual care (P < 0.001, P = 0.037).<br>> Self-efficacy (PRAISE, GSES): Mixed findings; some improvements in digital arms (P = 0.007), though not consistently significant.<br>> HADS (Anxiety/Depression): Variable outcomes—some studies showed improvement in digital groups, others found no difference or even worsening in controls.<br><br>Direction of change:<br>> Fewer hospital visits, exacerbations, and dyspnoea;<br>> Improved self-efficacy in some interventions;<br>> Mixed psychosocial impact.	6-Minute Walk Distance (6MWD) significantly improved in several intervention groups (e.g., MD ≈ +23.8 m), though results varied. In other studies, improvements were non-significant (P > .05).<br><br>CAT scores significantly improved in digital intervention groups (MD ≈ –1.29; 95% CI –2.39 to –0.20; P = .02 and P = .04 in different trials).<br><br>CRQ subdomains showed statistically significant improvements in dyspnoea (MD ≈ +0.45; P = .033) and fatigue (P = .028), but emotional function and mastery were not significantly affected (P > .05).<br><br>SF-36 PCS and MCS improved in intervention groups, with MCS showing a strong effect (P = .001), and PCS showing moderate improvement (P = .039), while EQ-5D changes did not exceed the minimal clinically important difference (MCID).<br><br>mMRC dyspnoea scale decreased significantly in some studies (P < .001; P = .037), though others found no effect.<br><br>Hospitalizations and ED visits were significantly reduced in some cases (P < .001; P = .0008), though non-significant reductions were also observed (RR = 0.65; 95% CI 0.27–1.53; P = .32).<br><br>HADS scores (anxiety and depression) showed mixed results: some significant reductions (P = .001 for depression; P = .04 for anxiety), while other findings were non-significant (P > .05).<br><br>Subgroup analysis revealed greater CAT improvement in patients with baseline CAT ≥ 20 (MD = –2.16; P = .02) and in those receiving both exercise and self-management interventions.	Meta-analysis was not conducted due to substantial clinical and methodological heterogeneity across the 13 included trials. Variability was observed in intervention types (e.g., apps, web platforms, video sessions), duration (6 weeks to 12 months), frequency, outcome measures (e.g., 6MWT, CAT, SGRQ), comparators (e.g., standard care, face-to-face PR), and participant characteristics (age, COPD severity). No I^2 or Q-statistics were reported, and subgroup analysis was not performed at the review level. Results were synthesized narratively.	Digital health–delivered pulmonary rehabilitation through mobile apps, web platforms, or videoconferencing is a viable and effective alternative to conventional rehabilitation for patients with COPD, particularly in settings where access to in-person programs is limited.<br><br>Despite substantial heterogeneity across studies, the evidence indicated that:<br><br>> Exercise capacity, symptom control, quality of life, and healthcare utilization generally improved with digital interventions compared to standard care.<br>> CAT scores showed statistically significant reductions, reflecting meaningful symptom improvement in patients using digital platforms.<br>> Positive trends were observed in 6MWD, SGRQ scores, and reduced hospitalizations, although not all reached statistical significance, highlighting the need for further large-scale trials.<br>> Multicomponent interventions that integrated exercise with self-management education and patient monitoring were more effective than exercise-only approaches.<br>> In light of common barriers to traditional PR, including cost, travel demands, and public health restrictions, digital formats were found to be feasible, accessible, and clinically advantageous.<br><br>Furthermore, digital PR programs that are interactive, tailored, and feedback-driven may support greater patient engagement, adherence, and long-term self-management.	**Heterogeneity Across Studies**<br>Considerable variability in:<br>> Types of digital interventions (apps, web, video)<br>> Duration and frequency of programs (6 weeks to 12 months)<br>> Outcome measures (e.g., CAT, 6MWT, CRQ, SGRQ)<br>> Comparator groups (standard care, in-person PR, education-only)<br>This heterogeneity prevented meta-analysis and limited direct comparison or effect size estimation.<br><br>**Small Sample Sizes**<br>Many trials were underpowered, with sample sizes ranging from 34 to 150 participants. This raises the risk of type II error (failing to detect real effects) and reduces the strength of conclusions.<br><br>**Limited Scope of Outcomes**<br>Several important outcomes were not consistently reported:<br>> Medication adherence was not assessed in any included trial.<br>> Some trials omitted key PR indicators (e.g., self-efficacy, exacerbation frequency).<br>Variation in outcome instruments also reduced comparability.<br><br>**Variable Quality of Included Trials**<br>Although PEDro scores ranged from 6 to 9 (good to excellent), some studies showed:<br>> Lack of blinding (patients, therapists, or assessors)<br>> Incomplete allocation concealment<br>Two studies were rated as having "some concerns" of bias using the Cochrane RoB 2 tool.<br><br>**Limited Generalizability**<br>Most trials were conducted in high-income countries with reliable internet access and healthcare infrastructure. Findings may not generalize to low-resource or rural settings with limited digital access.<br><br>**Lack of Long-Term Follow-Up**<br>Follow-up periods were generally short to moderate (3–12 months), limiting insights into:<br>> Sustained benefits<br>> Long-term adherence<br>> Durability of behavior change
Benchmarking the clinical outcomes of Healthentia SaMD in chronic disease management: a systematic literature review comparison	2024	Systematic review	35	RCTs (27), Pilot (1), Feasibility study (3), Cluster-RCTs (1), Non-RCTs (3)	Adults diagnosed with chronic diseases, including type 2 diabetes mellitus, cardiovascular conditions (heart failure, hypertension, post-MI), cancer, and COPD.<br>Disease severity ranged from mild to severe, often focusing on patients with poor control or recent exacerbations/hospitalizations.<br><br>Sample sizes ranged from 7 to 409 participants.<br>Participant ages typically ranged from 44 to 80 years, with mean ages in the 60s to early 70s depending on condition. Most studies had a slightly higher proportion of male participants, though several were gender-balanced.<br><br>Geographic distribution:<br>> Europe: 15 studies (e.g., Netherlands, UK, Spain, Finland, France)<br>> Asia: 11 studies (e.g., China, South Korea, India, Iran)<br>> North America: 6 studies (USA, Canada)<br>> Australia: 2 studies<br>> Multinational/unspecified: 1 study	Digital intervention designed for pulmonary rehabilitation categorized into:<br><br>> Web-based platforms (e.g., coaching programs, self-management systems)<br>> Smartphone applications (used for exercise guidance, progress tracking, and education)<br>> Videoconferencing systems (delivering live PR sessions with supervision and education)<br>> Online PR programs (structured digital versions of traditional PR, e.g., ""myPR"")<br><br>Key Features of Interventions<br>> Exercise training (endurance, strength, flexibility, breathing)<br>> Educational content (disease management, symptom awareness)<br>> Self-management tools (goal setting, monitoring, motivation)<br>> Personalization (intensity adjusted via Borg scale or clinical indicators)<br><br>Duration: Ranged from 6 weeks to 12 months, with frequency between 2–5 sessions/week	Digital interventions compared against non-digital standard care<br>Usual care / Standard care<br>> Routine in-person clinical visits, standard disease management protocols, physician consultations, and patient-initiated contact<br>Paper-based self-management plans<br>> Printed action plans for exacerbation monitoring or symptom tracking<br>Passive education / information-only controls<br>> Printed educational booklets or verbal advice provided at baseline but with no ongoing support or tracking<br>Non-interactive monitoring tools<br>> in some heart failure or diabetes studies, control groups used devices like blood glucose meters or home BP monitors without connected apps or feedback systems<br>Alternative non-digital interventions<br>> Support phone calls, nurse consultations, or community-based programs without mHealth integration<br><br>The comparator group did not receive any form of automated tracking, reminders, messaging, or clinical alerts	**Primary outcomes**<br>The review focused on several primary outcomes depending on disease area, including glycemic control (HbA1c), blood pressure, hospital readmissions, and COPD exacerbations.<br><br>Glycemic control was the most consistently measured outcome across diabetes studies.<br>eHealth interventions showed statistically significant reductions in HbA1c levels compared to control:<br>mHealth App (e.g., Yutangyihu): MD = −1.95% vs. −1.83% (control)<br>Smart Glucose Manager (SGM): MD = −0.96% (additional reduction at 6 months; p < 0.0001)<br>Hicare Smart K app: MD = −0.30% (p = 0.003)<br><br>Blood pressure was reported in multiple T2DM and cardiovascular studies:<br>mDPP App: significant reductions in both systolic and diastolic BP (p < 0.05)<br>ANODE platform: trend toward improved BP and dietary sodium intake<br><br>Exacerbation-related outcomes (COPD)<br>Lu et al.: ER visits MD = −0.70 (95% CI: −1.36 to −0.03)<br>Jang et al.: ER visits MD = −0.14 (95% CI: −0.28 to −0.01)<br>Boer et al.: No significant difference in exacerbation-free weeks (RR = 1.21; 95% CI: 0.77–1.91)<br><br>Hospital readmissions<br>Significant reductions were observed in two studies:<br>Wang et al. (T2DM): readmissions post-discharge decreased by 1.19 episodes (p < 0.05)<br>Park et al. (HF): 30-day readmissions = 10% (vs. national average of 25%)<br><br>**Quality of life measures**<br>Measured using instruments such as SF-36, EQ-5D, DQoL, EORTC QLQ-C30.<br>Significant improvements were found in quality of life scores with:<br>Aftercare App (lung cancer): EORTC QLQ-C30 global health status improved by +68 meters (6MWD as a functional correlate)<br>Davoudi et al. (HF): p < 0.001 in all QoL domains<br>Pain Guard App (cancer): global QoL significantly improved (p < 0.001)<br><br>No significant change reported in:<br>Boer et al. (COPD) and some T2DM studies (e.g., Karhula et al.)<br>QoL results were generally favorable for mHealth interventions, especially when combined with behavioral support or virtual coaching.<br><br>**Medication adherence**<br>Assessed in at least four studies using electronic monitoring, self-reports, and medication logs.<br>Significant improvements were found in:<br>Brath et al. (T2DM): minimal adherence increased from 89% (control) to 93% (intervention)<br>Kleinman et al.: adherence improved (39.0% vs. 12.8%; p = 0.03)<br>Other studies reported improved medication routines and reduced barriers to adherence (e.g., Huang et al., Johnston et al.)<br><br>**Disease management metrics**<br>Not meta-analyzed but reported qualitatively. Improvements were observed in:<br>Self-monitoring behaviors (blood glucose, diet, physical activity)<br>Knowledge of disease and treatment plans<br>Patient engagement and satisfaction<br>Reductions in emergency department visits and hospitalizations (particularly in COPD and HF)<br><br>**Instruments used and direction/magnitude of change**<br>HbA1c: Reduction observed in most T2DM studies using mobile apps and remote monitoring<br>BP: Moderate reductions in systolic and diastolic pressures<br>6MWD: Increased functional capacity (e.g., Aftercare app: +68 meters at 12 weeks)<br>QoL scores (SF-36, QLQ-C30, EQ-5D): Direction consistently favored mHealth over standard care<br>Medication adherence: Improved across all studies reporting it<br>Hospital readmissions/ER visits: Generally reduced in COPD, HF, and diabetes studies using RPM tools<br><br>Direction of change across outcomes was consistently favorable for digital health interventions compared to usual care.	Digital health interventions showed statistically significant improvements across key clinical and behavioral outcomes compared to standard care:<br><br>> HbA1c: Reduced by 0.3% to 1.9% in most T2DM studies (p < 0.001 in meta-analyses)<br>> Blood Pressure: Small but significant reductions in SBP/DBP (p = 0.02)<br>> COPD Outcomes: Fewer ER visits, lower exacerbation-related admissions (e.g., RR = 0.74), and reduced mortality (OR = 0.71)<br>> Medication Adherence: Improved significantly in intervention groups (e.g., 39% vs. 12.8%; p = 0.03)<br>> Physical Activity & Function: Increased step counts and 6MWD (e.g., +68m; p < 0.001)<br>> Quality of Life: Improved in multiple studies (e.g., p = 0.04 to < 0.001)<br><br>Overall, the direction and magnitude of change consistently favored digital interventions, especially when tools included real-time feedback, coaching, and self-monitoring features.	Heterogeneity was addressed based on data from included meta-analyses, though no new statistical pooling or inconsistency modeling was performed by the review authors.<br><br>> Moderate heterogeneity was reported for glycemic control (HbA1c), influenced by intervention duration and type.<br>> COPD outcomes showed variability across studies, with heterogeneity discussed but not re-quantified.<br>> Quality of life results varied due to differences in measurement instruments.<br><br>Subgroup analyses from original sources identified key contributors:<br>> Intervention type and delivery method (e.g., smartphone app vs. SMS)<br>> Intensity and duration of intervention<br>> Use of structured, multicomponent programs<br><br>Sensitivity analyses (in the original reviews) confirmed result stability after excluding low-quality or short-duration trials.<br>Sources of heterogeneity included:<br>> Differences in intervention design and follow-up duration<br>> Varying outcome measurement methods<br>> Diverse participant characteristics (e.g., age, disease severity, digital literacy)<br><br>Overall, heterogeneity was recognized and explored, though not statistically quantified by the umbrella review itself.	The review concluded that digital health interventions—such as mHealth apps and software as medical devices (SaMDs)—are effective tools for improving chronic disease management across a variety of conditions. Despite differences in intervention types and outcome measures, the evidence showed that:<br><br>> Clinical outcomes including glycemic control, blood pressure, exacerbation rates, and hospital readmissions generally favored the use of digital interventions.<br>> Statistically significant improvements were consistently observed in HbA1c reduction across diabetes studies, with several showing reductions ≥0.4%.<br>> Although not all quality of life outcomes reached statistical significance, a favorable trend was observed across studies using validated tools (e.g., SF-36, QLQ-C30, EQ-5D).<br>> Interventions that combined real-time monitoring, behavioral coaching, and feedback mechanisms demonstrated stronger and more consistent results than passive tracking solutions.<br>> In the context of long-term chronic disease care, digital tools were considered scalable, patient-centered, and adaptable for remote or hybrid care delivery models.<br><br>Moreover, platforms that enabled personalized engagement, self-management support, and integration with healthcare providers were associated with increased adherence, patient satisfaction, and better health outcomes.	**Search Scope and Source Limitations**<br>The review was conducted primarily through the EuropePMC database, with supplemental use of Cochrane and PubMed only when necessary.<br>This narrow search strategy may have introduced selection bias, potentially missing relevant studies indexed in other databases.<br><br>**Study Selection**<br>The review focused heavily on systematic reviews and their referenced RCTs, excluding:<br>> Observational studies<br>> Economic evaluations<br>> Non-comparative trials<br>This may limit the comprehensiveness of the evidence base, especially for newer or real-world applications.<br><br>**Heterogeneity Across Studies**<br>There was substantial heterogeneity in:<br>> Intervention types (e.g., passive apps vs. interactive coaching)<br>> Disease conditions<br>> Outcome measures (e.g., HbA1c, QoL, adherence)<br>This made direct comparisons and aggregation of results challenging, and no new meta-analyses were performed.<br><br>**Incomplete Reporting**<br>Some studies lacked detailed demographic or outcome data, especially regarding:<br>> Long-term adherence<br>> Cost-effectiveness<br>> Health system integration<br><br>**Generalizability Concerns**<br>Most included studies were conducted in high-income countries, limiting relevance for low- and middle-income settings. Patient populations varied widely in age, digital literacy, and disease severity, affecting the consistency of results.
Effectiveness of Digital Health Interventions for Chronic Obstructive Pulmonary Disease: Systematic Review and Meta-Analysis	2025	Systematic review and meta-analysis	17	RCTs	COPD patients aged between 62 and 74 years with a moderate to severe diagnosis.<br>The population frequently faced multi-morbidity and reduced mobility.<br><br>Sample sizes per study ranged from 17 to 375 participants, conducted in 11 countries: the USA, China, Sweden, Australia, the Netherlands, Norway, Denmark, Korea, England, Canada, and New Zealand.<br><br>No gender distribution extracted	Two major categories<br><br>**Self-management support tools**<br>Aimed to empower patients in daily disease monitoring and control with: symptom tracking, medication reminders, educational modules, health coaching, remote communication with clinicians, exacerbation alerts (some AI-driven) and vital sign monitoring.<br><br>**Telerehabilitation Platforms**<br>For the delivery of structured exercise regimens using the following components: video-guided pulmonary rehabilitation, activity tracking via wearables, exercise diaries, feedback and goal setting and real-time visual/audio feedback for exercises.<br><br>**Most used instruments/platforms**<br>> WeChat<br>> Mobile apps<br>> Web-based portals<br><br>**Key features**<br>Real-time monitoring: Vital signs (pulse oximetry, BP, weight)<br>Feedback systems: Tailored alerts and clinician review<br>Communication tools: Messaging, video consultations, peer forums<br>Education modules: COPD knowledge, self-care skills<br>Adherence tools: SMS reminders, scheduled calls<br>Device integration:<br>> Pedometers (e.g., Omron HJ-112)<br>> Pulse oximeters<br>> Treadmills/tablets<br>> Accelerometers<br>> Spirometers<br><br>**Duration of interventions**<br>Range: 3 to 24 months<br>Majority: 3–6 months duration<br>A few studies assessed 12-month outcomes, and one extended to 24 months	Usual care without digital intervention, which typically included:<br>> Routine outpatient follow-ups<br>> In-person rehabilitation (clinic- or hospital-based)<br>> General practitioner visits or home care<br>> No structured digital or remote tools<br><br>Some studies provided additional materials or activities to control groups beyond routine care:<br>> Educational Booklets: COPD information on self-management<br>> Paper Action Plans: Hardcopy version of a COPD management plan<br>> Telephone Support: Periodic check-ins or health advice.<br>> In-person Follow-ups: Routine in-person coaching without tech<br>> 12-topic Education Packet: Structured, non-digital education modules<br>> App with limited features: Same app platform, but control users received no active monitoring/alerts	**Primary outcomes**<br>The review focused on four key outcomes: quality of life (QoL), self-efficacy, functional capacity, and health care utilization.<br><br>**Quality of life** was the most consistently improved outcome:<br>> CAT: Significant improvements at 3, 6, and 12 months (e.g., MD = −2.53 at 12 months; p < 0.001)<br>> SGRQ: Improved at 6 months (MD = 3.25; p = 0.01)<br>> EQ-5D: Gains at 12 months (MD = 0.04; p = 0.02)<br>> QoL benefits were strongest with longer intervention duration.<br><br>**Self-efficacy**<br>Improved at 3 months (MD = 1.65; p = 0.002) and 6 months (MD = 1.94; p < 0.001); no sustained improvement at 12 months.<br>Short-term gains suggest initial user confidence in disease control.<br><br>**Functional capacity**<br>> mMRC (dyspnea): Improved at 3 months (MD = −0.23; p < 0.001)<br>> 6MWT: No significant improvement at any time point<br><br>While breathlessness decreased, physical endurance did not improve.<br><br>**Health care utilization**<br>No significant reductions in ED or hospital admissions (all-cause or COPD-specific). All p > 0.05.<br>Attributed to weak integration with care systems and inconsistent patient engagement.<br><br>**Medication adherence**<br>Not a primary focus of the meta-analysis and no quantitative effect estimates provided.<br><br>**Disease management metrics**<br>> Self-care behaviors: Frequently supported through education, symptom tracking, and remote coaching<br>> Activity levels: Promoted in telerehabilitation trials, though not reflected in 6MWT outcomes<br>> ER visits: Captured under utilization metrics; no significant change<br>Digital tools appear to enhance patient engagement in daily management, even without reducing acute care events.<br><br>**Instruments used and direction of change**<br>> QoL (CAT, EQ-5D): Consistently improved<br>> Self-efficacy: Positive short-term effects<br>> Dyspnea (mMRC): Improved at 3 months<br>> Medication adherence: Descriptively supported, not quantified<br>> Utilization and 6MWT: No significant changes<br><br>Overall direction favored digital interventions for symptom relief, self-care, and confidence, but not for clinical events or medication adherence.	The review reported detailed statistical outcomes for multiple domains, primarily using mean differences (MDs) with 95% confidence intervals (CIs) and p-values. Meta-analyses were conducted using fixed or random effects models depending on heterogeneity.<br><br>**Quality of life**<br>CAT:<br>> 3 months: MD = −1.65 (95% CI −3.17 to −0.14; p = 0.03)<br>> 6 months: MD = −2.43 (95% CI −3.93 to −0.94; p = 0.001)<br>> 12 months: MD = −2.53 (95% CI −3.91 to −1.16; p < 0.001)<br><br>SGRQ:<br>> 6 months: MD = 3.25 (95% CI 0.69 to 5.81; p = 0.01)<br><br>EQ-5D:<br>> 12 months: MD = 0.04 (95% CI 0.01 to 0.07; p = 0.02)<br><br>EQ-5D VAS:<br>> 12 months: MD = 5.88 (95% CI 0.38 to 11.37; p = 0.04)<br><br>**Self-efficacy**<br>> 3 months: MD = 1.65 (95% CI 0.62 to 2.69; p = 0.002)<br>> 6 months: MD = 1.94 (95% CI 0.83 to 3.05; p < 0.001)<br>> 12 months: MD = 3.12 (95% CI −2.29 to 8.53; p = 0.26)<br><br>**Dyspnea (mMRC)**<br>> 3 months: MD = −0.23 (95% CI −0.36 to −0.11; p < 0.001)<br>> 6 months: MD = −0.17 (95% CI −0.46 to 0.12; p = 0.26)<br>> 12 months: MD = −0.32 (95% CI −0.64 to 0.01; p = 0.05)<br><br>**Functional performance** (6MWT)<br>> 3 months: MD = 25.01 (95% CI −45.34 to 95.36; p = 0.49)<br>> 6 months: MD = −1.53 (95% CI −21.98 to 18.93; p = 0.88)<br><br>**Health care utilization**<br>> ED admissions: MD = −0.08 (95% CI −0.35 to 0.19; p = 0.57)<br>> Hospital admissions: MD = −0.19 (95% CI −0.44 to 0.05; p = 0.13)<br>> COPD-specific ED admissions: MD = −0.07 (95% CI −0.22 to 0.09; p = 0.40)<br>> COPD-specific hospital admissions: MD = −0.19 (95% CI −0.39 to 0.02; p = 0.07)<br><br>Interpretation<br>Strongest effects were observed in QoL (CAT) and self-efficacy (short-term), with low to moderate heterogeneity.<br>No significant effects in 6MWT or health care use, despite trend-level reductions in hospitalizations.	Assessed using the I^2 statistic, and random- or fixed-effects models.<br><br>**Low or no heterogeneity**<br>> CAT at 3, 6, and 12 months: I^2 = 0% to 12%<br>> mMRC (dyspnea) at 3 and 12 months: I^2 = 0%<br>> Health care utilization outcomes: I^2 = 0% for all comparisons<br>These findings suggest high consistency in effects for symptom relief and hospital use outcomes.<br><br>**Moderate heterogeneity**<br>> SGRQ at 6 months: I^2 = 37%<br>> Dyspnea at 6 months: I^2 = 50%<br>Reflects some variability in delivery models and comparator groups across studies.<br><br>**High heterogeneity**<br>> SGRQ at 3 months: I^2 = 91%<br>> EQ-5D at 3 months: I^2 = 92%<br>> Overall QoL at 3 months (combined measures): I^2 = 80%<br>> Self-efficacy at 12 months: I^2 = 91%<br>> 6MWT at 3 months: I^2 = 94%<br><br>High heterogeneity in these outcomes was attributed to:<br>> Variations in intervention focus (e.g., symptom tracking vs. rehab)<br>> Differences in comparator intensity (active vs. passive usual care)<br>> Population diversity (age, severity, comorbidities)<br>> Study design features (sample size, follow-up length)<br><br>No subgroup meta-analyses were conducted due to the limited number of studies	Digital health interventions show promise for improving several key outcomes in patients with COPD, particularly in quality of life, self-efficacy, and symptom relief. However, their overall clinical effectiveness remains uncertain due to methodological and implementation-related limitations.<br><br>**Effective** in:<br>> Quality of life: Especially with longer-duration interventions; consistent improvements in CAT, SGRQ, and EQ-5D at 6–12 months.<br>> Self-efficacy: Short-term gains at 3 and 6 months, likely driven by real-time feedback and support.<br>> Dyspnea (mMRC): Significant relief reported at 3 months, with trends sustained at 12 months.<br><br>**Not effective** in:<br>> Functional capacity (6MWT): No significant improvements at any time point.<br>> Health care utilization: No reductions in ED or hospital admissions.<br>> Medication adherence: Reported descriptively but not consistently measured or improved.<br><br>**Overall**<br>Digital tools may be most useful for patient education, symptom tracking, and behavioral support, but less effective at changing hard clinical outcomes.<br><br>Long-term engagement is a challenge, particularly for older adults, and "digital health fatigue" may reduce sustained benefits.<br><br>More robust, longer-term studies with standardized outcomes are needed to guide implementation.	> High heterogeneity: Wide variation in digital tool design, intervention content, and comparator conditions made it difficult to pool results, particularly for quality of life and functional outcomes.<br><br>> Small sample sizes and short durations: Many studies had limited participants and follow-up periods, reducing the reliability of long-term effect estimates.<br><br>> Limited reporting on adherence and engagement: Few trials described how they maintained user participation over time, which is critical for digital intervention success.<br><br>> Exclusion of real-world studies: Only RCTs were included, omitting valuable insights from pragmatic or observational studies that reflect typical clinical settings.<br><br>> Lack of integration with health systems: Most digital tools were standalone and did not connect with clinical workflows or enable timely interventions.

Telehealth improves quality of life of COPD patients: systematic review and meta-analysis	2024	Systematic review and meta-analysis	10	RCTs	Patients diagnosed with moderate to severe COPD.<br>Some studies focused on patients with frequent exacerbations or advanced symptoms.<br><br>The age ranged approximately from 63 to 71 years old.<br>No gender distribution summary performed.<br>The studies were conducted in high- and middle-income countries like the Netherlands, the UK, the US, Denmark, Korea, China, and Switzerland.	All studies evaluated telehealth interventions as the primary digital strategy.<br>The **modalities** used were mHealth applications, web-based platforms, and tablet systems with monitoring devices.<br><br>**Components**<br>Across studies, interventions typically included a combination of:<br><br>Education and Self-Management Support<br>> COPD disease education (e.g., causes, symptom recognition)<br>> Inhaler techniques (Farmer et al.)<br>> Breathing and coughing exercises<br>> Pulmonary rehabilitation content (Park et al., Spielmanns et al.)<br>> Real-time feedback and behavior modification coaching<br><br>Monitoring and Feedback<br>> Symptom tracking: breathlessness, sputum changes<br>> Vital signs: oxygen saturation, weight, lung function<br>> Activity tracking: daily steps using pedometers (e.g., Moy et al.)<br>> Telemonitoring: CHROMED system (Walker et al.)<br><br>Communication and Support<br>> Digital consultation platforms or messaging features with healthcare providers<br>> Peer chatrooms and forums for social support (e.g., Wang et al.)<br>> Feedback from trained professionals (e.g., Tupper et al., Spielmanns et al.)<br><br>Behavior Change Aids<br>> Motivational messages and reminders<br>> Visual content (videos, animations)<br>> Goal-setting modules and rewards<br><br>**Duration** of Interventions ranged from 3 months to 12 months, with the longest lasting 6–12 months.	Intervention group compared against standard care, like:<br>Face-to-face clinical follow-up: Most studies included routine in-person consultations with physicians or nurses following hospital discharge or diagnosis.<br><br>Pamphlets and educational material: Several control groups received printed educational materials, often on topics such as inhaler use or COPD symptom management.<br><br>No digital or telehealth components: Usual care excluded any use of mobile apps, telemonitoring, or internet-based support tools. This contrast was necessary to isolate the effect of digital interventions.<br><br>Routine care protocols: Care in the control groups followed existing national or local COPD management guidelines.	The primary focus was on quality of life (QoL), with secondary references to self-management and medication adherence.<br><br>**Primary Outcome: Quality of Life**<br>Most studies reported statistically significant improvements in QoL among participants who received telehealth support. These improvements were typically measured using:<br>> EQ-5D<br>> SGRQ<br>> CAT<br>> CRQ<br>> CCQ<br>> 15D<br>9 out of 10 studies reported positive, statistically significant effects on QoL with telehealth interventions, and just one study (Boer et al., 2019) reported no significant improvement.<br><br>**Disease Management Metrics**<br>While not the primary focus, some studies indirectly reported outcomes relevant to disease self-management:<br>> Self-monitoring compliance (e.g., pedometer data in Moy et al., online symptom reporting in Tupper et al.)<br>> Physical activity levels were encouraged via exercise prompts and monitored in apps.<br>> Improved self-efficacy and engagement with disease-related behaviors were noted, especially in education-focused apps like EDGE.<br><br>Although these outcomes were not always measured quantitatively, the authors noted improvements in self-care behaviors and digital engagement as key contributors to the observed benefits in QoL.<br><br>**Medication Adherence** was not quantitatively assessed across studies. Some interventions (e.g., apps with reminders, education modules) were designed to support adherence, but the review did not extract or report adherence metrics.<br><br>**Effect of change**<br>Quality of Life<br>> Significant Improvement in most studies<br>> Tools: EQ-5D, SGRQ, CAT, CRQ, CCQ, 15D<br><br>Self-management<br>> Behavior change, education, engagement<br>> Tools: N/A Supported but not systematically quantified<br><br>Medication adherence<br>> Not directly assessed<br>> Indirectly supported in intervention design	Quality of Life (QoL): Improved significantly across most included studies<br>• Meta-analysis: SMD = 0.27 (95% CI: 0.03 to 0.50), p = 0.02<br>• Instruments used: EQ-5D, SGRQ, CAT, CRQ, CCQ, 15D<br>• Consistent improvement in self-reported QoL post-intervention<br><br>Study-Level Effects: Effect sizes varied across trials<br>• Tupper et al. (2018): SMD = 0.29 (95% CI: 0.05 to 0.52)<br>• Spielmanns et al. (2023): SMD = 0.42 (95% CI: –0.09 to 0.93)<br>• Tabak et al. (2014): SMD = 2.90 (95% CI: 1.70 to 4.10) – notably higher than others<br>• Several studies (e.g., Moy et al., Walker et al.) reported small or non-significant effects<br><br>Heterogeneity: Moderate to high across included studies<br>• I^2 = 70% (indicating variability in intervention effects)<br>• Tau^2 = 0.08; Chi^2 = 30.26 (df = 9), p = 0.0004<br>• Sources of heterogeneity: intervention type, intensity, and duration differences<br><br>Publication Bias: Low risk based on visual inspection<br>• Funnel plot analysis indicated symmetrical distribution<br>• Suggests minimal publication bias among included studies<br><br>Overall, the direction and magnitude of change consistently favored telehealth interventions. Digital tools that incorporated education, remote monitoring, and self-management support were especially effective in enhancing patient quality of life.	The heterogeneity was assessed using:<br><br>> I^2 statistic: 70% - moderate to high heterogeneity<br>> Chi^2: 30.26 with 9 degrees of freedom, p-value: 0.0004 - the variation between studies is unlikely to be due to random chance<br>> Tau^2: 0.08 - this variance reflects meaningful differences in how interventions were implemented<br><br>**Sources of Heterogeneity**<br>No subgroup analysis performed.<br>The authors noted several heterogeneity contributors:<br><br>Intervention differences:<br>Some studies used mobile apps; others used web platforms or tablets with very different durations (from 20 days to 12 months). The components ranged from education-only apps to complex telemonitoring systems<br><br>Outcome measurement variation:<br>QoL was measured using diverse tools (SGRQ, EQ-5D, CAT, CRQ, CCQ, 15D), which are not directly comparable.<br><br>Population differences:<br>Participants were recruited from different countries with varying healthcare contexts, age ranges, and disease severity levels.<br><br>One potential outlier:<br>Tabak et al. (2014) reported an SMD of 2.90, far higher than all other studies, likely inflating overall heterogeneity	Telehealth demonstrates significant potential for enhancing the quality of life in COPD patients, particularly when integrated with education, symptom monitoring, and behavioral support tools. While the overall effect is positive, variability across interventions and populations limits generalizability.<br><br>**Effective in:**<br>> Quality of life: Consistent improvements reported across nearly all studies using EQ-5D, SGRQ, CAT, and CRQ; effect size small to moderate (SMD = 0.27; p = 0.02), with greatest gains observed in interventions incorporating personalized feedback and self-monitoring tools.<br>> Self-management behaviors: Improvements noted in patient engagement, education use, and physical activity (e.g., pedometer tracking, exercise coaching), although outcomes were described narratively rather than quantified.<br>> Health literacy and empowerment: Increased patient understanding of COPD and management strategies, especially in app-based interventions with visual and interactive components.<br><br>**Not directly assessed or inconclusive in**:<br>> Medication adherence: Not systematically measured in the included studies; some interventions included support features (e.g., reminders, inhaler tutorials), but adherence outcomes were not reported quantitatively.<br>> Clinical endpoints (e.g., exacerbations, ER visits, mortality): Not included in the scope of this review; authors focused exclusively on quality of life and did not extract or analyze data on acute care utilization or survival.<br>> Mechanism of effect: While improvements in behavior and QoL were observed, the pathways through which telehealth exerts its benefits remain poorly understood.<br><br>**Overall**<br>Telehealth appears to be an effective adjunct to standard care in COPD management, particularly for improving patient-centered outcomes such as quality of life and self-care behaviors. Its success seems tied to features like real-time feedback, digital education, and remote monitoring.<br><br>However, the diversity in intervention types, delivery duration, and measurement tools introduces moderate to high heterogeneity (I^2 = 70%), warranting cautious interpretation of pooled results.<br><br>To solidify implementation in routine practice, future trials should include standardized outcome measures, assess broader clinical endpoints, and evaluate long-term sustainability	**Blinding and Study Quality**<br>Lack of participant blinding was a major limitation in many included RCTs. This raises the risk of performance and response bias, particularly in subjective outcomes like self-reported quality of life.<br><br>**Heterogeneity in Interventions**<br>The included telehealth interventions were highly variable in:<br>> Duration (ranging from 20 days to 12 months)<br>> Delivery platform (mobile app, tablet, web interface)<br>> Content (education-only vs. exercise-based vs. remote monitoring)<br>This intervention diversity likely contributed to the moderate-to-high heterogeneity in the meta-analysis, complicating the interpretation of pooled effect sizes.<br><br>**Limited Scope of Outcomes**<br>The review focused exclusively on quality of life as the primary outcome.<br>> Important clinical endpoints like hospital readmission, emergency room visits, mortality, or exacerbation rates were not extracted or analyzed.<br>> Medication adherence and functional capacity were also not systematically measured, despite being relevant to COPD management.<br><br>**Lack of Mechanistic Insight**<br>While the review demonstrated that telehealth improved QoL, it did not explore the mechanisms through which this occurred. For instance, it remains unclear whether improvements were due to education, behavior change, symptom monitoring, or patient-provider interaction.<br><br>**Limited Generalizability**<br>Most included studies were conducted in high- or middle-income countries with access to digital infrastructure and healthcare support. Results may not generalize to low-resource settings or patients with low digital literacy, particularly older adults or socioeconomically disadvantaged populations.
Mobile Phone-Mediated Interventions to Improve Adherence to Prescribed Treatment in Chronic Obstructive Pulmonary Disease: A Systematic Review	2025	Systematic review	13	12 RCTs and 1 comparative cohort study	COPD patients with an average stage ranging from moderate to severe disease.<br>Some studies specifically targeted stable COPD, recently hospitalized, or frequently exacerbating patients.<br><br>The average age was 67 years with predominat male population (61%).<br><br>Studies were conducted in a diverse range of countries: Spain, China, Turkey, the USA, the Netherlands, the UK, Australia, Taiwan, and Hong Kong.	Focused on mobile phone-mediated digital health tools. The interventions fell into three categories:<br><br>**Mobile applications** with the following functions:<br>> Medication reminders<br>> Symptom tracking<br>> Exercise/activity monitoring<br>> Progress visualization<br>> Real-time feedback from healthcare providers<br>> Motivational messages<br><br>**SMS/Text Messaging Systems** used for:<br>> Reminders (take medication, exercise, or attend appointments)<br>> Symptom check-ins<br>> Communication with healthcare professionals<br><br>**Internet-Based Learning Platforms**:<br>> Interactive educational content (videos or quizzes)<br>> Modules tailored for disease understanding and self-management<br><br>Overall, they aimed to improve adherence, promote self-monitoring, and early detection via personalized support.<br><br>**Duration** of Interventions<br>Varied significantly across studies:<br>> Short-term interventions: as little as 8 weeks<br>> Medium-term: several months (e.g., 6-month app-based interventions)<br>> Long-term: up to 12 months in telerehabilitation trials	Digital interventions compared against **usual care**, like:<br>> Regular medical follow-up by general practitioners or specialists.<br>> Standard pharmacological treatment according to clinical guidelines.<br>> In some studies, educational booklets or brochures were included.<br>> No structured digital component or additional behavioral support.<br><br>**No Intervention / Wait-and-See**<br>Some control groups received no active intervention, allowing comparison to a natural progression of usual patient behavior. This served as a baseline for evaluating the standalone effect of mHealth interventions.<br><br>**Non-Digital Active Controls**<br>Used to ensure comparison to interventions without a mobile/digital element:<br>> Paper-based action plans (e.g., Boer et al.) for managing exacerbations.<br>> DVDs or brochures (e.g., Liu et al.) containing educational material and home exercise programs.<br>> Physiotherapy sessions or physical activity recommendations delivered offline (e.g., Tabak et al., Walters et al.)	**Primary Outcomes**<br>Digital interventions showed modest improvements in treatment adherence, especially when interactive features like reminders or feedback were included.<br><br>**Adherence**:<br>> Direct improvements in 2 studies (e.g., 87% vs. 66% adherence; P < 0.01).<br>> Other studies inferred adherence via app usage and self-report.<br><br>Direction of change: Generally positive, with higher engagement in intervention groups.<br><br>**Quality of Life Measures**<br>Assessed in 8 studies using validated tools; most showed improvement in intervention groups.<br>Tools used: CRQ, SGRQ, SF-12, CCQ:<br>> CRQ: +12.4 points in IG (P < 0.001)<br>> SGRQ: Less deterioration in IG vs. CG in several studies (P = 0.03–0.04)<br><br>Direction of change: Improved QoL, especially in physical and symptom-related domains.<br><br>**Medication Adherence**<br>Not directly measured in most studies but inferred from behavior (e.g., logging, follow-ups) rather than medication-taking rates.<br><br>**Disease Management Metrics**<br>Mixed but promising results on broader disease outcomes.<br>> Hospitalizations: Reduced in 3 studies (e.g., 13.7% vs. 29.1% readmission; P = 0.005); increased in 2.<br>> Physical activity: Mixed—some improvements (ISWT +51 m; P < 0.001), others no change or decline.<br>> Lung function: Mostly unchanged; one study showed gains (P = 0.002).<br>Self-care: Improved in educational interventions.<br><br>Direction of change: Fewer admissions and better self-care in some studies; physical outcomes variable.	Quantitative outcomes were reported for key domains, but results were variable across studies and often limited by small sample sizes.<br><br>**Treatment Adherence**<br>> Nguyen et al.: IG adherence = 87% (95% CI: 83–91) vs. CG = 66% (95% CI: 61–71); P < 0.01<br>> Galdiz et al.: Compliance rate = 60%; P = 0.045<br><br>**Physical Activity / Functional Capacity**<br>> Nguyen et al. (ISWT): +51.3 m in IG; P < 0.001<br>> Liu et al. (6MWT): IG −46 m vs. CG +68 m; P = 0.02<br>> Tabak et al.: Steps/day changes not statistically significant; P = 0.09–0.12<br><br>**Quality of Life**<br>> Galdiz et al. (CRQ): +12.4 points in IG (95% CI: +10.2 to +14.6); P < 0.001<br>> Walters et al. (SGRQ): CG deterioration significant; P = 0.03, IG not significant; P = 0.12<br>> Chau et al.: QoL decline less in IG (+2.3 pts) than CG (+7.8 pts); P = 0.04<br><br>**Hospitalizations**<br>> Ko Wai-San et al.: Readmission IG = 13.7% (95% CI: 10–17), CG = 29.1% (95% CI: 24–34); P = 0.005<br>> Halpin et al.: IG had more hospitalizations (7) vs. CG (3); P = 0.02<br><br>**Lung Function**<br>> Çevirme et al.: FEV_1 and FVC significantly improved in IG; Z = 3.103, P = 0.002<br>> Other studies reported no significant differences (P > 0.18)	Not quantified via I^2, but high methodological and clinical heterogeneity acknowledged.<br><br>Sources:<br>> small sample sizes,<br>> different mHealth types<br>> different intervention durations<br>> inconsistent outcome tools<br><br>All of this limits comparability and generalizability	Mobile phone-mediated interventions for COPD patients show potential benefits, particularly in treatment adherence and self-management, but the evidence remains inconclusive due to methodological limitations.<br><br>**Effectiveness**<br>> mHealth interventions may improve adherence, quality of life, and engagement in physical activity.<br>> Evidence for reduction in hospitalizations and improvements in lung function is mixed.<br>> Some interventions showed statistically significant benefits, but these were not consistent across studies.<br><br>**Quality of Evidence**<br>> Most studies had small sample sizes, short follow-up periods, and moderate risk of bias.<br>> Wide heterogeneity in interventions and outcome measures limits confidence in the findings.<br><br>**Clinical Implications**<br>> mHealth tools are promising for improving access and continuity of care, especially in underserved or remote populations.<br>> Can support behavioral changes, education, and symptom monitoring, contributing to more proactive disease management.<br><br>**Overall**<br>Current evidence suggests mHealth could improve adherence and self-management in COPD, but definitive conclusions cannot yet be drawn. Findings support further investment in rigorously designed studies to guide clinical adoption.	**Small Sample Sizes**<br>> Most included studies had limited numbers of participants, reducing the statistical power to detect meaningful effects.<br><br>**Short Intervention Durations**<br>> Interventions often lasted less than six months, which hindered the ability to assess long-term adherence and outcomes.<br><br>**Methodological Heterogeneity**<br>> The studies varied widely in intervention design, delivery modes, and measured outcomes, complicating comparisons.<br>> No meta-analysis was conducted due to this heterogeneity.<br><br>**Moderate to High Risk of Bias**<br>> A majority of studies had a moderate risk of bias, and two were judged to be at high risk.<br>> Common issues included lack of blinding, incomplete data, and unreported outcomes.<br><br>**Lack of Standardized Adherence Metrics**<br>> Adherence was measured using different proxies or self-reported data, making cross-study comparisons difficult.<br><br>**Limited Generalizability**<br>> Studies primarily included older adults with access to technology, excluding those with severe comorbidities or digital illiteracy.<br>> Findings may not apply to more diverse or underserved populations.<br><br>**No Subgroup Analyses**<br>> The review did not explore how factors like age, disease severity, or technology familiarity may have moderated intervention effects.
Cost-Effectiveness of Digital Health Interventions for Asthma or COPD: Systematic Review	2024	Systematic review	35 (21 COPD, 13 asthma, 1 both)	RCTs	Participants with COPD, asthma, or both.<br>COPD severity includes moderate to severe disease and severe to very severe disease.<br>Some studies required participants to have more than one exacerbation in the previous 12 months, signifying unstable or high-risk disease.<br><br>Asthma severity was divided into pediatric and adult.<br>Pediatric asthma studies included children with moderate to severe uncontrolled asthma, use of inhaled corticosteroids for at least 3 months and history of acute exacerbations.<br>Adult studies included impaired asthma-related health status and persistent asthma symptoms.<br><br>The mean age of participants ranged from 6 years (in pediatric asthma studies, 6 in total) to 73 years (in COPD populations).<br><br>The majority of the studies were conducted in Europe, other than in Oceania, Asia, and the Americas. Most trials were in high-income settings.<br><br>Gender distribution were not extracted.	Four main types of digital interventions:<br><br>**Real-time monitoring**<br>Use of digital devices at home to record physiological and symptom data, which was automatically transmitted to healthcare providers for review.<br>Features:<br>> Monitoring parameters: oxygen saturation, respiratory rate, PEF, spirometry, blood pressure, symptom scores.<br>> Devices: Bluetooth-enabled peripherals, integrated mobile apps, telehealth hubs.<br>> Feedback mechanisms: clinician alerts, patient dashboard updates, automated advice.<br><br>**Teleconsultations**<br>Structured video or phone consultations replacing or supplementing face-to-face visits.<br>Features<br>> Formats: video calls, telephone check-ins, mixed telehealth platforms with both monitoring and consultative components.<br>> Delivered by nurses, physicians, or pharmacists.<br>> Could be regular (e.g., monthly) or triggered by symptom alerts.<br>> Used for medication reviews, inhaler technique training, symptom assessment, or behavioral coaching.<br><br>**Electronic Patient Diaries**<br>Digital logs where patients entered self-reported data on symptoms, medication use, and triggers.<br>Features:<br>> Mostly asynchronous data entry (not real-time monitoring)<br>> Some included automated weekly prompts or ACT questionnaires<br>> Primarily aimed at self-management support, rather than direct clinical oversight<br><br>**Other**<br>> SMS reminders for medication adherence or vaccine prompts<br>> Educational videos/DVDs teaching breathing exercises or inhaler technique<br>> Web-based platforms providing asthma/COPD education or prompting symptom check-ins<br><br>**Duration of Interventions**<br>> Ranged from 2 weeks to 32 months<br>> Most common duration: 6–12 months<br>> Longer durations typically corresponded with more complex interventions or those including economic evaluations	Comparator group received usual care, which typically involved:<br><br>**Conventional In-person Care**<br>> Most studies used routine outpatient visits with general practitioners, pulmonologists, or asthma nurses as the control.<br>> This typically included regular clinic follow-ups, medication review, and physical examinations, based on clinical guidelines.<br><br>**No Digital Component**<br>> The control groups did not receive digital tools, telemonitoring, SMS, or web platforms.<br>> Patients relied solely on their in-person healthcare team for support and monitoring.<br><br>**Usual Self-Management**<br>Participants managed their condition using standard self-care strategies:<br>> Manual symptom tracking (e.g., paper diaries or none)<br>> Traditional medication routines without digital reminders<br>> No structured remote support or education<br><br>**Post-hospitalization Follow-up**<br>Some studies (particularly in COPD populations) had comparators who received standard post-exacerbation follow-up from hospital or primary care services, with no remote monitoring.<br>Others simply maintained existing care protocols without proactive outreach.<br><br>**Medication-only Support**<br>In some asthma studies, control groups received standard pharmacological treatment (e.g., inhaled corticosteroids or bronchodilators) without any behavioral or educational adjuncts.<br><br>**Minimal Contact**<br>A few studies described their comparator group as receiving "no additional intervention" or "minimal care," especially where the intervention involved intensive follow-up (e.g., weekly teleconsultations or daily symptom tracking).	**Primary Outcomes**<br>Economic metrics were the primary outcomes in most studies, particularly cost differences and cost-effectiveness of digital interventions compared to standard care.<br><br>Cost and Cost-Effectiveness:<br>Full economic evaluations (n = 11): ICERs ranged from €3,530.93 to €286,369.28 per QALY.<br>Partial economic evaluations (n = 24): Cost differences ranged from €0.12 to €85,217.86.<br>Several studies found digital health interventions to be less costly while maintaining or slightly reducing QALYs.<br><br>Direction of change: Generally favorable, with many interventions associated with lower overall healthcare costs and some improvements in health utility.<br><br>**Quality of Life Measures**<br>Reported in several studies using standardized health-related QoL instruments; changes varied depending on intervention type and disease group.<br><br>Tools used: EQ-5D, CAT, SGRQ:<br>EQ-5D and QALYs derived from it were common in full economic analyses.<br>CAT (COPD-specific): Used to track symptom severity and disease impact.<br>SGRQ (St. George's Respiratory Questionnaire): Used in selected COPD studies to assess symptom burden and activity limitation.<br><br>Direction of change: Mixed—some interventions led to increased QALYs and improved symptom scores, while others showed fewer QALYs despite cost savings.<br><br>**Medication Adherence**<br>Assessed either directly via smart devices or inferred through patient engagement metrics.<br><br>Measurement Approaches:<br>Smart inhaler sensors recorded ICS use in pediatric asthma trials.<br>Automated alerts and reminders were used to prompt medication use.<br>Some studies used self-report or digital diaries to estimate adherence patterns.<br><br>Direction of change: Improvements observed in studies with reminder systems and real-time adherence tracking; effect size not consistently reported.<br><br>**Disease Management Metrics**<br>Digital interventions aimed to reduce healthcare utilization and improve self-care behavior.<br><br>Healthcare Utilization:<br>Hospital admissions, ER visits, and unscheduled care were tracked in most studies.<br>Several studies reported reductions in hospital readmissions and emergency room usage, particularly among COPD patients.<br>Cost savings often reflected decreased acute care needs.<br><br>Behavioral and Self-Management Outcomes:<br>Some studies documented increased patient engagement in self-monitoring and education.<br>Interventions with feedback and coaching components promoted better disease awareness and timely action during symptom worsening.<br><br>Direction of change: Generally positive for utilization metrics and self-care engagement; variable effects on physical activity and lung function were not consistently reported.	**Cost-Effectiveness Metrics**<br>Statistical reporting focused heavily on economic analyses, particularly incremental cost-effectiveness ratios (ICERs) and direct cost comparisons between intervention and control groups.<br>ICERs:<br>Reported in 11 studies that conducted full economic evaluations.<br>Range: €3,530.93 to €286,369.28 per QALY.<br>In several cases, interventions were cost-saving but associated with slightly fewer QALYs.<br>Direction of change: Mixed; some interventions were cost-effective under commonly accepted willingness-to-pay thresholds, others were not.<br><br>**Cost Differences in Partial Analyses**<br>24 studies performed partial economic evaluations, focusing on cost comparisons without calculating QALYs or ICERs.<br>Cost Range and Variability:<br>The difference in total costs between intervention and control arms ranged from €0.12 to €85,217.86.<br>Interventions often resulted in fewer hospitalizations and outpatient visits, leading to lower costs.<br>Direction of change: Generally favorable for digital interventions, especially real-time monitoring and teleconsultation models.<br><br>**QALY Changes (Quality-Adjusted Life Years):**<br>Used in full economic evaluations to assess health-related utility gains or losses.<br>Reported Findings:<br>QALY gains ranged from 0.006 to 0.03 in some intervention groups.<br>In a few cases, QALYs were slightly lower in the intervention arm despite lower costs, indicating trade-offs in cost vs. health benefit.<br>One asthma study showed a 0.02 QALY gain with a DVD-based breathing intervention, deemed cost-effective.<br>Direction of change: Variable—several studies reported modest gains; others found lower QALYs despite economic advantages.<br><br>**Statistical Reporting Characteristics**<br>Confidence intervals and p-values:<br>> Not consistently reported across studies.<br>> Where ICERs and cost differences were calculated, uncertainty estimates (e.g., probabilistic sensitivity analysis) were rarely detailed in the main review.<br>Sensitivity analyses:<br>Not comprehensively summarized, though many full evaluations likely included them.<br>Limited information on statistical robustness or variability by subgroup.<br><br>**Summary of Direction of Effects**<br>> Real-time monitoring: Often cost-saving; some studies showed significant reductions in health resource use.<br>> Teleconsultations: Frequently favored in terms of cost and adherence.<br>> Electronic diaries and SMS: Mixed findings; small cost changes, inconsistent QALY effects.<br>> Overall trend: Interventions generally improved economic outcomes, but clinical impact (measured via QALYs or QoL) varied depending on the study design and digital tool complexity.	Not quantified via I^2 or Q-statistics, but substantial heterogeneity was clearly acknowledged and cited as the reason for not conducting a meta-analysis.<br><br>Sources:<br>> wide variation in digital intervention types (e.g., real-time monitoring, teleconsultations, SMS, apps)<br>> inclusion of both COPD- and asthma-specific studies with differing pathophysiology and care standards<br>> diverse populations ranging in age (6 to 73 years), disease severity (e.g., GOLD II–IV), and treatment settings<br>> differences in intervention duration (from 2 weeks to 32 months)<br>> inconsistent economic evaluation designs (full vs. partial analysis) and perspectives (healthcare, societal, unclear)<br>> geographic diversity (studies conducted in Europe, Asia, Oceania, and the Americas), with varying health system structures and resource availability	Digital health interventions were generally found to be economically favorable for managing asthma and COPD, especially when involving real-time monitoring and teleconsultations.<br><br>**Findings**:<br>> Most studies reported either cost savings or acceptable cost-effectiveness (based on ICER thresholds) for digital tools compared to standard care.<br>> Interventions commonly led to reduced healthcare utilization—fewer hospital admissions, emergency visits, and clinic appointments.<br>> Improvements in patient engagement, symptom monitoring, and self-care behaviors were frequently observed.<br>> Quality of life gains and medication adherence were reported in some studies, but these effects were less consistent and often modest.<br>> Full economic analyses supported the value of digital interventions more clearly than partial analyses, though both leaned positively.<br><br>The strongest economic and clinical benefits were associated with real-time physiological monitoring and structured teleconsultation models.<br>Simpler tools (e.g., SMS, electronic diaries) showed mixed results, with some positive trends in adherence and cost, but limited QoL impact.<br>Benefits were more pronounced in adult COPD populations with frequent exacerbations or recent hospitalizations.<br><br>Overall direction:<br>Digital interventions were found to be affordable, cost-saving, or cost-effective in most cases, suggesting strong potential for integration into chronic respiratory care. However, the variability in methods and reporting across studies calls for more standardized and robust future research.	> Predominance of partial analyses: Most studies reported only cost comparisons, limiting full cost-effectiveness assessment.<br>> Risk of bias: Many studies had unclear or high risk of bias, mainly due to missing data and inconsistent methodology.<br>> High heterogeneity: Wide variation in intervention types, outcome measures, and cost evaluation approaches reduced comparability.<br>> Geographic and temporal gaps: Most studies were from high-income countries and many were over 10 years old, limiting relevance to current technologies and low-resource settings.<br>> Short follow-up durations: Few studies assessed long-term effects or sustainability beyond 12 months.<br>> Limited reporting on adherence and QoL: Few studies used objective adherence measures or robust patient-centered outcomes.<br><br>Overall, while economic findings are promising, methodological inconsistencies and narrow geographic and temporal scope limit the strength and generalizability of conclusions.
The effects of telerehabilitation on physiological function and disease symptom for patients with chronic respiratory disease: a systematic review and meta-analysis	2024	Systematic review and meta-analysis	21	RCTs	Population with chronic respiratory diseases, including:<br>> COPD - the dominant condition across the studies. Most findings and conclusions were driven primarily by this condition<br>> Bronchiectasis<br>> Interstitial Lung Disease<br><br>The mean age across studies ranged approximately from mid-50s to mid-70s and they varied in gender composition, often with a predominantly male population.<br><br>Studies were conducted in diverse countries: Australia, China, Denmark, Spain, Norway, the United States, Canada, the UK, Switzerland, South Korea, and Greece.	A variety of telerehabilitation formats were used:<br><br>**Synchronous Video-Based Interventions**<br>Live video conferencing sessions with physiotherapists or rehab teams for real-time supervision and exercise guidance.<br><br>**Asynchronous or Automated Systems**<br>AI-supported Virtual Autonomous Physiotherapist Agent (VAPA) platforms provide personalized guidance and monitor patients.<br><br>**Telephone-Based Coaching and Health Mentoring**<br>Structured health coaching calls by nurses or peer mentors. Focus on goal setting, behavior change, and disease management support.<br><br>**Remote Monitoring Systems**<br>Participants used wearable or connected devices (e.g., pedometers, spirometers) to track: exercise sessions, oxygen saturation and respiratory rates.<br>Data was transmitted to healthcare teams for feedback and intervention adjustments.<br><br>**Home-Based Exercise Programs with Digital Support**<br>Exercise regimens performed at home, supported by instructional videos, written materials and mobile apps or digital diaries for self-monitoring.<br><br>**Key Components Across Interventions**<br>> Exercise Training: Aerobic and strength exercises tailored to patients' functional level.<br>> Education: Disease self-management, inhaler use, energy conservation techniques.<br>> Monitoring: Via telecommunication platforms or wearable devices.<br>> Feedback and Motivation: Real-time or delayed feedback from healthcare providers; behavioral nudging through calls or messages.<br>> Psychosocial Support: Addressing anxiety, depression, and promoting self-efficacy.<br><br>**Duration of Interventions**<br>Intervention periods varied considerably:<br>> Short-term programs: 8–12 weeks were common<br>> Medium to long-term programs: Up to 1–2 years in some studies	The comparator group received standard care. These were structured to reflect usual clinical practices in managing chronic respiratory diseases, particularly COPD.<br><br>**Conventional Pulmonary Rehabilitation**<br>Delivered in-person at hospitals or outpatient clinics. Included supervised aerobic and resistance training, breathing exercises, and education.<br><br>**Usual Care (Non-Rehabilitative)**<br>Some studies used non-rehabilitative standard medical care, such as:<br>> Routine follow-ups with primary care or specialists.<br>> Drug therapy management.<br>> No structured exercise or educational program.<br><br>**Educational or Supportive Contact**<br>In certain trials, comparators received:<br>> Educational booklets or advice on disease self-management.<br>> Social or check-in phone calls (non-interventional, non-clinical).<br>> No structured or personalized intervention beyond standard information provision.	**Primary Outcomes**<br>Telerehabilitation's effectiveness was primarily assessed through physical function and symptom severity indicators in COPD and other chronic respiratory disease populations.<br><br>Functional and Symptom Outcomes:<br>> 6-Minute Walk Distance (6MWD):<br>>> Short-term (≤ 6 months): MD = 7.52 meters (95% CI: 2.09 to 12.94); statistically significant but not clinically meaningful (MCID = 30m).<br>>> Long-term (> 6 months): MD = 12.89 (95% CI: -0.37 to 26.14); not statistically significant.<br><br>> Modified Medical Research Council Dyspnea Scale (mMRC):<br>>> Short-term: MD = -0.29 (95% CI: -0.41 to -0.18); statistically significant.<br>>> Long-term: MD = -0.38 (95% CI: -0.56 to -0.21); statistically significant.<br><br>Direction of change: Consistently favorable in the short term for both outcomes; mMRC remained significantly improved in the long term, unlike 6MWD.<br><br>**Quality of Life Measures**<br>Evaluated using COPD-specific instruments to assess perceived health status, symptom burden, and impact on daily living.<br><br>Tools Used: CAT and SGRQ:<br>> CAT (COPD Assessment Test):<br>>> Short-term: MD = -1.77 (95% CI: -3.52 to -0.02); significant.<br>>> Long-term: MD = -1.39 (95% CI: -3.83 to 1.05); not significant.<br><br>> SGRQ (St. George's Respiratory Questionnaire):<br>>> Activity Score:<br>>>> Short-term: MD = -1.71 (95% CI: -2.66 to -0.76); significant.<br>>>>Long-term: MD = -2.60 (95% CI: -6.00 to 0.80); not significant.<br><br>>> Impact Score:<br>>>> Short-term: MD = -1.26 (95% CI: -2.15 to -0.38); significant.<br>>>> Long-term: MD = -0.69 (95% CI: -3.09 to 1.70); not significant.<br><br>>> Symptom Score:<br>>>> Short-term: MD = -2.05 (95% CI: -3.05 to -1.05); significant.<br>>>> Long-term: MD = -1.66 (95% CI: -5.02 to 1.71); not significant.<br><br>Direction of change: Positive effects on quality of life in the short term across all domains; effects diminished over time and were no longer statistically significant beyond six months.<br><br>**Mental Health Measures**<br>Psychological well-being assessed through standardized anxiety and depression scales.<br>Tool Used: Hospital Anxiety and Depression Scale (HADS):<br>> Short-term (≤ 6 months): MD = -0.44 (95% CI: -0.86 to -0.03); statistically significant improvement.<br>> Long-term (> 6 months): MD = -0.21 (95% CI: -0.69 to 0.27); not significant.<br><br>Direction of change: Positive in the short term, indicating reduced anxiety and depression; long-term sustainability unclear due to reduced adherence and sample sizes.<br><br>Pulmonary Function Outcomes<br>Pulmonary function was evaluated to assess physiological improvement but did not yield significant effects.<br>Measurement Approaches:<br>> FEV1% predicted: MD = 2.19 (95% CI: -0.55 to 4.93); not significant.<br>> FEV1/FVC (%): MD = 2.26 (95% CI: -1.07 to 5.59); not significant.<br><br>Direction of change: Neutral. No consistent evidence of telerehabilitation improving lung function, consistent with prior meta-analyses on the topic.<br><br>**Disease Management Metrics**<br>Not directly assessed through objective healthcare utilization or adherence outcomes.<br>Behavioral and Self-Management Observations:<br>> Some included studies incorporated elements of remote coaching, peer mentoring, and educational reinforcement.<br>> Improvements in dyspnea, mental health, and self-reported QoL indirectly suggest better disease self-management.<br>> No standardized measures of medication adherence or hospital/ER utilization were reported across the included RCTs.<br><br>Direction of change: Indirect indicators (improved mMRC, HADS, and SGRQ scores) support enhanced self-management; however, lack of direct metrics limits definitive conclusions on disease management behavior and healthcare utilization.	Quantitative outcomes were reported across multiple domains, with most studies demonstrating short-term benefits of telerehabilitation over conventional care, although long-term effects were less consistent.<br><br>**Functional Capacity (6MWD)**<br>> Dai et al.: IG +7.52 m vs. CG; 95% CI: 2.09 to 12.94; P < 0.05 (≤6 months)<br>> Dai et al.: IG +12.89 m vs. CG; 95% CI: –0.37 to 26.14; P > 0.05 (>6 months)<br>> Trim-and-fill corrected estimate: MD = 5.836; 95% CI: 0.925 to 10.746; P = 0.020<br><br>**Dyspnea Severity (mMRC)**<br>> Dai et al.: IG –0.29 vs. CG; 95% CI: –0.41 to –0.18; P < 0.05 (≤6 months)<br>> Dai et al.: IG –0.38 vs. CG; 95% CI: –0.56 to –0.21; P < 0.05 (>6 months)<br><br>**Quality of Life (SGRQ and CAT)**<br>> SGRQ Activity Score: IG –1.71; 95% CI: –2.66 to –0.76; P < 0.05 (≤6 months)<br>> SGRQ Impact Score: IG –1.26; 95% CI: –2.15 to –0.38; P < 0.05 (≤6 months)<br>> SGRQ Symptom Score: IG –2.05; 95% CI: –3.05 to –1.05; P < 0.05 (≤6 months)<br>> CAT Score: IG –1.77; 95% CI: –3.52 to –0.02; P < 0.05 (≤6 months)<br>> CAT Score: IG –1.39; 95% CI: –3.83 to 1.05; P > 0.05 (>6 months)<br><br>**Mental Health (HADS)**<br>> Dai et al.: IG –0.44; 95% CI: –0.86 to –0.03; P < 0.05 (≤6 months)<br>> Dai et al.: IG –0.21; 95% CI: –0.69 to 0.27; P > 0.05 (>6 months)<br><br>**Pulmonary Function (FEV1%, FEV1/FVC)**<br>> FEV1%: MD = 2.19; 95% CI: –0.55 to 4.93; P > 0.05<br>> FEV1/FVC (%): MD = 2.26; 95% CI: –1.07 to 5.59; P > 0.05<br><br>**Publication Bias and Sensitivity Analysis**<br>> Egger's test for 6MWD: bias = 1.107; P = 0.019 → publication bias detected<br>> Sensitivity tests confirmed results remained stable when individual studies were excluded.	The heterogeneity was assessed using:<br>> I^2 statistic: Ranged from 0% (e.g., SGRQ, HADS) to 56% (CAT) — indicating low to moderate heterogeneity<br>> Chi^2 test: Used across analyses; not all values reported, but p-values guided model selection<br>> Model use: Fixed-effects applied when heterogeneity was low; random-effects used when I^2 ≥ 50% (e.g., CAT, FEV1%)<br><br>**Sources of Heterogeneity**<br>Subgroup analysis by follow-up duration (≤6 months vs. >6 months) was conducted to explore time-related effects.<br>Main contributors to heterogeneity included:<br><br>Intervention differences:<br>Wide variation in telerehabilitation formats — from video consultations to automated platforms — and durations ranging from 8 weeks to 2 years.<br><br>Outcome measurement variation:<br>QoL and symptom outcomes were measured using multiple tools (e.g., CAT, SGRQ, HADS), with some conceptual overlap but different scoring systems.<br><br>Population differences:<br>Studies included patients with varying disease types (mostly COPD, some ILD), age ranges, and international settings, adding variability in healthcare context and patient characteristics.<br><br>Publication bias:<br>Detected in 6MWD results (Egger's test p = 0.019); addressed using trim-and-fill adjustment, which confirmed the robustness of the pooled estimate.	Telerehabilitation is an effective short-term intervention for improving key health outcomes in patients with chronic respiratory diseases, particularly COPD.<br><br>**Effectiveness in the Short Term**<br>Telerehabilitation was found to significantly improve:<br>> Exercise capacity (e.g., 6MWD)<br>> Dyspnea symptoms (e.g., mMRC)<br>> Health-related quality of life (e.g., CAT, SGRQ)<br>> Psychological well-being (e.g., HADS)<br>These improvements were statistically significant across multiple outcomes, suggesting that remote, digitally supported rehabilitation is a viable and beneficial alternative to conventional center-based programs, especially for patients who face logistical barriers to in-person care.<br><br>**Limited Long-Term Effects**<br>While some benefits, particularly for dyspnea (mMRC), persisted beyond six months, most long-term effects were not statistically significant. The authors attributed this to factors such as:<br>> Reduced patient adherence over time<br>> Smaller follow-up samples<br>> Diminished engagement with digital platforms<br>They emphasized the need for more high-quality, long-term trials to clarify the sustained impact of telerehabilitation.<br><br>**Clinical Relevance and Cautions**<br>Although some outcomes (like 6MWD) showed statistically significant changes, the actual magnitude of improvement (e.g., +7.52m in 6MWD) did not reach clinically meaningful thresholds, calling for cautious interpretation when applying findings to practice.<br><br>**Overall**<br>Telerehabilitation offers a promising, scalable approach to managing COPD and similar conditions, improving symptoms, quality of life, and mental health in the short term. However, its long-term efficacy, cost-effectiveness, and implementation feasibility require further investigation. The review supports integrating digital rehabilitation tools into routine care but underlines the importance of personalized design, patient training, and follow-up continuity.	**Study and Population Variability**<br>> Included studies featured diverse patient groups with differences in age, disease type, and baseline severity.<br>> This variability contributed to inconsistent results and reduced comparability across trials.<br><br>**Methodological Concerns**<br>Many studies lacked clear reporting on:<br>> Allocation concealment<br>> Blinding of participants and outcome assessors<br>This introduced risks of selection, performance, and detection bias, especially for self-reported outcomes.<br><br>**Limited Long-Term Evidence**<br>> Most studies focused on short-term outcomes (≤6 months).<br>> Longer-term trials were few and often showed: weaker effects, lower patient adherence, higher attrition rates<br><br>**Intervention Heterogeneity**<br>Telerehabilitation programs differed in:<br>> Format (e.g., video coaching, automated systems)<br>> Duration (from 8 weeks to 2 years)<br>> Components (exercise, education, monitoring, mental health support)<br>These differences reduced consistency across studies.<br><br>**Lack of Economic Evaluation**<br>No included studies assessed: cost-effectiveness and healthcare resource use<br>This limits understanding of telerehabilitation's practical and financial feasibility.<br><br>**Generalizability Issues**<br>Studies were conducted in multiple countries with varying:<br>> Digital infrastructure<br>> Patient access to technology<br>> Healthcare delivery models<br>These factors limit the broader applicability of findings.

Mapping of digital health interventions for the self-management of COPD: A systematic review	2023	Systematic review	32	RCTs (23), quasi-experimental (4), observational/cohort (4), qualitative (1)	Patients with COPD and no restriction on COPD severity stage but in a stable condition.<br>The majority of studies included adults over 40 years with both male and female participants.<br><br>Most studies were from high-income countries and few from low and middle income countries: UK, USA, Netherlands, South Korea, Canada, Spain, China, Taiwan, Sweden, Romania, Denmark, Indonesia, Germany, Hong Kong and multi-country (Germany, Austria, Switzerland).	The digital interventions can be categorized into:<br><br>**Mobile-based Interventions:**<br>> Mobile apps<br>> Smartphone-enabled tools<br>> Text messaging systems<br>> Phone-based monitoring<br><br>**Web-based Platforms / Portals**<br>> Online educational and monitoring platforms<br>> Browser-accessed tools for diary and symptom tracking<br><br>**Tablet-based Interventions**<br>> Custom apps or rehab programs delivered via tablet<br><br>**Key components of interventions:**<br>Remote Patient Monitoring<br>> Symptom tracking: oxygen saturation, blood pressure, self-reports<br>> Exercise and symptom diaries<br><br>COPD Education<br>> Delivered via videos, online modules, or interactive sessions<br><br>Pulmonary Rehabilitation<br>> Guided physical exercises, breathing techniques (e.g., pursed-lip breathing)<br><br>Medication Management<br>> Adherence tracking<br>> Support for bronchodilator/steroid use during exacerbations<br><br>Exacerbation Management<br>> Action plans for early intervention<br>> Environmental control advice<br><br>Counseling and Behavioral Support<br>> Smoking cessation<br>> Dietary advice<br>> Anxiety and depression management<br><br>**Duration**<br>Range: 20 days to 2 years<br><br>Most common durations:<br>> 6 months<br>> 12 months<br>> Others: 4 weeks, 90 days, 9 months, etc.	**Comparator identified**<br>> Usual care: standard medication without any digital component.<br>> Waitlist Controls: participants were placed on a waitlist to receive the digital intervention later. Served as a temporary control group for assessing short-term effects.<br>> No Intervention / No Comparison Group: Some observational or feasibility studies lacked a formal comparator. These studies focused solely on assessing feasibility, usability, or adherence within a single group receiving the intervention.<br><br>**Type of usual care interventions**<br>> Routine clinical care: regular check-ups, medication management, and symptom monitoring by healthcare providers.<br>> In-person pulmonary rehabilitation: supervised exercise and education programs delivered face-to-face.<br>> Printed educational materials: booklets or handouts covering COPD management, inhaler use, and lifestyle advice.<br>> Non-digital follow-ups: standard nurse calls or clinic visits without digital tools.<br>> No additional intervention: some groups continued with existing care routines, without any enhancement.	**Primary outcomes** focused on reducing the frequency and severity of COPD exacerbations, lowering hospital admissions and emergency room visits, and enhancing self-management behaviors such as symptom tracking and adherence to action plans. Many interventions showed positive trends in reducing acute care utilization by enabling earlier detection and response to worsening symptoms.<br><br>**Quality of Life** was another core focus, evaluated using validated tools such as the SGRQ, CAT, CRQ, CCQ, and EQ-5D. Most studies reported moderate improvements in QoL, with patients experiencing better control over symptoms, enhanced functional capacity, and improved well-being. These gains were often linked to interventions that integrated education, activity tracking, and feedback loops.<br><br>**Medication adherence** improved in several studies through tools such as digital diaries, reminders, and monitoring of inhaler use. Participants demonstrated better consistency in medication intake and reduced inhaler errors, alongside increased confidence in managing their treatment regimen.<br><br>**Disease management metrics** included daily physical activity (e.g., step count), exercise capacity (e.g., 6MWT, ISWT), lung function (FEV_1, FVC), muscle strength, fatigue, and self-care behaviors like smoking cessation. Many of these metrics showed improvements, supporting the role of digital interventions in helping patients maintain clinical stability and engage in healthier lifestyles.<br><br>**Measurement instruments** used across studies included: SGRQ, CAT, EQ-5D, CRQ, CCQ, 6MWT, ISWT, ESWT, HADS, PHQ-9, GAD-7, BCKQ, PRAISE, and various self-efficacy and satisfaction scales. These allowed for both objective assessments and insights into patients' experiences and perceived benefits.<br><br>**Overall**, the review found that digital interventions delivered via mobile apps, web platforms, or tablets offered clinically meaningful benefits across multiple domains of COPD management.	The review presented a narrative synthesis rather than pooled statistical estimates.<br>Many individual studies reported statistically significant improvements in outcomes such as exacerbation rates, physical activity, quality of life (e.g., CAT, SGRQ), and adherence. However, the statistical findings were not uniformly reported across all studies.<br>The variation in study design, intervention components, and measurement tools limited comparability and precluded meta-analysis.<br><br>Overall, the direction of effect was generally positive, suggesting digital health interventions offer beneficial impacts for COPD self-management.	Not quantitatively assessed with I^2 or Q statistics.<br><br>The authors explicitly acknowledged substantial heterogeneity across studies in terms of:<br>> Intervention type: differences in digital intervention, duration, frequency, and intensity<br>> Population characteristics; variation in age, digital literacy, disease severity, and standard care settings<br>> Outcome measures: inconsistent use of tools to measure QoL, physical activity, and self-management<br>> Study designs: mix of RCTs, quasi-experimental studies, observational cohorts, and qualitative designs<br><br>These differences limited comparability and were cited as the main reason for using narrative synthesis.<br>No subgroup or sensitivity analyses were conducted to further explore heterogeneity.	Digital health interventions are a promising and increasingly relevant approach for supporting the self-management of COPD, especially in home and community settings. These interventions, particularly those delivered via mobile phones, apps, and web platforms, were associated with improvements in:<br><br>> Patient engagement<br>> Adherence to medication and self-management plans<br>> Reduction in COPD exacerbations and healthcare utilization<br>> Quality of life<br>> Physical activity and disease knowledge<br><br>While these benefits were consistently observed across many studies, the review emphasized that:<br>> Most of the evidence comes from high-income countries, where infrastructure and digital literacy are higher.<br>> There is a critical need for research in low- and middle-income countries to assess implementation feasibility, usability, cultural relevance, and long-term impact in resource-constrained settings.<br><br>The review supports the development of smartphone-enabled home-based COPD self-management platforms, particularly in low- and middle-income countries like India, where the COPD burden is high but digital health is still emerging.	**Protocol Deviations**<br>Although the review protocol was developed a priori, there were notable deviations:<br>> The team did not use the WHO's formal classification of digital health interventions (DHI v1.0), as a new version was pending release.<br>> The review protocol was not registered in PROSPERO, which may affect transparency and replicability.<br><br>**Lack of Meta-Analysis**<br>Due to substantial heterogeneity in interventions, populations, and outcomes, the authors chose not to conduct a meta-analysis.<br>This precluded formal assessment of effect sizes and statistical heterogeneity (e.g., I^2 values).<br><br>**Methodological Variability**<br>The included studies had diverse designs: RCTs, quasi-experimental, observational, and qualitative.<br><br>**Variability in study quality was noted**<br>> Several studies lacked blinding of participants or assessors<br>> Some studies did not provide sufficient detail on their procedures, leading to unclear risk of bias.<br><br>**Incomplete Reporting on Participant Characteristics**<br>Many studies did not report the literacy or digital literacy of participants, which is crucial when evaluating digital interventions.<br>In some cases, gender, age distribution, or disease severity was also incompletely described.<br><br>**Geographic Skew**<br>The majority of studies were conducted in high-income countries.<br>There is a lack of data from low- and middle-income countries, limiting the generalizability of findings to settings with different digital infrastructure and healthcare systems.<br><br>**Limited Focus on Data Privacy and Security**<br>None of the included studies explicitly addressed issues related to data protection, privacy, or confidentiality, which are especially important in digital health.<br><br>**Short-Term Follow-Up**<br>Many studies had short intervention periods (as brief as 20 days), limiting insight into long-term sustainability and outcomes.
Characteristics, Components, and Efficacy of Telerehabilitation Approaches for People with Chronic Obstructive Pulmonary Disease: A Systematic Review and Meta-Analysis	2022	Systematic review and meta-analysis	22 (14 in meta-analysis)	RCTs	Participants with diagnosed COPD with mild to moderate disease severity.<br><br>> Gender distribution: 59% male<br>> Age range: adults over 40, with the mean age ranging from 62 to 75 years old.<br><br>> Geographic Distribution: studies conducted in developed countries: UK, Spain, Denmark, Netherlands, Greece, China, Thailand, South Korea, Japan, Australia, and North America.	Focus only on telerehabilitation delivered in:<br>**Asynchronous modality**: Most common<br>> Interaction not in real time<br>> Patients performed activities independently, clinicians monitored remotely<br>> Required more complex infrastructure (e.g., apps, wearable devices, web portals)<br><br>**Synchronous modality**<br>> Real-time sessions via video conferencing<br>> Mimicked in-clinic rehabilitation sessions<br><br>Most interventions were multicomponent with a combination of the following:<br>> Exercise training: Aerobic, resistance, strength, inspiratory muscle exercises<br>> Education: COPD self-management, disease understanding, health behavior coaching<br>> Monitoring: Through pedometers, actigraphs, heart rate monitors, or online platforms<br>> Feedback: Online (real-time or platform-based), offline, or via app messages<br>> Decision support: Dynamic adjustment of intensity/frequency by clinicians based on performance<br>> Psychological support<br><br>**Duration**<br>Total program duration: 6 to 52 weeks<br>> Most common durations: 8–12 weeks<br>> Two studies examined 52-week maintenance programs<br><br>**Technologies**<br>> Platforms: WeChat, TELECARE, Pulmonary Explorer, Virtual Agent, Fitbug<br>> Devices: Smartphones, tablets, heart rate monitors, pulse oximeters, pedometers<br>> Digital content: Educational videos, interactive web content, serious games<br>> Communication tools: Messaging apps, phone calls, teleconferencing	**No intervention**<br>This group received no structured rehabilitation or exercise training during the study period. They represent the usual care or "wait-list" condition in many trials.<br><br>Features:<br>> No active rehabilitation program<br>> May have continued standard clinical follow-up (e.g., medication adjustments)<br>> Used as a reference group to evaluate the absolute effectiveness of telerehabilitation<br><br>**Conventional Intervention**<br>This group received traditional, in-person pulmonary rehabilitation, delivered in clinics, hospitals, or outpatient centers.<br><br>Features:<br>> Structured, face-to-face programs<br>> Matched in intensity and duration to the telerehabilitation arm<br>> Often included exercise sessions, education, and supervision by healthcare professionals	**Primary outcomes** across all studies were functional capacity, dyspnea, and quality of life<br><br>**Quality of life measure**s were frequently assessed using disease-specific and generic tools<br>> Disease-specific: SGRQ, CAT, CRQ, CCQ<br>> Generic: EQ-5D, SF-36, IADL<br><br>Functional capacity assessed through standardized walk tests and activity tracking<br>> Walk tests: 6-minute walk distance (6MWD), Endurance Shuttle Walk Test, Incremental Shuttle Walk Test<br>> Activity tracking: Actigraph, pedometer, Physical Activity Level measures<br><br>Dyspnea commonly evaluated with patient-reported scales and inspiratory effort metrics<br>> PROMs: Modified Medical Research Council Dyspnea Scale (mMRC), San Diego SOBQ<br>> Physiological: Pimax (max inspiratory pressure), spirometry (FEV_1, FVC)<br><br>**Disease management outcomes** included adherence, safety, and self-efficacy<br>> Adherence: % sessions completed, total session counts, engagement time<br>> Safety: adverse events, hospitalization rates, ED visits<br>> Self-management: PRAISE scale, COPD Self-Care Inventory, Exercise Self-Regulatory Efficacy<br><br>Mood and psychosocial outcomes assessed in several studies using validated instruments<br>> Mood: HADS, Beck Depression Inventory, STAI, GAD-7, POMS<br>> Social support: MOS Social Support Survey<br><br>**Medication adherence**: note evaluated<br><br>**Direction/magnitude of change**:<br>> Functional capacity (TR vs NI): SMD = 0.29 (95% CI: 0.08–0.49), p = 0.001<br>> Functional capacity (TR vs CI): SMD = 0.12 (95% CI: –0.23 to 0.46), not significant<br>> Dyspnea (TR vs NI): SMD = 0.76 (95% CI: 0.15–1.37), p = 0.01<br>> Dyspnea (TR vs CI): SMD = 0.27 (95% CI: 0.08–0.47), p = 0.001<br>> Quality of life (TR vs NI): SMD = 0.57 (95% CI: 0.15–1.00), p = 0.001<br>> Quality of life (TR vs CI): SMD = 0.05 (95% CI: –0.28 to 0.39), not significant<br><br>Enhanced effects observed with asynchronous TR models, particularly for:<br>> Functional capacity: g = 0.39 vs NI<br>> Dyspnea: g = 0.82 vs NI<br>> Quality of life: g = 0.60 vs NI	Meta-analysis showed a significant improvement in key COPD outcomes among patients receiving telerehabilitation compared to controls (Functional capacity: SMD = 0.29, 95% CI: 0.08–0.49; Dyspnea: SMD = 0.76, 95% CI: 0.15–1.37; Quality of life: SMD = 0.57, 95% CI: 0.15–1.00). Subgroup analyses demonstrated larger effects when interventions were delivered asynchronously (Functional capacity: SMD = 0.39; Dyspnea: SMD = 0.82; Quality of life: SMD = 0.60), compared to synchronous models (Dyspnea: SMD = 0.48, not significant; QoL: SMD = 0.15, not significant). Comparisons with conventional rehabilitation showed non-inferiority or modest effects (Dyspnea: SMD = 0.27, 95% CI: 0.08–0.47; Functional capacity: SMD = 0.12, NS; QoL: SMD = 0.05, NS).<br><br>Publication bias was examined using funnel plots and addressed through trim-and-fill procedures. Missing studies were imputed in several analyses, but findings remained statistically significant.	Heterogeneity was formally evaluated in all meta-analyses using:<br>> I^2 statistic<br>> Cochran's Q test<br><br>**Heterogeneity by Outcome**<br>Functional Capacity<br>> TR vs No Intervention (NI): I^2 = 52% → moderate heterogeneity<br>> TR vs Conventional Intervention (CI): I^2 = 0% → no heterogeneity<br>> Asynchronous TR vs NI: I^2 = 0% → no heterogeneity<br><br>Dyspnea<br>> TR vs NI: I^2 = 67.11% → moderate to high<br>> TR vs CI: I^2 = 0% → no heterogeneity<br>> Asynchronous TR vs NI: I^2 = 77.59% → high<br>> Synchronous TR vs NI: single study → I^2 not applicable<br><br>Quality of Life<br>> TR vs NI: I^2 = 68.21% → moderate to high<br>> TR vs CI: I^2 = 42.96% → low to moderate<br>> Asynchronous TR vs NI: I^2 = 54.74% → moderate<br>> Synchronous TR vs NI: I^2 = 17.08% → low<br><br>**Sources and Exploration of Heterogeneity**<br>Subgroup analyses were performed to explore potential sources of heterogeneity, focusing on:<br>> TR delivery model (asynchronous vs synchronous)<br>> Comparator type (no intervention vs conventional care)<br>> Intervention characteristics (e.g., feedback frequency, duration, technological sophistication)<br><br>Key insights:<br>> Asynchronous TR produced more stable and favorable outcomes with minimal heterogeneity in functional capacity<br>> Variability in dyspnea and QoL outcomes may reflect differences in outcome measurement, intervention complexity, and implementation strategies<br><br>**Publication Bias and Its Relationship to Heterogeneity**<br>> Funnel plots revealed asymmetry in several outcomes, particularly for QoL and dyspnea<br>> The trim-and-fill method was applied to adjust for potential publication bias<br>> Adjusted results remained statistically robust, supporting the reliability of the findings despite initial bias indicators<br><br>**Overall**<br>Heterogeneity was generally greater in comparisons with no intervention (Functional capacity: I^2 = 52%; Dyspnea: I^2 = 67.11%; QoL: I^2 = 68.21), reflecting broader variability in control conditions. In contrast, comparisons with conventional care showed low to negligible heterogeneity (e.g., Functional capacity and Dyspnea: I^2 = 0%). Subgroup analyses—particularly by TR model—successfully accounted for much of the observed variability.	Telerehabilitation (TR) is an effective and safe alternative to traditional in-person pulmonary rehabilitation for people with COPD, particularly those in mild to moderate severity.<br>> TR significantly improves functional capacity, dyspnea, and quality of life compared to no intervention<br>> TR is non-inferior to conventional rehabilitation and even superior for dyspnea relief<br><br>Asynchronous TR models were especially effective, offering greater scalability and flexibility<br>> Demonstrated stronger and more consistent effects on all outcomes<br>> Associated with lower heterogeneity in functional capacity outcomes<br><br>Adherence to TR was high in the majority of studies that reported it<br>> Several trials reported adherence rates above 80%, reinforcing its feasibility in home settings<br><br>Safety of TR was generally well-supported<br>> Most studies reported either no adverse events or minimal incidents<br>> Only one study reported notable safety issues (27% with events, 5 hospitalizations)<br><br>Comprehensive and tailored TR programs hold promise<br>> Multidimensional approaches (including education, self-management, exercise) showed stronger benefits<br>> However, many TR programs were still unimodal (exercise-only), indicating room for improvement<br><br>Digital health can expand access to rehabilitation, especially for early-stage patients<br>> TR addresses barriers such as transportation, cost, and clinical availability<br>> It supports continuity of care and early intervention strategies	**Heterogeneity of interventions and outcomes**<br>> Considerable variation existed across studies in terms of TR duration, frequency, intensity, components, and technological complexity<br>> Differences in outcome measures and tools (e.g., multiple scales for dyspnea and QoL) limited comparability across trials<br>> This heterogeneity complicates the interpretation and pooling of results, particularly in comparisons with no intervention<br><br>**Variability in methodological quality**<br>> 4 of the 22 included RCTs were rated low quality (TESTEX score ≤6)<br>> Common issues included lack of blinding, absence of intention-to-treat analysis, and incomplete reporting of adherence and safety metrics<br><br>**Inconsistent or incomplete reporting of patient-relevant outcomes**<br>> Only a subset of studies reported on adherence to telerehabilitation (8/22) and safety/adverse events (7/22)<br>> Medication adherence was not reported in any study<br>> This limits the ability to evaluate TR's full impact on disease management<br><br>**Short-term follow-up**<br>> Most interventions focused on immediate post-treatment outcomes<br>> Only two trials assessed long-term maintenance effects (e.g., over 12 months)<br>> Thus, the sustainability of TR benefits remains largely untested<br><br>**Lack of standardization in TR design**<br>> No consensus exists on the core components required for telerehabilitation to be effective (e.g., feedback mechanisms, decision support, platform type)<br>> The definition of TR itself varied across studies, with some equating it to any home-based program, even without dynamic clinical oversight<br><br>**Publication bias and missing data**<br>> Funnel plot asymmetry and trim-and-fill analyses indicated possible publication bias in key outcomes (especially dyspnea and quality of life)<br>> Although adjusted analyses remained significant, bias cannot be entirely ruled out
Effectiveness of internet-based self-management interventions on pulmonary function in patients with chronic obstructive pulmonary disease: A systematic review and meta-analysis	2023	Systematic review and meta-analysis	8	RCTs	Participants diagnosed with COPD with different levels of severity:<br>> 4 studies with moderate to very severe<br>> 1 study with mild to severe<br>> 3 studies did not report specific severity stages<br><br>Mean age of the participants: 62.7 to 73.5 years across studies<br>Gender distribution is predominantly male<br><br>Studies were conducted in China, Spain, and Germany.<br>Dominance of Chinese patient populations, raising a potential regional bias in applicability.	Internet-based self-management programs delivered with:<br>> Online platforms/web portals<br>> Mobile apps<br>> WeChat-based platforms<br>> Web-based call center (telephone + web system)<br>> Electronic Health Records-linked coaching systems<br><br>Most interventions were **multicomponent** and included combinations of the following:<br><br>**Health education**:<br>> Information about COPD, medications, inhalation techniques, pulmonary rehabilitation, smoking cessation, oxygen therapy, nutrition, and mental health.<br>**Self-monitoring**:<br>> Tracking symptoms, oxygen saturation, medication intake, physical activity, and mood.<br>**Reminders and alerts**:<br>> Medication and exercise reminders, breathing exercises, and exacerbation warning signs.<br>**Feedback and support**:<br>> Real-time or asynchronous interaction with healthcare providers (nurses, educators, administrators).<br>**Peer support and group interaction**:<br>> Noted especially in WeChat-based groups, facilitating communication among patients.<br>**Symptom surveillance and early warning**:<br>> Identification of acute exacerbations, triage advice, and follow-up alerts.<br><br>**Duration**<br>Varied across studies:<br>> Shortest duration: 2 months<br>> Longest duration: 12 months<br>> Others: 3, 6, or 9 months	Comparator groups received usual care or non-digital self-management interventions.<br><br>**Usual Care** was the most common comparator across studies (used in 5 of 8).<br>This included routine clinical visits, general medication prescriptions, and occasional verbal advice.<br><br>**Non-digital Self-Management** was present in 2 studies, where participants received education and care instructions typically delivered face-to-face or in print, with no interactive tools or behavioral prompts.<br><br>**Paper-Based Self-Management Materials**, in which one study offered a printed COPD manual with guidance on symptom management, medication, and pulmonary rehab exercises.	**Primary outcomes** across all studies were pulmonary function measures<br>Pulmonary function assessed using spirometry-based indicators<br>> FEV1 (% predicted): reported in 5 studies; no significant improvement (SMD = 0.17; 95% CI: –0.01 to 0.35; p = .06)<br>> FEV1 (Liters): reported in 3 studies; not statistically significant (SMD = 0.93; 95% CI: –0.25 to 2.11; p = .12)<br>> FEV1/FVC (%): reported in 5 studies; no significant difference (MD = 3.55; 95% CI: –1.41 to 8.50; p = .16)<br>> FVC (Liters): reported in 1 study; significantly improved in the intervention group (p < .05)<br>> FVC (% predicted): reported in 2 studies; no significant improvement (MD = 0.67; 95% CI: –14.43 to 15.76; p = .93)<br><br>**Quality of life measures** and **Medication Adherence** were not assessed or reported in the included studies<br><br>**Disease management** was not systematically reported, but several interventions included self-monitoring of symptoms, exercise tracking, and communication with healthcare providers<br><br>**Direction/magnitude of change**:<br>> FEV1 (%): small, non-significant effect (SMD = 0.17; p = .06)<br>> FEV1 (L): large, non-significant trend toward benefit (SMD = 0.93; p = .12)<br>> FEV1/FVC (%): small, non-significant increase (MD = 3.55; p = .16)<br>> FVC (L): significant improvement in one study only (p < .05)<br>> FVC (%): no meaningful change (MD = 0.67; p = .93)	FEV1 (% predicted)<br>Effect estimate: SMD = 0.17<br>95% CI: [–0.01, 0.35]<br>p-value: .06<br>Interpretation: Small, non-significant improvement<br><br>FEV1 (Liters)<br>Effect estimate: SMD = 0.93<br>95% CI: [–0.25, 2.11]<br>p-value: .12<br>Interpretation: Trend toward benefit, not statistically significant<br><br>FEV1/FVC (%)<br>Effect estimate: MD = 3.55<br>95% CI: [–1.41, 8.50]<br>p-value: .16<br>Interpretation: No significant effect<br><br>FVC (Liters)<br>Effect estimate: Not pooled<br>p-value: < .05 (reported from Zhao et al., 2022)<br>Interpretation: Significant improvement, but limited generalizability due to a single study<br><br>FVC (% predicted)<br>Effect estimate: MD = 0.67<br>95% CI: [–14.43, 15.76]<br>p-value: .93<br>Interpretation: No meaningful change	Assessed with<br>> I^2 Statistic<br>> Q Test (Chi-square), but mentioned in methodology, but specific Q values not reported and used alongside I^2 for evaluating heterogeneity in effect estimates<br><br>Model Used<br>Random-Effects Model (DerSimonian & Laird method) applied in all meta-analyses.<br>Chosen due to expected variability in study characteristics (e.g., population, intervention types, duration)<br><br>**Sources of Heterogeneity**<br>Potential contributors to heterogeneity included:<br>> Variation in intervention platforms (mobile apps, WeChat, EHR, telemonitoring)<br>> Different COPD severity levels among participants<br>> Diverse geographical and healthcare contexts (mostly China, some Europe)<br>> Duration of interventions (ranged from 2 to 12 months)<br>> Study design rigor and risk of bias<br><br>Subgroup Analyses for Heterogeneity Exploration<br>Conducted based on intervention duration:<br>> less than 3 months<br>> 3–6 months<br>> 6–12 months<br><br>Result:<br>No subgroup showed statistically significant improvement, suggesting duration alone did not explain the variability in outcomes.<br><br>**Overall**<br>Heterogeneity was expected and appropriately handled with random-effects models.<br><br>It likely reflects real clinical and methodological diversity rather than mere statistical noise.<br><br>The authors did not identify any dominant source of heterogeneity from subgroup analyses, indicating the variability was multifactorial and diffuse.<br><br>Let me know if you'd like a structured summary of this for data extraction or inclusion in your umbrella review table.	**Effectiveness of the Intervention**<br>Internet-based self-management interventions showed a significant effect on FVC (L) in one study.<br><br>> For other pulmonary function indicators (FEV1 %, FEV1 L, FEV1/FVC %, FVC %), no statistically significant improvements were found across studies.<br><br>This suggests that internet-based interventions may support pulmonary function improvement, but the effect is limited and not consistently demonstrated.<br><br>**Caution in Interpretation**<br>Authors stress that findings should be interpreted cautiously due to:<br>> Limited number of studies using pulmonary function as a primary outcome<br>> Small sample sizes and high variability in study designs<br>> Risk of bias in several studies (e.g., unclear randomization, lack of blinding)<br>> Short follow-up durations in most studies, whereas lung function decline is a long-term process<br>> Regional concentration (predominantly Chinese studies) limits generalizability<br><br>**Clinical Implications**<br>Internet-based interventions provide a promising alternative for patients who face barriers to face-to-face care, especially in contexts like the COVID-19 pandemic.<br>They can enhance accessibility and continuity of care for COPD management through remote education, monitoring, and support.<br><br>**Research Implications**<br>More high-quality RCTs are needed with:<br>> Larger sample sizes<br>> Longer intervention/follow-up durations (≥12 months)<br>> Standardized intervention components and comparator definitions<br>> Broader geographic representation<br><br>Future studies should explore other outcomes such as quality of life, medication adherence, and exacerbation rates to fully assess intervention value.<br><br>**Overall**<br>The review concludes that internet-based self-management interventions have potential, particularly for improving FVC (L), but the current evidence is inconclusive and limited in strength. Further research is essential to clarify their effectiveness and generalizability in COPD care.	**Protocol Not Registered**<br>The study protocol was not pre-registered in PROSPERO or any systematic review registry.<br>This increases the risk of selective outcome reporting or analytical flexibility and reduces transparency.<br><br>**Methodological Weaknesses in Included Studies**<br>Several trials had unclear or high risk of bias in key domains:<br>> Random sequence generation<br>> Allocation concealment<br>> Blinding (performance and detection bias)<br>> Attrition bias (differential dropout rates)<br>These issues compromise internal validity and reduce confidence in the pooled results.<br><br>**Limited Outcome Scope**<br>The review focused only on pulmonary function measures, excluding other clinically relevant outcomes such as:<br>> Quality of life<br>> Exacerbation frequency<br>> Hospital admissions<br>> Medication adherence<br>This narrow focus limits the comprehensiveness of the intervention evaluation.<br><br>**Small Number of Eligible Studies**<br>Only 8 studies were included in the meta-analysis, and some outcomes were reported by just 1 or 2 trials, which:<br>> Limits statistical power<br>> Prevents meaningful subgroup analyses for some outcomes<br>> Increases vulnerability to random error and publication bias<br><br>**Regional and Language Bias**<br>5 of 8 studies were conducted in China, and only studies published in English or Chinese were included.<br>This introduces potential geographic and cultural bias, limiting the global generalizability of findings.<br><br>**Short Follow-up Durations**<br>Most included studies had intervention durations of ≤12 months.<br>Given that pulmonary function decline occurs gradually over the years, short-term follow-up may fail to capture meaningful changes.
Effectiveness of Smartphone Devices in Promoting Physical Activity and Exercise in Patients with Chronic Obstructive Pulmonary Disease: A Systematic Review	2017	Systematic review	8	RCTs (5), non-RCTs (3)	Participants diagnosed with COPD were classified as having mild to moderate severity.<br><br>The mean age across studies ranged from 62 to 72 years.<br><br>Across studies, male participants were predominant. Overall, male participation ranged from 65% to 100%, depending on the study.<br><br>Detailed country-level geographical distribution data is not exhaustively provided in the review.	Smartphone-based platforms to promote physical activity (PA) and to control exercise programs. Two main types.<br><br>**Physical Activity Promotion via Smartphone Apps**<br>> Included step count monitoring, real-time feedback, and motivational messaging.<br>> Used integrated or external tri-axial accelerometers for activity tracking.<br><br>**Exercise Training via Music-Driven Programs**<br>> Smartphones used to play music paced to exercise intensity (80% of estimated VO_2max) during walking.<br>> Duration and intensity were recorded and adapted through the smartphone.<br><br>**Key components**<br>Monitoring:<br>> Devices tracked steps/day, steps/min, or walking session duration.<br>> Some used external sensors connected via Bluetooth®.<br><br>Feedback:<br>> Real-time visual feedback on the screen.<br>> Feedback frequency varied from daily to weekly.<br><br>Goal Setting:<br>Activity goals were either:<br>> Personalized (e.g., +20% of baseline),<br>> Or fixed (e.g., 50% of the physical activity level of healthy individuals).<br><br>Motivational Messaging:<br>> Text messages to encourage compliance and improve adherence.<br><br>Supervision and Adjustments:<br>> In music-driven programs, exercise intensity was adjusted every 4 weeks based on ISWT results.<br><br>Devices Used:<br>> Varied smartphones, e.g., HTC, Samsung Galaxy, LG Optimus, Motorola Droid.<br>> Some interventions used custom-built apps (e.g., MoveSense, eHealth).<br><br>**Duration of Interventions**<br>Ranged from 4 weeks to 12 months.<br><br>Example durations:<br>4 weeks: Short-term feedback interventions<br>6 months: Walking programs using music pacing<br>12 months: Longest program included supervised and self-managed phases	The comparator across the included studies was typically standard care, which consisted of the following components:<br><br>> **Routine COPD management**: Patients continued with their usual medical treatments, including physiotherapy and medications.<br><br>> **Verbal instructions** only: In most studies, participants in the control groups were advised verbally to maintain or increase daily walking but were not given structured exercise programs or behavioral interventions.<br><br>> **No use of digital tools**: Control groups did not use smartphones, apps, or any wearable technology for tracking or feedback.<br><br>> **No goal setting or motivational support**: Unlike intervention groups, controls received no personalized step goals, real-time feedback, or motivational text messages.<br><br>> **No structured or monitored exercise**: In trials using music-paced exercise interventions via smartphone, controls were asked to walk at home without guidance, feedback, or intensity prescriptions.	**Primary Outcomes**: Physical activity (PA):<br>Measured as steps/day or steps/minute using smartphone-integrated or Bluetooth-connected accelerometers.<br>Mixed results:<br>> One study (Tabak et al.) reported a 13% increase (104 steps/min) in PA at 4 weeks (p = 0.0008), but only after adjusting for reactivity effects.<br>> Another study (Vorrink et al.) showed a 2.5% decrease (159 steps) in steps/day at 12 months (p < 0.001), with no significant difference between intervention and control groups.<br>> Control groups generally experienced declines or no changes in PA.<br><br>**Quality of Life Measures**:<br>> Reported inconsistently; some studies evaluated dyspnoea, emotional function, self-control, and BMI.<br>> No statistically significant differences were observed between intervention and control groups.<br>> Specific instruments (e.g., SGRQ, CAT, EQ-5D) were not explicitly mentioned.<br><br>**Medication Adherence**: Not reported in any of the included studies.<br><br>**Disease Management Metrics**:<br>> Exercise capacity measured via Incremental Shuttle Walk Test (ISWT) and 6-Minute Walk Test (6MWT).<br>> Liu et al. reported a 21% increase in ISWT distance (68.4 m) at 12 months.<br>> Wang et al. reported an 18.3% increase (58.5 m) in ISWT distance at 6 months, while control group decreased by 13% (28.9 m).<br>> Vorrink et al. found no differences in 6MWT or PA levels after 12 months.<br><br>**Adherence and Engagement:**<br>> Adherence to smartphone use was high (89–100%).<br>> Moderate correlation observed between adherence and PA change (e.g., r = 0.62, p = 0.03).<br>> Dropouts due to technical issues or dissatisfaction with the device were noted in multiple studies.	Effect estimates and statistical significance were variably reported across studies. Notable findings include:<br><br>Tabak et al. (2014) reported a 13% increase in steps/minute (104 steps) in the intervention group after adjusting for a reactivity effect, with a p-value of 0.0008, indicating statistical significance.<br><br>Vorrink et al. (2016) found a 2.5% decrease in steps/day (−159 steps) at 12 months in the intervention group, with a p-value < 0.001, but no significant difference between intervention and control groups.<br><br>Liu et al. (2008) showed a 21% increase in ISWT distance (68.4 meters) and a 26.7% increase in walking time (502 seconds) at 3 months. No p-values or confidence intervals were reported.<br><br>Wang et al. (2014) found an 18.3% increase in ISWT distance (58.5 meters) in the intervention group, and a 13% decrease (28.9 meters) in the control group. No p-values were provided.<br><br>A significant correlation between program adherence and change in PA level was reported in one study, with r = 0.62, p = 0.03.<br><br>No pooled effect sizes (e.g., SMDs, mean differences, risk ratios) or confidence intervals were reported, as the review did not conduct a meta-analysis.	Formal heterogeneity statistics (e.g., I^2, Q) were not reported, as no meta-analysis was conducted. However, substantial clinical and methodological heterogeneity was acknowledged. Differences included intervention type and duration, devices and sensors used, feedback methods, outcome measures (e.g., steps/day vs. ISWT), and study designs (RCTs vs. non-RCTs). No subgroup or sensitivity analyses were performed to account for this variability.	Effectiveness of smartphone devices in promoting physical activity and exercise in patients with COPD is currently limited and inconclusive.<br><br>Key Takeaways<br>**Limited Effectiveness**:<br>Smartphone-based interventions showed minimal or no improvements in long-term physical activity (PA) levels.<br>While a few short-term gains were observed (e.g., small increases in steps/min and exercise capacity), they did not sustain over time (e.g., 12-month follow-ups showed no benefit or slight declines in PA).<br><br>**Exercise Capacity**:<br>Improvements in exercise capacity (measured via ISWT) were reported in some studies (e.g., +18–21%), but these findings were not consistent across all trials.<br><br>**Precision and Validity of Devices**:<br>Some studies showed good-to-excellent precision for smartphone-based PA measurements (r = 0.69–0.99), but methodological issues weakened confidence in these results.<br><br>Adherence was high, yet device-related technical issues and dropouts were frequent, potentially undermining intervention effectiveness.<br><br>No evidence for improvement in medication adherence or quality of life, and these outcomes were largely unassessed or underreported.<br><br>**Further high-quality studies are needed**, especially:<br>> Longer-duration trials<br>> Better control for reactivity and blinding<br>> Validated, standardized outcome measures<br>> Stronger attention to usability and engagement design for older COPD patients	**Small sample sizes** in the included studies limited statistical power and generalizability.<br><br>Most studies had **short intervention durations** (4–12 weeks), with only one extending to 12 months, limiting the ability to assess long-term effects.<br><br>**High risk of bias** was present in all studies: only 62.5% reported proper sequence generation, none concealed allocation, and only one used blinded evaluators.<br><br>**High dropout rates** (up to 35.4%) were reported, often due to technical problems or dissatisfaction with devices.<br><br>The **reactivity effect** (behavioral changes from being monitored) may have influenced PA measurements, as participants and control groups were not blinded to device feedback or study aims.<br><br>**No interventional study** verified the validity or precision of the smartphone devices used for measuring physical activity.<br><br>**Validation** studies themselves were **limited** by very small sample sizes, sometimes as low as a single subject, and used incomplete or inappropriate statistical methods (e.g., ANOVA, Pearson's r alone).<br><br>**Quality of life** and **medication adherence** outcomes were either **not assessed** or poorly reported, limiting conclusions about broader health impacts.

Computer and mobile technology interventions for self-management in chronic obstructive pulmonary disease	2017	Systematic review and meta-analysis	3	RCTs	All studies included participants with a clinical diagnosis of COPD but disease severity was underreported.<br><br>Study-specific average ages:<br>> Moy 2015: Mean age 67 years<br>> Tabak 2013: Mean age 66 years<br>> Voncken-Brewster 2015: Mean age 58 years<br><br>Predominantly male population:<br>> Moy 2015: 223 males out of 238 participants (94% - male US veterans)<br>> Voncken-Brewster 2015 (COPD subgroup): Gender breakdown not specifically stated for the subgroup of 284, but full sample (n=1325) had a more balanced distribution (627 men and 698 women).<br>> Tabak 2013: No detailed gender data reported, but the study stratified randomization by gender.<br><br>The studies were conducted in two countries: US (Moy 2015) and the Netherlands (Tobak 2013 - Twente, Voncken-Brewster 2015 — Maastricht)	**Moy et al. (2015)**<br>Type: Internet-mediated pedometer-based walking program<br><br>Technology Used:<br>> Personal computers with internet access<br>> Wearable pedometer device (participants uploaded data weekly)<br><br>Key Components:<br>> Personalized weekly step-count goals (based on recent average + 600 steps or up to 10,000 steps)<br>> Feedback via online dashboard for self-monitoring<br>>Weekly access to motivational and educational content<br>> Online community forum for peer support<br><br>Engagement: Participants wore a pedometer daily and uploaded data at least once weekly<br><br>Duration:<br>> 4-month intensive intervention phase (with new weekly content)<br>> 8-month maintenance phase (same content available but not updated)<br><br>**Tabak et al. (2013)**<br>Type: Telerehabilitation app with real-time feedback<br><br>Technology Used:<br>> Smartphone with 3D accelerometer (Bluetooth-enabled pedometer)<br>> Web portal for symptom tracking and activity diary<br><br>Key Components:<br>> Activity coach: real-time feedback on physical activity through a smartphone app<br>> Symptom diary triggering decision support alerts<br>> Nurse-led training provided pre-intervention<br><br>Engagement: Participants wore devices and used the app daily to report symptoms and receive feedback<br><br>Duration: 4 weeks<br><br>**Voncken-Brewster et al. (2015)**<br>Type: Web-based, computer-tailored behavior change program<br><br>Technology Used:<br>> "MasterYourBreath" online platform accessed via personal login<br><br>Key Components:<br>> Behavior change modules: physical activity and smoking cessation<br>> Six tailored components: health risk appraisal, motivational beliefs, social influence, goal/action planning, self-efficacy, and maintenance strategies<br>> Computer-generated personalized feedback<br><br>Engagement: Participants accessed content ad libitum<br><br>Duration: 6 months	All included studies compared digital interventions to standard, non-technological self-management support. These comparators typically involved face-to-face education, written materials, or non-interactive digital components, and lacked features like real-time feedback, goal setting, or personalized behavior change tools.<br><br>**Moy et al. (2015)**<br>Comparator: Participants received a pedometer and were asked to upload daily step-count data but received no goals, feedback, or educational/motivational content. The web dashboard only displayed total step counts. There was no peer interaction or individualized coaching.<br><br>**Tabak et al. (2013)**<br>Comparator: Participants received usual care, defined as regular in-person exercise training sessions and pulmonary rehabilitation programs.<br>No app, real-time feedback, or digital monitoring tools were used.<br><br>**Voncken-Brewster et al. (2015)**<br>Comparator: Participants received standard primary care, including chronic disease self-management coaching delivered face-to-face by practice nurses.<br>Focus was on improving self-management through traditional interaction, without technology-based support.	Findings were primarily focused on HRQoL and physical activity, with limited reporting on other clinical or behavioral outcomes.<br><br>**Health-Related Quality of Life (HRQoL)**<br>Reported in all 3 studies using validated tools:<br>> St George's Respiratory Questionnaire (SGRQ) – Moy 2015<br>> Clinical COPD Questionnaire (CCQ) – Tabak 2013, Voncken-Brewster 2015<br><br>Direction & Effect:<br>> Digital interventions significantly improved HRQoL at 4 weeks to 6 months (SMD = –0.22; 95% CI –0.40 to –0.03; p = 0.02)<br>> No significant effect at 12 months (MD = 1.1; 95% CI –2.2 to 4.5; p = 0.50)<br><br>**Hospital Admissions**<br>Reported in 1 study (Moy 2015) at 12 months<br>Effect: No significant difference (OR = 1.6; 95% CI 0.8 to 3.2; p = 0.19)<br><br>**Acute Exacerbations**<br>> Reported in 1 study (Moy 2015) at 12 months<br>Effect: No significant difference (OR = 1.4; 95% CI 0.7 to 2.8; p = 0.33)<br><br>**Physical Activity (Daily Step Count)**<br>> Reported in 2 studies (Moy 2015, Tabak 2013) using pedometers<br>Effect:<br>> Significant improvement at 4 weeks to 4 months (MD = 864.06 steps; 95% CI 369.66 to 1358.46; p = 0.0006)<br>> No significant difference at 12 months (MD = 107 steps; 95% CI –498.42 to 712.42)<br><br>**Smoking Cessation**<br>Reported in 1 study (Voncken-Brewster 2015) using 7-day prevalence self-report<br>Effect: No significant difference (OR = 1.06; 95% CI 0.43 to 2.66; p = 0.895)<br><br>**Medication Adherence**: Not reported in any of the included studies.	**Health-Related Quality of Life (HRQoL)**<br>> Pooled SMD (up to 6 months): –0.22 (95% CI: –0.40 to –0.03), p = 0.02<br>> Subgroup using CCQ only: MD = –0.28 (95% CI: –0.44 to –0.12), p = 0.0008<br>> At 12 months (Moy 2015): MD = 1.1 (95% CI: –2.2 to 4.5), p = 0.50<br><br>**Physical Activity (Step Count)**<br>> Pooled MD (up to 4 months): 864.06 steps (95% CI: 369.66 to 1358.46), p = 0.0006<br>> Tabak 2013: MD = 986 (95% CI: 312.02 to 1659.98), p = 0.004<br>> Moy 2015 at 4 months: MD = 722 (95% CI: –5.46 to 1449.46), p = 0.05<br>> Moy 2015 at 12 months: MD = 107 (95% CI: –498.42 to 712.42), p = 0.73<br><br>**Hospital Admissions (Moy 2015):**<br>> OR = 1.6 (95% CI: 0.8 to 3.2), p = 0.19<br><br>**Acute Exacerbations (Moy 2015):**<br>> OR = 1.4 (95% CI: 0.7 to 2.8), p = 0.33<br><br>**Smoking Cessation (Voncken-Brewster 2015):**<br>> OR = 1.06 (95% CI: 0.43 to 2.66), p = 0.895	Heterogeneity was assessed using the I^2 statistic and Chi^2 (Q) test. Across all meta-analyses in the review, heterogeneity was consistently low:<br><br>**Health-Related Quality of Life (HRQoL) – All Studies:**<br>> I^2 = 0%, Chi^2 = 0.39, p = 0.82<br>> Interpretation: No heterogeneity<br><br>**HRQoL (CCQ subgroup only):**<br>> I^2 = 0%, Chi^2 = 0.01, p = 0.91<br><br>**Physical Activity (Daily Step Count, up to 4 months):**<br>> I^2 = 0%, Chi^2 = 0.27, p = 0.60<br><br>**Model Used**: Random-effects model<br><br>**Subgroup Analyses**: Planned for age, education, COPD severity, and follow-up duration but not conducted due to lack of data.<br>> One internal analysis (Moy 2015) found that age was negatively associated with change in step count (–33 steps per year, p = 0.03)<br><br>**Conclusion**: No statistical heterogeneity was detected across the included studies; outcomes were consistent.	**Effectiveness of Digital Interventions**<br>Short-term benefits:<br>> Interventions delivered via computer or mobile technology significantly improved health-related quality of life (HRQoL) and physical activity levels in people with COPD for durations of up to 6 months.<br>> These interventions outperformed standard care (face-to-face, written, or basic digital education) on these outcomes.<br><br>Long-term outcomes (12 months):<br>> Improvements in HRQoL and physical activity were not sustained at 12 months.<br>> Engagement dropped off, and benefits were no longer significant in the maintenance phase.<br>> Hospitalizations, acute exacerbations, and smoking cessation showed no statistically significant differences between intervention and control groups at 12 months.<br><br>**Safety and Acceptability**<br>> Safety: No harm was reported from digital interventions.<br>One study (Moy 2015) reported more adverse events in the intervention group (mainly musculoskeletal) but noted these were anticipated and did not lead to hospitalization.<br>> Acceptability: Participants appeared to use and engage with the interventions during active phases, but sustained usage declined over time.<br><br>**Evidence Quality and Generalizability**<br>> The evidence comes from only three RCTs, all of which were assessed as having a high risk of bias and small sample sizes.<br>> While findings suggest potential benefits of smart technology in COPD self-management, the quality of evidence is low, and no firm conclusions can be drawn regarding routine clinical use.<br>> The interventions may be more beneficial for tech-savvy users or those with an interest in digital tools.	All three included studies were assessed as having a **high risk of bias**, particularly due to lack of blinding (performance and detection bias), self-reported outcomes, and incomplete outcome data.<br><br>**Small sample sizes** and only three included RCTs limited statistical power and precision; wide confidence intervals were common.<br><br>Important **outcomes** were **not reported**, including medication adherence, cost-effectiveness, lung function, functional capacity, anxiety/depression, and self-efficacy.<br><br>Only **one** study reported **12-month data**; others had short follow-up (4–6 months), limiting insights on sustained behavior change.<br><br>**Comparator** conditions were **poorly described**, hindering interpretation of relative effects.<br><br>**Generalizability** was **limited** due to demographic skew (e.g., mostly older male veterans in one study).<br><br>Patient **engagement** and **adherence** with digital tools were **not** consistently or quantitatively **reported**, making it difficult to assess long-term usage patterns or dropout impact.
Telehealthcare in COPD: A systematic review and meta-analysis on physical outcomes and dyspnea	2015	Systematic review and meta-analysis	9	RCTs	Patients diagnosed with moderate to severe COPD.<br><br>Mean ages across the studies ranged from approximately 64 to 73 years, with most studies clustering around 66–70 years.<br><br>Gender distribution: overall, 34% were women.<br><br>Studies were conducted in North America (Canada, USA), Europe (Spain, UK), and Asia (South Korea).	All included interventions were home-based and involved some form of communication technology, used primarily for feedback, monitoring, education, or exercise support.<br><br>**Key Components**<br>> Phone Calls: Most common modality. Delivered reinforcement, education, and exercise motivation. Frequency varied: weekly, biweekly, monthly.<br>> Web Platforms / Internet: Used for reporting symptoms, receiving tailored education, and chatting with clinicians. Interactive components included live chats and bulletin boards.<br>> Mobile Phones / SMS: Daily symptom logging and exercise tracking via mobile apps or SMS. Some studies included automated feedback or alerts to clinicians.<br>> Video Calls: Real-time education (e.g., pursed-lips breathing training) via Skype.<br>> Education Modules: Delivered either through websites or phone, often tailored to self-management and exercise strategies.<br>> Exercise Support: Many interventions included prescribed home exercise regimens and regular digital follow-up on adherence.<br>> Monitoring & Reporting Tools: Patients' self-reported symptoms, physical activity, and health metrics. Feedback was often given via the same platforms (website, app, or phone).<br><br>**Intervention Duration**<br>Ranged from 4 weeks to 18 months.<br>> Most common durations were 8 weeks, 6 months, or 12 months.<br>> Longer durations typically involved tapering contact frequency (e.g., weekly → monthly).	Usual care with standard clinical management and no added structured support (e.g., Bourbeau 2003; Garcia-Aymerich 2007; Waterhouse 2010 in one arm).<br><br>In-person supervised pulmonary rehabilitation (PR), consisting of 3 supervised exercise sessions per week for 8 weeks, followed by unsupervised home exercise with occasional follow-up (e.g., Maltais 2008; Waterhouse 2010).<br><br>Brief educational interventions such as a single education session or printed materials without reinforcement (e.g., Oh 2003; Nield 2012).<br><br>Home exercise plans without digital feedback, supported by paper-based materials or in-person coaching (e.g., Nguyen 2009; Carrieri-Kohlman 1996).<br><br>Non-feedback phone calls, where control groups received periodic calls about adherence or general health topics but without personalized coaching or interactive support (e.g., Carrieri-Kohlman 1996; Nguyen 2013).<br><br>Some control groups also had limited access to nurses or case managers by phone, but this was passive and reactive rather than structured or proactive (e.g., Bourbeau 2003).	**Primary outcomes** across the studies were physical activity level, physical capacity, and dyspnea.<br><br>> Physical activity level: Improvement found in telehealthcare group; pooled result showed MD = 64.7 minutes/week (95% CI: 54.4 to 74.9); significant effect (p < 0.001); evidence based on one study post-exclusion due to heterogeneity<br>> Physical capacity: No significant difference between groups; pooled MD = –1.3 meters (95% CI: –8.1 to 5.5); p = 0.708; measured primarily using 6MWT<br>> Dyspnea: No significant improvement; pooled SMD = 0.088 (95% CI: –0.056 to 0.233); p = 0.232; assessed using multiple validated scales<br><br>**Dyspnea was assessed using**:<br><br>> CRQ-D (Chronic Respiratory Questionnaire – Dyspnea subscale): Used in 5 studies; no significant between-group difference despite within-group improvements<br>> MRC Dyspnea Scale: Used in Garcia-Aymerich (2007); no change observed in either group<br>> SOBQ (Shortness of Breath Questionnaire): Used in Nield (2012); no between-group differences<br>> Borg Scale and VAS: Used in several studies during exercise testing; inconsistent findings, no pooled effect<br><br>**Physical capacity was assessed using**:<br><br>> 6-Minute Walk Test (6MWT): Most commonly reported measure (used in 7 studies); pooled MD = –1.3 meters (not significant); some studies showed within-group improvements, but no between-group effect<br>> Other capacity measures: Incremental treadmill test (ITT), arm endurance, cycling endurance, and Endurance Shuttle Walk Test (ESWT); results inconsistent or not pooled<br><br>**Physical activity level was measured using**:<br><br>> Accelerometers: Used in Nguyen (2009); step counts showed control group performed better than telehealthcare group<br>> Self-report questionnaires: Used in Nguyen (2013); only study included in final meta-analysis after heterogeneity adjustment<br>> Activity monitoring: Telehealthcare associated with increased minutes of endurance activity; improvements in both frequency and duration in some groups<br><br>**Medication adherence**: Not reported in any of the included studies<br><br>**Disease management metrics**<br><br>> Self-care and exercise adherence: Encouraged and monitored in many interventions; data not systematically reported<br>> Symptom tracking and goal setting: Part of intervention protocols (e.g., Nguyen 2009, 2013), but not analyzed as outcomes<br>> Hospital admissions or ED visits: Mentioned in rationale and background, but not included as outcomes or reported in results<br>> Patient satisfaction: Not measured or discussed in included studies<br><br>**Direction/magnitude of change**:<br><br>> Physical activity: MD = 64.7 minutes/week (95% CI: 54.4 to 74.9) – significant increase favoring telehealthcare<br>> Physical capacity (6MWT): MD = –1.3 meters (95% CI: –8.1 to 5.5) – no significant change<br>> Dyspnea (SMD): SMD = 0.088 (95% CI: –0.056 to 0.233) – no meaningful or statistically significant change<br>> Publication bias: Not detected (Egger's test p = 0.813 for physical capacity; p = 0.412 for dyspnea)	Physical activity level:<br>> Mean Difference (MD) = 64.7 minutes/week (95% CI: 54.4 to 74.9)<br>> p < 0.001 – statistically significant<br>> Based on final meta-analysis of two intervention groups from a single study (Nguyen 2013), after exclusion of outliers due to high heterogeneity<br><br>Physical capacity (6MWT):<br>> Mean Difference (MD) = –1.3 meters (95% CI: –8.1 to 5.5)<br>> p = 0.708 – not statistically significant<br>> After exclusion of two heterogeneous studies (Oh et al., 2003 and Nguyen 2013); final analysis included five study groups<br>‣ No publication bias detected (Egger's test p = 0.813)<br><br>Dyspnea:<br>> Standardized Mean Difference (SMD) = 0.088 (95% CI: –0.056 to 0.233)<br>> p = 0.232 – not statistically significant<br>> Meta-analysis included multiple dyspnea scales (CRQ-D, SOBQ, MRC, Borg, VAS) recalculated as SMDs<br>> No publication bias detected (Egger's test p = 0.412)	Heterogeneity was assessed using the I^2 statistic and addressed through sensitivity analyses and model selection:<br><br>**Physical Activity Level:**<br>> Initial heterogeneity was high (I^2 = 85%).<br>> After excluding an outlier study (Nguyen et al., 2009), heterogeneity dropped to 0%.<br>> Final meta-analysis included only two intervention groups from Nguyen et al. (2013).<br><br>**Physical Capacity (6MWT):**<br>> Initial heterogeneity was also high (I^2 = 82%).<br>> After excluding two outlier studies (Oh et al., 2003 and Nguyen et al., 2013), I^2 was reduced to 14%.<br><br>**Dyspnea:**<br>> No I^2 value explicitly reported, but heterogeneity was considered during analysis.<br>> Results from various dyspnea scales (CRQ-D, MRC, SOBQ, Borg, VAS) were standardized to SMDs.<br>> A random-effects model was used due to expected variation in measurements and delivery methods.<br><br>**Handling of Heterogeneity:**<br>> Random-effects models are applied when I^2 > 30%.<br>> Fixed-effects models are used when I^2 ≤ 30%.<br>> Outliers were excluded iteratively until heterogeneity was reduced below threshold.<br>> Publication bias was assessed with Egger's test (no significant bias detected).<br><br>**Sources of Heterogeneity Identified:**<br>> Differences in intervention formats (phone, mobile, internet).<br>> Variation in outcome measurement tools (objective vs. subjective).<br>> Comparator group strength and design variability.<br>> High risk of bias in many included studies.	Overall, the review concludes that telehealthcare may improve physical activity levels in COPD patients, but has no clear benefit on physical capacity or dyspnea.<br><br>**Physical activity:**<br>> Telehealthcare showed a statistically significant effect in increasing physical activity time compared to control<br>> However, this conclusion is based largely on a single study after exclusion of others due to heterogeneity<br>> The authors urge caution in interpretation due to this limited evidence base<br><br>**Physical capacity and dyspnea:**<br>> No significant differences between telehealthcare and comparator groups<br>> Both groups in many studies showed within-group improvements, suggesting benefit from general intervention exposure rather than digital format specifically<br><br>Telehealthcare design and delivery formats were varied, but commonly included feedback, education, and self-management support delivered via phone, internet, or mobile platforms.<br>> Phone-based counseling and reinforcement were the most frequently used modality<br>> Some studies used web portals, live video, or SMS-based systems<br>> Most interventions combined telehealth with education and exercise components<br><br>Telehealthcare was considered a potentially viable extension of pulmonary rehabilitation (PR) and may help address access barriers.<br><br>> Particularly relevant for patients unable to attend in-person PR due to transportation or geographic constraints<br>> The authors emphasized the need for more high-quality, well-powered studies to evaluate long-term effects and patient subgroups	**High risk of bias:** All studies were rated as having high risk of bias in at least two Cochrane domains. Common issues included lack of blinding (particularly of participants and personnel), selective outcome reporting, and incomplete outcome data.<br><br>**Blinding**: Blinding of participants and personnel was not feasible due to the nature of the interventions. In some cases, outcome assessors were also not blinded, increasing the risk of detection bias.<br><br>**Power and sample size**: Five studies did not report power calculations, and two studies failed to reach required sample sizes. One study recalculated power during the trial. Small sample sizes limited the statistical power and generalizability of findings.<br><br>**Heterogeneity**: There was substantial variability in intervention types (e.g., phone, internet, mobile), comparator interventions (e.g., usual care, supervised PR), outcome measures (e.g., different scales and instruments), and intervention durations (ranging from 4 weeks to 18 months). This heterogeneity limited meta-analytic synthesis and interpretability.<br><br>**Inconsistent reporting**: Some outcomes (e.g., dyspnea) were described in methods but not reported with numerical data. In some cases, results were transformed or selectively reported based on significance.<br><br>**Generalisability**: Participants were mostly patients with moderate to severe COPD. There was poor representation of women in some studies (e.g., one study included only men), and geographic scope was limited to high-income countries. No subgroup analyses by age, sex, or disease severity were performed.<br><br>**Intervention specificity**: Telehealthcare was often combined with education or exercise, making it difficult to isolate the specific effects of the digital component. Few studies used internet-based platforms as the primary delivery method.
The effect of smartphone interventions on patients with chronic obstructive pulmonary disease exacerbations: A systematic review and meta-analysis	2016	Systematic review and meta-analysis	6	RCTs (5), quasi-randomized controlled trial (1)	Some studies targeted patients diagnosed with moderate to severe COPD; others included participants across all stages of COPD severity.<br><br>Mean age across studies: 70 years<br>Gender distribution: 74% male<br>Geographic distribution: Netherlands, Italy, Germany, UK, USA, Taiwan	All interventions involved smartphones as an hub for different COPD self-management and monitoring functions.<br><br>Components<br>**Symptom Monitoring**<br>> Collect daily respiratory symptoms<br><br>**Physiological Monitoring**<br>Devices paired with smartphones to measure:<br>> Oxygen saturation<br>> Heart rate<br>> Lung function<br>> Physical activity<br>> Body temperature and galvanic skin response<br><br>**Exercise and Activity Tracking**<br>Some studies included home-based exercise programs delivered through the smartphone with feedback on weekly exercise progress and daily activity levels.<br><br>**Educational Content & Self-Management**<br>Several interventions provided:<br>> Guidance on symptom recognition<br>> COPD self-care strategies<br>> Instructions for managing mild exacerbations<br><br>**Feedback Mechanisms**<br>> Automated feedback: Triggered by symptom thresholds (e.g., advice to initiate medications).<br>> Human feedback: Daily or periodic follow-up calls from study nurses or physicians.<br>> Reminders: Automated phone calls or alerts to ensure adherence and medication supply.<br><br>**Web Portals** (1 Study)<br>One study (Tabak et al.) included a self-management web portal linked to the smartphone app for integrated data sharing and behavioral recommendations.	Control group went under standard care involving routine medical care without digital enhancements.<br><br>**Usual care included**<br>> Regular follow-up visits<br>> Prescribed medications<br>> Patient-initiated contact during symptom worsening<br>> Educational materials or prior training<br><br>**Minimal Technology Use**<br>Both the intervention and control groups received smartphones, but:<br>> Only the intervention group had automated phone calls for medication reminders and regimen adherence.<br>> The control group received no active reminders, despite having access to the device.<br><br>**No Digital Health Support**<br>In five of the six included studies, the control group had no access to digital monitoring, smartphone applications, or remote feedback systems.	**Primary outcomes** across the studies were COPD exacerbation frequency and user engagement.<br><br>COPD exacerbation frequency: Significant and consistent reduction in the intervention group; pooled OR = 0.20 (95% CI: 0.07 to 0.62); 80% reduction in odds; moderate heterogeneity (I^2 = 59%); moderate certainty.<br>User engagement and satisfaction: Mixed and sparsely reported; limited qualitative data from two studies; very low certainty.<br><br>**Quality of life measures** were not systematically reported.<br><br>SGRQ, CAT, EQ-5D: Not assessed in the included studies. No standardized QoL tools were used, and QoL was not a primary or secondary outcome in any meta-analyzed trial.<br>User feedback: Only two studies collected qualitative or satisfaction data (via interviews or Client Satisfaction Questionnaire), but not through validated QoL instruments.<br><br>**Medication adherence**: Not reported or measured across any of the included studies.<br><br>One study included automated medication reminders, but adherence impact was not quantified or evaluated.<br><br>**Disease management metrics**<br><br>Symptom monitoring and early detection: All studies enabled daily or near-daily tracking of symptoms; suggested improved self-management and early identification of exacerbations; qualitative support but no pooled metrics; low certainty.<br>Hospital admissions or emergency department visits: Not directly reported or pooled; presumed reduction based on exacerbation outcomes but not separately measured.<br>Patient satisfaction and usability: Two studies reported satisfaction or usability feedback; mixed findings; no consistent preference for digital interventions; very low certainty.<br><br>**Direction/magnitude of change**<br><br>> Exacerbation frequency: OR = 0.20 (95% CI: 0.07 to 0.62) – large and significant reduction in exacerbation risk with smartphone use.<br>> Hospital visits and admissions: Not assessed quantitatively – presumed secondary benefit but not validated.<br>> User satisfaction (qualitative): No meta-analyzed data; descriptive reports indicated generally favorable responses but highly variable.<br>> Quality of life: Not measurable due to absence of standardized instruments.	Pooled odds ratio (OR) for having a COPD exacerbation: 0.20<br><br>95% confidence interval (CI): 0.07 to 0.62<br><br>Indicates an 80% reduction in the odds of experiencing an exacerbation with smartphone intervention compared to usual care.<br><br>Statistical model used: Random effects model<br><br>Number of studies included in meta-analysis: 3<br><br>Heterogeneity reported:<br>> I^2 = 59% (moderate heterogeneity)<br>> χ^2 = 4.9, p = 0.08<br><br>Analysis types:<br>> 2 studies used intention-to-treat analysis<br>> 1 study used per-protocol analysis<br><br>Software used: Review Manager (RevMan) version 5.3<br><br>Publication bias: Not assessed due to insufficient number of studies (fewer than 10)	Heterogeneity was formally assessed using I^2 statistic and Chi-square (χ^2) test<br><br>Results<br>> I^2 = 59% - This indicates moderate heterogeneity among the studies included in the meta-analysis.<br><br>Interpretation: About 59% of the variability in effect estimates is due to differences between studies rather than chance.<br><br>> χ^2 = 4.9, p = 0.08 - The p-value suggests that heterogeneity is approaching statistical significance, but not quite reaching the conventional threshold (p < 0.05).<br><br>This further supports the presence of moderate inconsistency across studies.	Smartphone-based interventions show promising potential in helping to reduce the frequency of exacerbations in individuals with COPD. Findings from the meta-analysis revealed a statistically significant and clinically meaningful benefit, with patients using smartphone interventions experiencing an 80% lower chance of having at least one exacerbation compared to those receiving usual care (OR = 0.20). Notably, this positive effect was consistently observed across all three studies included in the meta-analysis, even though the studies differed somewhat in their designs, populations, and implementation strategies.<br><br>**Synergistic Approach Required**<br>The authors emphasize that smartphone use alone may not be sufficient. Effectiveness seems to rely on synergistic strategies, including:<br>> Regular symptom monitoring<br>> Physiological tracking (e.g., oxygen saturation, FEV_1)<br>> Education and self-management support<br>> Feedback—either automated or clinician-delivered<br><br>**Interpretation with Caution**<br>While promising, the results must be interpreted cautiously due to:<br>> Moderate heterogeneity (I^2 = 59%)<br>> Small sample sizes in included studies<br>> Variability in intervention components and study design	All included studies had **small sample sizes** (<100 participants), limiting statistical power and generalizability.<br><br>There was substantial **methodological heterogeneity** across studies in terms of intervention content, delivery mode, monitoring frequency, and feedback strategy.<br><br>**Inconsistent definitions** of exacerbation were used, ranging from symptom reports to medication use and web-based self-management tools, which may have introduced false positives.<br><br>Only one study was rated as "Good" quality; the others were rated "Fair" or "Poor," reflecting **risks** of **bias** due to unclear randomization, lack of blinding, and incomplete data.<br><br>**Follow-up durations** were **short** (4–9 months), preventing assessment of long-term effects and sustainability.<br><br>**Key outcomes** such as medication adherence and quality of life were either **not measured** or not assessed using validated tools.<br><br>Potential **selection bias** due to all participants being provided smartphones, possibly favoring more technologically adept individuals.<br><br>**User experience** data were **limited** and collected in only two studies, using non-standardized or qualitative methods.<br><br>**Publication bias** could not be assessed due to the small number of studies included in the meta-analysis (n=3).
A Systematic Review and Meta-Analysis of Telemonitoring Interventions on Severe COPD Exacerbations	2021	Systematic review and meta-analysis	22	RCTs	Patients with a COPD diagnosis who have severe conditions or a history of severe exacerbations were included. One study, however, exclusively included participants receiving home oxygen therapy.<br><br>The mean age of participants ranged between 63 and 81 years. Most studies reported that 30–70% of participants were male.<br><br>Geographic distribution of studies included: UK, Denmark, Spain, Italy, Ireland, France, Belgium, Greece, US, Canada, Taiwan, Hong Kong, South Korea, and Australia.	Telemonitoring was the central digital intervention examined.<br><br>**Devices and platforms:**<br>Online platforms (web portals, remote care systems) connected to:<br>> Mobile phones / smartphones<br>> Android tablets<br>> Personal digital assistants (PDAs)<br>> Dedicated telehealth equipment<br><br>Video conferencing included in some studies<br><br>**Monitored Parameters**<br>Most common types of patient data collected and transmitted:<br>> Oxygen saturation (SpO_2) – monitored in 91% of studies<br>> Symptoms – 73%<br>> Heart rate (HR) and blood pressure (BP)<br>> Spirometry (e.g., FEV_1)<br>> Body temperature (BT) and body weight (BW)<br>> Medication use/adherence<br>> Respiratory mechanics, activity levels, and electrocardiography (ECG) in some studies<br><br>**Clinical Response & Integration**<br>Data were monitored by healthcare professionals.<br>If abnormal values or missing data were detected, clinicians responded (e.g., phone call, visit scheduling).<br>Alerts or feedback mechanisms varied; some platforms allowed real-time feedback to patients.<br><br>**Additional Features in Some Interventions**<br>> Self-Management Support<br>Some interventions included educational videos, action plans, or self-efficacy coaching.<br>A few studies explicitly combined telemonitoring with self-management programs, aiming to empower patients.<br>> Telerehabilitation<br>A minority of studies added remote rehabilitation support, such as physical activity monitoring and video-based sessions.<br><br>**Post-Discharge Support**<br>Several interventions began immediately after hospitalization for COPD exacerbation to reduce readmission risk.<br><br>**Duration of Interventions**<br>Ranged from 2 to 26 months with 90% of studies had a minimum duration of 6 months.	The comparator was usual care, defined as the standard treatment and management patients would receive without any form of telemonitoring. This included:<br><br>> Routine clinical follow-up and monitoring by primary or specialist care providers.<br>> Standard pharmacological treatment as per national or international COPD guidelines (e.g., bronchodilators, corticosteroids).<br>> Access to emergency services and hospitalization as needed.<br>> In some studies, pulmonary rehabilitation, written action plans, or education may have been offered as part of standard care.<br>> Participants did not receive any digital intervention, including symptom tracking, remote vital sign monitoring, or telecommunication with healthcare teams.<br><br>All comparator groups were matched to intervention groups in terms of background care, ensuring that the only difference was the absence of telemonitoring technology. This controlled design allowed for the isolation of effects attributable to digital interventions.	**Primary outcomes** across the studies were hospital admissions, emergency room (ER) visits, and time to first exacerbation.<br>> Hospital admissions: No consistent or statistically significant reduction; only 5 of 22 studies (23%) reported fewer admissions with telemonitoring. Meta-analysis (10 studies): SMD = –0.10 (95% CI: –0.21 to 0.01); not significant.<br>> ER visits: Some evidence of benefit; 3 of 13 studies (23%) reported significant reduction. Meta-analysis (7 studies): SMD = –0.14 (95% CI: –0.28 to –0.01); statistically significant but small effect.<br>> Time to first admission: Mixed effects; 3 of 9 studies showed significant delay in time to first admission post-intervention. No pooled estimate reported.<br><br>**Quality of life measures were assessed using**:<br>> SGRQ: Improvements noted in 4 studies; outcomes met or approached clinical relevance (≥4-point MID) in some cases; tools used: SGRQ total score; direction: positive, but inconsistent across studies.<br>> EQ-5D: Mixed results; improvement in a few studies (e.g., Farmer et al.), unchanged in others. Assessed general health status, not COPD-specific.<br>> SF-36: Reported in multiple studies; generally no significant changes.<br>> CRQ / NCSI / CCQ: Used in a few studies; generally neutral results. CRQ total and dyspnoea domains used. Most showed no statistically significant change.<br><br>**Medication adherence**:<br>Not systematically assessed across all studies.<br><br>Disease management metrics<br>> Self-management and action plans: Improved in some studies (e.g., Boer et al.); assessed with self-report or symptom monitoring compliance; results not pooled.<br>> Dyspnoea (PEFR/Symptom logs): Some improvements noted (e.g., Cordova et al.), though not consistent across all studies.<br>> Hospital admissions and ER visits:<br>– ER visits: Statistically significant reduction (SMD = –0.14; 95% CI: –0.28 to –0.01);<br>– Hospitalizations: Not statistically significant (SMD = –0.10; 95% CI: –0.21 to 0.01).<br>> Healthcare utilization (readmission, PCP/nurse contact): Reduced in some studies (e.g., Walker, Farmer, Lewis); not consistently reported or pooled.<br>> Patient satisfaction: High across all 8 studies that assessed it; tools varied (interviews, custom questionnaires); direction: consistently positive.<br><br>**Direction/magnitude of change**:<br>> Hospital admissions (meta-analysis): SMD = –0.10 (95% CI: –0.21 to 0.01) – not significant.<br>> ER visits (meta-analysis): SMD = –0.14 (95% CI: –0.28 to –0.01) – small but statistically significant reduction.<br>> QoL (SGRQ): Reported improvements in 4 studies; in some cases, changes exceeded MID (>4 points), though results were inconsistent and not pooled.<br>> Costs: Statistically significant reduction in 4 of 8 studies; driven by reduced ER visits or hospitalization duration.<br>> Mortality: No significant difference observed in any study.<br>> Anxiety and depression (HADS): Mixed or no change; reported in 3 studies.<br>> Self-efficacy and knowledge (LINQ, SECD6): Reported in a few studies; results were neutral or modestly positive	**Hospital Admissions**<br>A total of 10 studies were included in the meta-analysis evaluating hospital admissions due to severe COPD exacerbations.<br>> Standardized Mean Difference (SMD): –0.10<br>> 95% Confidence Interval (CI): –0.21 to 0.01<br>> p-value: Not directly reported; result was not statistically significant<br>> Heterogeneity: I^2 = 24%, p = 0.22 → low heterogeneity<br>> Model Used: Fixed-effects model<br><br>**ER Visits**<br>A total of 7 studies were pooled for the analysis of emergency room visits due to COPD exacerbations.<br>> SMD: –0.14<br>> 95% CI: –0.28 to –0.01<br>> Statistical significance: Statistically significant (small effect size)<br>> Heterogeneity: I^2 = 18%, p = 0.29 → low heterogeneity<br>> Model Used: Fixed-effects model<br><br>**Publication Bias:**<br>Egger's test showed no evidence of publication bias for either outcome:<br>> Hospital admissions: p = 0.606<br>> ER visits: p = 0.583<br><br>**Other Statistical Findings**<br>> Time to first hospitalization: Reported in 9 studies; 3 showed statistically significant delays in the intervention group. No meta-analysis conducted.<br>> Length of hospital stay: Evaluated in 13 studies; only 2 showed a statistically significant reduction in admission duration.<br>> Cost-effectiveness: Assessed in 8 studies; 4 studies reported statistically significant reductions in healthcare costs, primarily from reduced ER visits and shorter hospital stays.<br>> Quality of Life (QoL): Reported in 13 studies using instruments such as SGRQ, EQ-5D, SF-36, and CRQ.<br>Five studies showed statistically significant improvements, but pooled analysis was not performed due to heterogeneity in outcome measures.<br>> Mortality, anxiety, and depression: Reported in multiple studies; no statistically significant differences were observed between intervention and control groups.	Hospital Admissions (Meta-analysis):<br>> I^2 = 24%, p = 0.22<br>> Interpretation: Low heterogeneity<br>> Model used: Fixed-effects model<br><br>ER Visits (Meta-analysis):<br>> I^2 = 18%, p = 0.29<br>> Interpretation: Low heterogeneity<br>> Model used: Fixed-effects model<br><br>Subgroup or source analysis:<br>The review did not conduct formal subgroup analyses or meta-regressions due to the low statistical heterogeneity. However, it acknowledged clinical heterogeneity across studies in:<br>> Study population characteristics (e.g., COPD severity, prior exacerbations)<br>> Types and formats of telemonitoring interventions<br>> Duration of follow-up (ranged from 2 to 26 months)<br>> Outcomes and instruments used<br><br>Overall<br>While statistical heterogeneity was low, clinical and methodological variability existed.<br>These differences did not materially affect the consistency of pooled effect estimates, particularly for ER visits.	Telemonitoring was effective in reducing emergency room (ER) visits due to severe COPD exacerbations.<br>The meta-analysis of 7 studies showed a small but statistically significant reduction in ER visits (SMD = –0.14; 95% CI: –0.28 to –0.01).<br>This suggests that telemonitoring may help detect early warning signs and allow timely intervention, thus preventing unnecessary ER use.<br><br>Telemonitoring did not significantly reduce hospital admissions for COPD exacerbations.<br>The meta-analysis of 10 studies found no statistically significant difference between intervention and control groups (SMD = –0.10; 95% CI: –0.21 to 0.01).<br>The authors concluded that telemonitoring alone is unlikely to prevent hospitalizations, possibly because exacerbations requiring admission might not be avoidable through early detection alone.<br><br>Quality of life, mortality, and cost-effectiveness outcomes were mixed or inconclusive.<br>While some studies reported improvements in QoL or cost savings, the overall evidence did not support a consistent benefit.<br>Mortality outcomes were unchanged across most studies.<br><br>Patient satisfaction with telemonitoring was high.<br>All 8 studies that assessed satisfaction reported positive feedback, indicating that telemonitoring is acceptable and feasible for patients with COPD.<br><br>Clinical heterogeneity was acknowledged, but statistical heterogeneity was low.<br>Despite differences in study design, technology used, and population characteristics, the effect sizes were consistent, especially for ER visit outcomes.<br><br>Telemonitoring may serve best as part of a broader digital care strategy.<br>The review suggests telemonitoring could be more effective when integrated with other interventions such as self-management education or telerehabilitation.	**Potential for missed studies**<br>Although a comprehensive search strategy was used across nine databases, the authors acknowledged that some relevant studies may have been missed.<br>Example: The PROMETE II trial, a large multicenter RCT, was not included because it was published as a research letter rather than a full article.<br><br>**Clinical and methodological heterogeneity**<br>There was substantial variation in patient populations, intervention formats, technologies used, and outcome definitions across the 22 studies.<br>Even though statistical heterogeneity was low (I^2 < 25%), the clinical heterogeneity made it difficult to generalize findings or identify which components of telemonitoring were most effective.<br><br>**Inconsistent outcome reporting**<br>Studies used a wide variety of tools to assess quality of life, costs, self-management, and other secondary outcomes.<br>This prevented meta-analysis of most secondary outcomes and made comparison across studies challenging.<br><br>**Blinding was difficult or not feasible**<br>Many studies had high risk of bias in participant and personnel blinding, which is typical for digital health interventions.<br>The inability to blind participants may have introduced performance bias, especially for subjective outcomes like QoL or satisfaction.<br><br>**Lack of standardized definitions for COPD exacerbation**<br>Exacerbation definitions varied, often relying on subjective symptom reports or healthcare utilization, which could inflate or misclassify event rates.<br>This inconsistency may partly explain why some studies showed benefit while others did not.<br><br>**Limited duration in some trials**<br>While most studies lasted ≥6 months, a few had short durations (as little as 2 months), which may not have been enough to capture meaningful changes in outcomes such as hospitalizations or QoL.<br><br>**Inadequate exploration of subgroup effects**<br>The authors did not conduct formal subgroup or sensitivity analyses to examine differential effects by disease severity, age, telemonitoring type, or other variables—potentially missing important effect modifiers.
