# Supplementary material for: Artificial Intelligence Methods and Digital Intervention Strategies for Predicting and Managing Chronic Obstructive Pulmonary Disease Exacerbations: An Umbrella Review
Source: Healthcare (Basel). 2025 Nov 24;13(23):3037. doi: 10.3390/healthcare13233037 (PMC12691994; doi:10.3390/healthcare13233037)
Supplement: Supplementary file 1 [file healthcare-13-03037-s001.zip › s1_full_tables.pdf]

Table S1. Inclusion criteria, including descriptions and unique identifiers used in the screening process.

Inclusion Criteria		
**Criteria**	**Description**	**Code**
Scope of Research	The review must focus primarily on COPD exacerbations and address at least one of the following domains: 1. The use of wearable-derived biosignals and/or artificial intelligence (AI) in predicting COPD exacerbations, including discussion of associated challenges or limitations 2. The impact of digital health interventions (e.g., mobile apps, remote monitoring, telehealth, automated reminders) on long-term disease management, quality of life, or medication adherence in COPD patients. Reviews focusing exclusively on other respiratory diseases or chronic conditions will be excluded.	INC_Scope
Type of Publication	Only systematic reviews, meta-analyses, or umbrella reviews published in peer-reviewed journals will be included. Narrative reviews, editorials, conference abstracts, and opinion papers will be excluded.	INC_ReviewTypeF
Relevance of Technologies Discussed	For Topic 1, included reviews must analyze or summarize the use of AI models and/or wearable-derived biosignals (e.g., heart rate, SpO_2, respiratory rate, activity level) in the context of COPD exacerbation prediction. For Topic 2, reviews must evaluate digital health interventions in the management of COPD.	INC_TechRelevance
Human-Centric Evidence Base	Reviews must synthesize evidence based on human studies. Reviews focusing solely on animal or in vitro studies will be excluded.	INC_Humans
Scalability or Practical Relevance	Included reviews should assess technologies or interventions that are scalable or feasible for clinical or real-world implementation (e.g., mobile sensors, wearable devices, apps), not limited to technologies restricted to hospital or advanced clinical settings (e.g., imaging modalities, invasive diagnostics).	INC_Scalability
Outcomes Reported	Reviews must report or summarize relevant outcomes such as: – For Topic 1: prediction accuracy, model performance, challenges in data collection/use – For Topic 2: effects on disease management, medication adherence, or quality of life.	INC_Outcomes

Table S2. Exclusion criteria, including descriptions and unique identifiers used in the screening process.

Exclusion Criteria		
**Criteria**	**Description**	**Code**
Non-relevant Topic	Reviews that do not focus on COPD or that focus on other chronic conditions or respiratory diseases (e.g., asthma, cystic fibrosis) will be excluded. Also excluded are reviews that do not address at least one of the following: – Use of wearable-derived biosignals for predicting COPD exacerbations – Use of AI techniques in forecasting COPD exacerbations – Impact of digital health interventions on disease management, quality of life, or medication adherence in COPD.	EXC_Scope
Non-human Evidence Base	Reviews focusing solely on animal or in vitro studies will be excluded.	EXC_Humans
Invasive or Non-scalable Technology Focus	Reviews focusing only on non-scalable, invasive, or hospital-based technologies (e.g., ultrasounds, imaging, bronchoscopy) without discussing wearables or scalable digital tools will be excluded.	EXC_Scalability
Publication Type	Non-systematic reviews, narrative reviews, editorials, preprints, conference abstracts, and posters will be excluded. Only systematic reviews, meta-analyses, or umbrella reviews are eligible.	EXC_PubType
Publication Period	Reviews not published between 2014 - 2024 will be excluded.	EXC_Year
Publication Language	Reviews not published in English will be excluded.	EXC_Language

Peer Review Status	Only reviews published in peer-reviewed journals will be considered; non-peer-reviewed sources will be excluded.	EXC_PeerReview

Table S3. Data extracted for RQ1 with column descriptions.

Column	Description
**Study**	Title of the review study, or first author + short title (e.g., "Smith et al., 2021")
**Year**	Year of publication of the review
**Review Type**	Type of review (e.g., Systematic Review and/or Meta-analysis)
**Number of Included Studies**	Total number of primary studies included in the review
**Population Summary**	Brief description of the patient population (e.g., COPD severity, age range, region)
**Dataset Used**	Names of datasets or data sources used (e.g., COPDGene, ECLIPSE, proprietary hospital data)
**Type of Data Used**	General types of data used (e.g., biosignals, clinical records, patient-reported outcomes)
**Technology / Devices**	Types of technologies or sensors referenced (e.g., wearable devices, apps, telemonitors)
**AI/ML Methods**	Broad categories of AI models used (e.g., machine learning, deep learning)
**Purpose of Prediction**	What the models aimed to predict (e.g., exacerbation, hospitalization, mortality)
**Model Inputs**	Summary of types of features used (e.g., vitals, symptoms, activity, environment)
**Performance Summary**	Any performance metric reported (AUC, accuracy, sensitivity, etc.)
**Validation Type**	Whether internal or external validation was mentioned
**Outcome**	Describes the overall outcomes reported in the review. This includes the number of included studies that focused on specific outcomes (e.g., exacerbation, hospitalization, mortality), any pooled effect estimates (if meta-analysis was done), and a high-level summary of the findings
**Main Challenges / Limitations**	Challenges in modeling or implementation mentioned by the author

Table S4. Data extracted for RQ2 with column descriptions.

Column	Description
**Study**	Title of the review article or shorthand (e.g., first author + abbreviated title).
**Year**	Year the review was published.
**Type of Review**	Indicate whether the review is a systematic review, meta-analysis, or both.
**Number of Included Studies**	Total number of primary studies included in the review.
**Study Design**	Type of studies included in the review: RCT, non-RCT, or both (e.g., if

	the review included both randomized and observational studies).
**P – Population**	Characteristics of the target population (e.g., COPD diagnosis, severity, age range, inclusion/exclusion criteria).
**I – Digital Intervention**	Type of digital health intervention (e.g., mHealth app, telemonitoring, telerehabilitation), its key components (e.g., education, feedback, monitoring), and duration (e.g., 6 weeks, 12 months).
**C – Comparator**	Description of the control or comparison group (e.g., usual care, in-person rehabilitation, educational booklet).
**O – Outcomes**	Summary of all reported outcomes and effects, including:<br>• Primary outcomes (e.g., exacerbation rate, hospital admissions)<br>• Quality of life measures (e.g., SGRQ, CAT, EQ-5D)<br>• Medication adherence (e.g., prescription refill, self-report)<br>• Disease management metrics (e.g., ER visits, activity levels, self-care)<br>Mention instruments used and indicate direction/magnitude of change.
**Statistical Findings**	Key effect estimates for primary outcomes (e.g., SMDs, mean differences, risk ratios), confidence intervals, and p-values.
**Heterogeneity Reported**	Whether heterogeneity was assessed and how (e.g., I^2 value, Q statistic); note any reported sources of heterogeneity or subgroup analyses.
**Main Conclusions**	Summary of the authors' overall conclusions, including whether the intervention was found effective, ineffective, or inconclusive.
**Limitations**	Methodological or contextual limitations noted by the review authors (e.g., small sample sizes, study heterogeneity, risk of bias in included trials).

Table S5. CCA values for RQ1, reflecting the aggregate degree of primary study overlap across included systematic reviews.

Reviews	Overlap Count	N	r	c	CCA Proportion	CCA Percentage
10.1177/14799723166423 vs. 10.1183/16000617.0061-2016	0	47	47	2	0	0
10.1177/14799723166423 vs. 10.1186/s12911-025-02870-7	2	60	58	2	0.03448275862	3.4
10.1177/14799723166423 vs. 10.1186/s12931-024-03033-4	0	67	67	2	0	0
10.1177/14799723166423 vs. 10.2196/52143	5	47	42	2	0.119047619	11.9
10.1183/16000617.0061-2016 vs. 10.1186/s12911-025-02870-7	1	61	60	2	0.01666666667	1.7

10.1183/16000617.0061-2016 vs. 10.1186/s12931-024-03033-4	1	68	67	2	0.01492537313	1.5
10.1183/16000617.0061-2016 vs. 10.2196/52143	0	48	48	2	0	0
10.1186/s12911-025-02870-7 vs. 10.1186/s12931-024-03033-4	1	81	80	2	0.0125	1.2
10.1186/s12911-025-02870-7 vs. 10.2196/52143	8	61	53	2	0.1509433962	15.1
10.1186/s12931-024-03033-4 vs. 10.2196/52143	0	68	68	2	0	0
**Overall**		**152**	**138**	**5**	**0.02536231884**	**2.5**

Table S6. CCA values for RQ2, reflecting the aggregate degree of primary study overlap across included systematic reviews.

Reviews	Overlap Count	N	r	c	CCA Proportion	CCA Percentage
10.1002/14651858.CD011425.pub2 vs. 10.1002/14651858.CD013246.pub2	0	29	29	2	0	0
10.1002/14651858.CD011425.pub2 vs. 10.1007/s10916-024-02135-2	1	32	31	2	0.03225806452	3.2
10.1002/14651858.CD011425.pub2 vs. 10.1016/j.cegh.2023.101427	0	38	38	2	0	0

10.1002/14651858.CD011425.pub2 vs. 10.1016/j.rmed.2014.10.008	0	15	15	2	0	0
10.1002/14651858.CD011425.pub2 vs. 10.1038/s41533-020-0167-1	1	19	18	2	0.05555555556	5.6
10.1002/14651858.CD011425.pub2 vs. 10.1080/15412555.2017.1358257	1	14	13	2	0.07692307692	7.7
10.1002/14651858.CD011425.pub2 vs. 10.1111/cea.14547	0	37	37	2	0	0
10.1002/14651858.CD011425.pub2 vs. 10.1111/jan.15693...10	0	10	10	2	0	0
10.1002/14651858.CD011425.pub2 vs. 10.1111/jan.15693...11	0	9	9	2	0	0
10.1002/14651858.CD011425.pub2 vs. 10.1111/jocn.17225	1	48	47	2	0.02127659574	2.1
10.1002/14651858.CD011425.pub2 vs. 10.1186/s12890-024-03104-8	0	27	27	2	0	0
10.1002/14651858.CD011425.pub2 vs. 10.2196/29475	0	18	18	2	0	0
10.1002/14651858.CD011425.pub2 vs. 10.2196/41753	0	16	16	2	0	0
10.1002/14651858.CD011425.pub2 vs. 10.2196/46439	4	146	142	2	0.02816901408	2.8
10.1002/14651858.CD011425.pub2 vs. 10.2196/76323	1	23	22	2	0.04545454545	4.5

10.1002/14651858.CD011425.pub2 vs. 10.2196/mhealth.5921	0	12	12	2	0	0
10.1002/14651858.CD011425.pub2 vs. 10.32725/kont.2024.024	2	16	14	2	0.1428571429	14.3
10.1002/14651858.CD011425.pub2 vs. 10.3389/fpubh.2024.1488687	0	55	55	2	0	0
10.1002/14651858.CD011425.pub2 vs. 10.3390/arm93020008	1	19	18	2	0.05555555556	5.6
10.1002/14651858.CD011425.pub2 vs. 10.3390/ijerph18136757	0	28	28	2	0	0
10.1002/14651858.CD011425.pub2 vs. 10.3390/ijerph192215165	0	28	28	2	0	0
10.1002/14651858.CD011425.pub2 vs. 10.3390/medicina60060963	1	19	18	2	0.05555555556	5.6
10.1002/14651858.CD013246.pub2 vs. 10.1007/s10916-024-02135-2	1	49	48	2	0.02083333333	2.1
10.1002/14651858.CD013246.pub2 vs. 10.1016/j.cegh.2023.101427	6	55	49	2	0.1224489796	12.2
10.1002/14651858.CD013246.pub2 vs. 10.1016/j.rmed.2014.10.008	3	32	29	2	0.1034482759	10.3
10.1002/14651858.CD013246.pub2 vs. 10.1038/s41533-020-0167-1	3	36	33	2	0.09090909091	9.1

10.1002/14651858.CD013246.pub2 vs. 10.1080/15412555.2017.1358257	0	31	31	2	0	0
10.1002/14651858.CD013246.pub2 vs. 10.1111/cea.14547	1	54	53	2	0.01886792453	1.9
10.1002/14651858.CD013246.pub2 vs. 10.1111/jan.15693...10	1	27	26	2	0.03846153846	3.8
10.1002/14651858.CD013246.pub2 vs. 10.1111/jan.15693...11	1	26	25	2	0.04	4
10.1002/14651858.CD013246.pub2 vs. 10.1111/jocn.17225	4	65	61	2	0.06557377049	6.6
10.1002/14651858.CD013246.pub2 vs. 10.1186/s12890-024-03104-8	0	44	44	2	0	0
10.1002/14651858.CD013246.pub2 vs. 10.2196/29475	2	35	33	2	0.06060606061	6.1
10.1002/14651858.CD013246.pub2 vs. 10.2196/41753	1	33	32	2	0.03125	3.1
10.1002/14651858.CD013246.pub2 vs. 10.2196/46439	3	163	160	2	0.01875	1.9
10.1002/14651858.CD013246.pub2 vs. 10.2196/76323	4	40	36	2	0.1111111111	11.1
10.1002/14651858.CD013246.pub2 vs. 10.2196/mhealth.5921	2	29	27	2	0.07407407407	7.4
10.1002/14651858.CD013246.pub2 vs. 10.32725/kont.2024.024	4	33	29	2	0.1379310345	13.8

10.1002/14651858.CD013246.pub2 vs. 10.3389/fpubh.2024.1488687	1	72	71	2	0.01408450704	1.4
10.1002/14651858.CD013246.pub2 vs. 10.3390/arm93020008	1	36	35	2	0.02857142857	2.9
10.1002/14651858.CD013246.pub2 vs. 10.3390/ijerph18136757	3	45	42	2	0.07142857143	7.1
10.1002/14651858.CD013246.pub2 vs. 10.3390/ijerph192215165	2	45	43	2	0.04651162791	4.7
10.1002/14651858.CD013246.pub2 vs. 10.3390/medicina60060963	2	36	34	2	0.05882352941	5.9
10.1007/s10916-024-02135-2 vs. 10.1016/j.cegh.2023.101427	1	58	57	2	0.01754385965	1.8
10.1007/s10916-024-02135-2 vs. 10.1016/j.rmed.2014.10.008	1	35	34	2	0.02941176471	2.9
10.1007/s10916-024-02135-2 vs. 10.1038/s41533-020-0167-1	1	39	38	2	0.02631578947	2.6
10.1007/s10916-024-02135-2 vs. 10.1080/15412555.2017.1358257	1	34	33	2	0.0303030303	3
10.1007/s10916-024-02135-2 vs. 10.1111/cea.14547	1	57	56	2	0.01785714286	1.8
10.1007/s10916-024-02135-2 vs. 10.1111/jan.15693...10	1	30	29	2	0.03448275862	3.4

10.1007/s10916-024-02135-2 vs. 10.1111/jan.15693...11	1	29	28	2	0.03571428571	3.6
10.1007/s10916-024-02135-2 vs. 10.1111/jocn.17225	1	68	67	2	0.01492537313	1.5
10.1007/s10916-024-02135-2 vs. 10.1186/s12890-024-03104-8	1	47	46	2	0.02173913043	2.2
10.1007/s10916-024-02135-2 vs. 10.2196/29475	1	38	37	2	0.02702702703	2.7
10.1007/s10916-024-02135-2 vs. 10.2196/41753	1	36	35	2	0.02857142857	2.9
10.1007/s10916-024-02135-2 vs. 10.2196/46439	5	166	161	2	0.03105590062	3.1
10.1007/s10916-024-02135-2 vs. 10.2196/76323	1	43	42	2	0.02380952381	2.4
10.1007/s10916-024-02135-2 vs. 10.2196/mhealth.5921	1	32	31	2	0.03225806452	3.2
10.1007/s10916-024-02135-2 vs. 10.32725/kont.2024.024	1	36	35	2	0.02857142857	2.9
10.1007/s10916-024-02135-2 vs. 10.3389/fpubh.2024.1488687	2	75	73	2	0.02739726027	2.7
10.1007/s10916-024-02135-2 vs. 10.3390/arm93020008	1	39	38	2	0.02631578947	2.6
10.1007/s10916-024-02135-2 vs. 10.3390/ijerph18136757	1	48	47	2	0.02127659574	2.1
10.1007/s10916-024-02135-2 vs. 10.3390/ijerph192215165	1	48	47	2	0.02127659574	2.1

10.1007/s10916-024-02135-2 vs. 10.3390/medicina60060963	1	39	38	2	0.02631578947	2.6
10.1016/j.cegh.2023.101427 vs. 10.1016/j.rmed.2014.10.008	0	41	41	2	0	0
10.1016/j.cegh.2023.101427 vs. 10.1038/s41533-020-0167-1	5	45	40	2	0.125	12.5
10.1016/j.cegh.2023.101427 vs. 10.1080/15412555.2017.1358257	3	40	37	2	0.08108108108	8.1
10.1016/j.cegh.2023.101427 vs. 10.1111/cea.14547	0	63	63	2	0	0
10.1016/j.cegh.2023.101427 vs. 10.1111/jan.15693...10	0	36	36	2	0	0
10.1016/j.cegh.2023.101427 vs. 10.1111/jan.15693...11	1	35	34	2	0.02941176471	2.9
10.1016/j.cegh.2023.101427 vs. 10.1111/jocn.17225	7	74	67	2	0.1044776119	10.4
10.1016/j.cegh.2023.101427 vs. 10.1186/s12890-024-03104-8	0	53	53	2	0	0
10.1016/j.cegh.2023.101427 vs. 10.2196/29475	1	44	43	2	0.02325581395	2.3
10.1016/j.cegh.2023.101427 vs. 10.2196/41753	5	42	37	2	0.1351351351	13.5
10.1016/j.cegh.2023.101427 vs. 10.2196/46439	8	172	164	2	0.0487804878	4.9

10.1016/j.cegh.2023.101427 vs. 10.2196/76323	8	49	41	2	0.1951219512	19.5
10.1016/j.cegh.2023.101427 vs. 10.2196/mhealth.5921	2	38	36	2	0.05555555556	5.6
10.1016/j.cegh.2023.101427 vs. 10.32725/kont.2024.024	5	42	37	2	0.1351351351	13.5
10.1016/j.cegh.2023.101427 vs. 10.3389/fpubh.2024.1488687	1	81	80	2	0.0125	1.2
10.1016/j.cegh.2023.101427 vs. 10.3390/arm93020008	5	45	40	2	0.125	12.5
10.1016/j.cegh.2023.101427 vs. 10.3390/ijerph18136757	3	54	51	2	0.05882352941	5.9
10.1016/j.cegh.2023.101427 vs. 10.3390/ijerph192215165	3	54	51	2	0.05882352941	5.9
10.1016/j.cegh.2023.101427 vs. 10.3390/medicina60060963	0	45	45	2	0	0
10.1016/j.rmed.2014.10.008 vs. 10.1038/s41533-020-0167-1	1	22	21	2	0.04761904762	4.8
10.1016/j.rmed.2014.10.008 vs. 10.1080/15412555.2017.1358257	0	17	17	2	0	0
10.1016/j.rmed.2014.10.008 vs. 10.1111/cea.14547	1	40	39	2	0.02564102564	2.6
10.1016/j.rmed.2014.10.008 vs. 10.1111/jan.15693...10	0	13	13	2	0	0

10.1016/j.rmed.2014.10.008 vs. 10.1111/jan.15693...11	1	12	11	2	0.09090909091	9.1
10.1016/j.rmed.2014.10.008 vs. 10.1111/jocn.17225	0	51	51	2	0	0
10.1016/j.rmed.2014.10.008 vs. 10.1186/s12890-024-03104-8	1	30	29	2	0.03448275862	3.4
10.1016/j.rmed.2014.10.008 vs. 10.2196/29475	1	21	20	2	0.05	5
10.1016/j.rmed.2014.10.008 vs. 10.2196/41753	0	19	19	2	0	0
10.1016/j.rmed.2014.10.008 vs. 10.2196/46439	3	149	146	2	0.02054794521	2.1
10.1016/j.rmed.2014.10.008 vs. 10.2196/76323	1	26	25	2	0.04	4
10.1016/j.rmed.2014.10.008 vs. 10.2196/mhealth.5921	0	15	15	2	0	0
10.1016/j.rmed.2014.10.008 vs. 10.32725/kont.2024.024	0	19	19	2	0	0
10.1016/j.rmed.2014.10.008 vs. 10.3389/fpubh.2024.1488687	0	58	58	2	0	0
10.1016/j.rmed.2014.10.008 vs. 10.3390/arm93020008	1	22	21	2	0.04761904762	4.8
10.1016/j.rmed.2014.10.008 vs. 10.3390/ijerph18136757	0	31	31	2	0	0
10.1016/j.rmed.2014.10.008 vs. 10.3390/ijerph192215165	0	31	31	2	0	0

10.1016/j.rmed.2014.10.008 vs. 10.3390/medicina60060963	0	22	22	2	0	0
10.1038/s41533-020-0167-1 vs. 10.1080/15412555.2017.1358257	3	21	18	2	0.1666666667	16.7
10.1038/s41533-020-0167-1 vs. 10.1111/cea.14547	0	44	44	2	0	0
10.1038/s41533-020-0167-1 vs. 10.1111/jan.15693...10	0	17	17	2	0	0
10.1038/s41533-020-0167-1 vs. 10.1111/jan.15693...11	1	16	15	2	0.06666666667	6.7
10.1038/s41533-020-0167-1 vs. 10.1111/jocn.17225	3	55	52	2	0.05769230769	5.8
10.1038/s41533-020-0167-1 vs. 10.1186/s12890-024-03104-8	0	34	34	2	0	0
10.1038/s41533-020-0167-1 vs. 10.2196/29475	2	25	23	2	0.08695652174	8.7
10.1038/s41533-020-0167-1 vs. 10.2196/41753	2	23	21	2	0.09523809524	9.5
10.1038/s41533-020-0167-1 vs. 10.2196/46439	5	153	148	2	0.03378378378	3.4
10.1038/s41533-020-0167-1 vs. 10.2196/76323	2	30	28	2	0.07142857143	7.1
10.1038/s41533-020-0167-1 vs. 10.2196/mhealth.5921	3	19	16	2	0.1875	18.8
10.1038/s41533-020-0167-1 vs. 10.32725/kont.2024.024	3	23	20	2	0.15	15

10.1038/s41533-020-0167-1 vs. 10.3389/fpubh.2024.1488687	0	62	62	2	0	0
10.1038/s41533-020-0167-1 vs. 10.3390/arm93020008	5	26	21	2	0.2380952381	23.8
10.1038/s41533-020-0167-1 vs. 10.3390/ijerph18136757	3	35	32	2	0.09375	9.4
10.1038/s41533-020-0167-1 vs. 10.3390/ijerph192215165	2	35	33	2	0.06060606061	6.1
10.1038/s41533-020-0167-1 vs. 10.3390/medicina60060963	1	26	25	2	0.04	4
10.1080/15412555.2017.1358257 vs. 10.1111/cea.14547	0	39	39	2	0	0
10.1080/15412555.2017.1358257 vs. 10.1111/jan.15693...10	0	12	12	2	0	0
10.1080/15412555.2017.1358257 vs. 10.1111/jan.15693...11	0	11	11	2	0	0
10.1080/15412555.2017.1358257 vs. 10.1111/jocn.17225	0	50	50	2	0	0
10.1080/15412555.2017.1358257 vs. 10.1186/s12890-024-03104-8	0	29	29	2	0	0
10.1080/15412555.2017.1358257 vs. 10.2196/29475	0	20	20	2	0	0
10.1080/15412555.2017.1358257 vs. 10.2196/41753	2	18	16	2	0.125	12.5
10.1080/15412555.2017.1358257 vs. 10.2196/46439	3	148	145	2	0.02068965517	2.1

10.1080/15412555.2017.1358257 vs. 10.2196/76323	0	25	25	2	0	0
10.1080/15412555.2017.1358257 vs. 10.2196/mhealth.5921	1	14	13	2	0.07692307692	7.7
10.1080/15412555.2017.1358257 vs. 10.32725/kont.2024.024	1	18	17	2	0.05882352941	5.9
10.1080/15412555.2017.1358257 vs. 10.3389/fpubh.2024.1488687	0	57	57	2	0	0
10.1080/15412555.2017.1358257 vs. 10.3390/arm93020008	3	21	18	2	0.1666666667	16.7
10.1080/15412555.2017.1358257 vs. 10.3390/ijerph18136757	0	30	30	2	0	0
10.1080/15412555.2017.1358257 vs. 10.3390/ijerph192215165	1	30	29	2	0.03448275862	3.4
10.1080/15412555.2017.1358257 vs. 10.3390/medicina60060963	1	21	20	2	0.05	5
10.1111/cea.14547 vs. 10.1111/jan.15693...10	0	35	35	2	0	0
10.1111/cea.14547 vs. 10.1111/jan.15693...11	0	34	34	2	0	0
10.1111/cea.14547 vs. 10.1111/jocn.17225	3	73	70	2	0.04285714286	4.3
10.1111/cea.14547 vs. 10.1186/s12890-024-03104-8	0	52	52	2	0	0
10.1111/cea.14547 vs. 10.2196/29475	0	43	43	2	0	0

10.1111/cea.14547 vs. 10.2196/41753	0	41	41	2	0	0
10.1111/cea.14547 vs. 10.2196/46439	3	171	168	2	0.01785714286	1.8
10.1111/cea.14547 vs. 10.2196/76323	2	48	46	2	0.04347826087	4.3
10.1111/cea.14547 vs. 10.2196/mhealth.5921	0	37	37	2	0	0
10.1111/cea.14547 vs. 10.32725/kont.2024.024	1	41	40	2	0.025	2.5
10.1111/cea.14547 vs. 10.3389/fpubh.2024.1488687	0	80	80	2	0	0
10.1111/cea.14547 vs. 10.3390/arm93020008	0	44	44	2	0	0
10.1111/cea.14547 vs. 10.3390/ijerph18136757	7	53	46	2	0.152173913	15.2
10.1111/cea.14547 vs. 10.3390/ijerph192215165	0	53	53	2	0	0
10.1111/cea.14547 vs. 10.3390/medicina60060963	0	44	44	2	0	0
10.1111/jan.15693...10 vs. 10.1111/jan.15693...11	0	7	7	2	0	0
10.1111/jan.15693...10 vs. 10.1111/jocn.17225	2	46	44	2	0.04545454545	4.5
10.1111/jan.15693...10 vs. 10.1186/s12890-024-03104-8	0	25	25	2	0	0

10.1111/jan.15693...10 vs. 10.2196/29475	0	16	16	2	0	0
10.1111/jan.15693...10 vs. 10.2196/41753	0	14	14	2	0	0
10.1111/jan.15693...10 vs. 10.2196/46439	4	144	140	2	0.02857142857	2.9
10.1111/jan.15693...10 vs. 10.2196/76323	0	21	21	2	0	0
10.1111/jan.15693...10 vs. 10.2196/mhealth.5921	0	10	10	2	0	0
10.1111/jan.15693...10 vs. 10.32725/kont.2024.024	0	14	14	2	0	0
10.1111/jan.15693...10 vs. 10.3389/fpubh.2024.1488687	0	53	53	2	0	0
10.1111/jan.15693...10 vs. 10.3390/arm93020008	0	17	17	2	0	0
10.1111/jan.15693...10 vs. 10.3390/ijerph18136757	0	26	26	2	0	0
10.1111/jan.15693...10 vs. 10.3390/ijerph192215165	1	26	25	2	0.04	4
10.1111/jan.15693...10 vs. 10.3390/medicina60060963	1	17	16	2	0.0625	6.2
10.1111/jan.15693...11 vs. 10.1111/jocn.17225	1	45	44	2	0.02272727273	2.3
10.1111/jan.15693...11 vs. 10.1186/s12890-024-03104-8	0	24	24	2	0	0

10.1111/jan.15693...11 vs. 10.2196/29475	1	15	14	2	0.07142857143	7.1
10.1111/jan.15693...11 vs. 10.2196/41753	0	13	13	2	0	0
10.1111/jan.15693...11 vs. 10.2196/46439	3	143	140	2	0.02142857143	2.1
10.1111/jan.15693...11 vs. 10.2196/76323	0	20	20	2	0	0
10.1111/jan.15693...11 vs. 10.2196/mhealth.5921	1	9	8	2	0.125	12.5
10.1111/jan.15693...11 vs. 10.32725/kont.2024.024	0	13	13	2	0	0
10.1111/jan.15693...11 vs. 10.3389/fpubh.2024.1488687	0	52	52	2	0	0
10.1111/jan.15693...11 vs. 10.3390/arm93020008	1	16	15	2	0.06666666667	6.7
10.1111/jan.15693...11 vs. 10.3390/ijerph18136757	1	25	24	2	0.04166666667	4.2
10.1111/jan.15693...11 vs. 10.3390/ijerph192215165	0	25	25	2	0	0
10.1111/jan.15693...11 vs. 10.3390/medicina60060963	0	16	16	2	0	0
10.1111/jocn.17225 vs. 10.1186/s12890-024-03104-8	7	63	56	2	0.125	12.5
10.1111/jocn.17225 vs. 10.2196/29475	2	54	52	2	0.03846153846	3.8

10.1111/jocn.17225 vs. 10.2196/41753	5	52	47	2	0.1063829787	10.6
10.1111/jocn.17225 vs. 10.2196/46439	11	182	169	2	0.07692307692	7.7
10.1111/jocn.17225 vs. 10.2196/76323	7	59	52	2	0.1346153846	13.5
10.1111/jocn.17225 vs. 10.2196/mhealth.5921	2	48	46	2	0.04347826087	4.3
10.1111/jocn.17225 vs. 10.32725/kont.2024.024	6	52	46	2	0.1304347826	13
10.1111/jocn.17225 vs. 10.3389/fpubh.2024.1488687	0	91	91	2	0	0
10.1111/jocn.17225 vs. 10.3390/arm93020008	2	55	53	2	0.03773584906	3.8
10.1111/jocn.17225 vs. 10.3390/ijerph18136757	4	64	60	2	0.06666666667	6.7
10.1111/jocn.17225 vs. 10.3390/ijerph192215165	10	64	54	2	0.1851851852	18.5
10.1111/jocn.17225 vs. 10.3390/medicina60060963	6	55	49	2	0.1224489796	12.2
10.1186/s12890-024-03104-8 vs. 10.2196/29475	0	33	33	2	0	0
10.1186/s12890-024-03104-8 vs. 10.2196/41753	0	31	31	2	0	0
10.1186/s12890-024-03104-8 vs. 10.2196/46439	9	161	150	2	0.07333333333	7.3

10.1186/s12890-024-03104-8 vs. 10.2196/76323	1	38	37	2	0.02702702703	2.7
10.1186/s12890-024-03104-8 vs. 10.2196/mhealth.5921	0	27	27	2	0	0
10.1186/s12890-024-03104-8 vs. 10.32725/kont.2024.024	0	31	31	2	0	0
10.1186/s12890-024-03104-8 vs. 10.3389/fpubh.2024.1488687	0	70	70	2	0	0
10.1186/s12890-024-03104-8 vs. 10.3390/arm93020008	1	34	33	2	0.0303030303	3
10.1186/s12890-024-03104-8 vs. 10.3390/ijerph18136757	0	43	43	2	0	0
10.1186/s12890-024-03104-8 vs. 10.3390/ijerph192215165	6	43	37	2	0.1621621622	16.2
10.1186/s12890-024-03104-8 vs. 10.3390/medicina60060963	4	34	30	2	0.1333333333	13.3
10.2196/29475 vs. 10.2196/41753	0	22	22	2	0	0
10.2196/29475 vs. 10.2196/46439	3	152	149	2	0.02013422819	2
10.2196/29475 vs. 10.2196/76323	1	29	28	2	0.03571428571	3.6
10.2196/29475 vs. 10.2196/mhealth.5921	0	18	18	2	0	0
10.2196/29475 vs. 10.32725/kont.2024.024	1	22	21	2	0.04761904762	4.8

10.2196/29475 vs. 10.3389/fpubh.2024.1488687	0	61	61	2	0	0
10.2196/29475 vs. 10.3390/arm93020008	0	25	25	2	0	0
10.2196/29475 vs. 10.3390/ijerph18136757	2	34	32	2	0.0625	6.2
10.2196/29475 vs. 10.3390/ijerph192215165	0	34	34	2	0	0
10.2196/29475 vs. 10.3390/medicina60060963	0	25	25	2	0	0
10.2196/41753 vs. 10.2196/46439	6	150	144	2	0.04166666667	4.2
10.2196/41753 vs. 10.2196/76323	2	27	25	2	0.08	8
10.2196/41753 vs. 10.2196/mhealth.5921	0	16	16	2	0	0
10.2196/41753 vs. 10.32725/kont.2024.024	2	20	18	2	0.1111111111	11.1
10.2196/41753 vs. 10.3389/fpubh.2024.1488687	0	59	59	2	0	0
10.2196/41753 vs. 10.3390/arm93020008	1	23	22	2	0.04545454545	4.5
10.2196/41753 vs. 10.3390/ijerph18136757	0	32	32	2	0	0
10.2196/41753 vs. 10.3390/ijerph192215165	4	32	28	2	0.1428571429	14.3

10.2196/41753 vs. 10.3390/medicina60060963	1	23	22	2	0.04545454545	4.5
10.2196/46439 vs. 10.2196/76323	7	157	150	2	0.04666666667	4.7
10.2196/46439 vs. 10.2196/mhealth.5921	3	146	143	2	0.02097902098	2.1
10.2196/46439 vs. 10.32725/kont.2024.024	6	150	144	2	0.04166666667	4.2
10.2196/46439 vs. 10.3389/fpubh.2024.1488687	9	189	180	2	0.05	5
10.2196/46439 vs. 10.3390/arm93020008	5	153	148	2	0.03378378378	3.4
10.2196/46439 vs. 10.3390/ijerph18136757	3	162	159	2	0.01886792453	1.9
10.2196/46439 vs. 10.3390/ijerph192215165	7	162	153	2	0.05882352941	5.9
10.2196/46439 vs. 10.3390/medicina60060963	5	153	146	2	0.04794520548	4.8
10.2196/76323 vs. 10.2196/mhealth.5921	1	23	22	2	0.04545454545	4.5
10.2196/76323 vs. 10.32725/kont.2024.024	5	27	22	2	0.2272727273	22.7
10.2196/76323 vs. 10.3389/fpubh.2024.1488687	1	66	65	2	0.01538461538	1.5
10.2196/76323 vs. 10.3390/arm93020008	3	30	27	2	0.1111111111	11.1

10.2196/76323 vs. 10.3390/ijerph18136757	3	39	36	2	0.08333333333	8.3
10.2196/76323 vs. 10.3390/ijerph192215165	2	39	37	2	0.05405405405	5.4
10.2196/76323 vs. 10.3390/medicina60060963	1	30	29	2	0.03448275862	3.4
10.2196/mhealth.5921 vs. 10.32725/kont.2024.024	1	16	15	2	0.06666666667	6.7
10.2196/mhealth.5921 vs. 10.3389/fpubh.2024.1488687	0	55	55	2	0	0
10.2196/mhealth.5921 vs. 10.3390/arm93020008	2	19	17	2	0.1176470588	11.8
10.2196/mhealth.5921 vs. 10.3390/ijerph18136757	1	28	27	2	0.03703703704	3.7
10.2196/mhealth.5921 vs. 10.3390/ijerph192215165	0	28	28	2	0	0
10.2196/mhealth.5921 vs. 10.3390/medicina60060963	1	19	18	2	0.05555555556	5.6
10.32725/kont.2024.024 vs. 10.3389/fpubh.2024.1488687	1	59	58	2	0.01724137931	1.7
10.32725/kont.2024.024 vs. 10.3390/arm93020008	3	23	20	2	0.15	15
10.32725/kont.2024.024 vs. 10.3390/ijerph18136757	3	32	29	2	0.1034482759	10.3
10.32725/kont.2024.024 vs. 10.3390/ijerph192215165	1	32	31	2	0.03225806452	3.2

10.32725/kont.2024.024 vs. 10.3390/medicina60060963	2	23	21	2	0.09523809524	9.5
10.3389/fpubh.2024.1488687 vs. 10.3390/arm93020008	1	62	61	2	0.01639344262	1.6
10.3389/fpubh.2024.1488687 vs. 10.3390/ijerph18136757	1	71	70	2	0.01428571429	1.4
10.3389/fpubh.2024.1488687 vs. 10.3390/ijerph192215165	0	71	71	2	0	0
10.3389/fpubh.2024.1488687 vs. 10.3390/medicina60060963	0	62	62	2	0	0
10.3390/arm93020008 vs. 10.3390/ijerph18136757	2	35	33	2	0.06060606061	6.1
10.3390/arm93020008 vs. 10.3390/ijerph192215165	2	35	33	2	0.06060606061	6.1
10.3390/arm93020008 vs. 10.3390/medicina60060963	2	26	24	2	0.08333333333	8.3
10.3390/ijerph18136757 vs. 10.3390/ijerph192215165	0	44	44	2	0	0
10.3390/ijerph18136757 vs. 10.3390/medicina60060963	0	35	35	2	0	0
10.3390/ijerph192215165 vs. 10.3390/medicina60060963	6	35	29	2	0.2068965517	20.7
**Overall**		**532**	**359**	**23**	**0.02190427956**	**2.2**

Table S10. Complete database search strings used in the literature search for PubMed, Scopus, and Web of Science.

	RQ1	RQ2
**PubMed**	(("pulmonary disease, chronic obstructive"[MeSH Terms] OR "chronic obstructive pulmonary disease"[Title/Abstract] OR "COPD"[Title/Abstract]) AND ("machine learning"[MeSH Terms] OR "machine learning"[Title/Abstract] OR "artificial intelligence"[MeSH Terms] OR "artificial intelligence"[Title/Abstract] OR "deep learning"[Title/Abstract]) AND ("systematic review"[Publication Type] OR "meta-analysis"[Publication Type] OR "systematic review"[Title/Abstract] OR "meta-analysis"[Title/Abstract] OR "review"[Title/Abstract]) AND ("wearable"[Title/Abstract] OR "wearable sensor"[Title/Abstract] OR "wearable device"[Title/Abstract] OR "biosignal"[Title/Abstract] OR "physiological signal"[Title/Abstract] OR "vital sign"[Title/Abstract] OR ("digital health"[Title/Abstract] OR "telemedicine"[Title/Abstract] OR "remote monitoring"[Title/Abstract]))) AND (meta-analysis[Filter] OR systematicreview[Filter])	(("Pulmonary Disease, Chronic Obstructive"[Mesh] OR "chronic obstructive pulmonary disease"[tiab] OR COPD[tiab])<br>AND<br>("digital health"[tiab] OR "mHealth"[tiab] OR "eHealth"[tiab] OR "telehealth"[tiab] OR "telemedicine"[Mesh] OR "wearable device"[tiab] OR "remote monitoring"[tiab] OR "digital intervention"[tiab] OR "digital therapeutics"[tiab] OR "dTx"[tiab])<br>AND<br>("disease management"[tiab] OR "self-management"[tiab] OR "quality of life"[tiab] OR "medication adherence"[tiab])<br>AND<br>("effectiveness"[tiab] OR "impact"[tiab] OR "outcomes"[tiab])<br>AND<br>("standard care"[tiab] OR "usual care"[tiab] OR "conventional care"[tiab])<br>AND<br>("systematic review"[Publication Type] OR "meta-analysis"[Publication Type] OR "systematic review"[tiab] OR "meta-analysis"[tiab] OR review[tiab]))
**Scopus**	(TITLE-ABS-KEY ("chronic obstructive pulmonary disease" OR copd) AND TITLE-ABS-KEY (exacerbation OR "acute episode") AND TITLE-ABS-KEY (predict* OR forecast* OR "early detection") AND TITLE-ABS-KEY ("machine learning" OR "artificial intelligence" OR "deep learning") AND TITLE-ABS-KEY (systematic OR meta)) AND PUBYEAR > 2015 AND PUBYEAR < 2026	(TITLE-ABS-KEY ("chronic obstructive pulmonary disease" OR copd) AND ("digital health" OR mhealth OR ehealth OR telehealth OR "wearable device*" OR "remote monitoring" OR dtx OR "digital intervention*" OR "digital therapeutic*") AND ("disease management" OR "self-management" OR "quality of life" OR "medication adherence") AND (effectiveness OR impact OR outcomes) AND ("systematic review" OR "meta analysis" OR "literature review")) AND PUBYEAR > 2015 AND PUBYEAR < 2026 AND (LIMIT-TO (DOCTYPE , "re")) AND (LIMIT-TO (LANGUAGE , "English"))
**WoS**	TS=(("COPD" OR "chronic obstructive pulmonary disease")<br>AND ("exacerbation" OR "acute episode")<br>AND ("prediction" OR "forecasting" OR "early detection")<br>AND ("machine learning" OR "artificial intelligence" OR "deep learning")<br>AND ("systematic review" OR "meta-analysis" OR "review"))	TS = (("digital health" OR mHealth OR eHealth OR telehealth OR "wearable device*" OR "remote monitoring" OR dtx OR "digital intervention*" OR "digital therapeutic*")<br>AND<br>("chronic obstructive pulmonary disease" OR COPD)<br>AND<br>("disease management" OR "self-management" OR "quality of life" OR "medication adherence")<br>AND<br>(effectiveness OR impact OR outcomes)<br>AND<br>("standard care" OR "usual care" OR "conventional care")<br>AND<br>("systematic review" OR "meta-analysis" OR review))
